# Bacterial communities in penile skin, male urethra, and vaginas of heterosexual couples with and without bacterial vaginosis

**DOI:** 10.1186/s40168-016-0161-6

**Published:** 2016-04-19

**Authors:** Marcela Zozaya, Michael J. Ferris, Julia D. Siren, Rebecca Lillis, Leann Myers, M. Jacques Nsuami, A. Murat Eren, Jonathan Brown, Christopher M. Taylor, David H. Martin

**Affiliations:** Children’s Hospital of New Orleans, 200 Henry Clay Ave., New Orleans, LA 70118 USA; Department of Pediatrics, Louisiana State University Health Sciences Center, 1901 Perdido St., New Orleans, LA 70112 USA; Department of Internal Medicine, Louisiana State University Health Sciences Center, 1542 Tulane Ave., New Orleans, LA 70112 USA; Department of Biostatistics and Bioinformatics, Tulane School of Public Health and Tropical Medicine, 1440 Canal St., New Orleans, LA 70112 USA; Marine Biological Laboratory, JBPC, 7 MBL St., Woods Hole, MA 02543 USA; Rally Software, 3333 Walnut St., Boulder, CO 80301 USA; Department of Microbiology Immunology and Parasitology, Louisiana State University Health Sciences Center, 1901 Perdido St., New Orleans, LA 70112 USA

**Keywords:** Bacterial vaginosis, Microbiome, Sexual transmission, Penile skin, Urethra, Vagina

## Abstract

**Background:**

The epidemiology of bacterial vaginosis (BV) suggests it is sexually transmissible, yet no transmissible agent has been identified. It is probable that BV-associated bacterial communities are transferred from male to female partners during intercourse; however, the microbiota of sexual partners has not been well-studied.

**Results:**

Pyrosequencing analysis of PCR-amplified 16S rDNA was used to examine BV-associated bacteria in monogamous couples with and without BV using vaginal, male urethral, and penile skin specimens. The penile skin and urethral microbiota of male partners of women with BV was significantly more similar to the vaginal microbiota of their female partner compared to the vaginal microbiota of non-partner women with BV. This was not the case for male partners of women with normal vaginal microbiota. Specific BV-associated species were concordant in women with BV and their male partners.

**Conclusions:**

In monogamous heterosexual couples in which the woman has BV, the significantly higher similarity between the vaginal microbiota and the penile skin and urethral microbiota of the male partner, supports the hypothesis that sexual exchange of BV-associated bacterial taxa is common.

**Electronic supplementary material:**

The online version of this article (doi:10.1186/s40168-016-0161-6) contains supplementary material, which is available to authorized users.

## Background

Bacterial vaginosis (BV) is a leading cause of vaginal infection [1] and the condition is associated with serious sequelae including increased risk of HIV transmission [2–4], adverse pregnancy outcomes [5–7], and acquisition of sexually transmitted infections (STIs) [8]. Moreover, antibiotic treatment success rates are low and recurrence rates are high [9–11]. BV is currently thought to be due to an ecological imbalance in the composition of the vaginal microbiota rather than a classic infection caused by a specific pathogen. BV is characterized by a decrease in the abundance of vaginal *Lactobacillus* species, which are generally regarded as beneficial, and an increase in non-*Lactobacillus* species that include *Gardnerella vaginalis*, *Atopobium vaginae*, *Megasphaera spp.*, *Sneathia spp.*, *Prevotella spp.*, and many others [12–21]. Over the past decade, studies using genetic sequencing analysis of 16S rRNA genes PCR-amplified from vaginal DNA specimens have dramatically increased the number of bacterial species known to inhabit the vaginal environment, many of which have been shown to be highly associated with BV [12–21].

Despite decades of study and considerable evidence suggesting that BV may be sexually transmitted, this hypothesis has not been accepted universally [22–25]. The most direct evidence for sexual transmission comes from a study of healthy women who developed BV after being inoculated with vaginal secretions from women with BV [26]. Studies of lesbian women also suggest that exchange of vaginal secretions during sexual encounters transmits BV [27]. Large studies of young sexually experienced and inexperienced women have shown that BV is uncommon in inexperienced women [28, 29] and that BV-associated bacteria are absent or rare in sexually unexposed women [30]. Additionally, there is evidence that shows an epidemiological association between heterosexual intercourse and BV [31, 32]. Moreover, *G. vaginalis* and other potential BV-causative bacteria can be cultivated from the male urethra, the glans penis, the coronal sulcus, and the prepuce [33, 34]. Recent studies employing cultivation-independent analyses confirm that many BV-associated species can also be detected in male penile skin, semen, urethral, and urine specimens [35–39]. A recent paper [40] shows that among uncircumcised males, the presence of BV-like flora in the coronal sulcus skin specimens correlates with BV diagnosed by the Nugent score in their sex partners. Based on these studies and our previous study which showed a high degree of similarity between *G. vaginalis* oligotypes in heterosexual couples [41], it seems probable that sexual exchange of BV-associated bacteria between male and female partners does occur. Such an exchange could potentially induce a change in the composition and abundance of vaginal species leading to BV. Here, we use a pyrosequencing analysis of 16S rRNA genes to examine the diversity, community composition, prevalence, and relative abundance of genital bacteria in monogamous couples. Clinical samples included vaginal specimens of women with Nugent and Amsel score-defined BV and normal vaginal microbiota, as well as penile skin and urethral specimens of their male sexual partners.

## Results

### Patient population, behavioral and sexually transmitted infection risk factors

The mean age of men and women in the BV-couples group (BV-couples) was 30.7 years for men (SD+/−9.6) and 27.6 years for women (SD+/−6.9). The mean age of men and women in the normal-couples group (normal-couples) was similar to that of the BV-couples (Table 1). The study population was predominantly African-American in both the normal and BV-couples. Based on independent reporting, the mean duration of the relationship between couples ranged from 39.8 to 46.9 months, and this interval did not differ significantly between BV and normal-couples. A past history of STIs, and other STI risk factors, did not differ between normal and BV-couples. Only 54 % of men in the BV-couples group (BV-males) were circumcised, compared to 74 % of men in the normal-couples group (normal-males) (*P* = 0.057).

**Table 1:** Demographics and STI risk factors in couples in which the female does or does not have bacterial vaginosis (BV)

	*Women*			*Men*		
	BV (*n* = 65)	Normal (*n* = 31)	*P* value^a^	BV (*n* = 65)	Normal (*n* = 65)	*P* value^a^
Mean age, years ± SD	27.3 ± 6.6	28.4 ± 7.7	0.473	30.0 ± 8.4	32.2 ± 12.0	0.295
African-American, *n* (%)	57 (88 %)	22 (71 %)	0.045	55 (85 %)	24 (77 %)	0.388
Married, *n* (%)	10 (15 %)	5 (16 %)	0.688	10 (15 %)	5 (16 %)	0.451
History of STD						
Gonorrhea, *n* (%)	17 (26 %)	7 (23 %)	0.705	17 (26 %)	8 (26 %)	0.971
Chlamydia, *n* (%)	29 (45 %)	8 (26 %)	0.077	20 (31 %)	6 (19 %)	0.239
Trichomonas, *n* (%)	19 (29 %)	9 (29 %)	0.984	2 (3 %)	1 (3 %)	0.969
Number of sex partners						
≥2 in last 12 months, *n* (%)	27 (42 %)	12 (39 %)	0.792	34 (52 %)	18 (58 %)	0.597
≥2 in last 60 days, *n* (%)	2 (3 %)	2 (7 %)	0.592	5 (8 %)	4 (13 %)	0.413
Mean duration of partnership^b^, months	39.8	44.7	0.654	46.9	46.3	0.963
Mean number of sexual acts/month^b^						
Vaginal intercourse	11.3	12.0	0.683	11.9	14.3	0.406
Received oral sex	6.0	5.0	0.581	5.7	7.7	0.477
Performed oral sex	4.5	4.7	0.920	4.8	7.6	0.294
Anal intercourse in last month, *n* (%)	12 (19 %)	5 (17 %)	0.832	10 (16 %)	3 (10 %)	0.535
Never or rarely use condoms^b^, *n* (%)	44 (68 %)	19 (61 %)	0.683	40 (62 %)	20 (65 %)	0.935
Circumcised, *n* (%)	–	–	–	35 (54 %)	23 (74 %)	0.057

^a^ *P* values are by Pearson’s chi-square of Fisher’s Exact tests for categorical variables and by *t* tests for continuous variables

^b^With enrolled partner

*SD* standard deviation, *STD* sexually transmitted disease

Women in the BV-couples group (BV-women) were more likely to report a past diagnosis of BV compared to women in the normal-couples group (normal-women) (80 vs. 58 %, *P* = 0.024) (Table 2). The mean Amsel score was 3.64 (SD+/−0.764) for BV-women and 0.28 (SD+/−0.797) for normal-women. Consistent with the diagnosis of BV, significantly more BV-women complained of vaginal discharge and odor (68 and 72 %, respectively) compared to normal-women. BV-women were found to have significantly higher amounts of vaginal secretions on examination compared to normal-women. None of the other variables measured differed between BV and normal-women (Table 2). Additional file 1: Table S1 and Additional file 2: Table S2 show the metadata used to perform these analyses.

**Table 2:** Medical histories and clinical findings in BV and normal-women

	*Women*		
	BV (*n* = 65)	Normal (*n* = 31)	*P* value^a^
Past history of BV, *n* (%)	52 (80 %)	18 (58 %)	0.024
Mean months since last episode of BV	9.7	5.9	0.332
History of douching, *n* (%)	22 (34 %)	9 (30 %)^b^	0.710
History of vaginal products or medications used, *n* (%)	13 (20 %)	9 (29 %)	0.345
Use of birth control, *n* (%)	26 (40 %)	14 (45 %)	0.631
Mean number of days since beginning of last menstrual period	25.7	23.5	0.824
Symptoms			
Vaginal discharge, *n* (%)	44 (68 %)	12 (39 %)	0.007
Vaginal odor, *n* (%)	47 (72 %)	7 (23 %)	<0.0001
Vaginal irritation, *n* (%)	17 (26 %)	5 (16 %)	0.275
Amount of discharge on examination			
None, scant, *n* (%)	24 (38 %)	25 (81 %)	<0.0001
Moderate, *n* (%)	31 (48 %)	6 (19 %)	
Copious, *n* (%)	9 (14 %)	0 (0 %)	
Mean Amsel score	3.64	0.28	<0.0001
Mean Nugent score	8.75	0.87	<0.0001

^a^ *P* values are by Pearson’s chi-square for categorical variables and by *t* tests for continuous variables

^b^Calculation based on *n* = 30

### Bacterial diversity in normal and BV specimens

As expected, the vaginal bacterial diversity of BV-women was significantly higher than that of normal-women, irrespective of the diversity metric used (*P* = 0.001) (Table 3). Likewise, the penile skin diversity of BV-males was significantly higher (*P* ≤ 0.038) than that of normal-males. However, the difference in penile skin bacterial diversity between BV-males and normal-males was not as great as the difference observed between BV-women and normal-women. In contrast to penile skin and vaginal specimens, the bacterial diversity of urethral specimens did not differ between BV and normal-males (Table 3).

**Table 3:** Microbial diversity of normal and BV vaginal, penile skin, and urethral specimens

Specimen type	Diversity estimator	BV mean (SD)	Normal mean (SD)	*t* stat	*P* value
*Vaginal*	Chao1	27.4 (9.3)	11.8 (5.9)	8.475	0.001
*Vaginal*	Observed species	23.2 (7.8)	9.6 (3.5)	9.191	0.001
*Vaginal*	PD whole tree	3.1 (0.7)	1.4 (0.6)	12.115	0.001
*Penile skin*	Chao1	38.5 (14.7)	31.1 (11.3)	2.189	0.038
*Penile skin*	Observed species	28.3 (10.4)	23.2 (7.5)	2.171	0.032
*Penile skin*	PD whole tree	2.8 (0.9)	2.3 (0.8)	2.284	0.018
*Male urethra*	Chao1	34.7 (26.3)	34.7 (25.3)	0.007	0.995
*Male urethra*	Observed species	24.6 (17.7)	26.9 (21.4)	−0.530	0.590
*Male urethra*	PD whole tree	3.0 (1.5)	3.0 (1.7)	0.015	0.987

*SD* standard deviation

The relative abundance of BV-associated bacteria was significantly higher in women with BV compared to normal-women (Additional file 3: Table S3). Moreover, many of these BV-associated bacteria were significantly more abundant in penile skin specimens and urethral specimens of BV-males, compared to normal-males.

### Effect of monogamous sexual partnerships on genital bacterial community composition

**Fig. 1:**
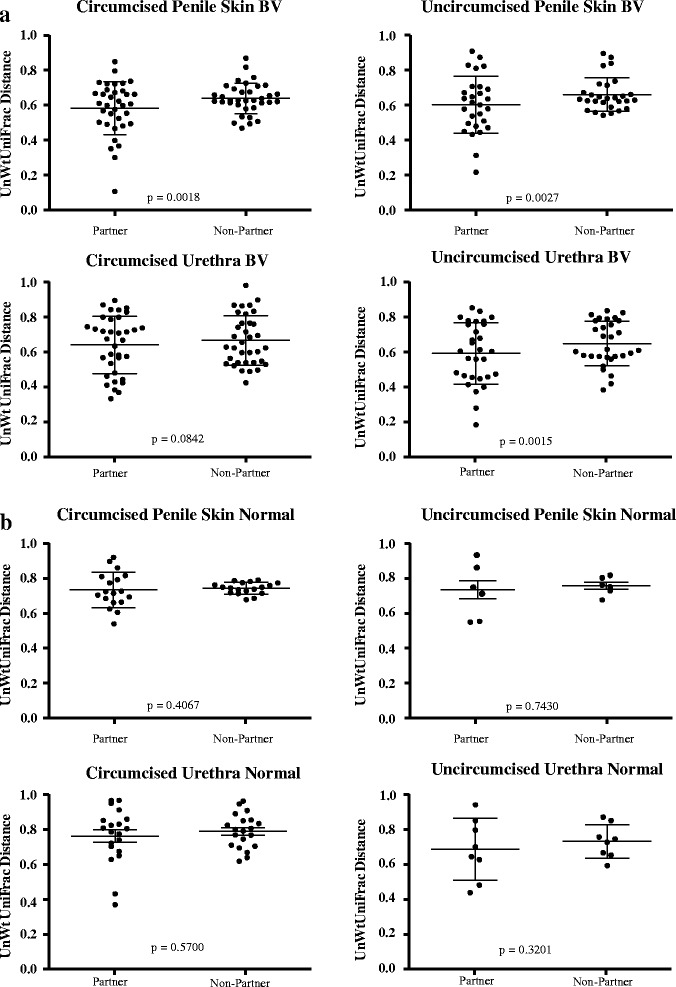
Comparison of unweighted UniFrac distances between the microbiota of sexual partners and non-partners. **a** BV-couples and **b** normal-couples. The analyses were stratified by the circumcision status of the male partners and include penile skin and male urethral specimens as indicated. *P* values are from paired *t* tests

We examined the influence of monogamous sexual partnerships on the composition of genital microbiota within couples (Fig. 1). The results showed that in the case of BV-couples, the penile skin communities of BV-males were significantly more similar to the vaginal communities of their sexual partner, compared to the other (non-partner) women in the study. This was true regardless of whether the BV-males were circumcised (*P* = 0.0018) or uncircumcised (*P* = 0.0027) (Fig. 1a). In the case of BV male urethral specimens, only the microbiota of uncircumcised BV-males was significantly more similar to the vaginal microbiota of their partner (*P* = 0.0015) (Fig. 1). Finally, among normal-couples, neither the penile skin nor the male urethral microbiota were more similar among sexual partners compared to non-partners (Fig. 1b).

**Fig. 2:**
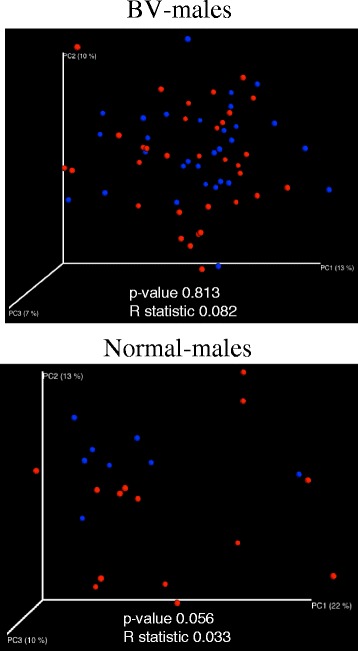
Principal coordinates analysis (PCoA) comparing unweighted UniFrac distances between the microbiota of circumcised (*red*) and uncircumcised (*blue*) penile skin specimens from males whose partners have BV or normal vaginal microbiota. ANOSIM *P* values and *R* statistic are indicated

Principal coordinate analyses showed no clear separation between the penile skin microbiota of circumcised and uncircumcised men, for both BV-males and normal-males (Fig. 2).

### Correlation between vaginal OTUs and penile skin and urethral OTUs of sexual partners

Spearman correlation coefficients were used to investigate associations between the most common OTUs in the vagina of BV-women and normal-women and the same OTUs in the penile skin and urethral specimens of their sexual partners (Tables 4 and 5). The majority of OTUs in vaginal and penile skin specimens of BV-couples showed a strong positive correlation. Correlations were only modestly weaker between vaginal and male urethral specimens in the BV-couples. In contrast, correlations among normal-couples were strikingly lower.

**Table 4:**
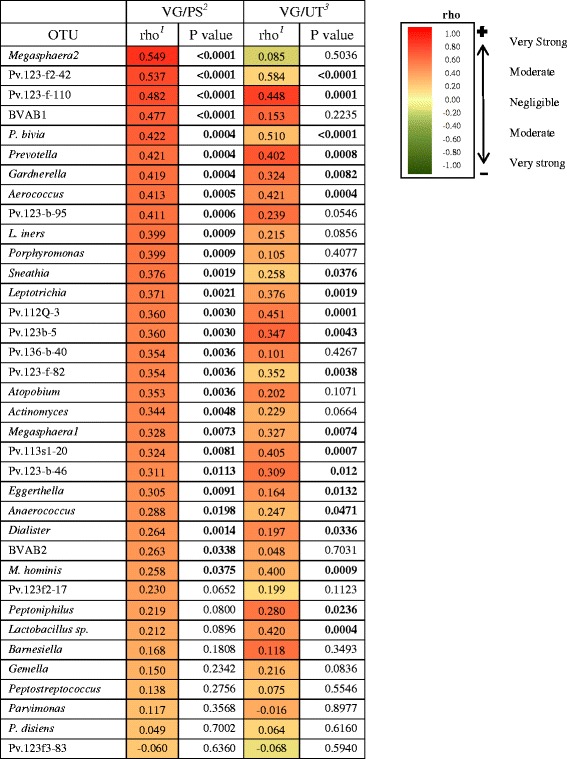
Correlation of specific OTUs* between vagina, penile skin, and male urethra from BV-couples

^1^Spearman correlation coefficient. The *gradient bar* on the right shows the strength of the correlations

Positive correlations (*red*); negative correlations (*green*)

^2^Associations between vagina (VG) and penile skin (PS)

^3^Associations between vagina and male urethra (UT)

*Only those vaginal OTUs which had a prevalence of ≥30 % among BV-women were included in this analysis

**Table 5:**
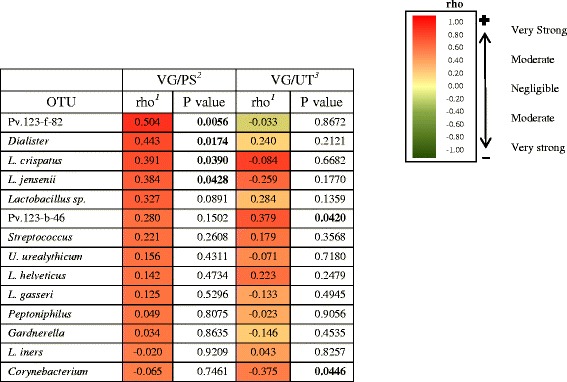
Correlation of specific OTUs* between vagina, penile skin and male urethra from normal-couples

^1^Spearman correlation coefficient. The *gradient bar* on the *right* shows the strength of the correlations

Positive correlations (*red*); negative correlations (*green*)

^2^Associations between vagina (VG) and penile skin (PS)

^3^Associations between vagina and male urethra (UT)

*Only those vaginal OTUs which had a prevalence of ≥30 % among normal-women were included in this analysis

## Discussion

While it is well-recognized that BV is associated with sexual activity, few studies provide evidence that BV-associated taxa are transferred during sexual intercourse. A notable outcome of our study, that is consistent with sexual exchange of BV-associated taxa, is that the penile skin microbiota of male partners of women with BV were more similar to the vaginal microbiota of their sexual partner compared to non-partner women who also had BV (Fig. 1). The data for BV-couples indicate that monogamous sexual intercourse, as defined in this study between a male partner and a female partner who has BV, has a homogenizing influence on the genital microbiota of the couple such that the genital microbiota of BV-couples become more similar over time. These results extend the data of Liu et al. [40] who have shown that BV-associated organisms are significantly increased in penile skin of BV-males. Taken together, the data from these two studies strongly support the hypothesis that BV-associated bacteria are sexually transmitted. Moreover, there appeared to be no influence of male circumcision on our findings, as the penile skin microbiota of both circumcised and uncircumcised BV-males was significantly more similar among sexual partners compared to non-partners. In contrast, no similar relationship was found in normal-couples, in either the penile skin or male urethral specimens. The reasons for the lack of significant similarity between normal-couples is unclear. However it seems likely that the high degree of similarity among the vaginal microbiota of normal-women, often predominated by one or two *Lactobacillus* species, such as *L. iners* or *L. crispatus*, decreases the discriminatory power of the analysis. Another possible explanation is that the predominant vaginal bacterial species in normal-women do not persist for an extended period of time in the environment of the penile skin or the male urethra.

Two longitudinal 16S rDNA-based studies have shown that circumcision significantly alters the composition of penile skin microbiota in paired specimens, collected pre- and post-circumcision [38, 42]. In contrast, we did not observe a significant difference in UniFrac distances of microbial communities in circumcised and uncircumcised male penile skin specimens, within either the normal male group or the BV male groups (Fig. 2). Significant differences in study design, study populations, sampling protocols, and analysis methods preclude direct comparisons of our results with those of Price et al. [38] and Liu et al. studies [42]. Price and Liu performed longitudinal studies comparing penile skin specimens before and (1 year) after circumcision, whereas ours was a cross-sectional study comparing groups of circumcised and uncircumcised men. In addition to study design, the characteristics of the female partners also differed between our study and those of Price and Liu [43]. Only 30 % of the regular sex partners of men in the Price and Liu studies had BV by the Nugent criteria [43]. Thus, over the 1-year observation period following circumcision, most of these men were not being continuously exposed to BV and many appear to have lost the bacteria which comprise BV-like microbiomes. In contrast, all of the BV men in our study were having concurrent intercourse with women who had BV. Thus, under this distinctly different condition, our data show that penile skin microbiomes do not differ between men based on circumcision status. Future prospective studies of male partners of women with BV, following successful treatment of the female partner, could be performed to test the hypothesis that circumcised men clear their penile skin of BV-like microbiomes more rapidly than uncircumcised men.

## Conclusions

In summary, we found that the penile skin of male partners of women with BV is colonized by many of the same bacterial taxa that are strongly associated with BV. More significantly, we found that the penile skin bacterial communities of male partners of women with BV were significantly more similar to the vaginal communities of their partner’s than to vaginal communities of other BV-women in the study, which strongly suggests that sexual transmission of BV-associated bacteria is a common occurrence. Urethral communities of male BV-partners were similarly related to the vaginal communities of their partners, though it is unclear how contamination of the male urethral swab specimen by penile skin organisms may have influenced this finding. The same similarity between genital microbiota of sexual partners was not seen in couples without BV. Taken together, these findings support the hypotheses that BV-associated bacteria are exchanged during sex and that male carriage of these organisms could contribute to relapses of clinical BV following treatment, and could be the cause of incident BV infection in previously unexposed women.

## Methods

### Ethics statement

All patients enrolled in this study gave written informed consent to their participation. The study protocol and consent form was approved by the LSU Health Sciences Center Institutional Review Board.

### Sample collection and clinical measurements

Couples enrolled in this study were recruited at the New Orleans sexually transmitted disease (STD) clinic. Couples in which the female had BV (BV-couples) were part of a larger cohort study designed to determine the role of genital tract microbiomes on BV treatment failure and relapse following treatment. Data presented here are from the enrollment visit only. Couples in which the female did not have BV (normal-couples) were recruited for the analyses presented here concerning the relationships between male and female partner microbiomes. All women were ≥18 years of age and attended the STD clinic with a partner with whom they had a monogamous sexual relationship for at least 1 month. The exclusion criteria included antibiotic use within the last 28 days, pregnancy, HIV infection, and any concurrent infection requiring either member of the couple to take antibiotics. Five vaginal swabs were collected from each woman: one for wet prep, KOH, and pH, one for Gram staining, one for *Trichomonas* culture, one for DNA extraction, and one for back-up. A urethral swab and two penile skin swabs were obtained from each male. The penile skin swabs were obtained by swabbing the glans, the coronal sulcus, and the shaft of the penis. The male swabs and the vaginal swabs intended for DNA extraction were placed in tubes containing the AssayAssure™ nucleic acid preservation media (Thermoscientific, Pittsburg, PA). Details on sample collection have been described previously [41]. The female specimens were characterized clinically for BV using Amsel criteria [31] and Nugent scores [44]. For the purpose of this study, the definition of BV required that the woman had a Nugent score of 7–10, and the definition of a normal woman was a Nugent score of 0–3. Based on these criteria, 96 couples were available for analysis including 65 in which the woman had BV and 31 in which the woman was normal. From these, 283 samples were available for analysis (96 vaginal, 94 urethral, and 93 penile skin). We use the terms BV-partners and normal partners to refer to males whose partners had BV and normal vaginal microbiota, respectively.

### Molecular methods

DNA extraction was performed using the QIAamp DNA mini kit (vaginal swabs) and QIAamp DNA micro kit (male specimens) (Qiagen Inc., Valencia, CA) according to the manufacturer’s instructions. An initial lysis step with 20 mg per milliliter lysozyme was included to improve DNA extraction from Gram-positive bacteria. The DNA obtained from the two penile skin swabs was combined to increase the yield. Bacterial tag-encoded FLX amplicon pyrosequencing analysis of the V4–V6 region of the 16S rRNA gene (530 F: GTGCCAGCMGCNGCGG and 1100R: GGGTTNCGNTCGTTG) was performed by a commercial facility (Research and Testing Laboratory, Lubbock, TX).

### Data analysis

Pyrosequencing post-processing was performed by the sequencing facility (http://www.researchandtesting.com/docs/Data_Analysis_Methodology.pdf). In brief, the data was quality-checked and denoised, including chimera checking. Sequences that were less than 200 bases in length, contained one or more ambiguous bases, had mean quality scores below Q25, or had a homopolymer region longer than six nucleotides were excluded from the analysis. The sequence files and mapping file for all samples have been deposited in Figshare (http://dx.doi.org/10.6084/m9.figshare.1321311). Taxonomic identification and data analysis was performed using an “in house” sequence analysis framework [45] and QIIME [46].

A total of 1,556,072 reads (vagina- 581,169 reads; penile skin- 471, 947 reads; urethra- 502,956 reads) with an average length of 455 ± 85 bases were obtained after pyrosequencing post-processing. The average number of reads per sample was 6053 (vaginal specimens), 5074 (penile skin specimens), and 5350 (urethral specimens). An initial taxonomic assignment of reads was performed using BLAST [47] at 97 % sequence similarity against a local database. The database contained 283 16S rRNA gene sequences, including sequences previously detected in PCR-based surveys of vaginal specimens [12, 14, 15, 48–51]. The list of sequences and NCBI reference numbers is available as Additional file 4. The reads that fell under the 97 % threshold, and thus were not matched to the local database sequences, were assigned to operational taxonomic units (OTUs) by the Ribosomal Database Project classifier [52] set at 80 % confidence threshold. A total of 70,793 reads (vagina- 16,523 [2.84 %] reads; penile skin- 13,610 [2.88 %] reads; urethra- 40,660 [8.08 %] reads) were below the 80 % confidence threshold for classification at the genus level and were excluded from further analysis. The taxonomic assignments are shown in Additional file 5: Table S4. Differences in the prevalence of individual taxa, between categories of specimens, were analyzed using Fisher’s exact test.

Analyses of bacterial diversity, as well as the distance measurements between bacterial communities, were performed using QIIME [46] after normalizing to a common sequencing depth. Diversity was measured using the average of ten random subsamples of each bacterial community and three diversity metrics, chao1, PD whole tree, and total OTUs. Distances between bacterial communities were measured using the unweighted UniFrac metric [53]. An unweighted UniFrac distance matrix containing the distance values between all the pairs of specimens in the study was generated using Python scripts within QIIME. To compare the bacterial community distances of sexual partners to the distances of non-partners, the following steps were used: (1) we obtained the UniFrac distance between a man and his sexual partner from the distance matrix. This was referred to as the partner distance; (2) we obtained all the distances between the man in step 1 and each of the other women in the study; (3) we calculated an average value for the distances obtained in step 2 and referred to this value as the non-partner distance; (4) we repeated steps 1, 2, and 3 for every man in the study; (5) we created two columns of paired data, each row containing a distance between a man and his sex-partner, and a distance between that same man and his “non-partner”; (6) we used a paired *t* test to test the statistical difference between the partner distances and the non-partner distances.

Spearman correlation coefficients were used to assess the correlation of specific organisms at different sites (vaginal, penile skin, and urethra). All organisms present in greater than 30 % of the BV-women and greater than 30 % of the normal-women were separately selected for this analysis. Correlation analyses for vaginal and penile skin and vaginal and urethral specimens were compared using *z* statistics following Fisher’s transformation. All analyses were conducted using SAS version 9.3.

#### Availability of supporting data

The sequence files and mapping file for all the samples included in this study have been deposited in Figshare (http://dx.doi.org/10.6084/m9.figshare.1321311).

## Additional files

Additional file 1: Table S1. Metadata from female patients. (XLS 72 kb) Additional file 2: Table S2. Metadata from male sexual partners. (XLS 60 kb) Additional file 3: Table S3. Average percent abundance of selected OTUs in vaginal specimens of BV- and normal-women, and in penile skin and urethral specimens from their sexual partners. (PDF 144 kb) Additional file 4: List of 16S rRNA gene sequences included in our local database. (PDF 644 kb) Additional file 5: Table S4. Taxonomic assignments of 16S rRNA sequences obtained from penile skin, urethral, and vaginal specimens. (XLSX 445 kb)
