# Supplementary material for: Bacterial communities in penile skin, male urethra, and vaginas of heterosexual couples with and without bacterial vaginosis
Source: Microbiome. 2016 Apr 19;4:16. doi: 10.1186/s40168-016-0161-6 (PMC4835890; doi:10.1186/s40168-016-0161-6)
Supplement: Additional file 3: Table S3. — Average percent abundance of selected OTUs in vaginal specimens of BV- and normal-women, and in penile skin and urethral specimens from their sexual partners. (PDF 144 kb) [file 40168_2016_161_MOESM3_ESM.pdf]

Table S3. Average Percent Abundance of Selected OTUs in Vaginal Specimens of BV- and Normal-women and in Penile Skin and Urethral Specimens from their sexual partners

	Vagina			***Penile Skin***			***Urethra***		
	**Mean % abundance**		**P value**	**Mean % abundance**		**P value**	**Mean % abundance**		**P value**
	**BV**	**Normal**		**BV**	**Normal**		**BV**	**Normal**	
Gardnerella vaginalis	15.94	2.80	**<0.0001*** **	1.79	0.40	**0.0005*** **	13.28	13.21	0.9776
Pv. 123-f2-42	11.01	0.08	**<0.0001*** **	0.64	0.08	**0.0443***	10.97	1.88	0.0706
Pv. 123-b-46	9.27	0.54	**<0.0001*** **	3.70	1.07	0.0867	2.42	0.55	**0.0040** **
Pv. 112Q-3	3.88	0.06	**<0.0001*** **	0.26	0.04	**0.0269***	3.77	1.04	0.8529
Leptotrichia amnionii	2.67	0.01	**<0.0001*** **	0.21	0.00	**0.0179***	1.32	0.12	0.1188
Peptoniphilus	2.17	0.46	**<0.0001*** **	11.62	12.83	0.2293	5.45	3.21	**0.0391***
Prevotella spp.	1.88	0.03	**<0.0001*** **	0.52	0.47	0.1790	1.07	0.53	**0.0049** **
Dialister	1.70	0.06	**<0.0001*** **	0.40	0.56	**0.0023** **	0.13	0.25	**0.0072** **
Eggerthella sp.	1.59	0.00	**<0.0001*** **	0.12	0.02	**0.0112***	0.21	0.18	**0.0473***
Porphyromonas	1.35	0.01	**<0.0001*** **	1.65	0.78	0.0784	0.46	0.06	0.3984
Atopobium vaginae	1.16	0.02	**<0.0001*** **	0.27	0.41	0.0710	0.18	0.20	0.0913
BVAB2	0.88	0.00	**<0.0001*** **	0.05	0.01	0.1494	0.21	0.31	**0.0236***
P. disiens	0.86	0.02	**<0.0001*** **	1.13	0.72	0.5892	0.42	0.15	0.2915
Sneathia	0.84	0.00	**<0.0001*** **	0.10	0.00	0.0706	1.29	0.25	**0.0070** **
Anaerococcus	0.83	0.02	**<0.0001*** **	4.17	3.64	**0.0132***	3.01	1.42	0.0590
Pv. 136-b-40	0.69	0.01	**<0.0001*** **	0.39	0.23	0.0644	0.12	0.00	**0.0290***
Peptostreptococcus	0.45	0.00	**<0.0001*** **	0.13	0.28	**0.0193***	0.09	0.15	0.1707
Aerococcus	0.27	0.05	**<0.0001*** **	0.06	0.04	0.7714	0.56	0.72	0.8143
Mycoplasma hominis	0.16	0.00	**<0.0001*** **	0.02	0.00	0.1214	0.00	0.00	0.0619
Pv. 123f3-83	0.14	0.00	**<0.0001*** **	0.04	0.02	0.1533	0.01	0.00	0.2264
Megasphaera 1	0.09	0.00	**<0.0001*** **	0.01	0.02	**0.0004*** **	0.02	0.00	0.0952
Megasphaera 2	0.06	0.00	**<0.0001*** **	0.00	0.00	**0.0162***	0.00	0.00	0.4532
Parvimonas	0.02	0.00	**0.0001*** **	0.01	0.01	0.9185	0.01	0.01	0.1066
Pv. 123f2-17	0.42	0.03	**0.0003*** **	0.48	0.29	0.2844	0.60	0.00	0.1234
Pv. 123b-95	0.04	0.00	**0.0004*** **	0.06	0.00	0.7269	0.03	0.00	0.8251
Gemella	0.09	0.01	**0.0008*** **	0.08	0.02	0.7603	2.15	0.44	0.6696
Mobiluncus curtisii	3.55	0.23	**0.0011** **	0.12	0.36	0.2071	4.33	6.10	0.171
Actinomyces	0.02	0.00	**0.0012** **	0.22	0.54	0.1758	0.06	0.06	0.3086
Barnesiella	0.25	0.00	**0.0016** **	0.06	0.02	0.3313	0.00	0.00	0.3422
Pv. 123b-4	0.02	0.00	**0.0017** **	0.00	0.00	0.9557	0.01	0.00	0.7736
Pv. 123-f-110	5.58	0.09	**0.0020** **	1.88	0.01	0.0643	0.43	1.02	0.4396
Pv. 123b-5	2.27	0.01	**0.0021** **	0.04	0.00	**0.0406***	0.02	0.37	0.5554
Bulleida	0.02	0.00	**0.0023** **	0.00	0.00	0.306	0.00	0.00	0.9368
P. bivia	1.85	0.06	**0.0028** **	1.67	1.61	0.8417	1.32	1.40	0.2566
U. urealyticum	0.02	0.09	**0.0044** **	0.01	0.02	0.4911	0.28	1.17	0.0845
BVAB3	0.11	0.00	**0.0075** **	0.05	0.00	0.1632	0.00	0.03	0.4532
BVAB1	2.31	0.02	**0.0091** **	0.15	0.00	0.1264	0.01	0.05	0.7458
Pv. 113s1-20	0.56	0.02	**0.0095** **	0.32	0.06	**0.0143***	0.19	0.02	0.2893
Pv. 123f-82	3.69	0.47	**0.0103***	3.73	5.05	0.5186	3.57	1.63	0.1474
Pv. 127-Q 23	0.04	0.00	**0.0145***	0.02	0.04	0.8808	0.02	0.00	0.2396
Mobiluncus mulieris	5.35	0.20	**0.0287***	0.37	0.00	0.3518	0.02	0.01	na
Moryella	1.71	0.00	**0.0372***	0.05	0.00	**0.0424***	0.03	0.12	0.9789
L. gasseri	0.00	0.09	**<0.0001*** **	0.00	0.00	0.8370	0.00	0.00	0.9789
L. helveticus	0.00	0.69	**<0.0001*** **	0.00	0.00	0.5535	0.00	0.00	0.1999
L. iners	5.88	64.03	**<0.0001*** **	1.95	3.04	0.0575	9.83	17.43	0.1873
L. jensenii	0.02	2.19	**<0.0001*** **	0.01	0.02	0.0513	0.02	0.08	0.4273
Lactobacillus spp	0.05	0.35	**<0.0001*** **	0.01	0.04	0.6216	0.09	0.07	0.7947
L. crispatus	0.01	22.14	**0.0002*** **	0.04	0.71	**0.0013*** **	0.01	0.05	0.7208
L. vaginalis	0.01	0.10	**0.0008*** **	0.00	0.12	0.9115	0.00	0.00	0.5953
L. reuteri	0.01	0.10	**0.0003*** **	0.00	0.02	0.5535	0.00	0.00	0.5953
