# Supplementary material for: Bacterial communities in penile skin, male urethra, and vaginas of heterosexual couples with and without bacterial vaginosis
Source: Microbiome. 2016 Apr 19;4:16. doi: 10.1186/s40168-016-0161-6 (PMC4835890; doi:10.1186/s40168-016-0161-6)
Supplement: Additional file 4: — List of 16S rRNA gene sequences included in our local database. (PDF 644 kb) [file 40168_2016_161_MOESM4_ESM.pdf]

>53TVGPR82|gb|CE02DLCKS|Mnola|Unknown Mycoplasmataceae
AGAGTTTGATCCTGGCTCAGGATTAACGCTAGCGGTATGCCTAATACATGCAAGTCGAGCGGAGGTAGCAATA
CCTTAGCGGCGAACGGGTGAGTAATACGTATCTAATCTGCCCTTTAGAAAGGAACAACAGATCGAAAGATTTG
CTAATACCTTATATGATATACATTCGCATGAAAGTATATTTAAAGTTGCGTTTGCAACGCTTTAGGATGAGGG
TGCGGTTTATCAGATAGTTGGTGAGGTAATGGCTCACCAAGTCAATGACGAATAGCTATGCTGAGAGGTAGAA
TAGCCACAAGGGGACTGAGACACGGCCCCTACTCCTACGGGAGGCAGCAGTAGGGAATTTTTCACAATGGGCG
AAAGCCTGATGGAGCAATACCGCGTGAATGATGAAGGTCTTCGGATTGTAAAATTCTTTTATTAGGGACGAAC
GGCACTAAGAGGAAATGCTTAGTGATTGACTGTACCTATTGAATAAGTAACGGCAAACTATGTGCCAGCAGCC
GCGGTAATACATAGGTTACAAGCGTTATCCGGATTTACTGGGCGTAAAGCGAGCGCAGGCTGATTAACAAGTC
TAGTGTGAAATGCAGTTGCTTAACAACTGTTTGCATTGGAAACTGTTAGTCTGGAGTGTGATAGGGAGTTCTG
GAACTCCATATGGAGCGGTGGAATGCGTAGATATATGGAAGAACACCTGTGGCGAAAGCGAGAACTTAGGTCA
CAACTGACGCTTAGGCTCGAAAGTGTGGGGAGCAAATAGGATTAGATACCCTAGTAGTCCACACCGTAAACGA
TGGTTATTAGTTGTTGGTACGAAGTACTAGTAACGAAGCTAACGCATTAAATAACCCGCCTGGGTAGTACATT
CGCAAGAATGAAACTCAAACGGAATTGACGGGGACCCGCACAAGTGGTGGAGCATGTTGTTTAATTCGACAGT
ACACGAAGAACCTTACCAGGGTTTGACATCCTCGGCAAAGCTATAGAAATATAGTGGAGGTTAACCGAGAGAC
AGGTGGTGCATGGTTGTCGTCAGCTCGTGTCGTGAGATGTTGGGTTAAGTCCCGCAACGAGCGCAACCCCTAT
TGTTAGTTACTTTATCTAACGAGACTGCTAACGCAAGTTAGAGGAAGGTGGGGATGACGTCAAATCATCATGC
CCTTTATATCCTGGGCCACAAACGTGCTACAATGGTTGGTACAAACTGTTGCCAAACTGTAAAGTGGAGCTAA
TCAGAAAAAACCAATCTCAGTTCGGATTGAGGGCTGAAATTCGTCCTCATGAAGTTGGAATCACTAGTAATCG
CGAATCAGACATGTCGCGGTGAATGCGTTCTCGGGTCTTGTACACACCGCCCGTCAAACTATGAGAGTCGTTA
ATACCTAAAACCGTCCTGCTAACCGTAAGGGAGCGGATGTCTAGGGTAGGAATGATGATTGGAGTTAAGTCGT
AACAAGGTAACC
>119352238|gb|EF120361.1|L.iners|Uncultured Lactobacillus sp. clone MZH0805-46 16S ribosomal RNA gene, partial sequence
GACGAACGCTGGCGGCGTGCCTAATACATGCAAGTCGAGCGAGTCTGCCTTGAAGATCGGAGTGCTTGCA
CTCTGTGAAACAAGATACAGGCTAGCGGCGGACGGGTGAGTAACACGTGGGTAACCTGCCCAAGAGATCG
GGATAACACCTGGAAACAGATGCTAATACCGGATAACAACAGATGATGCCTATCAACTGTTTAAAAGATG
GTTCTGCTATCACTCTTGGATGGACCTGCGGTGCATTAGCTAGTTGGTAGGGTAACGGCCTACCAAGGCG
ATGATGCATAGCCGAGTTGAGAGACTGATCGGCCACATTGGGACTGAGACACGGCCCAAACTCCTACGGG
AGGCAGCAGTAGGGAATCTTCCACAATGGACGCAAGTCTGATGGAGCAACGCCGCGTGAGTGAGGAAGGG
TTTCGGCTCGTAAAGCTCTGTTGTTGGTGAAGAAGGACAGGGGTAGTAACTGACCTTTGTTTGACGGTAA
TCAATTAGAAAGTCACGGCTAACTACGTGCCAGTAGCCGCGGTAATACGTAGGTGGCAAGCGTTGTCCGG
ATTTATTGGGCGTAAAGCGAGTGCAGGCGGCTCGATAAGTCTGATGTGAAAGCCTTCGGCTCAACCGGAG
AATTGCATCAGAAACTGTCGAGCTTGAGTACAGAAGAGGAGAGTGGAACTCCATGTGTAGCGGTGAAATG
CGTAGATATATGGAAGAACACCGGTGGCGAAGGCGGCTCTCTGGTCTGTTACTGACGCTGAGGCTCGAAA
GCATGGGTAGCGAACAGGATTAGATACCCTGGTAGTCCATGCCGTAAACGATGAGTGCTAAGTGTTGGGA
GGTTTCCGCCTCTCAGTGCTGCAGCTAACGCATTAAGCACTCCGCCTGGGGAGTACGACCGCAAGGTTGA
AACTCAAAGGAATTGACGGGGGCCCGCACAAGCGGTGGAGCATGTGGTTTAATTCGAAGCAACGCGAAGA
ACCTTACCAGGTCTTGACATCCATAGCCAGTCTAAGAGATTAGATGTTCCCTTCGGGGACTATGAGACAG
GTGGTGCATGGCTGTCGTCAGCTCGTGTCGTGAGATGTTGGGTTAAGTCCCGCGACGAGCGCAACCCTTG
TCATTAGTTGCCAGCATTAAGTTGGGCACTCTAATGAGACTGCCGGTGACAAACTGGAGGAAGGTGGGGA
TGACGTCAAGTCATCATGCCCCTTATGACCTGGGCTGCACACGTGCTACAATGGACGGTACAACGAGAAG
CGACCCTGTGAAGGCAAGCGGATCTCTGAAAGCCGTTCTCAGTTCGGATTGCAGGCTGCAACTCGCCTGC
ATGAAGCTGGAATCGCTAGTAATCGCAAATCAGCACGTTGCGGTGAATACGTTCCCGGGCCTTGTACACA
CCGCCCGTCACACCATGAGAGTCTGTAACGCCCGAAGCCGGCGGGATAACCGAAAGGAGTCAGCCGTCTA
AGGCGGGACAGATGATTAGGGTG
>42521630|gb|AY526083.1|L.iners|Lactobacillus iners 16S ribosomal RNA gene, partial sequence
GACGAACGCTGGCGGCGTGCCTAATACATGCAAGTCGAGCGAGTCTGCCTTGAAGATCGGAGTGCTTGCA
CTCTGTGAAACAAGATACAGGCTAGCGGCGGACGGGTGAGTAACACGTGGGTAACCTGCCCAAGAGATCG
GGATAACACCTGGAAACAGATGCTAATACCGGATAACAACAGATGATGCCTATCAACTGTTTAAAAGATG
GTTCTGCTATCACTCTTGGATGGACCTGCGGTGCATTAGCTAGTTGGTAGGGTAACGGCCTACCAAGGCG
ATGATGCATAGCCGAGTTGAGAGACTGATCGGCCACATTGGGACTGAGACACGGCCCAAACTCCTACGGG
AGGCAGCAGTAGGGAATCTTCCACAATGGACGCAAGTCTGATGGAGCAACGCCGCGTGAGTGAAGAAGGG

TTTCGGCTCGTAAAGCTCTGTTGTTGGTGAAGAAGGACAGGGGTAGTAACTGACCTTTGTTTGACGGTAA
TCAATTAGAAAGTCACGGCTAACTACGTGCCAGCAGCCGCGGTAATACGTAGGTGGCAAGCGTTGTCCGG
ATTTATTGGGCGTAAAGCGAGTGCAGGCGGCTCGATAAGTCTGATGTGAAAGCCTTCGGCTCAACCGGAG
AATTGCATCAGAAACTGTCGAGCTTGAGTACAGAAGAGGAGAGTGGAACTCCATGTGTAGCGGTGAAATG
CGTAGATATATGGAAGAACACCGGTGGCGAAGGCGGCTCTCTGGTCTGTTACTGACGCTGAGGCTCGAAA
GCATGGGTAGCGAACAGGATTAGATACCCTGGTAGTCCATGCCGTAAACGATGAGTGCTAAGTGTTGGGA
GGTTTCCGCCTCTCAGTGCTGCAGCTAACGCATTAAGCACTCCGCCTGGGGAGTACGACCGCAAGGTTGA
AACTCAAAGGAATTGACGGGGGCCCGCACAAGCGGTGGAGCATGTGGTTTAATTCGAAGCAACGCGAAGA
ACCTTACCAGGTCTTGACATCCATAGCCAGTCTAAGAGATTAGATGTTCCCTTCGGGGACTATGAGACAG
GTGGTGCATGGCTGTCGTCAGCTCGTGTCGTGAGATGTTGGGTTAAGTCCCGCAACGAGCGCAACCCTTG
TCATTAGTTGCCAGCATTAAGTTGGGCACTCTAATGAGACTGCCGGTGACAAACCGGAGGAAGGTGGGGA
TGACGTCAAGTCATCATGCCCCTTATGACCTGGGCTACACACGTGCTACAATGGACGGTACAACGAGAAG
CGACCCTGTGAAGGCAAGCGGATCTCTGAAAGCCGTTCTCAGTTCGGATTGCAGGCTGCAACTCGCCTGC
ATGAAGCTGGAATCGCTAGTAATCGCAAATCAGCACGTTGCGGTGAATACGTTCCCGGGCCTTGTACACA
CCGCCCGTCACACCATGAGAGTCTGTAACGCCCGAAGCCGGCGGGATAACCGAAAGGAGTCAGCCGTCTA
AGGCGGGAC
>66878605|gb|AY958899.1|L.iners|Uncultured bacterium clone rRNA126 16S ribosomal RNA gene, partial sequence
GCCCTTAGAGTTTGATCCTGGCTCAGGACGAACGCTGGCGGCGTGCCTAATACATGCAAGTCGAGCGAGT
CTGCCTTGAAGATCGGAGTGCTTGCACTCTGTGAAACAAGATACAGGCTAGCGGCGGACGGGTGAGTAAC
ACGTGGGTAACCTGCCCAAGAGATCGGGATAACACCTGGAAACAGATGCTAATACCGGATAACAACAGAT
GATGCCTATCAACTGTTTAAAAGATGGTTCTGCTATCACTCTTGGATGGACCTGCGGTGCATTAGCTAGT
TGGTAGGGTAACGGCCTACCAAGGCGATGATGCATAGCCGAGTTGAGAGACTGATCGGCCACATTGGGAC
TGAGACACGGCCCAAACTCCTACGGGAGGCAGCAGTAGGGAATCTTCCACAATGGACGCAAGTCTGATGG
AGCAACGCCGCGTGAGTGAAGAAGGGTTTCGGCTCGTAAAGCTCTGTTGTTGGTGAAGAAGGACAGGGGT
AGTAACTGACCTTTGTTTGACGGTAATCAATTAGAAAGTCACGGCTAACTACGTGCCAGCAGCCGCGGTA
ATACGTAGGTGGCAAGCGTTGTCCGGATTTATTGGGCGTAAAGCGAGTGCAGGCGGCTCGATAAGTCTGA
TGTGAAAGCCTTCGGCTCAACCGGAGAATTGCATCAGAAACTGTCGAGCTTGAGTACAGAAGAGGAGAGT
GGAACTCCATGTGTAGCGGTGAAATGCGTAGATATATGGAAGAACACCGGTGGCGAAGGCGGCTCTCTGG
TCTGTTACTGACGCTGAGGCTCGAAAGCATGGGTAGCGAACAGGATTAGATACCCTGGTAGTCCATGCCG
TAAACGATGAGTGCTAAGTGTTGGGAGGTTTCCGCCTCTCAGTGCTGCAGCTAACGCATTAAGCACTCCG
CCTGGGGAGTACGACCGCAAGGTTGAAACTCAAAGGAATTGACGGGGGCCCGCACAAGCGGTGGAGCATG
TGGTTTAATTCGAAGCAACGCGAAGAACCTTACCAGGTCTTGACATCCATAGCCAGTCTAAGAGATTAGA
TGTTCCCTTCGGGGACTATGAGACAGGTGGTGCATGGCTGTCGTCAGCTCGTGTCGTGAGATGTTGGGTT
AAGTCCCGCAACGAGCGCAACCCTTGTCATTAGTTGCCAGCATTAAGTTGGGCACTCTAATGAGACTGCC
GGTGACAAACCGGAGGAAGGTGGGGATGACGTCAAGTCATCATGCCCCTTATGACCTGGGCTACACACGT
GCTACAATGGACGGTACAACGAGAAGCGACCCTGTGAAGGCAAGCGGATCTCTGAAAGCCGTTCTCAGTT
CGGATTGCAGGCTGCAACTCGCCTGCATGAAGCTGGAATCGCTAGTAATCGCAAATCAGCACGTTGCGGT
GAATACGTTCCCGGGCCTTGTACACACCGCCCGTCACACCATGAGAGTCTGTAACGCCCGAAGCCGGCGG
GATAACCGAAAGGAGTCAGCCGTCTAAGGCGGGACAGATGATTAGGGTGAAGTCGTAACAAGGTAACCGT
AAAGGGC
>310975118|ref|NR_036982.1|L.iners|Lactobacillus iners strain DSM 13335 16S ribosomal RNA, partial sequence
TGGCTCAGGACGAACGCTGGCGGCGTGCCTAATACATGCAAGTCGAGCGAGTCTGCCTTGAAGATCGGAG
TGCTTGCACTCTGTGAAACAAGATACAGGCTAGCGGCGGACGGGTGAGTAACACGTGGGTAACCTGCCCA
AGAGATCGGGATAACACCTGGAAACAGATGCTAATACCGGATAACAACAGATGATGCCTATCAACTGTTT
AAAAGATGGTTCTGCTATCACTCTTGGATGGACCTGCGGTGCATTAGCTAGTTGGTAGGGTAACGGCCTA
CCAACGCGATGATGCATAGCCGAGTTGAGAGACTGATCGGCCACATTGGGACTGAGACACGGCCCAAACT
CCTACGGGAGGCAGCAGTAGGGAATCTTCCACAATGGACGCAAGTCTGATGGAGCAACGCCGCGTGAGTG
AAGAAGGGTTTCGGCTCGTAAAGCTCTGTTGTTGGTGAAGAAGGACAGGGGTAGTAACTGACCTTTGTTT
GACGGTAATCAATTAGAAAGTCACGGCTAACTACGTGCCAGCAGCCGCGGTAATACGTAGGTGGCAAGCG
TTGTCCGGATTTATTGGGCGTAAAGCGAGTGCAGGCGGCTCGATAAGTCTGATGTGAAAGCCTTCGGCTC
AACCGGAGAATTGCATCAGAAACTGTCGAGCTTGAGTACAGAAGAGGAGAGTGGAACTCCATGTGTAGCG
GTGAAATGCGTAGATATATGGAAGAACACCGGTGGCGAAGGCGGCTCTCTGGTCTGTTACTGACGCTGAG

GCTCGAAAGCATGGGTAGCGAACAGGATTAGATACCCTGGTAGTCCATGCCGTAAACGATGAGTGCTAAG
TGTTGGGAGGTTTCCGCCTCTCAGTGCTGCAGCTAACGCATTAAGCACTCCGCCTGGGGAGTACGACCGC
AAGGTTGAAACTCAAAGGAATTGACGGGGGCCCGCACAAGCGGTGGAGCATGTGGTTTAATTCGAAGCAA
CGCGAAGAACCTTACCAGGTCTTGACATCCATAGCCAGTCTAAGAGATTAGATGTTCCCTTCGGGGACTA
TGAGACAGGTGGTGCATGGCTGTCGTCAGCTCGTGTCGTGAGATGTTGGGTTAAGTCCCGCAACGAGCGC
AACCCTTGTCATTAGTTGCCAGCATTAAGTTGGGCACTCTAATGAGACTGCCGGTGACAAACCGGAGGAA
GGTGGGGATGACGTCAAGTCATCATGCCCCTTATGACCTGGGCTACACACGTGCTACAATGGACGGTACA
ACGAGAAGCGACCCTGTGAAGGCAAGCGGATCTCTGAAAGCCGTTCTCAGTTCGGATTGCAGGCTGCAAC
TCGCCTGCATGAAGCTGGAATCGCTAGTAATCGCAAATCAGCACGTTGCGGTGAATACGTTCCCGGGCCT
TGTACACACCGCCCGTCACACCATGAGAGTCTGTAACGCCCGAAGCCGGCGGGATAACCGAAAGGAGTCA
GCCGTCTAAGGCGGGACAGATGATTAGGGTGAAGTCGTAACAAGGTAGCCGTAGGAGAACCTGCGGCTG
>66878566|gb|AY958860.1|L.iners|Uncultured bacterium clone rRNA087 16S ribosomal RNA gene, partial sequence
TTCGCCCTTAGAGTTTGATCCTGGCTCAGGACGAACGCTGGCGGCGTGCCTAATACATGCAAGTCGAGCG
AGTCTGCCTTGAAGATCGGAGTGCTTGCACTCTGTGAAACAAGATACAGGCTAGCGGCGGACGGGTGAGT
AACACGTGGGTAACCTGTCCAAGAGATCGGGATAACACCTGGAAACAGATGCTAATACCGGATAACAACA
GGTGATGCCTATCAACTGTTTAAAAGATGGTTCTGCTATCACTCTTGGATGGACCTGCGGTGCATTAGCT
AGTTGGTAGGGTAACGGCCTACCAAGGCGATGATGCATAGCCGAGTTGAGAGACTGATCGGCCACATTGG
GACTGAGACACGGCCCAAACTCCTACGGGAGGCAGCAGTAGGGAATCTTCCACAATGGACGCAAGTCTGA
TGGAGCAACGCCGCGTGAGTGAAGAAGGGTTTCGGCTCGTAAAGCTCTGTTGTTGGTGAAGAAGGACAGG
GGTAGTAACTGACCTTTGTTTGACGGTAATCAATTAGAAAGTCACGGCTAACTACGTGCCAGCAGCCGCG
GTAATACGTAGGTGGCAAGCGTTGTCCGGATTTATTGGGCGTAAAGCGAGTGCAGGCGGCTCGATAAGTC
TGATGTGAAAGCCTTCGGCTCAACCGGAGAATTGCATCAGAAACTGTCGAGCTTGAGTACAGAAGAGGAG
AGTGGAACTCCATGTGTAGCGGTGAAATGCGTAGATATATGGAAGAACACCGGTGGCGAAGGCGGCTCTC
TGGTCTGTTACTGACGCTGAGGCTCGAAAGCATGGGCAGCGAACAGGATTAGATACCCTGGTAGTCCATG
CCGTAAACGATGAGTGCTAAGTGTTGGGAGGTTTCCGCCTCTCAGTGCTGCAGCTAACGCATTAAGCACT
CCGCCTGGGGAGTACGACCGCAAGGTTGAAACTCAAAGGAATTGACGGGGGCCCGCACAAGCGGTGGAGC
ATGTGGTTTAATTCGAAGCAACGCGAAGAACCTTACCAGGTCTTGACATCCATAGCCAGTCTAAGAGATT
AGATGTTCCCTTCGGGGACTATGAGACAGGTGGTGCATGGCTGTCGTCAGCTCGTGTCGTGAGATGTTGG
GTTAAGTCCCGCAACGAGCGCAACCCTTGTCATTAGTTGCCAGCATTAAGTTGGGCACTCTAATGAGACT
GCCGGTGACAAACCGGAGGAAGGTGGGGATGACGTCAAGTCATCATGCCCCTTATGACCTGGGCTACACA
CGTGCTACAATGGACGGTACAACGAGAAGCGACCCTGTGAAGGCAAGCGGATCTCTGAAAGCCGTTCTCA
GTTCGGATTGCAGGCTGCAACTCGCCTGCATGAAGCTGGAATCGCTAGTAATCGCAAATCAGCACGTTGC
GGTGAATACGTTCCCGGGCCTTGTACACACCGCCCGTCACACCATGAGAGTCTGTAACGCCCGAAGCCGG
CGGGATAACCGAAAGGAGTCAGCCGTCTAAGGCGGGACAGATGATTAGGGTGAAGTCGTAACAAGGTAGC
CGTAAAGGGC
>119352235|gb|EF120358.1|Megasphaera1|Uncultured Megasphaera sp. clone MZH9600 16S ribosomal RNA gene, partial sequence
GACGAACGCTGGCGGCGTGCTTAACACATGCAAGTCGAACGAGAGGACATGGGAAGCTTGCTTCCTATGA
AATCGAGTGGCAAACGGGTGAGTAACGCGTAAACAACCTGCCCTTCGGATGGGGATAACAGCCGGAAACG
GCTGCTAATACCGAATACGATCTTTTCGTCGCATGACGGAAAGAAGAAAGGATGGCCTCTATACAAAGCT
ATCGCCGAAGGAGGGGTTTGCGTCTGATTAGCTGGTTGGAGGGGTAACGGCCCAACAAGGCGATGATCAG
TAGCCGGTCTGAGAGGATGAACGGCCACATTGGGACTGAGACACGGCCCAGACTCCTACGGGAGGCAGCA
GTGGGGAATCTTCCGCAATGGACGAAAGTCTGACGGAGCAACGCCGCGTGAGTGAAGAAGGTCTTCGGAT
TGTAAAGCTCTGTTATACGGGACGAAAAAGACGGATGCCAACAGTATCCGTCCGTGACGGTACCGTAAGA
GAAAGCCACGGCTAACTACGTGCCAGCAGCCGCGGTAATACGTAGGTGGCAAGCGTTGTCCGGAATTATT
GGGCGTAAAGGGCGCGCAGGCGGTTCGGTAAGTCTGTCTTAAAAGTGCGGGGCTTAACCCCGTGAGGGGA
CGGAAACTGTCGAACTTGAGTGTCGGAGAGGAAAGCGGAATTCCTAGTGTAGCGGTGAAATGCGTAGATA
TTAGGAGGAACACCGGTGGCGAAAGCGGCTTTCTGGACGACAACTGACGCTGAGGCGCGAAAGCGTGGGG
AGCAAACAGGATTAGATACCCTGGTAGTCCACGCCGTAAACGATGGATACTAGGTGTAGGAGGCATCGAC
CCCTTCTGTGCCGTAGTTAACGCTATAAGTATCCCGCCTGGGGAGTACGGCCGCAAGGTTGAAACTCAAA
GGAATTGACGGGGGCCCGCACAAGCGGTGGAGTATGTGGTTTAATTCGACGCAACGCGAAGAACCTTACC
AAGCCTTGACATTGATCGCAATTTTCAGAGATGAGAAGTTCCTCTTCGGAGGACGAGAAAACAGGTGGTG
CACGGCTGTCGTCAGCTCGTGTCGTGAGATGTTGGGTTAAGTCCCGCAACGAGCGCAACCCCTATCTTCT

GTTACCAGCACGTAAGGGTGGGGACTCAGGAGAGACTGCCGCAGACAATGCGGAGGAAGGCGGGGATGAC
GTCAAGTCATCATGCCCCTTATGGCTTGGGCTACACACGTACTACAATGGCTCTAAATAGAGGGAAGCGA
AGGAGCGATCCGGAGCAAAACCCAAAAACAGAGTCCCAGTTCGGATTGCAGGCTGCAACTCGCCTGCATG
AAGCAGGAATCGCTAGTAATCGCAGGTCAGCATACTGCGGTGAATACGTTCCCGGGCCTTGTACACACCG
CCCGTCACACCACGAAAGTCATTCACACCCGAAGCCGGTGAGGTAACCGCAAGGAGCCAGCCGTCGAAGG
TGGGGGCGATGATTGGGGTG
>66878799|gb|AY959093.1|Megasphaera1|Uncultured bacterium clone rRNA320
16S ribosomal RNA gene, partial sequence
CCTTAGAGTTTGATCCTGGCTCAGGACGAACGCTGGCGGCGTGCTTAACACATGCAAGTCGAACGAGAGG
ACATGGGAAGCTTGCTTCCTATGAAATCGAGTGGCAAACGGGTGAGTAACGCGTAAACAACCTGCCCTTC
GGATGGGGATAACAGCCGGAAACGGCTGCTAATACCGAATACGATCTTTTCGTCGCATGACGGAAAGAAG
AAAGGATGGCCTCTATACAAAGCTATCGCCGAAGGAGGGGTTTGCGTCTGATTAGCTGGTTGGAGGGGTA
ACGGCCCAACAAGGCGATGATCAGTAGCCGGTCTGAGAGGATGAACGGCCACATTGGGACTGAGACACGG
CCCAGACTCCTACGGGAGGCAGCAGTGGGGAATCTTCCGCAATGGACGAAAGTCTGACGGAGCAACGCCG
CGTGAGTGAAGAAGGTCTTCGGATTGTAAAGCTCTGTCATACGGGACGAAAAAGACGGATGCCAACAGTA
TCCGTCCGTGACGGTACCGTAAGAGAAAGCCACGGCTAACTACGTGCCTGCAGCCGCGGTAATACGTAGG
TGGCGAGCGTTGTCCGGAATTATTGGGCGTAAAGGGCGCGCAGGCGGTTCGGTAAGTCTGTCTTAAAAGT
GCGGGGCTTAACCCCGTGAGGGGACGGAAACTGTCGAACTTGAGTGTCGGAGAGGAAAGCGGAATTCCTA
GTGTAGCGGTGAAATGCGTAGATATTAGGAGGAACACCGGTGGCGAAAGCGGCTTTCTGGACGACAACTG
ACGCTGAGGCGCGAAAGCGTGGGGAGCAAACAGGATTAGATACCCTGGTAGTCCACGCCGTAAACGATGG
ATACTAGGTGTAGGAGGTATCGACCCCTTCTGTGCCGTAGTTAACGCTATAAGTATCCCGCCTGGGGAGT
ACGGCCGCAAGGTTGAAACTCAAAGGAATTGACGGGGGCCCGCACAAGCGGTGGAGTATGTGGTTTAATT
CGACGCAACGCGAAGAACCTTACCAAGCCTTGACATTGATCGCAATTTTCAGAGATGAGAAGTTCCTCTT
CGGAGGACGAGAAAACAGGTGGTGCACGGCTGTCGTCAGCTCGTGTCGTGAGATGTTGGGTTAAGTCCCG
CAACGAGCGCAACCCCTATCTTCTGTTACCAGCACGTAAGGGTGGGGACTCAGGAGAGACTGCCGCAGAC
AATGCGGAGGAAGGCGGGGATGACGTCAAGTCATCATGCCCCTTATGGCTTGGGCTACACACGTACTACA
ATGGCTCTAAATAGAGGGAAGCGAAGGAGCGATCCGGAGCAAAACCCAAAAACAGAGTCCCAGTTCGGAT
TGCAGGCTGCAACTCGCCTGCATGAAGCAGGAATCGCTAGTAATCGCAGGTCAGCATACTGCGGTGAATA
CGTTCCTGGGCCTTGTACACACCGCCCGTCACACCACGAAAGTCATTCACACCCGAAGCCGGTGAGGTAA
CCGCAAGGAGCCAGCCGTCGAAGGTGGGGGCGATGATTGGGGTGAAGTCGTAACAAGGTAGCCGTAAAGG
GCG
>66878510|gb|AY958804.1|Megasphaera1|Uncultured bacterium clone rRNA031
16S ribosomal RNA gene, partial sequence
TCGCCCTTAGAGTTTGATCCTGGCTCAGGACGAACGCTGGCGGCGTGCTTAACACATGCAAGTCGAACGA
GAGGACATGGGAAGCTTGCTTCCTATGAAATCGAGTGGCAAACGGGTGAGTAACGCGTAAACAACCTGCC
CTTCGGATGGGGATAACAGCCGGAAACGGCTGCTAATACCGAATACGATCTTTTCGTCGCATGACGAAAA
GAAGAAAGGATGGCCTCTATACAAAGCTATCGCCGAAGGAGGGGTTTGCGTCTGATTAGCTGGTTGGAGG
GGTAACGGCCCAACAAGGCGATGATCAGTAGCCGGTCTGAGAGGATGAACGGCCACATTGGGACTGAGAC
ACGGCCCAGACTCCTACGGGAGGCAGCAGTGGGGAATCTTCCGCAATGGACGAAAGTCTGACGGAGCAAC
GCCGCGTGAGTGAAGAAGGTCTTCGGATTGTAAAGCTCTGTTATACGGGACGAAAAAGACGGATGCCAAC
AGTATCCGTCCGTGACGGTACCGTAAGAGAAAGCCACGGCTAACTACGTGCCAGCAGCCGCGGTAATACG
TAGGTGGCAAGCGTTGTCCGGAATTATTGGGCGTAAAGGGCGCGCAGGCGGTTCGGTAAGTCTGTCTTAA
AAGTGCGGGGCTTAACCCCGTGAGGGGACGGAAACTGTCGAACTTGAGTGTCGGAGAGGAAAGCGGAATT
CCTAGTGTAGCGGTGAAATGCGTAGATATTAGGAGGAACACCGGTGGCGAAAGCGGCTTTCTGGACGACA
ACTGACGCTGAGGCGCGAAAGCGTGGGGAGCAAACAGGATTAGATACCCTGGTAGTCCACGCCGTAAACG
ATGGATACTAGGTGTAGGAGGTATCGACCCCTTCTGTGCCGTAGTTAACGCTATAAGTATCCCGCCTGGG
GAGTACGGCCGCAAGGTTGAAACTCAAAGGAATTGACGGGGGCCCGCACAAGCGGTGGAGTATGTGGTTT
AATTCGACGCAACGCGAAGAACCTTACCAAGCCTTGACATTGATCGCAATTTTCAGAGATGAGAAGTTCC
TCTTCGGAGGACGAGAAAACAGGTGGTGCACGGCTGTCGTCAGCTCGTGTCGTGAGATGTTGGGTTAAGT
CCCGCAACGAGCGCAACCCCTATCTTCTGTTACCAGCACGTAAGGGTGGGGACTCAGGAGAGACTGCCGC
AGACAATGCGGAGGAAGGCGGGGATGACGTCAAGTCATCATGCCCCTTATGACCTGGGCTACACACGTGC
TACAATGGACGGTACAACGAGAAGCGACCCTGTGAAGGCAAGCGGATCTCTGAAAGCCGTTCCCAGTTCG
GATTGCAGGCTGCAACTCGCCTGCATGAAGCTGGAATCGCTAGTAATCGCAAATCAGCACGTTGCGGTGA
ATACGTTCCCGGGCCTTGTACACACCGCCCGTCACACCATGAGAGTCTGTAACGCCCGAAGCCGGCGGGA

TAACCGAAAGGAGTCAGCCGTCTAAGGCGGGACAGATGATTAGGGTGAA
>119352236|gb|EF120359.1|Megasphaera2|Uncultured Megasphaera sp. clone MZH4520 16S ribosomal RNA gene, partial sequence
GACGAACGCTGGCGGCGTGCTTAACACATGCAAGTCGAACGAGAGGATATGGAAAGCTTGCTTTCTATAA
AATCTAGTGGCAAACGGGTGAGTAACGCGTAAACAACCTGCCCTTCGGATGGGGACAACAGCTGGAAACG
GCTGCTAATACCGAATACGCTCTTTTCATCGCATGGTGAGAAGAAGAAAGGACGGCCTCTATACAAAGCG
GTCGCCGAAGGAGGGGTTTGCGTCTGATTAGCTAGTTGGAGGGGTAACGGCCCAACAAAGCGACGATCAG
TAGCCGGTCTGAGAGGATGAACGGCCACATTGGGACTGAGACACGGCCCAGACTCCTACGGGAGGCAGCA
GTGGGGAATCTTCCGCAATGGACGAAAGTCTGACGGAGCAACGCCGCGTGAGTGAAGACGGTCTTCGGAT
TGTAAGGCTCTGTTATACGGGACGAACGGCAAGGTGGTAAATAGCCATCATGAGTGACGGTACCGTAAGA
GAAAGCCACGGCTAACTACGTGCCAGCAGCCGCGGTAATACGTAGGTGGCAAGCGTTGTCCGGAATTATT
GGGCGTAAAGGGCGCGCAGGCGGTTTTTTAAGTCGGTCTTAAAAGTGCGGGGCTTAACCCCGTGAGGGGA
CCGAAACTGGAAGACTTGAGTGTCGGAGAGGAAAGCGGAATTCCTAGTGTAGCGGTGAAATGCGTAGATA
TTAGGAGGAACACCGGTGGCGAAAGCGGCTTTCTGGGCGACAACTGACGCTGAGGCGCGAAAGCGTGGGG
AGCAAACAGGATTAGATACCCTGGTAGTCCACGCCGTAAACGATGGATACTAGGTGTAGGAGGTATCGAC
TCCTTCTGTGCCGTAGTTAACGCTATAAGTATCCCGCCTGGGGAGTACGGCCGCAAGGTTGAAACTCAAA
GGTATTGACGGGGGCCCGCACAAGCGGTGGAGTATGTGGTTTAATTCGACGCAACGCGAAGAACCTTACC
AAGCCTTGACATTGATCGCAATTCTCAGAGATGAGAAGTTCCTCTTCGGAGGACGAGAAAACAGGTGGTG
CACGGCTGTCGTCAGCTCGTGTCGTGAGATGTTGGGTTAAGTCCCGCAACGAGCGCAACCCCTATCTTCT
GTTACCAGCACGTAAAGGTGGGGACTCAGGAGAGACTGCCGCAGACAATGCGGAGGAAGGCGGGGATGAC
GTCAAGTCATCATGCCCCTCATGGCTTGGGCTACACACGTACTACAATGGCTCTTAATAGAGGGAAGCGA
AGGAGTGATCTGGAGCAAACCCCAAAAACAGAGTCTCAGTTCGGATTGTAGGCTGCAACTCGCCTACATG
AAGCAGGAATCGCTAGTAATCGCAGGTCAGCATACTGCGGTGAATACGTTCCCGGGCCTTGTACACACCG
CCCGTCACACCACGAAAGTCATTCACACCCGAAGCCGGTGAGGTAGCCGCAAGGAGCCAGCCGTCGAAGG
TAGGGGTGATGATTGGGGTG
>63146120|gb|AY995255.1|Megasphaera2|Uncultured Megasphaera sp. clone FX8B2-5 16S ribosomal RNA gene, partial sequence
GGGGGCGTGCTTACCATGCAGTCGAACGAGAGGATATGGAAAGCTTGCTTTCTATAAAATCTAGTGGCAA
ACGGGTGAGTAACGCGTAAACAACCTGCCCTTCGGATGGGGACAACAGCTGGAAACGGCTGCTAATACCG
AATACGTTCTTTTCATCGCATGGTGAGAAGAAGAAAGGACGGCCTCTACACAAAGCGGTCGCCGAAGGAG
GGGTTTGCGTCTGATTAGCTAGTTGGAGGGGTAACGGCCCAACAAGGCGACGATCAGTAGCCGGTCTGAG
AGGATGAACGGCCACATTGGGACTGAGACACGGCCCAGACTCCTACGGGAGGCAGCAGTGGGGAATCTTC
CGCAATGGACGAAAGTCTGACGGAGCAACGCCGCGTGAGTGAAGACGGTCTTCGGATTGTAAAGCTCTGT
TATACGGGACGAACGGCAAGGTGGTAAATAGCCATCATGAGTGACGGTACCGTAAGAGAAAGCCACGGCT
AACTACGTGCCAGCAGCCGCGGTAATACGTAGGTGGCAAGCGTTGTCCGGAATTATTGGGCGTAAAGGGC
GCGCAGGCGGTTTTTTAAGTCGGTCTTAAAAGTGCGGGGCTTAACCCCGTGAGGGGACCGAAACTGGAAG
ACTTGAGTGTCGGAGAGGAAAGCGGAATTCCTAGTGTAGCGGTGAAATGCGTAGATATTAGGAGGAACAC
CGGTGGCGAAAGCGGCTTTCTGGACGACAACTGACGCTGAGGCGCGAAAGCGTGGGGAGCAAACAGGATT
AGATACCCTGGTAGTCCACGCCGTAAACGATGGATACTAGGTGTAGGAGGTATCGACTCCTTCTGTGCCG
TAGTTAACGCTATAAGTATCCCGCCTGGGGAGTACGGCCGCAAGGTTGAAACTCAAAGGAATTGACGGGG
GCCCGCACAAGCGGTGGAGTATGTGGTTTAATTCGACGCAACGCGAAGAACCTTACCAAGCCTTGACATT
GATCGCAATTTTCAGAGATGAGAAGTTCCTCTTCGGAGGACGAGAAAACAGGTGGTGCACGGCTGTCGTC
AGCTCGTGTCGTGAGATGTTGGGTTAAGTCCCGCAACGAGCGCAACCCCTATCTTCTGTTACCAGCACGT
AAAGGTGGGGACTCAGGAGAGACTGCCGCAGACAATGCGGAGGAAGGCGGGGATGACGTCAAGTCATCAT
GCCCCTTATGGCTTGGGCTACACACGTACTACAATGGCTCTTAATAGAGGGAAGCGAAGGAGTGATCTGG
AGCAAACCCCAAAAACAGAGTCTCAGTTCGGATTGTAGGCTGCAACTCGCCTACATGAAGCAGGAATCGC
TAGTAATCGCAGGTCAGCATACCTGCCGGG
>110169818|gb|DQ666099.1|Megasphaera2|Uncultured bacterium clone C7 16S ribosomal RNA gene, partial sequence
AGAGTTTGATCCTGGCTCAGGACGAACGCTGGCGGCGTGCTTAACACATGCAAGTCGAACGAGAGGATAT
GGAAAGCTTGCTTTCTATAAAATCTAGTGGCAAACGGGTGAGTAACGCGTAAACAACCTGCCCTTCGGAT
GGGGACAACAGCTGGAAACGGCTGCTAATACCGAATACGTTCTTTTCATCGCATGGTGAGAAGAAGAAAG
GACGGCCTCTACACAAAGCGGTCGCCGAAGGAGGGGTTTGCGTCTGATTAGCTAGTTGGAGGGGTAACGG
CCCAACAAGGCGACGATCAGTAGCCGGTCTGAGAGGATGAACGGCCACATTGGGACTGAGACACGGCCCA

GACTCCTACGGGAGGCAGCAGTGGGGAATCTTCCGCAATGGACGAAAGTCTGACGGAGCAACGCCGCGTG
AGTGAAGACGGTCTTCGGATTGTAAAGCTCTGTTATACGGGACGAACGGCAAGGTGGTAAATAGCCATCA
TGAGTGACGGTACCGTAAGAGAAAGCCACGGCTAACTACGTGCCAGCAGCCGCGGTAATACGTAGGTGGC
AAGCGTTGTCCGGAATTATTGGGCGTAAAGGGCGCGCAGGCGGTTTTTTAAGTCGGTCTTAAAAGTGCGG
GGCTTAACCCCGTGAGGGGACCGAAACTGGAAGACTTGAGTGTCGGAGAGGAAAGCGGAATTCCTAGTGT
AGCGGTGAAATGCGTAGATATTAGGAGGAACACCGGTGGCGAAAGCGGCTTTCTGGACGACAACTGACGC
TGAGGCGCGAAAGCGTGGGGAGCAAACAGGATTAGATACCCTGGTAGTCCACGCCGTAAACGATGGATAC
TAGGTGTAGGAGGTATCGACTCCTTCTGTGCCGTAGTTAACGCTATAAGTATCCCGCCTGGGGAGTACGG
CCGCAAGGTTGAAACTCAAAGGAATTGACGGGGGCCCGCACAAGCGGTGGAGTATGTGGTTTAATTCGAC
GCAACGCGAAGAACCTTACCAAGCCTTGACATTGATCGCAATTTTCAGAGATGAGAAGTTCCTCTTCGGA
GGACGAGAAAACAGGTGGTGCACGGCTGTCGTCAGCTCGTGTCGTGAGATGTTGGGTTAAGTCCCGCAAC
GAGCGCAACCCCTATCTTCTGTTACCAGCACGTAATGGTGGGGACTCAGGAGAGACTGCCGCAGACAATG
CGGAGGAAGGCGGGGATGACGTCAAGTCATCATGCCCCTTATGGCTTGGGCTACACACGTACTACAATGG
CTCTTAATAGAGGGAAGCGAAGGAGCGATCTGGAGCAAACCCCAAAAACAGAGTCTCAGTTCGGATTGTA
GGCTGCAACTCGCCTACATGAAGCAGGAATCGCTAGTAATCGCAGGTCAGCATACTGCGGTGAATACGTT
CCCGGGCCTTGTACACACCGCCCGTCACACCACGAAAGTCATTCACACCCGAAGCCGGTGAGGTAACCGC
AAGGAGCCAGCCGTCGAAGGTAGGGGTGATGATTGGGGTGAAGTCGTAACAAGGTAACC
>CP001849.1|1483562|1485035|Gardnerella|Gardnerella vaginalis 409-05, complete genome
CCCAATCACGAGCCTCACCTTAGACGGCTCCATCCCAAAAGGGTTAGGCCACCGGCTTCGGGTGCTGCCC
ACTTTCATGACTTGACGGGCGGTGTGTACAAGGCCCGGGAACGCATTCACCGCGACGTTGCTGATTCGCG
ATTACTAGCGACTCCGCCTTCACGTAGTCGAGTTGCAGACTACGATCCGAACTGAGACCGGTTTTAAGGG
ATCCGCTCCATGTCACCATGTCGCATCCCGTTGTACCGGCCATTGTAGCATGCGTGAAGCCCTGGACGTA
AGGGGCATGATGATCTGACGTCATCCCCACCTTCCTCCGAGTTAACCCCGGCGGTCCCCCGTGAGTTCCC
GGCATAACCCGCTGGCAACACAGGGCGAGGGTTGCGCTCGTTGCGGGACTTAACCCAACATCTCACGACA
CGAGCTGACGACGACCATGCACCACCTGTGAACCTGCCCCGAAGGGAAACCACATCTCTGCAGTCGACAG
GCACATGTCAAGCCCAGGTAAGGTTCTTCGCGTTGCATCGAATTAATCCGCATGCTCCGCCGCTTGTGCG
GGCCCCCGTCAATTTCTTTGAGTTTTAGCCTTGCGGCCGTACTCCCCAGGCGGGACGCTTAACGCGTTAG
CTCCGACACGGAACCCGTGGAATGGCCCCACATCCAGCGTCCACCGTTTACGGCGTGGACTACCAGGGT
ATCTAATCCTGTTCGCTCCCCACGCTTTCGCTTCTCAGCGTCAGTAACAGCCCAGAGACCTGCCTTCGCC
ATTGGTGTTCTTCCCGATATCTACACATTCCACCGTTACACCGGGAATTCCAGTCTCCCCTACTGCACTC
TAGCCCGCCCGTACCCGGCGCAGACCCACCGTTAAGCGATGGGCTTTCACACCAGACGCGACGAACCGCC
TACAAGCTCTTTACGCCCAATAATTCCGGATAACGCTTGCGCCCTACGTATTACCGCGGCTGCTGGCACG
TAGTTAGCCGGCGCTTATTCGAAAGGTACACTCACCCGAAAGCTTGCTCCCAATCAAAAGCGGTTTACAA
CCCGAAGGCCTTCATCCCGCACGCGGCGTCGCTGCGTCAGGGTTTCCCCCATTGCGCAATATTCCCCACT
GCTGCCTCCCGTAGGAGTCTGGGCCGTATCTCAGTCCCAATGTGGCCGTCCGCCCTCTCAGGCCGGCTAC
CCGTCGAAGCCTAGGTGGGCCATTACCCCGCCTACAAGCTGATAGGACGCGACCCCATCCCATGCCACTA
AACACTTTCCCAACAAGACATGCGTCAAGTTGGAGCATCCAGCATTACCACCCGTTTCCAAGAGCTATTC
TGGAGCATGGGGCAGGTTGGTCACGCATTACTCACCCGTTCGCCACTCTCACCAACCAGCAAGCTGGTCA
GATCCCGTTCGACTTGCATGTGTTAAGCACGCCGCCAGCGTTCATCCTGAGCCAGAATCGAACCCTCCAC
GAAA
>119352239|gb|EF120362.1|Gardnerella|Uncultured Gardnerella sp. clone MZH0805-03 16S ribosomal RNA gene, partial sequence
GATGAACGCTGGCGGCGTGCTTAACACATGCAAGTCGAACGGGATCTGACCAGCTTGCTGGTTGGTGAGA
GTGGCGAACGGGTGAGTAATGCGTGACCAACCTGCCCCATGCTCCAGAATAGCTCTTGGAAACGGGTGGT
AATGCTGGATGCTCCAACTTGACGCATGTCTTGTTGGGAAAGTGTTTAGTGGCATGGGATGGGGTCGCGT
CCTATCAGCTTGTAGGCGGGGTAATGGCCCACCTAGGCTTCGACGGGTAGCCGGCCTGAGAGGGCGGACG
GCCACATTGGGACTGAGATACGGCCCAGACTCCTACGGGAGGCAGCAGTGGGGAATATTGCGCAATGGGG
GAAACCCTGACGCAGCGACGCCGCGTGCGGGATGAAGGCCTTCGGGTTGTAAACCGCTTTTGATTGGGAG
CAAGCTTTCGGGTGAGTGTACCTTTCGAATAAGCGCCGGCTAACTACGTGCCAGCAGCCGCGGTAATACG
TAGGGCGCAAGCGTTATCCGGAATTATTGGGCGTAAAGAGCTTGTAGGCGGTTCGTCGCGTCTGGTGTGA
AAGCCCATCGCTTAACGGTGGGTCTGCGCCGGGTACGGGCGGGCTAGAGTGCAGTAGGGGAGACTGGAAT
TCCCGGTGTAACGGTGGAATGTGTAGATATCGGGAAGAACACCAATGGCGAAGGCAGGTCTCTGGCTGTT
ACTGACGCTGAGAAGCGAAAGCGTGGGGAGCGAACAGGATTAGATACCCTGGTAGTCCACGCCGTAAACG

GTGGACGCTGGATGTGGGGCCCATTCCACGGGTCCCGTGTCGGAGCTAACGCGTTAAGCGTCCCGCCTGG
GGAGTACGGCCGCAAGGCTAAAACTCAAAGAAATTGACGGGGGCCCGCACAAGCGGCGGAGCATGCGGAT
TAATTCGATGCAACGCGAAGAACCTTACCTGGGCTTGACATGTGCCTGTCGACTGCAGAGATGTGGTTTC
CCTTCGGGGCAGGTTCACAGGTGGTGCATGGTCGTCGTCAGCTCGTGTCGTGAGATGTTGGGTTAAGTCC
CGCAACGAGCGCAACCCTTGCCCTGTGTTGCCAGCGGGTTATGCCGGGAACTCACGGGGGACCGCCGGGG
TTAACTCGGAGGAAGGTGGGGATGACGTCGGATCATCATGCCCCTTACGTCCAGGGCTTCACGCATGCTA
CAATGGCCAGTACAACGGGTTGCGACATGGTGACATGGAGCTAATCCCTTAAAACTGGTCTCAGTTCGGA
TCGTAGTCTGCAACTCGACTACGTGAAGGCGGAGTCGCTAGTAATCGCGAATCAACAACGTCGCGGTGAA
TGCGTTCCCGGGCCTTGTACACACCGCCCGTCAAGTCATGAAAGTGGGCAGCACCCGAAGCCGGTGGCCT
GACCTTTTTGGAGGGAGCCGTCTAAGGTGAGGCTCGTGATTGGGACT
>66878517|gb|AY958811.1|Gardnerella|Uncultured bacterium clone rRNA038
16S ribosomal RNA gene, partial sequence
GCCCTTAGAGTTTGATCCTGGCTCAGGATGAACGCTGGCGGCGTGCTTAACACATGCAAGTCGAACGGGA
TCTGACCAGCTTGCTGGTTGGTGAGAGTGGCGAACGGGTGAGTAATGCGTGACCAACCTGCCCCATGCTC
CAGAATAGCTCTTGGAAACGGGTGGTAATGCTGGATGCTCCAACTTGACGCATGTCTTGTTGGGAAAGTG
TTTAGTGGCATGGGATGGGGTCGCGTCCTATCAGCTTGTAGGCGGGGTAATGGCCCACCTAGGCTTCGAC
GGGTAGCCGGCCTGAGAGGGCGGACGGCCACATTGGGACTGAGATACGGCCCAGACTCCTACGGGAGGCA
GCAGTGGGGAATATTGCGCAATGGGGGAAACCCTGACGCAGCGACGCCGCGTGCGGGATGAAGGCCTTCG
GGTTGTAAACCGCTTTTGATTGGGAGCAAGCCTTCGGGTGAGTGTACCTTTCGAATAAGCGCCGGCTAAC
TACGTGCCAGCAGCCGCGGTAATACGTAGGGCGCAAGCGTTATCCGGAATTATTGGGCGTAAAGAGCTTG
TAGGCGGTTCGTCGCGTCTGGTGTGAAAGCCCATCGCTTAACGGTGGGTCTGCGCCGGGTACGGGCGGGC
TAGAGTGCAGTAGGGGAGACTGGAATTCCCGGTGTAACGGTGGAATGTGTAGATATCGGGAAGAACACCA
ATGGCGAAGGCAGGTCTCTGGGCTGTTACTGACGCTGAGAAGCGAAAGCGTGGGGAGCGAACAGGATTAG
ATACCCTGGTAGTCCACGCCGTAAACGGTGGACGCTGGATGTGGGGCCCATTCCACGGGTTCCGTGTCGG
AGCTAACGCGTTAAGCGTCCCGCCTGGGGAGTACGGCCGCAAGGCTAAAACTCAAAGAAATTGACGGGGG
CCCGCACAAGCGGCGGAGCATGCGGATTAATTCGATGCAACGCGAAGAACCTTACCTGGGCTTGACATGT
GCCTGTCGACTGCAGAGATGTGGTTTCCCTTCGGGGCAGGTTCACAGGTGGTGCATGGTCGTCGTCAGCT
CGTGTCGTGAGATGTTGGGTTAAGTCCCGCAACGAGCGCAACCCTTGCCCTGTGTTGCCAGCGGGTTATG
CCGGGAACTCACGGGGGACCGCCGGGGTTAACTCGGAGGAAGGTGGGGATGACGTCAGATCATCATGCCC
CTTACGTCCAGGGCTTCACGCATGCTACAATGGCCAGTACAACGGGTTGCGACATGGTGACATGGAGCTA
ATCCCTTAAAACTGGTCCCAGTTCGGATCGTAGTCTGCAACTCGACTACGTGAAGGCGGAGTCGCTAGTA
ATCGCGAATCAGCAACGTCGCGGTGAATGCGTTCCCGGGCCTTGTACACACCGCCCGTCAAGTCATGAAA
GTGGGCAGCACCCGAAGCCGGTGGCCTGACCTTTTTGGAGGGAGCCGTCTAAGGTGAGGCTCGTGATTGG
GACTAAGTCGTAACAAGGTAACCGTAAAGGGCG
>66878877|gb|AY959171.1|Gardnerella|Uncultured bacterium clone rRNA398
16S ribosomal RNA gene, partial sequence
GCCCTTAGAGTTTGATCCTGGCTCACGATGAACGATGGCGGCGTGCTTAACACATGCAAGTCGAACGGGA
TCTGACCAGCTTGCTGGTTGGTGAGAGTGGCGAACGGGTGAGTAATGCGTGACCAACCTGCCCCATGCTC
CAGAATAGCTCTTGGAAACGGGTGGTAATGCTGGATGCTCCAACTTGACGCATGTCTTGTTGGGAAAGTG
TTTAGTGGCATGGGATGGGGTCGCGCCCTATCAGCTTGTAGGCGGGGTAATGGCCCACCTAGGCTTCGAC
GGGTAGCCGGCCTGAGAGGGCGGACGGCCACATTGGGACTGAGATACGGCCCAGACTCCTACGGGAGGCA
GCGGTGGGGAATATTGCGCAATGGGGGAAACCCTGACGCAGCGACGCCGCGTGCGGGATGAAGGCCTTCG
GGTTGTAAACCGCTTTTGATTGGGAGCAAGCCTTCGGGTGAGTGTACCTTTCGAATAAGCGCCGGCTAAC
TACGTGCCAGCAGCCGCGGTAATACGTAGGGCGCAAGCGTTATCCGGAATTATTGGGCGTAAAGAGCTTG
TAGGCGGTTCGTCGCGTCTGGTGTGAAAGCCCATCGCTTAACGGTGGGTCTGCGCCGGGTACGGGCGGGC
TAGAGTGCAGTAGGGGAGACTGGAATTCCCGGTGTAACGGTGGAATGTGTAGATATCGGGAAGAACACCA
ATGGCGAAGGCAGGTCTCTGGGCTGTTACTGACGCTGAGAAGCGAAAGCGTGGGGAGCGAACAGGATTAG
ATACCCTGGTAGTCCACGCCGTAAACGGTGGACGCTGGATGTGGGGCCCATTCCACGGGTTCCGTGTCGG
AGCTAACGCGTTAAGCGTCCCGCCTGGGGAGTACGGCCGCAAGGCTAAAACTCAAAGAAATTGACGGGGG
CCCGCACAAGCGGCGGAGCATGCGGATTAATTCGATGCAACGCGAAGAACCTTACCTGGGCTTGACATGT
GCCTGTCGACTGCAGAGATGTGGTTTCCCTTCGGGGCAGGTTCACAGGTGGTGCATGGTCGTCGTCAGCT
CGTGTCGTGAGATGTTGGGTTAAGTCCCGCAACGAGCGCAACCCTCGCCCTGTGTTGCCAGCGGGTTATG
CCGGGAACTCACGGGGGACCGCCGGGGTTAACTCGGAGGAAGGTGGGGATGACGTCAGATCATCATGCCC
CTTACGTCCAGGGCTTCACGCATGCTACAATGGCCGGTACAACGGGATGCGACATGGTGACATGGAGCGG

ATCCCTTAAAACCGGTCTCAGTTCGGATCGTAGTCTGCAACTCGACTACGTGAAGGCGGAGTCGCTAGTA
ATCGCGAATCAGCAACGTCGCGGTGAATGCGTTCCCGGGCCTTGTACACACCGCCCGTCAAGTCATGAAA
GTGGGCAGCACCCGAAGCCGGTGGCCTAACCCTTTTGGGATGGAGCCGTCTAAGGTGAGGCTCGTGATTG
GGACTAAGTCGTAACAAGGTAACCGTAAAGGGCGA
>119352240|gb|EF120363.1|Sneathia|Uncultured Sneathia sp. clone MZH0805-40 16S ribosomal RNA gene, partial sequence
GATAAACGCTGACAGAATGCTTAACACATGCAAGTCGATGATGGGAGCTAGCTTGCTAGAAGAAGTCATG
GCGGACGGGTGAGTAACGTGTAAAGAACTTACCATATAGACTGGGATAACAGAGGGAAACTTCTGATAAT
ACTGGATAAGTTAGTAGTAGCATTACTAAGTAATGAAAGGTAGCAATACGCTATATGAGAGCTTTGCATC
CTATTAGCTAGTTGGTGGGGTAAAAGCCTACCAAGGCGATGATAGGTAGCCGGCCCGAGAGGGTGGACGG
CCACAAGGGGACTGAGATACGGCCCTTACTCCTACGGGAGGCAGCAGTGGGGAATATTGGACAATGGAGG
CAACTCTGATCCAGCAATTCTGTGTGTGTGAAGAAGGTTTTAGGACTGTAAAACACTTTTAGTAGGGAAG
AAATAAATGACGGTACCTACAGAAGAAGCGACGGCTAAATACGTGCCAGCAGCCGCGGTAATACGTATGT
CGCGAGCGTTATCCGGAATTATTGGGCCTAAAGGGCATCTAGGCGGTAAGACAAGTTGAAGGTGAAAACC
TGTGGCTCAACCATAGGCTTGCCTACAAAACTGTTGAACTAGAGTACTGGAAAGGTGGGTGGAACTACAC
GAGTAGAGGTGAAATTCGTAGATATGTGTAGGAATGCCGATGTTGAAGATAACTCACTGGACAGCAACTG
ACGCTGAAGTGCGAAAGCTAGGGGAGCAAACAGGATTAGATACCCTGGTAGTCCTAGCTGTAAACGATGA
TCACTGGGTGTGGGGATTCGAAGTCTCTGTGCCGAAGCAAAAGCGATAAGTGATCCGCCTGGGGAGTACG
TTCGCAAGAATGAAACTCAAAGGAATTGACGGGGACCCGCACAAGTGGTGGAGCATGTGGTTTAATTCGA
CGCAACGCGAGGAACCTTACCAGATCTTGACATCCTCCGAAGAGCATAGAAGTATGCTTGTGCCTACGGG
AACGGAGAGACAGGTGGTGCACGGCTGTCGACAGCTCGTGTTGTGAGATGTTGGGTTAAGTCCCGCAACG
AGCGAAACCCCTATCATTAGTTACCATCATTAAGTTGGGGACTCTAATGAAACTGCCTACGAAGAGTAGG
AGGAAGGTGGGGATGACGTCAAGTCATCATGCCCCTTATGATCTGGGCTACACACGTGCTACAATGGGTA
GTACAAAGAGAAGCTTTGTAGCGATACATGGCGAAACTTAAAAAGCTATTCTTAGTTCGGATTGAAGTCT
GCAACTCGACTTCATGAAGTTGGAATCACTAGTAATCGTGAATCAGCAATGTCACGGTGAATACGTTCTC
GGGCCTTGTACACACCGCCCGTCACACCACGAGAGTTGTTTGCACCTGAAATTACCGGCCTAACCGTAAG
GAGGGAGGTACTGAAGGTGTGGATAGTGATTGGGGTG
>219878348|ref|NR_025487.1|Sneathia|Sneathia sanguinegens strain CCUG 41628 16S ribosomal RNA, partial sequence
TGGCTCAGGATAAACGCTGACAGAATGCTTAACACATGCAAGTCGATGATGGGAGCTAGCTTGCTAGAAG
AAGTCATGGCGGACGGGTGAGTAACGTGTAAAGAACTTACCATATAGACTGGGATAACAGAGGGAAACTT
CTGATAATACTGGATAAGTTAGTAGTAGCATTACTAAGTAATGAAAGGTAGCAATACGCTATATGAGAGC
TTTGCATCCTATTAGCTAGTTGGTGGGGTAAAAGCCTACCAAGGCGATGATAGGTAGCCGGCCTGAGAGG
GTGGACGGCCACAAGGGGACTGAGATACGGCCCTTACTCCTACGGGAGGCAGCAGTGGGGAATATTGGAC
AATGGAGGCAACTCTGATCCAGCAATTCTGTGTGTGTGAAGAAGGTTTTAGGACTGTAAAACACATTTTA
GTAGGGAAGAAAGAAATGACGGTACCTACAGAAGAAGCGACGGCTAAATACGTGCCAGCAGCCGCGGTAA
TACGTATGTCGCGAGCGTTATCCGGAATTATTGGGCTTAAAGGGCATCTAGGCGGTTAAACAAGTTGAAG
GTGAAAACCTGTGGCTCAACCATAGGCTTGCCTACAAAACTGTATAACTAGAGTACTGGAAAGGTGGGTG
GAACTACACGAGTAGAGGTGAAATTCGTAGATATGTGTAGGAATGCCGATGATGAAGATAACTCACTGGA
CAGCAACTGACGCTGAAGTGCGAAAGCTAGGGGAGCAAACAGGATTAGATACCCTGGTATCCTAGCTGTA
AACGATGATCACTGGGTGTGGGGATTCGAAGTCTCTGTGCCGAAGCAAAAGCGATAAGTGATCCGCCTGG
GGAGTACGTTCGCAAGAATGAAACTCAAAGGAATTGACGGGGACCCGCACAAGTGGTGGAGCATGTGGTT
TAATTCGACGCAACGCGAGGAACCTTACCAGATCTTGACATCCTCCGAAGAGCATAGAAGTATGCTTGTG
CCTACGGGAACGGAGAGACAGGTGGTGCATGGCTGTCCACAGCTCNTGTTGTGAGATGTTGGGTTAAGTC
CCGCAACGAGCGAAACCCCTATCATTAGTTACCATCATTAAGTTGGGGACTCTAATGAAACTGCCTACGA
AGAGTAGGAGGAAGGTGGGGATGACGTCAAGTCATCATGCCCCTTATGATCTGGGCTACACACGTGCTAC
AATGGATAGTACAAAGAGAAGCTTTGTAGCGATACATGGCAAAACTAAGAAAGCTATTCTTAGTTCGGAT
TGAAGTCTGCAACTCGACTTCATGAAGTTGGNATCACTAGTAATCGTGAATCAGCAATGTCACGGTGAAT
ACGTTCTCGGGGTCTTGTACACACCGCCCGTC
>66878620|gb|AY958914.1|Sneathia|Uncultured bacterium clone rRNA141 16S ribosomal RNA gene, partial sequence
CCCTTAGAGTTTGATCCTGGCTCAGGATAAACGCTGACAGAATGCTTAACACATGCAAGTCGATGATGGG
AGCTAGCTTGCTAGAAGAAGTCATGGCGGACGGGTGAGTAACGTGTAAAGAACTTACCATATAGACTGGG
ATAACAGAGGGAAACTTCTGATAATACTGGATAAGTTAGAAGTAGCATTACTAAGTAATGAAAGGTAGAA

ATACGCTATATGAGAGCTTTGCATCCTATTAGCTAGTTGGTGGGGTAAAAGCCTACCAAGGCGATGATAG
GTAGCCGGCCTGAGAGGGTGGACGGCCACAAGGGGACTGAGATACGGCCCTTACTCCTACGGGAGGCAGC
AGTGGGGAATATTGGACAATGGAGGCAACTCTGATCCAGCAATTCTGTGTGTGTGAAGAAGGTTTTAGGA
CTGTAAAACACTTTTAGTAGGGAAGAAAAAATGACGGTACCTACAGAAGAAGCGACGGCTAAATACGTGC
CAGCAGCCGCGGTAATACGTATGTCGCGAGCGTTATCCGGAATTATTGGGCTTAAAGGGCATCTAGGCGG
TTAAACAAGTTGAAGGTGAAAACCTGTGGCTCAACCATAGGCTTGCCTACAAAACTGTATAACTAGAGTA
CTGGAAAGGTGGGTGGAACTACACGAGTAGAGGTGAAATTCGTAGATATGTGTAGGAATGCCGATGATGA
AGATAACTCACTGGACAGCAACTGACGCTGAAGTGCGAAAGCTAGGGGAGCAAACAGGATTAGATACCCT
GGTAGTCCTAGCTGTAAACGATGATCACTGGGTGTGGGGATTCGAAGTCTCTGTGCCGAAGCAAAAGCGA
TAAGTGATCCGCCTGGGGAGTACGTTCGCAAGAATGAAACTCAAAGGAATTGACGGGGACCCGCACAAGT
GGTGGAGCATGTGGTTTAATTCGACGCAACGCGAGGAACCTTACCAGATCTTGACATCCTCCGAAGAGCA
TAGAAGTATGCTTGTGCCTACGGGAACGGAGAGACAGGTGGTGCATGGCTGTCGACAGCTCGTGTTGTGA
GATGTTGGGTTAAGTCCCGCAACGAGCGAAACCCCTATCATTAGTTACCATCATTAAGTTGGGGACTCTA
ATGAAACTGCCTACGAAGAGTAGGAGGAAGGTGGGGATGACGTCAAGTCATCATGCCCCTTATGATCTGG
GCTACACACGTGCTACAATGGATAGTACAAAGAGAAGCTTTGTAGCGATACATGGCAAAACTAAGAAAGC
TATTCTTAGTTCGGATTGAAGTCTGCAACTCGACTTCATGAAGTTGGAATCACTAGTAATCGTGAATCAG
CAATGTCACGGTGAATACGTTCTCGGGTCTTGTACACACCGCCCGTCACACCACGAGAGTTGTTTGTACC
TGAAATTACTGGCCTAACTGCAAAGGGGGAGGTACTGAAGGTGTGGATAGCGATTGGGGTGAAGTCGTAA
CAAGGTAACCGTAAAGGG
>239924008|gb|GQ179730.1|Sneathia|Uncultured Sneathia sp. clone VE12E10 16S ribosomal RNA gene, partial sequence
GATAAACGCTGACAGAATGCTTAACACATGCAAGTCGATGATGGGAGCTAGCTTGCTAGAAGAAGTCATG
GCGGACGGGTGAGTAACGTGTAAAGAACTTACCATATAGACTGGGATAACAGAGGGAAACTTCTGATAAT
ACTGGATAAGTTAGTAGTAGCATTACTAAGTAATGAAAGGTAGTAATACGCTATATGAGAGCTTTGCATC
CTATTAGCTAGTTGGTGGGGTAAAAGCCTACCAAGGCGATGATAGGTAGCCGGCCTGAGAGGGTGGACGG
CCACAAGGGGACTGAGATACGGCCCTTACTCCTACGGGAGGCAGCAGTGGGGAATATTGGACAATGGAGG
CAACTCTGATCCAGCAATTCTGTGTGTGTGAAGAAGGTTTTAGGACTGTAAAACACTTTTAGTAGGGAAG
AAAGAAATGACGGTACCTACAGAAGAAGCGACGGCTAAATACGTGCCAGCAGCCGCGGTAATACGTATGT
CGCGAGCGTTATCCGGAATTATTGGGCTTAAAGGGCATCTAGGCGGTTAAACAAGTTGAAGGTGAAAACC
TGTGGCTCAACCATAGGCTTGCCTACAAAACTGTATAACTAGAGTACTGGAAAGGTGGGTGGAACTACAC
GAGTAGAGGTGAAATTCGTAGATATGTGTAGGAATGCCGATGATGAAGATAACTCACTGGACAGCAACTG
ACGCTGAAGTGCGAAAGCTAGGGGAGCAAACAGGATTAGATACCCTGGTAGTCCTAGCTGTAAACGATGA
TCACTGGGTGTGGGGATTCGAAGTCTCTGTGCCGAAGCAAAAGCGATAAGTGATCCGCCTGGGGAGTACG
TTCGCAAGAATGAAACTCAAAGGAATTGACGGGGACCCGCACAAGTGGTGGAGCATGTGGTTTAATTCGA
CGCAACGCGAGGAACCTTACCAGATCTTGACATCCTCCGAAGAGCATAGAAGTATGCTTGTGCCTACGGG
AACGGAGAGACAGGTGGTGCATGGCTGTCGACAGCTCGTGTTGTGAGATGTTGGGTTAAGTCCCGCAACG
AGCGAAACCCCTATCATTAGTTACCATCATTAAGTTGGGGACTCTAATGAAACTGCCTACGAAGAGTAGG
AGGAAGGTGGGGATGACGTCAAGTCATCATGCCCCTTATGATCTGGGCTACACACGTGCTACAATGGATA
GTACAAAGAGAAGCTTTGTAGCGATACATGGCAAAACTAAGAAAGCTATTCTTAGTTCGGATTGAAGTCT
GCAACTCGACTTCATGAAGTTGGAATCACTAGTAATCGTGAATCAGCAATGTCACGGTGAATACGTTCTC
GGGTCTTGTACACACCGCCCGTCACACCACGAGAGTTGTTTGCACCTGAAATTACTGGCCTAACTGCAAA
GGGGGAGGTACTGAAGGTGTGGATAGCGATTGGGGTGAAGTCGTAACAAGGTA
>45476684|gb|AY489565.1|Leptotrichia|Leptotrichia amnionii 16S ribosomal RNA gene, partial sequence
CCCTGTACGACTTCACCCCAATCACTATCCACACCTTCAGTACCTCCCTCCTTCGGTTAGGCCGGTAATT
TCAGGTGCAAACAACTCTCGTGGTGTGACGGGCGGTGTGTACAAGGCCCGAGAACGTATTCACCGTGACA
TTGCTGATTCACGATTACTAGTGATTCCAACTTCATGAAGTCGAGTTGCAGACTTCAATCCGAACTAAGA
ATAGCTTTTTAAGTTTCGCCATGTATCGCTACAAAGCTTCTCTTTGTACTACCCATTGTAGCACGTGTGT
AGCCCAGATCATAAGGGGCATGATGACTTGACGTCATCCCCACCTTCCTCCTACTCTTCGTAGGCAGTTT
CATTAGAGTCCCCAACTTAATGATGGCAACTAATGATAGGGGTTTCGCTCGTTGCGGGACTTAACCCAAC
ATCTCACAACACGAGCTGTCGACAGCCATGCACCACCTGTCTCTCGGTTCCCGAAGGCACAAGTATACTT
CTATACTCTCCCGAGGATGTCAAGATCTGGTAAGGTTCCTCGCGTTGCGTCGAATTAAACCACATGCTCC
ACCACTTGTGCGGGCCCCCGTCAATTCCTTTGAGTTTCATTCTTGCGAACGTACTCCCCAGGCGGATCAC
TTATCGCTTTTGCTTCGGCACAGAGACTTCGCATCCCCACACCCAGTGATCATCGTTTACAGCTAGGACT

ACCAGGGTATCTAATCCTGTTTGCTCCCCTAGCTTTCGCACTTCAGCGTCAGTTTCTGTCCAGTGAGTTA
TCTTCATCATCGGCATTCCTACACATATCTACGAATTTCACCTCTACTCGTGTAGTTCCACCCACCTTTC
CAGTACTCTAGTTCAACAGTTTTGTAGGCAAGCCTATGGTTGAGCCACAGGTTTTCACCTTCAACTTGTC
TTACCGCCTAGATGCCCTTTAAGCCCAATAATTCCGGATAACGCTCGCAACATACGTATTACCGCGGCTG
CTGGCACGTATTTAGCCGTTGCTTCTTCTGTAGGTACCGTCATTTTTTTCTTCCCTACTAAAAGTGTTTT
ACAGTCCTAAAACCTTCTTCACACACACAGAATTGCTGGATCAGAGTTTCCTCCATTGTCCAATATTCCC
CACTGCTGCCTCCCGTAGGAGTAAGGGCCGTATCTCAGTCCCCTTGTGGCCGTCCACCCTCTCAGGCCGG
CTACCTATCATCGCCTTGGTAAGCTCTTACCTTACCAACTAGCTAATAGGACGCAAAGCTCTCCTTTAGT
GCCGTAGCTTTCATTTGCATATCATGCGATATACTAACTTATCCAGTATTATCAGAAGTTTCCCTCTGTT
ATCCCAGTCTAAAGGGCAAGTTCTTTACGCGTTACTCACCCGTCCGCCATGAGTCCATTTAGCAAGCTAA
ACTTCCTCATAGACTTGCATGTGTTAAGCATTCTGTCAGCGTTTATCCTGAGCCAGGATCAAACTCTA
>119352241|gb|EF120364.1|Leptotrichia|Uncultured Leptotrichia sp. clone MZH0805-11 16S ribosomal RNA gene, partial sequence
GATAAACGCTGACAGAATGCTTAACACATGCAAGTCTATGAGGAAGTTTAGCTCGCTAAATGGACTCATG
GCGGACGGGTGAGTAACGCGTAAAGAACTTGCCCTTTAGACTGGGATAACAGAGGGAAACTTCTGATAAT
ACTGGATAAGTTAGTATATCGCATGATATGCAAATGAAAGCTACGGCACTAAAGGAGAGCTTTGCGTCCT
ATTAGCTAGTTGGTAAGGTAAGAGCTTACCAAGGCGATGATAGGTAGCCGGCCTGAGAGGGTGGACGGCC
ACAAGGGGACTGAGATACGGCCCTTACTCCTACGGGAGGCAGCAGTGGGGAATATTGGACAATGGAGGAA
ACTCTGATCCAGCAATTCTGTGCGTGTGAAGAAGGTTTTAGGACTGTAAAACACTTTTAGTAGGGAAGAA
AAAAATGACGGTACCTACAGAAGAAGCAACGGCTAAATACGTGCCAGCAGCCGCGGTAATACGTATGTTG
CGAGCGTTATCCGGAATTATTGGGCTTAAAGGGCATCTAGGCGGTAAGACAAGTTGAAGGTGAAAACCTG
TGGCTCAACCATAGGCTTGCCTACAAAACTGTTGAGCTAGAGTACTGGAAAGGTGGGTGGAACTACACGA
GTAGAGGTGAAATTCGTAGATATGTGTAGGAATGCCGATGATGAAGATAACTCACTGGACAGAAACTGAC
GCTGAAGTGCGAAAGCTAGGGGAGCAAACAGGATTAGATACCCTGGTAGTCCTAGCTGTAAACGATGATC
ACTGGGTGTGGGGATGCGAAGTCTCTGTGCCGAAGCAAAAGCGATAAGTGATCCGCCTGGGGAGTACGTT
CGCAAGAATGAAACTCAAAGGAATTGACGGGGGCCCGCACAAGTGGTGGAGCATGTGGTTTAATTCGACG
CAACGCGGGGAACCTTACCAGATCTTGACATCCTCGGGAGAGTATAGAAGTATACTTGTGCCTTCGGGAA
CCGAGAGACAGGTGGTGCATGGCTGTCGACAGCTCGTGTTGTGAGATGTTGGGTTAAGTCCCGCAACGAG
CGAAACCCCTATCATTAGTTGCCACCATTAAGTTGGGGACTCTAATGAAACTGCCTACGAAGAGTAGGAG
GAAGGTGGGGATGACGTCAAGTCATCATGCCCCTTATGATCTGGGCTACACACGTGCTACAATGGGTAGT
ACAAAGAGAAGCTTTGTAGCGATACATGGCGAGACTTAAAAGGCTATTCTTAGTTCGGATTGAAGTCTGC
AACTCGACTTCATGAAGTTGGAATCACTAGTAATCGTGAATCAGCAATGTCACGGTGAATACGTTCTCGG
GCCTTGTACACACCGCCCGTCACACCACGAGAGTTGTTTGCACCTGAAATTACCGGCCTAACCGTAAGGA
GGGAGGTACTGAAGGTGTGGATAGTGATTGGGGTG
>186915011|gb|EU644469.1|Leptotrichia|Uncultured Leptotrichia sp. clone 531 16S ribosomal RNA gene, partial sequence
GTTTGATCCTGGCTCAGGATAAACGCTGACAGAATGCTTAACACATGCAAGTCTATGAGGAAGTTTAGCT
TGCTAAATGGACTCATGGCGGACGGGTGAGTAACGCGTAAAGAACTTGCCCTTTAGACTGGGATAACAGA
GGGAAACTTCTGATAATACTGGATAAGTTAGTATATCGCATGATATGCAAATGAAAGCTACGGCACTAAA
GGAGAGCTTTGCGTCCTATTAGCTAGTTGGTAAGGTAAGAGCTTACCAAGGCGATGATAGGTAGCCGGCC
TGAGAGGGTGGACGGCCACAAGGGGACTGAGATACGGCCCTTACTCCTACGGGAGGCAGCAGTGGGGAAT
ATTGGACAATGGAGGGAACTCTGATCCAGCAATTCTGTGTGTGTGAAGAAGGTTTTAGGACTGTAAAACA
CTTTTAGTAGGGAAGAAAAAAATGACGGTACCTACAGAAGAAGCAACGGCTAAATACGTGCCAGCAGCCG
CGGTAATACGTATGTTGCGAGCGTTATCCGGAATTATTGGGCTTAAAGGGCATCTAGGCGGTAAGACAAG
TTGAAGGTGAAAACCTGTGGCTCAACCATAGGCTTGCCTACAAAACTGTTGAACTAGAGTACTGGAAAGG
TGGGTGGAACTACACGAGTAGAGGTGAAATTCGTAGATATGTGTAGGAATGCCGATGATGAAGATAACTC
ACTGGACAGAAACTGACGCTGAAGTGCGAAAGCTAGGGGAGCAAACAGGATTAGATACCCTGGTAGTCCT
AGCTGTAAACGATGATCACTGGGTGTGGGGATGCGAAGTCTCTGTGCCGAAGCAAAAGCGATAAGTGATC
CGCCTGGGGAGTACGTTCGCAAGAATGAAACTCAAAGGAATTGACGGGGGCCCGCACAAGTGGTGGAGCA
TGTGGTTTAATTCGACGCAACGCGAGGAACCTTACCAGATCTTGACATCCTCGGGAGAGTATAGAAGTAT
ACTTGTGCCTTCGGGAACCGAGAGACAGGTGGTGCATGGCTGTCGACAGCTCGTGTTGTGAGATGTTGGG
TTAAGTCCCGCAACGAGCGAAACCCCTATCATTAGTTGCCATCATTAAGTTGGGGACTCTAATGAAACTG
CCTACGAAGAGTAGGAGGAAGGTGGGGATGACGTCAAGTCATCATGCCCCTTATGATCTGGGCTACACAC
GTGCTACAATGGGTAGTACAAAGAGAAGCTTTGTAGCGATACATGGCGAAACTTAAAAAGCTATTCTTAG

TTCGGATTGAAGTCTGCAACTCGACTTCATGAAGTTGGAATCACTAGTAATCGTGAATCAGCAATGTCAC
GGTGAATACGTTCTCGGGCCTTGTACACACCGCCCGTCACACCACGAGAGTTGTTTGCACCTGAAATTAC
CGGCCTAACCGTAAGGAGGGAGGTACTGAAGGTGTGGATAGTGATTGGGGTGAAGTCGTAACAAGGTA
>119352242|gb|EF120365.1|Peptostreptococcus|Uncultured Peptostreptococcus sp. clone MZH0805-43 16S ribosomal RNA gene, partial sequence
GACGAACGCTGGCGGCGTGCTTAACACATGCAAGTCGAACGAGAGTTAGATCGAATGAGTTTTCGGACAA
GTGAGATTTAACGAAAGTGGCGAACGGGTGAGTAACACGTGAGCAACCTGCCTTACACAGGGGGATAGCC
ATTGGAAACGGTGATTAATACCCCATAAGACCACAATACCGCATGGTAAAAGGGTAAAAGGGATACCGGT
GTAAGATGGGCTCGCGTCTGATTAGCTAGTTGGTGGGGTAAAGGCCTACCAAGGCGACGATCAGTAGCCG
GTCTGAGAGGATGAACGGCCACATTGTAACTGAGACACGGTCCAAACTCCTACGGGAGGCAGCAGTGGGG
AATATTGCACAATGGGGGAAACCCTGATGCAGCGACGCCGCGTGAGCGAAGAAGGCTTTCGAGTCGTAAA
GCTCTGTCCTATGAGAAGATAATGACGGTATCATAGGAGGAAGCCCTGGCTAAATACGTGCCAGCAGCCG
CGGTAATACGTATGGGGCGAGCGTTGTCCGGAATTATTGGGCGTAAAGGGTACGTAGGCGGTTTTTTAAG
TCAGGTGTCAAAGCGTGGAGCTTAACTCCATTAAGCACTTGAAACTGAAAGACTTGAGTGAAGGAGAGGA
AAGTGGAATTCCTAGTGTAGCGGTGAAATGCGTAGATATTAGGAGGAATACCGGTGGCGAAGGCGACTTT
CTGGACTTTTACTGACGCTCAGGTACGAAAGCGTGGGGAGCAAACAGGATTAGATACCCTGGTAGTCCAC
GCCGTAAACGATGAATGCTAGGTGTTGGGAGTCAAATCTCGGTGCCGAAGTTAACACATTAAGCATTCCG
CCTGGGGAGTACGGTGGCAACACTGAAACTCAAAGGAATTGACGGGGACCCGCACAAGCAGCGGAGCATG
TGGTTTAATTCGAAGCAACGCGAAGAACCTTACCAAGGCTTGACATATAGTTGAGTTATTGAGAAATTGA
TAAGTCCCTCGGGACAACTATACAGGTGGTGCATGGTTGTCGTCAGCTCGTGTCGTGAGATGTTGGGTTA
AGTCCCGCAACGAGCGCAACCCTTATTTTCAGTTATCAGCATTTAAGGTGGGGACTCTGAAGAGACTGCC
GATGACAAATCGGAGGAAGGTGGGGATGACGTCAAATCATCATGCCCTTTATGTCTTGGGCTACACACGT
GCTACAATGGTCGGTACAACGAGAAGCGAGATAGTGATGTTAAGCGAAACTCTAAAAGCCGATCTCAGTT
CGGATTGTAGGCTGCAACTCGCCTACATGAAGTCGGAGTTGCTAGTAATCGCGAATCAGAACGTCGCGGT
GAATGCGTTCCCGGGTCTTGTACACACCGCCCGTCACACCATGGGAGCTTGTAATACCCGAAGCCGTCGA
TCCAACCGCAAGGAGGAAGACGTCGAAGGCAGGACAAGTGACTGGGGTG
>66878646|gb|AY958940.1|Peptostreptococcus|Uncultured bacterium clone rRNA167 16S ribosomal RNA gene, partial sequence
CGCCCTTAGAGTTTGATCCTGGCTCAGGACGAACGCTGGCGGCGTGCTTAACACATGCAAGTCGAACGAG
AGTTAGATCGAATGAGTTTTCGGACAAGTGAGATTTAACGAAAGTGGCGAACGGGTGAGTAACACGTGAG
CAACCTGCCTTACACAGGGGGATAGCCATTGGAAACGGTGATTAATACCCCATAAGACCACAATACCGCA
TGGTAAAAGGGTAAAAGGGATACCGGTGTAAGATGGGCTCGCGTCTGATTAGCTAGTTGGTGGGGTAAAG
GCCTACCAAGGCGACGATCAGTAGCCGGTCTGAGAGGATGAACGGCCACATTGGAACTGAGACACGGTCC
AAACTCCTACGGGAGGCAGCAGTGGGGAATATTGCACAATGGGGGAAACCCTGATGCAGCGACGCCGCGT
GAGCGAAGAAGGCTTTCGAGTCGTAAAGCTCTGTCCTATGAGAAGATAATGACGGTATCATAGGAGGAAG
CCCTGGCTAAATACGTGCCAGCAGCCGCGGTAATACGTATGGGGCGAGCGTTGTCCGGAATTATTGGGCG
TAAAGGGTACGTAGGCGGTTTTTTAAGTCAGGTGTCAAAGCGTGGAGCTTAACTCCATTAAGCACTTGAA
ACTGAAAGACTTGAGTGAAGGAGAGGAAAGTGGAATTCCTAGTGTAGCGGTGAAATGCGTAGATATTAGG
AGGAATACCGGTGGCGAAGGCGACTTTCTGGACTTTTACTGACGCTCAGGTACGAAAGCGTGGGGAGCAA
ACAGGATTAGATACCCTGGTAGTCCACGCCGTAAACGATGAATGCTAGGTGTTGGGAGTCAAATCTCGGT
GCCGAAGTTAACACATTAAGCATTCCGCCTGGGGAGTACGGTGGCAACACTGAAACTCAAAGGAATTGAC
GGGGACCCGCACAAGCAGCGGAGCATGTGGTTTAATTCGAAGCAACGCGAAGAACCTTACCAAGGCTTGA
CATATAGTTGAGTTATTGAGAAATTGATAAGTCCCTCGGGACAACTATACAGGTGGTGCATGGTTGTCGT
CAGCTCGTGTCGTGAGATGTTGGGTTAAGTCCCGCAACGAGCGCAACCCTTATTTTCAGTTACCAGCATT
TAAGGTGGGGACTCTGAAGAGACTGCCGATGACAAATCGGAGGAAGGTGGGGATGACGTCAAATCATCAT
GCCCTTTATGTCTTGGGTTACACACGTGCTACAATGGTCGGTACAACGAGAAGCGAGATAGTGATGTTAA
GCGAAACTCTAAAAGCCGATCTCAGTTCGGATTGTAGGCTGCAACTCGCCTACATGAAGTCGGAGTTGCT
AGTAATCGCGAATCAGAACGTCGCGGTGAATGCGTTCCCGGGTCTTGTACACACCGCCCGTCACACCATG
GGAGCTTGTAATACCCGAAGCCGTCGATCCAACCGCAAGGAGGAAGACGTCGAAGGCAGGACAAGTGACT
GGGGTGAAGTCGTAACAAGGTAACCGTA
>239923970|gb|GQ179692.1|Peptostreptococcus|Uncultured Peptostreptococcus sp. clone VE58A09 16S ribosomal RNA gene, partial sequence
GACGAACGCTGGCGGCGTGCTTAACACATGCAAGTCGAACGAGAGTTAGATCGAATGAGTTTTCGGACAA
GTGAGATTTAACGAAAGTGGCGAACGGGTGAGTAACACGTGAGCAACCTGCCTTACACAGGGGGATAGCC

ATTGGAAACGGTGATTAATACCCCATAAGACCACAATGCCGCATGGTAAAAGGGTAAAAGGGATACCGGT
GTAAGATGGGCTCGCGTCTGATTAGCTAGTTGGTGGGGTAAAGGCCTACCAAGGCGACGATCAGTAGCCG
GTCTGAGAGGATGAACGGCCACATTGGAACTGAGACACGGTCCAAACTCCTACGGGAGGCAGCAGTGGGG
AATATTGCACAATGGGGGAAACCCTGATGCAGCGACGCCGCGTGAGCGAAGAAGGCTTTCGAGTCGTAAA
GCTCTGTCCTATGAGAAGATAATGACGGTATCATAGGAGGAAGCCCTGGCTAAATACGTGCCAGCAGCCG
CGGTAATACGTATGGGGCGAGCGTTGTCCGGAATTATTGGGCGTAAAGGGTACGTAGGCGGTTTTTTAAG
TCAGGTGTCAAAGCGTGGAGCTTAACTCCATTAAGCACTTGAAACTGAAAGACTTGAGTGAAGGAGAGGA
AAGTGGAATTCCTAGTGTAGCGGTGAAATGCGTAGATATTAGGAGGAATACCGGTGGCGAAGGCGACTTT
CTGGACTTTTACTGACGCTCAGGTACGAAAGCGTGGGGAGCAAGCAGGATTAGATACCCTGGTAGTCCAC
GCCGTAAACGATGAATGCTAGGTGTTGGGAGTCAAATCTCGGTGCCGAAGTTAACACATTAAGCATTCCG
CCTGGGGAGTACGGTGGCAACACTGAAACTCAAAGGAATTGACGGGGACCCGCACAAGCAGCGGAGCATG
TGGTTTAATTCGAAGCAACGCGAAGAACCTTACCAAGGCTTGACATATAGTTGAGTTATTGAGAAATTGA
TAAGTCCCTCGGGACAACTATACAGGTGGTGCATGGTTGTCGTCAGCTCGTGTCGTGAGATGTTGGGTTA
AGTCCCGCAACGAGCGCAACCCTTATTTTCAGTTACCAGCATTTAAGGTGGGGACTCTGAAGAGACTGCC
GATGACAAATCGGAGGAAGGTGGGGATGACGTCAAATCATCATGCCCTTTATGTCTTGGGCTACACACGT
GCTACAATGGTCGGTACAACGAGAAGCGAGATAGTGATGTTAAGCGAAACTCTAAAAGCCGATCTCAGTT
CGGATTGTAGGCTGCAACTCGCCTACATGAAGTCGGAGTTGCTAGTAATCGCGAATCAGAACGTCGCGGT
GAATGCGTTCCCGGGTCTTGTACACACCGCCCGTCACACCATGGGAGCTTGTAATACCCGAAGCCGTCGA
TCCAACCGCAAGGAGGAAGACGTCGAAGGCAGGACAAGTGACTGGGGTGAAGTCGTAACAAGGTA
>119352243|gb|EF120366.1|BVAB1|Uncultured Lachnospiraceae bacterium clone MZH0805-50 16S ribosomal RNA gene, partial sequence
GATGAACGCTGGCGGCGTGCTTAACACATGCAAGTCGAACGAAGTGCTACGACGGAAGTTTTCGGACGGA
AGATGTAGTTACTTAGTGGCGGACGGGTGAGTAACGCGTGGGGAACCTGCCCTGTACCGGGGGATAGCAG
CCGGAAACGGCTGATAATACCGCATAAGCGCACGAATGTCGCATGACATGGTGTGAAAAACTCCGGTGGT
ATAGGATGGACCCGCGTCTGATTAGCCGGTTGGTGGGGTAAAAGCCTACCAAAGCGAAGATCAGTAGCCG
AGTTGAGAGACTGACCGGCCACATTGGGACTGAGACACGGCCCAGACTCCTACGGGAGGCAGCAGTGGGG
AATATTGCACAATGGGCGAAAGCCTGATGCAGCGACGCCGCGTGAGCGAAGAAGTATTTCGGTATGTAAA
GCTCTATCAGAAGGGAAGAAAATGACGGTACCTTACTAAGAAGCTCCGGCTAAATACGTGCCAGCAGCCG
CGGTAATACGTATGGAGCAAGCGTTATCCGGATTTACTGGGTGTAAAGGGAGTGTAGGCGGCACTATAAG
TCTGATGTGAAAACCTAAGGCTTAACCATAGGATTGCATTGGAAACTGTAGAGCTGGAGTATCGGAGAGG
CAAGCGGAATTCCTGGTGTAGTGGTGAAATACGTAGATATCAGGAAGAACATCGGTGGCGAAGGCGGCTT
GCTGGATGATAACTGACGCTAAGGCTCGAAAGCGTGGGAAGCGAACAGGATTAGATACCCTGGTAGTCCA
CGCTGTAAACGATGAACACTAGGTGTTGGGAGGCTAAGCCTTTCAGTGCCGCAGCAAACGCAATAAGTGT
TCCACCTGGGGAGTACGTTCGCAAGAATGAAACTCAAAGGAATTGACGGGGACCCGCACAAGCGGTGGAG
CATGTGGTTTAATTCGAAGCAACGCGAAGAACCTTACCAAGTCTTGACATCCCTGTGACAGTATATGTAA
TGTATATTTTCTACGGAACACAGGAGACAGGTGGTGCATGGTTGTCGTCAGCTCGTGTCGTGAGATGTTG
GGTTAAGTCCCGCAACGAGCGCAACCCTTGTACTTAGTAGCCAGCATTAAGGTGGGCACTCTAAGTAGAC
TGCCGGGGTGAACCCGGAGGAAGGTGGGGATGACGTCAAATCATCATGCCCCTTATGACTTGGGCTACAC
ACGTGCTACAATGGCGTGAACAGAGGGAAGCGAAGGAGCGATCCGGAGCAAATCTCATAAAGCACGTCCC
AGTTCGGACTGCAGTCTGCAACTCGACTGCACGAAGCTGGAATCGCTAGTAATCGCAGATCATCATGCTG
CGGTGAATACGTTCCCGGGTCTTGTACACACCGCCCGTCACACCATGGGAGTCTGGAATGCCCGAAGTCA
GTGGCCAAACCGAGAGGATGGAGCTGCCGAAGGCAGGCCCGGTAACTGGGGTG
>66878775|gb|AY959069.1|BVAB1|Uncultured bacterium clone rRNA296 16S ribosomal RNA gene, partial sequence
TTCGCCCTTAGAGTTTGATCCTGGCTCAGGATGAACGCTGGCGGCGTGCTTAACACATGCAAGTCGAACG
AAGTGCTACGACGGAAGTTTTCGGACGGAAGATGTAGTTACTTAGTGGCGGACGGGTGAGTAACGCGTGG
GGAACCTGCCCTGTACCGGGGGATAGCAGCCGGAAACGGCTGATAATACCGCATAAGCGCACGAATGTCG
CATGACATGGTGTGAAAAACTCCGGTGGTATAGGATGGACCCGCGTCTGATTAGCCAGTTGGTGGGGTAA
AAGCCTACCAAAGCGAAGATCAGTAGCCGAGTTGAGAGACTGACCGGCCACATTGGGACTGAGACACGGC
CCAGACTCCTACGGGAGGCAGCAGTGGGGAATATTGCACAATGGGCGAAAGCCTGATGCAGCGACGCCGC
GTGAGCGAAGAAGTATTTCGGTATGTAAAGCTCTATCAGAAGGGAAGAAAATGACGGTACCTTACTAAGA
AGCTCCGGCTAAATACGTGCCAGCAGCCGCGGTAATACGTATGGAGCAAGCGTTATCCGGATTTACTGGG
TGTAAAGGGAGTGTAGGCGGCACTATAAGTCTGATGTGAAAACCTAAGGCTTAACCATAGGATTGCATTG
GAAACTGTAGAGCTGGAGTATCGGAGAGGCAAGCGGAATTCCTGGTGTAGTGGTGAAATACGTAGATATC

AGGAAGAACATCGGTGGCGAAGGCGGCTTGCTGGACGATAACTGACGCTAAGGCTCGAAAGCGTGGGAAG
CGAACAGGATTAGATACCCTGGTAGTCCACGCTGTAAACGATGAACACTAGGTGTTGGGAGGCTAAGCCT
TTCAGTGCCGCAGCAAACGCAATAAGTGTTCCACCTGGGGAGTACGTTCGCAAGAATGAAACTCAAAGGA
ATTGACGGGGACCCGCACAAGCGGTGGAGCATGTGGTTTAATTCGAAGCAACGCGAAGAACCTTACCAAG
TCTTGACATCCCTGTGACAGTATATGTAATGTATATTTTCTACGGAACACAGGAGACAGGTGGTGCATGG
TTGTCGTCAGCTCGTGTCGTGAGATGTTGGGTTAAGTCCCGCAACGAGCGCAACCCTTGTACTTAGTAGC
CAGCATTAAGGTGGGCACTCTAAGTAGACTGCCGGGGTGAACCCGGAGGAAGGTGGGGATGACGTCAAAT
CATCATGCCCCTTATGACTTGGGCTACACACGTGCTACAATGGCGTGAACAGAGGGAAGCGAAGGAGCGA
TCCGGAGCAAATCTCATAAAGCACGTCCCAGTTCGGACTGCAGTCTGCAACTCGACTGCACGAAGCTGGA
ATCGCTAGTAATCGCAGATCATCATGCTGCGGTGAATACGTTCCCGGGTCTTGTACACACCGCCCGTCAC
ACCATGGGAGTCTGGAATGCCCGAAGTCAGTGGCCAAACCGAAAGGATGGAGCTGCCGAAGGCAGGCCCG
GTAACTGGGGTGAAGTCGTAACAAGGTAGCCGTAAAG
>44307592|gb|AY471619.1|BVAB1|Uncultured bacterium clone 7200A-2c13Sm 16S ribosomal RNA gene, partial sequence
GATGAACGCTGGCGGCGTGCTTAACACATGCAAGTCGAACGAAGTGCTACGACGGAAGTTTTCGGACGGA
AGATGTAGTTACTTAGTGGCGGACGGGTGAGTAACGCGTGGGGAACCTGCCCTGTACCGGGGGATAGCAG
CCGGAAACGGCTGATAATACCGCATAAGCGCACAAATGTCGCATGACATGGTGTGAAAAACTCCGGTGGT
ATAGGATGGACCCGCGTCTGATTAGCCAGTTGGTGGGGTAAAAGCCTACCAAAGCGAAGATCAGTAGCCG
AGTTGAGAGACTGACCGGCCACATTGGGACTGAGACACGGCCCAGACTCCTACGGGAGGCAGCAGTGGGG
AATATTGCACAATGGGCGAAAGCCTGATGCAGCGACGCCGCGTGAGCGAAGAAGTATTTCGGTATGTAAA
GCTCTATCAGAAGGGAAGAAAATGACGGTACCTTACTAAGAAGCTCCGGCTAAATACGTGCCAGCAGCCG
CGGTAATACGTATGGAGCAAGCGTTATCCGGATTTACTGGGTGTAAAGGGAGTGTAGGCGGCACTATAAG
TCTGATGTGAAAACCTAAGGCTTAACCATAGGATTGCATTGGAAACTGTAGAGCTGGAGTATCGGAGAGG
CAAGCGGAATTCCTGGTGTAGTGGTGAAATACGTAGATATCAGGAAGAACATCGGTGGCGAAGGCGGCTT
GCTGGACGATAACTGACGCTAAGGCTCGAAAGCGTGGGAAGCGAACAGGATTAGATACCCTGGTAGTCCA
CGCTGTAAACGATGAACACTAGGTGTTGGGAGGCTAAGCCTTTCAGTGCCGCAGCAAACGCAATAAGTGT
TCCACCTGGGGAGTACGTTCGCAAGAATGAAACTCAAAGGAATTGACGGGGACCCGCACAAGCGGTGGAG
CATGTGGTTTAATTCGAAGCAACGCGAAGAACCTTACCAAGTCTTGACATCCCTGTGACAGTATATGTAA
TGTATATTTTCTACGGAACACAGGAGACAGGTGGTGCATGGTTGTCGTCAGCTCGTGTCGTGAGATGTTG
GGTTAAGTCCCGCAACGAGCGCAACCCTTGTACTTAGTAGCCAGCATTAAGGTGGGCACTCTAAGTAGAC
TGCCGGGGTGAACCCGGAGGAAGGTGGGGATGACGTCAAATCATCATGCCCCTTATGACTTGGGCTACAC
ACGTGCTACAATGGCGTGAACAGAGGGAAGCTAAGGAGCGATCCGGAGCAAAATCTCATAAAGCACGTCC
CAGTTCGGACTGCAGTCTGCAACTCGACTGCACGAAGCTGGAATCGCTAGTAATCGCAGATCATCATGCT
GCGGTGAATACGTTCCCGGGTCTTGTACACACCGCCCGTCACACCATGGGAGTCTGGAATGCCCGAAGTC
AGTGGCCAAACCGAAAGGATGGAGCTGCCGAAGGCAGGCCCGGTAACTGGGGTG
>66878803|gb|AY959097.1|BVAB1|Uncultured bacterium clone rRNA324 16S ribosomal RNA gene, partial sequence
TTCGCCCTTAGAGTTTGATCCTGGCTCAGGATGAACGCTGGCGGCGTGCTTAACACATGCAAGTCGAACG
AAGTGCTACGACGGAAGTTTTCGGACGGAAGATGTAGTTACTTAGTGGCGGACGGGTGAGTAACGCGTGG
GGAACCTGCCCTGTACCGGGGGATAGCAGCCGGAAACGGCTGATAATACCGCATAAGCGCACGAATGTCG
CATGACATGGTGTGAAAAACTCCGGTGGTATAGGATGGACCCGCGTCTGATTAGCCGGTTGGTGGGGTAA
AAGCCTACCAAAGCGAAGATCAGTAGCCGAGTTGAGAGACTGACCGGCCACATTGGGACTGAGACACGGC
CCAGACTCCTACGGGAGGCAGCAGTGGGGAATATTGCACAATGGGCGAAAGCCTGATGCAGCGACGCCGC
GTGAGCGAAGAAGTATTTCGGTATGTAAAGCTCTATCAGAAGGGAAGAAAATGACGGTACCTTACTAAGA
AGCTCCGGCTAAATACGTGCCAGCAGCCGCGGTAATACGTATGGAGCAAGCGTTATCCGGATTTACTGGG
TGTAAAGGGAGTGTAGGCGGCACTATAAGTCTGATGTGAAAACCTAAGGCTTAACCATAGGATTGCATTG
GAAACTGTAGAGCTGGAGTATCGGAGAGGTAAGCGGAATTCCTGGTGTAGTGGTGAAATACGTAGATATC
AGGAAGAACATCGGTGGCGAAGGCGGCTTGCTGGACGATAACTGACGCTAAGGCTCGAAAGCGTGGGAAG
CGAACAGGATTAGATACCCTGGTAGTCCACGCTGTAAACGATGAACACTAGGTGTTGGGAGGCTAAGCCT
TTCAGTGCCGCAGCAAACGCAATAAGTGTTCCACCTGGGGAGTACGTTCGCAAGAATGAAACTCAAAGGA
ATTGACGGGGACCCGCACAAGCGGTGGAGCATGTGGTTTAATTCGAAGCAACGCGAAGAACCTTACCAAG
TCTTGACATCCCTGTGACAGTATATGTAATGTATATTTTCTACGGAACACAGGAGACAGGTGGTGCATGG
TTGTCGTCAGCTCGTGTCGTGAGATGTTGGGTTAAGTCCCGCAACGAGCGCAACCCTTGTACTTAGTAGC
CAGCATTAAGGTGGGCACTCTAAGTAGACTGCCGGGGTGAACCCGGAGGAAGGTGGGGACGACGTCAAAT

CATCATGCCCCTTATGGCTTGGGCTACACACGTGCTACAATGGCGTGAACAGAGGGAAGCGAAGGAGCGA
TCCGGAGCAAATCTCATAAAGCACGTCCCAGTTCGGACTGCAGTCTGCAACTCGACTGCACGAAGCTGGA
ATCGCTAGTAATCGCAGATCATCATGCTGCGGTGAATACGTTCCCGGGTCTTGTACACACCGCCCGTCAC
ACCATGGGAGTCTGGAATGCCCGAAGTCAGTGGCCAAACCGAAAGGATGGAGCTGCCGAAGGCAGGCCCG
GTAACTGGGTGAA
>52222196|gb|AY724739.1|BVAB1|Uncultured bacterium clone 123-f 57 16S ribosomal RNA gene, partial sequence
TGGGGAATATTGCACAATGGGCGAAAGCCTGATGCAGCGACGCCGCGTGAGCGAAGAAGTATTTCGGTAT
GTAAAGCTCTATCAGAAGGGAAGAAAATGACGGTACCTTACTAAGAAGCTCCGGCTAAATACGTGCCAGC
AGCCGCGGTAATACGTATGGAGCAAGCGTTATCCGGATTTACTGGGTGTAAAGGGAGTGTAGGCGGCACT
ATAAGTCTGATGTGAAAACCTAAGGCTTAACCATAGGATTGCATTGGAAACTGTAGAGCTGGAGTATCGG
AGAGGCAAGCGGAATTCCTGGTGTAGTGGTGAAATACGTAGATATCAGGAAGAACATCGGTGGCGAAGGC
GGCTTGCTGGACGATAACTGACGCTAAGGCTCGAAAGCGTGGGAAGCGAACAGGATTAGATACCCTGGTA
GTCCACGCTGTAAACGATGAACACTAGGTGTTGGGAGGCTAAGCCTTTCAGTGCCGCAGCAAACGCAATA
AGTGTTCCACCTGGGGAGTACGTTCGCAAGAATGAAACTCAAAGGAATTGACGGGGACCCGCACAAGCGG
TGGAGCATGTGGTTTAATTCGAAGCAACGCGAAGAACCTTACCAAGTCTTGACATCCCTGTGACAGTATA
TGTAATGTATATTTTCTACGGAACACAGGAGACAGGTGGTGCATGGTTGTCGTCAGCTCGTGTCGTGAGA
TGTTGGGTTAAGTCCCGCAACGAGCGCAACCCTTGTACTTAGTAGCCAGCATTAAGGTGGGCACTCTAAG
TAGACTGCCGGGGTGAACCCGGAGGAAGGTGGGGATGACGTCAAATCATCATGCCCCTTATGACTTGGGC
TACACACGTGCTACAATGGCGTGAACAGAGGGAAGCGAAGGAGCGATCCGGAGCAAATCTCATAAAGCAC
GTCCCAGTTCGGACTGCAGTCTGCAACTCGACTGCACGAAGCTGGAATCGCTAGTAATCGCAGATCATCA
TGCTGCGGTGAATACGTTCCCGGGTCT
>66878839|gb|AY959133.1|P.timonensis|Uncultured bacterium clone rRNA360 16S ribosomal RNA gene, partial sequence
CGCCCTTAGAGTTTGATCCTGGCTCAGGATGAACGCTAGCTACAGGCTTAACACATGCAAGTCGCAGGGT
AACATGAGGAAAGCTTGCTTTCCTTGATGACGACTGGCGCACGGGTGAGTAACGCGTATCCAACCTTCCC
ATAACTACGGGATAACCCGTTGAAAGACGGCCTAATACCGTATGATATCGTTTGCTGACATCAAATAACG
ATTAAAGGTTTAGCGGTTATGGATGGGGATGCGTCTGATTAGCTTGTTGGCGGGGTAACGGCCCACCAAG
GCGACGATCAGTAGGGGTTCTGAGAGGAAGGTCCCCCACATTGGAACTGAGACACGGTCCAAACTCCTAC
GGGAGGCAGCAGTGAGGAATATTGGTCAATGGGCGAGAGCCTGAACCAGCCAAGTAGCGTGCAGGAAGAC
GGCCCTATGGGTTGTAAACTGCTTTTATGTGGGGATAAAGTGCGTGACGTGTCATGCATTGCAGGTACCA
CATGAATAAGGACCGGCTAATTCCGTGCCAGCAGCCGCGGTAATACGGAAGGTCCGGGCGTTATCCGGAT
TTATTGGGTTTAAAGGGAGCGTAGGCTGTCTATTAAGCGTGTTGTGAAATTTACCGGCTCAACCGGTGGC
TTGCAGCGCGAACTGGTCGACTTGAGTATGCAGGAAGTAGGCGGAATTCATGGTGTAGCGGTGAAATGCT
TAGATATCATGACGAACTCCGATTGCGCAGGCAGCTTACTGTAGCATAACTGACGCTGATGCTCGAAAGT
GCGGGTATCAAACAGGATTAGATACCCTGGTAGTCCGCACGGTAAACGATGGATGCTCGCTATTCGTCCT
ATTTGGATGAGTGGCCAAGTGAAAACATTAAGCATCCCACCTGGGGAGTACGCCGGCAACGGTGAAACTC
AAAGGAATTGACGGGGGCCCGCACAAGCGGAGGAACATGTGGTTTAATTCGATGATACGCGAGGAACCTT
ACCCGGGCTTGAACTGCCAGCGAACGATACAGAGATGTTGAGGCCCTTCGGGGCGCTGGTGGAGGTGCTG
CATGGTTGTCGTCAGCTCGTGCCGTGAGGTGTCGGCTTAAGTGCCATAACGAGCGCAACCCTTTTCTTTA
GTTGCCATCAGGTAATGCTGGGCACTCTATGGATACTGCCACCGTAAGGTGTGAGGAAGGTGGGGATGAC
GTCAAATCAGCACGGCCCTTACGTCCGGGGCTACACACGTGTTACAATGGGGCATACAGAGTGTTGGCTT
AACGCAAGTTTGGTCTAATCTTCAAAGTGTCTCCCAGTTCGGATTGGGGTCTGCAACCCGACCCCATGAA
GCTGGATTCGCTAGTAATCGCGCATCAGCCATGGCGCGGTGAATACGTTCCCGGGCCTTGTACACACCGC
CCGTCAAGCCATGAAAGCTGGGGGTGCCTGAAGTCCGTAACCGTTAAGGAGCGGCCTAGGGCAAAACTGG
TGATTGGGGCTAAGTCGTAACAAGGTAACCGTAAAGGGC
>302129314|dbj|AB547676.1|P.buccalis|Prevotella buccalis gene for 16S ribosomal RNA, partial sequence, strain: JCM 12246
AGAGTTTGATCCTGGCTCAGGATGAACGCTAGCTACAGGCTTAACACATGCAAGTCGCAGGGTAACGTGA
GGGAAGCTTGCTTCCCTTGACGACGACTGGCGCACGGGTGAGTAACGCGTATCCAACCTTCCCATGACCA
CGGGATAACCCGTTGAAAGACGGACTAATACCGTATGACGTCGTTTGCTGACATCAAATAACGATTAAAG
GTTTAGCGGTGATGGATGGGGATGCGTCTGATTAGCTTGTTGGCGGGGTAACGGCCCACCAAGGCGACGA
TCAGTAGGGGTTCTGAGAGGAAGGTCCCCCACATTGGAACTGAGACACGGTCCAAACTCCTACGGGAGGC
AGCAGTGAGGAATATTGGTCAATGGGCGAGAGCCTGAACCAGCCAAGTAGCGTGCAGGATGACGGCCCTA

TGGGTTGTAAACTGCTTTTATGCGGGGATAAAGTGCGCGACGTGTCGTGCATTGCAGGTACCGCATGAAT
AAGGACCGGCTAATTCCGTGCCAGCAGCCGCGGTAATACGGAAGGTCCGGGCGTTATCCGGATTTATTGG
GTTTAAAGGGAGCGTAGGCCGCCAGGTAAGCGTGTTGTGAAATGTACCGGCTCAACCGGTGAATTGCAGC
GCGAACTGTCTGGCTTGAGTGCACGGTAAGCAGGCGGAATTCATGGTGTAGCGGTGAAATGCTTAGATAT
CATGAAGAACTCCGATTGCGAAGGCAGCTTGCTGCAGTGCGACTGACGCTGATGCTCGAAGGTGCGGGTA
TCAAACAGGATTAGATACCCTGGTAGTCCGCACGGTAAACGATGGATGCCCGCTGTCCGCCCTTTTGTGG
CGGGTGGCCAAGCGAAAGCGTTAAGCATCCCACCTGGGGAGTACGCCGGCAACGGTGAAACTCAAAGGAA
TTGACGGGGGCCCGCACAAGCGGAGGAACATGTGGTTTAATTCGATGATACGCGAGGAACCTTACCCGGG
CTTGAACTGCCAGTGAACGATACAGAGATGTTGAGGCCCTTCGGGGCGCTGGTGGAGGTGCTGCATGGTT
GTCGTCAGCTCGTGCCGTGAGGTGTCGGCTTAAGTGCCATAACGAGCGCAACCCCTTTTTTCAGTTGCCA
TCAGGTAATGCTGGGCACTCTGGAGATACTGCCACCGCAAGGTGTGAGGAAGGTGGGGATGACGTCAAAT
CAGCACGGCCCTTACGTCCGGGGCTACACACGTGTTACAATGGGGCATACAGAGTGTTGGCTTAACGCAA
GTTTGGTCTAATCTTCAAAGTGTCTCCCAGTTCGGACTGGGGTCTGCAACCCGACCCCACGAAGCTGGAT
TCGCTAGTAATCGCGCATCAGCCATGGCGCGGTGAATACGTTCCCGGGCCTTGTACACACCGCCCGTCAA
GCCATGAAAGCCGGGGGTGCCTGAAGTCCGTAACCGTCAAGGAGCGGCCTAGGGCAAAACTGGTGATTGG
GGCTAAGTCGTAACAAGGTAACC
>302129308|dbj|AB547670.1|P.amnii|Prevotella amnii gene for 16S ribosomal RNA, partial sequence, strain: JCM 14753
AGAGTTTGATCCTGGCTCAGGATGAACGCTAGCTATAGGCTTAACACATGCAAGTCGAGGGGCAGCATAT
AGATTGCTTGCAATTTATGATGGCGACCGGCGCACGGGTGAGTAACGCGTATCCAACCTACCCATTACTA
GGGAATAACCCAGCGAAAGTTGGCCTAATGCCCTATGTAGTCGTTTGATCGCCTGAGATTTCGACGAAAG
ATTTATCGGTATTGGATGGGGATGCGTCTGATTAGCTTGTTGGCGGGGTAAAGGCCCACCAAGGCGACGA
TCAGTAGGGGTTCTGAGAGGAAGGTCCCCCACATTGGAACTGAGACACGGTCCAAACTCCTACGGGAGGC
AGCAGTGAGGAATATTGGTCAATGGGCGAGAGCCTGAACCAGCCAAGTAGCGTGCAGGATGACGGCCCTA
TGGGTTGTAAACTGCTTTTATATGGGAATAAAGTGAGGGACGTGTCCCTTATTGCATGTACCATATGAAT
AAGGACCGGCTAATTCCGTGCCAGCAGCCGCGGTAATACGGAAGGTCCAGGCGTTATCCGGATTTATTGG
GTTTAAAGGGAGCGTAGGCTGTTTGTTAAGCGTGTTGTGAAATGTAAGAGCTCAACTTTTAGATTGCAGC
GCGAACTGGCAGACTTGAGTGCGCACAACGTAGGCGGAATTCATGGTGTAGCGGTGAAATGCTTAGATAT
CATGACGAACTCCGATTGCGAAGGCAGCTTACGGGAGCGCAACTGACGCTAAAGCTCGAAGGTGCGGGTA
TCGAACAGGATTAGATACCCTGGTAGTCCGCACAGTAAACGATGGATGCCCGCTGTTAGCACCTAGTGTT
AGCGGCTAAGCGAAAGCATTAAGCATCCCACCTGGGGAGTACGCCGGCAACGGTGAAACTCAAAGGAATT
GACGGGGGCCCGCACAAGCGGAGGAACATGTGGTTTAATTCGATGATACGCGAGGAACCTTACCCGGGCT
TGAATTGCAGATGTTTATATCAGAGATGATATATTCCCTTCGGGGCATTTGTGAAGGTGCTGCATGGTTG
TCGTCAGCTCGTGCCGTGAGGTGTCGGCTTAAGTGCCATAACGAGCGCAACCCCTTTTTTAGTTGCCAT
CAGGTAGTGCTGGGCACTCTAGAGATACTGCCACCGTAAGGTGTGAGGAAGGTGGGGATGACGTCAAATC
AGCACGGCCCTTACGTCCGGGGCTACACACGTGTTACAATGGGTGGTACAGAGAGTTGGTTGTACGCAAG
TGCGATCTAATCCTAAAAACCATTCTCAGTTCGGACTGGGGTCTGCAACCCGACCCCACGAAGCTGGATT
CGCTAGTAATCGCGCATCAGCCATGGCGCGGTGAATACGTTCCCGGGCCTTGTACACACCGCCCGTCAAG
CCATGAAAGCCGGGGGTGCCTGAAGTTCGTGACCGTAAGGATCGACCTAGGGCAAAACTGGTAATTGGGG
CTAAGTCGTAACAAGGTAACC
>110169812|gb|DQ666093.1|P.amnii|Uncultured bacterium clone Y3 16S ribosomal RNA gene, partial sequence
AGAGTTTGATCCTGGCTCAGGATGAACGCTAGCTATAGGCTTAACACATGCAAGTCGAGGGGCAGCATAT
AGATTGCTTGCAATTTATGATGGCGACCGGCGCACGGGTGAGTAACGCGTATCCAACCTACCCATTACTA
GGGAATAACCCAGCGAAAGTTGGCCTAATGCCCTATGTAGTCGTTTGATCGCCTGAGATTTCGACGAAAG
ATTTATCGGTATTGGATGGGGATGCGTCTGATTAGCTTGTTGGCGGGGTAAAGGCCCACCAAGGCAACGA
TCAGTAGGGGTTCTGAGAGGAAGGTCCCCCACATTGGAACTGAGACACGGTCCAAACTCCTACGGGAGGC
AGCAGTGAGGAATATTGGTCAATGGGCGAGAGCCTGAACCAGCCAAGTAGCGTGCAGGATGACGGCCCTA
TGGGTTGTAAACTGCTTTTATATGGGAATAAAGTGAGGGACGTGTCCCTTATTGCATGTACCATACGAAT
AAGGACCGGCTAATTCCGTGCCAGCAGCCGCGGTAATACGGAAGGTCCAGGCGTTATCCGGATTTATTGG
GTTTAAAGGGAGCGTAGGCTGTTTGTTAAGCGTGTTGTGAAATGTAGGAGCTCAACTTTTAGATTGCAGC
GCGAACTGGCAGACTTGAGTGCGCACAACGTAGGCGGAATTCATGGTGTAGCGGTGAAATGCTTAGATAT
CATGACGAACTCCGATTGCGAAGGCAGCTTACGGGAGCGCAACTGACGCTAAAGCTCGAAGGTGCGGGTA
TCGAACAGGATTAGATACCCTGGTAGTCCGCACAGTAAACGATGGATGCCCGCTGTTAGCACCTAGTGTT

AGCGGCTAAGCGAAAGCATTAAGCATCCCACCTGGGGAGTACGCCGGCAACGGTGAAACTCAAAGGAATT
GACGGGGGCCCGCACAAGCGGAGGAACATGTGGTTTAATTCGATGATACGCGAGGAACCTTACCCGGGCT
TGAATTGCAGATGTTTATGTCAGAGATGATGTATTCCTTTCGGGGCATTTGTGAAGGTGCTGCATGGTTG
TCGTCAGCTCGTGCCGTGAGGTGTCGGCTTAAGTGCCATAACGAGCGCAACCCCTTTTTTTAGTTGCCAT
CAGGTAGTGCTGGGCACTCTAGAGATACTGCCACCGTAAGGTGTGAGGAAGGTGGGGATGACGTCAAATC
AGCACGGCCCTTACGTCCGGGGCTACACACGTGTTACAATGGGTGGTACAGAGAGTTGGTTGTACGCAAG
TGCAATCTAATCCTAAAAACCATTCTCAGTTCGGACTGGGGTCTGCAACCCGACCCCACGAAGCTGGATT
CGCTAGTAATCGCGCATCAGCCATGGCGCGGTGAATACGTTCCCGGGCCTTGTACACACCGCCCGTCAAG
CCATGAAAGCCGGGGGTGCCTGAAGTTCGTGACCGTAAGGATCGACCTAGGGCAAAACTGGTAATTGGGG
CTAAGTCGTAACAAGGTAACC
>119352237|gb|EF120360.1|Atopobium|Uncultured Atopobium sp. clone MZH0805-07 16S ribosomal RNA gene, partial sequence
GATGAACGCTGGCGGCGCGCCTAACACATGCAAGTCGAACGGTTAAAGCATCTTCGGATGTGTATAAAGT
GGCGAACGGCTGAGTAACACGTGGGCAACCTGCCCTTTGCACTGGGATAGCCTCGGGAAACCGAGGTTAA
TACCGGATACTCCATATCCATCGCATGATAGATATGGGAAAGCTCCGGCGGCAAAGGATGGGCCCGCGGC
CTGTTAGCTAGTTGGTGGGGTAGTGGCCTACCAAGGCAATGATGGGTAGCCGGGTTGAGAGACCGACCGG
CCAGATTGGGACTGAGACACGGCCCAGACTCCTACGGGAGGCAGCAGTGGGGAATCTTGCACAATGGGCG
AAAGCCTGATGCAGCGACGCCGCGTGCGGGATGAAGGCCTTCGGGTTGTAAACCGCTTTCAGCAGGGACG
AGGCCGCAAGGTGACGGTACCTGCAGAAGAAGCCCCGGCTAACTACGTGCCTGCAGCCGCGGTAATACGT
AGGGGGCAAGCGTTATCCGGATTCATTGGGCGTAAAGCGCGCGTAGGCGGTCTGTTAGGTCAGGAGTCAA
ATCTGGGGGCTCAACCCCTATCCGCTCCTGATACCGGCAGGCTTGAGTCTGGTATGGGAAGGTGGAATTC
CAAGTGTAGCGGTGAATTGCGCAGATATTTGGAAGAACACCAGTGGCGAAGGCGGCCTTCTGGGCCATGA
CTGACGCTGAGGCGCGAAAGCTAGGGGAGCGAACAGGATTAGATACCCTGGTAGTCCTAGCTGTAAACGA
TGGACACTAGGTGTGGGGAGATTATACTTTCCGTGCCGCAGCTAACGCATTAAGTGTCCCGCCTGGGGAG
TACGGTCGCAAGACTAAAACTCAAAGGAATTGACGGGGGCCCGCACAAGCAGCGGAGCATGTGGCTTAAT
TCGAAGCAACGCGAAGAACCTTACCAGGGCTTGACATTTAGGTGAAGCAGTGGAAACACTGTGGCCGAAA
GGAGCCTAAACAGGTGGTGCATGGCTGTCGTCAGCTCGTGTCGTGAGATGTTGGGTTAAGTCCCGCAACG
AGCGCAACCCTCGTCGCATGTTGCCAGCGGTTCGGCCGGGCACCCATGCGAGACCGCCGGCGTTAAGCCG
GAGGAAGGTGGGGACGACGTCAAGTCATCATGCCCCTTATGTCCTGGGCTGCACACGTGCTACAATGGCC
GGCACAGAGGGCTGCTACTGCGCGAGCAGGAGCGAATCCCTAAAGCCGGTCCCAGTTCGGATTGGAGGCT
GCAACTCGCCTCCATGAAGTCGGAGTTGCTAGTAATCGCGGATCAGCACGCCGCGGTGAATGCGTTCCCG
GGCCTTGTACACACCGCCCGTCACACCACCCGAGTCGTCTGCACCCGAAGTCGTCGGCCTAACCCGCAAG
GGAGGGAGACGCCGAAGGTGTGGAGGGTAAGGGGGGTG
>66878878|gb|AY959172.1|Atopobium|Uncultured bacterium clone rRNA399 16S ribosomal RNA gene, partial sequence
CGCCCTTAGAGTTTGATCCTGGCTCAGGATGAACGCTGGCGGCGCGCCTAACACATGCAAGTCGAACGGT
TAAAGCATCTTCGGATGTGTATAAAGTGGCGAACGGCTGAGTAACACGTGGGCAACCTGCCCTTTGCACT
GGGATAGCCTCGGGAAACCGAGGTTAATACCGGATACTCCATATCCATCGCATGATAGATATGGGAAAGC
TCCGGCGGCAAAGGATGGGCCCGCGGCCTGTTAGCTAGTTGGTGGGGTAGTGGCCTACCAAGGCAATGAT
GGGTAGCCGAGTTGAGAGACCGACCGGCCAGATTGGGACTGAGACACGGCCCAGACTCCTACGGGAGGCA
GCAGTGGGGAATCTTGCACAATGGGCGAAAGCCTGATGCAGCGACGCCGCGTGCGGGATGAAGGCCTTCG
GGTTGTAAACCGCTTTCAGCAGGGACGAGGCCGCAAGGTGACGGTACCTGCAGAAGAAGCCCCGGCTAAC
TACGTGCCAGCAGCCGCGGTAATACGTAGGGGGCAAGCGTTATCCGGATTCATTGGGCGTAAAGCGCGCG
TAGGCGGTCTGTTAGGTCAGGAGTCAAATCTGGGGGCTCAACCCCTATCCGCTCCTGATACCGGCAGGCT
TGAGTCTGGTATGGGAAGGTGGAATTCCAAGTGTAGCGGTGAAATGCGCAGATATTTGGAAGAACACCAG
TGGCGAAGGCGGCCTTCTGGGCCATGACTGACGCTGAGGCGCGAAAGCTAGGGGAGCGAACAGGATTAGA
TACCCTGGTAGTCCTAGCTGTAAACGATGGACACTAGGTGTGGGGAGATTATACTTTCCGTGCCGCAGCT
AACGCATTAAGTGTCCCGCCTGGGGAGTACGGTCGCAAGACTAAAACTCAAAGGAATTGACGGGGGCCCG
CACAAGCAGCGGAGCATGTGGCTTAATTCGAAGCAACGCGAAGAACCTTACCAGGGCTTGACATTTAGGT
GAAGCAGTGGAAACACTGTGGCCGAAAGGAGCCTAAACAGGTGGTGCATGGCTGTCGTCAGCTCGTGTCG
TGAGATGTTGGGTTAAGTCCCGCAACGAGCGCAACCCTCGTCGCATGTTGCCAGCGGTTCGGCCGGGCAC
CCATGCGAGACCGCCGGCGTTAAGCCGGAGGAAGGTGGGGACGACGTCAAGTCATCATGCCCCTTATGTC
CTGGGCTGCACACGTGCTACAATGGCCGGCACAGAGGGCTGCTACTGCGCGAGCAGGAGCGAATCCCTAA
AGCCGGTCCCAGTTCGGATTGGAGGCTGCAACTCGCCTCCATGAAGTCGGAGTTGCTAGTAATCGCGGAT

CAGCACGCCGCGGTGAATGCGTTCCCGGGCCTTGTACACACCGCCCGTCACACCACCCGAGTCGTCTGCA
CCCGAAGTCGTCGGCCTAACCCGCGAGGGAGGGAGACGCCGAAGGTGTGGAGGGTAAGGGGGGTGAAGTC
GTAACAAGGTAGCCGTAAAGGGC
>12240234|gb|AF325325.1|Atopobium|Atopobium vaginae 16S ribosomal RNA gene, partial sequence
GATGAACGCTGGCGGCGCGCCTAACACATGCAAGTCGAACGGTTAAAGCATCTTCGGATGTGTATAAAGT
GGCGAACGGCTGAGTAACACGTGGGCAACCTGCCCTTTGCACTGGGATAGCCTCGGGAAACCGAGGTTAA
TACCGGATACTCCATATTTGTCGCATGGCGAATATGGGAAAGCTCCGGCGGCAAAGGATGGGCCCGCGGC
CTGTTAGCTAGTTGGTGGGGTAGTGGCCTACCAAGGCAATGATGGGTAGCCGGGTTGAGAGACCGACCGG
CCAGATTGGGACTGAGACACGGCCCAGACTCCTACGGGAGGCAGCAGTGGGGAATCTTGCACAATGGGCG
AAAGCCTGATGCAGCGACGCCGCGTGCGGGATGAAGGCCTTCGGGTTGTAAACCGCTTTCAGCAGGGACG
AGGCCGCAAGGTGACGGTACCTGCAGAAGAAGCCCCGGCTAACTACGTGCCAGCAGCCGCGGTAATACGT
AGGGGGCAAGCGTTATCCGGATTCATTGGGCGTAAAGCGCGCGTAGGCGGTCTGTTAGGTCAGGAGTTAA
ATCTGGGGGCTCAACCCCTATCCGCTCCTGATACCGGCAGGCTTGAGTCTGGTAGGGGAAGATGGAATTC
CAAGTGTAGCGGTGAAATGCGCAGATATTTGGAAGAACACCGGTGGCGAAGGCGGTCTTCTGGGCCATGA
CTGACGCTGAGGCGCGAAAGCTAGGGGAGCGAACAGGATTAGATACCCTGGTAGTCCTAGCTGTAAACGA
TGGACACTAGGTGTGGGGAGATTATACTTTCCGTGCCGCAGCTAACGCATTAAGTGTCCCGCCTGGGGAG
TACGGTCGCAAGACTAAAACTCAAAGGAATTGACGGGGGCCCGCACAAGCAGCGGAGCATGTGGCTTAAT
TCGAAGCAACGCGAAGAACCTTACCAGGGCTTGACATTTAGGTGAAGCAGTGGAAACACTGTGGCCGAAA
GGAGCCTAAACAGGTGGTGCATGGCTGTCGTCAGCTCGTGTCGTGAGATGTTGGGTTAAGTCCCGCAACG
AGCGCAACCCTTGTCGCATGTTGCCAGCGGTTCGGCCGGGCACCCATGCGAGACCGCCGGCGTTAAGCCG
GAGGAAGGTGGGGACGACGTCAAGTCATCATGCCCCTTATGTCCTGGGCTGCACACGTGCTACAATGGCC
GGCACAGAGGGCTGCTACTGCGCGAGCAGAAGCGAATCCCTAAAGCCGGTCCCAGTTCGGATTGGAGGCT
GCAACTCGCCTCCATGAAGTCGGAGTTGCTAGTAATCGCGGATCAGCACGCCGCGGTGAATGCGTTCCCG
GGCCTTGTACACACCGCCCGTCACACCACCCGAGTCGTCTGCACCCGAAGTCGTCGGCCTAACCCGCAAG
GGAGGGAGGCGCCGAAGGTGTGGAGGGTAAGGGGGGT
>265679041|ref|NR_029349.1|Atopobium|Atopobium vaginae DSM 15829 strain 961*00022/98 16S ribosomal RNA, partial sequence
TCAGGATGAACGCTGGCGGCGCGCCTAACACATGCAAGTCGAACGGTTAAAGCATCTTCGGATGTGTATA
AAGTGGCGAACGGCTGAGTAACACGTGGGCAACCTGCCCTTTGCACTGGGATAGCCTCGGGAAACCGAGG
TTAATACCGGATACTCCATATTTNTCGCATGGCGAATATGGGAAAGCTCCGGCGGCAAAGGATGGGCCCG
CGGCCTGTTAGCTAGTTGGTGGGGTAGTGGCCTACCAAGGCAATGATGGGTAGCCGGGTTGAGAGACCGA
CCGGCCAGATTGGGACTGAGACACGGCCCAGACTCCTACGGGAGGCAGCAGTGGGGAATCTTGCACAATG
GGCGAAAGCCTGATGCAGCGACGCCGCGTGCGGGATGAAGGCCTTCGGGTTGTAAACCGCTTTCAGCAGG
GACNAGGCCGCAAGGTGACGGTACCTGCANAAGAAGCCCCGGCTAACTACGTGCCAGCAGCCGCGGTAAT
ACGTAGGGGGCAAGCGTTATCCGGATTCATTGGGCGTAAAGCGCGCGTAGGCGGTCTGTTAGGTCAGGAG
TTAAATCTGGGGGCTCAACCCCTATCCGCTCCTGATACCGGCAGGCTTGAGTCTGGTAGGGGAAGATGGA
ATTCCAAGTGTAGCGGTGAAATGCGCAGATATTTGGAAGAACACCGGTGGCGAAGGCGGTCTTCTGGGCC
ATGACTGACGCTGAGGCGCGAAAGCTAGGGGAGCGAACAGGATTAGATACCCTGGTAGTCCTANCTGTAA
ACGATGGACACTAGGTGTGGGGAGATTATACTTTCCGTGCCGCAGCTAACGCATTAAGTGTCCCGCCTGG
GGAGTACGGTCGCAAGACTAAAACTCAAAGGAATTGACGGGGGCCCGCACAAGCAGCGGAGCATGTGGCT
TAATTCGAAGCAACGCGAAGAACCTTACCAGGGCTTGACATTTAGGTGAAGCAGTGGAAACACTGTGGCC
GAAAGGAGCCTAAACAGGTGGTGCATGGCTGTCGTCAGCTCGTGTCGTGAGATGTTGGGTTAAGTCCCGC
AACGAGCGCAACCCTTGTCGCATGTTGCCAGCGGTTCGGCCGGGCACCCATGCGAGACCGCCGGCGTTAA
GCCGGAGGAAGGTGGGGACGACGTCAAGTCATCATGCCCCTTATGTCCTGGGCTGCACACGTGCTACAAT
GGCCGGCACAGAGGGCTGCTACTGCGCGAGCAGAAGCGAATCCCTAAAGCCGGTCCCAGTTCGGATTGGA
GGCTGCAACTCGCCTCCATGAAGTCGGAGTTGCTAGTAATCGCGGATCAGCACGCCGCGGTGAATGCGTT
CCCGGGCCTTGTACACACCGA
>66878497|gb|AY958791.1|L.crispatus|Uncultured bacterium clone rRNA018 16S ribosomal RNA gene, partial sequence
ACGAACGCTGGCGGCGTGCCTAATACATGCAAGTCGAGCGAGCGGAACTAACAGATTTACCTCGGTAATG
ACGTTAGGAAAGCGAGCGGCGGATGGGTGAGTAACACGTGGGGAACCTGCCCCATAGTCTGGGATACCAC
TTGGAAACAGGTGCTAATACCGGATAAGAAAGCAGATCGCATGATCAGCTTTTAAAAGGCGGCGTAAGCT
GTCGCTATGGGATGGCCCCGCGGTGCATTAGCTAGTTGGTAAGGTAAAGGCTTACCAAGGCGATGATGCA

TAGCCGAGTTGAGAGACTGATCGGCCACATTGGGACCGAGACACGGCCCAAACTCCTACGGGAGGCAGCA
GTAGGGAATCTTCCACAATGGACGCAAGTCTGGTGGAGCAACGCCGCGTGAGTGAAGAAGGTTTTCGGAT
CGTAAAGCTCTGTTGTTGGTGAAGAAGGATAGAGGTAGTAACTGGCCTTTATTTGACGGTAATCAACCAG
AAAGTCACGGCTAACTACGTGCCAGCAGCCGCGGTAATACGTAGGTGGCAAGCGTTGTCCGGATTTATTG
GGCGTAAAGCGAGCGCAGGCGGAAGAATAAGTCTGATGTGAAAGCCCTCGGCTTAACCGAGGAACTGCAT
CGGAAACTGTTTTTCTTGAGTGCAGAAGAGGAGAGTGGAACTCCATGTGTAGCGGTGGAATGCGTAGATA
TATGGAAGAACACCAGTGGCGAAGGCGGCTCTCTGGTCTGCAACTGACGCTGAGGCTCGAAAGCATGGGT
AGCGAACAAGATTAGATACCCTGGTAGTCCATGCCGTAAACGATGAGTGCTAAGTGTTGGGAGGTTTCCG
CCTCTCAGTGCTGCAGCTAACGCATTAAGCACTCCGCCTGGGGAGTACGACCGCAAGGTTGAAACTCAAA
GGAATTGACGGGGGCCCGCACAAGCGGTGGAGCATGTGGTTTAATTCGAAGCAACGCGAAGAACCTTACC
AGGTCTTGACATCTAGTGCCATTTGTAGAGATACAAAGTTCCCTTCGGGGACGCTAAGACAGGTGGTGCA
TGGCTGTCGTCAGCTCGTGTCGTGAGATGTTGGGTTAAGTCCCGCAACGAGCGCAACCCTTGTTATTAGT
TGCCAGCATTAAGTTGGGCACTCTAATGAGACTGCCGGTGACAAACCGGAGGAAGGTGGGGATGACGTCA
AGTCATCATGCCCCTTATGACCTGGGCTACACACGTGCTACAATGGGCAGTACAACGAGAAGCGAGCCTG
CGAAGGCAAGCGAATCTCTGAAAGCTGTTCTCAGTTCGGACTGCAGTCTGCAACTCGACTGCACGAAGCT
GGAATCGCTAGTAATCGCGGATCAGCACGCCGCGGTGAATACGTTCCCGGGCCTTGTACACACCGCCCGT
CACACCATGGGAGTCTGCAATGCCCAAAGCCGGTGGCCTAACCTTCGGGAAGGAGCCGTCTAAGGCAGGG
CAGATGACTGGGGTGAAGTCGTAACAAGGTAGCCGAAGGG
>66878635|gb|AY958929.1|L.crispatus|Uncultured bacterium clone rRNA156
16S ribosomal RNA gene, partial sequence
GCCCTTAGAGTTTGATCCTGGCTCAGGACGAACGCTGGCGGCGTGCCTAATACATGCAAGTCGAGCGAGC
GGAACTAACAGATTTACTTCGGTAATGACGTTAGGAAAGCGAGCGGCGGATGGGTGAGTAACACGTGGGG
AACCTGCCCCATAGTCTGGGATACCACTTGGAAACAGGTGCTAATACCGGATAAGAAAGCAGATCGCATG
ATCAGCTTTTAAAAGGCGGCGTAAGCTGTCGCTATGGGATGGCCCCGCGGTGCATTAGCTAGTTGGTAAG
GTAAAGGCTTACCAAGGCGATGATGCATAGCCGAGTTGAGAGACTGATCGGCCACATTGGGACTGAGACA
CGGCCCAAACTCCTACGGGAGGCAGCAGTAGGGAATCTTCCACAATGGACGCAAGTCTGATGGAGCAACG
CCGCGTGAGTGAAGAAGGTTTTCGGATCGTAAAGCTCTGTTGTTGGTGAAGAAGGACAGAGGTAGTAACT
GGCCTTTATTTGACGGTAATCAACCAGAAAGTCACGGCTAACTACGTGCCAGCAGCCGCGGTAATACGTA
GGTGGCAAGCGTTGTCCGGATTTATTGGGCGTAAAGCGAGCGCAGGCGGAAGAATAAGTCTGATGTGAAA
GCCCTCGGCTTAACCGAGGAACTGCATCGGAAACTGTTTTTCTTGAGTGCAGAAGAGGAGAGTGGAACTC
CATGTGTAGCGGTGGAATGCGTAGATATATGGAAGAACACCAGTGGCGAAGGCGGCTCTCTGGTCTGCAA
CTGACGCTGAGGCTCGAAAGCATGGGTAGTGAACAGGATTAGATACCCTGGTAGTCCATGCCGTAAACGA
TGAGTGCTAAGTGTTGGGAGGTTTCCGCCTCTCAGTGCTGCAGCTAACGCATTAAGCACTCCGCCTGGGG
AGTACGACCGCAAGGTTGAAACTCAAAGGAATTGACGGGGGCCCGCACAAGCGGTGGAGCATGTGGTTTA
ATTCGAAGCAACGCGAAGAACCTTACCAGGTCTTGACATCTAGTGCCATTTGTAGAGATACAAAGTTCCC
TTCGGGGACGCTAAGACAGGTGGTGCATGGCTGTCGTCAGCTCGTGTCGTGAGATGTTGGGTTAAGTCCC
GCAACGAGCGCAACCCTTGTTATTAGTTGCCAGCATTAAGTTGGGCACTCTAATGAGACTGCCGGTGACA
AACCGGAGGAAGGTGGGGATGACGTCAAGTCATCATGCCCCTTATGACCTGGGCTACACACGTGCTACAA
TGGGCAGTACAACGAGAAGCGAGCCTGCGAAGGCAAGCGAATCTCTGAAAGCTGTTCTCAGTTCGGACTG
CAGTCTGCAACTCGACTGCACGAAGCTGGAATCGCTAGTAATTGCGGATCAGCACGCCGCGGTGAATACG
TTCCCGGGCCTTGTACACACCGCCCGTCACACCATGGGAGTCTGCAATGCCCAAAGCCGGTGGCCTAACC
TTCGGGAAGGAGCCGTCTAAGGCAGGGCAGATGACTGGGGTGAAGTCGTAACAAGGTAGCCGTAAAGGGC
GAA
>323669187|emb|FR683088.1|L.crispatus|Lactobacillus crispatus partial 16S
rRNA gene, type strain DSM 20584T
GCCGGCGTGCCTAATACATGCAAGTCGAGCGAGCGGAACTAACAGATTTACTTCGGTAATGACGTTAGGA
AAGCGAGCGGCGGATGGGTGAGTAACACGTGGGGAACCTGCCCCATAGTCTGGGATACCACTTGGAAACA
GGTGCTAATACCGGATAAGAAAGCAGATCGCATGATCAGCTTTTAAAAGGCGGCGTAAGCTGTCGCTATG
GGATGGCCCCGCGGTGCATTAGCTAGTTGGTAAGGTAAAGGCTTACCAAGGCGATGATGCATAGCCGAGT
TGAGAGACTGATCGGCCACATTGGGACTGAGACACGGCCCAAACTCCTACGGGAGGCAGCAGTAGGGAAT
CTTCCACAATGGACGCAAGTCTGATGGAGCAACGCCGCGTGAGTGAAGAAGGTTTTCGGATCGTAAAGCT
CTGTTGTTGGTGAAGAAGGATAGAGGTAGTAACTGGCCTTTATTTGACGGTAATCAACCAGAAAGTCACG
GCTAACTACGTGCCAGCAGCCGCGGTAATACGTAGGTGGCAAGCGTTGTCCGGATTTATTGGGCGTAAAG
CGAGCGCAGGCGGAAGAATAAGTCTGATGTGAAAGCCCTCGGCTTAACCGAGGAACTGCATCGGAAACTG

TTTTTCTTGAGTGCAGAAGAGGAGAGTGGAACTCCATGTGTAGCGGTGGAATGCGTAGATATATGGAAGA
ACACCAGTGGCGAAGGCGGCTCTCTGGTCTGCAACTGACGCTGAGGCTCGAAAGCATGGGTAGCGAACAG
GATTAGATACCCTGGTAGTCCATGCCGTAAACGATGAGTGCTAAGTGTTGGGAGGTTTCCGCCTCTCAGT
GCTGCAGCTAACGCATTAAGCACTCCGCCTGGGGAGTACGACCGCAAGGTTGAAACTCAAAGGAATTGAC
GGGGGCCCGCACAAGCGGTGGAGCATGTGGTTTAATTCGAAGCAACGCGAAGAACCTTACCAGGTCTTGA
CATCTAGTGCCATTTGTAGAGATACAAAGTTCCCTTCGGGGACGCTAAGACAGGTGGTGCATGGCTGTCG
TCAGCTCGTGTCGTGAGATGTTGGGTTAAGTCCCGCAACGAGCGCAACCCTTGTTATTAGTTGCCAGCAT
TAAGTTGGGCACTCTAATGAGACTGCCGGTGACAAACCGGAGGAAGGTGGGGATGACGTCAAGTCATCAT
GCCCCTTATGACCTGGGCTACACACGTGCTACAATGGGCAGTACAACGAGAAGCGAGCCTGCGAAGGCAA
GCGAATCTCTGAAAGCTGTTCTCAGTTCGGACTGCAGTCTGCAACTCGACTGCACGAAGCTGGAATCGCT
AGTAATCGCGGATCAGCACGCCGCGGTGAATACGTTCCCGGGCCTTGTACACACCGCCCGTCACACCATG
GGAGTCTGCAATGCCCAAAGCCGGTGGCCTAACCTTCGGGAAGGAGCCGTCTAAGGCAGGGCAGATGACT
GGGGTGAAGTCGTAACAAGGTAGCCGTAGGAGAACCTGCGGTTGGA
>310941361|dbj|AB597000.1|L.crispatus|Lactobacillus crispatus gene for 16S rRNA, partial sequence, strain: CE3
GAGTTTGATCCTGGCTCAGGACGAACGCTGGCGGCGTGCCTAATACATGCAAGTCGAGCGAGCGGAACTA
ACAGATTTACTTCGGTAATGACGTTAGGAAAGCGAGCGGCGGATGGGTGAGTAACACGTGGGGAACCTGC
CCCATAGTCTGGGATACCACTTGGAAACAGGTGCTAATACCGGATAAGAAAGCAGATCGCATGATCAGCT
TTTAAAAGGCGGCGTAAGCTGTCGCTATGGGATGGCCCCGCGGTGCATTAGCTAGTTGGTAAGGTAAAGG
CTTACCAAGGCGATGATGCATAGCCGAGTTGAGAGACTGATCGGCCACATTGGGACTGAGACACGGCCCA
AACTCCTACGGGAGGCAGCAGTAGGGAATCTTCCACAATGGACGCAAGTCTGATGGAGCAACGCCGCGTG
AGTGAAGAAGGTTTTCGGATCGTAAAGCTCTGTTGTTGGTGAAGAAGGATAGAGGTAGTAACTGGCCTTT
ATTTGACGGTAATCAACCAGAAAGTCACGGCTAACTACGTGCCAGCAGCCGCGGTAATACGTAGGTGGCA
AGCGTTGTCCGGATTTATTGGGCGTAAAGCGAGCGCAGGCGGAAGAATAAGTCTGATGTGAAAGCCCTCG
GCTTAACCGAGGAACTGCATCGGAAACTGTTTTTCTTGAGTGCAGAAGAGGAGAGTGGAACTCCATGTGT
AGCGGTGGAATGCGTAGATATATGGAAGAACACCAGTGGCGAAGGCGGCTCTCTGGTCTGCAACTGACGC
TGAGGCTCGAAAGCATGGGTAGCGAACAGGATTAGATACCCTGGTAGTCCATGCCGTAAACGATGAGTGC
TAAGTGTTGGGAGGTTTCCGCCTCTCAGTGCTGCAGCTAACGCATTAAGCACTCCGCCTGGGGAGTACGA
CCGCAAGGTTGAAACTCAAAGGAATTGACGGGGGCCCGCACAAGCGGTGGAGCATGTGGTTTAATTCGAA
GCAACGCGAAGAACCTTACCAGGTCTTGACATCTAGTGCCATTTGTAGAGATACAAAGTTCCCTTCGGGG
ACGCTAAGACAGGTGGTGCATGGCTGTCGTCAGCTCGTGTCGTGAGATGTTGGGTTAAGTCCCGCAACGA
GCGCAACCCTTGTTATTAGTTGCCAGCATTAAGTTGGGCACTCTAATGAGACTGCCGGTGACAAACCGGA
GGAAGGTGGGGATGACGTCAAGTCATCATGCCCCTTATGACCTGGGCTACACACGTGCTACAATGGGCAG
TACAACGAGAAGCGAGCCTGCGAAGGCAAGCGAATCTCTGAAAGCTGTTCTCAGTTCGGACTGCAGTCTG
CAACTCGACTGCACGAAGCTGGAATCGCTAGTAATCGCGGATCAGCACGCCGCGGTGAATACGTTCCCGG
GCCTTGTACACACCGCCCGTCACACCATGGGAGTCTGCAATGCCCAAAGCCGGTGGCCTAACCTTCGGGA
AGGAGCCGTCTAAGGCAGGGCAGATGACTGGGGTGAAGTCGTAACAAGGTAGCC
>255040323|gb|FJ557001.1|L.crispatus|Lactobacillus crispatus strain BL221 16S ribosomal RNA gene, partial sequence
CGCTGGCGGCGTGCCTAATACATGCAAGTCGAGCGAGCGGAACTAACAGATTTACTTCGGTAATGACGTT
AGGAAAGCGAGCGGCGGATGGGTGAGTAACACGTGGGGAACCTGCCCCATAGTCTGGGATACCACTTGGA
AACAGGTGCTAATACCGGATAAGAAAGCAGATCGCATGATCAGCTTTTAAAAGGCGGCGTAAGCTGTCGC
TATGGGATGGCCCCGCGGTGCATTAGCTAGTTGGTAAGGTAAAGGCTTACCAAGGCGATGATGCATAGCC
GAGTTGAGAGACTGATCGGCCACATTGGGACTGAGACACGGCCCAAACTCCTACGGGAGGCAGCAGTAGG
GAATCTTCCACAATGGACGCAAGTCTGATGGAGCAACGCCGCGTGAGTGAAGAAGGTTTTCGGATCGTAA
AGCTCTGTTGTTGGTGAAGAAGGATAGAGGTAGTAACTGGCCTTTATTTGACGGTAATCAACCAGAAAGT
CACGGCTAACTACGTGCCAGCAGCCGCGGTAATACGTAGGTGGCAAGCGTTGTCCGGATTTATTGGGCGT
AAAGCGAGCGCAGGCGGAAGAATAAGTCTGATGTGAAAGCCCTCGGCTTAACCGAGGAACTGCATCGGAA
ACTGTTTTTCTTGAGTGCAGAAGAGGAGAGTGGAACTCCATGTGTAGCGGTGGAATGCGTAGATATATGG
AAGAACACCAGTGGCGAAGGCGGCTCTCTGGTCTGCAACTGACGCTGAGGCTCGAAAGCATGGGTAGCGA
ACAGGATTAGATACCCTGGTAGTCCATGCCGTAAACGATGAGTGCTAAGTGTTGGGAGGTTTCCGCCTCT
CAGTGCTGCAGCTAACGCATTAAGCACTCCGCCTGGGGAGTACGACCGCAAGGTTGAAACTCAAAGGAAT
TGACGGGGGCCCGCACAAGCGGTGGAGCATGTGGTTTAATTCGAAGCAACGCGAAGAACCTTACCAGGTC
TTGACATCTAGTGCCATTTGTAGAGATACAAAGTTCCCTTCGGGGACGCTAAGACAGGTGGTGCATGGCT

GTCGTCAGCTCGTGTCGTGAGATGTTGGGTTAAGTCCCGCAACGAGCGCAACCCTTGTTATTAGTTGCCA
GCATTAAGTTGGGCACTCTAATGAGACTGCCGGTGACAAACCGGAGGAAGGTGGGGATGACGTCAAGTCA
TCATGCCCCTTATGACCTGGGCTACACACGTGCTACAATGGGCAGTACAACGAGAAGCGAGCCTGCGAAG
GCAAGCGAATCTCTGAAAGCTGTTCTCAGTTCGGACTGCAGTCTGCAACTCGACTGCACGAAGCTGGAAT
CGCTAGTAATCGCGGATCAGCACGCCGCGGTGAATACGTTCCCGGGCCTTGTACACACCGCCCGTCACAC
CATGGGAGTCTGCAATG
>52222197|gb|AY724740.1|BVAB2|Uncultured bacterium clone 123-f 23 16S ribosomal RNA gene, partial sequence
TGGGGAATATTGGGCAATGGGCGAAAGCCTGACCCAGCAACGCCGCGTGAGTGATGAAGGCCTTCGGGTT
GTAAAACTCTTTGGACAGGGACGAAGAAAGTGACGGTACCTGTAGAACAAGCCACGGCTAACTACGTGCC
AGCAGCCGCGGTAATACGTAGGTGGCGAGCGTTATCCGGATTTACTGGGCGTAAAGGGCGTGTAGGCGGC
TAGATAAGTGTGATGTTTAAATCCAAGGCTTAACCTTGGGGTTCATTACAAACTGTTTAGCTTGAGTGCT
GGAGAGGATAGTGGAATTCCTAGTGTAGCGGTAAAATGCGTAGATATTAGGAGGAACACCGGTGGCGAAG
GCGGCTATCTGGACAGTAACTGACGCTGAGGCGCGAAAGCGTGGGGAGCAAACAGGATTAGATACCCTGG
TAGTCCACGCCGTAAACGATGAATACTAGCTGTAGGAGGTATCGACCCCTTCTGTGGCGCAGTTAACACA
ATAAGTATTCCGCCTGGGGAGTACGGCCGCAAGGTTAAAACTCAAAGGAATTGACGGGGACCCGCACAAG
CAGTGGATTATGTGGTTTAATTCGAAGCAACGCGAAGAACCTTACCAGGACTTGACATCCTCTGACGATT
CAGGAGACTGAATTTTCTCTTCGGAGACAGAGAGACAGGTGGTGCATGGTTGTCGTCAGCTCGTGTCGTG
AGATGTTGGGTTAAGTCCCGCAACGAGCGCAACCCCTATTGATTGTTGCTAACAGTAAGATGAGCACTCA
ATTGAGACTGCCGTTGATAAAACGGAGGAAGGTGGGGACGACGTCAAATCATCATGCCCCTTATGTTCTG
GGCTACACACGTAATACAATGGCTGTGACAGAGGGAAGCAAGAGGGCGACCTTAAGCGAATCCCAAAACG
CAGTCTCAGTTCGGATTGCAGGCTGCAACTCGCCTGCATGAAGTCGGAATTGCTAGTAATGGCAGGTCAG
CATACTGCCGTGAATACGTTCCCGGGTCT
>63146135|gb|AY995270.1|BVAB2|Uncultured bacterium clone FX88B4-11 16S ribosomal RNA gene, partial sequence
TGGGGGTGCTACCATGCAAGTCGAACGGAGTTAATTTGAGGAAGCAAGCTTGCTTGAAAAATTAAATTAA
CTTAGTGGCGGACGGGCGAGTAACACGTGAGCAACCTGCCTCTTACAGGGGAATAACAACGGGAAACCGT
TGCTAATACCGCATAACATGTTGAAAGGGCATCCTTTTAACATCAAAGGAGCAATCCGGTAAGAGATGGG
CTCGCGTCCGATTAGCTAGTTGGTAGGGTAACGGCCTACCAAGGCGACGATCGGTAGCCGGACTGAGAGG
TCGAACGGCCGCATTGGGACTGAGACACGGCCCAGACTCCTACGGGAGGCAGCAGTGGGGAATATTGGGC
AATGGGCGAAAGCCTGACCCAGCAACGCCGCGTGAGTGATGAAGGCCTTCGGGTTGTAAAACTCTTTGGA
CAGGGACGAAGAAAGTGACGGTACCTGTAGAACAAGCCACGGCTAACTACGTGCCAGCAGCCGCGGTAAT
ACGTAGGTGGCGAGCGTTATCCGGATTTACTGGGCGTAAAGGGCGTGTAGGCGGCTAGATAAGTGTGATG
TTTAAATCCAAGGCTTAACCTTGGGGTTCATTACAAACTGTTTAGCTTGAGTGCTGGAGAGGATAGTGGA
ATTCCTAGTGTAGCGGTAAAATGCGTAGATATTAGGAGGAACACCGGTGGCGAAGGCGGCTATCTGGACA
GTAACTGACGCTGAGGCGCGAAAGCGTGGGGAGCAAACAGGATTAGATACCCTGGTAGTCCACGCCGTAA
ACGATGAATACTAGCTGTAGGAGGTATCGACCCCTTCTGCGGCGCAGTTAACACAATAAGTATTCCGCCT
GGGGAGTACGGCCGCAAGGTTAAAACTCAAAGGAATTGACGGGGACCCGCACAAGCAGTGGATTATGTGG
TTTAATTCGAAGCAACGCGAAGAACCTTACCAGGACTTGACATCCTCTGACGATTCAGGAGACTGAATTT
TCTCTTCGGAGACAGAGAGACAGGTGGTGCATGGTTGTCGTCAGCTCGTGTCGTGAGATGTTGGGTTAAG
TCCCGCAACGAGCGCAACCCCTATTGATTGTTGCTAACAGTAAGATGAGCACTCAATTGAGACTGCCGTT
GATAAAACGGAGGAAGGTGGGGACGACGTCAAATCATCATGCCCCTTATGTTCTGGGCTACACACGTAAT
ACAATGGCTGTGACAGAGGGAAGCAAGAGGGCGACCTTAAGCGAATCCCAAAACGCAGTCTCAGTTCGGA
TTGCAGGCTGCAACTCGCCTGCATGAAGTCGGAATTGCTAGTAATGGCAGGTCAGCATACTGCCCGGTAT
>66878594|gb|AY958888.1|BVAB2|Uncultured bacterium clone rRNA115 16S ribosomal RNA gene, partial sequence
GCCCTTAGAGTTTGATCCTGGCTCAGGATGAACGCTGGCGGCGTGCTTAACACATGCAAGTCGAACGGAG
TTAATTTGAGGAAGCAAGCTTGCTTGAAGAATTAAATTAACTTAGTGGCGGACGGGCGAGTAACACGTGA
GCAACCTGCCTCTTACAGGGGAATAACAACGGGAAACCGTTGCTAATACCGCATAACATGTTGAAAGGGC
ATCCTTTTAACATCAAAGGAGCAATCCGGTAAGAGATGGGCTCGCGTCCGATTAGCTAGTTGGTAGGGTA
ACGGCCTACCAAGGCGACGATCGGTAGCCGGACTGAGAGGTCGAACGGCCGCATTGGGACTGAGACACGG
CCCAGACTCCTACGGGAGGCAGCAGTGGGGAATATTGGGCAATGGGCGAAAGCCTGACCCAGCAACGCCG
CGTGAGTGATGAAGGCCTTCGGGTTGTAAAACTCTTTGGACAGGGACGAAGAAAGTGACGGTACCTGTAG
AACAAGCCACGGCTAACTACGTGCCAGCAGCCGCGGTAATACGTAGGTGGCGAGCGTTATCCGGATTTAC

TGGGCGTAAAGGGCGTGTAGGCGGCTAGATAAGTGTGATGTTTAAATCCAAGGCTTAACCTTGGGGTTCA
TTACAAACTGTTTAGCTTGAGTGCTGGAGAGGATAGTGGAATTCCTAGTGTAGCGGTAAAATGCGTAGAT
ATTAGGAGGAACACCGGTGGCGAAGGCGGCTATCTGGACAGTAACTGACGCTGAGGCGCGAAAGCGTGGG
GAGCAAACAGGATTAGATACCCTGGTAGTCCACGCCGTAAACGATGAATACTAGCTGTAGGAGGTATCGA
CCCCTTCTGTGGCGCAGTTAACACAATAAGTATTCCGCCTGGGGAGTACGGCCGCAAGGTTAAAACTCAA
AGGAATTGACGGGGACCCGCACAAGCAGTGGATTATGTGGTTTAATTCGAAGCAACGCGAAGAACCTTAC
CAGGACTTGACATCCTCTGACGATTCAGGAGACTGAATTTTCTCTTCGGAGACAGAGAGACAGGTGGTGC
ATGGTTGTCGTCAGCTCGTGTCGTGAGATGTTGGGTTAAGTCCCGCAACGAGCGCAACCCCTATTGATTG
TTGCTAACAGTAAGATGAGCACTCAATTGAGACTGCCGTTGATAAAACGGAGGAAGGTGGGGACGACGTC
AAATCATCATGCCCCTTATGTTCTGGGCTACACACGTAATACAATGGCTGTGACAGAGGGAAGCAAGAGG
GCGACCTTAAGCGAATCCCAAAACGCAGTCTCAGTTCGGATTGCAGGCTGCAACTCGCCTGCATGAAGTC
GGAATTGCTAGTAATGGCAGGTCAGCATACTGCCGTGAATACGTTCCCGGGTCTTGTACACACCGCCCGT
CACACCATGAGAGTTTACAACACCCAAAGTCAGTAGCTTAACAGCAATGAGGGCGCTGCCTAAGGTGGGG
TAAATAATTGGGGTGAAGTCGTAACAAGGTAACCGTAAAGGGC
>145582947|emb|AM697115.1|BVAB2|Uncultured bacterium partial 16S rRNA
gene, isolate BF0002B065
GATGAACGCTGGCGGCGTGCTTAACACATGCAAGTCGAACGGAGTTAATTTGAGGAAGCAAGCTTGTTTG
AAGAATTAAATTAACTTAGTGGCGGACGGGCGAGTAACACGTGAGCAACCTGCCTCTTACAGGGGAATAA
CAACGGGAAACCGTTGCTAATACCGCATAACATGTTGAAAGGGCATCCTTTTAACATCAAAGGAGCAATC
CGGTAAGAGATGGGCTCGCGTCCGATTAGCTAGTTGGTAGGGTAACGGCCTACCAAGGCGACGATCGGTA
GCCGGACTGAGAGGTCGAACGGCCGCATTGGGACTGAGACACGGCCCAGACTCCTACGGGAGGCAGCAGT
GGGGAATATTGGGCAATGGGCAAAAGCCTGACCCAGCAACGCCGCGTGAGTGATGAAGGCCTTCGGGTTG
TAAAACTCTTTGGACAGGGACGAAGAAAGTGACGGTACCTGTAGAACAAGCCACGGCTAACTACGTGCCA
GCAGCCGCGGTAATACGTAGGTGGCGAGCGTTATCCGGATTTACTGGGCGTAAAGGGCGTGTAGGCGGCT
AGATAAGTGTGATGTTTAAATCCAAGGCTTAACCTTGGGGTTCATTACAAACTGTTTAGCTTGAGTGCTG
GAGAGGATAGTGGAATTCCTAGTGTAGCGGTAAAATGCGTAGATATTAGGAGGAACACCGGTGGCGAAGG
CGGCTATCTGGACAGTAACTGACGCTGAGGCGCGAAAGCGTGGGGAGCAAACAGGATTAGATACCCTGGT
AGTCCACGCCGTAAACGATGAATACTAGCTGTAGGAGGTATCGACCCCTTCTGTGGCGCAGTTAACACAA
TAAGTATTCCGCCTGGGGAGTACGGCCGCAAGGTTAAAACTCAAAGGAATTGACGGGGACCCGCACAAGC
AGTGGATTATGTGGTTTAATTCGAAGCAACGCGAAGAACCTTACCAGGACTTGACATCCTCTGACGATTC
AGGAGACTGAATTTTCTCTTCGGAGGCAGAGAGACAGGTGGTGCATGGTTGTCGTCAGCTCGTGTCGTGA
GATGTTGGGTTAAGTCCCGCAACGAGCGCAACCCCTATTGATTGTTGCTAACAGTAAGATGAGCACTCAA
TTGAGACTGCCGTTGATAAAACGGAGGAAGGTGGGGACGACGTCAAATCATCATGCCCCTTATGTTCTGG
GCTACACACGTAATACAATGGCTGTGACAGAGGGAAGCAAGAGGGCGACCTTAAGCGAATCCCAAAACGC
AGTCTCAGTTCGGATTGCAGGCTGCAACTCGCCTGCATGAAGTCGGAATTGCTAGTAATGGCAGGTCAGC
ATACTGCCGTGAATACGTTCCCGGGTCTTGTACACACCGCCCGTCACACCATGAGAGTTTACAACACCCA
AAGTCAGTAGCTTAACAGCAATGAGGGCGCTGCCTAAGGTGGGGTAAATAATTTGGGGTGAAGTCGTAAC
AAGGTAGCCGTATCGGAAGG
>63146137|gb|AY995272.1|BVAB2|Uncultured bacterium clone FX8B4-11 16S
ribosomal RNA gene, partial sequence (95%)
GGGGGCGGCTACACATGCAGTCGAACGGAGTTAATTTGAGGAAGCAAGCTTGCTTGAAAAATTAAATTAA
CTTAGTGGCGGACGGGCGAGTAACACGTGAGCAACCTGCCTCTTACAGGGGAATAACAACGGGAAACCGT
TGCTAATACCGCATAACATGTTGAAAGGGCATCCTTTTAACATCAAAGGAGCAATCCGGTAAGAGATGGG
CTCGCGTCCGATTAGCTAGTTGGTAGGGTAACGGCCTACCAAGGCGACGATCGGTAGCCGGACTGAGAGG
TCGAACGGCCGCATTGGGACTGAGACACGGCCCAGACTCCTACGGGAGGCAGCAGTGGGGAATATTGGGC
AATGGGCGAAAGCCTGACCCAGCAACGCCGCGTGAGTGATGAAGGCCTTCGGGTTGTAAAACTCTTTGGA
CAGGGACGAAGAAAGTGACGGTACCTGTAGAACAAGCCACGGCTAACTACGTGCCAGCAGCCGCGGTAAT
ACGTAGGTGGCGAGCGTTATCCGGATTTACTGGGCGTAAAGGGCGTGTAGGCGGCTAGATAAGTGTGATG
TTTAAATCCAAGGCTTAACCTTGGGGTTCATTACAAACTGTTTAGCTTGAGTGCTGGAGAGGATAGTGGA
ATTCCTAGTGTAGCGGTAAAATGCGTAGATATTAGGAGGAACACCGGTGGCGAAGGCGGCTATCTGGACA
GTAACTGACGCTGAGGCGCGAAAGCGTGGGGAGCAAACAGGATTAGATACCCTGGTAGTCCACGCCGTAA
ACGATGAATACTAGCTGTAGGAGGTATCGACCCCTTCTGTGGCGCAGTTAACACATAAGTATTCCGCCTG
GGGAGTACGGCCACAAGGTTGAAACTCAAAGGAATTGACGGGGGCCCGCTCAAGCAGTGGAGTATGTGGT
TTAATTAGAAGCAACGCGAAGAACCTTACCAGGGTTTGTCATCCTTTGAACGATGAAGAGATAGATATTT

CCATTCGGGGACAAGGAGACAGGTGGTGCATGGTTGTCGTCAGCTCGTGTCGTGAGATGTTGGGTTAAGT
CCCGCAACGAGCCCAACCCCTATTGCCAGTTGCCATCATTTAGTTGGGCACTCAGGTGAGACTGCCGTTG
ATAAAACGGAGGAAGGTGGGGATGACGTCAAATCATCATGCCCCTTATATTCTGGGCTACACACGTAATA
CAATGGCTACAACAGAGAGAAGCGAGAGGGCGACCCTAAGCAAATCCCCAAAAGCAGTCTCAGTTCGAAT
GGCAGGCTGCAACTCGCCTGCATGAAGTCGGAATTGCTAGTAATGGCAGGTCAGCAAACTTCTTGTTTTT
C
>52222198|gb|AY724741.1|BVAB3|Uncultured bacterium clone 123f 17 16S ribosomal RNA gene, partial sequence
TGGGGAATATTGGGCAATGGGCGAAAGCCTGACCCAGCAACGCCGCGTGAAGTATGAAGGCCTTCGGGTT
GTAAACTTCTTTGATCAGGGAAGAAACAAATGACGGTACCTGAAAAACAAGCCACGGCTAACTACGTGCC
AGCAGCCGCGGTAATACGTAGGTGGCGAGCGTTATCCGGATTTACTGGGTGTAAAGGGCGTGCAGGCGGG
CTGATAAGTCAGATGTGAAATCCCCGAGCTTAACTCGGGAACTGCATCTGATACTGTTGGTCTTGAGTGC
TGGAGAGGATAGTGGAATTCCTAGTGTAGCGGTAAAATGCGCAGATATTAGGAGGAACACCAGTGGCGAA
GGCGGCTATCTGGACAGTAACTGACGCTGAGGCGCGAAAGCGTGGGTAGCAAACAGGATTAGATACCCTG
GTAGTCCACGCCGTAAACGATGATTACTAGGTGTAGGAGGTATCGACCCCTTCTGTGCCGGAGTTAACAC
AATAAGTAATCCACCTGGGGAGTACGGCCGCAAGGTTGAAACTCAAAGGAATTGACGGGGGCCCGCACAA
GCAGTGGAGTATGTGGTTTAATTCGACGCAACGCGAAGAACCTTACCAGGGTTTGACATCCCTTGAACGA
TGTAGAGATACATAATTCCCTTCGGGGACAAGGAGACAGGTGGTGCATGGTTGTCGTCAGCTCGTGTCGT
GAGATGTTGGGTTAAGTCCCGCAACGAGCGCAACCCCTATTGCCAGTTGCCATCATTTAGTTGGGCACTC
AGGCGAGACTGCCGTTGATAAAACGGAGGAAGGTGGGGATGACGTCAAATCATCATGCCCCTTATATCCT
GGGCTACACACGTACTACAATGGCTACAACAGAGAGCAGCGACGTCGCGAGGCGAAGCAAATCCCCAAAT
GTAGTCTCAGTTCGGATTGCAGGCTGCAACTCGCCTGCATGAAGCCGGAATTGCTAGTAATGGCAGGTCA
GCATACTGCCGTGAATACGTTCTCGGGCCT
>306850711|gb|GQ900635.1|BVAB3|Bacterium BVAB3-Strain 5 16S ribosomal RNA gene, partial sequence
GATGAACGCTGGCGGCGTGCTTAACACATGCAAGTCGAACGGAGCTTTGAGGAAGTGCTTGCACAGAATT
AAAGCTTAGTGGCGGACGGGTGAGTAACGCGTGAGGAACCTGCCTTTCACAGTGGAATAACAACGGGAAA
CCGTTGCTAATGGCGCATGACATCGCATTGAGGCATCTCAGAGCGATTAAAGGAGAAATCCGGTGAAAGA
TGGACTCGCGTCCGATTAGCTAGTTGGTGAGGTAACGGCCCACCAAGGCGACGATCGGTAGCCGAACTGA
GAGGTTGATCGGCCACATTGGGACTGAGACACGGCCCAGACTCCTACGGGAGGCAGCAGTGGGGAATATT
GGGCAATGGGCGAAAGCCTGACCCAGCAACGCCGCGTGAAGTATGAAGGCCTTCGGGTTGTAAACTTCTT
TGATCAGGGAAGAAACAAATGACGGTACCTGAAAAACAAGCCACGGCTAACTACGTGCCAGCAGCCGCGG
TAATACGTAGGTGGCGAGCGTTATCCGGATTTACTGGGTGTAAAGGGCGTGCAGGCGGGCTGATAAGTCA
GATGTGAAATCCCCGAGCTTAACTCGGGAACTGCATCTGATACTGTTGGTCTTGAGTGCTGGAGAGGATA
GTGGAATTCCTAGTGTAGCGGTAAAATGCGCAGATATTAGGAGGAACACCAGTGGCGAAGGCGGCTATCT
GGACAGTAACTGACGCTGAGGCGCGAAAGCGTGGGTAGCAAACAGGATTAGATACCCTGGTAGTCCACGC
CGTAAACGATGATTACTAGGTGTAGGAGGTATCGACCCCTTCTGTGCCGGAGTTAACACAATAAGTAATC
CACCTGGGGAGTACGGCCGCAAGGTTGAAACTCAAAGGAATTGACGGGGGCCCGCACAAGCAGTGGAGTA
TGTGGTTTAATTCGACGCAACGCGAAGAACCTTACCAGGGTTTGACATCCCTTGAACGATGTAGAGATAC
ATAATTCCCTTCGGGGACAAGGAGACAGGTGGTGCATGGTTGTCGTCAGCTCGTGTCGTGAGATGTTGGG
TTAAGTCCCGCAACGAGCGCAACCCCTATTGCCAGTTGCCATCATTTAGTTGGGCACTCAGGCGAGACTG
CCGTTGATAAAACGGAGGAAGGTGGGGATGACGTCAAATCATCATGCCCCTTATATCCTGGGCTACACAC
GTACTACAATGGCTACAACAGAGAGCAGCGACGTCGCGAGGCGAAGCAAATCCCCAAATGTAGTCTCAGT
TCGGATTGCAGGCTGCAACTCGCCTGCATGAAGCCGGAATTGCTAGTAATGGCAGGTCAGCATACTGCCG
TGAATACGTTCTCGGGCCTTGTACACACCGCCCGTCACACCATGAGAGTCAGTAACACCCGAAGTCAGTA
GTCTAACCGCAAGGGGGACGCTGCCGAAGGTGGGACCGATAATTGGGGTGAAGTCGTAACAAGGTAGCCG
TATCGGAAGG
>63146139|gb|AY995274.1|BVAB3|Uncultured bacterium clone FX77B4-11 16S ribosomal RNA gene, partial sequence
GCCGGGGTGCCTTACACATGCAAGTCGAACGGAGTTAATTTGAGGAAGCAAGCTTGCTTGAAGAATTGAA
TTAACTTAGTGGCGGACGGGCGAGTAACACGTGAGCAACCTGCCTCTTACAGGGGAATAACAACGGGAAA
CCGTTGCTAATACCGCATAACATGTTGAAAGGGCATCCTTTTAACATCAAAGGAGCAATCCGGTAAGAGA
TGGGCTCGCGTCCGATTAGTTAGTTGGTAGGGTAACGGCCTACCAAGGCGACGATCGGTAGCCGGACTGA
GAGGTCGAACGGCCGCATTGGGACTGAGACACGGCCCAGACTCCTACGGGAGGCAGCAGTGGGGAATATT

GGGCAATGGGCGAAAGCCTGACCCAGCAACGCCGCGTGAAGTATGAAGGCCTTCGGGTTGTAAACTTCTT
TGATCAGGGAAGAAACAAATGACGGTACCTGAAAAACAAGCCACGGCTAACTACGTGCCAGCAGCCGCGG
TAATACGTAGGTGGCGAGCGTTATCCGGATTTACTGGGTGTAAAGGGCGTGCAGGCGGGCTGATAAGTCA
GATGTGAAATCCCCGAGCTTAACTCGGGAACTGCATCTGATACTGTTGGTCTTGAGTGCTGGAGAGGATA
GTGGAATTCCTAGTGTAGCGGTAAAATGCGCAGATATTAGGAGGAACACCAGTGGCGAAGGCGGCTATCT
GGACAGTAACTGACGCTGAGGCGCGAAAGCGTGGGTAGCAACCAGGATTAGATACCCTGGTAGTCCACGC
CGTAAACGATGATTACTAGGTGTAGGAGGTATCGACCCCTTCTGTGCCGGAGTTAACACAATAAGTAATC
CACCTGGGGAGTACGGCCGCAAGGTTGAAACTCAAAGGAATTGACGGGGGCCCGCACAAGCAGTGGAGTA
TGTGGTTTAATTCGACGCAACGCGAAGAACCTTACCAAGGCTTGACATATAGTTGAGTTATTGAGAAATT
GATAAGTCCCTCGGGACAACTATACAGGTGGTGCATGGTTGTCGTCAGCTCGTGTCGTGAGATGTTGGGT
TAAGTCCCGCAACGAGCGCAACCCTTATTTTCAGTTACCAGCATTTAAGGTGGGGACTCTGAAGAGACTG
CCGATGACAAATCGGAGGAAGGTGGGGATGACGTCAAATCATCATGCCCTTTATGTCTTGGGCTACACAC
GTGCTACAATGGTCGGTACAACGAGAAGCGAGATAATGATGTTAAGCGAAACTCTAAAAGCCGATCTCAG
TTCGGATTGTAGGCTGCAACTCGCCTACATGAAGTCGGAGTTGCTAGTAATCGCGAATCAGAACGTCGCG
GGA
>63146138|gb|AY995273.1|BVAB3|Uncultured bacterium clone FX93B4-11 16S ribosomal RNA gene, partial sequence
TGCCGGGGGTGCTTAACACATGCAGTCGACGGAGCTTTGAGGAAGTGCTTGTACAGAATTAAAGCTTAGT
GGCGGACGGGTGAGTAACGCGTGAGGAACCTGCCTTTCACAGTGGAATAACAACGGGAAACCGTTGCTAA
TGGCGCATGACATCGCATTGAGGCATCTCAGAGCGATTAAAGGAGAAATCCGGTGAAAGATGGACTCGCG
TCCGATTAGCTAGTTGGTGAGGTAACGGCCCACCAAGGCGACGATCGGTAGCCGAACTGAGAGGTTGATC
GGCCACATTGGGACTGAGACACGGCCCAGACTCCTACGGGAGGCAGCAGTGGGGAATATTGGGCAATGGG
CGAAAGCCTGACCCAGCAACGCCGCGTGAAGTATGAAGGCCTTCGGGTTGTAAACTTCTTTGATCAGGGA
AGAAACAAATGACGGTACCTGAAAAACAAGCCACGGCTAACTACGTGCCAGCAGCCGCGGTAATACGTAG
GTGGCGAGCGTTATCCGGATTTACTGGGTGTAAAGGGCGTGCAGGCGGGCTGATAAGTCAGATGTGAAAT
CCCCGAGCTTAACTCGGGAACTGCATCTGATACTGTTAGTCTTGAGTGCTGGAGAGGATAGTGGAATTCC
TAGTGTAGCGGTAAAATGCGCAGATATTAGGAGGAACACCAGTGGCGAAGGCGGCTATCTGGACAGTAAC
TGACGCTGAGGCGCGAAAGCGTGGGTAGCAAACAGGATTAGATACCCTGGTAGTCCACGCCGTAAACGAT
GATTACTAGGTGTAGGAGGTATCGACCCCTTCTGTGCCGGAGTTAACACAATAAGTAATCCACCTGGGGA
GTACGGCCGCAAGGTTGAAACTCAAAGGAATTGACGGGGGCCCGCACAAGCAGTGGAGTATGTGGTTTAA
TTCGACGCAACGCGAAGAACCTTACCAGGGTTTGACATCCCTTGAACGATGTAGAGATACATAATTCCCT
TCGGGGACAAGGAGACAGGTGGTGCATGGTTGTCGTCAGCTCGTGTCGTGAGATGTTGGGTTAAGTCCCG
CAACGAGCGCAACCCCTATTGCCAGTTGCCATCATTTAGTTGGGCACTCAGGCGAGACTGCCGTTGATAA
AACGGAGGAAGGTGGGGATGACGTCAAATCATCATGCCCCTTATATCCTGGGCTACACACGTACTACAAT
GGCTACAACAGAGAGCAGCGACGTCGCGAGGCGAAGCAAATCCCCAAATGTAGTCTCAGTTCGGATTGCA
GGCTGCAACTCGCCTGCATGAAGCCGGAATTGCTAGTAATGGCAGGTCAGCATATTGCCGGAC
>52222194|gb|AY738705.1|Veillonella|Uncultured Veillonella sp. clone 7BVA-4 16S ribosomal RNA gene, partial sequence
TGGGGAATCTTCCGCAATGGACGAAAGTCTGACGGAGCAACGCCGCGTGAGTGATGACGGTCTTCGGATT
GTAAAGCTCTGTTAATCGGGACGAATGGTTTGTGTGCAAATAGTGCATAGACATGACGGTACCGGAATAG
AAAGCCACGGCTAACTACGTGCCAGCAGCCGCGGTAATACGTAGGTGGCAAGCGTTGTCCGGAATTATTG
GGCGTAAAGCGCGCGCAGGCGGACTAGCCAGTCAGTCTTAAAAGTTCGGGGCTTAACCCCGTGATGGGAT
TGAAACTACTAGTCTAGAGTATCGGAGAGGAAAGTGGAATTCCTAGTGTAGCGGTGAAATGCGTAGATAT
TAGGAAGAACACCAGTGGCGAAGGCGACTTTCTGGACGAACACTGACGCTGAGGCGCGAAAGCCAGGGGA
GCGAACGGGATTAGATACCCCGGTAGTCCTGGCCGTAAACGATGGGTACTAAGTGTGGGAGGTATCGACC
CCTTCCGTGCTGCAGTTAACGCAATAAGTACCCCGCCTGGGGAGTACGGTCGCAAGACTGAAACTCAAAG
GAATTGACGGGGGCCCGCACAAGCGGTGGAGTATGTGGTTTAATTCGACGCAACGCGAAGAACCTTACCA
GGTCTTGACATTGATGGACAGAACTAGAGATAGTTTTTCTTCTTCGGAAGCCAGAAAACAGGTGGTGCAC
GGTTGTCGTCAGCTCGTGTCGTGAGATGTTGGGTTAAGTCCCGCAACGAGCGCAACCCCTATCTTATGTT
GCCAGCACGTAATGGTGGGAACTCATGAGAGACTGCCGCAGACAATGCGGAGGAAGGCGGGGATGACGTC
AAATCATCATGCCCCTTATGACCTGGGCTACACACGTACTACAATGGGAGTTAATAAAGAGAAGCGAAAC
CGCGAGGTGGAGCGAACCTCACAAACACTCTCTCAGTTCGGATTGCAGGCTGCAACTCGCCTGCATGAAG
TCGGAATCGCTAGTAATCGCAGGTCAGCATACTGCGGTGAATACGTTCCCGGGCCT

>63146101|gb|AY995236.1|Veillonella|Uncultured Veillonella sp. clone FX50B4-3 16S ribosomal RNA gene, partial sequence
AGGGGGTGCTTTACACATGCAAGTCGAACGAAGAGACATGGAGCTTGCTCCTATGAATCTTAGTGGCGA
ACGGGTGAGTAACGCGTAATCAACCTGCCCTACAATGGGGGACAACAGTTGGAAACGACTGCTAATACCG
CATACGACCTACGATTGGCATCAATCGTAGGTGAAAGGTGGCCTCTGCATGTAAGCTATCGTTGTAGGAG
GGGATTGCGTCTGATTAGCTAGTTGGAGGGGTAACGGCCCACCAAGGCGATGATCAGTAGCCGGTCTGAG
AGGATGAACGGCCACATTGGGACTGAGACACGGCCCAAACTCCTACGGGAGGCAGCAGTGGGGAATCTTC
CGCAATGGACGAAAGTCTGACGGAGCAACGCCGCGTGAGTGATGACGGTCTTCGGATTGTAAAGCTCTGT
TAATCGGGACGAATGGTTTGTGTGCAAATAGTGCATAGACATGACGGTACCGGAATAGAAAGCCACGGCT
AACTACGTGCCAGCAGCCGCGGTAATACGTAGGTGGCAAGCGTTGTCCGGAATTATTGGGCGTAAAGCGC
GCGCAGGCGGACTAGCCAGTCAGTCTTAAAAGTTCGGGGCTTAACCCCGTGATGGGATTGAAACTACTAG
TCTAGAGTATCGGAGAGGAAAGTGGAATTCCTAGTGTAGCGGTGAAATGCGTAGATATTAGGAAGAACAC
CAGTGGCGAAGGCGACTTTCTGGACGAACACTGACGCTGAGGCGCGAAAGCCAGGGGAGCGAACGGGATT
AGATACCCCGGTAGTCCTGGCCGTAAACGATGGGTACTAAGTGTGGGAGGTATCGACCCCTTCCGTGCTG
CAGTTAACGCAATAAGTACCCCGCCTGGGGAGTACGGTCGCAAGACTGAAACTCAAAGGAATTGACGGGG
GCCCGCACAAGCGGTGGAGTATGTGGTTTAATTCGACGCAACGCGAAGAACCTTACCAGGTCTTGACATT
GATGGACAGAACTAGAGATAGTTTTTCTTTTTCGGAAGCCAGAAAACAGGTGGTGCACGGTTGTCGTCAG
CTCGTGTCGTGAGATATTGGGTTAAGTCCCGCAACGAGCGCAACCCCTATCTTATGTTGCCAGCACGTAA
TGGTGGGAACTCATGAGAGACTGCCGCAGACAATGCGGAGGAAGGCGGGGATGACGTCAAATCATCATGC
CCCTTATGACCTGGGCTACACACGTACTACAATGGGAGTTAATAAAGAGAAGCGAAACCGCGAGGTGGAG
CGAGCCTCACAAACACTCTCTCAGTTCGGATTGCAGGCTGCAACTCGCCTGCATGAAGTCGGAATCGCTA
GTAATCGCAGGTCAGCATACTGCGGGAC
>239923965|gb|GQ179687.1|Veillonella|Uncultured Veillonella sp. clone VE28C02 16S ribosomal RNA gene, partial sequence
GACGAACGCTGGCGGCGTGCTTAACACATGCAAGTCGAACGAAGAGACATGGAGCTTGCTCCTATGAAT
CTTAGTGGCGAACGGGTGAGTAACGCGTAATCAACCTGCCCTACAATGGGGGGACAACAGTTGGAAACGA
CTGCTAATACCGCATACGACCTACGATTGACATCAATCGTAGGTGAAAGGTGGCCTCTACATGTAAGCTA
TCGTTGTAGGAGGGGATTGCGTCTGATTAGCTAGTTGGAGGGGTAACGGCCCACCAAGGCGATGATCAGT
AGCCGGTCTGAGAGGATGAACGGCCACATTGGGACTGAGACACGGCCCAAACTCCTACGGGAGGCAGCAG
TGGGGAATCTTCCGCAATGGACGAAAGTCTGACGGAGCAACGCCGCGTGAGTGATGACGGTCTTCGGATT
GTAAAGCTCTGTTAATCGGGACGAATGGTTTGTGTGCAAATAGTGCATGGACATGACGGTACCGGAATAG
AAAGCCACGGCTAACTACGTGCCAGCAGCCGCGGTAATACGTAGGTGGCAAGCGTTGTCCGGAATTATTG
GGCGTAAAGCGCGCGCAGGCGGACTAGCCAGTCAGTCTTAAAAGTTCGGGGCTTAACCCCGTGATGGGAT
TGAAACTACTAGTCTAGAGTATCGGAGAGGAAAGTGGAATTCCTAGTGTAGCGGTGAAATGCGTAGATAT
TAGGAAGAACACCAGTGGCGAAGGCGACTTTCTGGACGAACACTGACGCTGAGGCGCGAAAGCCAGGGGA
GCGAACGGGATTAGATACCCCGGTAGTCCTGGCCGTAAACGATGGGTACTAAGTGTGGGAGGTATCGACC
CCTTCCGTGCTGCAGTTAACGCAATAAGTACCCCGCCTGGGGAGTACGGTCGCAAGACTGAAACTCAAAG
GAATTGACGGGGGCCCGCACAAGCGGTGGAGTATGTGGTTTAATTCGACGCAACGCGAAGAACCTTACCA
GGTCTTGACATTGATGGACAGAACTAGAGATAGTTTTTCTTCTTCGGAAGCCAGAAAACAGGTGGTGCAC
GGTTGTCGTCAGCTCGTGTCGTGAGATGTTGGGTTAAGTCCCGCAACGAGCGCAACCCCTATCTTATGTT
GCCAGCACGTAATGGTGGGAACTCATGAGAGACTGCCGCAGACAATGCGGAGGAAGGCGGGGATGACGTC
AAATCATCATGCCCCTTATGACCTGGGCTACACACGTACTACAATGGGAGTTAATAAAGAGAAGCGAAAC
CGCGAGGTGGAGCGAACCTCACAAACACTCTCTCAGTTCGGATTGCAGGCTGCAACTCGCCTGCATGAAG
TCGGAATCGCTAGTAATCGCAGGTCAGCATACTGCGGTGAATACGTTCCCGGGCCTTGTACACACCGCCC
GTCACACCACGAAAGTCGGAAGTACCCAAAGCCGGTGGGGTAACCTTCGGGAGCCAGCCGTCTAAGGTAA
AGTCGATGATTGGGGTGAAGTCGTAACAAGGTA
>52222191|gb|AY738702.1|Ureaplasma|Uncultured Ureaplasma sp. clone 127-f 65 16S ribosomal RNA gene, partial sequence
TAGGGAATTTTTCACAATGGGCGCAAGCCTTATGAAGCAATGCCGCGTGAACGATGAAGGTCTTATAGAT
TGTAAAGTTCTTTTATATGGGAAGAAACGCTAAAATAGGAAATGATTTTAGTTTGACTGTACCATTTGAA
TAAGTATCGGCTAACTATGTGCCAGCAGCCGCGGTAATACATAGGATGCAAGCGTTATCCGGATTTACTG
GGCGTAAAACGAGCGCAGGCGGGTTTGTAAGTTTGGTATTAAATCTAGATGCTTAACGTCTAGCTGTATC
AAAAACTGTAAACCTAGAGTGTAGTAGGGAGTTGGGGAACTCCATGTGGAGCGGTAAAATGCGTAGATAT
ATGGAAGAACACCGGTGGCGAAGGCGCCAACTTGGACTATCACTGACGCTTAGGCTCGAAAGTGTGGGGA

GCAAATAGGATTAGATACCCTAGTAGTCCACACCGTAAACGATCATCATTAAATGTCGGCCCGAATGGGT
CGGTGTTGTAGCTAACGCATTAAATGATGTGCCTGGGTAGTACATTCGCAAGAATGAAACTCAAACGGAA
TTGACGGGGACCCGCACAAGTGGTGGAGCATGTTGCTTAATTTGACAATACACGTAGAACCTTACCTAGG
TTTGACATCTATTGCGATGCTATAGAAATATAGTTGAGGTTAACAATATGACAGGTGGTGCATGGTTGTC
GTCAGCTCGTGTCGTGAGATGTTGGGTTAAGTCCCGCAACGAGCGCAACCCCTTTCGTTAGTTACTTTTC
TAGCGATACTGCTACCGCAAGGTAGAGGAAGGTGGGGATGACGTCAAATCATCATGCCCCTTATATCTAG
GGCTGCAAACGTGCTACAATGGCTAATACAAACTGCTGCAAAATCGTAAGATGAAGCGAAACAGAAAAAG
TTAGTCTCAGTTCGGATAGAGGGCTGCAATTCGTCCTCTTGAAGTTGGAATCACTAGTAATCGCGAATCA
GACATGTCGCGGTGAATACGTTCTCGGGTC
>4377989|gb|AF073457.1|U.parvum|Ureaplasma parvum serovar 14 16S
ribosomal RNA gene, partial sequence
ATTAACGCTGGCGGCATGCCTAATACATGCAAATCGAACGAAGCCTTTTAGGCTTAGTGGTGAACGGGTG
AGTAACACGTATCCAATCTACCCTTAAGTTGGGGATAACTAGTCGAAAGATTAGCTAATACCGAATAATA
ACATCAATATCGCATGAGAAGATGTAGAAAGTCGCTCTTTGTGGCGACGCTTTTGGATGAGGGTGCGACG
TATCAGATAGTTGGTGAGGTAATGGCTCACCAAGTCAATGACGCGTAGCTGTACTGAGAGGTAGAACAGC
CACAATGGGACTGAGACACGGCCCATACTCCTACGGGAGGCAGCAGTAGGGAATTTTTCACAATGGGCGC
AAGCCTTATGAAGCAATGCCGCGTGAACGATGAAGGTCTTATAGATTGTAAAGTTCTTTTATATGGGAAG
AAACGCTAAAATAGGAAATGATTTTAGTTTGACTGTACCATTTGAATAAGTATCGGCTAACTATGTGCCA
GCAGCCGCGGTAATACATAGGATGCAAGCGTTATCCGGATTTACTGGGCGTAAAACGAGCGCAGGCGGGT
TTGTAAGTTTGGTATTAAATCTAGATGCTTAACGTCTAGCTGTATCAAAAACTGTAAACCTAGAGTGTAG
TAGGGAGTTGGGGAACTCCATGTGGAGCGGTAAAATGCGTAGATATATGGAAGAACACCGGTGGCGAAGG
CGCCAACTTGGACTATCACTGACGCTTAGGCTCGAAAGTGTGGGGAGCAAATAGGATTAGATACCCTAGT
AGTCCACACCGTAAACGATCATCATTAAATGTCGGCCCGAATGGGTCGGTGTTGTAGCTAACGCATTAAA
TGATGTGCCTGGGTAGTACATTCGCAAGAATGAAACTCAAACGGAATTGACGGGGACCCGCACAAGTGGT
GGAGCATGTTGCTTAATTTGACAATACACGTAGAACCTTACCTAGGTTTGACATCTATTGCGATGCTATA
GAAATATAGTTGAGGTTAACAATATGACAGGTGGTGCATGGTTGTCGTCAGCTCGTGTCGTGAGATGTTG
GGTTAAGTCCCGCAACGAGCGCAACCCCTTTCGTTAGTTACTTTTCTAGCGATACTGCTACCGCAAGGTA
GAGGAAGGTGGGGATGACGTCAAATCATCATGCCCCTTATATCTAGGGCTGCAAACGTGCTACAATGGCT
AATACAAACTGCTGCAAAATCGTAAGATGAAGCGAAACAGAAAAAGTTAGTCTCAGTTCGGATAGAGGGC
TGCAATTCGTCCTCTTGAAGTTGGAATCACTAGTAATCGCGAATCAGACATGTCGCGGTGAATACGTTCT
CGGGTCTTGTACACACCGCCCGTCAAACTATGGGAGCTGGTAATATCTAAAACCGCAAAGCTAACCTTTT
GGAGGCATGCGTCTAGGGTAGGATCGGTGACTGGAGTTA
>507369|gb|U06095.1|U.urealyticum|UUU06095 Ureaplasma urealyticum U26
(serovar 14) 16S ribosomal RNA gene
ATTAACGCTGGCGGCATGCCTAATACATGCAAATCGAACGAAGCCTTTTAGGCTTAGTGGTGAACGGGTG
AGTAACACGTATCCAATCTACCCTTAAGTTGGGGATAACTAGTCGAAAGATTAGCTAATACCGAATAATA
ACATCAATATCGCATGAGAAGATGTAGAAAGTCGCTCTTTGTGGCGACGCTTTTGGATGAGGGTGCGACG
TATCAGATAGTTGGTGAGGTAATGGCTCACCAAGTCAATGACGCGTAGCTGTACTGAGAGGTAGAACAGC
CACAATGGGACTGAGACACGGCCCATACTCCTACGGGAGGCAGCAGTAGGGAATTTTTCACAATGGGCGC
AAGCCTTATGAAGCAATGCCGCGTGAACGATGAAGGTCTTATAGATTGTAAAGTTCTTTTATATGGGAAG
AAACGCTAAAATAGGAAATGATTTTAGTTTGACTGTACCATTTGAATAAGTATCGGCTAACTATGTGCCA
GCAGCCGCGGTAATACATAGGATGCAAGCGTTATCCGGATTTACTGGGCGTAAAACGAGCGCAGGCGGTT
TGTAAGTTTGGTATTAAATCTAGATGCTTAACGTCTAGCTGTATCAAAAACTGTAAACCTAGAGTGTAGT
AGGGAGTTGGGGAACTCCATGTGGAGCGGTAAAATGCGTAGATATATGGAAGAACACCGGTGGCGAAGGC
GCCAACTTGGACTATCACTGACGCTTAGGCTCGAAAGTGTGGGGAGCAAATAGGATTAGATACCCTAGTA
GTCCACACCGTAAACGATCATCATTAAATGTCGGCCCGAATGGGTCGGTGTTGTAGCTAACGCATTAAAT
GATGTGCCTGGGTAGTACATTCGCAAGAATGAAACTCAAACGGAATTGACGGGGACCCGCACAAGTGGTG
GAGCATGTTGCTTAATTTGACAATACACGTAGAACCTTACCTAGGTTTGACATCTATTGCGATGCTATAG
AAATATAGTTGAGGTTAACAATATGACAGGTGGTGCATGGTTGTCGTCAGCTCGTGTCGTGAGATGTTGG
GTTAAGTCCCGCAACGAGCGCAACCCCTTTCGTTAGTTACTTTTCTAGCGATACTGCTACCGCAAGGTAG
AGGAAGGTGGGGATGACGTCAAATCATCATGCCCCTTATATCTAGGGCTGCAAACGTGCTACAATGGCTA
ATACAAACTGCTGCAAAATCGTAAGATGAAGCGAAACAGAAAAAGTTAGTCTCAGTTCGGATAGAGGGCT
GCAATTCGTCCTCTTGAAGTTGGAATCACTAGTAATCGCAAATCGGACATGTCGCGGTGAATACGTTCTC
GGGTCTTGTACACACCGCCCGTCAAACTATGGGAGCTGGTAATATCTAAAACCGCAAAGCTAACCTTTTG

GAGGCATGCGTCTAGGGTAGGATCGGTGACTGGAGTTA
>239923993|gb|GQ179715.1|Ureaplasma|Uncultured Ureaplasma sp. clone VE3B04 16S ribosomal RNA gene, partial sequence
GATTAACGCTGGCGGCATGCCTAATACATGCAAATCGAACGAAGCCTTTTAGGCTTAGTGGTGAACGGGT
GAGTAACACGTATCCAATCTACCCTTAAGTTGGGGATAACTAGTCGAAAGATTAGCTAATACCGAATAAT
AACATCAATATCGCATGAGAAGATGTAGAAAGTCGCTCTTTGTGGCGACGCTTTTGGATGAGGGTGCGAC
GTATCAGATAGTTGGTGAGGTAATGGCTCACCAAGTCAATGACGCGTAGCTGTACTGAGAGGTAGAACAG
CCACAATGGGACTGAGACACGGCCCATACTCCTACGGGAGGCAGCAGTAGGGAATTTTTCACAATGGGCG
CAAGCCTTATGAAGCAATGCCGCGTGAACGATGAAGGTCTTATAGATTGTAAAGTTCTTTTATATGGGAA
GAAACGCTAAAATAGGAAATGATTTTAGTTTGACTGTACCATTTGAATAAGTATCGGCTAACTATGTGCC
AGCAGCCGCGGTAATACATAGGATGCAAGCGTTATCCGGATTTACTGGGCGTAAAACGAGCGCAGGCGGG
TTTGTAAGTTTGGTATTAAATCTAGATGCTTAACGTCTAGCTGTATCAAAAACTGTAAACCTAGAGTGTA
GTAGGGAGTTGGGGAACTCCATGTGGAGCGGTAAAATGCGTAGATATATGGAAGAACACCGGTGGCGAAG
GCGCCAACTTGGACTATCACTGACGCTTAGGCTCGAAAGTGTGGGGAGCAAATAGGATTAGATACCCTAG
TAGTCCACACCGCAAACGATCATCATTAAATGTCGGCCCGAATGGGCCGGTGTTGCAGCTAACGCATTAA
ATGATGTGCCTGGGTAGTACATTCGCAAGAATGAAACTCAAACGGAATTGACGGGGACCCGCACAAGTGG
TGGAGCATGTTGCTTAATTTGACAATACACGTAGAACCTTACCTAGGTTTGACATCTATTGCGATGCTAT
AGAAATATAGTTGAGGTTAACAATATGACAGGTGGTGCATGGTTGTCGTCAGCTCGTGTCGTGAGATGTT
GGGTTAAGTCCCGCAGCGAGCGCAACCCCTTTCGTTAGTTACTTTTCTAGCGATACTGCTACCGCAAGGT
AGAGGAAGGTGGGGATGACGTCAAATCATCATGCCCCTTGTATCTAGGGCTGCAAACGTGCTACAATGGC
TAATACAAACTGCTGCAAAATCGTAAGATGAAGCGAAACAGAAAAAGTTAGTCTCAGTTCGGATAGAGGG
CTGCAATTCGTCCTCTTGAAGTTGGAATCACTAGTAATCGCGAATCAGACATGTCGCGGTGAATACGTTC
TCGGGTCTTGTGCACACCGCCCGTCAAACTATGGGAGCTGGTAATATCTAAAACCGCAAAGCTAACCTTT
TGGAGGCATGCGTCTAGGGTAGGATCGGTGACTGGAGTTAAGTCGTAACAAGGTA
>52222187|gb|AY738698.1|Dialister|Uncultured Dialister sp. clone BV2-33 16S ribosomal RNA gene, partial sequence
TGGGGAATCTTCCGCAATGGGCGAAAGCCTGACGGAGCAACGCCGCGTGAGTGAAGACGGCCTTCGGGTT
GTAAAACTCTGTGATTCGGGACGAAAGATAAGTAGACGAATAATCTGCATAAGTGACGGTACCGAAAAAG
CAAGCCACGGCTAACTACGTGCCAGCAGCCGCGGTAATACGTAGGTGGCAAGCGTTGTCCGGAATTATTG
GGCGTAAAGCGCGCGCAGGCGGCTACTTAAGTCCATCTTAAAAGTGCGGGGCTTAACCCCGTGATGGGAT
GGAAACTGAGAAGCTGGAGTGTCGGAGAGGAAAGTGGAATTCCTAGTGTAGCGGTGAAATGCGTAGAGAT
TAGGAAGAACACCGGTGGCGAAGGCGACTTTCTGGACGACAACTGACGCTTAGGCGCGAAAGCGTGGGGA
GCAAACAGGATTAGATACCCTGGTAGTCCACGCCGTAAACGATGGATACTAGGTGTAGGAGGTATCGACC
CCTTCTGTGCCGGAGTTAACGCAATAAGTATCCCGCCTGGGAAGTACGATCGCAAGATTAAAACTCAAAG
GAATTGACGGGGGCCCGCACAAGCGGTGGAGTATGTGGTTTAATTCGACGCAACGCGAAGAACCTTACCA
GGTCTTGACATTGATCGCTATTTTCAGAAATGAGAAGTTCTCCTTCGGGAGACGAGAAAACAGGTGGTGC
ACGGCTGTCGTCAGCTCGTGTCGTGAGATGTTGGGTTAAGTCCCGCAACGAGCGCAACCCCTATCATTTG
TTGCCAGCACGCAAAGGTGGGAACTCAAATGAGACCGCCGCAGACAATGCGGAGGAAGGCGGGGACGACG
TCAAGTCATCATGCCCCTTATGACCTGGGCTACACACGTACTACAATGGGTGTCAACAAAGAGAAGCGAA
GGAGCGATCCGGAGCAAACCTCAAAAACACACCCCCAGTTCAGATCGCAGGCTGCAACTCGCCTGCGTGA
AGCAGGAATCGCTAGTAATCGCGGGTCAGCATACCGCGGTGAATACGTTCCCGGGCCT
>297038037|gb|HM344442.1|Dialister|Uncultured bacterium clone ncd1057a03c1 16S ribosomal RNA gene, partial sequence
GACGAACGCTGGCGGCGTGCTTAACACATGCAAGTCGAACGGGAAGACATGAAGAGCTTGCTCTTTATGA
AATCCAGTGGCAAACGGGTGAGTAACACGTAAACAACCTGCCTTCAGGATGGGGACAACAGACGGAAACG
ACTGCTAATACCGAATACGATTCTTGAGTCGCATGACACAAGAAAGAAAGGGTGGCCTCTACTTGTAAGC
TATCGCCTGGAGAGGGGTTTGCGTCCGATTAGGTAGTTGGTGAGGTAACGGCCCACCAAGCCGACGATCG
GTAGCCGGTCTGAGAGGATGAACGGCCACATTGGAACTGAGACACGGTCCAGACTCCTACGGGAGGCAGC
AGTGGGGAATCTTCCGCAATGGGCGAAAGCCTGACGGAGCAACGCCGCGTGAGTGAAGACGGCCTTCGGG
TTGTAAAACTCTGTGATTCGGGACGAAAGATAAGTAGACGAATAATCTGCATAAGTGACGGTACCGAAAA
AGCAAGCCACGGCTAACTACGTGCCAGCAGCCGCGGTAATACGTAGGTGGCAAGCGTTGTCCGGAATTAT
TGGGCGTAAAGCGCGCGCAGGCGGCTACTTAAGTCCATCTTAAAAGTGCGGGGCTTAACCCCGTGATGGG
ATGGAAACTGAGAAGCTGGAGTGTCGGAGAGGAAAGTGGAATTCCTAGTGTAGCGGTGAAATGCGTAGAG
ATTAGGAAGAACACCGGTGGCGAAGGCGACTTTCTGGACGACAACTGACGCTTAGGCGCGAAAGCGTGGG

GAGCAAACAGGATTAGATACCCTGGTAGTCCACGCCGTAAACGATGGATACTAGGTGTAGGAGGTATCGA
CCCCTTCTGTGCCGGAGTTAACGCAATAAGTATCCCGCCTGGGAAGTACGATCGCAAGATTAAAACTCAA
AGGAATTGACGGGGGCCCGCACAAGCGGTGGAGTATGTGGTTTAATTCGACGCAACGCGAAGAACCTTAC
CAGGTCTTGACATTGATCGCTATTTTCAGAAATGAGAAGTTCTCCTTCGGGAGACGAGAAAACAGGTGGT
GCACGGCTGTCGTCAGCTCGTGTCGTGAGATGTTGGGTTAAGTCCCGCAACGAGCGCAACCCCTATCATT
TGTTGCCAGCACGCAAAGGTGGGAACTCAAATGAGACCGCCGCAGACAATGCGGAGGAAGGCGGGGACGA
CGTCAAGTCATCATGCCCCTTATGACCTGGGCTACACACGTACTACAATGGGTGTCAACAAAGAGAAGCG
AAGGAGCGATCCGGAGCAAACCTCAAAAACACACCCCCAGTTCAGATCGCAGGCTGCAACTCGCCTGCGT
GAAGCAGGAATCGCTAGTAATCGCGGGTCAGCATACCGCGGTGAATACGTTCCCGGGCCT
>66878648|gb|AY958942.1|Dialister|Uncultured bacterium clone rRNA169 16S ribosomal RNA gene, partial sequence
GCCCTTAGAGTTTGATCCTGGCTCAGGACGAACGCTGGCGGCGTGCTTAACACATGCAAGTCGAACGGGA
AGACATGAAGAGCTTGCTCTTTATGAAATCCAATGGCAAACGGGTGAGTAACACGTAAACAACCTGCCTT
CAGGATGGGGACAACAGACGGAAACGACTGCTAATACCGAATACGATTCTTGAGTCGCATGACACAAGAA
AGAAAGGGTGGCCTCTACTTGTAAGCTATCGCCTGAAGAGGGGTTTGCGTCCGATTAGGTAGTTGGTGAG
GTAACGGCCCACTAAGCCGACGATCGGTAGCCGGTCTGAGAGGATGAACGGCCACATTGGAACTGAGACA
CGGTCCAGACTCCTACGGGAGGCAGCAGTGGGGAATCTTCCGCAATGGGCGAAAGCCTGACGGAGCAACG
CCGCGTGAGTGAAGACGGCCTTCGGGTTGTAAAGCTCTGTGATTCGGGACGAAAGATAAGTAGACGAATA
ATCTGCATAAGTGACGGTACCGAAAAAGCAAGCCACGGCTAACTACGTGCCAGCAGCCGCGGTAATACGT
AGGTGGCAAGCGTTGTCCGGAATTATTGGGCGTAAAGCGCGCGCAGGCGGCTACTTAAGTCCATCTTAAA
AGTGCGGGGCTTAACCCCGTGATGGAATGGAAACTGAGAAGCTGGAGTGTCGGAGAGGAAAGTGGAATTC
CTAGTGTAGCGGTGAAATGCGTAGAGATTAGGAAGAACACCGGTGGCGAAGGCGACTTTCTGGACGACAA
CTGACGCTTAGGCGCGAAAGCGTGGGGAGCAAACAGGATTAGATACCCTGGTAGTCCACGCCGTAAACGA
TGGATACTAGGTGTAGGAGGTATCGACCCCTTCTGTGCCGGAGTTAACGCAATAAGTATCCCGCCTGGGA
AGTACGATCGCAAGATTAAAACTCAAAGGAATTGACGGGGGCCCGCACAAGCGGTGGAGTATGTGGTTTA
ATTCGACGCAACGCGAAGAACCTTACCAGGTCTTGACATTGATCGCTATTTTCAGAAATGAGAAGTTCTC
CTTCGGGAGACGAGAAAACAGGTGGTGCACGGCTGTCGTCAGCTCGTGTCGTGAGATGTTGGGTTAAGTC
CCGCAACGAGCGCAACCCCTATCATTTGTTGCCAGCACGCAAAGGTGGGAACTCAAATGAGACCGCCGCA
GACAATGCGGAGGAAGGCGGGGACGACGTCAAGTCATCATGCCCCTTATGACCTGGGCTACACACGTACT
ACAATGGGTGTCAACAAAGAGAAGCGAAGGAGCGATCCGGAGCAAACCTCAAAAACACACCCCCAGTTCA
GATCGCAGGCTGCAACTCGCCTGCGTGAAGCAGGAATCGCTAGTAATCGCGGGTCAGCATACCGCGGTGA
ATACGTTCCCGGGCCTTGTACACACCGCCCGTCACACTATGAGAGTCGGAAACACCCGAAGCCGGTGAGG
TAACCGCAAGGAGCCAGCCGTCGAAGGTGGAGCTGATGATTGGAGTGAAGTCGTAACAAGGTAACCGTAA
AGGGC
>63146134|gb|AY995269.1|Dialister|Uncultured bacterium clone FX1B4-5 16S ribosomal RNA gene, partial sequence
AGCGGCGTGCTTACCATGCAAGTCGAACGGGAAGACATGAAGAGCTTGCTCTTTATGAAATCCAGTGGCA
AACGGGTGAGTAACACGTAAACAACCTGCCTTCAGGATGGGGACAACAGACGGAAACGACTGCTAATACC
GAGTACGATTCTTGAGTCGCATGACACAAGAAAGAAAGGGTGGCCTCTACTTGTAAGCTATCGCCTGGAG
AGGGGTTTGCGTCCGATTAGGTAGTTGGTGAGGTAACGGCCCACCAAGCCGACGATCGGTAGCCGGTCTG
AGAGGATGAACGGCCACATTGGAACTGAGACACGGTCCAGACTCCTACGGGAGGCAGCAGTGGGGAATCT
TCCGCAATGGGCGAAAGCCTGACGGAGCAACGCCGCGTGAGTGAAGACGGCCTTCGGGTTGTAAAACTCT
GTGATTCGGGACGAAAGATAAGTAGACGAATAATCTGCATAAGTGACGGTACCGAAAAAGCAAGCCACGG
CTAACTACGTGCCAGCAGCCGCGGTAATACGTAGGTGGCAAGCGTTGTCCGGAATTATTGGGCGTAAAGC
GCGCGCAGGCGGCTACTTAAGTCCATCTTAAAAGTGCGGGGCTTAACCCCGTGATGGGATGGAAACTGAG
AAGCTAGAGTGTCGGAGAGGAAAGTGGAATTCCTAGTGTAGCGGTGAAATGCGTAGAGATTAGGAAGAAC
ACCGGTGGCGAAGGCGACTTTCTGGACGACAACTGACGCTTAGGCGCGAAAGCGTGGGGAGCAAACAGGA
TTAGATACCCTGGTAGTCCACGCCGTAAACGATGGATACTAGGTGTAGGAGGTATCGACCCCTTCTGTGC
CGGAGTTAACGCAATAAGTATCCCGCCTGGGAAGTACGATCGCAAGATTAAAACTCAAAGGAATTGACGG
GGGCCCGCACAAGCGGTGGAGTATGTGGTTTAATTCGACGCAACGCGAAGAACCTTACCAGGTCTTGACA
TTGATCGCTATTTTCAGAAATGAGAAGTTCTCTTTCGAGAGACGAGAAAACAGGTGGTGCACGGCTGTCG
TCAGCTCGTGTCGTGAGATGTTGGGTTAAGTCCCGCAACGAGCGCAACCCCTATCATTTGTTGCCAGCAC
GCAAAGGTGGGAACTCAAATGAGACCGCCGCAGACAATGCGGAGGAAGGCGGGGACGACGTCAAGTCATC
ATGCCCCTTATGACCTGGGCTACACACGTACTACAATGGGTGTCAACAAAGAGAAGCGAAGGAGCGATCC

GGAGCAAACCTCAAAAACACACCCCCAGTTCAGATCGCAGGCTGCAACTCGCCTGCGTGAAGCAGGAATC
GCTAGTAATCGCGGGTCAGCATACCCGCCGGTATCT
>52222185|gb|AY738696.1|Aerococcus|Uncultured Aerococcus sp. clone 141-b
19 16S ribosomal RNA gene, partial sequence
TAGGGAATCTTCCGCAATGGACGCAAGTCTGACGGAGCAACGCCGCGTGAGTGAAGAAGGTTTTCGGATC
GTAAAACTCTGTTGTAAGAGAAGAACAAATTGTAGAGTAACTGCTACAGTCTTGACGGTATCTTACCAGA
AAGCCACGGCTAACTACGTGCCAGCAGCCGCGGTAATACGTAGGTGGCAAGCGTTGTCCGGATTTATTGG
GCGTAAAGGGGGCGCAGGCTGCTTCTTAAGTCTGATGTGAAAGCCCACGGCTTAACCGTGGAAGTGCATT
GGAAACTGGGAAGCTTGAGTACAGAAGAGGAAAGTGGAACTCCATGTGTAGCGGTGGAATGCGTAGATAT
ATGGAAGAACACCAGTGGCGAAAGCGACTTTCTGGTCTGTCACTGACGCTGAGGCCCGAAAGCGTGGGTA
GCAAACAGGATTAGATACCCTGGTAGTCCACGCCGTAAACGATGAGCGCTAGGTGTTGGAGGGTTTCCAC
CCTTCAGTGCCGCAGCTAACGCATTAAGCGCTCCGCCTGGGGAGTACGACCGCAAGGTTGAAACTCAAAG
GAATTGACGGGGACCCGCACAAGCGGTGGAGCATGTGGTTTAATTCGAAGCAACGCGAAGAACCTTACCA
AGTCTTGACATCCTTTGACCACTCTAGAGATAGAGCTTTCCCTTCGGGGACAAAGTGACAGGTGGTGCAT
GGTTGTCGTCAGCTCGTGTCGTGAGATGTTGGGTTAAGTCCCGCAACGAGCGCAACCCCTATTGTTAGTT
GCCAGCATTGAGTTGGGCACTCTAGCAAGACTGCCGGTGACAAACCGGAGGAAGGCGGGGATGACGTCAA
ATCATCATGCCCCTTATGACTTGGGCTACACACGTGCTACAATGGATGGTACAACGGGCAGCGAGCTCGC
GAGAGTCAGCGAATCCCTTAAAGCCATTCTCAGTTCGGATTGTAGTCTGCAACTCGACTACATGAAGCCG
GAATCGCTAGTAATCGCGGATCAGCACGCCGCGGTGAATACGTTCCCGGGTCT
>66878879|gb|AY959173.1|Aerococcus|Uncultured bacterium clone rRNA400 16S
ribosomal RNA gene, partial sequence
CGCCCTTAGAGTTTGATCCTGGCTCAGGACGAACGCTGGCGGCGTGCCTAATACATGCAAGTCGAGCGAA
CTTTGAAAGTGCTTGCGCTTTCAAAGTTAGCGGCGGACGGGTGAGTAACACGTAAGGAACCTACCGATAA
GCGGGGGACAACATCCGGAAACGGGTGCTAATACCGCATAGGAAGTTTGTTCGCATGAACAAACCTAGAA
AGATGGCTCTGCTATCACTTATCGATGGCCTTGCGGTGCATTAACTAGTTGGCGAGGTAACGGCTCACCA
AGGTGATGATGCATAGCCGACCTGAGAGGGTAATCGGCCACATTGGGACTGAGACACGGCCCAAACTCCT
ACGGGAGGCAGCAGTAGGGAATCTTCCGCAATGGACGCAAGTCTGACGGAGCAACGCCGCGTGAGTGAAG
AAGGTTTTCGGATCGTAAAACTCTGTTGTAAGAGAAGAACAAATTGTAGAGTAACTGCTACAGTCTTGAC
GGTATCTTACCAGAAAGCCACGGCTAACTACGTGCCAGCAGCCGCGGTAATACGTAGGTGGCAAGCGTTG
TCCGGATTTATTGGGCGTAAAGGGGGCGCAGGCTGCTTCTTAAGTCTGATGTGAAAGCCCACGGCTTAAC
CGTGGAAGTGCATTGGAAACTGGGAAGCTTGAGTACAGAAGAGGAAAGTGGAACTCCATGTGTAGCGGTG
GAATGCGTAGATATATGGAAGAACACCAGTGGCGAAAGCGACTTTCTGGTCTGTCACTGACGCTGAGGCC
CGAAAGCGTGGGTAGCAAACAGGATTAGATACCCTGGTAGTCCACGCCGTAAACGATGAGCGCTAGGTGT
TGGAGGGTTTCCACCCTTCAGTGCCGCAGCTAACGCATTAAGCGCTCCGCCTGGGGAGTACGACCGCAAG
GTTGAAACTCAAAGGAATTGACGGGGACCCGCACAAGCGGTGGAGCATGTGGTTTAATTCGAAGCAACGC
GAAGAACCTTACCAAGTCTTGACATCCTTTGACCACTCTAGAGATAGAGCTTTCCCTTCGGGGACAAAGT
GACAGGTGGTGCATGGTTGTCGTCAGCTCGTGTCGTGAGATGTTGGGTTAAGTCCCGCAACGAGCGCAAC
CCCTATTGTTAGTTGCCAGCATTGAGTTGGGCACTCTAGCAAGACTGCCGGTGACAAACCGGAGGAAGGC
GGGGATGACGTCAAATCATCATGCCCCTTATGACTTGGGCTACACACGTGCTACAATGGATGGTACAACG
GGCAGCGAGCTCGCGAGAGTCAGCGAATCCCTTAAAGCCATTCTCAGTTCGGATTGTAGTCTGCAACTCG
ACTACATGAAGCCGGAATCGCTAGTAATCGCGGATCAGCACGCCGCGGTGAATACGTTCCCGGGTCTTGT
ACACACCGCCCGTCACACCACGAGAGTTTGTAACACCCGAAGTCGGTGAGGTAACCTTTTGGAGCCAGCC
GCCGAAGGTGGGACAAGTGATTGGGGTGAAGTCGTAACAAGGTAACCGTAAAGGGCG
>239924009|gb|GQ179731.1|Aerococcus|Uncultured Aerococcus sp. clone
VE4G11 16S ribosomal RNA gene, partial sequence
GACGAACGCTGGCGGCGTGCCTAATACATGCAAGTCGAGCGAACAGAGAAAGTGCTTGCACTTTCAAAGT
TAGCGGCGGACGGGTGAGTAACACGTAAGGAACCTACCGATAAGCGGGGGACAACATCCGGAAACGGGCG
CTAATACCGCATAGGAAGTTTGTTCGCATGAACAAACTTAGAAAGATGGCTCTGCTATCACTTATCGATG
GCCTTGCGGTGCATTAACTAGTTGGCGAGGTAACGGCTCACCAAGGTGATGATGCATAGCCGACCTGAGA
GGGTAATCGGCCACATTGGGACTGAGACACGGCCCAAACTCCTACGGGAGGCAGCAGTAGGGAATCTTCC
GCAATGGACGCAAGTCTGACGGAGCAACGCCGCGTGAGTGAAGAAGGTTTTCGGATCGTAAAACTCTGTT
GTAAGAGAAGAACAAATTGTAGAGTAACTGCTACAGTCTTGACGGTATCTTACCAGAAAGCCACGGCTAA
CTACGTGCCAGCAGCCGCGGTAATACGTAGGTGGCAAGCGTTGTCCGGATTTATTGGGCGTAAAGGGGGC
GCAGGCTGCTTCTTAAGTCTGATGTGAAAGCCCACGGCTTAACCGTGGAAGTGCATTGGAAACTGGGAAG

CTTGAGTACAGAAGAGGAAAGTGGAACTCCATGTGTAGCGGTGGAATGCGTAGATATATGGAAGAACACC
AGTGGCGAAAGCGACTTTCTGGTCTGTCACTGACGCTGAGGCCCGAAAGCGTGGGTAGCAAACAGGATTA
GATACCCTGGTAGTCCACGCCGTAAACGATGAGCGCTAGGTGTTGGAGGGTTTCCACCCTTCAGTGCCGC
AGCTAACGCATTAAGCGCTCCGCCTGGGGAGTACGACCGCAAGGTTGAAACTCAAAGGAATTGACGGGGA
CCCGCACAAGCGGTGGAGCATGTGGTTTAATTCGAAGCAACGCGAAGAACCTTACCAAGTCTTGACATCC
TTTGACCACTCTAGAGATAGAGCTTTCCCTTCGGGGACAAAGTGACAGGTGGTGCATGGTTGTCGTCAGC
TCGTGTCGTGAGATGTTGGGTTAAGTCCCGCAACGAGCGCAACCCCTATTGTTAGTTGCCAGCATTGAGT
TGGGCACTCTAGCAAGACTGCCGGTGATAAACCGGAGGAAGGCGGGGATGACGTCAAATCATCATGCCCC
TTATGACTTGGGCTACACACGTGCTACAATGGATGGTACAACGGGCAGCGAGCTCGCGAGAGTCAGCGAA
TCCCTTAAAGCCATTCTCAGTTCGGATTGTAGTCTGCAACTCGACTACATGAAGCCGGAATCGCTAGTAA
TCGCGGATCAGCACGCCGCGGTGAATACGTTCCCGGGTCTTGTACACACCGCCCGTCACACCACGAGAGT
TTGTAACACCCGAAGTCGGTGAGGTAACCTTTTGGAGCCAACCGCCGAAGGTGGGACAAGTGATTGGGGT
GAATGTCGTAACAAGGTA
>5701789|emb|Y17318.1|Aerococcus|Aerococcus sp. CCUG28826 16S rRNA gene
GACGAACGCTGGCGGCGTGCCTAATACATGCAAGTCGAGCGAACAGAGAAAGTGCTTGCACTTTCAAAGT
TAGCGGCGGACGGGTGAGTAACACGTAAGGAACCTACCGATAAGCGGGGGACAACATCCGGAAACGGGTG
CTAATACCGCATAGGAAGTTTGTTCGCATGAACAAACTTAGAAAGATGGCTCTGCTATCACTTATCGATG
GCCTTGCGGTGCATTAACTAGTTGGCGAGGTAACGGCTCACCAAGGTGATGATGCATAGCCGACCTGAGA
GGGTAATCGGCCACATTGGGACTGAGACACGGCCCAAACTCCTACGGGAGGCAGCAGTAGGGAATCTTCC
GCAATGGACGCAAGTCTGACGGAGCAACGCCGCGTGAGTGAAGAAGGTTTTCGGATCGTAAAACTCTGTT
GTAAGAGAAGAACAAATTGTAGAGTAACTGCTACAGTCTTGACGGTATCTTACCAGAAAGCCACGGCTAA
CTACGTGCCAGCAGCCGCGGTAATACGTAGGTGGCAAGCGTTGTCCGGATTTATTGGGCGTAAAGGGGGC
GCAGGCTGCTTCTTAAGTCTGATGTGAAAGCCCACGGCTTAACCGTGGAAGTGCATTGGAAACTGGGAAG
CTTGAGTACAGAAGAGGAAAGTGGAACTCCATGTGTAGCGGTGGAATGCGTAGATATATGGAAGAACACC
AGTGGCGAAAGCGACTTTCTGGTCTGTCACTGACGCTGAGGCCCGAAAGCGTGGGTAGCAAACAGGATTA
GATACCCTGGTAGTCCACGCCGTAAACGATGAGCGCTAGGTGTTGGAGGGTTTCCACCCTTCAGTGCCGC
AGCTAACGCATTAAGCGCTCCGCCTGGGGAGTACGACCGCAAGGTTGAAACTCAAAGGAATTGACGGGGA
CCCGCACAAGCGGTGGAGCATGTGGTTTAATTCGAAGCAACGCGAAGAACCTTACCAAGTCTTGACATCC
TTTGACCACTCTGGAGATAGAGTTTTCCCTTCGGGGACAAAGTGACAGGTGGTGCATGGTTGTCGTCAGC
TCGTGTCGTGAGATGTTGGGTTAAGTCCCGCAACGAGCGCAACCCCTATTGTTAGTTGCCAGCATTGAGT
TGGGCACTCTAGCAAGACTGCCGGTGACAAACCGGAGGAAGGCGGGGATGACGTCAAATCATCATGCCCC
TTATGACTTGGGCTACACACGTGCTACAATGGATGGTACAACGGGCAGCGAGCTCGCGAGAGTCAGCGAA
TCCCTTAAAGCCATTCTCAGTTCGGATTGTAGTCTGCAACTCGACTACATGAAGCCGGAATCGCTAGTAA
TCGCGGATCAGCACGCCGCGGTGAATACGTTCCCGGGTCTTGTACACACCGCCCGTCACACCACGAGAGT
TTGTAACACCCGAAGTCGGTGAGGTAACCTTTTGGAGCCAGCCGCCGAAGGTGGGACAAGTGATTGGGGT
GAAGTCGTAACAAGGTAGCCGTAGGAGAACC
>63146102|gb|AY995237.1|Aerococcus|Uncultured Aerococcus sp. clone
FX44W16 16S ribosomal RNA gene, partial sequence
AGTGGCGGCTTACTTATAATGCAAGTCGAGCGACAGAGAAAGTGCTTGCACTTTCAAAGTTAGCGGCGGA
CGGGTGAGTAACACGTAAGGAACCTACCGATAAGCGGGGGACAACATCCGGAAACGGGTGCTAATACCGC
ATAGGAAGTTTGTTCGCATGAACAAACTTAAAAAGATGGGTCTGCTATCACTTATCGATGGCCTTGCGGT
GCATTAACTAGTTGGCGAGGTAACGGCTCACCAAGGTGATGATGCATAGCCGACCTGAGAGGGTAATCGG
CCACATTGGGACTGAGACACGGCCCAAACTCCTACGGGAGGCAGCAGTAGGGAATCTTCCGCAATGGACG
CAAGTCTGACGGAGCAACGCCGCGTGAGTGAAGAAGGTTTTCGGATCGTAAAACTCTGTTGTAAGAGAAG
AACAAATTGTAGAGTAACTGCTACAGTCTTGACGGTATCTTACCAGAAAGCCACGGCTAACTACGTGCCA
GCAGCCGCGGTAATACGTATGTGGCAAGCGTTGTCCGGATTTATTGGGCGTAAAGGGGGCGCAGGCTGCT
TCTTAAGTCTGATGTGAAAGCCCACGGCTTAACCCGTGGAAGTGCATTGGAAACTGGGAAGCTTGAGTAC
AGAAGAGGAAAGTGGAACTCCATGTGTAGCGGTGGAATGCGTAGATATATGGAAGAACACCAGTGGCGAA
AGCGACTTTCTGGTCTGTCACTGACGCTGAGGCCTGAAAGCGTGGGTAGCAAACAGGATTAGATACCCTG
GTAGTCCACGCCGTAAACGATGAGCGCTAGGTGTTGGAGGGTTTCCACCCTTCAGTGCCGCAGCTAACGC
ATTAAGCGCTCCGCCTGGGGAGTACGACCGCAAGGTTGAAACTCAAAGGAATTGACGGGGACCCGCACAA
GCGGTGGAGCATGTGGTTTAATTCGAAGCAACGCGAAGAACCTTACCAAGTCTTGACATCCTTTGACCAC
TCTAGAGATAGAGCTTTCCCTTCGGGGACAAAGTGACAGGTGGTGCATGGTTGTCGTCAGCTCGTGTCGT
GAGGTGTTGGGTTAAGTCCCGCAACGAGCGCAACCCCTATTGTTAGTTGCCAGCATTGAGTTGGGCACTC

TAGCAAGACTGCCGGTGACAAACCGGAGGAAGGCGGGGATGACGTCAAATCATCATGCCCCTTATGACTT
GGGCTACACACGTGCTACAATGGATGGTACAACGGGCAGCGAGCTCGCGAGAGTCAGCGAATCCCTTAAA
GCCATTCTCAGTTCGGATTGTAGTCTGCAACTCGACTACATGAAGCCGGAATCGCTAGTAATCGCGATCA
GCACGAAAACCGTATTTCCCCG
>52222183|gb|AY738694.1|Anaerococcus|Uncultured Anaerococcus sp. clone 123-f2 5 16S ribosomal RNA gene, partial sequence
TGGGGAATTTTGCACAATGGGGGAAACCCTGATGCAGCGACGCCGCGTGATTTAGAAGGCCTTCGGGTTG
TAAAAATCTTTTGTATGGGAAGAAAATGACAGTACCATACGAATAAGGACCGGCTAATTACGTGCCAGCA
GCCGCGGTAATACGTAAGGTCCGAGCGTTGTCCGGAATCATTGGGCGTAAAGGGTACGTAGGCGGATAAG
CAAGTTAGAAGTGAAATCCTATAGCTCAACTATAGTAAGCTTTTAAAACTGCTCATCTTGAGGTATGGAA
GGGAAAGTGGAATTCCTAGTGTAGCGGTGAAATGCGCAGATATTAGGAGGAATACCGGTGGCGAAGGCGA
CTTTCTGGCCATAAACTGACGCTGAGGTACGAAAGCGTGGGTAGCAAACAGGATTAGATACCCTGGTAGT
CCACGCCGTAAACGATGAGTGTTAGGTGTCTGGAATAATCTGGGTGCCGCAGCTAACGCAATAAACACTC
CGCCTGGGGAGTACGCACGCAAGTGTGAAACTCAAAGGAATTGACGGGGACCCGCACAAGCAGCGGAGCA
TGTGGTTTAATTCGACGCAACGCGAAGAACCTTACCAAGTCTTGACATATTACGGAGGGAATTAGAGATA
GTTCCTTACTTCTTCGGAAGACTGTAATACAGGTGGTGCATGGTTGTCGTCAGCTCGTGTCGTGAGATGT
TGGGTTAAGTCCCATAACGAGCGCAACCCCTATATTTAGTTACCATCATTAAGTTGGGGACTCTAGATAT
ACTGCCGGTGATAAACCGGAGGAAGGTGGGGATGACGTCAAATCATCATGCCCTTTATGACTTGGGCTAC
ACACGTGCTACAATGGCAGGTACACAGGGAAGCGAGACTGCGAAGTTAAGCAAAACTCAAAAAGCCTGTC
CCAGTTCGGATTGCACTCTGCAACTCGAGTGCATGAAGTTGGAGTTGCTAGTAATCGTGGATCAGAATGC
CGCGGTGAATGCGTTCCCGGGTCT
>257480662|gb|GQ422749.1|Anaerococcus|Anaerococcus tetradius strain F0127 16S ribosomal RNA gene, partial sequence
AGAGTTTGATCCTGGCTCAGGATTAACGCTGGCGGCGTGCATAACACATGCAAGTCGAACGATGAAGATA
TTTAGATTCCTTCGGGATGAAAAATATTGGATTAGTGGCGGACGGGTGAGTAACGCGTGAGTAACCTGCC
TTACACAAGGGGATAGCCGTTGGAAACGACGAATAATACCCTATGACATAAACTCTTCGCATGAGGAGCT
TATCAAAGATTTATCGGTGTAAGATGGACTCGCGTCTGATTAGCTAGTTGGTGAGGTAACGGCCCACCAA
GGCAACGATCAGTAGCCGGCTTGAGAGAGTGTACGGCCACATTGGGACTGAGACACGGCCCAGACTCCTA
CGGGAGGCAGCAGTGGGGAATTTTGCACAATGGGGGAAACCCTGATGCAGCGACGCCGCGTGATTTAGAA
GGCCTTCGGGTTGTAAAAATCTTTTGTATGGGAAGAAAATGACAGTACCATACGAATAAGGACCGGCTAA
TTACGTGCCAGCAGCCGCGGTAATACGTAAGGTCCGAGCGTTGTCCGGAATCATTGGGCGTAAAGGGTAC
GTAGGCGGATAAGCAAGTTAGAAGTGAAATCCTATAGCTCAACTATAGTAAGCTTTTAAAACTGCTCATC
TTGAGGTATGGAAGGGAAAGTGGAATTCCTAGTGTAGCGGTGAAATGCGCAGATATTAGGAGGAATACCG
GTGGCGAAGGCGACTTTCTGGCCATAAACTGACGCTGAGGTACGAAAGCGTGGGTAGCAAACAGGATTAG
ATACCCTGGTAGTCCACGCCGTAAACGATGAGTGTTAGGTGTCTGGAATAATCTGGGTGCCGCAGCTAAC
GCAATAAACACTCCGCCTGGGGAGTACGCACGCAAGTGTGAAACTCAAAGGAATTGACGGGGACCCGCAC
AAGCAGCGGAGCATGTGGTTTAATTCGACGCAACGCGAAGAACCTTACCAAGTCTTGACATATTACGGAG
GGAATTAGAGATAGTTCCTTACTTCTTCGGAAGACTGTAATACAGGTGGTGCATGGTTGTCGTCAGCTCG
TGTCGTGAGATGTTGGGTTAAGTCCCATAACGAGCGCAACCCCTATATTTAGTTACCATCATTAAGTTGG
GGACTCTAGATATACTGCCGGTGATAAACCGGAGGAAGGTGGGGATGACGTCAAATCATCATGCCCTTTA
TGACTTGGGCTACACACGTGCTACAATGGCAGGTACACAGGGAAGCGAGACTGCGAAGTTAAGCAAAACT
CAAAAAGCCTGTCCCAGTTCGGATTGCACTCTGCAACTCGAGTGCATGAAGTTGGAGTTGCTAGTAATCG
TGGATCAGAATGCCGCGGTGAATGCGTTCCCGGGTCTTGTACACACCGCCCGTCACACCATGGAAGTTGG
CAATACCCGAAGCCTGTGAGCTAACCATTAGGAAGCAGCAGTCGAAGGTAGGGTCAGTAACTGGGGTGAA
GTCGTAACAAGGTAGCCGTATCGGAAGGTGC
>66878532|gb|AY958826.1|Anaerococcus|Uncultured bacterium clone rRNA053 16S ribosomal RNA gene, partial sequence
CCTTAGAGTTTGATCCTGGCTCAGGATAAACGCTGGCGGCGTGCATAACACATGCAAGTCGAACGATGAA
ACTTAACTGATTTCTTCGGAATGATTTTAAGTGGATTAGTGGCGGACGGGTGAGTAACGCGTGAGTAACC
TGCCTTGCACAAGGGGATAGCCGTTGGAAACGACGAATAATACCCTATGATATAAACTCTTCGCATGAAG
GGCTTATCAAAGATTTATCGGTGTAAGATGGACTCGCGTCTGATTAGCTAGTTGGTGGGATAAAAGCCTA
CCAAGGCAACGATCAGTAGCCGGCTTGAGAGAGTGTACGGCCACATTGGGACTGAGACACGGCCCAGACT
CCTACGGGAGGCAGCAGTGGGGAATTTTGCACAATGGGGGCAACCCTGATGCAGCGACGCCGCGTGATTT
AGAAGGCCTTCGGGTTGTAAAAATCTTTTGTATGGGAAGAAAATGACAGTACCATACGAATAAGGACCGG

CTAATTACGTGCCAGCAGCCGCGGTAATACGTAAGGTCCGAGCGTTGTCCGGAATCATTGGGCGTAAAGG
GTACGTAGGCGGATAAGCAAGTTAGAAGTGAAATCCTATAGCTCAACTATAGTAAGCTTTTAAAACTGCT
CATCTTGAGGTATGGAAGGGAAAGTGGAATTCCTAGTGTAGCGGTGAAATGCGCAGATATTAGGAGGAAT
ACCGGTGGCGAAGGCGACTTTCTGGCCATAAACTGACGCTGAGGTACGAAAGCGTGGGTAGCAAACAGGA
TTAGATACCCTGGTAGTCCACGCCGTAAACGATGAGTGTTAGGTGTCTGGAATAATCTGGGTGCCGCAGC
TAACGCAATAAACACTCCGCCTGGGGAGTACGCACGCAAGTGTGAAACTCAAAGGAATTGACGGGGACCC
GCACAAGCAGCGGAGCATGTGGTTTAATTCGACGCAACGCGAAGAACCTTACCAAGTCTTGACATATTAC
GGAGGGAATTAGAGATAGTTCCTTACTTCTTCGGAAGACTGTAATACAGGTGGTGCATGGTTGTCGTCAG
CTCGTGTCGTGAGATGTTGGGTTAAGTCCCATAACGAGCGCAACCCCTATATTTAGTTACCATCATTAAG
TTGGGGACTCTAGATATACTGCCGGTGATAAACCGGAGGAAGGTGGGGATGACGTCAAATCATCATGCCC
TTTATGACTTGGGCTACACACGTGCTACAATGGCAGGTACACAGGGAAGCGAGACTGCGAAGTTAAGCAA
AACTCAAAAAGCCTGTCCCAGTTCGGATTGCACTCTGCAACTCGAGTGCATGAAGTTGGAGTTGCTAGTA
ATCGTGGATCAGAATGCCGCGGTGAATGCGTTCCCGGGTCTTGTACACACCGCCCGTCACACCATGGAAG
TTGGCAATACCCGAAGCCTGTGAGCTAACCATTAGGAAGCAGCAGTCGAAGGT
>52222181|gb|AY738692.1|Peptoniphilus|Uncultured Peptoniphilus sp. clone 123-b 35 16S ribosomal RNA gene, partial sequence
TGGGGAATATTGCACAATGGGGGAAACCCTGATGCAGCGACGCCGCGTGAGCGAAGAAGGCCTTCGGGTC
GTAAAGCTCTTTTATATGGGAAGATAATGACGGTACCATAAGAAAAAGCCCCGGCTAACTACGTGCCAGC
AGCCGCGGTAATACGTAGGGGGCTAGCGTTGTCCGGAATCACTGGGCGTAAAGGGTTCGCAGGCGGCAAT
GCAAGTCAGATGTAAAAGGCAAAGGCTCAACCTTTGTAAGCATCTGAAACTGTATAGCTTGAGAAGTGTA
GAGGCAAGTGGAATTTTTAGTGTAGCGGTGAAATGCGTAGATATTAAAAAGAATACCGGTGGCGAAGGCG
ACTTGCTGGGCACAATCTGACGCTGAGGAACGAAAGCGTGGGGAGCAAACAGGATTAGATACCCTGGTAG
TCCACGCCGTAAACGATGAGTGCTAGGTGTCGGTATAAATCGGTGCCGCAGTTAACACAATAAGCACTCC
GCCTGGGGAGTACGTGCGCAAGCATGAAACTCAAAGGAATTGACGGGGACCCGCACAAGCAGCGGAGCAT
GTGGTTTAATTCGAAGCAACGCGAAGAACCTTACCAGGGCTTGACATATAAGAGACGAACTTAGAGATAA
GTTTTCTTCTTCGGAAGCCCTTATACAGGTGGTGCATGGTTGTCGTCAGCTCGTGTCGTGAGATGTTGGG
TTAAGTCCCGCAACGAGCGCAACCCTTATTACTAGTTACCAGCATTTCGGATGGGGACTCTAGAAAGACT
GCCGATGATAAATCGGAGGAAGGTGGGGATGACGTCAAATCATCATGCCCTATATGCCCTGGGCAACACA
CGTGCTACAATGGCCGTAACAAAGAGAAGCGAAATCGCAAGGTCAAGCAAACCTCAAAAAGACGGTCTCA
GTTCGGATTGTTCTCTGCAACTCGAGAACATGAAGTCGGAGTTGCTAGTAATCGCAGATCAGAATGCTGC
GGTGAATGCGTTCCCGGGTCT
>257480663|gb|GQ422750.1|Peptoniphilus|Peptoniphilus sp. oral taxon 836 strain F0141 16S ribosomal RNA gene, partial sequence
GAGTTTGATCCTGGCTCAGGACGAACGCTGGCGGCGCGCTTAACACATGCAAGTCGAGCGATGAAATCTT
AACAGAACCCTTCGGGGCGAAGATAAGACGGATTAGCGGCGGACGGGTGAGTAACACGTGAGTAACCTGC
CTTTTACACAGGGATAGCCTCGGGAAACCGGGATTAAAACCTGATGAAACTATCAAATCACATGATTAAG
AAAGTTAAAACTCCGGTGGTAAAAGATGGACTCGCGTCCCATTAGCTAGTTGGTGAAGGTAACGGCCCAC
CAAGGCAACGATGGGTAGCCGGCCTGAGAGGGTGAACGGCCACATTGGAACTGAGAAACGGTCCAAACTC
CTACGGGAGGCAGCAGTGGGGAATATTGCACAATGGGGGAAACCCTGATGCAGCGACGCCGCGTGAGCGA
AGAAGGCCTTCGGGTCGTAAAGCTCTTTTATATGGGAAGATAATGACGGTACCATAAGAAAAAGCCCCGG
CTAACTACGTGCCAGCAGCCGCGGTAATACGTAGGGGGCTAGCGTTGTCCGGAATCACTGGGCGTAAAGG
GTTCGCAGGCGGCAATGCAAGTCAGATGTAAAAGGCAAAGGCTCAACCTTTGTAAGCATCTGAAACTGTA
TAGCTTGAGAAGTGTAGAGGCAAGTGGAATTTTTAGTGTAGCGGTGAAATGCGTAGATATTAAAAAGAAT
ACCGGTGGCGAAGGCGACTTGCTGGGCACAATCTGACGCTGAGGAACGAAAGCGTGGGGAGCAAACAGGA
TTAGATACCCTGGTAGTCCACGCCGTAAACGATGAGTGCTAGGTGTCGGTATAAATCGGTGCCGCAGTTA
ACACAATAAGCACTCCGCCTGGGGAGTACGTGCGCAAGCATGAAACTCAAAGGAATTGACGGGGACCCGC
ACAAGCAGCGGAGCATGTGGTTTAATTCGAAGCAACGCGAAGAACCTTACCAGGGCTTGACATATAAGAG
ACGAACTTAGAGATAAGTTTTCTTCTTCGGAAGCCCTTATACAGGTGGTGCATGGTTGTCGTCAGCTCGT
GTCGTGAGATGTTGGGTTAAGTCCCGCAACGAGCGCAACCCTTATTACTAGTTACCAGCATTTCGGATGG
GGACTCTAGAAAGACTGCCGATGATAAATCGGAGGAAGGTGGGGATGACGTCAAATCATCATGCCCTATA
TGCCCTGGGCAACACACGTGCTACAATGGCCGTAACAAAGAGAAGCGAAATCGCAAGGTCAAGCAAACCT
CAAAAAGACGGTCTCAGTTCGGATTGTTCTCTGCAACTCGAGAACATGAAGTCGGAGTTGCTAGTAATCG
CAGATCAGAATGCTGCGGTGAATGCGTTCCCGGGTCTTGTACACACCGCCCGTCACACCATGGGAGCTTG
TAATACCCGAAGCCTGTGAGCTAACCGCAAGGAAGCAGCAGTCGAAGGTAGGACAAGTGACTGGGGTGAA

GTCGTAACAAGGTAGCCGTATCGGAAGGTGCGG
>29825720|gb|AF542230.1|Peptoniphilus|Peptoniphilus lacrimalis strain CCUG 31350 16S ribosomal RNA gene, partial sequence
TGCAAGTCGAGCGATGAAATCTTAACAGAACCCTTCGGGGTGAAGATAAGACGGATTAGCGGCGGACGGG
TGAGTAACACGTGAGTAACCTGCCTTTTACACAGGGATAGCCTCGGGAAACCGGGATTAAAACCTGATGA
AACTATCAAATCACATGATTAAGAAAGTTAAAACTCCGGTGGTAAAAGATGGACTCGCGTCCCATTAGCT
AGTTGGTGAAGGTAACGGCCCACCAAGGCAACGATGGGTAGCCGGCCTGAGAGGGTGAACGGCCACATTG
GAACTGAGAAACGGTCCAAACTCCTACGGGAGGCAGCAGTGGGGAATATTGCACAATGGGGGAAACCCTG
ATGCAGCGACGCCGCGTGAGCGAAGAAGGCCTTCGGGTCGTAAAGCTCTTTTATATGGGAAGATAATGAC
GGTACCATAAGAAAAAGCCCCGGCTAACTACGTGCCAGCAGCCGCGGTAATACGTAGGGGGCTAGCGTTG
TCCGGAATCACTGGGCGTAAAGGGTTCGCAGGCGGCAATGCAAGTCAGATGTAAAAGGCAAAGGCTCAAC
CTTTGTAAGCATCTGAAACTGTATAGCTTGAGAAGTGTAGAGGCAAGTGGAATTTTTAGTGTAGCGGTGA
AATGCGTAGATATTAAAAAGAATACCGGTGGCGAAGGCGACTTGCTGGGCACAATCTGACGCTGAGGAAC
GAAAGCGTGGGGAGCAAACAGGATTAGATACCCTGGTAGTCCACGCCGTAAACGATGAGTGCTAGGTGTC
GGTATAAATCGGTGCCGCAGTTAACACAATAAGCACTCCGCCTGGGGAGTACGTGCGCAAGCATGAAACT
CAAAGGAATTGACGGGGACCCGCACAAGCAGCGGAGCATGTGGTTTAATTCGAAGCAACGCGAAGAACCT
TACCAGGGCTTGACATATAAGAGACGAACTTAGAGATAAGTTTTCTTCTTCGGAAGCCCTTATACAGGTG
GTGCATGGTTGTCGTCAGCTCGTGTCGTGAGATGTTGGGTTAAGTCCCGCAACGAGCGCAACCCTTATTA
CTAGTTACCAGCATTTCGGATGGGGACTCTAGAAAGACTGCCGATGATAAATCGGAGGAAGGTGGGGATG
ACGTCAAATCATCATGCCCTATATGCCCTGGGCAACACACGTGCTACAATGGCCGTAACAAAGAGAAGCG
AAATCGCAAGGTCAAGCAAACCTCAAAAAGACGGTCTCAGTTCGGATTGTTCTCTGCAACTCGAGAACAT
GAAGTCGGAGTTGCTAGTAATCGCAGATCAGAATGCTGCGGTGAATGCGTTCCCGGGTCTTGTACACACC
GCCCGTCACACCATGGGAGCTTGTAATACCCGAAGGCCTGTGAGCTAACCGCAAGGAAGCAGCAGTCGAA
GGTAGG
>52222173|gb|AY738684.1|M.mulieris|Uncultured Mobiluncus sp. clone 123-f 85 16S ribosomal RNA gene, partial sequence
TGGGGAATATTGCACAATGGACGGAAGTTTGATGCAGCGACGCCGCGTGGAGGGTGTAGGCCTTCGGGTT
GTGAACTCCTTTTTCTCGTGAAAAAGGCATGCTTTTGGGTGTGTTGATGGTAGCGGGGGAAGAAGCGCCG
GCTAACTACGTGCCAGCAGCCGCGGTAATACGTAGGGCGCGAGCGTTGTCCGGATTTATTGGGCGTAAAG
AGCTCGTAGGTGGTTCGTCGCGTCTGTCGTGAAAGCCAGCAGCTTAACTGTTGGTCTGCGGTGGGTACGG
GCGGGCTTGAGTGCGGTAGGGGTGACTGGAATTCCTGGTGTAGCGGTGGAATGCGCAGATATCAGGAGGA
ACACCGATGGCGAAGGCAGGTCACTGGGCCGTTACTGACGCTGAGGAGCGAAAGCGTGGGGAGCGAACAG
GATTAGATACCCTGGTAGTCCACGCTGTAAACGTTGGGAACTAGGTGTGGGGATGCTATCCTGTGTTTCT
GCGCCGTAGCTAACGCATTAAGTTCCCCGCCTGGGGAGTACGGCCGCAAGGCTAAAACTCAAAGGAATTG
ACGGGGGCCCGCACAAGCGGCGGAGCATGCGGATTAATTCGATGCAACGCGAAGAACCTTACCAAGGCTT
GACATACACTGCGACATGCCAGAGATGGTGTGGCCTTCGGGGTGGTGTACAGGTGGTGCATGGTTGTCGT
CAGCTCGTGTCGTGAGATGTTGGGTTAAGTCCCGCAACGAGCGCAACCCTTGCCTCATGTTGCCAGCACG
TTATGGTGGGGACTCGTGAGGGACTGCCGGGGTTAACTCGGAGGAGGGTGGGGATGACGTCAAATCATCA
TGCCCCTTATGTCTTGGGCTTCACGCATGCTACAATGGCCAGTACAGAGGGTTGCGATACCGTGAGGTGG
GGCTAATCTCTTAAAGCTGGTCTCGGTTCGGATTGGGGTCTGCAACTCGACCCCATGAAGTTGGAGTCGC
TAGTAATCGCAGATCAGCATTGCTGCGGTGAATACGTTCTCGGGCCT
>34787157|emb|AJ576087.1|M.mulieris|Mobiluncus mulieris 16S rRNA gene, strain CCUG 30101
GGACGAACGCTGGCGGCGTGCTTAACACATGCAAGTCGAACGATGAAGCCGCAGCTTGCTGTGGTGGATT
AGTGGCGAACGGGCGAGTAACACGTGAGTAACCTGTCCTTTTCTTTGGGATAACGGCTGGAAACGGCTGC
TAATACTGGATATTCAGGCTGCATCGCATGGTGTGGTTTGGAAAGGTTTTTTCTGGAATGGGGTGGGCTC
GCGGCCTATCAGCTTGTTGGTGGGGTGATGGCTTACCAAGGCTTTGACGGGTAGCCGGCCTGAGAGGGTG
GTCGGTCGCACTGGGACTGAGATACGGCCCAGACTCCTACGGGAGGCAGCAGTGGGGAATATTGCACAAT
GGACGGAAGTTTGATGCAGCGACGCCGCGTGGAGGGTGTAGGCCTTCGGGTTGTGAACTCCTTTTTCTCG
TGAAAAAGGCATGCTTTTGGGTGTGTTGATGGTAGCGGGGGAAGAAGCGCCGGCTAACTACGTGCCAGCA
GCCGCGGTAATACGTAGGGCGCGAGCGTTGTCCGGATTTATTGGGCGTAAAGAGCTCGTAGGTGGTTCGT
CGCGTCTGTCGTGAAAGCCAGCAGCTTAACTGTTGGTCTGCGGTGGGTACGGGCGGGCTTGAGTGCGGTA
GGGGTGACTGGAATTCCTGGTGTAGCGGTGGAATGCGCAGATATCAGGAGGAACACCGATGGCGAAGGCA
GGTCACTGGGCCGTTACTGACGCTGAGGAGCGAAAGCGTGGGGAGCGAACAGGATTAGATACCCTGGTAG

TCCACGCTGTAAACGTTGGGAACTAGGTGTGGGGATGCTATCCTGTGTTTCTGCGCCGTAGCTAACGCAT
TAAGTTCCCCGCCTGGGGAGTACGGCCGCAAGGCTAAAACTCAAAGGAATTGACGGGGGCCCGCACAAGC
GGCGGAGCATGCGGATTAATTCGATGCAACGCGAAGAACCTTACCAAGGCTTGACATACACTGCGACATG
CCAGAGATGGTGTGGCCTTCGGGGTGGTGTACAGGTGGTGCATGGTTGTCGTCAGCTCGTGTCGTGAGAT
GTTGGGTTAAGTCCCGCAACGAGCGCAACCCTTGCCTCATGTTGCCAGCACGTTATGGTGGGGACTCGTG
AGGGACTGCCGGGGTTAACTCGGAGGAGGGTGGGGATGACGTCAAATCATCATGCCCCTTATGTCTTGGG
CTTCACGCATGCTACAATGGCCAGTACAGAGGGTTGCGATACCGTGAGGTGGGGCTAATCTCTTAAAGCT
GGTCTCGGTTCGGATTGGGGTCTGCAACTCGACCCCATGAAGTTGGAGTCGCTAGTAATCGCAGATCAGC
ATTGCTGCGGTGAATACGTTCTCGGGCCTTGTACACACCGCCCGTCACGTCACGAAAGTTGGTAACACCC
GAAGCCCGTGGCCTAGCCTTTTTGGGGGG
>34787158|emb|AJ576088.1|M.curtisii|Mobiluncus curtisii 16S rRNA gene, strain CCUG 44166B
GGATTAGTGGCGAACGGGCGAGTAACACGTGAGTAACCTGTCCTTTTCTTTGGGATAACGGCTGGAAACG
GCTGCTAATACTGGATATTCAGGCGTCACCGCATGGTGGTGTTTGGAAAGGTTTTTTCTGGGATTGGGTG
GGCTCGCGGCCTATCAGCTTGTTGGTGGGGTGATGGCTTACCAAGGCTTTGACGGGTAGCCGGCCTGAGA
GGGTGGTCGGTCGCACTGGGACTGAGATACGGCCCAGACTCCTACGGGAGGCAGCAGTGGGGAATATTGC
ACAATGGACGGAAGTTTGATGCAGCGACGCCGCGTGGAGGGTGTAGGCCTTCGGGTTGTGAACTCCTTTT
TCTCGCGAAAAAGGCACAGTTTTGGCTGTGTTGATGGTAGTGGGGGAAGAAGCGCCGGCTAACTACGTGC
CAGCAGCCGCGGTAATACGTAGGGCGCGAGCGTTGTCCGGATTTATTGGGCGTAAAGAGCTCGTAGGTGG
TTCGTCGCGTCTGTCGTGAAAGCCAGCAGCTTAACTGTTGGTCTGCGGTGGGTACGGGCGGGCTTGAGTG
CGGTAGGGGTGACTGGAATTCCTGGTGTAGCGGTGGAATGCGCAGATATCAGGAGGAACACCGATGGCGA
AGGCAGGTCACTGGGCCGTTACTGACACTGAGGAGCGAAAGCGTGGGGAGCGAACAGGATTAGATACCCT
GGTAGTCCACGCTGTAAACGTTGGGAACTAGGTGTGGGGATGCTATCCTGTGTTTCTGCGCCGTAGCTAA
CGCATTAAGTTCCCCGCCTGGGGAGTACGGTCGCAAGGCTAAAACTCAAAGGAATTGACGGGGGCCCGCA
CAAGCGGCGGAGCATGCGGATTAATTCGATGCAACGCGAAGAACCTTACCAAGGCTTGACATACACTGCG
ATGGTTCCAGAGATGGGCCAGCCTTCGGGGTGGTGTACAGGTGGTGCATGGTTGTCGTCAGCTCGTGTCG
TGAGATGTTGGGTTAAGTCCCGCAACGAGCGCAACCCTTGCCTCATGTTGCCAGCGGTTCGGCCGGGGAC
TCGTGGGGGACTGCCGGGGTTAACTCGGAGGAGGGTGGGGATGACGTCAAATCATCATGCCCCTTATGTC
TTGGGCTTCACGCATGCTACAATGGCCAGTACAGAGGGTTGCGATACCGTGAGGTGGGGCTAATCTCTTA
AAGCTGGTCTCGGTTCGGATTGGGGTCTGCAACTCGACCCCATGAAGTTGGAGTCGCTAGTAATCGCAGA
TCAGCATTGCTGCGGTGAATACGTTCTCGGGCCTTGTACACACCGCCCGTCACGTCACGAAAGTTGGTAA
CACCCGAAGCCCGTGGCCTAGCCTTTTTGGGGGGAGCGGTCGAAGGTGGGATTGGCGATTGGGACGAAGT
CGTAACAAGGTAGCCGTACC
>52222169|gb|AY738680.1|L.helveticus|Uncultured Lactobacillus sp. clone vag1-89 16S ribosomal RNA gene, partial sequence
TAGGGAATCTTCCACAATGGACGCAAGTCTGATGGAGCAACGCCGCGTGAGTGAAGAAGGTTTTCGGATC
GTAAAGCTCTGTTGTTGGTGAAGAAGGATAGAGGTAGTAACTGGCCTTTATTTGACGGTAATCAACCAGA
AAGTCACGGCTAACTACGTGCCAGCAGCCGCGGTAATACGTAGGTGGCAAGCGTTGTCCGGATTTATTGG
GCGTAAAGCGAGCGCAGGCGGAAGAATAAGTCTGATGTGAAAGCCCTCGGCTTAACCGAGGAACTGCATC
GGAAACTGTTTTTCTTGAGTGCAGAAGAGGAGAGTGGAACTCCATGTGTAGCGGTGGAATGCGTAGATAT
ATGGAAGAACACCAGTGGCGAAGGCGGCTCTCTGGTCTGCAACTGACGCTGAGGCTCGAAAGCATGGGTA
GCGAACAGGATTAGATACCCTGGTAGTCCATGCCGTAAACGATGAGTGCTAAGTGTTGGGAGGTTTCCGC
CTCTCAGTGCTGCAGCTAACGCATTAAGCACTCCGCCTGGGGAGTACGACCGCAAGGTTGAAACTCAAAG
GAATTGACGGGGGCCCGCACAAGCGGTGGAGCATGTGGTTTAATTCGAAGCAACGCGAAGAACCTTACCA
GGTCTTGACATCCATAGCCAGTCTAAGAGATTAGATGTTCCCTTCGGGGACTATGAGACAGGTGGTGCAT
GGCTGTCGTCAGCTCGTGTCGTGAGATGTTGGGTTAAGTCCCGCAACGAGCGCAACCCTTGTCATTAGTT
GCCAGCATTAAGTTGGGCACTCTAATGAGACTGCCGGTGACAAACCGGAGGAAGGTGGGGATGACGTCAA
GTCATCATGCCCCTTATGACCTGGGCTACACACGTGCTACAATGGACGGTACAACGAGAAGCGACCCTGT
GAAGGCAAGCGGATCTCTGAAAGCCGTTCTCAGTTCGGATTGCAGGCTGCAACTCGCCTGCATGAAGCTG
GAATCGCTAGTAATCGCAAATCAGCACGTTGCGGTGAATACGTTCCCGGGCCT
>66878916|gb|AY959210.1|L.helveticus|Uncultured bacterium clone rRNA437 16S ribosomal RNA gene, partial sequence
CCTTAGAGTTTGATCCTGGCTCAGGACGAACGCTGGCGGCGTGCCTAATACATGCAAGTCGAGCGAGTCT
GCCTTGAAGATCGGAGTGCTTGCACTCTGTGAAACAAGATACAGGCTAGCGGCGGACGGGTGAGTAACAC

GTGGGTAACCTGCCCAAGAGATCGGGATAACACCTGGAAACAGATGCTAATACCGGATAAGAAAGCAGAT
CGCATGATCAGCTTTTAAAAGGCGGCGTAAGCTGTCGCTATGGGATGGCCCCGCGGTGCATTAGCTAGTT
GGTAAGGTAAAGGCTTACCAAGGCGATGATGCATAGCCGAGTTGAGAGACTGATCGGCCACATTGGGACT
GAGACACGGCCCAAACTCCTACGGGAGGCAGCAGTAGGGAATCTTCCACAATGGACGCAAGTCTGATGGA
GCAACGCCGCGTGAGTGAAGAAGGTTTTCGGATCGTAAAGCTCTGTTGTTGGTGAAGAAGGATAGAGGTA
GTAACTGGCCTTTATTTGACGGTAATCAACCAGTAAGTCACGGCTAACTACGTGCCAGCAGCCGCGGTAA
TACGTAGGTGGCAAGCGTTGTCCGAATTTATTGGGCGTAAAGCGAGCGCAGGCGGAAGAATAAGTCTGAT
GTGAAAGCCCTCGGCTTAACCGAGGAACTGCATCGGAAACTGTTTTTCTTGAGTGCAGAAGAGGAGAGTG
GAACTCCATGTGTAGCGGTGGAATGCGTAGATATATGGAAGAACACCAGTGGCGAAGGCGGCTCTCTGGT
CTGCAACTGACGCTGAGGCTCGAGAGCATGGGTAGCGAACAGGATTAGATACCCTGGTAGTCCATGCCGT
AAACGATGAGTGCTAAGTGTTGGGAGGTTTCCGCCTCTCAGTGCTGCAGCTAACGCATTAAGCACTCCGC
CTGGGGAGTACGACCGCAAGGTTGAAACTCAAAGGAATTGACGGGGGCCCGCACAAGCGGTGGAGCATGT
GGTTTAATTCGAAGCAACGCGAAGAACCTTACCAGGTCTTGACATCCATAGCCAGTCTAAGAGATTAGAT
GTTCCCTTCGGGGACTATGAGACAGGTGGTGCATGGCTGTCGTCAGCTCGTGTCGTGAGATGTTGGGTTA
AGTCCCGCAACGAGCGCAACCCTTGTCATTAGTTGCCAGCATTAAGTTGGGCACTCTAATGAGACTGCCG
GTGACAAACCGGAGGAAGGTGGGGATGACGTCAAGTCATCATGCCCCTTATGACCTGGGCTACACACGTG
CTACAATGGACGGTACAACGAGAAGCGACCCTGTGAAGGCAAGCGGATCTCTGAAAGCCGTTCTCAGTTC
GGATTGCAGGCTGCAACTCGCCTGCATGAAGCTGGAATCGCTAGTAATCGCAAATCAGCACGTTGCGGTG
AATACGTTCCCGGGCCTTGTACACACCGCCCGTCACACCATGAGAGTCTGTAACGCCCGAAGCCGGCGGG
ATAACCGAAAGGAGTCAGCCGTCTAAGGCGGGACAGATGATTAGGGTGAAGTCGTAACAAGGTAGCCGTA
AAGGGC
>50080125|dbj|AB158767.1|L.vaginalis|Lactobacillus vaginalis gene for 16S ribosomal RNA, strain:MF2123
CGGTGTGCCTAATACATGCAAGTCGAGCGCACTGGCCNAACTGATATGACGTGCTTGCACTGAATTGACG
TTGGATTCCNAGTGAGCGGCGGACGGGTGAGTAACACGTGGGCAACCTGCCCTGAAGCGGGGGATAACAT
CTGGAAACAGGTGCTAATACCGCATAACAACGAAAACCACATGGTTTTCGTTTCAAAGATGGTTTCGGCT
ATCACTTCAGGATGGGCCCGCGGTGCATTAGCTAGTTGGTAAGGTAACGGCTTACCAAGGCGATGATGCA
TAGCCGAGTTGAGAGACTGATCGGCCACAATGGAACTGAGACACGGTCCATACTCCTACGGGAGGCAGCA
GTAGGGAATCTTCCACAATGGGCGCAAGCCTGATGGAGCAACACCGCGTGAGTGAAGAAGGGTTTCGGCT
CGTAAAGCTCTGTTGTTGGAGAAGAACGTGCGTGAGAGTAACTGTTCACGCAGTGACGGTATCCAACCAG
AAAGTCACGGCTAACTACGTGCCAGCAGCCGCGGTAATACGTAGGTGGCAAGCGTTATCCGGATTTATTG
GGCGTAAAGCGAGCGCAGGCGGTTGCTTAGGTCTGATGTGAAAGCCTTCGGCTTAACCGAAGAAGTGCAT
CGGAAACCGGGCGACTTGAGTGCAGAAGAGGACAGTGGAACTCCATGTGTAGCGGTGGAATGCGTAGATA
TATGGAAGAACACCAGTGGCGAAGGCGGCTGTCTGGTCTGCAACTGACGCTGAGGCTCGAAAGCATGGGT
AGCGAACAGGATTAGATACCCTGGTAGTCCATGCCGTAAACGATGAGTGCTAGGTGTTGGAGGGTTTCCG
CCCTTCAGTGCCGGAGCTAACGCATTAAGCACTCCGCCTGGGGAGTACGACCGCAAGGTTGAAACTCAAA
GGAATTGACGGGGGCCCGCACAAGCGGTGGAGCATGTGGTTTAATTCGAAGCTACGCGAAGAACCTTACC
AGGTCTTGACATCTTGCGCTAACCTAAGAGATTAGGCGTTCCCTTCGGGGACGCAATGACAGGTGGTGCA
TGGTCGTCGTCAGCTCGTGTCGTGAGATGTTGGGTTAAGTCCCGCAACGAGCGCAACCCTTGTTACTAGT
TGCCAGCATTTAGTTGGGCACTCTAGTGAGACTGCCGGTGACAAACCGGAGGAAGGTGGGGACGACGTCA
GATCATCATGCCCCTTATGACCTGGGCTACACACGTGCTACAATGGACGGTACAACGAGTTGCGAACTCG
CGAGAGTAAGCTAATCTCTTAAAGCCGTTCTCAGTTCGGACTGTAGGCTGCAACTCGCCTACACGAAGTC
GGAATCGCTAGTAATCGCGGATCAGCATGCCGCGGTGAATACGTTCCCGGGCCTTGTACACACCGCCCGT
CACACCATGGAAGTTTGCAATGCCCAAAGTCGGTGGCCTAACCATTTGGAGGGAACCGCCTAAGGCAGGG
CAGATGACTGGGGTGAAGTCGTAACAAGGTAG
>66878761|gb|AY959055.1|L.vaginalis|Uncultured bacterium clone rRNA282 16S ribosomal RNA gene, partial sequence
CCCTTAGAGTTTGATCCTGGCTCAGGATGAACGCCGGCGGTGTGCCTAATACATGCAAGTCGAGCGCACT
GGCCCAACTGATATGATGTGCTTGCACTGAATTGACGTTGGATTCCCAGTGAGCGGCGGACGGGTGAGTA
ACACGTGGGCAACCTGCCCTGAAGCAGGGGATAACATCTGGAAACAGGTGCTAATACCGCATAACAACGA
AAACCACATGGTTTTCGTTTTAAAGATGGTTTCGGCTATCACTTCAGGATGGGCCCGCGGTGCATTAGCT
AGTTGGTAAGGTAACGGCTTACCAAGGCGATGATGCATAGCCGAGTTGAGAGACTGATCGGCCACAATGG
AACTGAGACACGGTCCATACTCCTACGGGAGGCAGCAGTGGGGAATCTTCCACAATGGGCGCAAGCCTGA
TGGAACAACACCGCGTGAGTGAAGAAGGGTTTCGGCTCGTAAAGCTCTGTTGTTGGAGAAGAACGTGCGT

GAGAGTAACTGTTCACGCAGTGACGGTATCCAACCAGAAAGTCACGGCTAACTACGTGCCAGCAGCCGCG
GTAATACGTAGGTGGCAAGCGTTATCCGGATTTATTGGGCGTAAAGCGAGCGCAGGCGGTTGCTTAGGTC
TGATGTGAAAGCCTTCGGCTTAACCGAAGAAGTGCATCGGAAACCGGGCGACTTGAGTGCAGAAGAGGAC
AGTGGAACTCCATGTGTAGCGGTGGAATGCGTAGATATATGGAAGAACACCAGTGGCGAAGGCGGCTGTC
TGGTCTGCAACTGACGCTGAGGCTCGAAAGCATGGGTAGCGAACAGGATTAGATACCCTGGTAGTCCATG
CCGTAAACGATGAGTGCTAGGTGTTGGAGGGTTTCCGCCCTTCAGTGCCGGAGCTAACGCATTAAGCACT
CCGCCTGGGGAGTACGACCGCAAGGTTGAAACTCAAAGGAATTGACGGGGGCCCGCACAAGCGGTGGAAC
ATGTGGTTTAATTCGAAGCTACGCGAAGAACCTTACCAGGTCTTGACATCTTGCGCTAACCTAAGAGATT
AGGCGTTCCCTTCGGGGACGCAATGACAGGTGGTGCATGGTCGTCGTCAGCTCGTGTCGTGAGATGTTGG
GTTAAGTCCCGCAACGAGCGCAACCCTTGTTACTAGTTGCCAGCATTTAGTTGGGCACTCTAGTGAGACT
GCCGGTGACAAACCGGAGGAAGGTGGGGACGACGTCAGATCATCATGCCCCTTATGACCTGGGCTACACA
CGTGCTACAATGGACGGTACAACGAGTTGCGAACTCGCGAGAGTAAGCTAATCTCTTAAAGCCGTTCTCA
GTTCGGACTGTAGGTTGCAACTCGCCTACACGAAGTCGGAATCGCTAGTAATCGCGGATCAGCATGCCGC
GGTGAATACGTTCCCGGGCCTTGTACACACCGCCCGTCACACCATGGAAGTTTGCAATGCCCAAAGTCGG
TGGTCTAACCATTTGGAGGGAACCGCCTAAGGCAGGGCAGATGACTGGGGTGAA
>52222172|gb|AY738683.1|Gemella|Uncultured Gemella sp. clone BV7-73 16S ribosomal RNA gene, partial sequence
TAGGGAATCTTCCGCAATGGACGAAAGTCTGACGGAGCAACGCCGCGTGAGTGAAGAAGGATTTCGGTTC
GTAAAACTCTGTTGTTAGGGAAGAAAAAATGTATAGTAACTATATACAAAAGAGACGGTACCTAACCAGA
AAGCCACGGCTAACTACGTGCCAGCAGCCGCGGTAATACGTAGGTGGCAAGCGTTGTCCGGAATTATTGG
GCGTAAAGCGCGCGCAGGTGGTTTAGAAAGTCTGATGTGAAAGCCCACGGCTCAACCGTGGAGGGTCATT
GGAAACTAATAAACTTGAGTGCAGGAGAGAAAAGTGGAATTCCTAGTGTAGCGGTGAAATGCGTAGAGAT
TAGGAGGAACACCGGTGGCGAAAGCGGCTTTTTGGCCTGCAACTGACACTGAGGCGCGAAAGCGTGGGGA
GCAAACAGGATTAGATACCCTGGTAGTCCACGCCGTAAACGATGAGTGCTAAGTGTTGGAGTCAAAAGAC
TTCAGTGCTGCAGCAAACGCATTAAGCACTCCGCCTGGGGAGTACGATCGCAAGATTGAAACTCAAAGGA
ATTGACGGGGACCCGCACAAGCGGTGGAGTATGTGGTTTAATTCGAAGCAACGCGAAGAACCTTACCAAG
TCTTGACATACAGTGAAGATATAAGAAATTATATTGTTTTAATGTTTACATTAAACACTGATACAGGTGG
TGCATGGTTGTCGTCAGCTCGTGTCGTGAGATGTTGGGTTAAGTCCCGCAACGAGCGCAACCCTTATATC
TAGTTACCAGCAGTAAGATGGGGACTCTAGATAGACTGCCAGTGACAAACTGGAGGAAGGTGGGGATGAC
GTCAAATCATCATGCCCCTTATGACTTGGGCTACACACGTACTACAATGGATAGGAACAAAGAGAAGCGA
CCTCGCAAGAGCAAGCCAACCTCAGAAAACTATTCTCAGTTCGGATTGTAGGCTGCAACTCGCCTACATG
AAGCTGGAATCGCTAGTAATCGCGAATCAGAATGTCGCGGTGAATACGTTCCCGGGTCT
>167599469|gb|EU427463.1|Gemella|Gemella sp. WAL 1945J 16S ribosomal RNA gene, partial sequence
CGAACTTTAAGAGTGCTTGCACACTTAAAGTTAGCGGCGAACGGGTGAGTAACACGTAAAGAACCTACCT
TATAGACAGGGACAACTATTGGAAACGATAGCTAATACCTGATAAGAAAGAAACTCGCATGAGAGAAGTT
CGAAAGTCGGAGCAATCTGACACTATAAGATGGCTTTGCGGTGCATTAGCTAGTTGGTAGGGTAAAAGCC
TACCAAGGCGACGATGCATAGCCGACCTGAGAGGGTGATCGGCCACATTGGGACTGAGACACGGCCCAGA
CTCCTACGGGAGGCAGCAGTAGGGAATCTTCCGCAATGGACGCAAGTCTGACGGAGCAACGCCGCGTGAG
TGAAGAAGGATTTCGGTTCGTAAAACTCTGTTGTTAGGGAAGAAAAAATGTATAATAACTATATACAAAA
GAGACGGTACCTAACCAGAAAGCCACGGCTAACTACGTGCCAGCAGCCGCGGTAATACGTAGGTGGCAAG
CGTTGTCCGGAATTATTGGGCGTAAAGCGCGCGCAGGTGGTTTAGAAAGTCTGATGTGAAAGCCCACGGC
TCAACCGTGGAGGGTCATTGGAAACTAATAAACTTGAGTGCAGGAGAGAAAAGTGGAATTCCTAGTGTAG
CGGTGAAATGCGTAGAGATTAGGAGGAACACCGGTGGCGAAAGCGGCTTTTTGGCCTGCAACTGACACTG
AGGCGCGAAAGCGTGGGGAGCAAACAGGATTAGATACCCTGGTAGTCCACGCCGTAAACGATGAGTGCTA
AGTGTTGGAGTCAAAAGACTTCAGTGCTGCCAGCAAACGCATTAAGCACTCCGCCTGGGGAGTACGATCG
CAAGATTGAAACTCAAAGGAATTGACGGGGACCCGCACAAGCGGTGGAGTATGTGGTTTAATTCGAAGCA
ACGCGAAGAACCTTACCAAGTCTTGACATACAGTGAAGATATAAGAAATTATATTGTTTTAATGTTTACA
TTAAACACTGATACAGGTGGTGCATGGTTGTCGTCAGCTCGTGTCGTGAGATGTTGGGTTAAGTCCCGCA
ACGAGCGCAACCCTTATATCTAGTTACCAGCAGTAAGATGGGGACTCTAGATAGACTGCCAGTGACAAAC
TGGAGGAAGGTGGGGATGACGTCAAATCATCATGCCCCTTATGACTTGGGCTACACACGTACTACAATGG
ATAGGAACAAAGAGAAGCGACCTCGCAAGAGCAAGCCAACCTCAGAAAACTATTCTCAGTTCGGATTGTA
GGCTGCAACTCGCCTACATGAAGCTGGAATCGCTAGTAATCGCGAATCAGAATGTCGCGGTGAATACGTT
CCCGGGTCTTGTACACACCGCCCGTCACACCACGAGAGTTTGTAACACCTGAAGACGGTGGCCTAACCGT

AAG
>63146109|gb|AY995244.1|Gemella|Uncultured Gemella sp. clone FX80W16 16S ribosomal RNA gene, partial sequence
AGCCGGCGTGCCTATACATGCAGTCGAGCGAACTTTAAGAGTGCTTGCACACTTAAAGTTAGCGGCGAAA
GGGTGAGTAACACGTAAAGAACCTACCTTATAGACAGGGACAACTATTGGAAACGATAGCTAATACCTGA
TAAGAAAGAAACTCGCATGAGAGAAGTTCGAAAGTCGGAGCAATCTGACACTATAAGATGGCTTTGCGGT
GCATTAGCTAGTTGGTAGGGTAAAAGCCTACCAAGGCGACGATGCATAGCCGACCTGAGAGGGTGATCGG
CCACATTGGGACTGAGACACGGCCCAGACTCCTACGGGAGGCAGCAGTAGGGAATCTTCCGCAATGGACG
AAAGTCTGACGGAGCAACGCCGCGTGAGTGAAGAAGGATTTCGGTTCGTAAAACACTGTTGTTAGGGAAG
AAAAAATGTATAGTAACTATATACAAAAGAGACGGTACCTAACCAGAAAGCCACGGCTAACTACGTGCCA
GCAGCCGCGGTAATACGTAGGTGGCAAGCGTTGTCCGGAATTATTGGGCGTAAAGCGCGCGCAGGTGGTT
TAGAAAGTCTGATGTGAAAGCCCACGGCTCAACCGTGGAGGGTCATTGGAAACTAATAAACTTGAGTGCA
GGAGAGTAAAGTGGAATTCCTAGTGTAGCGGTGAAATGCGTAGAGATTAGGAGGAACACCGGTGGCGAAA
GCGGCTTTTTGGCCTGCAACTGACACTGAGGCGCGAAAGCGTGGGGAGCAAACAGGGTTAGATACCCTGG
TAGTCCACGCCGTAAACGATGAGTGCTAAGTGTTGGAGTCAAAAGACTTCAGTGCTGCAGCAAACGCATT
AAGCACTCCGCCTGGGGAGTACGATCGCAAGATTGAAACTCAAAGGAATTGACGGGGACCCGCACAAGCG
GTGGAGTATGTGGTTTAATTCGAAGCAACGCGAAGAACCTTACCAAGTCTTGACATACAGTGAAGATATA
AGAAATTATATTGTTTTAATGTTTACATTAAACACTGATACAGGTGGTGCATGGTTGTCGTCAGCTCGTG
TCGTGAGATGTTGGGTTAAGTCCCGCAACGAGCGCAACCCTTATATCTAGTTACCAGCAGTAAGATGGGG
ACTCTAGATAGACTGCCAGTGACAAACTGGAGGAAGGTGGGGATGACGTCAAATCATCATGCCCCTTATG
ACTTGGGCTACACACGTACTACAATGGATAGGAACAAAGAGAAGCGACCTCGCAAGAGCAAGCCAACCTC
AGAAAACTATTCTCAGTTCGGATTGTAGGCTGCAACTCGCCTACATGAAGCTGGAATCGCTAGTAATCGC
GAATCAGAATTCGCGGTAATT
>52222163|gb|AY738674.1|Porphyromonas|Uncultured Porphyromonas sp. clone 123-f 45 16S ribosomal RNA gene, partial sequence
TGAGGAATATTGGTCAATGGGCGAGAGCCTGAACCAGCCAAGTCGCGTGAAGGAAGACTGCCCGCAAGGG
TTGTAAACTTCTTTTGTATGGGATTAAAGTCACCTACGTGTAGGTGTTTGCAGTTACCATACGAATAAGC
ATCGGCTAACTCCGTGCCAGCAGCCGCGGTAATACGGAGGATGCGAGCGTTATCCGGAATTATTGGGTTT
AAAGGGTGCGTAGGTTGCAAGGGAAGTCAGGGGTGAAAAGCTATAGCTCAACTATGGTCTTGCCTTTGAA
ACTCTCTAGCTAGAGTGTACTGGAGGTACGTGGAACGTGTGGTGTAGCGGTGAAATGCATAGATATCACA
CAGAACTCCGATTGCGCAGGCAGCGTACTACATTACAACTGACACTGAAGCACGAAAGCGTGGGTATCAA
ACAGGATTAGATACCCTGGTAGTCCACGCAGTAAACGATGAATACTAGATCTATGCGATATGACAGTATG
GGTCTAAGCGAAAGCGATAAGTATTCCACCTGGGGAGTACGCCGGCAACGGTGAAACTCAAAGAGATTGG
CGGGGGTCCGCACAAGCGGAGGAACATGTGGTTTAATTCGATGATACGCGAGGAACCTTACCCGGGATTG
AAATGTAGATGCATGAGGCTGAGAGGTCTCTTCCCTTCGGGGCTTCTATGTAGGTGCTGCATGGTTGTCG
TCAGCTCGTGCCGTGAGGTGTCGGCTTAAGTGCCATAACGAGCGCAACCCGCGTCGATAGTTACTAACGA
GTTAAGTCGAGGACTCTATCGAGACAGCCGTCGTAAGACGAGAGGAAGGAGCGGATGACGTCAAATCAGC
ACGGCCCTTACATCCGGGGCGACACACGTGTTACAATGGTAGGGACAGCGAGCAGCCATCTGGCGACAGA
GAGCTAATCTATAAACCCTATCCCAGTTCGGATCGGAGTCTGCAACTCGACTCTGTGAAGCTGGATTCGC
TAGTAATCGCGCATCAGCCATGGCGCGGTGAATACGTTCCCGGTCCT
>257480659|gb|GQ422746.1|Porphyromonas|Porphyromonas uenonis strain F0120 16S ribosomal RNA gene, partial sequence
AAATGAGTTTGACTCTGGCTCAGGATGAACGCTAGCGATAGGCTTAACACATGCAAGTCGAGGGGCAGCG
AGATGTAGCAATACGTCGTCGGCGACCGGCGAATGGGTGAGTAACACGTATGCAACTTACCTCTTAGTGG
TGAATAACTCGATGAAAGTCGGACTAATACACCATACTCTCCTTAGATCACATGAGAAGAGGAGGAAAGA
TTAATCGCTAAGAGATAGGCCTGCGTTCCATTAGCTAGTTGGTAAGGTAACGGCTTACCAAGGCAACGAT
GGATAGGGGGACTGAGAGGTTGACCCCCCACATTGACACTGAGATACGGGTCAAACTCCTACGGGAGGCA
GCAGTGAGGAATATTGGTCAATGGGCGAGAGCCTGAACCAGCCAAGTCGCGTGAAGGAAGACTGCCCGCA
AGGGTTGTAAACTTCTTTTGTATGGGATTAAAGTCACCTACGTGTAGACGTTTGCAGTTACCATACGAAT
AAGCATCGGCTAACTCCGTGCCAGCAGCCGCGGTAATACGGAGGATGCGAGCGTTATCCGGAATTATTGG
GTTTAAAGGGTGCGTAGGTTGCAAGGGAAGTCAGGGGTGAAAAGCTATAGCTCAACTATGGTCTTGCCTT
TGAAACTCTCTAGCTAGAGTGTACTGGAGGTACGTGGAACGTGTGGTGTAGCGGTGAAATGCATAGATAT
CACACAGAACTCCGATTGCGCAGGCAGCGTACTACATTACAACTGACACTGAAGCACGAAAGCGTGGGTA
TCAAACAGGATTAGATACCCTGGTAGTCCACGCAGTAAACGATGAATACTAGATCTATGCGATATGACAG

TATGGGTCTAAGCGAAAGCGATAAGTATTCCACCTGGGGAGTACGCCGGCAACGGTGAAACTCAAAGAGA
TTGGCGGGGGTCCGCACAAGCGGAGGAACATGTGGTTTAATTCGATGATACGCGAGGAACCTTACCCGGG
ATTGAAATGTAGATGCATGAGGCTGAGAGGTCTCTTCCCTTCGGGGCTTCTATGTAGGTGCTGCATGGTT
GTCGTCAGCTCGTGCCGTGAGGTGTCGGCTTAAGTGCCATAACGAGCGCAACCCGCGTCGATAGTTACTA
ACGAGTCAAGTCGAGGACTCTATCGAGACAGCCGTCGTAAGACGAGAGGAAGGAGCGGATGACGTCAAAT
CAGCACGGCCCTTACATCCGGGGCGACACACGTGTTACAATGGTAGGGACAGCGAGCAGCCATCTGGCGA
CAGAGAGCTAATCTATAAACCCTATCCCAGTTCGGATCGGAGTCTGCAACTCGACTCTGTGAAGCTGGAT
TCGCTAGTAATCGCGCATCAGCCATGGCGCGGTGAATACGTTCCCGGACCTTGCACACACCGCCCGTCAA
GCCATGGGAGTCGGGGGTACCTGAAGAGCGTGACCGTCACAGGAGCGCTTGAGGGTAAAACTGGTGACTG
GGGCTAAGTCGTAACAAGGTAGCCGTACCGGAAGGTGC
>302129291|dbj|AB547653.1|Porphyromonas|Porphyromonas asaccharolytica gene for 16S ribosomal RNA, partial sequence, strain: JCM 6326
CTGGCTCAGGATGAACGCTAGCGATAGGCTTAACACATGCAAGTCGAGGGGCAGCGAGATGTAGCAATAC
GTCGTCGGCGACCGGCGAATGGGTGAGTAACACGTATGCAACTTACCTCTTAGTGGTGAATAACCCGATG
AAAGTCGGACTAATACACCATATACTCCTTAGATCCCATGAGAAGAGGAGGAAAGATTAATCGCTAAGAG
ATGGGCCTGCGTTCCATTAGCTAGTTGGTAAGGTAACGGCTTACCAAGGCAACGATGGATAGGGGGACTG
AGAGGTTGACCCCCCACATTGACACTGAGATACGGGTCAAACTCCTACGGGAGGCAGCAGTGAGGAATAT
TGGTCAATGGGCGAGAGCCTGAACCAGCCAAGTCGCGTGAAGGAAGACTGCCCGCAAGGGTTGTAAACTT
CTTTTGTATGGGATTAAAGTCACCTACGTGTAGGTGTTTGCAGTTACCATACGAATAAGCATCGGCTAAC
TCCGTGCCAGCAGCCGCGGTAATACGGAGGATGCGAGCGTTATCCGGAATTATTGGGTTTAAAGGGTGCG
TAGGTTGCAAGGGAAGTCAGGGGTGAAAAGCTGTAGCTCAACTATGGTCTTGCCTTTGAAACTCTCTAGC
TAGAGTGTACTGGAGGTACGTGGAACGTGTGGTGTAGCGGTGAAATGCATAGATATCACACAGAACTCCG
ATTGCGCAGGCAGCGTACTACATTACAACTGACACTGAAGCACGAAAGCGTGGGTATCCAACAGGATTAG
ATACCCTGGTAGTCCACGCAGTAAACGATGAATACTAGATCTATGCGATATACAGTATGGGTCTAAGCGA
AAGCGATAAGTATTCCACCTGGGGAGTACGCCGGCAACGGTGAAACTCAAAGAGATTGGCGGGGGTCCGC
ACAAGCGGAGGAACATGTGGTTTAATTCGATGATACGCGAGGAACCTTACCCGGGATTGAAATGTAGATG
CATGAGGCTGAGAGGTCTCTTCCCTTCGGGGCTTCTATGTAGGTGCTGCATGGTTGTCGTCAGCTCGTGC
CGTGAGGTGTCGGCTTAAGTGCCATAACGAGCGCAACCCGCGTCGATAGTTACTAACGAGTCAAGTCGAG
GACTCTATCGAGACAGCCGTCGTAAGACGTGAGGAAGGAGCGGATGACGTCAAATCAGCACGGCCCTTAC
ATCCGGGGCGACACACGTGTTACAATGGTAGGGACAGCGAGCAGCCATCTGGTGACAGAGAGCTAATCTA
TAAACCCTATCCCAGTTCGGATCGGAGTCTGCAACTCGACTCTGTGAAGCTGGATTCGCTAGTAATCGCG
CATCAGCCATGGCGCGGTGAATACGTTCCCGGACCTTGCACACACCGCCCGTCAAGCCATGGGAGTCGGG
GGTACCTGAAGAGCGTGACCGTCACAGGAGCGCTTGAGGGTAAAACTGGTGACTGGGGCTAAGTCGTAAC
AAGGTAACC
>52222160|gb|AY738671.1|L.gasseri|Uncultured Lactobacillus sp. clone vag2-24 16S ribosomal RNA gene, partial sequence
TAGGGAATCTTCCACAATGGACACAAGTCTGATGGAGCAACGCCGCGTGAGTGAAGAAGGGTTTCGGCTC
GTAAAGCTCTGTTGGTAGTGAAGAAAGATAGAGGTAGTAACTGGCCTTTATTTGACGGTAATTACTTAGA
AAGTCACGGCTAACTACGTGCCAGCAGCCGCGGTAATACGTAGGTGGCAAGCGTTGTCCGGATTTATTGG
GCGTAAAGCGAGTGCAGGCGGTTCAATAAGTCTGATGTGAAAGCCTTCGGCTCAACCGGAGAATTGCATC
AGAAACTGTTGAACTTGAGTGCAGAAGAGGAGAGTGGAACTCCATGTGTAGCGGTGGAATGCGTAGATAT
ATGGAAGAACACCAGTGGCGAAGGCGGCTCTCTGGTCTGCAACTGACGCTGAGGCTCGAAAGCATGGGTA
GCGAACAGGATTAGATACCCTGGTAGTCCATGCCGTAAACGATGAGTGCTAAGTGTTGGGAGGTTTCCGC
CTCTCAGTGCTGCAGCTAACGCATTAAGCACTCCGCCTGGGGAGTACGACCGCAAGGTTGAAACTCAAAG
GAATTGACGGGGGCCCGCACAAGCGGTGGAGCATGTGGTTTAATTCGAAGCAACGCGAAGAACCTTACCA
GGTCTTGACATCCAGTGCAAACCTAAGAGATTAGGAGTTCCCTTCGGGGACGCTGAGACAGGTGGTGCAT
GGCTGTCGTCAGCTCGTGTCGTGAGATGTTGGGTTAAGTCCCGCAACGAGCGCAACCCTTGTCATTAGTT
GCCATCATTAAGTTGGGCACTCTAATGAGACTGCCGGTGACAAACCGGAGGAAGGTGGGGATGACGTCAA
GTCATCATGCCCCTTATGACCTGGGCTACACACGTGCTACAATGGACGGTACAACGAGAAGCGAACCTGC
GAAGGCAAGCGGATCTCTGAAAGCCGTTCTCAGTTCGGACTGTAGGCTGCAACTCGCCTACACGAAGCTG
GAATCGCTAGTAATCGCGGATCAGCACGCCGCGGTGAATACGTTCCCGGGCCT
>294438974|gb|GU454858.1|L.gasseri|Lactobacillus gasseri strain 177-3 16S ribosomal RNA gene, partial sequence
CTATACATGCAAGTCGAGCGAGCTTGCCTAGATGAATTTGGTGCTTGCACCAAATGAAACTAGATACAAG

CGAGCGGCGGACGGGTGAGTAACACGTGGGTAACCTGCCCAAGAGACTGGGATAACACCTGGAAACAGAT
GCTAATACCGGATAACAACACTAGACGCATGTCTAGAGTTTAAAAGATGGTTCTGCTATCACTCTTGGAT
GGACCTGCGGTGCATTAGCTAGTTGGTAAGGTAACGGCTTACCAAGGCAATGATGCATAGCCGAGTTGAG
AGACTGATCGGCCACATTGGGACTGAGACACGGCCCAAACTCCTACGGGAGGCAGCAGTAGGGAATCTTC
CACAATGGACGCAAGTCTGATGGAGCAACGCCGCGTGAGTGAAGAAGGGTTTCGGCTCGTAAAGCTCTGT
TGGTAGTGAAGAAAGATAGAGGTAGTAACTGGCCTTTATTTGACGGTAATTACTTAGAAAGTCACGGCTA
ACTACGTGCCAGCAGCCGCGGTAATACGTAGGTGGCAAGCGTTGTCCGGATTTATTGGGCGTAAAGCGAG
TGCAGGCGGTTCAATAAGTCTGATGTGAAAGCCTTCGGCTCAACCGGAGAATTGCATCAGAAACTGTTGA
ACTTGAGTGCAGAAGAGGAGAGTGGAACTCCATGTGTAGCGGTGGAATGCGTAGATATATGGAAGAACAC
CAGTGGCGAAGGCGGCTCTCTGGTCTGCAACTGACGCTGAGGCTCGAAAGCATGGGTAGCGAACAGGATT
AGATACCCTGGTAGTCCATGCCGTAAACGATGAGTGCTAAGTGTTGGGAGGTTTCCGCCTCTCAGTGCTG
CAGCTAACGCATTAAGCACTCCGCCTGGGGAGTACGACCGCAAGGTTGAAACTCAAAGGAATTGACGGGG
GCCCGCACAAGCGGTGGAGCATGTGGTTTAATTCGAAGCAACGCGAAGAACCTTACCAGGTCTTGACATC
CAGTGCAAACCTAAGAGATTAGGAGTTCCCTTCGGGGACGCTGAGACAGGTGGTGCATGGCTGTCGTCAG
CTCGTGTCGTGAGATGTTGGGTTAAGTCCCGCAACGAGCGCAACCCTTGTCATTAGTTGCCATCATTAAG
TTGGGCACTCTAATGAGACTGCCGGTGACAAACCGGAGGAAGGTGGGGATGACGTCAAGTCATCATGCCC
CTTATGACCTGGGCTACACACGTGCTACAATGGACGGTACAACGAGAAGCGAACCTGCGAAGGCAAGCGG
ATCTCTGAAAGCCGTTCTCAGTTCGGACTGTAGGCTGCAACTCGCCTACACGAAGCTGGAATCGCTAGTA
ATCGCGGATCAGCACGCCGCGGTGAATACGTTCCCGGGCCTTGTACACACCGCCCGTCACACCATGAGAG
TCTGTAACACCCAAAGCCGGTGGGATAACCTTTATAGGAGTCAGCCGTCTAAGGTAGA
>257074927|dbj|AB517146.1|L.gasseri|Lactobacillus gasseri gene for 16S rRNA, complete sequence
GAGTTTGATCCTGGCTCAGGACGAACGCTGGCGGCGTGCCTAATACATGCAAGTCGAGCGAGCTTGCCTA
GATGAATTTGGTGCTTGCACCAAATGAAACTAGATACAAGCGAGCGGCGGACGGGTGAGTAACACGTGGG
TAACCTGCCCAAGAGACTGGGATAACACCTGGAAACAGATGCTAATACCGGATAACAACACTAGACGCAT
GTCTAGAGTTTAAAAGATGGTTCTGCTATCACTCTTGGATGGACCTGCGGTGCATTAGCTAGTTGGTAAG
GTAACGGCTTACCAAGGCAATGATGCATAGCCGAGTTGAGAGACTGATCGGCCACATTGGGACTGAGACA
CGGCCCAAACTCCTACGGGAGGCAGCAGTAGGGAATCTTCCACAATGGACGCAAGTCTGATGGAGCAACG
CCGCGTGAGTGAAGAAGGGTTTCGGCTCGTAAAGCTCTGTTGGTAGTGAAGAAAGATAGAGGTAGTAACT
GGCCTTTATTTGACGGTAATTACTTAGAAAGTCACGGCTAACTACGTGCCAGCAGCCGCGGTAATACGTA
GGTGGCAAGCGTTGTCCGGATTTATTGGGCGTAAAGCGAGTGCAGGCGGTTCAATAAGTCTGATGTGAAA
GCCTTCGGCTCAACCGGAGAATTGCATCAGAAACTGTTGAACTTGAGTGCAGAAGAGGAGAGTGGAACTC
CATGTGTAGCGGTGGAATGCGTAGATATATGGAAGAACACCAGTGGCGAAGGCGGCTCTCTGGTCTGCAA
CTGACGCTGAGGCTCGAAAGCATGGGTAGCGAACAGGATTAGATACCCTGGTAGTCCATGCCGTAAACGA
TGAGTGCTAAGTGTTGGGAGGTTTCCGCCTCTCAGTGCTGCAGCTAACGCATTAAGCACTCCGCCTGGGG
AGTACGACCGCAAGGTTGAAACTCAAAGGAATTGACGGGGGCCCGCACAAGCGGTGGAGCATGTGGTTTA
ATTCGAAGCAACGCGAAGAACCTTACCAGGTCTTGACATCCAGTGCAAACCTAAGAGATTAGGAGTTCCC
TTCGGGGACGCTGAGACAGGTGGTGCATGGCTGTCGTCAGCTCGTGTCGTGAGATGTTGGGTTAAGTCCC
GCAACGAGCGCAACCCTTGTCATTAGTTGCCATCATTAAGTTGGGCACTCTAATGAGACTGCCGGTGACA
AACCGGAGGAAGGTGGGGATGACGTCAAGTCATCATGCCCCTTATGACCTGGGCTACACACGTGCTACAA
TGGACGGTACAACGAGAAGCGAACCTGCGAAGGCAAGCGGATCTCTGAAAGCCGTTCTCAGTTCGGACTG
TAGGCTGCAACTCGCCTACACGAAGCTGGAATCGCTAGTAATCGCGGATCAGCACGCCGCGGTGAATACG
TTCCCGGGCCTTGTACACACCGCCCGTCACACCATGAGAGTCTGTAACACCCAAAGCCGGTGGGATAACC
TTTATAGGAGTCAGCCGTCTAAGGTAGGACAGATGATTAGGGTGAAGTCGTAACAAGGTAGCC
>66878804|gb|AY959098.1|L.gasseri|Uncultured bacterium clone rRNA325 16S ribosomal RNA gene, partial sequence
GCCCTTAGAGTTTGATCCTGGCTCAGGACGAACGCTGGCGGCGTGCCTAATACATGCAAGTCGAGCGAGC
TTGCCTAGATGAATTTGGTGCTTGCACCAGATGAAACTAGATACAAGCGAGCGGCGGACGGGTGAGTAAC
ACGTGGGTAACCTGCCCAAGAGACTGGGATAACACCTGGAAACAGATGCTAATACCGGATAACAACACTA
GACGCATGTCTAGAGTTTAAAAGATGGTTCTGCTATCACTCTTGGATGGACCTGCGGTGCATTAGCTAGT
TGGTAAGGTAACGGCTTACCAAGGCAATGATGCATAGCCGAGTTGAGAGACTGATCGGCCACATTGGGAC
TGAGACACGGCCCAAACTCCTACGGGAGGCAGCAGTAGGGAATCTTCCACAATGGACGCAAGTCTGATGG
AGCAACGCCGCGTGAGTGAAGAAGGGTTTCGGCTCGTAAAGCTCTGTTGGTAGTGAAGAAAGATAGAGGT
AGTAACTGGCCTTTATTTGACGGTAATTACTTAGAAAGTCACGGCTAACTACGTGCCAGCAGCCGCGGTA

ATACGTAGGTGGCAAGCGTTGTCCGGATTTATTGGGCGTAAAGCGAGTGCAGGCGGTTCAATAAGTCTGA
TGTGAAAGCCTTCGGCTCAACCGGAGAATTGCATCAGAAACTGTTGAACTTGAGTGCAGAAGAGGAGAGT
GGAACTCCATGTGTAGCGGTGGAATGCGTAGATATATGGAAGAACACCAGTGGCGAAGGCGGCTCTCTGG
TCTGCGACTGACGCTGAGGCTCGAAAGCATGGGTAGCGAACAGGATTAGATACCCTGGTAGTCCATGCCG
TAAACGATGAGTGCTAAGTGTTGGGAGGTTTCCGCCTCTCAGTGCTGCAGCTAACGCATTAAGCACTCCG
CCTGGGGAGTACGACCGCAAGGTTGAAACTCAAAGGAATTGACGGGGGCCCGCACAAGCGGTGGAGCATG
TGGTTTAATTCGAAGCAACGCGAAGAACCTTACCAGGTCTTGACATCCAGTGCAAACCTAAGAGATTAGG
TGTTCCCTTCGGGGACGCTGAGACAGGTGGTGCATGGCTGTCGTCAGCTCGTGTCGTGAGATGTTGGGTT
AAGTCCCGCAACGAGCGCAACCCTTGTCATTAGTTGCCATCATTAAGTTGGGCACTCTAATGAGACTGCC
GGTGACAAACCGGAGGAAGGTGGGGATGACGTCAAGTCATCATGCCCCTTATGACCTGGGCTACACACGT
GCTACAATGGACGGTACAACGAGAAGCGAACCTGCGAAGGCAAGCGGATCTCTGAAAGCCGTTCTCAGTT
CGGACTGTAGGCTGCAACTCGCCTACACGAAGCTGGAATCGCTAGTAATCGCGGATCAGCACGCCGCGGT
GAATACGTTCCCGGGCCTTGTACACACCGCCCGTCACACCATGAGAGTCTGTAACACCCAAAGCCGGTGG
GATAACCTTTATAGGAGTCAGCCGTCTAAGGTAGGACAGATGATTAGGGTGAAGTCGTAACAAGGTAACC
GTAAAGGGCG
>52222149|gb|AY738660.1|L.jensenii|Uncultured Lactobacillus sp. clone vag4-103 16S ribosomal RNA gene, partial sequence
TAGGGAAATCTTCCACAATGGACGCAAGTCTGATGGAGCAACGCCGCGTGAGTGAAGAAGGTTTTCGGAT
CGTAAAGCTCTGTTGTTGGTGAAGAAGGATAGAGGTAGTAACTGGCCTTTATTTGACGGTAATCAACCAG
AAAGTCACGGCTAACTACGTGCCAGCAGCCGCGGTAATACGTAGGTGGCAAGCGTTGTCCGGATTTATTG
GGCGTAAAGCGAGCGCAGGCGGATTGATAAGTCTGATGTGAAAGCCTTCGGCTCAACCGAAGAACTGCAT
CAGAAACTGTCAATCTTGAGTGCAGAAGAGGAGAGTGGAACTCCATGTGTAGCGGTGGAATGCGTAGATA
TATGGAAGAACACCAGTGGCGAAGGCGGCTCTCTGGTCTGTAACTGACGCTGAGGCTCGAAAGCATGGGT
AGCGAACAGGATTAGATACCCTGGTAGTCCATGCCGTAAACGATGAGTGCTAAGTGTTGGGAGGTTTCCG
CCTCTCAGTGCTGCAGCTAACGCATTAAGCACTCCGCCTGGGGAGTACGACCGCAAGGTTGAAACTCAAA
GGAATTGACGGGGGCCCGCACAAGCGGTGGAGCATGTGGTTTAATTCGAAGCAACGCGAAGAACCTTACC
AGGTCTTGACATCCTTTGACCACCTAAGAGATTAGGTTTTCCCTTCGGGGACAAAGAGACAGGTGGTGCA
TGGCTGTCGTCAGCTCGTGTCGTGAGATGTTGGGTTAAGTCCCGCAACGAGCGCAACCCTTGTTAATAGT
TGCCAGCATTAAGTTGGGCACTCTATTGAGACTGCCGGTGACAAACCGGAGGAAGGTGGGGATGACGTCA
AGTCATCATGCCCCTTATGACCTGGGCTACACACGTGCTACAATGGGCAGTACAACGAGAAGCGAACCTG
TGAAGGCAAGCGGATCTCTTAAAGCTGTTCTCAGTTCGGACTGTAGGCTGCAACTCGCCTACACGAAGCT
GGAATCGCTAGTAATCGCGGATCAGCACGCCGCGGTGAATACGTTCCCGGGCCT
>7621500|gb|AF243143.1|L.jensenii|Lactobacillus jensenii strain BLB1a 16S ribosomal RNA gene, partial sequence
GCGTGCCTAATACATGCAAGTCGAGCGAGCTTGCCTATTGAAATTCTTCGGAATGGACATAGATACAAGC
TAGCGGCGGATGGGTGAGTAACGCGTGGGTAACCTGCCCTTAAGTCTGGGATACCATTTGGAAACAGATG
CTAATACCGGATAAAAGCTACTTTCGCATGAAAGAAGTTTAAAAGGCGGCGTAAGCTGTCGCTAAAGGAT
GGACCTGCGATGCATTAGCTAGTTGGTAAGGCAACGGCTTACCAAGGCGATGATGCATAGCCGAGTTGAG
AGACTGATCGGCCACATTGGGACTGAGACACGGCCCAAACTCCTACGGGAGGCAGCAGTAGGGAATCTTC
CACAATGGACGCAAGTCTGATGGAGCAACGCCGCGTGAGTGAAGAAGGTTTTCGGATCGTAAAGCTCTGT
TGTTGGTGAAGAAGGATAGAGGTAGTAACTGGCCTTTATTTGACGGTAATCAACCAGAAAGTCACGGCTA
ACTACGTGCCAGCAGCCGCGGTAATACGTAGGTGGCAAGCGTTGTCCGGATTTATTGGGCGTAAAGCGAG
CGCAGGCGGATTGATAAGTCTGATGTGAAAGCCTTCGGCTCAACCGAAGAACTGCATCAGAAACTGTCAA
TCTTGAGTGCAGAAGAGGAGAGTGGAACTCCATGTGTAGCGGTGGAATGCGTAGATATATGGAAGAACAC
CAGTGGCGAAGGCGGCTCTCTGGTCTGTAACTGACGCTGAGGCTCGAAAGCATGGGTAGCGAACAGGATT
AGATACCCTGGTAGTCCATGCCGTAAACGATGAGTGCTAAGTGTTGGGAGGTTTCCGCCTCTCAGTGCTG
CAGCTAACGCATTAAGCACTCCGCCTGGGGAGTACGACCGCAAGGTTGAAACTCAAAGGAATTGACGGGG
GCCCGCACAAGCGGTGGAGCATGTGGTTTAATTCGAAGCAACGCGAAGAACCTTACCAGGTCTTGACATC
CTTTGACCACCTAAGAGATTAGGTTTTCCCTTCGGGGACAAAGAGACAGGTGGTGCATGGCTGTCGTCAG
CTCGTGTCGTGAGATGTTGGGTTAAGTCCCGCAACGAGCGCAACCCTTGTTAATAGTTGCCAGCATTAAG
TTGGGCACTCTATTGAGACTGCCGGTGACAAACCGGAGGAAGGTGGGGATGACGTCAAGTCATCATGCCC
CTTATGACCTGGGCTACACACGTGCTACAATGGGCAGTACAACGAGAAGCGAACCTGTGAAGGCAAGCGG
ATCTCTTAAAGCTGTTCTCAGTTCGGACTGTAGGCTGCAACTCGCCTACACGAAGCTGGAATCGCTAGTA
ATCGCGGATCAGCACGCCGCGGTGAATACGTTCCCGGGCCTTGTACACACCGCCCGTCACACCATGAGAG

TTTGTAACACCCAAAGTCGGTGAGGTAACTTTTGGAGCCAGCCGCTTAAGGTGGGACAGATGATTAGGGT
GAAGTCGTAACAAGGTAGCTGTAGGAGAACTA
>66878835|gb|AY959129.1|L.jensenii|Uncultured bacterium clone rRNA356 16S ribosomal RNA gene, partial sequence
ACGAACGCTGGCGGCGTGCCTAATACATGCAAGTCGAGCGAGTCTGCCTTGAAGATCGGAGTGCTTGCAC
TCTGTGAAACAAGATACAGGCTAGCGGCGGACGGGTGAGTAACACGTGGGTAACCTGCCCAAGAGATCGG
GATAACACCTGGAAACAGATGCTAATACCGGATAACAACAGATGATGCCTATCAACTGTTTAAAAGATGG
TTCTGCTATCACTCTTGGATGGACCTGCGGTGCATTAGCTAGTTGGTAGGGTAACGGCCTACCAAGGCGA
TGATGCATAGCCGAGTTGAGAGACTGATCGGCCACATTGGGACTGAGACACGGCCCAAACTCCTACGGGA
GGCAGCAGTAGGGAATCTTCCACAATGGACGCAAGTCTGATGGAGCAACGCCGCGTGAGTGAAGAAGGTT
TTCGGATCGTAAAGCTCTGTTGTTGGTGAAGAAGGATAGAGGTAGTAACTGGCCTTTATTTGACGGTAAT
CAACCAGAAAGTCACGGCTAACTACGTGCCAGCAGCCGCGGTAATACGTAGGTGGCAAGCGTTGTCCGGA
TTTATTGGGCGTAAAGCGAGCGCAGGCGGATTGATAAGTCTGATGTGAAAGCCTTCGGCTCAACCGAAGA
ACTGCATCAGAAACTGTCAATCTTGAGTGCAGAAGAGGAGAGTGGAACTCCATGTGTAGCGGTGGAATGC
GTAGATATATGGAAGAACACCAGTGGCGAAGGCGGCTCTCTGGTCTGTAACTGACGCTGAGGCTCGAAAG
CATGGGTAGCGAACAGGATTAGATACCCTGGTAGTCCATGCCGTAAACGATGAGTGCTAAGTGTTGGGAG
GTTTCCGCCTCTCAGTGCTGCAGCTAACGCATTAAGCACTCCGCCTGGGGAGTACGACCGCAAGGTTGAA
ACTCAAAGGAATTGACGGGGGCCCGCACAAGCGGTGGAGCATGTGGTTTAATTCGAAGCAACGCGAAGAA
CCTTACCAGGTCTTGACATCCTTTGACCACCTAAGAGATTAGTTTTTCCCTTCGGGGACAAAGAGACAGG
TGGTGCATGGCTGTCGTCAGCTCGTGTCGTGAGATGTTGGGTTAAGTCCCGCAACGAGCGCAACCCTTGT
TAATAGTTGCCAGCATTAAGTTGGGCACTCTATTGAGACTGCCGGTGACAAACCGGAGGAAGGTGGGGAT
GACGTCAAGTCATCATGCCCCTTATGACCTGGGCTACACACGTGCTACAATGGGCAGTACAACGAGAAGC
GAACCTGTGAAGGCAAGCGGATCTCTTAAAGCTGTTCTCAGTTCGGACTGTAGGCTGCAACTCGCCTACA
CGAAGCTGGAATCGCTAGTAATCGCGGATCAGCACGCCGCGGTGAATACGTTCCCGGGCCTTGTACACAC
CGCCCGTCACACCATGAGAGTTTGTAACACCCAAAGTCGGTGAGGTAACCTTTGGAGCCAGCCGCCTAAG
GTGGGACAGATGATTAGGGTGAAGTCGTAACAAGGTAACCGTAAAGGGC
>52222145|gb|AY738656.1|Eggerthella|Uncultured Eggerthella sp. clone 123-f2 68 16S ribosomal RNA gene, partial sequence
TGGGGAATATTGCGCAATGGGGGAAACCCTGACGCAGCAACGCCGCGTGCGGGATGAAGGCCTTCGGGTT
GTAAACCGCTTTCAGCAGGGAAGACATCGACGGTACCTGCAGAAGAAGCCCCGGCTAACTACGTGCCAGC
AGCCGCGGTAATACGTAGGGGGCGAGCGTTATCCGGATTCATTGGGCGTAAAGCGCGCGCAGGCGGTTGC
TCAAGCGGAACCTCTAATCTCGGGGCTTAACCTCGAGCCGGGTTCCGAACTGGACGACTCGAGTGCGGTA
GAGGCAGATGGAATTCCCGGTGTAGCGGTGGAATGCGCAGATATCGGGAAGAACACCAACGGCGAAGGCA
GTCTGCTGGGCCGTCACTGACGCTGAGGCGCGAAAGCTGGGGGAGCGAACAGGATTAGATACCCTGGTAG
TCCCAGCCGTAAACGATGAGCGCTGGGTGTGGGAGATTACATCTTCCGTGCCGAAGCTAACGCATTAAGC
GCTCCGCCTGGGGAGTACGGCCGCAAGGCTAAAACTCAAAGGAATTGACGGGGGCCCGCACAAGCAGCGG
AGCATGTGGCTTAATTCGAAGCAACGCGAAGAACCTTACCAGGGCTTGACATGTAGGTGAAGCGGCGGAA
ACGTCGTGGCCGAAAGGAGCCTACACAGGTGGTGCATGGCTGTCGTCAGCTCGTGTCGTGAGATGTTGGG
TTAAGTCCCGCAACGAGCGCAACCCCTGCCCCGTGTTACCAGCATTTAGTTGGGGACTCGCGGGGGACTG
CCGGCGTCAAGCCGGAGGAAGGCGGGGATGACGTCAAGTCATCATGCCCCTTATGCCCTGGGCCGCACAC
GTGCTACAATGGCCGGCACAGCGGGCTGCAACCTAGCGATAGGAAGCGAATCCCGTAAAGCCGGTCCCAG
TTCGGATTGGAGGCTGAAACCCGCCTCCATGAAGCCGGAGTTGCTAGTAATCGCGGATCAGCACGCCGCG
GTGAATGCGTTCCCGGGCCT
>66878729|gb|AY959023.1|Eggerthella|Uncultured bacterium clone rRNA250 16S ribosomal RNA gene, partial sequence
CGCCCTTAGAGTTTGATCCTGGCTCAGGATGAACGCTGGCGGCGTGCCTAACACATGCAAGTCGAACGAT
TAAAGCACCTTCGGGTGTGTATAGAGTGGCGAACGGGTGAGTAACACGTGACCAACCTGCCTCTTACATT
GGGACAACCAAAAGAAATTCTGGCTAATACCAAATACTCCGCACATATCACATGATGTATGCGGGAAAGC
TTTTGCGGTAAGAGATGGGGTCGCGGCCCATTAGGTAGACGGCGGGGTAGAAGCCCACCGTGCCGATGAT
GGGTAGCCGGGTTGAGAGACCGACCGGCCACATTGGGACTGAGATACGGCCCAGACTCCTACGGGAGGCA
GCAGTGGGGAATATTGCGCAATGGGGGAAACCCTGACGCAGCAACGCCGCGTGCGGGATGAAGGCCTTCG
GGTTGTAAACCGCTTTCAGCAGGGAAGACATCGACGGTACCTGCAGAAGAAGCCCCGGCTAACTACGTGC
CAGCAGCCGCGGTAATACGTAGGGGGCGAGCGTTATCCGGATTCATTGGGCGTAAAGCGCGCGCAGGCGG
TTGCTCAAGCGGAACCTCTAATCTCGGGGCTTAACCTCGAGCCGGGTTCCGAACTGGACGACTCGAGTGC

GGTAGAGGCAGATGGAATTCCCGGTGTAGCGGTGGAATGCGCAGATATCGGGAAGAACACCAACGGCGAA
GGCAGTCTGCTGGGCCGTCACTGACGCTGAGGCGCGAAAGCTGGGGGAGCGAACAGGATTAGATACCCTG
GTAGTCCCAGCCGTAAACGATGAGCGCTGGGTGTGGGAGATTACATCTTCCGTGCCGAAGCTAACGCATT
AAGCGCTCCGCCTGGGGAGTACGGCCGCAAGGCTAAAACTCAAAGGAATTGACGGGGGCCCGCACAAGCA
GCGGAGCATGTGGCTTAATTCGAAGCAACGCGAAGAACCTTACCAGGGCTTGACATGTAGGTGAAGCGGC
GGAAACGTCGTGGCCGAAAGGAGCCTACACAGGTGGTGCATGGCTGTCGTCAGCTCGTGTCGTGAGATGT
TGGGTTAAGTCCCGCAACGAGCGCAACCCCTGCCCCGTGTTACCAGCATTTAGTTGGGGACTCGCGGGGG
ACTGCCGGCGTCAAGCCGGAGGAAGGCGGGGATGACGTCAAGTCATCATGCCCCTTATGCCCTGGGCCGC
ACACGTGCTACAATGGCCGGCACAGCGGGCTGCAACCTAGCGATAGGAAGCGAATCCCGTAAAGCCGGTC
CCAGTTCGGATTGGAGGCTGAAACCCGCCTCCATGAAGCCGGAGTTGCTAGTAATCGCGGATCAGCACGC
CGCGGTGAATGCGTTCCCGGGCCTTGTACACACCGCCCGTCACACCACCCGAGTCGTCTGCACCCGAAGC
CGCCGGCCGAACCCCTTTGGGGACGGAGGCGTCGAAGGTGTGGAGGGTGAGGGGGGTGAAGTCGTAACAA
GGTAACCGTAAAGGGC
>66878619|gb|AY958913.1|Eggerthella|Uncultured bacterium clone rRNA140
16S ribosomal RNA gene, partial sequence
CGCCCTTAGAGTTTGATCCTGGCTCAGGATGAACGCTGGCGGCGTGCCTAACACATGCAAGTCGAGCGAT
TAAAGCACCTTCGGGTGTGTATAGAGTGGCGAACGGGTGAGTAACACGTGACCAACCTGCCTCTTACATT
GGGACAACCAAAAGAAATTCTGGCTAATACCAAATACTCCGCACATATCACATGATGTATGCGGGAAAGC
TTTTGCGGTAAGAGATGGGGTCGCGGCCCATTAGGTAGACGGCGGGGTAGAAGCCCACCGTGCCGATGAT
GGGTAGCCGGGTTGAGAGACCGACCGGCCACATTGGGACTGAGATACGGCCCAGACTCCTACGGGAGGCA
GCAGTGGGGAATATTGCGCAATGGGGGAAACCCTGACGCAGCAACGCCGCGTGCGGGATGAAGGCCTTCG
GGTTGTAAACCGCTTTCAGCAGGGAAGACATCGACGGTACCTGCAGAAGAAGCCCCGGCTAACTACGTGC
CAGCAGCCGCGGTAATACGTAGGGGGCGAGCGTTATCCGGATTCATTGGGCGTAAAGCGCGCGCAGGCGG
TTGCTCAAGCGGAACCTCTAATCTCGGGGCTTAACCTCGAGCCGGGTTCCGAACTGGACGACTCGAGTGC
GGTAGAGGCAGATGGAATTCCCGGTGTAGCGGTGGAATGCGCAGATATCGGGAAGAACACCAACGGCGAA
GGCAGTCTGCTGGGCCGTCACTGACGCTGAGGCGCGAAAGCTGGGGGAGCGAACAGGATTAGATACCCTG
GTAGTCCCAGCCGTAAACGATGAGCGCTGGGTGTGGGAGATTACATCTTCCGTGCCGAAGCTAACGCATT
AAGCGCTCCGCCTGGGGAGTACGGCCGCAAGGCTAAAACTCAAAGGAATTGACGGGGGCCCGCACAAGCA
GCGGAGCATGTGGCTTAATTCGAAGCAACGCGAAGAACCTTACCAGGGCTTGACATGTAGGTGAAGCGGC
GGAAACGTCGTGGCCGAAAGGAGCCTACACAGGTGGTGCATGGCTGTCGTCAGCTCGTGTCGTGAGATGT
TGGGTTAAGTCCCGCAACGAGCGCAACCCCTGCCCCGTGTTACTAGCATTTAGTTGGGGACTCGCGGGGG
ACTGCCGGCGTCAAGCCGGAGGAAGGCGGGGATGACGTCAAGTCATCATGCCCCTTATGCCCTGGGCCGC
ACACGTGCTACAATGGCCGGCACAGCGGGCTGCAACCTAGCGATAGGAAGCGAATCCCGTAAAGCCGGTC
CCAGTTCGGATTGGAGGCTGAAACCCGCCTCCATGAAGCCGGAGTTGCTAGTAATCGCGGATCAGCACGC
CGCGGTGAATGCGTTCCCGGGCCTTGTACACACCGCCCGTCACACCACCCGAGTCGTCTGCACCCGAAGC
CGCCGGCCGAACCCCTTTGGGGACGGAGGCGTCGAAGGTGTGGAGGGTGAGGGGGGTGAA
>167736297|dbj|AB379693.1|Eggerthella|Eggerthella sp. YY7918 gene for 16S
rRNA, partial sequence
GAGTTTGATCCTGGCTCAGGATGAACGCTGGCGGCGTGCCTAACACATGCAAGTCGAACGATTAAAGCGC
CTTCGGGCGTGTATAGAGTGGCGAACGGGTGAGTAACACGTGACCAACCTACCCTTCTCTTCGGGACAAC
CTTGGGAAACCGAGGCTAATACCGAATACTCCGGCGAGTGCGCATGCACTCACCGGGAAAGCTTTCGCGG
AGAAGGATGGGGTCGCGGCCCATCAGGTAGACGGCGGGGTAGCGGCCCACCGTGCTTTTGACGGGTAGCC
GGGTTGAGAGACCGACCGGCCACATTGGGACTGAGATACGGCCCAGACTCCTACGGGAGGCAGCAGTGGG
GAATTTTGCGCAATGGGGGAAACCCTGACGCAGCAACGCCGCGTGCGGGATGAAGGCCTTCGGGTCGTAA
ACCGCTTTCAGCAGGGAAGAAATTCGACGGTACCTGCAGAAGAAGCCCCGGCTAACTACGTGCCAGCAGC
CGCGGTAATACGTAGGGGGCGAGCGTTATCCGGATTCATTGGGCGTAAAGCGCGCGTAGGCGGCCGCCTA
AGCGGGACCTCTAACCTTGGGGCTCAACCTCAAGCCGGGTTCCGAACTGGGTGGCTCGAGTTTGGTAGAG
GAAGATGGAATTCCCGGTGTAGCGGTGGAATGCGCAGATATCGGGAAGAACACCGATGGCGAAGGCAGTC
TTCTGGGCCATAACTGACGCTGAGGCGCGAAAGCTAGGGGAGCAAACAGGATTAGATACCCTGGTAGTCC
TAGCCGTAAACGATGGGCACTAGGTGTGGGGAGATACTCTTTCCGTGCCGAAGCAAACGCATTAAGTGCC
CCGCCTGGGGAGTACGGCCGCAAGGCTAAAACTCAAAGGAATTGACGGGGGCCCGCACAAGCAGCGGAGC
ATGTGGCTTAATTCGAAGCAACGCGAAGAACCTTACCAGGGCTTGACATGCGCATGAAGCCGGGGAAACT
CGGTGGCCGAAAGGAGTGCGCGCAGGTGGTGCATGGCTGTCGTCAGCTCGTGTCGTGAGATGTTGGGTTA
AGTCCCGCAACGAGCGCAACCCCTGTCCTGTGTTGCCAGCATTCAGTTGGGGACTCGCAGGAGACTGCCG

GCGTCAAGCCGGAGGAAGGTGGGGACGACGTCAAGTCATCATGCCCCTTATGCCCTGGGCTGCACACGTG
CTACAATGGCCGGCACAACGGGCTGCTACCTGGCGACAGGAAGCGAATCCCTTAAAGCCGGTCCCAGTTC
GGATCGGAGGCTGCAACCCGCCTCCGTGAAGTCGGAGTTGCTAGTAATCGCGGATCAGCATGCCGCGGTG
AATACGTTCCCGGGCCTTGTACACACCGCCCGTCACACCACCCGAGTCGTCTGCACCCGAAGCCGCCGGC
CGAACCCATTTGGGACGGAGGCGTCGAAGGTGTGGAGGGTAAGGGGGGTGAAGTCGTAACAAGGTAGCC
>145286402|gb|EF495247.1|L.rhamnosus|Lactobacillus rhamnosus strain L60
16S ribosomal RNA gene, partial sequence
GCTCAGGATGAACGCNGGCGGCGTGCNTAATACATGCAAGTCGAACGAGTTCTGATTATTGAAAGGTGCT
TGCATCTTGATTTAATTTTGAACGAGTGGCGGACGGGTGAGTAACACGTGGGTAACCTGCCCTTAAGTGG
GGGATAACATTTGGAAACAGATGCTAATACCGCATAAATCCAAGAACCGCATGGTTCTTGGCTGAAAGAT
GGCGTAAGCTATCGCTTTTGGATGGACCCGCGGCGTATTAGCTAGTTGGTGAGGTAACGGCTCACCAAGG
CAATGATACGTAGCCGAACTGAGAGGTTGATCGGCCACATTGGGACTGAGACACGGCCCAAACTCCTACG
GGAGGCAGCAGTAGGGAATCTTCCACAATGGACGCAAGTCTGATGGAGCAACGCCGCGTGAGTGAAGAAG
GCTTTCGGGTCGTAAAACTCTGTTGTTGGAGAAGAATGGTCGGCAGAGTAACTGTTGTCGGCGTGACGGT
ATCCAACCAGAAAGCCACGGCTAACTACGTGCCAGCAGCCGCGGTAATACGTAGGTGGCAAGCGTTATCC
GGATTTATTGGGCGTAAAGCGAGCGCAGGCGGTTTTTTAAGTCTGATGTGAAAGCCCTCGGCTTAACCGA
GGAAGTGCATCGGAAACTGGGAAACTTGAGTGCAGAAGAGGACAGTGGAACTCCATGTGTAGCGGTGAAA
TGCGTAGATATATGGAAGAACACCAGTGGCGAAGGCGGCTGTCTGGTCTGTAACTGACGCTGAGGCTCGA
AAGCATGGGTAGCGAACAGGATTAGATACCCTGGTAGTCCATGCCGTAAACGATGAATGCTAGGTGTTGG
AGGGTTTCCGCCCTTCAGTGCCGCAGCTAACGCATTAAGCATTCCGCCTGGGGAGTACGACCGCAAGGTT
GAAACTCAAAGGAATTGACGGGGGCCCGCACAAGCGGTGGAGCATGTGGTTTAATTCGAAGCAACGCGAA
GAACCTTACCAGGTCTTGACATCTTTTGATCACCTGAGAGATCAGGTTTCCCCTTCGGGGGCAAAATGAC
AGGTGGTGCATGGTTGTCGTCAGCTCGTGTCGTGAGATGTTGGGTTAAGTCCCGCAACGAGCGCAACCCT
TATGACTAGTTGCCAGCATTTAGTTGGGCACTCTAGTAAGACTGCCGGTGACAAACCGGAGGAAGGTGGG
GATGACGTCAAATCATCATGCCCCTTATGACCTGGGCTACACACGTGCTACAATGGATGGTACAACGAGT
TGCGAGACCGCGAGGTCAAGCTAATCTCTTAAAGCCATTCTCAGTTCGGACTGTANGCTGCAACTCGCCT
ACACGAAGTCGGAATCGCTAGTAATCGCGGATCAGCACGCCGCGGTGAATACGTTCCCGGGCCTTGTACA
CA
>63146108|gb|AY995243.1|Finegoldia|Uncultured Finegoldia sp. clone
FX50B4-14 16S ribosomal RNA gene, partial sequence
ACCGGGGATGCTTACCATGCAAGTCGAACGGGAATTAGTAGACAGAAACCTCGGTGGAAGACTACTAATG
AGAGTGGCGAACGGGTGAGTAACGCGTGAGCAACCTGCCTATGACAGAGGGATAGCCTCGGGAAACCGGG
ATTAATACCGCATAAAATCGTAGAAACGCATGTTTCAACGGTCAAAGATTTATCGGTCATAGATGGGCTC
GCGTCTGATTAGCTAGTTGGTGAGATAACAGCCCACCAAGGCGACGATCAGTAGCCGGTCTGAGAGGATG
AACGGCCACATTGGAACTGAGACACGGTCCAAACTCCTACGGGAGGCAGCAGTGGGGAATATTGCACAAT
GGGGGAAACCCTGATGCAGCGACGCCGCGTGAACGAAGAAGGTATTCGTATCGTAAAGTTCTGTCCTATG
GGAAGATAATGACAGTACCATAGAAGAAAGCTCCGGCTAAATACGTGCCAGCAGCCGCGGTAATACGTAT
GGAGCGAGCGTTGTCCGGAATTATTGGGCGTAAAGGGTACGCAGGCGGTTTAGAGAGTCTAATGTTAAAA
ATCGGGGCTCAACCCCGTAAAGCATTAGAAACTATTAAACTTGAGTAGTGGAGAGAAAAGTGGAATTCCT
AGTGTAGTGGTGAAATACGTAGATATTAGGAGGAATACCAGTAGCGAAGGCGACTTTCTGGACACAAACT
GACGCTGAGGTACGAAAGCGTGGGGAGCAAACAGGATTAGATACCCTGGTAGTCCACGCCGTAAACGATG
AATGCTAGGTGTTGGGTGTCAAAGCTCAGTGCCGAAGTTAACACATTAAGCATTCCGCCTGGGGAGTACG
CACGCAAGTGTGAAACTCAAAGGAATTGACGGGGACCCGCACAAGCAGCGGAGCATGTGGTTTAATTCGA
TGCAACGCGAAGAACCTTACCAGGGCTTGACATGTGGGTGAAAGGTATAGAGATATACCCCTCTCTTTAT
GAGACATCCATACAGGTGGTGCATGGTTGTCGTCAGCTCGTGTCGTGAGATGTTGGGTTAAGTCCCGCAA
CGAGCGCAACCCCTATACTTAGTTACCAGCGAGTAAAGTCGGGGACTCTAAGTAGACTGCCGATGACAAA
TCGGAGGAAGGTGGGGATGACGTCAAATCATCATGCCCTTTATGTCCTGGGCTACACACGTGCTACAATG
GTTGGTACAGAGGGCAGCTATATAGTGATATAATGCAAAACTCGAAAGCCAATCCCAGTTCGGATTGTAG
GCTGCAACTCGCCTACATGAAGTCGGAGTTGCTAGTAATCGCGAATCAGAATGACCGCCGGTTTA
>66878758|gb|AY959052.1|Finegoldia|Uncultured bacterium clone rRNA279 16S
ribosomal RNA gene, partial sequence
CGCCCTTAGAGTTTGATCCTGGCTCAGGACGAACGCTGGCGGCGTGCTTAACACATGCAAGTCGAACGGG
ATTTAGTAAACAGAAGCCTCGGTGGAAGATTACTAATGAGAGTGGCGAACGGGTGAGTAACGCGTGAGCA
ACCTGCCTATGACAGTGGGATAGCCTCGGGAAACCGGGATTAATACCGCATAAAATCGTAGAAACACATG

TTTCAACGGTCAAAGATTTATCGGTCATAGATGGGCTCGCGTCTGATTAGCTAGTTGGTGAGATAACAGC
CCACCAAGGCGACGATCAGTAGCCGGTCTGAGAGGATGAACGGCCACATTGGAACTGAGACACGGTCCAA
ACTCCTACGGGAGGCAGCAGTGGGGAATATTGCACAATGGGGGAAACCCTGATGCAGCGACGCCGCGTGA
ACGAAGAAGGTATTCGTATCGTAAAGTTCTGTCCTATGGGAAGATAATGACAGTACCATAGAAGAAAGCT
CCGGCTAAATACGAGCCAGCAGCCGCGGTAATACGTATGGAGCGAGCGTTGTCCGGAATTATTGGGCGTA
AAGGGTACGCAGGCGGTTTAATAAGTCGAATGTTAAAGATCGGGGCTCAACCCCGTAAAGCATTGGAAAC
TGATAAACTTGAGTAGTGGAGAGGAAAGTGGAATTCCTAGTGTAGTGGTGAAATACGTAGATATTAGGAG
GAATACCAGTAGCGAAGGCGACTTTCTGGACACAAACTGACGCTGAGGTACGAAAGCGTGGGGAGCAAAC
AGGATTAGATACCCTGGTAGTCCACGCCGTAAACGATGAATGCTAGGTGTTGGGGGTCAAACCTCGGTGC
CGAAGTTAACACATTAAGCATTCCGCCTGGGGAGTACGCACGCAAGTGTGAAACTCAAAGGAATTGACGG
GGACCCGCACAAGCAGCGGAGCATGTGGTTTAATTCGATGCAACGCGAAGAACCTTACCAGGGCTTGACA
TGTGGGTGAAAGGTATAGAGATATACCCCTCTCTTTATGAGACATCCATACAGGTGGTGCATGGTTGTCG
TCAGCTCGTGTCGTGAGATGTTGGGTTAAGTCCCGCAACGAGCGCAACCCCTATACTTAGTTACCAGCGA
ATAATGTCGGGGACTCTAAGTAGACTGCCGATGACAAATCGGAGGAAGGTGGGGATGACGTCAAATCATC
ATGCCCTTTATGTCCTGGGCTACACACGTGCTACAATGGTTGGTACAGAGGGCAGCTATATAGTGATATA
ATGCAAAACTCGAAAGCCAATCCCAGTTCGGATTGTAGGCTGCAACTCGCCTACATGAAGTCGGAGTTGC
TAGTAATCGCGGATCAGAATGTCGCGGTGAATGCGTTCCCGGGTCTTGTACACACCGCCCGTCACACCAT
GGGAGTTGATAATACCCGAAGCCTGTGACCTAATTGAGGAGCAGTCGAAGGTAGGATTGATGACTGGGGT
GAAGTCGTAACAAGGTAGCCGTAAAGGGCGAATTCG
>183398314|gb|AY958908.2|Finegoldia|Uncultured bacterium clone rRNA135
16S ribosomal RNA gene, partial sequence
TTGAACGCTGGCGGCGTGCTTAACACATACAAGTCGAACGGGATTTAGTAGACAGAAGCCTCGGTGGAAG
ATTACTAATGAGAGTGGCGAACGGGTGAGTAACGCGTGAGCAACCTGCCTATGACAGTGGGATAGCCTCG
GGAAACCGGGATTAATACCGCATAAAATCGTAAAAACACATGTTTCAACGGTCAAAGATTTATCGGTCAT
AGATGGGCTCGCGTCTGATTAGCTAGTTGGTGAGATAACAGCCCACCAAGGCGACGATCAGTAGCCGGTC
TGAGAGGATGAACGGCCACATTGGAACTGAGACACGGTCCAAACTCCTACGGGAGGCAGCAGTGGGGAAT
ATTGCACAATGGGGGAAACCCTGATGCAGCGACGCCGCGTGAACGAAGAAGGTATTCGTATCGTAAAGTT
CTGTCCTATGGGAAGATAATGACAGTACCATAGAAGAAAGCTCCGGCTAAGTACGTGCCAGCAGCCGCGG
TAATACGTATGGAGCGAGCGTTGTCCGGAATTATTGGGCGTAAAGGGTACGCAGGCGGTTTAATAAGTCG
AATGTTAAAGATCGGGGCTCAACCCCGTAAAGCATTGGAAACTGATAAACTTGAGTAGTGGAGAGGAAAG
TGGAATTCCTAGTATAGTGGTGAAATACGTAGATATTAGGAGGAATACCAGTAGCGAAGGCGACTTTCTG
GACACAAACTGACGCTGAGGTACGAAAGCGTGGGGAGCAAACAGGATTAGATACCCTGGTAGTCCACGCC
GTAAACGATGAATGCTAGGTGTTGGGGGTCAAACCTCGGTGCCGAAGTTAACACATTAAGCATTCCGCCT
GGGGAGTACGCACGCAAGTGTGAAACTCAAAGGAATTGACGGGGACCCGCACAAGCAGCGGAGCATGTGG
TTTAATTCGATGCAACGCGAAGAACCTTACCAGGGCTTGACATGTGGGTGAAAGGTATAGAGATATACCC
CTCTCTTTATGAGACATCCATACAGGTGGTGCATGGTTGTCGTCAGCTCGTGTCGTGAGATGTTGGGTTA
AGTCCCGCAACGAGCGCAACCCCTATACTTAGTTACCAGCGAGTAATGTCGGGGACTCTAAGTAGACTGC
CGATGACAAATCGGAGGAAGGTGGGGATGACGTCAAATCATCATGCCCTTTATGTCCTGGGCTACACACG
TGCTACAATGGTTGGTACAGAGGGCAGCTATATAGTGATATAATGCAAAACTCGAAAGCCAATCCCAGTT
CGGATTGTAGGCTGCAACTCGCCTACATGAAGTCGGAGTTGCTAGTAATCGCGGATCAGAATGTCGCGGT
GAATGCGTTCCCGGGTCTTGTACACACCGCCCGTCACACCATGGGAGTTGATAATACCCGAAGCCTGTGA
CCTAATTGAGGAGCAGTCGAAGGTAGGATTGATGACTGGGGTGAAGTCGTAACAAGGTAACCGTA
>63146127|gb|AY995262.1|Moryella|Uncultured bacterium clone FX28B4-3 16S
ribosomal RNA gene, partial sequence
GGGGGGTGCTTACCATGCAAGTCGACGAGAGATTCGAGCGGAAGTTTTCGGACGGAAGAGCGCCATTTCG
AGTGGCGGACGGGTGAGTAACGCGTGGGGAACCTGCCTCATACAGGGGGATACCAGCCGGAAACGGCTGT
TAAAACCGCATAAGACCACAGAGCCGCGTGGCTCAGGGGTAAAAACTCCGGTGGTATGAGATGGACCCGC
GTTCGATTAGCCAGTTGGCGGGGTAACGGCCCACCAAAGCGACGATCGATAGCCGATCTGAGAGGATGAC
CGGCCACATTGGGACTGAGACACGGCCCAAACTCCTACGGGAGGCAGCAGTGGGGAATATTGCACAATGG
AGGGAACTCTGATGCAGCGACGCCGCGTGAGTGAAGAAGTATTTCGGTATGTAAAGCTCTATCAGCAGGG
AAGAAGATGACGGTACCTGAATAAGAAGCCCCGGCTAACTACGTGCCAGCAGCCGCGGTAATACGTAGGG
GGCAAGCGTTATCCGGATTTACTGGGTGTAAAGGGAGCGCAGACGGCTGTGCAAGTCTGAAGTGAAAGCC
CGCGGCTCAACTGCGGGATTGCTTTGGAAACTGTGCGGCTTGAGTATCGGAGAGGTAAGCGGAATTCCAA
GTGTAGCGGTGAAATGCGTAGATATTTGGAGGAACACCGGTGGCGAAGGCGGCTTACTGGACGAAAACTG

ACGTTGAGGCTCGAAGGCGTGGGGAGCAAACAGGATTAGATACCCTGGTAGTCCACGCGGTAAACGATGA
ATACTAGGTGTCGGTGCCCAAAGGGTATCGGTGCCGTCGCAAACGCAATAAGTATTCCACCTGGGGAGTA
CGTTCGCAAGAATGAAACTCAAAGGAATTGACGGGGACCCGCACAAGCAGTGGAGCATGTGGTTTAATTC
GAAGCAACGCGAAGAACCTTACCAAGTCTTGACATCCCGATGACCGGCTCGTAACGGAGCCTTCTCTACG
GAGCAGCGGAGACAGGTGGTGCATGGTTGTCGTCAGCTCGTGTCGTGAGATGTTGGGTTAAGTCCCGCAA
CGAGCGCAACCCCTATTGCCAGTAGCCAGCAGGAGAGCTGGGCACTCTGGCGAGACTGCCCGGGATAACC
GGGAGGAAGGTGGGGATGACGTCAAATCATCATGCCCCTTATGATCTGGGCTACACACGTGCTACAATGG
CGTGAACAAAGGGAAGCGAAGCCGTGAGGTGAAGCAAATCTCAGAAAACACGTCTCAGTTCGGACTGTAG
TCTGCAACTCGACTACACGAAGCTGGAATCGCTAGTAATCGTGGATCAGAATGCCAGCGGAT
>63146123|gb|AY995258.1|Moryella|Uncultured bacterium clone FX2B4-3 16S ribosomal RNA gene, partial sequence
CGGCGGGGGGCTTACACATGCAAGTCGAACGAGAGATCCGGACGGAAGTTTTCGGACGGAAGAGCGGATT
TCGAGTGGCGGACGGGTGAGTAACGCGTGGGGAACCTGCCTCATACAGGGGGATACCAGCCGGAAACGGC
TGTTAAAACCGCATAAGACCACAGAGCCGCATGGCTCAGGGGTAAAAACTCCGGTGGTATGAGATGGACC
CGCGTTCGATTAGCCAGTTGGCGGGGTAACGGCCCACCAAAGCGACGATCGATAGCCGATCTGAGAGGAT
GACCGGCCACATTGGGACTGAGACACGGCCCAAACTCCTACGGGAGGCAGCAGTGGGGAATATTGCACAA
TGGAGGGAACTCTGATGCAGCGACGCCGCGTGAGTGAAGAAGTATTTCGGTATGTAAAGCTCTATCAGCA
GGGAAGAAGATGACGGTACCTGAATAAGAAGCCCCGGCTAACTACGTGCCAGCAGCCGCGGTAATACGTA
GGGGGCAAGCGTTATCCGGATTTACTGGGTGTAAAGGGAGCGCAGACGGCTGTGCAAGTCTGAAGTGAAA
GCCCGCGGCTCAACTGCGGGATTGCTTTGGAAACTGTGCGGCTTGAGTATCGGAGAGGTAAGCGGAATTC
CAAGTGTAGCGGTGAAATGCGTAGATATTTGGGGGAACACCGGTGGCGAAGGCGGCTTACTGGACGAAAA
CTGACGTTGAGGCTCGAAGGCGTGGGGAGCAAACAGGATTAGATACCCTGGTAGTCCACGCGGTAAACGA
TGAATACTAGGTGTCGGTGCCCAAAGGGTATCGGTGCCGTCGCAAACGCAATAAGTATTCCACCTGGGGA
GTACGTTCGCAAGAATGAAACTCAAAGGAATTGACGGGGACCCGCACAAGCAGTGGAGCATGTGGTTTAA
TTCGAAGCAACGCGAAGAACCTTACCAAGTCTTGACATCCCGATGACCGGCTCGTAACGGAGCCTTCTCT
ACGGAGCAGCGGAGACAGGTGGTGCATGGTTGTCGTCAGCTCGTGTCGTGAGATGTTGGGTTAAGTCCCG
CAACGAGCGCAACCCCTATTGCCAGTAGCCAGCAGGAGAGCTGGGCACTCTGGCGAGACTGCCCGGGATA
ACCGGGAGGAAGGTGGGGATGACGTCAAATCATCATGCCCCTTATGATTTGGGCAACACACGTGCTACAA
TGGCGTAAACAGAGGGAAGCAAAGGAGTGATCCGGAGCGAATCCCAAAAATAACGTCTCAGTTCGGACTG
TAGTCTGCAACCCGACTACACGAAGCTGGAATTGCTAGTAATCGCGAATCAGCATGCGCGGTG
>22094834|gb|AF527773.1|Moryella|Moryella indoligenes strain MDA2477 16S ribosomal RNA gene, partial sequence (93%)
GCGTGCTTAACACATGCAAGTCGAACGAGAGATACAGGAGGAAGTTTTCGGATGGAATCCGGTAGATCGA
GTGGCGGACGGGTGAGTAACGCGTGGGGAACCTGCCTCATACAGGGGGATAACAGTTGGAAACGACTGTT
AATACCGCATAAGACCACAGAGTCGCATGACTCAGGGGTAAAAACTCCGGTGGTATGAGATGGACCCGCG
TTCGATTAGCCAGTTGGCGGGGTAACGGCCCACCAAAGCGACGATCGATAGCCGATTTGAGAGAATGACC
GGCCACATTGGGACTGAGACACGGCCCAAACTCCTACGGGAGGCAGCAGTGGGGAATATTGCACAATGGG
GGGAACCCTGATGCAGCGACGCCGCGTGAGTGAAGAAGTATTTCGGTATGTAAAGCTCTATCAGCAGGGA
AGAAGATGACGGTACCTGAGTAAGAAGCCCCGGCTAACTACGTGCCAGCAGCCGCGGTAATACGTAGGGG
GCAAGCGTTATCCGGATTTACTGGGTGTAAAGGGAGCGCAGACGGCTATGCAAGTCTGAAGTGAAAGCCC
GGGGCTCAACCCCGGGACTGCTTTGGAAACTGTGTAGCTAGAGTGTCGGAGAGGTAAGCAGAATTCCCAG
TGTAGCGGTGAAATGCGTAGATATTGGGAAGAATACCGGTGGCGAAGGCGGCTTACTGGACGATAACTGA
CGTTCAGGCTCGAAGGCGTGGGGAGCAAACAGGATTAGATACCCTGGTAGTCCACGCGGTAAACGATGAA
TACTAGGTGTTGGCATCCAAAGGATGTCGGTGCCGTCGCAAACGCAATAAGTATTCCACCTGGGGAGTAC
GTTCGCAAGAATGAAACTCAAAGGAATTGACGGGGACCCGCACAAGCAGTGGAGCATGTGGTTTAATTCG
AAGCAACGCGAAGAACCTTACCAAGTCTTGACATCCCGATGACCGGCCTGTAAAGAGGCCTTCTCTTCGG
AGCATTGGAGACAGGTGGTGCATGGTTGTCGTCAGCTCGTGTCGTGAGATGTTGGGTTAAGTCCCGCAAC
GAGCGCAACCCCTATTGCCAGTAGCCAGCAGGTAAAGCTGGGCACTCTGGCGAGACTGCCTGGGATAACC
AGGAGGAAGGTGGGGATGACGTCAAATCATCATGCCCCTTATGATTTGGGCAACACACGTGCTACAATGG
CGTAAACAGAGAGAAGCGAAGGAGCGATCCGGAGCGAATCTAAAAATAACGTCTCAGTTCGGACTGCAGT
CTGCAACTCGACTGCACGAAGCTGGAATTGCTAGTAATCGCGAATCAGCATGTCGCGGTGAATACGTTCC
CGGGTCTTGTACACACCGCCCGTCACACCATGGGAGTCAGCAACGCCCGAAGTCAGTGACCCAACCTTAA
GGAGGGAGCTGCCGAAGGCGGGGCGGATAACTGGGGTGAAGTCGTAACAAGGTAGCCGTATCGGAAGGTG
CGGTTGGATCACCTCCTAA

>63146124|gb|AY995259.1|Roseburia|Uncultured bacterium clone FX5B4-3 16S ribosomal RNA gene, partial sequence (87%)
GCCGGCGTCTTACCATGCAAGTCGAACGAAGCACCCTGTTTTAATTCCTTCGGGAAAGCGAGCAGGGTGA
CTGAGTGGCGGACGGGTGATTAACACGGGGATAACCTGCCTCTTGGTGGGGGATAACAGTGGGAAACGGC
TGCTATTACCGCATAACACCACAGAGCCGCATGGCTCGGGGGGAAAAACTCCGGTGGTATGAGATGGACC
CGCGTTCGATTAGCCAGTTGGCGGGGTAACGGCCCACCACAGCCACAATCGATAGCCGATCTGATAGGGC
CACCGGCCCCATTGGGACTGATACACGGCCCAGACTCCTACGGGAGGGAGCAGTGGGGAATATTGCACAA
TGGGGGAAACCCTGATGCAGCGACGCCCCGCGAGCGAAAAACTATTTCGGTTTGTAAAGCTCTATCAGCA
GGGAAGATAATGACGGTACCTGGCTAAGAAGCTCCGGCTAAATACGTGCCAGCAGCCGCGGTAATACGTA
TGGAGCAAGCATTATCCGGATTTACTGAGTGTGAAGGGAGCGCATGCGGCATGGCGAGACTGATGTGCAA
TCCCGGGGCTCACCGCCGGCACTGCATTGGAAACTGTCCGGCTAGAGTATCGACAGGGGTACGCGGAGTC
CTAGTGGAATGTGTGAAATGCGTAGATATTAGGACGAACACCGGCGGCGAAGGCGGCTTACTGGTCGATA
ACTGACGCTGAGGCTCGAAAGCGTGGGGAGCAAACAGGATTAGATACTATTGTAGTCCACACCGTAAACG
ATGAATACTAGGTGTTCGCATGCAAGCATGTCAGTGCCGAAGCAAACGCATTAAGTATTCCACCTGGGGA
GTACGTTCGCAAGAATGAAACTCAAAGGAATTGACGGGGACCCGCACAAGCGGTGGAGCATGTGGTTTAA
TTCGAAGCAACGCGAAGAACCTTACCAGGCCTTGACATCCCGATGACCGGTTGGTAACGGGACTTTCTTT
TCGGAGCAGCGGAGACAGGTGGTGCATGGTTGTCGTCAGCTCGTGTCGTGAGATGTTGGTTTAAGTCCCG
CAACGAGCGCAACCCTTGTCCTTAGTAGCCAGCAGTAAGATGGGCACTCTAGGGAGACTCCCGGGGATAA
TCCGGAGGAAGGTGGGGATGACGTCAAATCGTCATGCCCCTTATGGTATGGGCTACACCCGTGTTCCAAT
GGCGTAAACAAAGAGAGGCAAAGCTGTGAAGTGGAGCGAATCTCAAAAATAACGTCCCAGTTCGGATGGT
AGTCTGCAATTCGATTACATGAAGCGGGAATCGCTAGTAATCGCGAATCAGAATGCGCAGATT
>219846801|ref|NR_026393.1|Flackamia|Facklamia hominis strain ATCC 700628 16S ribosomal RNA, partial sequence
CGAACGCTGGCGGCGTGCCTAATACATGCAAGTCGAACGAACGGAGCTAGGAACTGAACCCGGCAAAGTT
AGTGGCGCACGGGTGAGTAACACGTGGAGAACCTACCCTTTTGCGGGGGATAACCATTGGAAACGATGAC
TAATACCGCATAGAATCGCAGATCGCATGATCAACGAAGGAAAGACGGCTTCGGCTGTCGCAAAAGGATG
GCTCCGCGGTGCATTAGCTAGATGGTGGGGTAACGGCCTACCATGGCAATGATGCATAGCCGACCTGAGA
GGGTGATCGGCCACATTGGGACTGAGACACGGCCCAAACTCCTACGGGAGGCAGCAGTAGGGANTCTTCC
GCAATGGGCGCAANNCTGACGGAGCAACGCCGCGTGTGTGAAGAAGGTCTTCGGATCGTAAAGCACTGTT
GTTAGAGAAGAACGACCGCTAGAGTAACTGTTAGCGGAGTGACGGTATCTAACCAGAAAGCCACGGCTAA
CTACGTGCCAGCAGCCGCGGTAATACGTAGGTGGCAAGCGTTGTCCGGATTTATTGGGCGTAAAGGGAGC
GCAGGCGGTGACTTAAGTCTGATGTGAAAGCCCACGGCTCAACCGTGGAGGGTCATTGGAAACTGGGTCA
CTTGAGTNCAGAAGAGGAAAGNGGAATTCCATGTGTAGCGGTGAAATGCGTAGATATATGGAGGAACACC
AGTGGCGAAGGCGACTTTCTGGTCTGTAACTGACGCTGAGGCTCGAAAGCGTGGGGAGCAAACAGGATTA
GATACCCTGGTAGTCCACGCCGTAAACGATGAGTGCTAAGTGTTGGAGGGTTTCCACCCTTCAGTGCTGG
AGTTAACGCAATAAGCACTCCGCCTGGGGAGTACGGCCGCAAGGCTGAAACTCAAAGGAATTGACGGGGA
CCCGCACAAGCGGTGGAGCATGTGGTTTAATTCGAAGCAACGCGAAGAACCTTACCAGGTCTTGACATCT
CTTGCATAGCCTAGAGATAGGTGAAGCCCTTCGGGGCAAGAAGACAGGTGGTGCATGGTTGTCGTCAGCT
CGTGTCGTGAGATGTTGGGTTAAGTCCCGCAACGAGCGCAACCCTTATCACTAGTTGCCAGCATTCAGAT
GGGGACTCTAGTGAGACTGCCGGTGACAAACCGGAGGAAGGTGGGGATGACGTCAAATCATCATGCCCCT
TATGACCTGGGCTACACACGTGCTACAATGGATGGTACAACGAGCAGCGAACTCGCAAGGGCAAGCGAAT
CTCTTAAAGCCATTCTCAGTTCGGATTGTTCTCTGCAACTCGAGAACATGAAGCCGGAATCGCTAGTAAT
CGCGGATCAGCACGCCGCGGTGAATACGTTCCCGGGTCTTGTACACACCGCCCGTCACACCACGAGAGTT
TGTAACACCCGAAGCCGGTGGCCTAACTTTATGAAGCCGTCGAAGGTGGGACAGATGATTGGGGTGAAGT
CGTAACAAGGTAGCCGTATCGGAAGGTGCGGCTGG
>66878569|gb|AY958863.1|Flackamia|Uncultured bacterium clone rRNA090 16S ribosomal RNA gene, partial sequence
CCCTTAGAGTTTGATCCTGGCTCAGGACGAACGCTGGCGGCGTGCCTAATACATGCAAGCCGAACGAACG
GAGCTAGGAACTTGTTCCCGGCAAAGTTAGTGGCGCACGGGTGAGTAACACGTGGAGAACCTACCCTTTT
GCGGGGGATAACCATTGGAAACGATGACTAATACCGCATAGAATCGCAGATCGCATGATCAACGAAGGAA
AGACGGCTTCGGCTGTCGCAAAAGGATGGCTCCGCGGTGCATTAGCTAGATGGTGGGGTAACGGCCTACC
ATGGCAATGATGCATAGCCGACCTGAGAGGGTGATCGGCCACATTGGGACTGAGACACGGCCCAAACTCC
TACGGGAGGCAGCAGTAGGGAATCTTCCGCAATGGGCGCAAGCCTGACGGAGCAACGCCGCGTGTGTGAA
GAAGGTCTTCGGATCGTAAAGCACTGTTGTTAGAGAAGAACGACCGCTAGAGTAACTGTTAGCGGAGTGA

CGGTATCTAACCAGAAAGCCACGGCTAACTACGTGCCAGCAGCCGCGGTAATACGTAGGTGGCAAGCGTT
GTCCGGATTTATTGGGCGTAAAGGGAGCGCAGGCGGTGACTTAAGTCTGATGTGAAAGCCCACGGCTCAA
CCGTGGAGGGTCATTGGAAACTGGGTCACTTGAGTGCAGAAGAGGAAAGTGGAATTCCATGTGTAGCGGT
GAAATGCGTAGATATATGGAGGAACACCAGTGGCGAAGGCGACTTTCTGGTCTGTAACTGACGCTGAGGC
TCGAAAGCGTGGGGAGCAAACAGGATTAGATACCCTGGTAGTCCACGCCGTAAACGATGAGTGCTAAGTG
TTGGAGGGTTTCCACCCTTCAGTGCTGGAGTTAACGCAATAAGCACTCCGCCTGGGGAGTACGGCCGCAA
GGCTGAAACTCAAAGGAATTGACGGGGACCCGCACAAGCGGTGGAGCATGTGGTTTAATTCGAAGCAACG
CGAAGAACCTTACCAGGTCTTGACATCTCTTGCATAGCCTAGAGATAGGTGAAGCCCTTCGGGGCAAGAA
GACAGGTGGTGCATGGTTGTCGTCAGCTCGTGTCGTGAGATGTTGGGTTAAGTCCCGCAACGAGCGCAAC
CCTTATCACTAGTTGCCAGCATTCAGATGGGGACTCTAGTGAGACTGCCGGTGACAAACCGGAGGAAGGT
GGGGATGACGTCAAATCATCATGCCCCTTATGACCTGGGCTACACACGTGCTACAATGGATGGTACAACG
AGCAGCGAACTCGCAAGGGCAAGCGAATCTCTTAAAGCCATTCTCAGTTCGGATTGTTCTCTGCAACTCG
AGAACATGAAGCCGGAATCGCTAGTAATCGCGGATCAGCACGCCGCGGTGAATACGTTCCCGGGTCTTGT
ACACACCGCCCGTCACACCACGAGAGTTTGTAACACCCGAAGCCGGTGGCCTAACTTTATGAGGGAGCCG
TCGAAGGTGGGACAGATGATTGGGGTGAAGTCGTAACAAGGTAACCGTAAAGGGC
>66878725|gb|AY959019.1|Flackamia|Uncultured bacterium clone rRNA246 16S
ribosomal RNA gene, partial sequence
CCCTTAGAGTTTGATCCTGGCTCAGGACGAACGCTGGCGGCGTGCCTAATACATGCAAGTCGAACGAACC
GACGGAGAACTTGTTCTCTTGACGTGAGTGGCGGACGGGTGAGTAACACGTGGGAAACCTACCCTTCAGC
GGGGGATAACGGTTCGAAAGAATCGCTAATACCGCATAAGAGGATCAGTCGCCTGACTGATTCGTAAAAG
ATGGCTCTGCTATCACTGAAGGATGGGCCCGCGGCGCATTAGCTAGTTGGTAAGGTAACGGCTTACCAAG
GCGATGATGCGTAGCCGACCTGAGAGGGTAATCGGCCACATTGGGACTGAGACACGGCCCAAACTCCTAC
GGGAGGCAGCAGTAGGGAATCTTCCGCAATGGACGCAAGTCTGACGGAGCAACGCCGCGTGTGTGAAGAA
GGTTTTCGGATCGTAAAGCACTGTTATTGGCCAAGAACACCCCAAGTAGTAACTGGCTTGGGATTGACGG
TAACCAATCAGAAAGCCACGGCTAACTACGTGCCAGCAGCCGCGGTAATACATAGGTGGCAAGCGTTGTC
CGGATTTATTGGGCGTAAAGGGAGCGCAGGCGGTGACTTAAGTCTGATGTGAAAGCCCACGGCTTAACCG
TGGAGGGTCATTGGAAACTGGGTCACTTGAGTGCAGAAGAGGAAAGCGGAATTCCATGTGTAGCGGTGAA
ATGCGTAGATATATGGAGGAACACCAGTGGCGAAGGCGGCTTTCTGGTCTGTAACTGACGCTGAGGCTCG
AAAGCGTGGGGAGCAAACAGGATTAGATACCCTGGTAGTCCACGCCGTAAACGATGAGTGCTAAGTGTTG
GGGGGCTTCCACCCCTCAGTGCTGGCGTTAACGCAATAAGCACTCCGCCTGGGGAGTACGGTCGCAAGAC
TGAAACTCAAAGGAATTGACGGGGACCCACACAAGCGGTGGAGCATGTGGTTTAATTCGAAGCAACGCGG
AGAACCTTACCAGGTCTTGACATAGGATGCATAGGCTAGAGATAGCTGAAGTCCTTCGGGACATCCATAC
AGGTGGTGCATGGTTGTCGTCAGCTCGTGTCGTGAGATGTTGGGTTAAGTCCCGCAACGAGCGCAACCCT
TATAACTAGTTGCCAGCATTGAGATGGGGACTCTAGTTAGACTGCCGGTGACAAACCGGAGGAAGGTGGG
GATGACGTCAAATCATCATGCCCCTTATGACCTGGGCTACACACGTGCTACAATGGACGATACAACGAGC
AGCGAACTCGCGAGGGCAAGCGAATCTCTGAAAGTCGTTCTCAGTTCGGATTGCAGGCTGCAACTCGCCT
GCATGAAGCCGGAATCGCTAGTAATCGTGGATCAGCACGCCACGGTGAATCCGTTCCCGGGTCTTGTACA
CACCGCCCGTCACACCACGAGAGTTTGTAACACCCGAAGCCGGTGGCCTAACACTGAGGGAGCCGTCGAA
GGTGGGACAGATGATTGGGGTGAA
>157907331|dbj|AB362599.1|E.faecalis|Enterococcus faecalis gene for 16S
rRNA, partial sequence, strain: NRIC 0110
TAGAGTTTGATCATGGCTCAGGACGAACGCTGGCGGCGTGCCTAATACATGCAAGTCGAACGCTTCTTTC
CTCCCGAGTGCTTGCGCTCAATTGGAAAGAGGAGTGGCGGACGGGTGAGTAACACGTGGGTAACCTACCC
ATCAGAGGGGGATAACACTTGGAAACAGGTGCTAATACCGCATAACAGTTTATGCCGCATGGCATAAGAG
TGAAAGGCGCTTTCGGGTGTCGCTGATGGATGGACCCGCGGTGCATTAGCTAGTTGGTGAGGTAACGGCT
CACCAAGGCCACGATGCATAGCCGACCTGAGAGGGTGATCGGCCACACTGGGACTGAGACACGGCCCAGA
CTCCTACGGGAGGCAGCAGTAGGGAATCTTCGGCAATGGACGAAAGTCTGACCGAGCAACGCCGCGTGAG
TGAAGAAGGTTTTCGGATCGTAAAACTCTGTTGTTAGAGAAGAACAAGGACGTTAGTAACTGAACGTCCC
CTGACGGTATCTAACCAGAAAGCCACGGCTAACTACGTGCCAGCAGCCGCGGTAATACGTAGGTGGCAAG
CGTTGTCCGGATTTATTGGGCGTAAAGCGAGCGCAGGCGGTTTCTTAAGTCTGATGTGAAAGCCCCCGGC
TCAACCGGGGAGGGTCATTGGAAACTGGGAGACTTGAGTGCAGAAGAGGAGAGTGGAATTCCATGTGTAG
CGGTGAAATGCGTAGATATATGGAGGAACACCAGTGGCGAAGGCGGCTCTCTGGTCTGTAACTGACGCTG
AGGCTCGAAAGCGTGGGGAGCAAACAGGATTAGATACCCTGGTAGTCCACGCCGTAAACGATGAGTGCTA
AGTGTTGGAGGGTTTCCGCCCTTCAGTGCTGCAGCAAACGCATTAAGCACTCCGCCTGGGGAGTACGACC

GCAAGGTTGAAACTCAAAGGAATTGACGGGGGCCCGCACAAGCGGTGGAGCATGTGGTTTAATTCGAAGC
AACGCGAAGAACCTTACCAGGTCTTGACATCCTTTGACCACTCTAGAGATAGAGCTTTCCCTTCGGGGAC
AAAGTGACAGGTGGTGCATGGTTGTCGTCAGCTCGTGTCGTGAGATGTTGGGTTAAGTCCCGCAACGAGC
GCAACCCTTATTGTTAGTTGCCATCATTTAGTTGGGCACTCTAGCGAGACTGCCGGTGACAAACCGGAGG
AAGGTGGGGATGACGTCAAATCATCATGCCCCTTATGACCTGGGCTACACACGTGCTACAATGGGAAGTA
CAACGAGTCGCTAGACCGCGAGGTCATGCAAATCTCTTAAAGCTTCTCTCAGTTCGGATTGCAGGCTGCA
ACTCGCCTGCATGAAGCCGGAATCGCTAGTAATCGCGGATCAGCACGCCGCGGTGAATACGTTCCCGGGC
CTTGTACACACCGCCCGTCACACCACGAGAGTTTGTAACACCCGAAGTCGGTGAGGTAACCTTTTTGGAG
CCAGCCGCCTAAGGTGGGATAGATGATTGGGGTGAAGTCGTAACAAGGTAGCCGTATCGGAAGGTGCGGT
TGGATCACCTCCTTA
>66878696|gb|AY958990.1|Enterococcus|Uncultured bacterium clone rRNA217
16S ribosomal RNA gene, partial sequence
ACGAACGCTGGCGGCGTGCCTAATACATGCAAGTCGAACGCTTCTTTCCTCCCGAGTGCTTGCGCTCAAT
TGGAAAGAGGAGTGGCGGACGGGTGAGTAACACGTGGGTAACCTACCCATCAGAGGGGGATAACACTTGG
AAACAGGTGCTAATACCGCATAACAGTTTATGCCGCATGGCATAAGAGTGAAAGGCGCTTTCGGGTGTCG
CTGATGGATGGACCCGCGGTGCATTAGCTAGTTGGTGAGGTAACGGCTCACCAAGGCCACGATGCATAGC
CGACCTGAGAGGGTGATCGGCCACACTGGGACTGAGACACGGCCCAGACTCCTACGGGAGGCAGCAGTAG
GGAATCTTCGGCAATGGACGAAAGTCTGACCGAGCAACGCCGCGTGAGTGAAGAAGGTTTTCGGATCGTA
AAACTCTGTTGTTAGAGAAGAACAAGGACGTTAGTAACTGAACGTCCCCTGACGGTATCTAACCAGAAAG
CCACGGCTAACTACGTGCCAGCAGCCGCGGTAATACGTAGGTGGCAAGCGTTGTCCGGATTTATTGGGCG
TAAAGCGAGCGCAGGCGGTTTCTTAAGTCTGATGTGAAAGCCCCCGGCTCAACCGGGGAGGGTCATTGGA
AACTGGGAGACTTGAGTGCAGAAGAGGAGAGTGGAATTCCATGTGTAGCGGTGAAATGCGTAGATATATG
GAGGAACACCAGTGGCGAAGGCGGCTCTCTGGTCTGTAACTGACGCTGAGGCTCGAAAGCGTGGGGAGCA
AACAGGATTAGATACCCTGGTAGTCCACGCCGTAAACGATGAGTGCTAAGTGTTGGAGGGTTTCCGCCCT
TCAGTGCTGCAGCAAACGCATTAAGCACTCCGCCTGGGGAGTACGACCGCAAGGTTGAAACTCAAAGGAA
TTGACGGGGGCCCGCACAAGCGGTGGAGCATGTGGTTTAATTCGAAGCAACGCGAAGAACCTTACCAGGT
CTTGACATCCTTTGACCACTCTAGAGATAGAGCTTTCCCTTCGGGGACAAAGTGACAGGTGGTGCATGGT
TGTCGTCAGCTCGTGTCGTGAGATGTTGGGTTAAGTCCCGCAACGAGCGCAACCCTTATTGTTAGTTGCC
ATCATTTAGTTGGGCACTCTAGCGAGACTGCCGGTGACAAACCGGAGGAAGGTGGGGATGACGTCAAATC
ATCATGCCCCTTATGACCTGGGCTACACACGTGCTACAATGGGAAGTACAACGAGTCGCTAGACCGCGAG
GTCATGCAAATCTCTTAAAGCTTCTCTCAGTTCGGATTGCAGGCTGCAACTCGCCTGCATGAAGCCGGAA
TCGCTAGTAATCGCGGATCAGCACGCCGCGGTGAATACGTTCCCGGGCCTTGTACACACCGCCCGTCACA
CCACGAGAGTTGTAACACCCGAAGTCGGTGAGGTAACCTTTTTGGAGCCAGCCGCCTAAGGTGGGATAGA
TGATTGGGGTGAAGTCGTAACAAGGTAACCGTAAAGGGC
>28274377|gb|AY188353.1|S.vestibularis|Streptococcus vestibularis strain
ATCC 49124 16S ribosomal RNA gene, complete sequence
ATGGGAGAGTTTGATCCTGGCTCAGGACGAACGCTGGCGGCGTGCCTAATACATGCAAGTAGAACGCTGA
AGAGAGGAGCTTGCTCTTCTTGGATGAGTTGCGAACGGGTGAGTAACGCGTAGGTAACCTGCCTTGTAGC
GGGGGATAACTATTGGAAACGATAGCTAATACCGCATAACAATAGGTGACACATGTCATTTATTTGAAAG
GGGCAATTGCTCCACTACAAGATGGACCTGCGTTGTATTAGCTAGTAGGTGAGGTAACGGCTCACCTAGG
CGACGATACATAGCCGACCTGAGAGGGTGATCGGCCACACTGGGACTGAGACACGGCCCAGACTCCTACG
GGAGGCAGCAGTAGGGAATCTTCGGCAATGGGGGCAACCCTGACCGAGCAACGCCGCGTGAGTGAAGAAG
GTTTTCGGATCGTAAAGCTCTGTTGTAAGTCAAGAACGAGTGTGAGAGTGGAAAGTTCACACTGTGACGG
TAGCTTACCAGAAGGGACGGCTAACTACGTGCCAGCAGCCGCGGTAATACGTAGGTCCCGAGCGTTGTCC
GGATTTATTGGGCGTAAAGCGAGCGCAGGCGGTTTGATAAGTCTGAAGTTAAAGGCTGTGGCTCAACCAT
AGTTCGCTTTGGAAACTGTCAAACTTGAGTGCAGAAGGGGAGAGTGGAATTCCATGTGTAGCGGTGAAAT
GCGTAGATATATGGAGGAACACCGGTGGCGAAAGCGGCTCTCTGGTCTGTAACTGACGCTGAGGCTCGAA
AGCGTGGGGAGCGAACAGGATTAGATACCCTGGTAGTCCACGCCGTAAACGATGAGTGCTAGGTGTTGGA
TCCTTTCCGGGATTCAGTGCCGCAGCTAACGCATTAAGCACTCCGCCTGGGGAGTACGACCGCAAGGTTG
AAACTCAAAGGAATTGACGGGGGCCCGCACAAGCGGTGGAGCATGTGGTTTAATTCGAAGCAACGCGAAG
AACCTTACCAGGTCTTGACATCCCGATGCTATTTCTAGAGATAGAAAGTTACTTCGGTACATCGGTGACA
GGTGGTGCATGGTTGTCGTCAGCTCGTGTCGTGAGATGTTGGGTTAAGTCCCGCAACGAGCGCAACCCCT
ATTGTTAGTTGCCATCATTCAGTTGGGCACTCTAGCGAGACTGCCGGTAATAAACCGGAGGAAGGTGGGG
ATGACGTCAAATCATCATGCCCCTTATGACCTGGGCTACACACGTGCTACAATGGTTGGTACAACGAGTT

GCGAGTCGGTGACGGCAAGCTAATCTCTTAAAGCCAATCTCAGTTCGGATTGTAGGCTGCAACTCGCCTA
CATGAAGTCGGAATCGCTAGTAATCGCGGATCAGCACGCCGCGGTGAATACGTTCCCGGGCCTTGTACAC
ACCGCCCGTCACACCACGAGAGTTTGTAACACCCGAAGTCGGTGAGGTAACCTTTTGGAGCCAGCCGCCT
AAGGTGGGATAGATGATTGGGGTGAAGTCGTAACAAGGTAGCCGTATCGGAAGGTGCGGCTGGATCAC
>66878674|gb|AY958968.1|Streptococcus|Uncultured bacterium clone rRNA195
16S ribosomal RNA gene, partial sequence
TCGCCCTTAGAGTTTGATCCTGGCTCAGGACGAACGCTGGCGGCGTGCCTAATACATGCAAGTAGAACGC
TGAAGAGAGGAGCTTGCTCTTCTTGAATGAGTTGCGAACGGGTGAGTAACGCGTAGGTAACCTGCCTTGT
AGCGGGGGATAACTATTGGAAACGATAGCTAATACCGCATAACAATGGGTGACACATGTCATTTATTTG
AAAGGGGCAATTGCTCCACTACAAGATGGACCTGCGTTGTATTAGCTAGTAGGTGAGGTAACGGCTCACC
TAGGCGACGATACATAGCCGACCTGAGAGGGTGATCGGCCACACTGGGACTGAGACACGGCCCAGACTCC
TACGGGAGGCAGCAGTAGGGAATCTTCGGCAATGGGGGCAACCCTGACCGAGCAACGCCGCGTGAGTGAA
GAAGGTTTTCGGATCGTAAAGCTCTGTTGTAAGTCAAGAACGAGTGTGAGAGTGGAAAGTTCACACTGTG
ACGGTAGCTTACCAGAAAGGGACGGCTAACTACGTGCCAGCAGCCGCGGTAATACGTAGGTCCCGAGCGT
TGTCCGGATTTATTGGGCGTAAAGCGAGCGCAGGCGGTTTGATAAGTCTGAAGTTAAAGGCTGTGGCTCA
ACCATAGTTCGCTTTGGAAACTGTCAAACTTGAGTGCAGAAGGGGAGAGTGGAATTCCATGTGTAGCGGT
GAAATGCGTAGATATATGGAGGAACACCGGTGGCGAAAGCGGCTCTCTGGTCTGTAACTGACGCTGAGGC
TCGAAAGCGTGGGGAGCGAACAGGATTAGATACCCTGGTAGTCCACGCCGTAAACGATGAGTGCTAGGTG
TTGGATCCTTTCCGGGACTCAGTGCCGCAGCTAACGCATTAAGCACTCCGCCTGGGGAGTACGACCGCAA
GGTTGAAACTCAAAGGAATTGACGGGGGCCCGCACAAGCGGTGGAGCATGTGGTTTAATTCGAAGCAACG
CGAAGAACCTTACCAGGTCTTGACATCCCGATGCTATTTCTAGAGATAGGAAGTTTCTTCGGAACATCGG
TGACAGGTGGTGCATGGTTGTCGTCAGCTCGTGTCGTGAGATGTTGGGTTAAGTCCCGCAGCGAGCGCAA
CCCTTATTGTTAGTTGCCATCATTGAGTTGGGCACTCTAGCGAGACTGCCGGTAATAAACCGGAGGAAGG
TGGGGATGACGTCGAATCATCATGCCCCTTATGACCTGGGCTACACACGTGCTACAATGGCTGGTACAAC
GAGTCGCAAGCCGGTGACGGCAAGCTAATCTCTGAAAGCCAGTCTCAGTTCGGATTGTAGGCTGCAACTC
GCCTACATGAAGTCGGAATCGCTAGTAATCGCGGATCAGCACGCCGCGGTGAATACGTTCCCGGGCCTTG
TACACACCGCCCGTCACACCACGAGAGTTTGTAACACCCGAAGTCGGTGAGGTAACCGTAAGGAGCCAGC
CGCCTAAGGTGGGATAGATGATTGGGGTGAAGTCGTAACAAGGTAGCCAAG
>23477252|gb|AF459432.1|Streptococcus|Streptococcus agalactiae 16S
ribosomal RNA gene, partial sequence
TAGAGTTTGATCCTGGCTCAGGACGAACGCTGGCGGCGTGCCTAATACATGCAAGTAGAACGCTGAGGTT
TGGTGTTTACACTAGACTGATGAGTTGCGAACGGGTGAGTAACGCGTAGGTAACCTGCCTCATAGCGGGG
GATAACTATTGGAAACGATAGCTAATACCGCATAAGAGTAATTAACACATGTTAGTTATTTAAAAGGAGC
AATTGCTTCACTGTGAGATGGACCTGCGTTGTATTAGCTAGTTGGTGAGGTAAAGGCTCACCAAGGCGAC
GATACATAGCCGACCTGAGAGGGTGATCGGCCACACTGGGACTGAGACACGGCCCAGACTCCTACGGGAG
GCAGCAGTAGGGAATCTTCGGCAATGGACGGAAGTCTGACCGAGCAACGCCGCGTGAGTGAAGAAGGTTT
TCGGATCGTAAAGCTCTGTTGTTAGAGAAGAACGTTGGTAGGAGTGGAAAATCTACCAAGTGAGGGTAAC
TAACCAGAAAGGGACGGCTAACTACGTGCCAGCAGCCGCGGTAATACGTAGGTCCCGAGCGTTGTCCGGA
TTTATTGGGCGTAAAGCGAGCGCAGGCGGTTCTTTAAGTCTGAAGTTAAAGGCAGTGGCTTAACCATTGT
ACGCTTTGGAAACTGGAGGACTTGAGTGCAGAAGGGGAGAGTGGAATTCCATGTGTAGCGGTGAAATGCG
TAGATATATGGAGGAACACCGGTGGCGAAAGCGGCTCTCTGGTCTGTAACTGACGCTGAGGCTCGAAAGC
GTGGGGAGCAAACAGGATTAGATACCCTGGTAGTCCACGCCGTAAACGATGAGTGCTAGGTGTTAGGCCC
TTTCCGGGGCTTAGTGCCGCAGCTAACGCATTAAGCACTCCGCCTGGGGAGTACGACCGCAAGGTTGAAA
CTCAAAGGAATTGACGGGGGCCCGCACAAGCGGTGGAGCATGTGGTTTAATTCGAAGCAACGCGAAGAAC
CTTACCAGGTCTTGACATCCTTCTGACCGGCCTAGAGATAGGCTTTCTCTTCGGAGCAGAAGTGACAGGT
GGTGCATGGTTGTCGTCAGCTCGTGTCGTGAGATGTTGGGTTAAGTCCCGCAACGAGCGCAACCCCTATT
GTTAGTTGCCATCATTAAGTTGGGCACTCTAGCGAGACTGCCGGTAATAAACCGGAGGAAGGTGGGGATG
ACGTCAAATCATCATGCCCCTTATGACCTGGGCTACACACGTGCTACAATGGTTGGTACAACGAGTCGCA
AGCCGGTGACGGCAAGCTAATCTCTTAAAGCCAATCTCAGTTCGGATTGTAGGCTGCAACTCGCCTACAT
GAAGTCGGAATCGCTAGTAATCGCGGATCAGCACGCCGCGGTGAATACGTTCCCGGGCCTTGTACACACC
GCCCGTCACACCACGAGAGTTTGTAACACCCGAAGTCGGTGAGGTAACCTTTTAGGAGCCAGCCGCCTAA
GGTGGGATAGATGATTGGGGTGAAGTCGTAACAAGGTAGCCGTATCGGAAGGTGCGGCTGGATCACCT
>66878711|gb|AY959005.1|Streptococcus|Uncultured bacterium clone rRNA232
16S ribosomal RNA gene, partial sequence

CGCCCTTAGAGTTTGATCCTGGCTCAGGACGAACGCTGGCGGCGTGCCTAATACATGCAAGTAGAACGCT
GAGGTTTGGTGTTTACACTAGACTGATGAGTTGCGAACGGGTGAGTAACGCGTAGGTAACCTGCCTCATA
GCGGGGGATAACTATTGGAAACGATAGCTAATACCGCATAAGAGTAATTAACACATGTTAGTTATTTAAA
AGGAGCAATTGCTTCACTGTGAGATGGACCTGCGTTGTATTAGCTAGTTGGTGAGGTAAAGGCTCACCAA
GGCGACGATACATAGCCGACCTGAGAGGGTGATCGGCCACACTGGGACTGAGACACGGCCCAGACTCCTA
CGGGAGGCAGCAGTAGGGAATCTTCGGCAATGGACGGAAGTCTGACCGAGCAACGCCGCGTGAGTGAAGA
AGGTTTTCGGATCGTAAAGCTCTGTTGTTAGAGAAGAACGTTGGTAGGAGTGGAAAATCTACCAAGTGAC
GGTAACTAACCAGAAAGGGACGGCTAACTACGTGCCAGCAGCCGCGGTAATACGTAGGTCCCGAGCGTTG
TCCGGATTTATTGGGCGTAAAGCGAGCGCAGGCGGTTCTTTAAGTCTGAAGTTAAAGGCAGTGGCTTAAC
CATTGTACGCTTTGGAAACTGGAGGACTTGAGTGCAGAAGGGGAGAGTGGAATTCCATGTGTAGCGGTGA
AATGCGTAGATATATGGAGGAACACCGGTGGCGAAAGCGGCTCTCTGGTCTGTAACTGACGCTGAGGCTC
GAAAGCGTGGGGAGCAAACAGGATTAGATACCCTGGTAGTCCACGCCGTAAACGATGAGTGCTAAGTGTT
AGGCCCTTTCCGGGGCTTAGTGCCGCAGCTAACGCATTAAGCACTCCGCCTGGGGAGTACGACCGCAAGG
TTGAAACTCAAAGGAATTGACGGGGGCCCGCACAAGCGGTGGAGTATGTGGTTTAATTCGAAGCAACGCG
AAGAACCTTACCAGGTCTTGACATCCTTCTGACCGGCCTAGAGATAGGCTTTCTCTTCGGAGCAGAAGTG
ACAGGTGGTGCATGGTTGTCGTCAGCTCGTGTCGTGAGATGTTGGGTTAAGTCCCGCAACGAGCGCAACC
CCTATTGTTAGTTGCCATCATTAAGTTGGGCACTCTAGCGAGACTGCCGGTAATAAACCGGAGGAAGGTG
GGGATGACGTCAAATCATCATGCCCCTTATGACCTGGGCTACACACGTGCTACAATGGTTGGTACAACGA
GTCGCAAGCCGGTGACGGCAAGCTAATCTCTTAAAGCCAATCTCAGTCCGGATTGTAGGCTGCAACTCGC
CTACATGAAGTCGGAATCGCTAGTAATCGCGGATCAGCACGCCGCGGTGAATACGTTCCCGGGCCTTGTA
CACACCGCCCGTCACACCACGAGAGTTTGTAACACCCGAAGTCGGTGAGGTAACCTTTTAGGAGCCAGCC
GCCTAAGGTGGGATAGATGATTGGGGTGAAGTCGTAACAAGGTAACCGTAAAGGGC
>62530458|gb|AY986764.1|S.anginosus|Streptococcus anginosus strain ChDC YA12 16S ribosomal RNA gene gene, partial sequence
AGAGTTTGATCCTGGCTCAGGACGAACGCTGGCGGCGTGCCTAATACATGCAAGTAGGACGCACAGTTTA
TACCGTAGCTTGCTACACCATAGACTGTGAGTTGCGAACGGGTGAGTAACGCGTAGGTAACCTACCTATT
AGAGGGGGATAACTATTGGAAACGATAGCTAATACCGCATAACAGTATGTAACACATGTTAGATGCTTGA
AAGATGCAATTGCATCGCTAGTAGATGGACCTGCGTTGTATTAGCTAGTAGGTAGGGTAAAGGCCTACCT
AGGCAACGATACATAGCCGACCTGAGAGGGTGATCGGCCACACTGGGACTGAGACACGGCCCAGACTCCT
ACGGGAGGCAGCAGTAGGGAATCTTCGGCAATGGGGGGAACCCTGACCGAGCAACGCCGCGTGAGTGAAG
AAGGTTTTCGGATCGTAAAGCTCTGTTGTTAAGGAAGAACGAGTGTGAGAATGGAAAGTTCATGCTGTGA
CGGTACTTAACCAGAAAGGGACGGCTAACTACGTGCCAGCAGCCGCGGTAATACGTAGGTCCCGAGCGTT
GTCCGGATTTATTGGGCGTAAAGCGAGCGCAGGCGGTTAGAAAAGTCTGAAGTGAAAGGCAGTGGCTCAA
CCATTGTAGGCTTTGGAAACTGTTTAACTTGAGTGCAGAAGGGGAGAGTGGAATTCCATGTGTAGCGGTG
AAATGCGTAGATATATGGGGGAACACCGGTGGCGAAAGCGGCTCTCTGGTCTGTAACTGACGCTGAGGCT
CGAAAGCGTGGGGAGCGAACAGGATTAGATACCCTGGTAGTCCACGCCGTAAACGATGAGTGCTAGGTGT
TAGGTCCTTTCCGGGACTTAGTGCCGCAGCTAACGCATTAAGCACTCCGCCTGGGGAGTACGACCGCAAG
GTTGAAACTCAAAGGAATTGACGGGGGCCCGCACAAGCGGTGGAGCATGTGGTTTAATTCGAAGCAACGC
GAAGAACCTTACCAGGTCTTGACATCCCAGTGACCGCCCTAGAGATAGGGTTTCCCTTCGGGGCACTGGT
GACAGGTGGTGCATGGTTGTCGTCAGCTCGTGTCGTGAGATGTTGGGTTAAGTCCCGCAACGAGCGCAAC
CCTTATTGTTAGTTGCCATCATTAAGTTGGGCACTCTAGCGAGACTGCCGGTAATAAACCGGAGGAAGGT
GGGGATGACGTCAAATCATCATGCCCCTTATGACCTGGGCTACACACGTGCTACAATGGCTGGTACAACG
AGTCGCAAGCCGGTGACGGCAAGCTAATCTCTGAAAGCCAGTCTCAGTTCGGATTGTAGGCTGCAACTCG
CCTACATGAAGTCGGAATCGCTAGTAATCGCGGATCAGCACGCCGCGGTGAATACGTTCCCGGGCCTTGT
ACACACCGCCCGTCACACCACGAGAGTTTGTAACACCCGAAGTCGGTGAGGTAACCGTAAGGAGCCAGCC
GCCTAAGGTGGGATAGATGATTGGGGTGAAGTCGTAACAAGGTAACCGTA
>66878700|gb|AY958994.1|Streptococcus|Uncultured bacterium clone rRNA221 16S ribosomal RNA gene, partial sequence
GCCCTTAGAGTTTGATCCTGGCTCAGGACGAACGCTGGCGGCGTGCCTAATACATGCAAGTAGGACGCAC
AGTTTATACCGTAGCTTGCTACACCATAGACTGTGAGTTGCGAACGGGTGAGTAACGCGTAGGTAACCTA
CCTATTAGAGGGGGATAACTATTGGAAACGATAGCTAATACCGCATAACAGTATGTAACACATGTTAGAT
GCTTGAAAGATGCAATTGCATCGCTAGTAGATGGACCTGCGTTGTATTAGCTAGTAGGTAGGGTAATGGC
CTACCTAGGCAACGATACATAGCCGACCTGAGAGGGTGATCGGCCACACTGGGACTGAGACACGGCCCAG
ACTCCTACGGGAGGCAGCAGTAGGGAATCTTCGGCAATGGGGGGAACCCTGACCGAGCAACGCCGCGTGA

GTGAAGAAGGTTTTCGGATCGTAAAGCTCTGTTGTTAAGGAAGAACGAGTGTGAGAATGGAAAGTTCATG
CTGTGACGGTACTTAACCAGAAAGGGACGGCTAACTACGTGCCAGCAGCCGCGGTAATACGTAGGTCCCG
AGCGTTGTCCGGATTTATTGGGCGTAAAGCGAGCGCAGGCGGTTAGAAAAGTCTGAAGTGAAAGGCAGTG
GCTCAACCATTGTAGGCTTTGGAAACTGTTTAACTTGAGTGCAGAAGGGGAGAGTGGAATTCCATGTGTA
GCGGTGAAATGCGTAGATATATGGGGGAACACCGGTGGCGAAAGCGGCTCTCTGGTCTGTAACTGACGCT
GAGGCTCGAAAGCGTGGGGAGCGAACAGGATTAGATACCCTGGTAGTCCACGCCGTAAACGATGAGTGCT
AGGTGTTAGGTCCTTTCCGGGACTTAGTGCCGCAGCTAACGCATTAAGCACTCCGCCTGGGGAGTACGAC
CGCAAGGTTGAAACTCAAAGGAATTGACGGGGGCCCGCACAAGCGGTGGAGCATGTGGTTTAATTCGAAG
CAACGCGAAGAACCTTACCAGGTCTTGACATCCCAGTGACTGCCCTAGAGATAGGGTTTCCCTTCGGGGC
ACTGGTGACAGGTGGTGCATGGTTGTCGTCAGCTCGTGTCGTGAGATGTTGGGTTAAGTCCCGCAACGAG
CGCAACCCTTATTGTTAGTTGCCATCATTGAGTTGGGCACTCTAGCGAGACTGCCGGTAATAAACCGGAG
GAAGGTGGGGATGACGTCAAATCATCATGCCCCTTATGACCTGGGCTACACACGTGCTACAATGGCTGGT
ACAACGAGTCGCAAGCCGGTGACGGCAAGCTAATCTCTGAAAGCCAGTCTCAGTTCGGATTGTAGGCTGC
AACTCGCCTACATGAAGTCGGAATCGCTAGTAATCGCGGATCAGCACGCCGCGGTGAATACGTTCCCGGG
CCTTGTACACACCGCCCGTCACACCACGAGAGTTTGTAACACCCGAAGTCGGTGAGGTAACCGTAAGGAG
CCAGCCGCCTAAGGTGGGATAGATGATTGGGGTGAA
>325651809|dbj|AB618791.1|B.fragilis|Bacteroides fragilis gene for 16S ribosomal RNA, partial cds, strain: JCM 17585
AGAGTTTGATCCTGGCTCAGGATGAACGCTAGCTACAGGCTTAACACATGCAAGTCGAGGGGCATCAGGA
AGAAAGCTTGCTTTCTTTGCTGGCGACCGGCGCACGGGTGAGTAACACGTATCCAACCTGCCCTTTACTC
GGGGATAGCCTTTCGAAAGAAAGATTAATACCCGATAGCATAATGATTCCGCATGGTTTCATTATTAAAG
GATTCCGGTAAAGGATGGGGATGCGTTCCATTAGGTTGTTGGTGAGGTAACGGCTCACCAAGNCTTCGAT
GGATAGGGGTTCTGAGAGGAAGGTCCCCCACATTGGAACTGAGACACGGTCCAAACTCCTACGGGAGGCA
GCAGTGAGGAATATTGGTCAATGGGCGCTAGCCTGAACCAGCCAAGTAGCGTGAAGGATGAAGGCTCTAT
GGGTCGTAAACTTCTTTTATATAAGAATAAAGTGCAGTATGTATACTGTTTTGTATGTATTATATGAATA
AGGATCGGCTAACTCCGTGCCAGCAGCCGCGGTAATACGGAGGATCCGAGCGTTATCCGGATTTATTGGG
TTTAAAGGGAGCGTAGGTGGACTGGTAAGTCAGTTGTGAAAGTTTGCGGCTCAACCGTAAAATTGCAGTT
GATACTGTCAGTCTTGAGTACAGTAGAGGTGGGCGGAATTCGTGGTGTAGCGGTGAAATGCTTAGATATC
ACGAAGAACTCCGATTGCGAAGGCAGCTCACTGGACTGCAACTGACACTGATGCTCGAAAGTGTGGGTAT
CAAACAGGATTAGATACCCTGGTAGTCCACACAGTAAACGATGAATACTCGCTGTTTGCGATATACAGTA
AGCGGCCAAGCGAAAGCATTAAGTATTCCACCTGGGGAGTACGCCGGCAACGGTGAAACTCAAAGGAATT
GACGGGGGCCCGCACAAGCGGAGGAACATGTGGTTTAATTCGATGATACGCGAGGAACCTTACCCGGGCT
TAAATTGCAGTGGAATGATGTGGAAACATGTCAGTGAGCAATCACCGCTGTGAAGGTGCTGCATGGTTGT
CGTCAGCTCGTGCCGTGAGGTGTCGGCTTAAGTGCCATAACGAGCGCAACCCTTATCTTTAGTTACTAAC
AGGTTATGCTGAGGACTCTAGAGAGACTGCCGTCGTAAGATGTGAGGAAGGTGGGGATGACGTCAAATCA
GCACGGCCCTTACGTCCGGGGCTACACACGTGTTACAATGGGGGGTACAGAAGGCAGCTAGCGGGTGACC
GTATGCTAATCCCAAAAGCCTCTCTCAGTTCGGATCGAAGTCTGCAACCCGACTTCGTGAAGCTGGATTC
GCTAGTAATCGCGCATCAGCCACGGCGCGGTGAATACGTTCCCGGGCCTTGTACACACCGCCCGTCAAGC
CATGGGAGCCGGGGGTACCTGAAGTACGTAACCGCAAGGATCGTCCTAGGGTAAAACTGGTGACTGGGGC
TAAGTCGTAACAAGGTAACC
>66878591|gb|AY958885.1|Bacteroides|Uncultured bacterium clone rRNA112 16S ribosomal RNA gene, partial sequence
AGTTTGATCCTGGCTCAGGATGAACGCTAGCTACAGGCTTAACACATGCAAGTCGAGGGGCATCAGGAAG
AAAGCTTGCTTTCTTTGCTGGCGACCGGCGCACGGGTGAGTAACACGTATCCAACCTGCCCTTTACTCGG
GGATAGCCTTTCGAAAGAAAGATTAATACCCGATAGCATAATGATTCCGCATGGTTTCATTATTAAAGGA
TTCCGGTAAAGGATGGGGATGCGTTCCATTAGGTTGTTGGTGAGGTAACGGCCCACCAAGCCTTCGATGG
ATAGGGGTTCTGAGAGGAAGGTCCCCCACATTGGAACTGAGACACGGTCCAAACTCCTACGGGAGGCAGC
AGTGAGGAATATTGGTCAATGGGCGTTAGCCTGAACCAGCCAAGTAGCGTGAAGGATGAAGGCTCTATGG
GTCGTAAACTTCTTTTATATAAGAATAAAGTGCAGTATGTATACTGTTTTGTATGTATTATATGAATAAG
GATCGGCTAACTCCGTGCCAGCAGCCGCGGTAATACGGAGGATCCGAGCGTTATCCGGATTTATTGGGTT
TAAAGGGAGCGTAGGTGGACTGGTAAGTCAGTTGTGAAAGTTTGCGGCTCAACCGTAAAATTGCAGTTGA
TACTGTCAGTCTTGAGTACAGTAGAGGTGGGCGGAATTCGTGGTGTAGCGGTGAAATGCTTAGATATCAC
GAAGAACTCCGATTGCGAAGGCAGCTCACTGGACTGCAACTGACACTGATGCTCGAAAGTGTGGGTATCA
AACAGGATTAGATACCCTGGTAGTCCACACAGTAAACGATGAATACTCGCTGTTTGCGATATACAGTAAG

CGGCCAAGCGAAAGCATTAAGTATTCCACCTGGGGAGTACGCCGGCAACGGTGAAACTCAAAGGAATTGA
CGGGGGCCCGCACAAGCGGAGGAACATGTGGTTTAATTCGATGATACGCGAGGAACCTTACCCGGGCTTA
AATTGCAGTGGAATGATGTGGAAACATGTCAGTGAGCAATCACCACTGTGAAGGTGCTGCATGGTTGTCG
TCAGCTCGTGCCGTGAGGTGTCGGCTTAAGTGCCATAACGAGCGCAACCCTTATCTTTAGTTACTAACAG
GTTATGCTGAGGACTCTGGAGAGACTGCCGTCGTAAGATGTGAGGAAGGTAGGGATGACGTCAAATCAGC
ACGGCCCTTACGTCCGGGGCTACACACGTGTTACAATGGGGGGTACAGAAGGCAGCTAGCGGGTGACCGT
ATGCTAATCCCAAAAGCCTCTCTCAGTTCGGATCGAAGTCTGCAACCCGACTTCGTGAAGCTGGATTCGC
TAGTAATCGCGCATCAGCCACGGCGCGGTGAATACGTTCCCGGGCCTTGTACACACCGCCCGTCAAGCCA
TGGGAGCCGGGGGTACCTGAAGTACGTAACCGCAAGGATCGTCCTAGGGTAAAACTGGTGACTGGGGCTA
AGTCGTAACAAGGTAACCGTAAAGGGCG
>66878483|gb|AY958777.1|Bergeylla|Uncultured bacterium clone rRNA004 16S
ribosomal RNA gene, partial sequence
CCCTTAGAGTTTGATCCTGGCTCAGGATGAACGCTAGCGGGAGGCCTAACACATGCAAGCCGAGCGGGAT
TTATTGGTTAGCTTGCTAACAGATAATGAGAGCGGCGTACGGGTGCGTAACATGTGTGCAATCTGCCCTT
ATCAAGGGGATAGCCCGGAGAAATCCGGATTAATACCCTATAATATATGGGATGGCATCATTTCATATTG
AAAGCATTAGCGGATAAGGATGAGCACGCACAAGATTAGCTAGTTGGTAAGGTAACGGCTTACCAAGGCG
ATGATCTTTAGGGGTCCTGAGAGGGAGATCCCCCACACTGGGACTGAGACACGGCCCAGACTCCTACGGG
AGGCAGCAGTGAGGAATATTGGACAATGGGTGTAAGCCTGATCCAGCCATCCCGCGTGAAGGAAGACGGT
TCTATGGATTGTAAACTTCTTTTATACAGGGATAAACCTACCCTCGTGAGGGTAGCTGAAGGTACTGTAT
GAATAAGCACCGGCTAACTCCGTGCCAGCAGCCGCGGTAATACGGAGGGTGCAAGCGTTATCCGGATTTA
TTGGGTTTAAAGGGTCCGTAGGCGGGCTGATAAGTCAGTGGTGAAATCCTGCAGCTTAACTGTAGAACTG
CCATTGATACTGTTAGTCTTGAGTATATTTGAAGTAGCTGGAATAAGTAGTGTAGCGGTGAAATGCATAG
ATATTACTTAGAACACCAATTGCGAAGGCAGGTTACTAAGATAAGACTGACGCTGAGGGACGAAAGCGTG
GGGAGCGAACAGGATTAGATACCCTGGTAGTCCACGCTGTAAACGATGCTGACTCGTTTTTGGTTTTTAG
GGATCAGAGACTAAGCGAAAGTGATAAGTCGGCCACCTGGGGAGTACGACCGCAAGGTTGAAACTCAAAG
GAATTGACGGGGGCCCGCACAAGCGGTGGATTATGTGGTTTAATTCGATGATACGCGAGGAACCTTACCA
AGACTTAAATGGGAATTGACAGCTGTAGAAATACGGTTTTCTTCGGACAATTTTCAAGGTGCTGCATGGT
TGTCGTCAGCTCGTGCCGTGAGGTGTTAGGTTAAGTCCTGCAACGAGCGCAACCCCTGTCACTAGTTGCC
ATCATTAAGTTGGGGACTCTAGTGAGACTGCCTGCGCAAGCAGAGAGGAAGGTGGGGATGACGTCAAATC
ATCACGGCCCTTACGTCTTGGGCCACACACGTAATACAATGGCCGGTACAGAGGGCAGCTACTATGTGAA
TAGATGCGAATCTCGAAAGCCGGTCTCAGTTCGGATTGGAGTCTGCAACTCGACTCTATGAAGCTGGAAT
CGCTAGTAATCGCGCATCAGCCATGGCGCGGTGAATACGTTCCCGGGCCTTGTACACACCGCCCGTCAAG
CCATGGAAGTTCGGGGTACCTGAAGTCGGTGACCGTAAAAGGAGCTGCCTAGGGTAAAACGAGTAACTAG
GGCTAAGTCGTAACAAGGTAGCCGTAAAGGGCG
>307816497|gb|HQ154560.1|Elizabethkingia|Elizabethkingia meningoseptica
strain R3-4A 16S ribosomal RNA gene, partial sequence
CCTGGTTTGATCCTGGCTCAGGATGAACGCTAGCGGGAGGCCTAACACATGCAAGCCGAGCGGTAGAGAT
TCTTCGGGATCTTGAGAGCGGCGTACGGGTGCGGAACACGTGTGCAACCTGCCTTTATCAAGGGGATAGC
CTTTCGAAAGGAAGATTAATACCCTATAATATATGATTCGGCATCGGATTATATTGAAAACTACGGTGGA
TAAAGATGGGCACGCGCAAGATTAGCTAGTTGGTGAGGTAACGGCTCACCAAGGCGACGATCTTTAGGGG
GCCTGAGAGGGTGATCCCCCACACTGGTACTGAGACACGGACCAGACTCCTACGGGAGGCAGCAGTGAGG
AATATTGGACAATGGGTGGAAGCCTGATCCAGCCATCCCGCGTGTAGGAAGACGGCCCTATGGGTTGTAA
ACTACTTTTATCTGGGGATAAACCTACTTACGTGTAAGTAGCTGAAGGTACCAGATGAATAAGCACCGGC
TAACTCCGTGCCAGCAGCCGCGGTAATACGGAGGGTGCAAGCGTTATCCGGATTTATTGGGTTTAAAGGG
TCCGTAGGCGGACTGATAAGTCAGTGGTGAAATCCGACAGCTTAACTGTCGAACTGCCATTGATACTGTT
AGTCTTGAGTAAGGTTGAAGTGGCTGGAATAAGTAGTGTAGCGGTGAAATGCATAGATATTACTTAGAAC
ACCAATTGCGAAGGCAGGTCACTAAGTCTTAACTGACGCTGATGGACGAAAGCGTGGGGAGCGAACAGGA
TTAGATACCCTGGTAGTCCACGCCGTAAACGATGATTACTCGTTTTTGGGTTTTAGGATTCAGAGACTAA
GCGAAAGTGATAAGTAATCCACCTGGGGAGTACGTTCGCAAGAATGAAACTCAAAGGAATTGACGGGGGC
CCGCACAAGCGGTGGAGCATGTGGTTTAATTCGATGATACGCGAGGAACCTTACCAAGACTTAAATGGGA
AATGACAGATTTAGAAATAGATCCTTCTTCGGACATTTTTCAAGGTGCTGCATGGTTGTCGTCAGCTCGT
GCCGTGAGGTGTTAGGTTAAGTCCTGCAACGAGCGCAACCCCTGTCACTAGTTGCTAACATTAAGTTGAG
GACTCTAGTGAGACTGCCTACGCAAGTAGAGAGGAAGGTGGGGATGACGTCAAATCATCACGGCCCTTAC
GTCTTGGGCCACACACGTGCTACAATGGCCGGTACAGAGGGCAGCTACCTAGTGATAGGATGCAAATCTC

GAAAGCCGGTCTCAGTTCGGATTGGAGTCTGCAACTCGACTCTATGAAGCTGGAATCGCTAGTAATCGCG
CATCAGCCATGGCGCGGTGAATACGTTCCCGGGCCTTGTACACACCGCCCGTCAAGCCATGGAAGCTGGG
GGTACCTGAAGTCGGTGACCGTAAAAGGAGCTGCCTAGGGTAAAACTAGTAACTAGGGCTAAGTCGTACA
GGGTAGCCGTACA
>66878795|gb|AY959089.1|Elizabethkingia|Uncultured bacterium clone
rRNA316 16S ribosomal RNA gene, partial sequence
CCCTTAGAGTTTGATCCTGGCTCAGGATGAACGCTAGCGGGAGGCCTAACACATGCAAGCCGAGCGGTAG
AGATTCTTCGGAATCTTGAGAGCGGCGTACGGGTGCGGAACACGTGTGCAACCTGCCTTTATCAAGGGGA
TAGCCTTTCGAAAGGAAGATTAATACCCTATAATATATGATTCGGCATCGGATTATATTGAAAACTACGG
TGGATAAAGATGGGCACGCGCAAGATTAGCTAGTTGGTGAGGTAACGGCTCACCAAGGCGACGATCTTTA
GGGGGCCTGAGAGGGTGATCCCCCACACTGGTACTGAGACACGGACCAGACTCCTACGGGAGGCAGCAGT
GAGGAATATTGGACAATGGGTGGAAGCCTGATCCAGCCATCCCGCGTGTAGGAAGACGGCCCTATGGGTT
GTAAACTACTTTTATCTGGGGATAAACCTACTTACGTGTAAGTAGCTGAAGGTACCAGATGAATAAGCAC
CGGCTAACTCCGTGCCAGCAGCCGCGGTAATACGGAGGGTGCAAGCGTTATCCGGATTTATTGGGTTTAA
AGGGTCCGTAGGCGGGCTTATAAGTCAGTGGTGAAATCCGACAGCTTAACTGTCGAACTGCCATTGATAC
TGTGAGCCTTGAGTAAGGTTGAAGTGGCTGGAATAAGTAGTGTAGCGGTGAAATGCATAGATATTACTTA
GAACACCAATTGCGAAGGCAGGTCACTAAGTCTTAACTGACGCTGATGGACGAAAGCGTGGGGAGCGAAC
AGGATTAGATACCCTGGTAGTCCACGCCGTAAACGATGATTACTCGTTTTTGGGTTTTAGGATTCAGAGA
CTAAGCGAAAGTGATAAGTAATCCACCTGGGGAGTACGTTCGCAAGAATGAAACTCAAAGGAATTGACGG
GGGCCCGCACAAGCGGTGGAGCATGTGGTTTAATTCGATGATACGCGAGGAACCTTACCAAGACTTAAAT
GGGAAATGACAGATTTAGAAATAGATCCTTCTTCGGACATTTTTCAAGGTGCTGCATGGTTGTCGTCAGC
TCGTGCCGTGAGGTGTTAGGTTAAGTCCTGCAACGAGCGCAACCCCTGTCACTAGTTGCTAACATTAAGT
TGAGGACTCTAGTGAGACTGCCTACGCAAGTAGAGAGGAAGGTGGGGATGACGTCAAATCATCACGGCCC
TTACGTCTTGGGCCACACACGTGCTACAATGGCCGGTACAGAGGGCAGCTACCTAGTGATAGGATGCAAA
TCTCGAAAGCCGGTCTCAGTTCGGATTGGAGTCTGCAACTCGACTCTATGAAGCTGGAATCGCTAGTAAT
CGCGCATCAGCCATGGCGCGGTGAATACGTTCCCGGGCCTTGTACACACCGCCCGTCAAGCCATGGAAGC
TGGGGGTACCTGAAGTCGGTGACCGTAAAAGGAGCTGCCTAGGGTAAAACTAGTAACTAGGGCTAAGTCG
TAACAAGGTAGCCGTAAAGGGC
>66878561|gb|AY958855.1|Sphingobacteria|Uncultured bacterium clone
rRNA082 16S ribosomal RNA gene, partial sequence
GCCCTTAGAGTTTGATCCTGGCTCAGGATGAACGCTAGCGGCAGGCTTAATACATGCAAGTCGAGGGGCA
TCAGCTTATAGCAATATAGGGCTGGCGACCGGCAAACGGGTGCGGAACACGTACACAACCTTCCTTTAAG
AGGGGGATAGCCCATAGAAATGTGGATTAATACCCCGTAAGATAGTGGGATGGCATCATACTACTATTAT
AGTTACGACGCTTGAAGATGGGTGTGCGTCTGATTAGGTAGTTGGCGGGGTAAAGGCCCACCAAGCCTTC
GATCAGTAGCTGATGTGAGAGCATGATCAGCCACACGGGCACTGAGACACGGGCCCGACTCCTACGGGAG
GCAGCAGTAAGGAATATTGGTCAATGGACGCAAGTCTGAACCAGCCATGCCGCGTGAAGGATGAAGGTCC
TCTGGATTGTAAACTTCTTTTATAGGGGGCGAAAAAAGGGAAATCTTTCTCACTTGACAGTACCCTATGA
ATAAGCACCGGCTAACTCCGTGCCAGCAGCCGCGGTAATACGGAGGGTGCAAGCGTTATCCGGATTCACT
GGGTTTAAAGGGTGCGTAGGCGGGCGTATAAGTCAGTGGTGAAATCCTGGAGCTTAACTCCAGAACTGCC
ATTGATACTATATGTCTTGAATATGGTGGAGGTAAGCGGAATATGTCATGTAGCGGTGAAATGCATAGAT
ATGACATAGAACACCTATTGCGAAGGCAGCTTACTACGCCTATATTGACGCTGAGGCACGAAAGCGTGGG
GATCAAACAGGATTAGATACCCTGGTAGTCCACGCCCTAAACGATGATTACTCGACGTGTGCGATAAACG
GTACGCGTCTGAGCGAAAGCATTAAGTAATCCACCTGGGAAGTACGACCGCAAGGTTGAAACTCAAAGGA
ATTGGCGGGGGTCCGCACAAGCGGTGGAGCATGTGGTTTAATTCGATGATACGCGAGGAACCTTACCTGG
GCTAGAATGCAGTCTGACCGTGGGTGAAAGCTCATTTTGTAGCAATACACAGATTGTAAGGTGCTGCATG
GCTGTCGTCAGCTCGTGCCGTGAGGTGTTGGGTTAAGTCCCGCAACGAGCGCAACCCCTATCATTAGTTG
CCAACAGGTAAAGCTGGGAACTCTAGTGAAACTGCCGTCGTAAGACGCGAGGAAGGAGGGGATGATGTCA
AGTCATCATGGCCTTTATGCCCAGGGCTACACACGTGCTACAATGGGGTGGACAAAGGGCTGCGACACAG
TGATGTGAAGCGAATCCCAAAAACCACTTCTCAGTTCAGATCGGAGTCTGCAACTCGACTCCGTGAAGCT
GGAATCGCTAGTAATCGTATATCAGCAATGATACGGTGAATACGTTCCCGGACCTTGCACACACCGCCCG
TCAAGCCATGGGAGCCGGGTGTACCTAAAGTCGGTAACCGCAAGGATCTGCCTAGGGTAAAATCGGTGAC
TGGGGCTAAGTCGTAACAAGGTAACCGTAAAGGGCG
>433525|emb|X71862.1|Actinomyces|Actinomyces neuii subsp. anitratus 16S
rRNA gene, strain 50/90

CGAACGCTGGCGGCGTGCTTAACACATGCAAGTCGAACGGGATCCATTAGCGCTTTTGTGTTTTTGGTGA
GAGTGGCGAACGGGTGAGTAACACGTGAGTAACCTGCCCTTTTCTTTGGGATAAGCCTGGGAAACTGGGT
CTAATACTGGATGTTCCGNCTTCCTCGCATGGGGTTGTTGGGAAAGGTTTTTTCTGGATTGGATGGGCT
CGCGGCTTATCAGCTTGTTGGTGGGGTGATGGCTTACCAAGGCTTTGACGGGTAGCCGGCCTGAGAGGGT
GGTCGGTCACACTGGGACTGAGATACGGCCCAGACTCCTACGGGAGGCAGCAGTGGGGGATATTGCACAA
TGGACGGAAGTCTGATGCACCGACGTCGTGTGGGGGATGAAGGCCTTCGGGTTGTAAACTCCTTTCGCCC
GCGGAAAAGGCAGGGTTTTGGCCTTGTTGATGGTAGTGGGTAAAGAAGCGCCGGCTCACTACGTGCCAGC
AGCCGCGGTAATACGTAGGGCGCGAGCGTTGTCCGGAATTATTGGGCGTAAAGGGCTCGTAGGCGGTTTG
TCGCGTCTAGCGTTTAAGGCTCGGGCTTAACCCGGGTTTGCGTTGGGTACGGGCAGGCTTGAGTGCGGTA
GGGGTAACTGGAATTCCTGGTGTAGCGGTGGAATGCGCAGATATCAGGAGGAACACCGGTGGCGAAGGCG
GGTTACTGGGCCGTTACTGACGCTGAGGAGCGAGAGCGTGGGGAGCGAACAGGATTAGATACCCTGGTAG
TCCATGCTGTAAACGTTGGGCACTAGGTGTGGGGGCTGGTTTCATGGTTCCGCGCCGTAGCTAACGCATT
AAGTGCCCCGCCTGGGGAGTACGGCCGCAAGGCTAAAACTCAAAGGAATTGACGGGGGCCCGCACAAGCG
GCGGAGCATGCGGATTAATTCGATGCAACGCGAAGAACCTTACCAAGGCTTGACATGCACCGCGAGACTG
CAGAGATGTGGTTGCCTTCGGGGTGGTGTGCAGGTGGTGCATGGTTGTCGTCAGCTCGTGTCGTGAGATG
TTGGTTAAGTCCCGCAACGAGCGCAACCCTTGTCTCATGTTGCCAGCACGTTATGGTGGGGACTCGTGAG
AGACTGCCGGGGTTAACTCGGAGGAAGGTGGGGATGACGTCAAATCATGATGCCCCATATGTCTTGGGCT
TCACGCATGCTACAGTGGTGTCTACAGAGGGTTGCTATTCCGTGAGGTTGAGCGAATCTCTTAAAGGGCA
TCTTGGTTCGGATCGCAGTCTGCAACTCGACTGCGTGAAGGTGGAGTCGCTAGTAATCGCAGATGAGCAT
TGCTGCGGTGAATACGTTCTCGGGCCTTGTACACACCGCCCGTCACGTCACGAAAGTTGGTAACACCCGA
AGCCTGTGGCCTAACCTTTTTGGGGGGAGTGGTCGAAGGTGGGGCTAGCGATTGGGACGAAGTCGTAACA
AGGTAGCCGTACCGGAAGG
>66878786|gb|AY959080.1|Actinomyces|Uncultured bacterium clone rRNA307
16S ribosomal RNA gene, partial sequence
GCCCTTAGAGTTTGATCCTGGCTCAGGACGAACGCTGACGGCGTGCTTAACACATGCAAGTCGAACGGGA
TCCATTAGCGCTTTTGTGTTTTTGGTGAGAGTGGCGAACGGGTGAGTAACACGTGAGTAACCTGCCCTTT
TCTTTGGGATAAGCCTGGGAAACTGGGTCTAATACTGGATGTTCCGGCTTCCTCGCATGGGGTTGTTGGG
AAAGGTTTTTTCTGGATTGGGATGGGCTCGCGGCTTATCAGCTTGTTGGTGGGGTGATGGCTTACCAAGG
CTTTGACGGGTAGCCGGCCTGAGAGGGTGGTCGGTCACACTGGGACTGAGATACGGCCCAGACTCCTACG
GGAGGCAGCAGTGGGGGATATTGCACAATGGACGGAAGTCTGATGCAGCGACGTCGTGTGGGGGATGAAG
GCCTTCGGGTTGTAAACTCCTTTCGCCCGCGGAAAAGGCAGGGTTTTGGCCTTGTTGATGGTAGTGGGTA
AAGAAGCGCCGGCTAACTACGTGCCAGCAGCCGCGGTAATACGTAGGGCGCGAGCGTTGTCCGGAATTAT
TGGGCGTAAAGGGCTCGTAGGCGGTTTGTCGCGTCTAGCGTTTAAGGCTCGGGCTTAACCCGGGTTTGCG
TTGGGTACGGGCAGGCTTGAGTGCGGTAGGGGTAACTGGAATTCCTGGTGTAGCGGTGGAATGCGCAGAT
ATCAGGAGGAACACCGGTGGCGAAGGCGGGTTACTGGGCCGTTACTGACGCTGAGGAGCGAGAGCGTGGG
GAGCGAACAGGATTAGATACCCTGGTAGTCCATGCTGTAAACGTTGGGCACTAGGTGTGGGGGCTGGTTT
CATGGTTTCCGCGCCGTAGCTAACGCATTAAGTGCCCCGCCTGGGGAGTACGGCCGCAAGGCTAAAACTC
AAAGGAATTGACGGGGGCCCGCACAAGCGGCGGAGCATGCGGATTAATTCGATGCAACGCGAAGAACCTT
ACCAAGGCTTGACATGCACCGCGAGACTGCAGAGATGTGGTTGCCTTCGGGGTGGTGTGCAGGTGGTGCA
TGGTTGTCGTCAGCTCGTGTCGTGAGATGTTGGGTTAAGTCCCGCAACGAGCGCAACCCTTGTCTCATGT
TGCCAGCACGTTATGGTGGGGACTCGTGAGAGACTGCCGGGGTTAACTCGGAGGAAGGTGGGGATGACGT
CAAATCATCATGCCCCTTATGTCTTGGGCTTCACGCATGCTACATTGGTGTCTACAGAGGGTTGCTATTC
CGTGAGGTTGAGCGAATCTCTTAAAGGGCATCTTGGTTCGGATCGCAGTCTGCAACTCGACTGCGTGAAG
GTGGAGTCGCTAGTAATCGCAGATCAGCATTGCTGCGGTGAATACGTTCTCGGGCCTTGTACACACCGCC
CGTCACGTCACGAAAGTTGGTAACACCCGAAGCCTGTGGCCTAACCTTTTTGGGGGGAGTGGTCGAAGGT
GGGGCTAGCGATTGGGACGAA
>27803995|emb|AJ428402.1|Varibaculum|Varibaculum cambriensis partial 16S
rRNA gene, strain CCUG 44998
TCAGGATGAACGCTGGCGGCGTGCTTAACACATGCAAGTCGAACGGGATCCAAGAAGTGCTTGCACTTTT
TGGTGAGAGTGGCGAACGGGTGAGTAACACGTGAGTAACCTGTCCTTTTCTTTGGGATAAGCATCCGAAA
GGGTGTTTAATACCTTATGTTCTGTCTGCCTCGCATGGGGTGGGTGGGAAAGATTTTTTCGGTAAAGGGT
GGGCTCGCGGTCTATCAGCTTGTTGGTGGGGTGATGGCCTACCAAGGCTTTGACGGGTAGCCGGCCTGAG
AGGGTGGTCGGTCACATTGGGACTGAGATACGGCCCAGACTCCTGCGGGAGGCAGCAGTGGGGGATATTG
CACAATGGGCGAAAGCCTGATGCAGCGACGCCGCGTGGGGGATGAAGGCCTTCGGGTTGTAAACTTCTTT

TGCTCTGAACAAGGCGCACCGTGGGGTGTGTTGAGTGTAGGGGTTGATTAGCGCCGGCTAACTACGTGCC
AGCAGCCGCGGTAATACGTAGGGCGCGAGCGTTGTCCGGAATTATTGGGCGTAAAGGGCTTGTAGGTGGC
TGGTTGCGTCTGTCGTGAAAGCTCATGGCTTAACTGTGGGTTTGCGGTGGGTACGGGCTGGCTTGAGTGC
AGTAGGGGAGGCTGGAATTCCTGGTGTAGCGGTGGAATGCGCAGATATCAGGAGGAATACCGGTGGCGAA
GGCGGGTCTCTGGGCTGTTACTGACACTGAGGAGCGAAAGCATGGGGAGCGAACAGGATTAGATACCCTG
GTAGTCCATGCTGTAAACGGTGGGAACTGGGTGTGGGGGGCTTTTTTGTCTTCTGCGTCGTAGCTAACGC
GTTAAGTTCCCCGCCTGGGGAGTACGGTCGCAAGGCTAAAACTCAAAGGAATTGACGGGGGCCCGCACAA
GCGGCGGAGCATGCGGATTAATTCGATGCAACGCGAAGAACCTTACCAAGGCTTGACATGCACTGGACCG
ATCCAGAGATGGGTTTTCCTTTTTGGCTGGTGTGCAGGTGGTGCATGGTTGTCGTCAGCTCGTGTCGTGA
GATGTTGGGTTAAGTCCCGCAACGAGCGCAACCCTTGTCCTATGTTGCCAGCAAGTTGTGTTGGGGACTC
GTGGGAGACTGCCGGGGTTAACTCGGAGGAAGGTGGGGATGACGTCAAATCATCATGCCCCTTATGTCTT
GGGCTTCACGCATGCTACATTGGCGTCTACAGAGGGTTGCGATACCGTAAGGTGGGGCGAATCTCTTAAA
GGGCGTCTTGGTTCGGATCGGGGTCTGCAACTCGGCCCCGTGAAGGTGGAGTCGCTAGTAATCGCAGATC
AGCAACGCTGCGGTGAATACGTTCTCGGGCCTTGTACACACCGCCCGTCACGTCACGAAAGTTGGTAACA
CTCGAAGCTTGTGGCCTAACTCTTTTTGAGGGGGAGCGGGTGAAGGTGGGGCTAGCGATTGGGACGAAGT
CGTAACAAGGTAGCCGTACCGGAAGGTGCGGCTGGATCAC
>66878754|gb|AY959048.1|Varibaculum|Uncultured bacterium clone rRNA275
16S ribosomal RNA gene, partial sequence
CCTTAGAGTTTGATCCTGGCTCAGGATGAACGCTGGCGGCGTGCTTAACACATGCAAGTCGAACGGGATC
CAAGGGGTGCTTGCACTTTTTGGTGAGAGTGGCGAACGGGTGAGTAACACGTGAGTAACCTGTCCTTTTC
TTTGGGATAAGCATTCGAAAGGGTGTCTAATACCTTATGTTCTGTCTGCCTCGCATGGGGTGGGTGGGAA
AGGCTTTGGCCGGATTTGGGTGGGCTCGCGGTCTATCAGCTTGTTGGTGGGGTGATGGCTTACCAAGGCT
TTGACGGGTAGCCGGCCTGAGAGGGTGGTCGGTCACATTGGGACTGAGATACGGCCCAGACTCCTGCGGG
AGGCAGCAGTGGGGGATATTGCACAATGGGCGAAAGCCTGATGCAGCGACGCCGCGTGGGGGATGAAGGC
CTTCGGGTTGTAAACTTCTTTTGCTCTGAACAAGGCGCACTAATGGGTGTGTTGAGTGTAGGGGTTGATT
AGCGCCGGCTAACTACGTGCCAGCAGCCGCGGTAATACGTAGGGCGCGAGCGTTGTCCGGAATTATTGGG
CGTAAAGGGCTTGTAGGTGGCTGGTTGCGTCTGTCGTGAAAGCTCATGGCTTAACTGTGGGTTTGCGGTG
GGTACGGGCTGGCTTGAGTGCAGTAGGGGAGGCTGGAATTCCTGGTGTAGCGGTGGAATGCGCAGATATC
AGGAGGAATACCGGTGGCGAAGGCGGGTCTCTGGGCTGTTACTGACACTGAGGAGCGAAAGCATGGGGAG
CGAACAGGATTAGATACCCTGGTAGTCCATGCTGTAAACGTTGGGAACTGGGTGTGGGGGGCTTTTTTGT
CTTCTGCGTCGTAGCTAACGCGTTAAGTTCCCCGCCTGGGGAGTACGGTCGCAAGGCTAAAACTCAAAGG
AATTGACGGGGGCCCGCACAAGCGGCGGAGCATGCGGATTAATTCGATGCAACGCGAAGAACCTTACCAA
GGCTTGACATGCACTGGACCGATCCAGAGATGGGTTTTCCTTTTTGGCTGGTGTGCAGGTGGTGCATGGT
TGTCGTCAGCTCGTGTCGTGAGATGTTGGGTTAAGTCCCGCAACGAGCGCAACCCTTGTCCTATGTTGCC
AGCAAGTTGTGTTGGGGACTCGTGGGAGACTGCCGGGGTTAACTCGGAGGAAGGTGGGGATGACGTCAAA
TCATCATGCCCCTTATGTCTTGGGCTTCACGCATGCTACATTGGCGTCTACAAAGGGTTGCGATACCGTA
AGGTGGTGCGAATCTCTTAAAGGGCGTCTTGGTTCGGATCGGGGTCTGCAACTCGGCCCCGTGAAGGTGG
AGTCGCTAGTAGTCGCAGATCAGCAACGCTGCGGTGAATACGTTCTCGGGCCTTGTACACACCGCCCGTC
ACGTCACGAAAGTTGGTAACACTCGAAGCTTGTGGCCTAACTCTTTTTGAGGGGGAGCGGGTGAAGGTGG
GGCTAGCGATTGGGACGAA
>52429823|gb|AY725812.1|Corynebacterium|Corynebacterium sp. C18 16S
ribosomal RNA gene, partial sequence
TTATGGAGAGTTTGATCCTGGCTCAGGACGAACGCTGGCGGCGTGCTTAACACATGCAAGTCGAACGGTA
AGGCCCCAGCTTGCTGGGGTACACGAGTGGCGAACGGGTGAGTAACACGTGGGTGACCTGCCCTGCACTT
CGGGATAAGCCTGGGAAACTGGGTCTAATACCGGATAGGACCTCAGCGTAGGGTTGGGGTGGAAAGTTTT
TTCGGTGCAGGATGGGCCCGCGGCCTATCAGCTTGTTGGTGGGGTAATGGCCTACCAAGGCGGCGACGGG
TAGCCGGCCTGAGAGGGTGGACGGCCACATTGGGACTGAGACACGGCCCAGACTCCTACGGGAGGCAGCA
GTGGGGAATATTGCACAATGGGCGGAAGCCTGATGCAGCGACGCCGCGTGGGGGATGACGGCCTTCGGGT
TGTAAACTCCTTTCACCATCGACGAAGGGTTTCTGACGGTAGATGGAGAAGAAGCACCGGCTAACTACGT
GCCAGCAGCCGCGGTAATACGTAGGGTGCGAGCGTTGTCCGGAATTACTGGGCGTAAAGAGCTCGTAGGT
GGTTTGTCGCGTCGTCTGTGAAATTCCGGGGCTTAACTCCGGGCGTGCAGGCGATACGGGCATAACTTGA
GTACTGTAGGGGAGACTGGAATTCCTGGTGTAGCGGTGAAATGCGCAGATATCAGGAGGAACACCGGTGG
CGAAGGCGGGTCTCTGGGCAGTAACTGACGCTGAGGAGCGAAAGCATGGGTAGCGAACAGGATTAGATAC
CCTGGTAGTCCATGCCGTAAACGGTGGGCGCTAGGTGTGGGTTTCCTTCCACGGGATCCGTGCCGTAGCT

AACGCATTAAGCGCCCCGCCTGGGGAGTACGGCCGCAAGGCTAAAACTCAAAGGAATTGACGGGGGCCCG
CACAAGCGGCGGAGCATGTGGATTAATTCGATGCAACGCGAAGAACCTTACCTGGGCTTGACATGTACGG
GATCGGGCCAGAGATGGTCTTTCCCTTGTGGCTCGTATACAGGTGGTGCATGGTTGTCGTCAGCTCGTGT
CGTGAGATGTTGGGTTAAGTCCCGCAACGAGCGCAACCCTTGTCTCATGTTGCCAGCACGTTGTGGTGGG
GACTCGTGAGAGACTGCCGGGGTCAACTCGGAGGAAGGTGGGGATGACGTCAAATCATCATGCCCCTTAT
GTCCAGGGCTTCACACATGCTACAATGGCCGGTACAGTGGGTTGCGATGCCGTGAGGTGGAGCTAATCCC
TTAAAGCCGGTCTCAGTTCGGATCGGGGTCTGCAACTCGACCCCGTGAAGTCGGAGTCGCTAGTAATCGC
AGATCAGCAACGCTGCGGTGAATACGTTCCCGGGCCTTGTACACACCGCCCGTCACGTCATGAAAGTCGG
TAACACCCGAAGCCAGTGGCCCAACCTTTTGTGGGGGGAGCTGTCGAAGGTGGGATCGGCGATTGGGACG
AAGTCGAACAAGGAGCCGA
>56480628|gb|AY831726.1|Corynebacterium|Corynebacterium amycolatum 16S ribosomal RNA gene, partial sequence
GCTGGCGGCGTGCTTAACACATGCAAGTCGAACGGTAAGGCTCCAGCTTGCTGGGGTACACGAGTGGCGA
ACGGGTGAGTAACACGTGGGTGACCTGCCCTGCACTTCGGGATAAGCCTGGGAAACTGGGTCTAATACCG
GATAGGACCGCACCGTGAGGGTGTGGTGGAAAGTTTTTCGGTGTGGGATGGGCCCGCGGCCTATCAGCT
TGTTGGTGGGGTAATGGCCTACCAAGGCGGCGACGGGTAGCCGGCCTGAGAGGGTGGACGGCCACATTGG
GACTGAGACACGGCCCAGACTCCTACGGGAGGCAGCAGTGGGGAATATTGCACAATGGGCGGAAGCCTGA
TGCAGCGACGCCGCGTGGGGGATGACGGCCTTCGGGTTGTAAACTCCTTTCACCATCGACGAAGGGTTTC
TGACGGTAGATGGAGAAGAAGCACCGGCTAACTACGTGCCAGCAGCCGCGGTAATACGTAGGGTGCGAGC
GTTGTCCGGAATTACTGGGCGTAAAGAGCTCGTAGGTGGTTTGTCGCGTCGTCTGTGAAATTCCGGGGCT
TAACTCCGGGCGTGCAGGCGATACGGGCATAACTTGAGTACTGTAGGGGAGACTGGAATTCCTGGTGTAG
CGGTGAAATGCGCAGATATCAGGAGGAACACCGGTGGCGAAGGCGGGTCTCTGGGCAGTAACTGACGCTG
AGGAGCGAAAGCATGGGGAGCGAACAGGATTAGATACCCTGGTAGTCCATGCCGTAAACGGTGGGCGCTA
GGTGTGGGTTTCCTTCCACGGGATCCGTGCCGTAGCTAACGCATTAAGCGCCCCGCCTGGGGAGTACGGC
CGCAAGGCTAAAACTCAAAGGAATTGACGGGGGCCCGCACAAGCGGCGGAGCATGTGGATTAATTCGATG
CAACGCGAAGAACCTTACCTGGGCTTGACATATACAGGATCGCGCCAGAGATGGTGTTTCCCTTGTGGCT
TGTATACAGGTGGTGCATGGTTGTCGTCAGCTCGTGTCGTGAGATGTTGGGTTAAGTCCCGCAACGAGCG
CAACCCTTGTCTTATGTTGCCAGCACGTTGTGGTGGGGACTCGTAAGAAACTGCCGGGGTTAACTCGGAG
GAAGGTGGGGATGACGTCAAATCATCATGCCCCTTATGTCCAGGGCTTCACACATGCTACAATGGTCGGT
ACAGTGGGTTGCCAGTCCGTGAGGGCGAGCTAATCCCGCAAAGCCGGTCTCAGTTCGGATCGGGGTCTGC
AACTCGACCCCGTGAAGTCGGAGTCGCTAGTAATCGCAGATCAGCAACGCTGCGGTGAATACGTTCCCGG
GCCTTGTACACACCGCCCGTCACGTCATGAAAGTCGGTAACACCCGAAGCCAGGGCCTAACACATTTTGT
GGGGGAGCTGTGAAGGTGGG
>66878616|gb|AY958910.1|Corynebacterium|Uncultured bacterium clone rRNA137 16S ribosomal RNA gene, partial sequence
CGCCCTTAGAGTTTGATCCTGGCTCAGGACGAACGCTGGCGGCGTGCTTAACACATGCAAGTCGAACGGT
AAGGCTCCAGCTTGCTGGGGTACACGAGTGGCGAACGGGTGAGTAACACGTGGGTGACCTGCCCTGCACT
TCGGGATAAGCCTGGGAAACTGGGTCTAATACCGGATAGGACCGCACCGTGAGGGTGTGGTGGAAAGTTT
TTTCGGTGTGGGATGGGCCCGCGGCCTATCAGCTTGTTGGTGGGGTAATGGCCTACCAAGGCGGCGACGG
GTAGCCGGCCTGAGAGGGTGGACGGCCACATTGGGACTGAGACACGGCCCAGACTCCTACGGGAGGCAGC
AGTGGGGAATATTGCACAATGGGCGGAAGCCTGATGCAGCGACGCCGCGTGGGGGATGACGGCCTTCGGG
TTGTAAACTCCTTTCACCATCGACGAAGGGTTTCTGACGGTAGATGGAGAAGAAGCACCGGCTAACTACG
TGCCAGCAGCCGCGGTAATACGTAGGGTGCGAGCGTTGTCCGGAATTACTGGGCGTAAAGAGCTCGTAGG
TGGTTTGTCGCGTCGTCTGTGAAATTCCGGGGCTTAACTCCGGGCGTGCAGGCGATACGGGCATAACTTG
AGTACTGTAGGGGAGACTGGAATTCCTGGTGTAGCGGTGAAATGCGCAGATATCAGGAGGAACACCGGTG
GCGAAGGCGGGTCTCTGGGCAGTAACTGACGCTGAGGAGCGAAAGCATGGGGAGCGAACAGGATTAGATA
CCCTGGTAGTCCATGCCGTAAACGGTGGGCGCTAGGTGTGGGTTTCCTTCCACGGGATCCGTGCCGTAGC
TAACGCATTAAGCGCCCCGTCTGGGGAGTACGGCCGCAAGGCTAAAACTCAAAGGAATTGACGGGGGCCC
GCACAAGCGGCGGAGCATGTGGATTAATTCGATGCAACGCGAAGAACCTTACCTGGGCTTGACATATACA
GGATCGCGCCAGAGATGGTGTTTCCCTTGTGGCTTGTATACAGGTGGTGCATGGTTGTCGTCAGCTCGTG
TCGTGAGATGTTGGGTTAAGTCCCGCAACGAGCGCAACCCTTGTCTTATGTTGCCAGCACGTTGTGGTGG
GGACTCGTAAGAAACTGCCGGGGTTAACTCGGAGGAAGGTGGGGATGACGTCAAATCATCATGCCCCTTA
TGTCCAGGGCTTCACACATGCTACAATGGTCGGTACAGTGGGTTGCCGGTCCGTGAGGGCGAGCTAATCC
CGCAAAGCCGGTCTCAGTTCGGATCGGGGTCTGCAACTCGACCCCGTGAAGTCGGAGTCGCTAGTAATCG

CAGATCAGCAACGCTGCGGTGAATACGTTCCCGGGCCTTGTACACACCGCCCGTCACGTCATGAAAGTCG
GTAACACCCGAAGCCAGTGGCCTAACACATTTTGTGGGGGAGCTGTCGAAGGTGGGATTGGCGATTGGGA
CGAA
>66878644|gb|AY958938.1|Corynebacterium|Uncultured bacterium clone
rRNA165 16S ribosomal RNA gene, partial sequence
TCGCCCTTAGAGTTTGATCCTGGCTCAGGATGAACGCTGGCGGCGTGCTTAACACATGCAAGTCGAACGG
AAAGGCCCTTGCTTGCAAGGGTGCTCGAGTGGCGAACGGGTGAGTAACACGTAGGTGATCTGCCTTTCAC
TTCGGGATAAGCTTGGGAAACTGGGTCTAATACCGGATATTCACGGCACCGTGGTTGGTGTGGTGGAAAG
CTTTTGCGGTGGGAGATGAGCTTGCGGCCTATCAGCTTGTTGGTGGGGTAATGGCCTACCAAGGCGGCGA
CGGGTAGCCGGCCTGAGAGGGTGTGCGGCCACATTGGGACTGAGATACGGCCCAGACTCCTACGGGAGGC
AGCAGTGGGGAATTTTGCACAATGGGCGGAAGCCTGATGCAGCGACGCCGTGTGGGGGATGAAGGCCTTC
GGGTTGTAAACTCCTTTCGTCAGGGACGAAGTTTGTTTGACGGTACCTGGATAAGAAGCACCGGCTAACT
ACGTGCCAGCAGCCGCGGTAATACGTAGGGTGCGAGCGTTGTCCGGATTTACTGGGCGTAAAGGGCTCGT
AGGTGGTGTGTTGCGTCGTCTGTGTAATCCAGGGGCTTAACTTTTGGTTGGCAGGCGATACGGGCATTGC
TTGAGTGCTGTAGGGGAGACTGGAATTCCTGGTGTAGCGGTGAAATGCGCAGATATCAGGAGGAACACCG
ATGGCGAAGGCAGGTCTCTGGGCAGTCACTGACGCTGAGGAGCGAGAGCATGGGTAGCGAACAGGATTAG
ATACCCTGGTAGTCTATGCTGTAAACGGTGGGCGCTAGGTGTGAGCCTCTTCCACGGGGTTTGTGCCGTA
GCTAACGCTTTAAGCGCCCCGCCTGGGGAGTACGGCCGCAAGGCTAAAACTCAAAGGAATTGACGGGGGC
CCGCACAAGCGGCGGAGCATGTGGATTAATTCGATGCAACGCGAAGAACCTTACCTGGGCTTGACATAGG
CAGGATGGGCGCAGAGATGTGTCGTCCCTTTGTGGTCTGTTTACAGGTGGTGCATGGTTGTCGTCAGCTC
GTGTCGTGAGATGTTGGGTTAAGTCCCGCAACGAGCGCAACCCTTGTCTTATGTTGCCAGCACGTTGTGG
TGGGGACTCGTGAGAGACTGCCGGGGTTAACTCGGAGGAAGGTGGGGATGACGTCAAATCATCATGCCCC
TTATGTCCAGGGCTTCACACATGCTACAATGGTTGGTACAGTGCGTGAGCAACACCGTGAGGTGGAGCTA
ATCGCTAAAGCCAGTCTCAGTTCGGATTGGGGTCTGCAACTCGACCTCATGAAGTCGGAGTCGCTAGTAA
TCGCAGATCAGCAGTGCTGCGGTGAATACGTTCCCGGGCCTTGTACACACCGCCCGTCACGTCATGAAAG
TTGGTAACACCCGAAGCCCATGGCCTAACCGGTTTTTGCCGGGGGGAGTGGTCGAAGGTGGGATTGGCGA
TTGGGACGAAGTCGTAACAAGGTAACCGTAAAGGGC
>66878649|gb|AY958943.1|Corynebacterium|Uncultured bacterium clone
rRNA170 16S ribosomal RNA gene, partial sequence
ATGAACGCTGGCGGCGTGCTTAACACATGCAAGTCGAACGGAAAGGCTCCTGCTTGCAGGGGTACTCGAG
TGGCGAACGGGTGAGTAACACGTGGGTGATCTGCCCTGTACTTCGGGATAAGCTTGGGAAACTGGGTCTA
ATACCGGATAGGACAACTTTTTGGATATTGTTGTGGAAAGCTTTTGCGGTATGGGATGAGCTCGCGGCCT
ATCAGCTTGTTGGTGGGGTAATGGCCTACCAAGGCGTCGACGGGTAGCCGGCCTGAGAGGGTGTACGGCC
ACATTGGGACTGAGATACGGCCCAGACTCCTACGGGAGGCAGCAGTGGGGAATATTGCACAATGGGCGCA
AGCCTGATGCAGCGACGCCGCGTGGGGGATGAAGGCCTTCGGGTTGTAAACTCCTTTCGCTAGGGACGAA
GCTTTTTTGTGACGGTACCTGGAGAAGAAGCACCGGCTAACTACGTGCCAGCAGCCGCGGTAATACGTAG
GGTGCGAGCGTTGTCCGGAATTACTGGGCGTAAAGAGCTCGTAGGTGGTTTGTCGCGTCGTTTGTGTAAG
TCCACAGCTTAACTGTGGGACTGCAGGCGATACGGGCATAACTTGAGTGCTGTAGGGGAGACTGGAATTC
CTGGTGTAGCGGTGGAATGCGCAGATATCAGGAGGAACACCGATGGCGAAGGCAGGTCTCTGGGCAGTAA
CTGACGCTGAGGAGCGAAAGCATGGGTAGCGAACAGGATTAGATACCCTGGTAGTCCATGCCGTAAACGG
TGGGCGCTAGGTGTGAGTCCCTTCCACGGGGTTCGTGCCGTAGCTAACGCATTAAGCGCCCCGCCTGGGG
AGTACGGCCGCAAGGCTAAAACTCAAAGGAATTGACGGGGGCCCGCACAAGCGGCGGAGCATGTGGATTA
ATTCGATGCAACGCGAAGAACCTTACCTGGGCTTGACATGTAGCGGATCGCCGCAGAGATGTGGTTTCCC
TTGTGGTCGCTATACAGGTGGTGCATGGTTGTCGTCAGCTCGTGTCGTGAGATGTTGGGTTAAGTCCCGC
AACGAGCGCAACCCTTGTCTTATGTTGCCAGCACGTGATGGTGGGGACTCATGAGAGACTGCCGGGGTTA
ACTCGGAGGAAGGTGGGGATGACGTCAAATCATCATGCCCCTTATGTCCAGGGCTTCACACATGCTACAA
TGGTCGGTACAGCGCGTTTGCTACCTTGTGAGGGGATGCTAATCGCACAAAGCCGGCCTTAGTTCGGATT
GGGGTCTGCAACTCGACCCCATGAAGTCGGAGTCGCTAGTAATCGCAGATCAGCAACGCTGCGGTGAATA
CGTTCCCGGGCCTTGTACACACCGCCCGTCGCGTCATGAAAGTTGGTAACACCCGAAGCCAGTGGCCTGT
CATGGGAGCTGTCGAAGGTGGGATCGGCGATTGGGACGAAGTCGTAACAAGGTAGCCGTAAAGGGC
>66878672|gb|AY958966.1|Corynebacterium|Uncultured bacterium clone
rRNA193 16S ribosomal RNA gene, partial sequence
TCGCCCTTAGAGTTTGATCCTGGCTCAGGACGAACGCTGGCGGCGTGCTTAACACATGCAAGTCGAACGG
AAAGGCCCTGCTTGCAGGGTGCTCGAGTGGCGAACGGGTGAGTAACACGTGGGTGATCTGCCCCTTACTT

TGGGATAAGCCTGGGAAACTGGGTCTAATACTGGATAGGACCATGCTGTAGGTGGTGTGGTGGAAAGATT
AGTTTCGGTAAGGGATGAGCTCGCGGCCTATCAGCTTGTTGGTGGGGTAATGGCCTACCAAGGCGTCGAC
GGGTAGCCGGCCTGAGAGGGTGGACGGCCACATTGGGACTGAGATACGGCCCAGACTCCTACGGGAGGCA
GCAGTGGGGAATATTGCACAATGGGCGCAAGCCTGATGCAGCGACGCCGCGTGGGGGATGACGGCCTTCG
GGTTGTAAACTCCTTTCAGCCATGACGAAGCCCGTGTGGTGACGGTAGTGGTAGAAGAAGCACCGGCTAA
CTACGTGCCAGCAGCCGCGGTAATACGTAGGGTGCGAGCGTTGTCCGGAATTACTGGGCGTAAAGAGCTC
GTAGGTGGTCTGTCGCGTCATTTGTGAAAGCCCGGGGCTTAACTCCGGGTTGGCAGGTGATACGGGCATG
ACTGGAGTACTGTAGGGGAGACTGGAATTCCTGGTGTAGCGGTGAAATGCGCAGATATCAGGAGGAACAC
CGGTGGCGAAGGCGGGTCTCTGGGCAGTAACTGACGCTGAGGAGCGAAAGCATGGGTAGCGAACAGGATT
AGATACCCTGGTAGTCCATGCCGTAAACGGTGGGCGCTAGGTGTGGGTTTCCTTCCACGGGATCCGTGCC
GTAGCTAACGCATTAAGCGCCCCGCCTGGGGAGTACGGCCGCAAGGCTAAAACTCAAAGGAATTGACGGG
GGCCCGCACAGGCGGCGGAGCATGTGGATTAATTCGATGCAACGCGAAGAACCTTACCCGGGCTTGACAT
GCACTGGATGCGGCCAGAGATGGTTGTTCCCTTTGTGGCTGGTGTGCAGGTGGTGCATGGTTGTCGTCAG
CTCGTGTCGTGAGATGTTGGGTTAAGTCCCGCAACGAGCGCAACCCTTGTCTCGTGTTGCCAGCATTTGG
TTGGGGACTCGCGGGAGACTGCCGGGGTTAACTCGGAGGAAGGTGGGGATGACGTCAAATCATCATGCCC
CTTATGTCCAGGGCTTCACACATGCTACAATGGCTGGTACAGAGAGTTGCGATACCGTGAGGTGGGGCTA
ATCTCGTAAAGCCAGTCTCAGTTCGGATTGGAGTCTGCAACTCGACTCCATGAAGTCGGAGTCGCTAGTA
ATCGCAGATCAGCAATGCTGCGGTGAATACGTTCCCGGGCCTTGTACACACCGCCCGTCACGTCATGAAA
GTTGGTAACACCCGAAGCCGGTGGCCTAAACTCGTTAGGGAGCCGTCGAAGGTGGGATTGGCGATTGGGA
CGAA
>66878673|gb|AY958967.1|Corynebacterium|Uncultured bacterium clone
rRNA194 16S ribosomal RNA gene, partial sequence
CGCCCTTAGAGTTTGATCCTGGCTCAGGACGAACGCTGGCGGCGTGCTTAACACATGCAAGTCGAACGGA
AAGGCCCTGCTTGCAGGGTACTCGAGTGGCGAACGGGTGAGTAACACGTGGGTGATCTGCCCTGCACTTC
GGGATAAGCCTGGGAAACTGGGTCTAATACCGGATAGGAGCCATTTTTAGTGTGATGGTTGGAAAGTTTT
TTCGGTGTAGGATGAGCTCGCGGCCTATCAGCTTGTTGGTGGGGTAATGGCCTACCAAGGCGGCGACGGG
TAGCCGGCCTGAGAGGGTGGACGGCCACATTGGGACTGAGATACGGCCCAGACTCCTACGGGAGGCAGCA
GTGGGGAATATTGCACAATGGGCGCAAGCCTGATGCAGCGACGCCGCGTGGGGGATGACGGCCTTCGGGT
TGTAAACTCCTTTCGCTAGGGACGAAGCTTTTTGTGACGGTACCTAGATAAGAAGCACCGGCTAACTACG
TGCCAGCAGCCGCGGTAATACGTAGGGTGCGAGCGTTGTCCGGAATTACTGGGCGTAAAGGGCTCGTAGG
TGGTTTGTCGCGTCGTCTGTGAAATTCCGGGGCTTAACTCCGGGCGTGCAGGCGATACGGGCATAACTTG
AGTACTGTAGGGGTAACTGGAATTCCTGGTGTAGCGGTGAAATGCGCAGATATCAGGAGGAACACCGATG
GCGAAGGCAGGTTACTGGGCAGTTACTGACGCTGAGGAGCGAAAGCATGGGTAGCGAACAGGATTAGATA
CCCTGGTAGTCCATGCCGTAAACGGTGGGCGCTAGGTGTGAGGGTCTTTTCACGACTTTCGTGCCGTAGC
TAACGCATTAAGCGCCCCGCCTGGGGAGTACGGCCGCAAGGCTAAAACTCAAAGGAATTGACGGGGGCCC
GCACAAGCGGCGGAGCATGTGGATTAATTCGATGCAACGCGAAGAACCTTACCTGGGCTTGACATACACC
GGATCGGGCTAGAGATAGTCTTTCCCTTTGTGGCTGGTGTACAGGTGGTGCATGGTTGTCGTCAGCTCGT
GTCGTGAGATGTTGGGTTAAGTCCCGCAACGAGCGCAACCCTTGTCTTATGTTGCCAGCATTTGGTTGGG
GACTCATGAGAGACTGCCGGGGTCAACTCGGAGGAAGGTGGGGATGACGTCAAATCATCATGCCCCTTAT
GTCCAGGGCTTCACACATGCTACAATGGTCGGTACAACGCGCAGCGACACTGTGAGGTGGAGCGAATCGC
TGAAAGCCGGCCTTAGTTCGGATTGGGGTCTGCAACTCGACCCCATGAAGTCGGAGTCGCTAGTAATCGC
AGATCAGCAATGCTGCGGTGAATACGTTCCCGGGCCTTGTACACACCGCCCGTCACGTCATGAAAGTTGG
TAACACCCGAAGCCGGTGGCCCAAACTTGTTAGGGAGCCGTCGAAGGTGGGATCGGCGATTGGGACGAA
>66878690|gb|AY958984.1|Corynebacterium|Uncultured bacterium clone
rRNA211 16S ribosomal RNA gene, partial sequence
CCCTTAGAGTTTGATCCTGGCTCAGGATGAACGCTGGCGGCGTGCTTAACACATGCAAGTCGAACGGAAA
GGCCAGTGCTTGCACTGGTACTCGAGTGGCGAACGGGTGAGTAACACGTGGGTGATCTGCCCTGTACTTC
GGGATAAGCTTGGGAAACTGGGTCTAATACCGGATAGGACAACTTTTTGGATATTGTTGTGGAAAGCTTT
TGCGGTATGGGATGAGCTCGCGGCCTATCAGCTTGTTGGTGGGGTAATGGCCTACCAAGGCGTCGACGGG
TAGCCGGCCTGAGAGGGTGTACGGCCACATTGGGACTGAGATACGGCCCAGACTCCTACGGGAGGCAGCA
GTGGGGAATATTGCACAATGGGCGCAAGCCTGATGCAGCGACGCCGCGTGGGGGATGAAGGCCTTCGGGT
TGTAAACTCCTTTCGCTAGGGACGAAGCGCAAGTGACGGTACCTGGAGAAGAAGCACCGGCTAACTACGT
GCCAGCAGCCGCGGTAATACGTAGGGTGCGAGCGTTGTCCGGAATTACTGGGCGTAAAGAGCTCGTAGGT
GGTTTGTCGCGTCGTTTGTGTAAGTCCACAGCTTAACTGTGGGACTGCAGGCGATACGGGCATAACTTGA

GTGCTGTAGGGGAGACTGGAATTCCTGGTGTAGCGGTGAAATGCGCAGATATCAGGAGGAACACCGATGG
CGAAGGCAGGTCTCTGGGCAGTAGCTGACGCTGAGGAGCGAAAGCATGGGTAGCGAACAGGATTAGATAC
CCTGGTAGTCCATGCCGTAAACGGTGGGCGCTAGGTGTGAGTCCCTTCCACGGGATTTGTGCCGTAGCTA
ACGCATTAAGCGCCCCGCCTGGGGAGTACGGCCGCAAGGCTAAAACTCAAAGGAATTGACGGGGGCCCGC
ACGAGCGGCGGAGCATGTGGATTAATTCGATGCAACGCGAAGAACCTTACCTGGGCTTGACATGTAGCGG
ATCGCCGCAGAGATGTGGTTTCCCTTGTGGTCGCTATACAGGTGGTGCATGGTTGTCGTCAGCTCGTGTC
GTGAGATGTTGGGTTAAGTCCCGCAACGAGCGCAACCCTTGTCTTATGTTGCCAGCACGTAATGGTGGGG
ACTCATGAGAGACTGCCGGGGTTAACTCGGTGGAAGGTGGGGATGACGTCAAATCATCATGCCCCTTATG
TCCAGGGCTTCACACATGCTACAATGGTCGGTACAGCGCGTTTGCTACCTTGTGAGGGGATGCTAATCGC
ACAAAGCCGGCCTTAGTTCGGATTGGGGTCTGCAACTCGACCCCATGAAGTCGGAGTCGCTAGTAATCGC
AGATCAGCAACGCTGCGGTGAATACGTTCCCGGGCCTTGTACACACCGCCCGTCACGTCATGAAAGTTGG
TAACACCCGAAGCCAGTGGCCTGTCATGGGAGCTGTCGAAGGTGGGATCGGCGATTGGGACGAAGTCGTA
ACAAGGTAACCGTAAAGGGCG
>158905533|gb|EU086796.1|Brevibacterium|Brevibacterium paucivorans strain 28 16S ribosomal RNA gene, partial sequence
GGAGAAGCTGGCGGCGTGCTTAACACATGCAAGTCGAACGATGAAGCTCTAGCTTGCTGGGGTGGATTAG
TGGCGAACGGGTGAGTAACACGTGAGTAACCTGCCCTTTACTTCGGGATAAGCTTGGGAAACTGGGTCTA
ATACCGGATATTCTGCATGGTCGGATGGTTGTGTAGGAAAGATTTTTTGGTAAGGGATGGGCTCGCGGCC
TATCAGTTTGTTGGTGGGGTGATGGCCTACCAAGACGACGACGGGTAGCCGGCCTGAGAGGGCGACCGGC
CACACTGGGACTGAGACACGGCCCAGACTCCTACGGGAGGCAGCAGTGGGGAATATTGCACAATGGGGGA
AACCCTGATGCAGCGACGCCGCGTGGGGGATGACGGCCTTCGGGTTGTAAACCTCTTTCAGTAGGGAAGA
AGCGAAAGTGACGGTACCTGCAGAAGAAGTACCGGCTAACTACGTGCCAGCAGCCGCGGTAATACGTAGG
GTACTAGCGTTGTCCGGAATTATTGGGCGTAAAGAGCTCGTAGGTGGTTTGTCGCGTCTGCTGTGGAAAC
GTGCCGCTTAACGGTGCGCGTGCAGTGGGTACGGGCGGACTAGAGTGCAGTAGGGGAGTCTGGAATTCCT
GGTGTAGCGGTGAAATGCGCAGATATCAGGAGGAACACCGGTGGCGAAGGCGGGACTCTGGGCTGTTACT
GACGCTGAGGAGCGAAAGCATGGGGAGCGAACAGGATTAGATACCCTGGTAGTCCATGCCGTAAACGTTG
GGCACTAGGTGTGGGGTCCATTCCACGGATTCTGCGCCGTAGCTAACGCATTAAGTGCCCCGCCTGGGGA
GTACGGCCGCAAGGCTAAAACTCAAAGGAATTGACGGGGGCCCGCACAAGCGGCGGAGCATGCGGATTAA
TTCGATGCAACGCGAAGAACCTTACCAAGGCTTGACATGCACTAGACCATCGCAGAGATGTGGTTTTCTT
TTTGACTGGTGTACAGGTGGTGCATGGTTGTCGTCAGCTCGTGTCGTGAGATGTTGGGTTAAGTCCCGCA
ACGAGCGCAACCCTCGTTCTATGTTGCCAGCACGTGATGGTGGGAACTCATGGGAGACTGCCGGGGTCAA
CTCGGAGGAAGGTGGGGATGACGTCAAATCATCATGCCCTTTATGTCTTGGGCTTCACGCATGCTACAAT
GGCCGGTACAGAGGGTGGCGATACCGTGAGGTGGAGCGAATCTCTTAAAGCCGGTCTCAGTTCGGATCGT
AGTCTGCAACTCGACTACGTGAAGTCGGAGTCGCTAGTAATCGCAGATCAGCAATGCTGCGGTGAATACG
TTCCCGGGCCTTGTACACACCGCCCGTCAAGTCACGAAAGTCGGTAACACCCGAAGCCGGTGGCCTAACC
CTGTGTGGGGGGAGCCGTCGAAGGTGGGACTGGTGATTGGGACT
>66878914|gb|AY959208.1|Brevibacterium|Uncultured bacterium clone rRNA435 16S ribosomal RNA gene, partial sequence
ACGAACGCTGGCGGCGTGCTTAACACATGCAAGTCGAGCGCTGAAGCTTGGTGTTTTCACTGGGTGGATG
AGTGGCGAACGGGTGAGTAACACGTGAGTAACCTGCCCTTTACTTCGGGATAAGCTTGGGAAACTGGGTC
TAATACCGGATATTCTGCGTGATCGTATGGTTGTGTAGGAAAGATTTTTTGGTAAGGGATGGGCTCGCGG
CCTATCAGTTTGTTGGTGGGGTGATGGCCTACCAAGACGACGACGGGTAGCCGGCCTGAGAGGGCGACCG
GCCACACTGGGACTGAGACACGGCCCAGACTCCTACGGGAGGCAGCAGTGGGGAATATTGCACAATGGGG
GAAACCCTGATACAGCGACGCCGCGTGGGGGATGACGGCCTTCGGGTTGTAAACCTCTTTCAGTAGGGAA
GAAGCGTAAGTGACGGTACCTGCAGAAGAAGTACCGGCTAACTACGTGCCAGCAGCCGCGGTAATACGTA
GGGTACTAGCGTTGTCCGGAATTATTGGGCGTAAAGAGCTCGTAGGTGGTTTGTCGCGTCTGCTGTGGAA
ACGTGCCGCTTAACGGTGCGCGTGCAGTGGGTACGGGCGGACTAGAGTGCAGTAGGGGAGTCTGGAATTC
CTGGTGTAGCGGTGAAATGCGCAGATATCAGGAGGAACACCGGTGGCGAAGGCGGGACTCTGGGCTGTTA
CTGACGCTGAGGAGCGAAAGCATGGGGAGCGAACAGGATTAGATACCCTGGTAGTCCATGCCGTAAACGT
TGGGCACTAGGTGTGGGGTCCATTCCACGGATTCTGCGCCGTAGCTAACGCATTAAGTGCCCCGCCTGGG
GAGTACGGCCGCAAGGCTAAAACTCAAAGGAATTGACGGGGGCCCGCACAAGCGGCGGAGCATGCGGATT
AATTCGATGCAACGCGAAGAACCTTACCAAGGCTTGACATGCACTGGATCGCTGCAGAGATGTGGTTTTC
TTTTTGACTGGTGTACAGGTGGTGCATGGTTGTCGTCAGCTCGTGTCGTGAGATGTTGGGTTAAGTCCCG
CAACGAGCGCAACCCTCGTTCTATGTTGCCAGCACGTGATGGTGGGAACTCATGGGAGACTGCCGGGGTC

AACTCGGAGGAAGGTGGGGATGACGTCAAATCATCATGCCCTTTATGTCTTGGGCTTCACGCATGCTACA
ATGGCCGGTACAGAGGGTGGCGATACCGTGAGGTGGAGCGAATCTCTTAAAGCCGGTCTCAGTTCGGATC
GTAGTCTGCAACTCGACTACGTGAAGTCGGAGTCGCTAGTAATCGCAGATCAGCAATGCTGCGGTGAATA
CGTTCCCGGGCCTTGTACACACCGCCCGTCAAGTCACGAAAGTCGGTAACACCCGAAGCCGGTGGCCTAA
CCTGTGTGGGGGGAGCCGTCGAAGGTGGGACTGGTGATTGGGACTAA
>1359505|emb|X91032.1|Brachybacterium|Brachybacterium faecium 16S
ribosomal RNA gene
AGAGTTTGATNNTGGCTCAGGACGAACGCTGGCGGCGTGCTTAACACATGCAAGTCGAACGATGACGGTG
GTGCTTGCACTGCCTGATTAGTGGCGAACGGGTGAGTAACACGTGAGTAACCTGCCCCTCACTTCGGGAT
AACCTCGGGAAATCGAGGCTAATACCGGATATGAGCTTCTGCCGCATGGTGGGGGTTGTAAAGATTTATC
GGTGAGGGATGGACTCGCGGCCTATCAGTTTGTTGGTGAGGTAATGGCTCACCAAGGCGATGACGGGTAG
CCGGCCTGAGAGGGCGACCGGCCACACTGGGACTGAGACACGGCCCAGACTCCTACGGGAGGCAGCAGTG
GGGAATATTGCACAATGGGCGAAAGCCTGATGCAGCGACGCCGCGTGGGGGATGACGGCCTTCGGGTTGT
AAACCCCTTTCAGTAGGGAAGAAGCGAGAGTGACGGTACCTGCAGAAGAAGCGCCGGCTAACTACGTGCC
AGCAGCCGCGGTAATACGTAGGGCGCAAGCGTTGTCCGGAATTATTGGGCGTAAAGAGCTTGTAGGTGGC
TTGTCGCGTCTGCCGTGAAAACCCGAGGCTCAACCTCGGGCGTGCGGTGGGTACGGGCAGGCTAGAGTGT
GGTAGGGGAGACTGGAACTCCTGGTGTAGCGGTGAAATGCGCAGATATCAGGAAGAACACCGATGGCGAA
GGCAGGTCTCTGGGCCATTACTGACACTGAGAAGCGAAAGCATGGGTAGCGAACAGGATTAGATACCCTG
GTAGTCCATGCCGTAAACGTTGGGCACTAGGTGTGGGGGACATTCCACGTTCTCCGCGCCGTAGCTAACG
CATTAAGTGCCCCGCCTGGGGAGTACGGCCGCAAGGCTAAAACTCAAAGGAATTGACGGGGGCCCGCACA
AGCGGCGGAGCATGCTGATTAATTCGATGCAACGCGAAGAACCTTACCAAGGCTTGACATGCACCGGACG
GTCGCAGAGATGTGGCTTTCTTCGGACTGGTGCACAGGTGGTGCATGGTTGTCGTCAGCTCGTGTCGTGA
GATGTTGGGTTAAGTCCCGCAACGAGCGCAACCCTTGTTCTATGTTGCCAGCACGTGATGGTGGGGACTC
ATAGGAGACTGCCGGGGTCAACTCGGAGGAAGGTGGGGACGACGTCAAATCATCATGCCCCTTATGTCTT
GGGCTTCAAGCATGCTACAATGGCCGGTACAATGGGTTGCGAAACTGTGAGGTGGAGCGAATCCCAAAAA
GCCGGCCTCAGTTCGGATTGGGGTCTGCAACTCGACCCCATGAAGTCGGAGTCGCTAGTAATCGCAGATC
AGCAACGCTGCGGTGAATACGTTCCCGGGCCTTGTACACACCGCCCGTCAAGTCACGAAAGTCGGTAACA
CCCGAAGCCAGTGGCCCATCCTCGTGAGGGAGCTGTCGAAGGTGGGATCGGTGATTGGGACTAAGTCGTA
ACAAGGTAGCCGTACCGGAAGGTGCGGCTGGATCACCTCCTTT
>66878893|gb|AY959187.1|Brachybacterium|Uncultured bacterium clone
rRNA414 16S ribosomal RNA gene, partial sequence
CGCCCTTAGAGTTTGATCCTGGCTCAGGACGAACGCTGGCGGCGTGCTTAACACATGCGAGTCGAACGAT
GACGGTGGTGCTTGCATCACCTGATTAGTGGCGAACGGGTGAGTAACACGTGAGTAACCTGCCCTTCACT
TCGGGATAACCTCGGGAAATCGTGGCTAATACCGGATATGAGCTCCTGCCGCATGGTGGGGGTTGGAAAG
ATTTATCGGTGAAGGATGGACTCGCGGCCTATCAGTTTGTTGGTGAGGTAATGGCTCACCAAGGCGATGA
CGGGTAGCCGGCCTGAGAGGGCGACCGGCCACACTGGGACTGAGACACGGCCCAGACTCCTACGGGAGGC
AGCAGTGGGGAATATTGCACAATGGGCGAAAGCCTGATGCAGCGACGCCGCGTGGGGGATGACGGCCTTC
GGGTTGTAAACCCCTTTCAGTAGGGAAGAAGCGAAAGTGACGGTACCTGCAGAAGAAGCGCCGGCTAACT
ACGTGCCAGCAGCCGCGGTAATACGTAGGGCGCAAGCGTTGTCCGGAATTATTGGGCGTAAAGAGCTCGT
AGGTGGCTTGTCGCGTCTGCCGTGAAAACCCGAGGCTCAACCTCGGGCGTGCGGTGGGTACGGGCAGGCT
AGAGTGTGGTAGGGGAGACTGGAACTCCTGGTGTAGCGGTGAAATGCGCAGATATCAGGAAGAACACCGA
TGGCGAAGGCAGGTCTCTGGGCCATTACTGACACTGAGGAGCGAAAGCATGGGTAGCGAACAGGATTAGA
TACCCTGGTAGTCCATGCCGTAAACGTTGGGCACTAGGTGTGGGGGACATTCCACGTTTTCCGCGCCGTA
GCTAACGCATTAAGTGCCCCGCCTGGGGAGTACGGCCGCAAGGCTAAAACTCAAAGGAATTGACGGGGGC
CCGCACAAGCGGCGGAGCATGCTGATTAATTCGATGCAACGCGAAGAACCTTACCAAGGCTTGACATGCA
CCGGACGGCCGCAGAGATGTGGCTTTCTTCGGACTTGTGCACAGGTGGTGCATGGTTGTCGTCAGCTCGT
GTCGTGAGATGTTGGGTTAAGTCCCGCAACGAGCGCAACCCTTGTTCTATGTTGCCAGCACGTGATGGTG
GGGACTCATAGGAGACTGCCGGGGTCAACTCGGAGGAAGGTGGGGACGACGTCAAATCATCATGCCCCTT
ATGTCTTGGGCTTCAAGCATGCTACAATGGTCGGTACAATGGGTTGCGAAACTGTGAGGTGGAGCGAATC
CCAAAAAGCCGGCCTCAGTTCGGATTGGGGTCTGCAACTCGACCCCATGAAGTCGGAGTCGCTAGTAATC
GCAGATCAGCAACGCTGCGGTGAATACGTTCCCGGGCCTTGTACACACCGCCCGTCAAGTCACGAAAGTC
GGTAACACCCGAAGCCAGTGGCCCATCCTTGTGAGGGAGCTGTCGAAGGTGGGATCGGTGATTGGGACTA
A

>299818468|gb|HM222676.1|Kocuria|Kocuria sp. 0712C1-3 16S ribosomal RNA gene, partial sequence
AGAGTTTGATCCTGGCTCAGGACGAACGCTGGCGGCGTGCTTAACACATGCAAGTCGAACGCTGAAGCAC
CAGCTTGCTGGTGTGGATGAGTGGCGAACGGGTGAGTAATACGTGAGTAACCTGCCCTTGACTCTGGGAT
AAGCCCGGGAAACTGGGTCTAATACTGGATGCTACATGTCACCGCATGGTGGTGTGTGGAAAGGGTTTAC
TGGTCTTGGATGGGCTCACGGCCTATCAGCTTGTTGGTGAGGTAATGGCTCACCAAGGCGACGACGGGTA
GCCGGCCTGAGAGGGTGACCGGCCACACTGGGACTGAGACACGGCCCAGACTCCTACGGGAGGCAGCAGT
GGGGAATATTGCACAATGGGCGAAAGCCTGATGCAGCGACGCCGCGTGAGGGATGACGGCCTTCGGGTTG
TAAACCTCTTTCAGCAGGGAAGAAGCCACAAGTGACGGTACCTGCAGAAGAAGCGCCGGCTAACTACGTG
CCAGCAGCCGCGGTAATACGTAGGGCGCAAGCGTTGTCCGGAATTATTGGGCGTAAAGAGCTCGTAGGCG
GTTTGTCGCGTCTGCTGTGAAAGCCCGGGGCTTAACCCCGGGTGTGCAGTGGGTACGGGCAGACTAGAGT
GCAGTAGGGGAGACTGGAATTCCTGGTGTAGCGGTGGAATGCGCAGATATCAGGAGGAACACCGATGGCG
AAGGCAGGTCTCTGGGCTGTTACTGACGCTGAGGAGCGAAAGCATGGGGAGCGAACAGGATTAGATACCC
TGGTAGTCCATGCCGTAAACGTTGGGCACTAGGTGTGGGGGACATTCCACGTTTTCCGCGCCGTAGCTAA
CGCATTAAGTGCCCCGCCTGGGGAGTACGGCCGCAAGGCTAAAACTCAAAGGAATTGACGGGGGCCCGCA
CAAGCGGCGGAGCATGCGGATTAATTCGATGCAACGCGAAGAACCTTACCAAGGCTTGACATATACCGGA
TCGTTCCAGAGATGGTTCTTCCCCTTTGGGGTCGGTATACAGGTGGTGCATGGTTGTCGTCAGCTCGTGT
CGTGAGATGTTGGGTTAAGTCCCGCAACGAGCGCAACCCTCGTTCCATGTTGCCAGCACGTGATGGTGGG
GACTCATGGGAGACTGCCGGGGTCAACTCGGAGGAAGGTGGGGATGACGTCAAATCATCATGCCCCTTAT
GTCTTGGGCTTCACGCATGCTACAATGGCCGGTACAAAGGGTTGCGATACTGCGAGGTGGAGCTAATCCC
AAAAAGCCGGTCTCAGTTCGGATTGAGGTCTGCAACTCGACCTCATGAAGTCGGAGTCGCTAGTAATCGC
AGATCAGCAACGCTGCGGTGAATACGTTCCCGGGCCTTGTACACACCGCCCGTCAAGTCACGAAAGTCGG
TAACACCCGAAGCCGGTGGCCCAACCCTTGTGGAGGGAGCCGTCGAAGGTGGGACTGGCGATTGGGACTA
AGTCGTAACAAGGTAACC
>66878593|gb|AY958887.1|Kocuria|Uncultured bacterium clone rRNA114 16S ribosomal RNA gene, partial sequence
CCCTTAGAGTTTGATCCTGGCTCAGGACGAACGCTGGCGGCGTGCTTAACACATGCAAGTCGAACGCTGA
AGCACCAGCTTGCTGGTGTGGATGAGTGGCGAACGGGTGAGTAATACGTGAGTAACCTGCCCTTGACTCT
GGGATAAGCCCGGGAAACTGGGTCTAATACTGGATGCTACATGTCACCGCATGGTGGTGTGTGGAAAGGG
TTTACTGGTCTTGGATGGGCTCACGGCCTATCAGCTTGTTGGTGAGGTAATGGCTCACCAAGGCGACGAC
GGGTAGCCGGCCTGAGAGGGTGACCGGCCACACTGGGACTGAGACACGGCCCAGACTCCTACGGGAGGCA
GCAGTGGGGAATATTGCACAATGGGCGAAAGCCTGATGCAGCGACGCCGCGTGAGGGATGACGGCCTTCG
GGTTGTAAACCTCTTTCAGCAGGGAAGAAGCCACAAGTGACGGTACCTGCAGAAGAAGCGCCGGCTAACT
ACGTGCCAGCAGCCGCGGTAATACGTAGGGCGCAAGCGTTGTCCGGAATTATTGGGCGTAAAGAGCTCGT
AGGCGGTTTGTCGCGTCTGCTGTGAAAGCCCGGGGCTTAACCCCGGGTGTGCAGTGGGTACGGGCAGACT
AGAGTGCAGTAGGGGAGACTGGAATTCCTGGTGTAGCGGTGGAATGCGCAGATATCAGGAGGAACACCGA
TGGCGAAGGCAGGTCTCTGGGCTGTTACTGACGCTGAGGAGCGAAAGCATGGGGAGCGAACAGGATTAGA
TACCCTGGTAGTCCATGCCGTAAACGTTGGGCACTAGGTGTGGGGGACATTCCACGTTTTCCGCGCCGTA
GCTAACGCATTAAGTGCCCCGCCTGGGGAGTACGGCCGCAAGGCTAAAACTCAAAGGAATTGACGGGGGC
CCGCACAAGCGGCGGAGCATGCGGATTAATTCGATGCAACGCGAAGAACCTTACCAAGGCTTGACATATA
CCGGATCGTTCCAGAGATGGTTCTTCCCCTTTGGGGTCGGTATACAGGTGGTGCATGGTTGTCGTCAGCT
CGTGTCGTGAGATGTTGGGTTAAGTCCCGCAACGAGCGCAACCCTCGTTCCATGTTGCCAGCACGTGATG
GTGGGGACTCATGGGAGACTGCCGGGGTCAACTCGGAGGAAGGTGGGGATGACGTCAAATCATCATGCCC
CTTATGTCTTGGGCTTCACGCATGCTACAATGGCCGGTACAAAGGGTTGCGATACTGTGAGGTGGAGCTA
ATCCCAAAAAGCCGGTCTCAGTTCGGATTGAGGTCTGCAACTCGACCTCATGAAGTCGGAGTCGCTAGTA
ATCGCAGATCAGCAACGCTGCGGTGAATACGTTCCCGGGCCTTGTACACACCGCCCGTCAAGTCACGAAA
GTCGGTAACACCCGAAGCCGGTGGCCCAACCCTTGTGGAGGGAGCCGTCGAAGGTGGGACTGGCGATTGG
GACTAA
>119721297|gb|EF151509.1|Nesterenkonia|Nesterenkonia sp. YIM 90721 16S ribosomal RNA gene, partial sequence (95%)
CGATTAGAGTTTGATCCTGGCTCAGGATGAACGCTGGCGGCGTGCTTAACACATGCAAGTCGAACGATGA
AGCCCGTGCTTGCACGGGTGGATTAGTGGCGAACGGGTGAGTATCACGTGAGTAACCTGCCCTTGACTCT
GGGATAAGCCTGGGAAACTGGGTCTAATACCGGATATGACCAGTCCTCGCATGAGGTGCTGGTGGAAAGA
TTTTATCGGTCTTGGATGGACTCGCGGCCTATCAGCTAGACGGTGAGGTAACGGCTCACCGTGGCGATGA

CGGGTAGCCGGCCTGAGAGGGTGACCGGCCACACTGGGACTGAGACACGGCCCAGACTCCTACGGGAGGC
AGCAGTGGGGAATATTGCACAATGGGCGCAAGCCTGATGCAGCGACGCCGCGTGCGGGATGACGGCCTTC
GGGTTGTAAACCGCTTTCAGCAGGGAAGAAGCTTTTGTGACGGTACCTGCAGAAGAAGCGCCGGCTAACT
ACGTGCCAGCAGCCGCGGTAATACGTAGGGCGCGAGCGTTATCCGGAATTATTGGGCGTAAAGAGCTCGT
AGGCGGCTTGTCGCGTCTGCTGTGAAAGCCCGGGGCTTAACCCCGGGTGTGCAGTGGGTACGGGCAGGCT
AGAGTGCAGTAGGGGAGACTGGAATTCCTGGTGTAGCGGTGAAATGCGCAGATATCAGGAGGAACACCGA
TGGCGAAGGCAGGTCTCTGGGCTGTTACTGACGCTGAGGAGCGAAAGCATGGGGAGCGAACAGGATTAGA
TACCCTGGTAGTCCATGCCGTAAACGTTGGGCACTAGGTGTGGGGGACATTCCACGTTTTCCGCGCCGTA
GCTAACGCATTAAGTGCCCCGCCTGGGGAGTACGGCCGCAAGGCTAAAACTCAAAGGAATTGACGGGGGC
CCGCACAAGCGGCGGAGCATGCGGATTAATTCGATGCAACGCGAAGAACCTTACCAAGGCTTGACATGGA
CCGGATCGCTGCAGAGATGTAGTTTCCCTTCGGGGCTGGTTCACAGGTGGTGCATGGTTGTCGTCAGCTC
GTGTCGTGAGATGTTGGGTTAAGTCCCGCAACGAGCGCAACCCTTGTCCTATGTTGCCAGCACGTAATGG
TGGGGACTCATGGGAGACTGCCGGGGTCAACTCGGAGGAAGGTGGGGATGACGTCAAATCATCATGCCCC
TTATGTCTTGGGCTTCACGCATGCTACAATGGCCGGTACAGTGGGTTGCGATACTGTGAGGTGGAGCTAA
TCCCTAAAAGCCGGTCTCAGTTCGGATCGAAGTCTGCAACTCGACTTCGTGAAGTTGGAGTCGCTAGTAA
TCGCAGATCAGCAACGCTGCGGTGAATACGTTCCCGGGCCTTGTACACACCGCCCGTCAAGTCACGAAAG
TTGGTAACACCCGAAGCCCACGGCCCAACCGGTTTTCCGGGGGGAGTGGTCGAAGGTGGGACTGGCGATT
GGGACTAAGTCGTAACAAGGTAGCCGTACCGGAAGGTGCGGCTGGATCA
>66878582|gb|AY958876.1|Nesterenkonia|Uncultured bacterium clone rRNA103
16S ribosomal RNA gene, partial sequence
ACGAGCGCTGGCGGCGTGCCTAATACATGCAAGTCGAACGATGAAGCTGCCTGCTTGCAGGTGGTGGATT
AGTGGCGAACGGGTGAGTATCACGTGAGTAACCTGCCCTTGACTCTGGGATAAGCCCGGGAAACTGGGTC
TAATACCGGATATGACCTCTCATCGCATGGTGGGGGGTGGAAAGTTTTTAACGGTCTTGGATGGGCTCGC
GGCCTATCAGCTTGACGGTGGGGTAGTGGCCTACCGTGGCTTTGACGGGTAGCCGGCCTGAGAGGGTGAC
CGGCCACACTGGGACTGAGACACGGCCCAGACTCCTACGGGAGGCAGCAGTGGGGAATATTGCACAATGG
GCGCAAGCCTGATGCAGCGACGCCGCGTGCGGGATGACGGCCTTCGGGTTGTAAACCGCTTTCAGTACAG
AAGAAGCCCTTTTTGGGTGACGGTATGTGCAGAAGAAGCGCCGGCTAACTACGTGCCAGCGGCCGCGGTA
ATACGTAGGGCGCGAGCGTTATCCGGAATTATTGGGCGTAAAGAGCTTGTAGGCGGTTTGCCGCGTCTGC
TGTGAAAGCCCGGGGCTTAACTCCGGGTGTGCAGTGGGTACGGGCAGGCTAGAGTGCAGTAGGGGAGACT
GGAATTCCTGGTGTAGCGGTGAAATGCGCAGATATCAGGAGGAACACCGATGGCGAAGGCAGGTCTCTGG
GCTGTTTCTGACGCTGAGAAGCGAAAGCATGGGTAGCGAACAGGATTAGATACCCTGGTAGTCCATGCTG
TAAACGTTGGGCACTAGGTGTGGGGGGCGTTCCTCGTCGTCCGCGCCGTAGCTAACGCATTAAGTGCCCC
GCCTGGGGAGTACGGCCGCAAGGCTAAAACTCAAAGGAATTGACGGGGGCCCGCACAAGCGGCGGAGCAT
GCGGATTAATTCGATGCAACGCGAAGAACCTTACCAAGGCTTGACATACACCGGATCGCGCTGGAGACAG
TGTTTCCCTTCGGGGCTGGTGTACAGGTGGTGCATGGTTGTCGTCAGCTCGTGTCGTGAGATGTTGGGTT
AAGTCCCGCAACGAGCGCAACCCTTGTCCTATGTTGCCAGCACGTAGTGGTGGGGACTCATGGGAGACTG
CCGGGGTCAACTCGGAGGAAGGTGGGGATGACGTCAAATCATCATGCCCCTTATGTCTTGGGCTTCACGC
ATGCTACAATGGCTGGTACAGTGGGTTGCGATACCGTGAGGTGGAGCTAATCCCTGAAAGCTGGTCTCAG
TTCGGATCGAAGTCTGCAACTCGACTTCGTGAAGTTGGAGTCGCTAGTAATCGCAGATCAGCAATGCTGC
GGTGAATACGTTCCCGGGCCTTGTACACACCGCCCGTCAAGTCACGAAAGTTGGTAACACCCGAAGCTCA
CGGCCTAACCGGTTTTTCCGGGGGGAGTGGTCGAAGGCGGGACTGGCGATTGGGACTAAGTCGTAACAAG
GTAACCGTAAAGGGC
>66878837|gb|AY959131.1|Nesterenkonia|Uncultured bacterium clone rRNA358
16S ribosomal RNA gene, partial sequence
GCCCTTAGAGTTTGATCCTGGCTCAGGATGAACGCTGGCGGCGTGCTTAACACATGCAAGTCGAACGATG
ATGCTTCCAGCTTTGCTGGGGGTGGATTAGTGGCGAACGGGTGAGTATCACGTGAGTAACCTGCCCTTGA
CTCTGGGATAAGCCCGGGAAACTGGGTCTAATACCGGATAGGACTTCGCATCGCATGGTGTGGGGTTGAA
AGTTTTTAACGGTCTTGGATGGGCTCGCGGCCTATCAGTTTGACGGTGGGGTAGTGGCCTACCGTGGCGA
TGACGGGTAGCCGGCCTGAGAGGGTGACCGGCCACACTGGGACTGAGACACGGCCCAGACTCCTACGGGA
GGCAGCAGTGGGGAATATTGCACAATGGGCGCAAGCCTGATGCAGCGACGCCGCGTGCGGGATGACGGCC
TTCGGGTTGTAAACCGCTTTCAGTAGGGACGAAGCCCTTCGGGGTGACGGTACTTGCAGAAGAAGCGCCG
GCTAACTACGTGCCAGCAGCCGCGGTAATACGTAGGGCGCGAGCGTTATCCGGAATTATTGGGCGTAAAG
AGCTTGTAGGCGGTTTGTCACGTCTGCTGTGAAAGCCCGGGGCTCAACTCCGGGTGTGCAGTGGGTACGG
GCTGACTAGAGTGCAGTAGGGGAGACTGGAATTCCTGGTGTAGCGGTGAAATGCGCAGATATCAGGAGGA

ACACCGATGGCGAAGGCAGGTCTCTGGGCTGTTTCTGACGCTGAGAAGCGAAAGCATGGGTAGCGAACAG
GATTAGATACCCTGGTAGTCCATGCCGTAAACGTTGGGCACTAGGTGTGGGGGGCGTTCCTCGTCGTCCG
CGCCGTAGCTAACGCATTAAGTGCCCCGCCTGGGGAGTACGGCCGCAAGGCTAAAACTCAAAGGAATTGA
CGGGGGCCCGCACAAGCGGCGGAGCATGCGGATTAATTCGATGCAACGCGAAGAACCTTACCAAGGCTTG
ACATACACCGGACCGCCCTAGAGATAGGGCTTCCCTTCGGGGCTGGTGTACAGGTGGTGCATGGTTGTCG
TCAGCTCGTGTCGTGAGATGTTGGGTTAAGTCCCGCAACGAGCGCAACCCTTGTCCTATGTTGCCAGCAC
GTAATGGTGGGGACTCATGGGAGACTGCCGGGGTCAACTCGGAGGAAGGTGGGGATGACGTCAAATCATC
ATGCCCCTTATGTCTTGGGCTTCACGCATGCTACAATGGCCGGTACAGTGGGTTGCGATACTGTGAAGTG
GAGCTAATCCCTAAAAGCCGGTCTCAGTTCGGATCGAAGTCTGCAACTCGACTTCGTGAAGTTGGAGTCG
CTAGTAATCGCAGATCAGCAATGCTGCGGTGAATACGTTCCCGGGCCTTGTACACACCGCCCGTCAAGTC
ACGAAAGTTGGTAACACCCGAAGCCGGTGGCCCAGCCCTTGTGGGGGGAGCCGTCGAAGGTGGGACGAGC
GATTGGGACTAA
>219878136|ref|NR_025275.1|Propionimicrobium|Propionimicrobium lymphophilum strain DSM 4903 16S ribosomal RNA, partial sequence
TCAGGACGAACGCTGGCGGCGTGCTTAACACATGCAAGTCGAGCGGTAAGGCCCTTTCGGGGGTACACGA
GCGGCGAACGGGTGAGTAACGCGTGAGTAACCTGCCCCCCTCTCTGGGATAACAGTTGGAAACGGCTGCT
AATACCGGATATTCAGACCTTTAGGCATCTTTTGGTTTGGAAAGTTCTGGCGGTGGGGGATGACTCGCGT
CCTATCAGCTTGTTGGTGGGGTAGTGGCCTACCAAGGCGACGACGGGTAGCCGGCCTGAGAGGGCGACCG
GCCACATTGGGACTGAGATACGGCCCAAACTCCTACGGGAGGCAGCAGTGGGGAATATTGCACAATGGGG
GAAACCCTGATGCAGCAACGCGCGTGCGGGATGACGGCCTTCGGGTTGTAAACCGCTTTCAGCCATGACG
AAGCTTTTGTGACGGTAGTGGCAGAAGAAGCACCGGCTAACTACGTGCCAGCAGCCGCGGTGATACGTAG
GGTGCGAGCGTTGTCCGGAATTATTGGGCGTAAAGAGCTTGTAGGCGGTTTGTCGCGTCGAAAGTGTAAA
CTCAGTGCTTAACGCTGAGCCTGCTTTCGATACGGGTTGACTAGAGGAAGGTAGGGGAGAATGGAATTCC
CGGTGGAGCGGTGGAATGCGCAGATATCGGGAGGAACACCAGTGGCGAAGGCGGTTCTCTGGACCTTTCC
TGACGCTGAGAAGCGAAAGCGTGGGGTAGCAAACAGGCTTAGATACCCTGGTAGTCCACGCCGTAAACGG
TGGGTACTAGGTGTGGGGGACATTCCACGTTCTCTGTGCCGTAGCTAACGCATTAAGTACCCCGCCTGGG
GAGTACGGCCGCAAGGTAAAACTCAAAGGAATTGACGGGGCCCCGCACAAGCGGCGGAGCATGCGGATTA
ATTCGATGCAACGCGAAGAACCTTACCTGGGTTTGAAATATACCGGAAACGTCTAGAGATAGGCGCCCCG
TAAGGTCGGTATACAGGTGGTGCATGGCTGTCGTCAGCTCGTGTCGTGAGATGTTGGGTTAAGTCCCGCA
ACGAGCGCAACCCTCGTCTAATGTTGCCAGCAAGTTATGTTGGGGACTCGTTAGAGACCGCCGAGGTCAA
CTCGGAGGAAGGTGAGGACGACGTCAAGTCATCATGCCCCTTATGTCCAGGGCTTCACGCATGCTACAAT
GGTCGGTACAGTCAGTTGCGAGCCTGTAAGGGTTAGCGAATCTGTAAAAGCCGGCCTCAGTTCGGATTGG
GGTCTGCAACTCGACCCCATGAAGTCGGAGTCGCTAGTAATCGCAGATCAGCAACGCTGCGGTGAATACG
TTCCCGGGGCTTGTACACACCGCCCGTCAAGTCATGAAAGTTGGCAACACCCGAAGCCAGTGGCCTAACC
CTTGTGGGGGGGGAGCTGTCGAAGGTGGGGCTGATAATTAGGACTAAGTCGTAACAAGGTAGCCGTACCG
GAAGGTGCGGCTGGATCACCTCCTTTCTAAGG
>66878575|gb|AY958869.1|Propionimicrobium|Uncultured bacterium clone rRNA096 16S ribosomal RNA gene, partial sequence
GCCCTTAGAGTTTGATCCTGGCTCAGGACGAACGCTGGCGGCGTGCTTAACACATGCAAGTCGAGCGGTA
AGGCCCTTTCGGGGGTACACGAGCGGCGAACGGGTGAGTAACGCGTGAGTAACCTGCCCCCCTCTCTGGG
ATAACAGTTGGAAACGGCTGCTAATACCGGATATTCAGACCTTTAGGCATCTTTTGGTTTGGAAAGTTCT
GGCGGTGGGGGATGGACTCGCGTCCTATCAGCTTGTTGGTGGGGTAGTGGCCTACCAAGGCGACGACGGG
TAGCCGGCCTGAGAGGGCGACCGGCCACATTGGGACTGAGATACGGCCCAAACTCCTACGGGAGGCAGCA
GTGGGGAATATTGCACAATGGGGGAAACCCTGATGCAGCAACGCCGCGTGCGGGATGACGGCCTTCGGGT
TGTAAACCGCTTTCAGCCATGACGAAGCTTTTGTGACGGTAGTGGCAGAAGAAGCACCGGCTAACTACGT
GCCAGCAGCCGCGGTGATACGTAGGGTGCGAGCGTTGTCCGGAATTATTGGGCGTAAAGAGCTTGTAGGC
GGTTTGTCGCGTCGAAAGTGTAAACTCAGTGCTTAACGCTGAGCCTGCTTTCGATACGGGCTGACTAGAG
GAAGGTAGGGGAGAATGGAATTCCCGGTGGAGCGGTGGAATGCGCAGATATCGGGAGGAACACCAGTGGC
GAAGGCGGTTCTCTGGACCTTTCCTGACGCTGAGAAGCGAAAGCGTGGGTAGCAAACAGGCTTAGATACC
CTGGTAGTCCACGCCGTAAACGGTGGGTACTAGGTGTGGGGGACATTCCACGTTCTCTGTGCCGTAGCTA
ACGCATTAAGTACCCCGCCTGGGGAGTACGGCCGCAAGGCTAAAACTCAAAGGAATTGACGGGGCCCCGC
GCAAGCGGCGGAGCATGCGGATTAATTCGATGCAACGCGAAGAACCTTACCTGGGTTTGAGATATACCGG
AAACGTCTAGAGATAGGCGCCCCGTAAGGTCGGTATACAGGTGGTGCATGGCTGTCGTCAGCTCGTGTCG
TGAGATGTTGGGTTAAGTCCCGCAACGAGCGCAACCCTCGTCTAATGTTGCCAGCAAGTTATGTTGGGGA

CTCGTTAGAGACCGCCGAGGTCAACTCGGAGGAAGGTGAGGACGACGTCAAGTCATCATGCCCCTTATGT
CCAGGGCTTCACGCATGCTACAATGGTCGGTACAGTCAGTTGCGAGCCTGTAAGGGTTAGGGAATCTGTA
AAAGCCGGCCTCAGTTCGGATTGGGGTCTGCAACTCGACCCCATGAAGTCGGAGTCGCTAGTAATCGCAG
ATCAGCAACGCTGCGGTGAATACGTTCCCGGGGCTTGTACACACCGCCCGTCAAGTCATGAAAGTTGGCA
ACACCCGAAGCCAGTGGCCTAACCCTTGTGGGGGGAGCTGTCGAAGGTGGGGCTGATAATTAGGACTAAG
TCGTAACAAGGTAACCGTAAAGGG
>301072772|gb|HM596282.1|Scardovia|Scardovia wiggsiae strain F0424 16S ribosomal RNA gene, partial sequence
GGGTTCGATTCTGGCTCAGGACGAACGCTGGCGGCGTGCTTAACACATGCAAGTCGAACGGGATCCACTG
GGCTTTTGTTTGGTGGTGAGAGTGGCGAACGGGTGAGTAATGCGTGACTAACCTGCCGTATGGTTGGGGA
TAGCTCCTGGAAACGGGTGGTAATACCCAATGCTCCAGCTGGATGCATGTCTGGTTGGGAAAGCTTTTTG
TGCCATATGATGGGGTCGCGTCCTATCAGCTTGTTGGTGGGGTGATGGCCTACCAAGGCGTCGACGGGTA
GCCGGCCTAAGAGGGCGACCGGCCACATTGGGACTGAGATACGGCCCAGACTCCTACGGGAGGCAGCAGT
GGGGAATATTGCACAATGGGCGCAAGCCTGATGCAGCGACGCCGCGTGCGGGATGGAGGCCTTCGGGTTG
TAAACCGCTTTTATAGGGGGGCAAGCTATGCCTGTGTGGTGTGGTGAGTGGACTTTATGAATAAGCACCG
GCTAACTACGTGCCAGCAGCCGCGGTAATACGTAGGGTGCAAGCGTTGTCCGGATTTATTGGGCGTAAAG
GGCTCGTAGGCGGTTTGTTGCGTCTGGTGTGAAAGCTTACTGCTTAACGGTAGGTTGCGCTGGATACGGG
CAGGCTTGAGTGCAGTAGGGGAGACTGGAATTCTCGGTGTAACGGTGGAATGTGTAGATATCGGGAAGAA
CACCTATGGCGAAGGCAGGTCTCTGGGCTGTTACTGACGCTGAGGAGCGAAAGCGTGGGGAGCGAACAGG
ATTAGATACCCTGGTAGTCCATGCTGTAAACGGTGGACGCTGGATGTGGGGCCCATTTCCACGGGTTCTG
TGTCGGAGCTAACGCGTTAAGCGTCCCGCCTGGGGAGTACGGCCGCAAGGTTAAAACTCAAAGAAATTGA
CGGGGGCCCGCACAAGCGGCGGAGCATGCGGATTAATTCGATGCAACGCGAAGAACCTTACCAGGGCTTG
ACATAACCTGGATGATGCCAGAGATGGTGTGTCCCTTCGGGGCTGGGTTACAGGTGGTGCATGGTCGTCG
TCAGCTCGTGTCGTGAGATGTTGGGTTAAGTCCCGCAACGAGCGCAACCCTTGCCTTGTGTTACCAGCGG
GTCGTGCCGGGGACTCACAAGGGACCGCCGGGGTTAACTCGGAGGAAGGTGGGGATGACGTCAGATCATC
ATGCCCCTTACGTCCTGGGCTTCACGCATGCTACAATGGCTGGTACAGCGGGATGCGATACTGTAAGGTG
GAGCGGATCCTGTAAAACCGGTCTCAGTTCGGATCGGGGCCTGCAACTCGGCCTCGTGAAGGTGGAGTCG
CTAGTAATCGCGGATCAGCAGTGCCGCGGTGAATGCGTTCCCGGGCCTTGTACACACCGCCCGTCAAGTC
ATGAAAGTGGGCAGCACCCGAAGCCGGTGGCCTAACCTGTTGTGGGGGGAGCCGTCTAAGGTGAGGTTCG
CGATTGGGACTAAGTCGTAACAAGGTAGCCGTACCGGAAGGTGC
>66878608|gb|AY958902.1|Scardovia|Uncultured bacterium clone rRNA129 16S ribosomal RNA gene, partial sequence
CGCCCTTAGAGTTTGATCCTGGCTCAGGACGAACGCTGGCGGCGTGCTTAACACATGCAAGTCGAACGGG
ATCCACTGGGCTTTTGTTTGGTGGTGAGAGTGGCGAACGGGTGAGTAATGCGTGACTAACCTGCCGTATG
GTTGGGGATAGCTCCTGGAAACGGGTGGTAATACCCAATGCTCCAGCTGGATGCATGTCTGGTTGGGAAA
GCTTTTGTGCCATATGATGGGGTCGCGTCCTATCAGCTTGTTGGTGGGGTGATGGCCTACCAAGGCGTCG
ACGGGTAGCCGGCCTAAGAGGGCGACCGGCCACATTGGGACTGAGATACGGCCCAGACTCCTACGGGAGG
CAGCAGTGGGGAATATTGCACAATGGGCGCAAGCCTGATGCAGCGACGCCGCGTGCGGGATGGAGGCCTT
CGGGTTGTAAACCGCTTTTATAGGGGGGCAAGCTATGCCTGTGTGGTGTGGTGAGTGGACTTTATGAATA
AGCACCGGCTAACTACGTGCCAGCAGCCGCGGTAATACGTAGGGTGCAAGCGTTGTCCGGATTTATTGGG
CGTAAAGGGCTCGTAGGCGGTTTGTTGCGTCTGGTGTGAAAGCTTACTGCTTAACGGTAGGTTGCGCTGG
ATACGGGCAGGCTTGAGTGCAGTAGGGGAGACTGGAATTCTCGGTGTAACGGTGGAATGTGTAGATATCG
GGAAGAACACCTATGGCGAAGGCAGGTCTCTGGGCTGTTACTGACGCTGAGGAGCGAAAGCGTGGGGAGC
GAACAGGATTAGATACCCTGGTAGTCCATGCTGTAAACGGTGGACGCTGGATGTGGGGCCCATTTCCACG
GGTTCTGTGTCGGAGCTAACGCGTTAAGCGTCCCGCCTGGGGAGTACGGCCGCAAGGTTAAAACTCAAAG
AAATTGACGGGGGCCCGCACAAGCGGCGGAGCATGCGGATTAATTCGATGCAACGCGAAGAACCTTACCA
GGGCTTGACATAACCTGGATGATGCCAGAGATGGTGTGTCCCTTCGGGGCTGGGTTACAGGTGGTGCATG
GTCGTCGTCAGCTCGTGTCGTGAGATGTTGGGTTAAGTCCCGCAACGAGCGCAACCCTTGCCTTGTGTTA
CCAGCGGGTCGTGCCGGGGACTCACAAGGGACCGCCGGGGTTAACTCGGAGGAAGGTGGGGATGACGTCA
GATCATCATGCCCCTTACGTCCTGGGCTTCACGCATGCTACAATGGCTGGTACAGCGGGATGCGATACTG
TAAGGTGGAGCGGATCCTGTAAAACCGGTCTCAGTTCGGATCGGGGCCTGCAACTCGGCCTCGTGAAGGT
GGAGTCGCTAGTAATCGCGGATCAGCAGTGCCGCGGTGAATGCGTTCCCGGGCCTTGTACACACCGCCCG
TCAAGTCATGAAAGTGGGCAGCACCCGAAGCCGGTGGCCTAACCTATGTGGGGGGAGCCGTCTAAGGTGA
GGTTCGCGATTGGGACTAAGTCGTAACAAGGTAACCGTAAAGGGC

>291419623|gb|GU733463.1|Janthinobacterium|Janthinobacterium sp. Lc10-10 16S ribosomal RNA gene, partial sequence
AGAGTTTGATCCTGGCTCAGATTGAACGCTGGCGGCATGCCTTACACATGCAAGTCGAACGGCAGCACGG
AGCTTGCTCTGGTGGCGAGTGGCGAACGGGTGAGTAATATATCGGAACGTACCCTAGAGTGGGGGATAAC
GTAGCGAAAGTTACGCTAATACCGCATACGATCTAAGGATGAAAGTGGGGGATCGCAAGACCTCATGCTC
GTGGAGCGGCCGATATCTGATTAGCTAGTTGGTAGGGTAAAAGCCTACCAAGGCATCGATCAGTAGCTGG
TCTGAGAGGACGACCAGCCACACTGGAACTGAGACACGGTCCAGACTCCTACGGGAGGCAGCAGTGGGGA
ATTTTGGACAATGGGCGAAAGCCTGATCCAGCAATGCCGCGTGAGTGAAGAAGGCCTTCGGGTTGTAAAG
CTCTTTTGTCAGGGAAGAAACGGTGAGAGCTAATATCTTTTGCTAATGACGGTACCTGAAGAATAAGCAC
CGGCTAACTACGTGCCAGCAGCCGCGGTAATACGTAGGGTGCAAGCGTTAATCGGAATTACTGGGCGTAA
AGCGTGCGCAGGCGGTTTTGTAAGTCTGATGTGAAATCCCCGGGCTCAACCTGGGAATTGCATTGGAGAC
TGCAAGGCTAGAATCTGGCAGAGGGGGGTAGAATTCCACGTGTAGCAGTGAAATGCGTAGATATGTGGAG
GAACACCGATGGCGAAGGCAGCCCCCTGGGTCAAGATTGACGCTCATGCACGAAAGCGTGGGGAGCAAAC
AGGATTAGATACCCTGGTAGTCCACGCCCTAAACGATGTCTACTAGTTGTCGGGTCTTAATTGACTTGGT
AACGCAGCTAACGCGTGAAGTAGACCGCCTGGGGAGTACGGTCGCAAGATTAAAACTCAAAGGAATTGAC
GGGGACCCGCACAAGCGGTGGATGATGTGGATTAATTCGATGCAACGCGAAAAACCTTACCTACCCTTGA
CATGGCTGGAATCCTTGAGAGATCGAGGGAGTGCTCGAAAGAGAACCAGTACACAGGTGCTGCATGGCTG
TCGTCAGCTCGTGTCGTGAGATGTTGGGTTAAGTCCCGCAACGAGCGCAACCCTTGTCATTAGTTGCTAC
GAAAGGGCACTCTAATGAGACTGCCGGTGACAAACCGGAGGAAGGTGGGGATGACGTCAAGTCCTCATGG
CCCTTATGGGTAGGGCTTCACACGTCATACAATGGTACATACAGAGCGCCGCCAACCCGCGAGGGGGAGC
TAATCGCAGAAAGTGTATCGTAGTCCGGATTGTAGTCTGCAACTCGACTGCATGAAGTTGGAATCGCTAG
TAATCGCGGATCAGCATGTCGCGGTGAATACGTTCCCGGGTCTTGTACACACCGCCCGTCACACCATGGG
AGCGGGTTTTACCAGAAGTAGGTAGCTTAACCGCAAGGAGGGCGCTTACCACGGTAGGATTCGTGACTGG
GGTGAAGTCGTAACAAGGTAGCCGTATCGGAAGGTGCGGCTGGATCACCTCCTTAC
>66878491|gb|AY958785.1|Janthinobacterium|Uncultured bacterium clone rRNA012 16S ribosomal RNA gene, partial sequence
CCCTTAGAGTTTGATCCTGGCTCAGATTGAACGCTGGCGGCATGCCTTACACATGCAAGTCGAACGGCAG
CACGGAGCTTGCTCTGGTGGGGAGTGGCGAACGGGTGAGTAATATATCGGAACGTACCCTAGAGTGGGGG
ATAACGTAGCGAAAGTTACGCTAATACCGCATACGATCTAAGGATGAAAGTGGGGGATCGCAAGACCTCA
TGCTCGTGGAGCGGCCGATATCTGATTAGCTAGTTGGTAGGGTAAAAGCCTACCAAGGCATCGATCAGTA
GCTGGTCTGAGAGGACGACCAGCCACACTGGAACTGAGACACGGTCCAGACTCCTACGGGAGGCAGCAGT
GGGGAATTTTGGACAATGGGCGAAAGCCTGATCCAGCAATGCCGCGTGAGTGAAGAAGGCCTTCGGGTTG
TAAAGCTCTTTTGTCAGGGAAGAAACGGTGAGAGCTAATATCTTTTGCTAATGACGGTACCTGAAGAATA
AGCACCGGCTAACTACGTGCCAGCAGCCGCGGTAATACGTAGGGTGCAAGCGTTAATCGGAATTACTGGG
CGTAAAGCGTGCGCAGGCGGTTTTGTAAGTCTGATGTGAAGTCCCCGGGCTCAACCTGGGAATTGCATTG
GAGACTGCAAGGCTAGAATCTGGCAGAGGGGGGTAGAATTCCACGTGTAGCAGTGAAATGCGTAGATATG
TGGAGGAACACCGATGGCGAAGGCAGCCCCCTGGGTCAAGATTGACGCTCATGCACGAAAGCGTGGGGAG
CAAACAGGATTAGATACCCTGGTAGTCCACGCCCTAAACGATGTCTACTAGTTGTCGGGTCTTAATTGAC
TTGGTAACGCAGCTAACGCGTGAAGTAGACCGCCTGGGGAGTACGGTCGCAAGATTAAAACTCAAAGGAA
TTGACGGGGACCCGCACAAGCGGTGGATGATGTGGATTAATTCGATGCAACGCGAAAAACCTTACCTACC
CTTGACATGGCTGGAATCCTTGAGAGATCAGGGAGTGCTCGAAAGAGAACCAGTACACAGGTGCTGCATG
GCTGTCGTCAGCTCGTGTCGTGAGATGTTGGGTTAAGTCCCGCAACGAGCGCAACCCTTGTCATTAGTTG
CTACGAAAGGGCACTCTAATGAGACTGCCGGTGACAAACCGGAGGAAGGTGGGGATGACGTCAAGTCCTC
ATGGCCCTTATGGGTAGGGCTTCACACGTCATACAATGGTACATACAGAGCGCCGCCAACCCGCGAGGGG
GAGCTAATCGCAGAAAGTGTATCGTAGTCCGGATTGTAGTCTGCAACTCGACTGCATGAAGTTGGAATCG
CTAGTAATCGCGGATCAGCATGTCGCGGTGAATACGTTCCCGGGTCTTGTACACACCGCCCGTCACACCA
TGGGAGCGGGTTTTACCAGAAGTAGGTAGCTTAACCGCAAGGAGGGCGCTTACCACGGTAGGATTCGTGA
CTGGGGTGAAGTCGTAACAAGGTAACCGTAAAGGGCG
>219846773|ref|NR_026365.1|Janthinobacterium|Janthinobacterium lividum strain DSM 1522 16S ribosomal RNA, partial sequence
TTGAACGCTGGCGGCATGCCTTACACATGCAAGTCGAACGGCAGCACGGAGCTTGCTCTGGTGGCGAGTG
GCGAACGGGTGAGTAATATATCGGAACGTACCCTAGAGTGGGGGATAACGTAGCGAAAGTTACGCTAATA
CCGCATACGATCTAAGGATGAAAGTGGGGGATCGCAAGACCTCATGCTCGTGGAGCGGCCGATATCTGAT
TAGCTAGTTGGTAGGGTAAAAGCCTACCAAGGCATCGATCAGTAGCTGGTCTGAGAGGACGACCAGCCAC

ACTGGAACTGAGACACGGTCCAGACTCCTACGGGAGGCAGCAGTGGGGAATTTTGGACAATGGGCGAAAG
CCTGATCCAGCAATGCCGCGTGAGTGAAGAAGGCCTTCGGGTTGTAAAGCTCTTTTGTCAGGGAAGAAAC
GGTGAGAGCTAATATCTCTTGCTAATGACGGTACCTGAAGAATAAGCACCGGCTAACTACGTGCCAGCAG
CCGCGGTAATACGTAGGGTGCAAGCGTTAATCGGAATTACTGGGCGTAAAGCGTGCGCAGGCGGTTTTGT
AAGTCTGATGTGAAATCCCCGGGCTCAACCTGGGAATTGCATTGGAGACTGCAAGGCTAGAATCTGGCAG
AGGGGGGTAGAATTCCACGTGTAGCAGTGAAATGCGTAGATATGTGGAGGAACACCGATGGCGAAGGCAG
CCCCCTGGGTCAAGATTGACGCTCATGCACGAAAGCGTGGGGAGCAAACAGGATTAGATACCCTGGTAGT
CCACGCCCTAAACGATGTCTACTAGTTGTCGGGTCTTAATTGACTTGGTAACGCAGCTAACGCGTGAAGT
AGACCGCCTGGGGAGTACGGTCGCAAGATTAAAACTCAAAGGAATTGACGGGGACCCGCACAAGCGGTGG
ATGATGTGGATTAATTCGATGCAACGCGAAAAACCTTACCTACCCTTGACATGGCTGGAATCCCCGAGAG
ATTGGGGAGTGCTCGAAAGAGAACCAGTACACAGGTGCTGCATGGCTGTCGTCAGCTCGTGTCGTGAGAT
GTTGGGTTAAGTCCCGCAACGAGCGCAACCCTTGTCATTAGTTGCTACGAAAGGGCACTCTAATGAGACT
GCCGGTGACAAACCGGAGGAAGGTGGGGATGACGTCAAGTCCTCATGGCCCTTATGGGTAGGGCTTCACA
CGTCATACAATGGTACATACAGAGCGCCGCCAACCCGCGAGGGGGAGCTAATCGCAGAAAGTGTATCGTA
GTCCGGATTGTAGTCTGCAACTCGACTGCATGAAGTTGGAATCGCTAGTAATCGCGGATCAGCATGTCGC
GGTGAATACGTTCCCGGGTCTTGTACACACCGCCCGTCACACCATGGGAGCGGGTTTTACCAGAAGTAGG
TAGCTTAACCGCAAGGAGGGCGCTTACCACGGTAGGATTCGTGACTGGGGTGAAGTCGTAACAAGGTAG
>306411029|gb|HM234001.1|Delftia|Delftia sp. IHB B 4037 16S ribosomal RNA gene, partial sequence
AGAGTTTGATCTTGGCTCAGATTGAACGCTGGCGGCATGCCTTACACATGCAAGTCGAACGGTAACAGGT
CTTCGGACGCTGACGAGTGGCGAACGGGTGAGTAATACATCGGAACGTGCCCAGTCGTGGGGGATAACTA
CTCGAAAGAGTAGCTAATACCGCATACGATCTGAGGATGAAAGCGGGGGACCTTCGGGCCTCGCGCGATT
GGAGCGGCCGATGGCAGATTAGGTAGTTGGTGGGATAAAAGCTTACCAAGCCGACGATCTGTAGCTGGTC
TGAGAGGACGACCAGCCACACTGGGACTGAGACACGGCCCAGACTCCTACGGGAGGCAGCAGTGGGGAAT
TTTGGACAATGGGCGAAAGCCTGATCCAGCAATGCCGCGTGCAGGATGAAGGCCTTCGGGTTGTAAACTG
CTTTTGTACGGAACGAAAAAGCTTCTCCTAATACGAGAGGCCCATGACGGTACCGTAAGAATAAGCACCG
GCTAACTACGTGCCAGCAGCCGCGGTAATACGTAGGGTGCGAGCGTTAATCGGAATTACTGGGCGTAAAG
CGCGCGCAGGCGGTTATGTAAGACAGATGTGAAATCCCCGGGCTCAACCTGGGAACTGCATTTGTGACTG
CATGGCTAGAGTACGGTAGAGGGGGATGGAATTCCGCGTGTAGCAGTGAAATGCGTAGATATGCGGAGGA
ACACCGATGGCGAAGGCAATCCCCTGGACCTGTACTGACGCTCATGCACGAAAGCGTGGGGAGCAAACAG
GATTAGATACCCTGGTAGTCCACGCCCTAAACGATGTCAACTGGTTGTTGGGAATTAGTTTTCTCAGTAA
CGAAGCTAACGCGTGAAGTTGACCGCCTGGGGAGTACGGCCGCAAGGTTGAAACTCAAAGGAATTGACGG
GGACCCGCACAAGCGGTGGATGATGTGGTTTAATTCGATGCAACGCGAAAAACCTTACCCACCTTTGACA
TGGCAGGAAGTTTCCAGAGATGGATTCGTGCTCGAAAGAGAACCTGCACACAGGTGCTGCATGGCTGTCG
TCAGCTCGTGTCGTGAGATGTTGGGTTAAGTCCCGCAACGAGCGCAACCCTTGTCATTAGTTGCTACATT
TAGTTGGGCACTCTAATGAGACTGCCGGTGACAAACCGGAGGAAGGTGGGGATGACGTCAAGTCCTCATG
GCCCTTATAGGTGGGGCTACACACGTCATACAATGGCTGGTACAGAGGGTTGCCAACCCGCGAGGGGGAG
CTAATCCCATAAAACCAGTCGTAGTCCGGATCGCAGTCTGCAACTCGACTGCGTGAAGTCGGAATCGCTA
GTAATCGCGGATCAGCATGCCGCGGTGAATACGTTCCCGGGTCTTGTACACACCGCCCGTCACACCATGG
GAGCGGGTCTCGCCAGAAGTAGGTAGCCTAACCGCAAGGAGGGCGCTTACCACGGCGGGGTTCGTGACTG
GGGTGAAGTCGTAACAAGGTACCCGT
>66878813|gb|AY959107.1|Delftia|Uncultured bacterium clone rRNA334 16S ribosomal RNA gene, partial sequence
CCCTTAGAGTTTGATCCTGGCTCAGATTGAACGCTGGCGGCATGCCTTACACATGCAAGTCGAACGGTAA
CAGGTCTTCGGACGCTGACGAGTGGCGAACGGGTGAGTAATACATCGGAACGTGCCCAGTCGTGGGGGAT
AACTACTCGAAAGAGTAGCTAATACCGCATACGATCTGAGGATGAAAGCGGGGGACCTTCGGGCCTCGCG
CGATTGGAGCGGCCGATGGCAGATTAGGTAGTTGGTGGGATAAAAGCTTACCAAGCCGACGATCTGTAGC
TGGTCTGAGAGGACGACCAGCCACACTGGGACTGAGACACGGCCCAGACTCCTACGGGAGGCAGCAGTGG
GGAATTTTGGACAATGGGCGAAAGCCTGATCCAGCAATGCCGCGTGCAGGATGAAGGCCTTCGGGTTGTA
AACTGCTTTTGTACGGAACGAAAAAGCTTCTCCTAATACGAGAGGCCCATGACGGTACCGTAAGAATAAG
CACCGGCTAACTACGTGCCAGCAGCCGCGGTAATACGTAGGGTGCGAGCGTTAATCGGAATTACTGGGCG
TAAAGCGTGCGCAGGCGGTTATGTAAGACAGATGTGAAATCCCCGGGCTCAACCTGGGAACTGCATTTGT
GACTGCATGGCTAGAGTACGGTAGAGGGGGATGGAATTCCGCGTGTAGCAGTGAAATGCGTAGATATGCG
GAGGAACACCGATGGCGAAGGCAATCCCCTGGACCTGTACTGACGCTCATGCACGAAAGCGTGGGGAGCA

AACAGGATTAGATACCCTGGTAGTCCACGCCCTAAACGATGTCAACTGGTTGTTGGGAATTAGTTTTCTC
AGTAACGAAGCTAACGCGTGAAGTTGACCGCCTGGGGAGTACGGCCGCAAGGTTGAAACTCAAAGGAATT
GACGGGGACCCGCACAAGCGGTGGATGATGTGGTTTAATTCGATGCAACGCGAAAAACCTTACCCACCTT
TGACATGGCAGGAAGTTTCCAGAGATGGATTCGTGCTCGAAAGAGAACCTGCACACAGGTGCTGCATGGC
TGTCGTCAGCTCGTGTCGTGAGATGTTGGGTTAAGTCCCGCAACGAGCGCAACCCTTGTCATTAGTTGCT
ACATTTAGTTGGGCACTCTAATGAGACTGCCGGTGACAAACCGGAGGAAGGTGGGGATGACGTCAAGTCC
TCATGGCCCTTATAGGTGGGGCTACACACGTCATACAATGGCTGGTACAGAGGGTTGCCAACCCGCGAGG
GGGAGCTAATCCCATAAAACCAGTCGTAGTCCGGATCGCAGTCTGCAACTCGACTGCGTGAAGTCGGAAT
CGCTAGTAATCGCGGATCAGCATGCCGCGGTGAATACGTTCCCGGGTCTTGTACACACCGCCCGTCACAC
CATGGGAGCGGGTCTCGCCAGAAGTAGGTAGCCTAACCGCAAGGAGGGCGCTTACCACGGCGGGGTTCGT
GACTGGGGTGAAGTCGTAACAAGGTAGCCGTAAAGGGCGA
>265678855|ref|NR_029161.1|Comamonas|Comamonas testosteroni strain KS 0043 16S ribosomal RNA, complete sequence
CGAACTATAGAGTTTGATCCTGGCTCAGATTGAACGCTGGCGGCATGCTTTACACATGCAAGTCGAACGG
TAACAGGTCTTCGGATGCTGACGAGTGGCGAACGGGTGAGTAATACATCGGAACGTGCCTAGTAGTGGGG
GATAACTACTCGAAAGAGTAGCTAATACCGCATGAGATCTACGGATGAAAGCAGGGGACCTTCGGGCCTT
GTGCTACTAGAGCGGCTGATGGCAGATTAGGTAGTTGGTGGGGTAAAGGCTTACCAAGCCTGCGATCTGT
AGCTGGTCTGAGAGGACGACCAGCCACACTGGGACTGAGACACGGCCCAGACTCCTACGGGAGGCAGCAG
TGGGGAATTTTGGACAATGGGCGAAAGCCTGATCCAGCAATGCCGCGTGCAGGATGAAGGCCCTCGGGTT
GTAAACTGCTTTTGTACGGAACGAAAAGCCTGGGGCTAATATCCCCGGGTCATGACGGTACCGTAAGAAT
AAGCACCGGCTAACTACGTGCCAGCAGCCGCGGTAATACGTAGGGTGCAAGCGTTAATCGGAATTACTGG
GCGTAAAGCGTGCGCAGGCGGTTTTGTAAGACAGTGGTGAAATCCCCGGGCTCAACCTGGGAACTGCCAT
TGTGACTGCAAGGCTAGAGTGCGGCAGAGGGGGATGGAATTCCGCGTGTAGCAGTGAAATGCGTAGATAT
GCGGAGGAACACCGATGGCGAAGGCAATCCCCTGGGCCTGCACTGACGCTCATGCACGAAAGCGTGGGGA
GCAAACAGGATTAGATACCCTGGTAGTCCACGCCCTAAACGATGTCAACTGGTTGTTGGGTCTTAACTGA
CTCAGTAACGAAGCTAACGCGTGAAGTTGACCGCCTGGGGAGTACGGCCGCAAGGTTGAAACTCAAAGGA
ATTGACGGGGACCCGCACAAGCGGTGGATGATGTGGTTTAATTCGATGCAACGCGAAAAACCTTACCCAC
CTTTGACATGGCAGGAACTTACCAGAGATGGTTTGGTGCTCGAAAGAGAACCTGCACACAGGTGCTGCAT
GGCTGTCGTCAGCTCGTGTCGTGAGATGTTGGGTTAAGTCCCGCAACGAGCGCAACCCTTGCCATTAGTT
GCTACATTCAGTTGAGCACTCTAATGGGACTGCCGGTGACAAACCGGAGGAAGGTGGGGATGACGTCAAG
TCCTCATGGCCCTTATAGGTGGGGCTACACACGTCATACAATGGCTGGTACAAAGGGTTGCCAACCCGCG
AGGGGGAGCTAATCCCATAAAGCCAGTCGTAGTCCGGATCGCAGTCTGCAACTCGACTGCGTGAAGTCGG
AATCGCTAGTAATCGTGGATCAGAATGTCACGGTGAATACGTTCCCGGGTCTTGTACACACCGCCCGTCA
CACCATGGGAGCGGGTCTCGCCAGAAGTAGGTAGCCTAACCGTAAGGAGGGCGCTTACCACGGCGGGGTT
CGTGACTGGGGTGAAGTCGTAACAAGGTAGCCGTATCGGAAGGTGCGGCTGGATCACCTCCTTTCT
>66878699|gb|AY958993.1|Comamonas|Uncultured bacterium clone rRNA220 16S ribosomal RNA gene, partial sequence
CGCCCTTAGAGTTTGATCCTGGCTCAGATTGAACGCTGGCGGCATGCTTTACACATGCAAGTCGAACGGT
AACAGGTCTTCGGATGCTGACGAGTGGCGAACGGGTGAGTAATACATCGGAACGTGCCTAGTAGTGGGGG
ATAACTACTCGAAAGAGTAGCTAATACCGCATGAGATCTACGGATGAAAGCAGGGGACCTTCGGGCCTTG
TGCTACTAGAGCGGCTGATGGCAGATTAGGTAGTTGGTGGGGTAAAGGCTTACCAAGCCTGCGATCTGTA
GCTGGTCTGAGAGGACGACCAGCCACACTGGGACTGAGACACGGCCCAGACTCCTACGGGAGGCAGCAGT
GGGGAATTTTGGACAATGGGCGAAAGCCTGATCCAGCAATGCCGCGTGCAGGATGAAGGCCCTCGGGTTG
TAAACTGCTTTTGTACGGAACGAAAAGCCTGGGGCTAATATCCCCGGGTCATGACGGTACCGTAAGAATA
AGCACCGGCTAACTACGTGCCAGCAGCCGCGGTAATACGTAGGGTGCAAGCGTTAATCGGAATTACTGGG
CGTAAAGCGTGCGCAGGCGGTTTTGTAAGACAGTGGTGAAATCCCCGGGCTCAACCTGGGAACTGCCATT
GTGACTGCAAAGCTAGAGTGCGGCAGAGGGGGATGGAATTCCGCGTGTAGCAGTGAAATGCGTAGATATG
CGGAGGAACACCGATGGCGAAGGCAATCCCCTGGGCCTGCACTGACGCTCATGCACGAAAGCGTGGGGAG
CAAACAGGATTAGATACCCTGGTAGTCCACGCCCTAAACGATGTCAACTGGTTGTTGGGTCTTAACTGAC
TCAGTAACGAAGCTAACGCGTGAAGTTGACCGCCTGGGGAGTACGGCCGCAAGGTTGAAACTCAAAGGAA
TTGACGGGGACCCGCACAAGCGGTGGATGATGTGGTTTAATTCGATGCAACGCGAAAAACCTTACCCACC
TTTGACATGGCAGGAACTTACCAGAGATGGTTTGGTGCTCGAAAGAGAACCTGCACACAGGTGCTGCATG
GCTGTCGTCAGCTCGTGTCGTGAGATGTTGGGTTAAGTCCCGCAACGAGCGCAACCCTTGCCATTAGTTG
CTACATTCAGTTGAGCACTCTAATGGGACTGCCGGTGACAAACCGGAGGAAGGTGGGGATGACGTCAAGT

CCTCATGGCCCTTATAGGTGGGGCTACACACGTCATACAATGGCTGGTACAAAGGGTTGCCAACCCGCGA
GGGGGAGCTAATCCCATAAAGCCAGTCGTAGCCCGGATCGCAGTCTGCAACTCGACTGCGTGAAGTCGGA
ATCGCTAGTAATCGTGGATCAGAATGTCACGGTGAATACGTTCCCGGGTCTTGTACACACCGCCCGTCAC
ACCATGGGAGCGGGTCTCGCCAGAAGTAGGTAGCCTAACCGTAAGGAGGGCGCTTACCACGGCGGGGTTC
GTGACTGGGGTGAAGTCGTAACAAGGTAGCCGTAAAGGG
>269784593|dbj|AB503703.1|Ralstonia|Ralstonia sp. RS2 gene for 16S rRNA, partial sequence
AGAGTTTGATCCTGGCTCAGATTGAACGCTGGCGGCATGCCTTACACATGCAAGTCGAACGGCAGCATGA
TCTAGCTTGCTAGATTGATGGCGAGTGGCGAACGGGTGAGTAATACATCGGAACGTGCCCTGTAGTGGGG
GATAACTAGTCGAAAGATTAGCTAATACCGCATACGACCTGAGGGTGAAAGTGGGGGACCGCAAGGCCTC
ATGCTATAGGAGCGGCCGATGTCTGATTAGCTAGTTGGTGGGGTAAAGGCCCACCAAGGCGACGATCAGT
AGCTGGTCTGAGAGGACGATCAGCCACACTGGGACTGAGACACGGCCCAGACTCCTACGGGAGGCAGCAG
TGGGGAATTTTGGACAATGGGCGAAAGCCTGATCCAGCAATGCCGCGTGTGTGAAGAAGGCCTTCGGGTT
GTAAAGCACTTTTGTCCGGAAAGAAATGGCTCTGGTTAATACCTGGGGTCGATGACGGTACCGGAAGAAT
AAGGACCGGCTAACTACGTGCCAGCAGCCGCGGTAATACGTAGGGTCCAAGCGTTAATCGGAATTACTGG
GCGTAAAGCGTGCGCAGGCGGTTGTGCAAGACCGATGTGAAATCCCCGAGCTTAACTTGGGAATTGCATT
GGTGACTGCACGGCTAGAGTGTGTCAGAGGGGGGTAGAATTCCACGTGTAGCAGTGAAATGCGTAGAGAT
GTGGAGGAATACCGATGGCGAAGGCAGCCCCCTGGGATAACACTGACGCTCATGCACGAAAGCGTGGGGA
GCAAACAGGATTAGATACCCTGGTAGTCCACGCCCTAAACGATGTCAACTAGTTGTTGGGGATTCATTTC
CTTAGTAACGTAGCTAACGCGTGAAGTTGACCGCCTGGGGAGTACGGTCGCAAGATTAAAACTCAAAGGA
ATTGACGGGGACCCGCACAAGCGGTGGATGATGTGGATTAATTCGATGCAACGCGAAAAACCTTACCTAC
CCTTGACATGCCACTAACGAAGCAGAGATGCATTAGGTGCTCGAAAGAGAAAGTGGACACAGGTGCTGCA
TGGCTGTCGTCAGCTCGTGTCGTGAGATGTTGGGTTAAGTCCCGCAACGAGCGCAACCCTTGTCTCTAGT
TGCTACGAAAGGGCACTCTAGAGAGACTGCCGGTGACAAACCGGAGGAAGGTGGGGATGACGTCAAGTCC
TCATGGCCCTTATGGGTAGGGCTTCACACGTCATACAATGGTGCATACAGAGGGTTGCCAAGCCGCGAGG
TGGAGCTAATCCCAGAAAATGCATCGTAGTCCGGATCGTAGTCTGCAACTCGACTACGTGAAGCTGGAAT
CGCTAGTAATCGCGGATCAGCATGCCGCGGTGAATACGTTCCCGGGTCTTGTACACACCGCCCGTCACAC
CATGGGAGTGGGCTTTACCAGAAGTAGTTAGCCTAACCGCAAGGAGGGCGATTACCACGGTAGGGTTCAT
GACTGGGGTGAAGTCGTAACAAGGTAGCCGTATCGGAAGGTGCGGCTGGATCACCTCCTT
>239923955|gb|GQ179677.1|Ralstonia|Uncultured Ralstonia sp. clone VE6A10 16S ribosomal RNA gene, partial sequence
ATTGAACGCTGGCGGCATGCCTTACACATGCAAGTCGAACGGCAGCATGATCTAGCTTGCTAGATTGATG
GCGAGTGGCGAACGGGTGAGTAATACATCGGAACGTGCCCTGTAGTGGGGGATAACTAGTCGAAAGATTA
GCTAATACCGCATACGACCTGAGGGTGAAAGTGGGGGACCGCAAGGCCTCATGCTATAGGAGCGGCCGAT
GTCTGATTAGCTAGTTGGTGGGGTAAAGGCCCACCAAGGCGACGATCAGTAGCTGGTCTGAGAGGACGAT
CAGCCACACTGGGACTGAGACACGGCCCAGACTCCTACGGGAGGCAGCAGTGGGGAATTTTGGACAATGG
GCGAAAGCCTGATCCAGCAATGCCGCGTGTGTGAAGAAGGCCTTCGGGTTGTAAAGCACTTTTGTCCGGA
AAGAAATGGCTCTGGTTAATACCTGGGGTCGATGACGGTACCGGAAGAATAAGGACCGGCTAACTACGTG
CCAGCAGCCGCGGTAATACGTAGGGTCCAAGCGTTAATCGGAATTACTGGGCGTAAAGCGTGCGCAGGCG
GTTGTGCAAGACCGATGTGAAATCCCCGAGCTTAACTTGGGAATTGCATTGGTGACTGCACGGCTAGAGT
GTGTCAGAGGGGGGTAGAATTCCACGTGTAGCAGTGAAATGCGTAGAGATGTGGAGGAATACCGATGGCG
AAGGCAGCCCCCTGGGATAACACTGACGCTCATGCACGAAAGCGTGGGGAGCAAACAGGATTAGATACCC
TGGTAGTCCACGCCCTAAACGATGTCAACTAGTTGTTGGGGATTCATTTCCTTAGTAACGTAGCTAACGC
GTGAAGTTGACCGCCTGGGGAGTACGGTCGCAAGATTAAAACTCAAAGGAATTGACGGGGACCCGCACAA
GCGGTGGATGATGTGGATTAATTCGATGCAACGCGAAAAACCTTACCTACCCTTGACATGCCACTAACGA
AGCAGAGATGCATTAGGTGCTCGAAAGAGAAAGTGGACACAGGTGCTGCATGGCTGTCGTCAGCTCGTGT
CGTGAGATGTTGGGTTAAGTCCCGCAACGAGCGCAACCCTTGTCTCTAGTTGCTACGAAAGGGCACTCTA
GAGAGACTGCCGGTGACAAACCGGAGGAAGGTGGGGATGACGTCAAGTCCTCATGGCCCTTATGGGTAGG
GCTTCACACGTCATACAATGGTGCATACAGAGGGTTGCCAAGCCGCGAGGTGGAGCTAATCCCAGAAAAT
GCATCGTAGTCCGGATCGTAGTCTGCAACTCGACTACGTGAAGCTGGAATCGCTAGTAATCGCGGATCAG
CATGCCGCGGTGAATACGTTCCCGGGTCTTGTACACACCGCCCGTCACACCATGGGAGTGGGCTTTACCA
GAAGTAGTTAGCCTAACCGCAAGGAGGGCGATTACCACGGTAGGGTTCATGACTGGGGTGAAGTCGTAAC
AAGGTA

>239923961|gb|GQ179683.1|Aeromonas|Uncultured Aeromonas sp. clone VE39G03 16S ribosomal RNA gene, partial sequence
ATTGAACGCTGGCGGCAGGCCTAACACATGCAAGTCGAGCGGCAGCGGGAAAGTAGCTTGCTACTTTTGC
CGGCGAGCGGCGGACGGGTGAGTAATGCCTGGGGATCTGCCCAGTCGAGGGGGATAACAGTTGGAAACGA
CTGCTAATACCGCATACGCCCTACGGGGGAAAGGAGGGGACCTTCGGGCCTTTCGCGATTGGATGAACCC
AGGTGGGATTAGCTAGTTGGTGGGGTAATGGCTCACCAAGGCGACGATCCCTAGCTGGTCTGAGAGGATG
ATCAGCCACACTGGAACTGAGACACGGTCCAGACTCCTACGGGAGGCAGCAGTGGGGAATATTGCACAAT
GGGGGAAACCCTGATGCAGCCATGCCGCGTGTGTGAAGAAGGCCTTCGGGTTGTAAAGCACTTTCAGCGA
GGAGGAAAGGTTGGCGCCTAATACGTGTCAACTGTGACGTTACTCGCAGAAGAAGCACCGGCTAACTCCG
TGCCAGCAGCCGCGGTAATACGGAGGGTGCAAGCGTTAATCGGAATTACTGGGCGTAAAGCACACGCAGG
CGGTTGGATAAGTTAGATGTGAAAGCCCCGGGCTCAACCTGGGAATTGCATTTAAAACTGTCCAGCTAGA
GTCTTGTAGAGGGGGGTAGAATTCCAGGTGTAGCGGTGAAATGCGTAGAGATCTGGAGGAATACCGGTGG
CGAAGGCGGCCCCCTGGACAAAGACTGACGCTCAGGTGCGAAAGCGTGGGGAGCAAACAGGATTAGATAC
CCTGGTAGTCCACGCCGTAAACGATGTCGATTTGGAGGCTGTGTCCTTGAGATGTGGCTTCCGGAGCTAA
CGCGTTAAATCGACCGCCTGGGGAGTACGGCCGCAAGGTTAAAACTCAAATGAATTGACGGGGGCCCGCA
CAAGCGGTGGAGCATGTGGTTTAATTCGATGCAACGCGAAGAACCTTACCTGGCCTTGACATGTCTGGAA
TCCTGTAGAGATGCGGGAGTGCCTTCGGGAATCAGAACACAGGTGCTGCATGGCTGTCGTCAGCTCGTGT
CGTGAGATGTTGGGTTAAGTCCCGCAACGAGCGCAACCCCTGTCCTTTGTTGCCAGCACGTAATGGTGGG
AACTCAAGGGAGACTGCCGGTGATAAACCGGAGGAAGGTGGGGATGACGTCAAGTCATCATGGCCCTTAC
GGCCAGGGCTACACACGTGCTACAATGGCGCGTACAGAGGGCTGCAAGCTAGCGATAGTGAGCGAATCCC
AAAAAGCGCGTCGTAGTCCGGATCGGAGTCTGCAACTCGACTCCGTGAAGTCGGAATCGCTAGTAATCGC
GAATCAGAATGTCGCGGTGAATACGTTCCCGGGCCTTGTACACACCGCCCGTCACACCATGGGAGTGGGT
TGCACCAGAAGTAGATAGCTTAACCTTCGGGAGGGCGTTTACCACGGTGTGATTCATGACTGGGGTGAAG
TCGTAACAAGGTA
>62953206|emb|AJ967026.1|Shewanella|Shewanella sp. LMG 23023 16S rRNA gene, strain LMG 23023
ATTGAACGCTGGCGGCAGGCCTAACACATGCAAGTCGAGCGGCAGCGGGAAGATAGCTTGCTATCTTTGC
CGGCRAGCGGCGGACGGGTGAGTAATGCCTAGGGATCTGCCCAGTCGAGGGGGATAACAGTTGGAAACGA
CTGCTAATACCGCATACGCCCTACGGGGGAAAGGAGGGGACCTTCGGGCCTTCCGCGATTGGATGAACCT
AGGTGGGATTAGCTAGTTGGTGAGGTAATGGCTCACCAAGGCGACGATCCCTAGCTGTTCTGAGAGGATG
ATCAGCCACACTGGGACTGAGACACGGCCCAGACTCCTACGGGAGGCAGCAGTGGGGAATATTGCACAAT
GGGGGAAACCCTGATGCAGCCATGCCGCGTGTGTGAAGAAGGCCTTCGGGTTGTAAAGCACTTTCAGTAG
GGAGGAAAGGTAGCAGCTTAATACGCTGTTGCTGTGACGTTACCTACAGAAGAAGGACCGGCTAACTCCG
TGCCAGCAGCCGCGGTAATACGGAGGGTCCGAGCGTTAATCGGAATTACTGGGCGTAAAGCGTGCGCAGG
CGGTTTGTTAAGCGAGATGTGAAAGCCCCGGGCTCAACCTGGGAATTGCATTTCGAACTGGCGAACTAGA
GTCTTGTAGAGGGGGGTAGAATTCCAGGTGTAGCGGTGAAATGCGTAGAGATCTGGAGGAATACCGGTGG
CGAAGGCGGCCCCCTGGACAAAGACTGACGCTCAGGCACGAAAGCGTGGGGAGCAAACAGGATTAGATAC
CCTGGTAGTCCACGCCGTAAACGATGTCTACTCGGAGTTTGGTGTCTTGAACACTGGGCTCTCAAGCTAA
CGCATTAAGTAGACCGCCTGGGGAGTACGGCCGCAAGGTTAAAACTCAAATGAATTGACGGGGGCCCGCA
CAAGCGGTGGAGCATGTGGTTTAATTCGATGCAACGCGAAGAACCTTACCTACTCTTGACATCCASRGAA
TTCGCTAGAGATAGCTTAGTGCCTTCGGGAACYCTGAGACAGGTGCTGCATGGCTGTCGTCAGCTCGTGT
TGTGAAATGTTGGGTTAAGTCCCGCAACGAGCGCAACCCCTATCCTTATTTGCCAGCACGTAATGGTGGG
AACTCTAGGGAGACTGCCGGTGATAAACCGGAGGAAGGTGGGGACGACGTCAAGTCATCATGGCCCTTAC
GAGTAGGGCTACACACGTGCTACAATGGCGAGTACAGAGGGTTGCAAAGCCGCGAGGTGGAGCTAATCTC
ACAAAGCTCGTCGTAGTCCGGATTGGAGTCTGCAACTCGACTCCATGAAGTCGGAATCGCTAGTAATCGT
GGATCAGAATGCCACGGTGAATACGTTCCCGGGCCTTGTACACACCGCCCGTCACACCATGGGAGTGGGC
TGCAAAAGAAGTGGGTAGCTTAACCTTCGGGGGGGCGCTCACCACTTTGTGGTTCATGACTGGGGTGAAG
TCGTAACAAGGTAGCCCTAGGGGAACC
>66878789|gb|AY959083.1|Shewanella|Uncultured bacterium clone rRNA310 16S ribosomal RNA gene, partial sequence
TTGAACGCTGGCGGCAGGCCTAACACATGCAAGTCGAGCGGCAGCGGGAAGATAGCTTGCTATCTTTGCC
GGCGAGCGGCGGACGGGTGAGTAATGCCTAGGGATCTGCCCAGTCGAGGGGGATAACAGTTGGAAACGAC
TGCTAATACCGCATACGCCCTACGGGGGAAAGGAGGGGACCTTCGGGCCTTCCGCGATTGGATGAACCTA
GGTGGGATTAGCTAGTTGGTGAGGTAATGGCTCACCAAGGCGACGATCCCTAGCTGTTCTGAGAGGATGA

```
TCAGCCACACTGGGACTGAGACACGGCCCAGACTCCTACGGGAGGCAGCAGTGGGGAATATTGCACAATG
GGGGAAACCCTGATGCAGCCATGCCGCGTGTGTGAAGAAGGCCTTCGGGTTGTAAAGCACTTTCAGTAGG
GAGGAAAGGTAGCAGCTTAATACGCTGTTGCTGTGACGTTACCTACAGAAGAAGGACCGGCTAACTCCGT
GCCAGCAGCCGCGGTAATACGGAGGGTCCGAGCGTTAATCGGAATTACTGGGCGTAAAGCGTGCGCAGGC
GGTTTGTTAAGCGAGATGTGAAAGCCCCGGGCTCAACCTGGGAATTGCATTTCGAACTGGCGAACTAGAG
TCTTGTAGAGGGGGGTAGAATTCCAGGTGTAGCGGTGAAATGCGTAGAGATCTGGAGGAATACCGGTGGC
GAAGGCGGCCCCCTGGACAAAGACTGACGCTCAGGCACGAAAGCGTGGGGAGCAAACAGGATTAGATACC
CTGGTAGTCCACGCCGTAAACGATGTCTACTCGGAGTTTGGTGTCTTGAACACTGGGGCTCTCAAGCTAA
CGCATTAAGTAGACCGCCTGGGGAGTACGGCCGCAAGGTTAAAACTCAAATGAATTGACGGGGGCCCGCA
CAAGCGGTGGAGCATGTGGTTTAATTCGATGCAACGCGAAGAACCTTACCTACTCTTGACATCCACGGAA
TTCGCTAGAGATAGCTTAGTGCCTTCGGGAACCGTGAGACAGGTGCTGCATGGCTGTCGTCAGCTCGTGT
TGTGAAATGTTGGGTTAAGTCCCGCAACGAGCGCAACCCCTATCCTTATTTGCCAGCACGTAATGGTGGG
AACTCTAGGGAGACTGCCGGTGATAAACCGGAGGAAGGTGGGGACGACGTCAAGTCATCATGGCCCTTAC
GAGTAGGGCTACACACGTGCTACAATGGCGAGTACAGAGGGTTGCAAAGCCGCGAGGTGGAGCTAATCTC
ACAAAGCTCGTCGTAGTCCGGATTGGAGTCTGCAACTCGACTCCATGAAGTCGGAATCGCTAGTAATCGT
GGATCAGAATGCCACGGTGAATACGTTCCCGGGCCTTGTACACACCGCCCGTCACACCATGGGAGTGGGC
TGCAAAAGAAGTGGGTAGCTTAACCTTCGGGGGGGCGCTCACCACTTTGTGGTTCATGACTGGGGTGAA
>239923982|gb|GQ179704.1|Enterobacter|Uncultured Enterobacter sp. clone
VE45H12 16S ribosomal RNA gene, partial sequence
ATTGAACGCTGGCGGCAGGCCTAACACATGCAAGTCGAGCGGTAACAGGGAGTAGCTTGCTACTCCGCTG
ACGAGCGGCGGACGGGTGAGTAATGTCTGGGGATCTGCCTGATGGAGGGGGATAACTACTGGAAACGGTA
GCTAATACCGCATAACGTCGCAAGACCAAAGAGGGGGACCTTCGGGCCTCTTGCCATCGGATGAACCCAG
ATGGGATTAGCTAGTAGGTGGGGTAATGGCTCACCTAGGCGACGATCCCTAGCTGGTCTGAGAGGATGAC
CAGCCACACTGGAACTGAGACACGGTCCAGACTCCTACGGGAGGCAGCAGTGGGGAATATTGCACAATGG
GCGCAAGCCTGATGCAGCCATGCCGCGTGTGTGAAGAAGGCCTTCGGGTTGTAAAGCACTTTCAGCGAGG
AGGAAGGCATTAAGGTTAATAACCTTGGTGATTGACGTTACTCGCAGAAGAAGCACCGGCTAACTCCGTG
CCAGCAGCCGCGGTAATACGGAGGGTGCAAGCGTTAATCGGAATTACTGGGCGTAAAGCGCACGCAGGCG
GTTTGTTAAGTCGGATGTGAAATCCCCGGGCTCAACCTGGGAACTGCATTCGAAACTGGCAAGCTTGAGT
CTTGTAGAGGGGGGTAGAATTCCAGGTGTAGCGGTGAAATGCGTAGAGATCTGGAGGAATACCGGTGGCG
AAGGCGGCCCCCTGGACAAAGACTGACGCTCAGGTGCGAAAGCGTGGGGAGCAAACAGGATTAGATACCC
TGGTAGTCCACGCCGTAAACGATGTCGACTTGGAGGTTGTGCCCTTGAGGCGTGGCTTCCGGAGCTAACG
CGTTAAGTCGACCGCCTGGGGAGTACGGCCGCAAGGTTAAAACTCAAATGAATTGACGGGGGCCCGCACA
AGCGGTGGAGCATGTGGTTTAATTCGATGCAACGCGAAGAACCTTACCTACTCTTGACATCCAGAGAACT
TTCCAGAGATGGATTGGTGCCTTCGGGAACTCTGAGACAGGTGCTGCATGGCTGTCGTCAGCTCGTGTTG
TGAAATGTTGGGTTAAGTCCCGCAACGAGCGCAACCCTTATCCTTTGTTGCCAGCGGTTCGGCCGGGAAC
TCAAAGGAGACTGCCAGTGATAAACTGGAGGAAGGTGGGGATGACGTCAAGTCATCATGGCCCTTACGAG
TAGGGCTACACACGTGCTACAATGGCGCATACAAAGAGAAGTGACCTCGCGAGAGCAAGCGGACCTCATA
AAGTGCGTCGTAGTCCGGATTGGAGTCTGCAACTCGACTCCATGAAGTCGGAATCGCTAGTAATCGTAGA
TCAGAATGCTACGGTGAATACGTTCCCGGGCCTTGTACACACCGCCCGTCACACCATGGGAGTGGGTTGC
AAAAGAAGTAGGTAGCTTAACCTTCGGGAGGGCGCTTACCACTTTGTGATTCATGACTGGGGTGAAGTCG
TAACAAGGTA
>66878681|gb|AY958975.1|Enterobacter|Uncultured bacterium clone rRNA202
16S ribosomal RNA gene, partial sequence
TTGAACGCTGGCGGCAGGCCTAACACATGCAAGTCGAGCGGTAACAGGGAGTAGCTTGCTACTCCGCTGA
CGAGCGGCGGACGGGTGAGTAATGTCTGGGGATCTGCCTGATGGAGGGGGATAACTACTGGAAACGGTAG
CTAATACCGCATAACGTCGCAAGACCAAAGAGGGGGACCTTCGGGCCTCTTGCCATCGGATGAACCCAGA
TGGGATTAGCTAGTAGGTGGGGTAATGGCTCACCTAGGCGACGATCCCTAGCTGGTCTGAGAGGATGACC
AGCCACACTGGAACTGAGACACGGTCCAGACTCCTACGGGAGGCAGCAGTGGGGAATATTGCACAATGGG
CGCAAGCCTGATGCAGCCATGCCGCGTGTGTGAAGAAGGCCTTCGGGTTGTAAAGCACTTTCAGCGAGGA
GGAAGGCATTAAGGTTAATAACCTTGGTGATTGACGTTACTCGCAGAAGAAGCACCGGCTAACTCCGTGC
CAGCAGCCGCGGTAATACGGAGGGTGCAAGCGTTAATCGGAATTACTGGGCGTAAAGCGCACGCAGGCGG
TTTGTTAAGTCGGATGTGAAATCCCCGGGCTCAACCTGGGAACTGCATTCGAAACTGGCAAGCTTGAGTC
TTGTAGAGGGGGGTAGAATTCCAGGTGTAGCGGTGAAATGCGTAGAGATCTGGAGGAATACCGGTGGCGA
AGGCGGCCCCCTGGACAAAGACTGACGCTCAGGTGCGAAAGCGTGGGGAGCAAACAGGATTAGATACCCT
```

GGTAGTCCACGCCGTAAACGATGTCGACTTGGAGGTTGTGCCCTTGAGGCGTGGCTTCCGGAGCTAACGC
GTTAAGTCGACCGCCTGGGGAGTACGGCCGCAAGGTTAAAACTCAAATGAATTGACGGGGGCCCGCACAA
GCGGTGGAGCATGTGGTTTAATTCGATGCAACGCGAAGAACCTTACCTACTCTTGACATCCAGAGAACTT
TCCAGAGATGGATTGGTGCCTTCGGGAACTCTGAGACAGGTGCTGCATGGCTGTCGTCAGCTCGTGTTGT
GAAATGTTGGGTTAAGTCCCGCAACGAGCGCAACCCTTATCCTTTGTTGCCAGCGGTTCGGCCGGGAACT
CAAAGGAGACTGCCAGTGATAAACTGGAGGAAGGTGGGGATGACGTCAAGTCATCATGGCCCTTACGAGT
AGGGCTACACACGTGCTACAATGGCGCATACAAAGAGAAGCGACCTCGCGAGAGCAAGCGGACCTCATAA
AGTGCGTCGTAGTCCGGATTGGAGTCTGCAACTCGACTCCATGAAGTCGGAATCGCTAGTAATCGTAGAT
CAGAATGCTACGGTGAATACGTTCCCGGGCCTTGTACACACCGCCCGTCACACCATGGGAGTGGGTTGCA
AAAGAAGTAGGTAGCTTAACCTTCGGGAGGGCGCTTACCACTTTGTGATTCGTGACTGGGGTGAAGTCGT
AACAAGGTAACCGTAAAGGGC
>194399042|gb|EU855202.1|Enterobacter|Enterobacter sp. CTSP21 16S ribosomal RNA gene, partial sequence
AGAGTTTGATCCTGGCTCAGATTGAACGCTGGCGGCAGGCCTAACACATGCAAGTCGAGCGGTAGCACAG
AGAGCTTGCTCTCGGGTGACGAGCGGCGGACGGGTGAGTAATGTCTGGGAAACTGCCTGATGGAGGGGGA
TAACTACTGGAAACGGTAGCTAATACCGCATAACGTCGCAAGACCAAAGAGGGGGACCTTCGGGCCTCTT
GCCATCAGATGTGCCCAGATGGGATTAGCTAGTAGGTGGGGTAACGGCTCACCTAGGCGACGATCCCTAG
CTGGTCTGAGAGGATGACCAGCCACACTGGAACTGAGACACGGTCCAGACTCCTACGGGAGGCAGCAGTG
GGGAATATTGCACAATGGGCGCAAGCCTGATGCAGCCATGCCGCGTGTATGAAGAAGGCCTTCGGGTTGT
AAAGTACTTTCAGCGGGGAGGAAGGCGATAAGGTTAATAACCTTGTCGATTGACGTTACCCGCAGAAGAA
GCACCGGCTAACTCCGTGCCAGCAGCCGCGGTAATACGGAGGGTGCAAGCGTTAATCGGAATTACTGGGC
GTAAAGCGCACGCAGGCGGTCTGTCAAGTCGGATGTGAAATCCCCGGGCTCAACCTGGGAACTGCATTCG
AAACTGGCAGGCTAGAGTCTTGTAGAGGGGGGTAGAATTCCAGGTGTAGCGGTGAAATGCGTAGAGATCT
GGAGGAATACCGGTGGCGAAGGCGGCCCCCTGGACAAAGACTGACGCTCAGGTGCGAAAGCGTGGGGAGC
AAACAGGATTAGATACCCTGGTAGTCCACGCCGTAAACGATGTCGACTTGGAGGTTGTGCCCTTGAGGCG
TGGCTTCCGGAGCTAACGCGTTAAGTCGACCGCCTGGGGAGTACGGCCGCAAGGTTAAAACTCAAATGAA
TTGACGGGGGCCCGCACAAGCGGTGGAGCATGTGGTTTAATTCGATGCAACGCGAAGAACCTTACCTACT
CTTGACATCCAGAGAACTTTCCAGAGATGGATTGGTGCCTTCGGGAACTCTGAGACAGGTGCTGCATGGC
TGTCGTCAGCTCGTGTTGTGAAATGTTGGGTTAAGTCCCGCAACGAGCGCAACCCTTATCCTTTGTTGCC
AGCGGTTCGGCCGGGAACTCAAAGGAGACTGCCAGTGATAAACTGGAGGAAGGTGGGGATGACGTCAAGT
CATCATGGCCCTTACGAGTAGGGCTACACACGTGCTACAATGGCGCATACAAAGAGAAGCGACCTCGCGA
GAGCAAGCGGACCTCATAAAGTGCGTCGTAGTCCGGATTGGAGTCTGCAACTCGACTCCATGAAGTCGGA
ATCGCTAGTAATCGTAGATCAGAATGCTACGGTGAATACGTTCCCGGGCCTTGTACACACCGCCCGTCAC
ACCATGGGAGTGGGTTGCAAAAGAAGTAGGTAGCTTAACCTTCGGGAGGGCGCTTACCACTTTGTGATTC
ATGACTGGGGTGAAGTCGTAACAAGGTAGCC
>66878543|gb|AY958837.1|Escherichia/Shigella|Uncultured bacterium clone rRNA064 16S ribosomal RNA gene, partial sequence
AGAGTTTGATCCTGGCTCAGATTGAACGCTGGCGGCAGGCCTAACACATGCAAGTCGAACGGTAACAGGA
AACAGCTTGCTGTTTCGCTGACGAGTGGCGGACGGGTGAGTAATGTCTGGGAAACTGCCTGATGGAGGGG
GATAACTACTGGAAACGGTAGCTAATACCGCATAACGTCGCAAGACCAAAGAGGGGGACCTTCGGGCCTC
TTGCCATCGGATGTGCCCAGATGGGATTAGCTTGTTGGTGGGGTAACGGCTCACCAAGGCGACGATCCCT
AGCTGGTCTGAGAGGATGACCAGCCACACTGGAACTGAGACACGGTCCAGACTCCTACGGGAGGCAGCAG
TGGGGAATATTGCACAATGGGCGCAAGCCTGATGCAGCCATGCCGCGTGTATGAAGAAGGCCCTCGGGTT
GTAAAGTACTTTCAGCGGGGAGGAAGGGAGTAAAGTTAATACCTTTGCTCATTGACGTTACCCGCAGAAG
AAGCACCGGCTAACTCCGTGCCAGCAGCCGCGGTGATACGGAGGGTGCAAGCGTTAATCGGAATTACTGG
GCGTAAAGCGCACGTAGGCGGTTTGTTAAGTCAGATGTGAAATCCCCGGGCTCAACCTGGGAACTGCATC
TGATACTGGCAAGCTTGAGTCTCGTAGAGGGGGGTAGAATTCTAGGTGTAGCGGTGAAATGCGTAGAGAT
CTGGAGGAATACCGGTGGCGAAGGCGGCCCCCTGGACGAAGACTGACGCTCAGGTGCGAAAGCGTGGGGA
GCAAACAGGATTAGATACCCTGGTAGTCCACGCCGTAAACGATGTCGACTTGGAGGTTGTGCCCTTGAGG
CGTGGCTTCCGGAGCTAACGCGTTAAGTCGACCGCCTGGGGAGTACGGCCGCAAGGTTAAAACTCAAATG
AATTGACGGGGGCCCGCACAAGCGGTGGAGCATGTGGTTTAATTCGATGCAACGCGAAGAACCTTACCTG
GTCTTGACATCCACGGAAGTTTTCAGAGATGAGAATGTGCCTTCGGGAACCGTGAGACAGGTGCTGCATG
GCTGTCGTCAGCTCGTGTTGTGGAATGTTGGGTTAAGTCCCGCAACGAGCGCAACCCTTATCCTTTGTTG
CCAGCGGTCCGGCCGGGAACTCAAAGGAGACTGCCAGTGATAAACTGGAGGAAGGTGGGGATGACGTCAA

GTCATCATGGCCCTTACGACCAGGGCTACACACGTGCTACAATGGCGCATACAAAGAGAGGCGACCTCGC
GAGAGCAAGCGGACCTCATAAAGTGCGTCGTAGTCCGGATTGGAGTCTGCAACTCGACTCCATGAAGTCG
GAATCGCTAGTAATCGTGGATCAGAATGCCACGGTGAATACGTTCCCGGGCCTTGTACACACCGCCCGTC
ACACCATGGGAGTGGGTTGCAAAAGAAGTAGGTAGCTTAACCTCCGGGAGGGCGCTTACCACTTTGTGAT
TCATGACTGGGGTGAA
>295983632|gb|HM021350.1|Escherichia/Shigella|Uncultured bacterium clone 13L-19 16S ribosomal RNA gene, partial sequence
CTTGCGGCAGGCCTACACATGCAAGTCGAACGGTAACAGGAAACAGCTTGCTGTTTCGCTGACGAGTGGC
GGACGGGTGAGTAATGTCTGGGAAACTGCCTGATGGAGGGGGATAACTACTGGAAACGGTAGCTAATACC
GCATAACGTCGCAAGACCAAAGAGGGGGACCTTCGGGCCTCTTGCCATCGGATGTGCCCAGATGGGATTA
GCTAGTAGGTGGGGTAACGGCTCACCTAGGCGACGATCCCTAGCTGGTCTGAGAGGATGACCAGCCACAC
TGGAACTGAGACACGGTCCAGACTCCTACGGGAGGCAGCAGTGGGGAATATTGCACAATGGGCGCAAGCC
TGATGCAGCCATGCCGCGTGTATGAAGAAGGCCTTCGGGTTGTAAAGTACTTTCAGCGGGGAGGAAGGGA
GTAAAGTTAATACCTTTACTCATTGACGTTACCCGCAGAAGAAGCACCGGCTAACTCCGTGCCAGCAGCC
GCGGTAATACGGAGGGTGCAAGCGTTAATCGGAATTACTGGGCGTAAAGCGCACGCAGGCGGTTTGTTAA
GTCAGATGTGAAATCCCCGGGCTCAACCTGGGAACTGCATCTGATACTGGCAAGCTTGAGTCTCGTAGAG
GGGGGTAGAATTCCAGGTGTAGCGGTGAAATGCGTAGAGATCTGGAGGAATACCGGTGGCGAAGGCGGCC
CCCTGGACGAAGACTGACGCTCAGGTGCGAAAGCGTGGGGAGCAAACAGGATTAGATACCCTGGTAGTCC
ACGCCGTAAACGATGTCGACTTGGAGGTTGTGCCCTTGAGGCGTGGCTTCCGGAGCTAACGCGTTAAGTC
GACCGCCTGGGGAGTACGGCCGCAAGGTTAAAACTCAAATGAATTGACGGGGGCCCGCACAAGCGGTGGA
GCATGTGGTTTAATTCGATGCAACGCGAAGAACCTTACCTGGTCTTGACATCCACGGAAGTTTTCAGAGA
TGAGAATGTGCCTTCGGGAACCGTGAGACAGGTGCTGCATGGCTGTCGTCAGCTCGTGTTGTGAAATGTT
GGGTTAAGTCCCGCAACGAGCGCAACCCTTATCCTTTGTTGCCAGCGGTCCGGCCGGGAACTCAAAGGAG
ACTGCCAGTGATAAACTGGAGGAAGGTGGGGATGACGTCAAGTCATCATGGCCCTTACGACCAGGGCTAC
ACACGTGCTACAATGGCGCATACAAAGAGAAGCGACCTCGCGAGAGCAAGCGGACCTCATAAAGTGCGTC
GTAGTCCGGATTGGAGTCTGCAACTCGACTCCATGAAGTCGGAATCGCTAGTAATCGTGGATCAGAATGC
CACGGTGAATACGTTCCCGGGCCTTGTACACACCGCCCGTCACACCATGGGAGTGGGTTGCAAAAGAAGT
AGGTAGCTTAACCTTCGGGAGGGCGCTACCCACTTTTTGTTAAGGGG
>239923984|gb|GQ179706.1|Morganella|Uncultured Morganella sp. clone VE3A10 16S ribosomal RNA gene, partial sequence
ATTGAACGCTGGCGGCAGGCCTAACACATGCAAGTCGGGCGGTAACAGGGAGAAGCTTGCTTCTCTGCTG
ACGAGCGGCGGACGGGTGAGTAATGTATGGGGATCTGCCTGATGGCGGGGGATAACTACTGGAAACGGTA
GCTAATACCGCATAATGTCTACGGACCAAAGCGGGGGACCTCCGGGCCTCGCGCCATCAGATGAACCCAT
ATGGGATTAGCTTGTAGGTGAGGTAACGGCTCACCTAGGCGACGATCCCTAGCTGGTCTGAGAGGATGAT
CAGCCACACTGGGACTGAGACACGGCCCAGACTCCTACGGGAGGCAGCAGTGGGGAATATTGCACAATGG
GCGCAAGCCTGATGCAGCCATGCCGCGTGTATGAAGAAGGCCTTCGGGTTGTAAAGTACTTTCAGTCGGG
AGGAAGGTGGTAAGGTTAATAACCTTATCAATTGACGTTACCGACAGAAGAAGCACCGGCTAACTCCGTG
CCAGCAGCCGCGGTAATACGGAGGGTGCAAGCGTTAATCGGAATTACTGGGCGTAAAGCGCACGCAGGCG
GTTGATTGAGTCAGATGTGAAATCCCCGGGCTTAACCCGGGAATTGCATCTGATACTGGTCAGCTAGAGT
CTTGTAGAGGGGGGTAGAATTCCATGTGTAGCGGTGAAATGCGTAGAGATGTGGAGGAATACCGGTGGCG
AAGGCGGCCCCCTGGACAAAGACTGACGCTCAGGTGCGAAAGCGTGGGGAGCAAACAGGATTAGATACCC
TGGTAGTCCACGCTGTAAACGATGTCGACTTGGAGGTTGTGCCCTTGAGGCGTGGCTTCCGGAGCTAACG
CGTTAAGTCGACCGCCTGGGGAGTACGGCCGCAAGGTTAAAACTCAAATGAATTGACGGGGGCCCGCACA
AGCGGTGGAGCATGTGGTTTAATTCGATGCAACGCGAAGAACCTTACCTACTCTTGACATCCAGAGAACT
TAGCAGAGATGCTTTGGTGCCTTCGGGAACTCTGAGACAGGTGCTGCATGGCTGTCGTCAGCTCGTGTTG
TGAAATGTTGGGTTAAGTCCCGCAACGAGCGCAACCCTTATCCTTTGTTGCCAGCGCGTGATGGCGGGAA
CTCAAAGGAGACTGCCGGTGATAAACCGGAGGAAGGTGGGGATGACGTCAAGTCATCATGGCCCTTACGA
GTAGGGCTACACACGTGCTACAATGGCGTATACAAAGGGAAGCGACCCCGCGAGGGCAAGCGGAACTCAT
AAAGTACGTCGTAGTCCGGATTGGAGTCTGCAACTCGACTCCATGAAGTCGGAATCGCTAGTAATCGTAG
ATCAGAATGCTACGGTGAATACGTTCCCGGGCCTTGTACACACCGCCCGTCACACCATGGGAGTGGGTTG
CAAAAGAAGTAGGTAGCTTAACCTCCGGGGGGGCGCTTACCACTTTGTGATTCATGACTGGGGTGAAGTC
GTAACAAGGTA
>81157826|emb|AM156948.1|Morganella|Morganella sp. TR 90 16S rRNA gene, isolate TR 90

GGAATTCGATTAGAGTTTGATCCTGGCTCAGATTGAACGCTGGCGGCAGGCCTAACACATGCAAGTCGGG
CGGTAACAGGGGGAAGCTTGCTTCTCTGCTGACGAGCGGCGGACGGGTGAGTAATGTATGGGGATCTGCC
TGATGGCGGGGGATAACTACTGGAAACGGTAGCTAATACCGCATAATGTCTTCGGACCAAAGCGGGGGAC
CTCAGGGCCTCGCGCCATCAGATGAACCCATATGGGATTAGCTAGTAGGTGAGGTAACGGCTTACCTAGG
CGACGATCCCTAGCTGGTCTGAGAGGATGATCAGCCACACTGGGACTGAGATACGGCCCAGACTCCTACG
GGTGGCAGCAGTGGGGAATATTGCACAATGGGCGCAAGCCTGATGCAGCCATGCCGCGTGTATGAAGAAG
GCCTTCGGGTTGTAAAGTACTTTCAGTCGGGAGGAAGGTGGTAAGGTTAATAACCTTATCAATTGACGTT
ACCGACAGAAGAAGCACCGGCTAACTCCGTGCCAGCAGCCGCGGTAATACGGAGGGTGCAAGCGTTAATC
GGAATTACTGGGCGTAAAGCGCACGCAGGCGGTTGATTGAGTCAGATGTGAAATCCCCGGGCTTAACCCG
GGAATTGCATCTGATACTGGTCAGCTAGAGTCTTGTAGAGGGGGGTAGAATTCCATGTGTAGCGGTGAAA
TGCGTAGAGATGTGGAGGAATACCGGTGGCGAAGGCGGCCCCCTGGACAAAGACTGACGCTCAGGTGCGA
AAGCGTGGGGAGCAAACAGGATTAGATACCCTGGTAGTCCACGCTGTAAACGATGTCGACTTGGAGGTTG
TGCCCTTGAGGCGTGGCTTCCGGAGCTAACGCGTTAAGTCGACCGCCTGGGGAGTACGGCCGCAAGGTTA
AAACTCAAATGAATTGACGGGGGCCCGCACAAGCGGTGGAGCATGTGGTTTAATTCGATGCAACGCGAAG
AACCTTACCTACTCTTGACATCCAGAGAACTTAGCAGAGATGCTTTGGTGCCTTCGGGAACTCTGAGACA
GGTGCTGCATGGCTGTCGTCAGCTCGTGTTGTGAAATGTTGGGTTAAGTCCCGCAACGAGCGCAACCCTT
ATCCTTTGTTGCCAGCGCGTGATGGCGGGAACTCAAAGGAGACTGCCGGTGATAAACCGGAGGAAGGTGG
GGATGACGTCAAGTCATCATGGCCCTTATGAGTAGGGCTACACACGTGCTACAATGGCGTATACAAAGGG
AAGCGACCCCGCGAGGGCAAGCGGAACTCATAAAGTACGTCGTAGTCCGGATTGGAGTCTGCAACTCGAC
TCCATGAAGTCGGAATCGCTAGTAATCGTAGATCAGAATGCTACGGTGAATACGTTCCCGGGCCTTGTAC
ACACCGCCCGTCACACCATGGGAGTGGGTTGCAAAAGAAGTAGGTAGCTTAACCTCCGGGAGGGCGCTTA
CCACTTTGTGATTCATGACTGGGGTGAAGTCGTAACAAGGTAACCGTAAATCACTAGTGAATTCGCGGCC
GCCTGCAG
>302746245|gb|HM122047.1|Morganella|Morganella morganii subsp. morganii strain ATCC 25830 16S ribosomal RNA gene, partial sequence
TCCAGAGTTTGATCATGGCTCAGATTGAACGCTGGCGGCAGGCCTAACACATGCAAGTCGGGCGGTAACA
GGGAGAAGCTTGCTTCTCTGCTGACGAGCGGCGGACGGGTGAGTAATGTATGGGGATCTGCCTGATGGCG
GGGGAGAACTACTGGAACGGTAGCTAATACCGCATAATGTCTTCGGACCAAAGCGGGGGACCTCCGGGCC
TCGCGCCATCAGATGAACCCATATGGGATTAGCTAGTAGGTGAGGTAACGGCTTACCTAGGCGACGATCC
CTAGCTGGTCTGAGAGGATGATCAGCCACACTGGGACTGAGACACGGCCCAGACTCCTACGGGAGGCAGC
AGTGGGGAATATTGCACAATGGGCGCAAGCCTGATGCAGCCATGCCGCGTGTATGAAGAAGGCCTTCGGG
TTGTAAAGTACTTTCAGTCGGGAGGAAGGTGTCAAGGTTAATAACCTTGGCAATTGACGTTACCGACAGA
AGAAGCACCGGCTAACTCCGTGCCAGCAGCCGCGGTAATACGGAGGGTGCAAGCGTTAATCGGAATTACT
GGGCGTAAAGCGCACGCAGGCGGTTGATTGAGTCAGATGTGAAATCCCCGGGCTTAACCCGGGAATTGCA
TCTGATACTGGTCAGCTAGAGTCTTGTAGAGGGGGGTAGAATTCCATGTGTAGCGGTGAAATGCGTAGAG
ATGTGGAGGAATACCGGTGGCGAAGGCGGCCCCCTGGGACAAGACTGACGCTCAGGTGCGAAAGCGTGGG
GAGCAAACAGGATTAGATACCCTGGTAGTCCACGCTGTAAACGATGTCGACTTGGAGGTTGTGCCCTTGA
GGCGTGGCTTCCGGAGCTAACGCGTTAAGTCGACCGCCTGGGGAGTACGGCCGCAAGGTTAAAACTCAAA
TGAATTGACGGGGGCCCGCACAAGCGGTGGAGCATGTGGTTTAATTCGATGCAACGCGAAGAACCTTACC
TACTCTTGACATCCAGAGAACTTAGCAGAGATGCTTTGGTGCCTTCGGGAACTCTGAGACAGGTGCTGCA
TGGCTGTCGTCAGCTCGTGTTGTGAAATGTTGGGTTAAGTCCCGCAACGAGCGCAACCCTTATCCTTTGT
TGCCAGCGCGTGATGGCGGGAACTCAAAGGAGACTGCCGGTGATAAACCGGAGGAAGGTGGGGATGACGT
CAAGTCATCATGGCCCTTACGAGTAGGGCTACACACGTGCTACAATGGCGTATACAAAGGGAAGCGACCC
CGCGAGGGCAAGCGGAACTCATAAAGTACGTCGTAGTCCGGATTGGAGTCTGCAACTCGACTCCATGAAG
TCGGAATCGCTAGTAATCGTAGATCAGAATGCTACGGTGAATACGTTCCCGGGCCTTGTACACACCGCCC
GTCACACCATGGGAGTGGGTTGCAAAAGAAGTATGTAGCTTAACCTCCGGGAGGGCGCTTACCACTTTGT
G
>171191216|gb|EU373416.1|Providencia|Providencia sp. YRL09 16S ribosomal RNA gene, partial sequence
CGGAGAGTTTGATCCTGGCTCAGATTGAACGCTGGCGGCAGGCCTAACACATGCAAGTCGAGCGGTAACA
GGGGAAGCTTGCTTCCCGCTGACGAGCGGCGGACGGGTGAGTAATGTATGGGGATCTGCCCGATAGAGGG
GGATAACTACTGGAAACGGTAGCTAATACCGCATAATCTCTAAGGAGCAAAGCAGGGGAACTTCGGTCCT
TGCGCTATCGGATGAACCCATATGGGATTAGCTAGTAGGTGAGGTAATGGCTCACCTAGGCGACGATCCC
TAGCTGGTCTGAGAGGATGATCAGCCACACTGGGACTGAGACACGGCCCAGACTCCTACGGGAGGCAGCA

GTGGGGAATATTGCACAATGGGCGCAAGCCTGATGCAGCCATGCCGCGTGTATGAAGAAGGCCCTAGGGT
TGTAAAGTACTTTCAGTCGGGAGGAAGGCGTTGATGCTAATATCATCAACGATTGACGTTACCGACAGAA
GAAGCACCGGCTAACTCCGTGCCAGCAGCCGCGGTAATACGGAGGGTGCAAGCGTTAATCGGAATTACTG
GGCGTAAAGCGCACGCAGGCGGTTGATTAAGTTAGATGTGAAATCCCCGGGCTTAACCTGGGAATGGCAT
CTAAGACTGGTCAGCTAGAGTCTTGTAGAGGGGGGTAGAATTCCATGTGTAGCGGTGAAATGCGTAGAGA
TGTGGAGGAATACCGGTGGCGAAGGCGGCCCCCTGGACAAAGACTGACGCTCAGGTGCGAAAGCGTGGGG
AGCAAACAGGATTAGATACCCTGGTAGTCCACGCTGTAAACGATGTCGATTTGAAGGTTGTTCCCTTGAG
GAGTGGCTTTCGGAGCTAACGCGTTAAATCGACCGCCTGGGGAGTACGGCCGCAAGGTTAAAACTCAAAT
GAATTGACGGGGGCCCGCACAAGCGGTGGAGCATGTGGTTTAATTCGATGCAACGCGAAGAACCTTACCT
ACTCTTGACATCCAGAGAATTTAGCAGAGATGCTTTAGTGCCTTCGGGAACTCTGAGACAGGTGCTGCAT
GGCTGTCGTCAGCTCGTGTTGTGAAATGTTGGGTTAAGTCCCGCAACGAGCGCAACCCTTATCCTTTGTT
GCCAGCGATTCGGTCGGGAACTCAAAGGAGACTGCCGGTGATAAACCGGAGGAAGGTGGGGATGACGTCA
AGTCATCATGGCCCTTACGAGTAGGGCTACACACGTGCTACAATGGCGTATACAAAGAGAAGCGACCTCG
CGAGAGCAAGCGGAACTCATAAAGTACGTCGTAGTCCGGATTGGAGTCTGCAACTCGACTCCATGAAGTC
GGAATCGCTAGTAATCGTAGATCAGAATGCTACGGTGAATACGTTCCCGGGCCTTGTACACACCGCCCGT
CACACCATGGGAGTGGGTTGCAAAAGAAGTAGGTAGCTTAACCTTCGGGAGGGCGCTTACCACTTTGTGA
TTCATGACTGGGGTGAAGTCGTAACAAGGTAGCCGTA
>66878658|gb|AY958952.1|Providencia|Uncultured bacterium clone rRNA179
16S ribosomal RNA gene, partial sequence
CCCTTAGAGTTTGATCCTGGCTCAGATTGAACGCTGGCGGCAGGCCTAACACATGCAAGTCGAGCGGTAA
CAGGGGAAAGCTTGCTTTCCCGCTGACGAGCGGCGGACGGGTGAGTAATGTATGGGGATCTGCCCGATAG
AGGGGGATAACTACTGGAAACGGTGGCTAATACCGCATAATCTCTAAGGAGCAAAGCAGGGGAACTTCGG
TCCTTGCGCTATCGGATGAACCCATATGGGATTAGCTAGTAGGTGAGGTAATGGCTCACCTAGGCGACGA
TCCCTAGCTGGTCTGAGAGGATGATCAGCCACACTGGGACTGAGACACGGCCCAGACTCCTACGGGAGGC
AGCAGTGGGGAATATTGCACAATGGGCGCAAGCCTGATGCAGCCATGCCGCGTGTATGAAGAAGGCCTTA
GGGTTGTAAAGTACTTTCAGTCGGGAGGAAGGCGTTGATGCGAATACCATCAGCGATTGACGTTACCGAC
AGAAGAAGCACCGGCTAACTCCGTGCCAGCAGCCGCGGTAATACGGAGGGTGCAAGCGTTAATCGGAATT
ACTGGGCGTAAAGCGCATGCAGGCGGTTGATTAAGTTAGATGTGAAATCCCCGGGCTTAACCTGGGAATG
GCATCTAAAACTGGTCAGCTAGAGTCTTGTAGAGGGGGGTAGAATTCCATGTGTAGCGGTGAAATGCGTA
GAGATGTGGAGGAATACCGGTGGCGAAGGCGGCCCCCTGGACAAAGACTGACGCTCAGATGCGAAAGCGT
GGGGAGCAAACAGGATTAGATACCCTGGTAGTCCACGCTGTAAACGATGTCGACTTGAAGGTTGTTCCCT
TGAGGAGTGGCTTTCGGAGCTAACGCGTTAAATCGACCGCCTGGGGAGTACGGCCGCAAGGTTAAAACTC
AAATGAATTGACGGGGGCCCGCACAAGCGGTGGAGCATGTGGTTTAATTCGATGCAACGCGAAGAACCTT
ACCTACTCTTGACATCCAGAGAACTTAGCAGAGATGCTTTGGCGCCTTCGGGAACTCTGAGACAGGTGCT
GCATGGCTGTCGTCAGCTCGTGTTGTGAAATGTTGGGTTAAGTCCCGCAACGAGCGCAACCCTTATCCTT
TGTTGCCAGCACGTGATGGTGGGAACTCAAAGGAGACTGCCGGTGATAAACCGGAGGAAGGTGGGGATGA
CGTCAAGTCATCATGGCCCTTACGAGTAGGGCTACACACGTGCTACAATGGCGTATACAAAGAGAAGCAA
ACTCGCGAGAGCTAGCGGAACTCATAAAGTACGTCGTAGTCCGGATTGGAGTCTGCAACTCGACTCCATG
AAGTCGGAATCGCTAGTAATCGTAGATCAGAATGCTACGGTGAATACGTTCCCGGGCCTTGTACACACCG
CCCGTCACACCATGGGAGTGGGTTGCAAAAGAAGTAGGTAGCTTAACCTTCGGGAGGGCGCTTACCACTT
TGTGATTCATGACTGGGGTGAA
>310975248|ref|NR_037112.1|Serratia|Serratia proteamaculans strain 4364
16S ribosomal RNA, partial sequence
TGGCTCAGATTGAACGCTGGCGGCAGGCCTAACACATGCAAGTCGAGCGGTAGCACAGGAGAGCTTGCTC
TCTGGGTGACGAGCGGCGGACGGGTGAGTAATGTCTGGGAAACTGCCTGATGGAGGGGGATAACTACTGG
AAACGGTAGCTAATACCGCATAACGTCTTCGGACCAAAGTGGGGGACCTTCGGGCCTCACGCCATCAGAT
GTGCCCAGATGGGATTAGCTAGTAGGTGGGGTAATGGCTCACCTAGGCGACGATCCCTAGCTGGTCTGAG
AGGATGACCAGCCACACTGGAACTGAGACACGGTCCAGACTCCTACGGGAGGCAGCAGTGGGGAATATTG
CACAATGGGCGCAAGCCTGATGCAGCCATGCCGCGTGTGTGAAGAAGGCCTTCGGGTTGTAAAGCACTTT
CAGCGAGGAGGAAGGGTAGTGTGTTAATAGCACATTGCATTGACGTTACTCGCAGAAGAAGCACCGGCTA
ACTCCGTGCCAGCAGCCGCGGTAATACGGAGGGTGCAAGCGTTAATCGGAATTACTGGGCGTAAAGCGCA
CGCAGGCGGTTTGTTAAGTCAGATGTGAAATCCCCGCGCTTAACGTGGGAACTGCATTTGAAACTGGCAA
GCTAGAGTCTTGTAGAGGGGGGTAGAATTCCAGGTGTAGCGGTGAAATGCGTAGAGATCTGGAGGAATAC
CGGTGGCGAAGGCGGCCCCCTGGACAAAGACTGACGCTCAGGTGCGAAAGCGTGGGGAGCAAACAGGATT

AGATACCCTGGTAGTCCACGCTGTAAACGATGTCGACTTGGAGGTTGTGCCCTTGAGGCGTGGCTTCCGG
AGCTAACGCGTTAAGTCGACCGCCTGGGGAGTACGGCCGCAAGGTTAAAACTCAAATGAATTGACGGGGG
CCCGCACAAGCGGTGGAGCATGTGGTTTAATTCGATGCAACGCGAAGAACCTTACCTACTCTTGACATCC
AGAGAATTCGCTAGAGATAGCTTAGTGCCTTCGGGAACTCTGAGACAGGTGCTGCATGGCTGTCGTCAGC
TCGTGTTGTGAAATGTTGGGTTAAGTCCCGCAACGAGCGCAACCCTTATCCTTTGTTGCCAGCACGTAAT
GGTGGGAACTCAAAGGAGACTGCCGGTGATAAACCGGAGGAAGGTGGGGATGACGTCAAGTCATCATGGC
CCTTACGAGTAGGGCTACACACGTGCTACAATGGCGTATACAAAGAGAAGCGAACTCGCGAGAGCCAGCG
GACCTCATAAAGTACGTCGTAGTCCGGATCGGAGTCTGCAACTCGACTCCGTGAAGTCGGAATCGCTAGT
AATCGTAGATCAGAATGCTACGGTGAATACGTTCCCGGGCCTTGTACACACCGCCCGTCACACCATGGGA
GTGGGTTGCAAAAGAAGTAGGTAGCTTAACCTTCGGGAGGGCGCTTACCACTTTGTGATTCATGACTGGG
GTGAAGTCGTAACAAGGTAACCGTAGGGGAACCTGCGG
>66878677|gb|AY958971.1|Serratia|Uncultured bacterium clone rRNA198 16S ribosomal RNA gene, partial sequence
CCTTAGAGTTTGATCCTGGCTCAGATTGAACGCTGGCGGCAGGCCTAACACATGCAAGTCGAGCGGTAGC
ACAAGAGAGCTTGCTCTCTGGGTGACGAGCGGCGGACGGGTGAGTAATGTCTGGGAAACTGCCTGATGGA
GGGGGATAACTACTGGAAACGGTAGCTAATACCGCATAACGTCTTCGGACCAAAGTGGGGGACCTTCGGG
CCTCACGCCATCAGATGTGCCCAGATGGGATTAGCTAGTAGGTGGGGTAATGGCTCACCTAGGCGACGAT
CCCTAGCTGGTCTGAGAGGATGACCAGCCACACTGGAACTGAGACACGGTCCAGACTCCTACGGGAGGCA
GCAGTGGGGAATATTGCACAATGGGCGCAAGCCTGATGCAGCCATGCCGCGTGTGTGAAGAAGGCCTTCG
GGTTGTAAAGCACTTTCAGCGAGGAGGAAGGGTAGTGTGTTAATAGCACATTGCATTGACGTTACTCGCA
GAAGAAGCACCGGCTAACTCCGTGCCAGCAGCCGCGGTAATACGGAGGGTGCAAGCGTTAATCGGAATTA
CTGGGCGTAAAGCGCACGCAGGCGGTTTGTTAAGTCAGATGTGAAATCCCCGCGCTTAACGTGGGAACTG
CATTTGAAACTGGCAAGCTAGAGTCTTGTAGAGGGGGGTAGAATTCCAGGTGTAGCGGTGAAATGCGTAG
AGATCTGGAGGAATACCGGTGGCGAAGGCGGCCCCCTGGACAAAGACTGACGCTCAGGTGCGAAAGCGTG
GGGAGCAAACAGGATTAGATACCCTGGTAGTCCACGCTGTAAACGATGTCGACTTGGAGGTTGTGCCCTT
GAGGCGTGGCTTCCGGAGCTAACGCGTTAAGTCGACCGCCTGGGGAGTACGGCCGCAAGGTTAAAACTCA
AATGAATTGACGGGGGCCCGCACAAGCGGTGGAGCATGTGGTTTAATTCGATGCAACGCGAAGAACCTTA
CCTACTCTTGACATCCAGAGAATTCGCTAGAGATAGCTTAGTGCCTTCGGGAACTCTGAGACAGGTGCTG
CATGGCTGTCGTCAGCTCGTGTTGTGAAATGTTGGGTTAAGTCCCGCAACGAGCGCAACCCTTATCCTTT
GTTGCCAGCACGTAATGGTGGGAACTCAAAGGAGACTGCCGGTGATAAACCGGAGGAAGGTGGGGATGAC
GTCAAGTCATCATGGCCCTTACGAGTAGGGCTACACACGTGCTACAATGGCGTATACAAAGAGAAGCGAA
CTCGCGAGAGCAAGCGGACCTCATAAAGTACGTCGTAGTCCGGATCGGAGTCTGCAACTCGACTCCGTGA
AGTCGGAATCGCTAGTAATCGTAGATCAGAATGCTACGGTGAATACGTTCCCGGGCCTTGTACACACCGC
CCGTCACACCATGGGAGTGGGTTGCAAAAGAAGTAGGTAGCTTAACCTTCGGGAGGGCGCTTACCACTTT
GTGATTCATGACTGGGGTGAAGTCGTAACAAGGTAGCCGTAAAG
>28624924|gb|AY167282.1|Halomonas|Halomonas variabilis strain ANT9112 16S ribosomal RNA gene, partial sequence
GAACGCTGGCGGCAGGCCTAACACATGCAAGTCGAGCGGTAACAGATCTAGCTTGCTAGATGCTGACGAG
CGGCGGACGGGTGAGTAATGCATAGGAATCTGCCCGATAGTGGGGGATAACCTGGGGAAACCCAGGCTAA
TACCGCATACGTCCTACGGGAGAAAGGGGGCTCCGGCTCCCGCTATTGGATGAGCCTATGTCGGATTAGC
TAGTTGGTGAGGTAAAGGCTCACCAAGGCGACGATCCGTAGCTGGTCTGAGAGGATGATCAGCCACATCG
GGACTGAGACACGGCCCGAACTCCTACGGGAGGCAGCAGTGGGGAATATTGGACAATGGGGGCAACCCTG
ATCCAGCCATGCCGCGTGTGTGAAGAAGGCCCTCGGGTTGTAAAGCACTTTCAGCGAGGAAGAACGCCTG
TCGGTTAATACCCGGCAGGAAAGACATCACTCGCAGAAGAAGCACCGGCTAACTCCGTGCCAGCAGCCGC
GGTAATACGGAGGGTGCAAGCGTTAATCGGAATTACTGGGCGTAAAGCGCGCGTAGGTGGCTTGATAAGC
CGGTTGTGAAAGCCCCGGGCTCAACCTGGGAACGGCATCCGGAACTGTCAGGCTAGAGTGCAGGAGAGGA
AGGTAGAATTCCCGGTGTAGCGGTGAAATGCGTAGAGATCGGGAGGAATACCAGTGGCGAAGGCGGCCTT
TCTGGACTGACACTGACACTGAGGTGCGAAAGCGTGGGTAGCAAACAGGATTAGATACCCTGGTAGTCCA
CGCCGTAAACGATGTCGACCAGCCGTTGGGTGCCTAGAGCACTTTGTGGCGAAGTTAACGCGATAAGTCG
ACCGCCTGGGGAGTACGGCCGCAAGGTTAAAACTCAAATGAATTGACGGGGGCCCGCACAAGCGGTGGAG
CATGTGGTTTAATTCGATGCAACGCGAAGAACCTTACCTACCCTTGACATCTACAGAAGCCGGAAGAGAT
TCTGGTGTGCCTTCGGGAACTGTAAGACAGGTGCTGCATGGCTGTCGTCAGCTCGTGTTGTGAAATGTTG
GGTTAAGTCCCGTAACGAGCGCAACCCTTGTCCTTATTTGCCAGCGAGTAATGTCGGGAACTCTAAGGAG
ACTGCCGGTGACAAACCGGAGGAAGGTGGGGACGACGTCAAGTCATCATGGCCCTTACGGGTAGGGCTAC

ACACGTGCTACAATGGCCGGTACAAAGGGCTGCGAGCTCGCGAGAGTCAGCGAATCCCTTAAAGCCGGTC
TCAGTCCGGATCGGAGTCTGCAACTCGACTCCGTGAAGTCGGAATCGCTAGTAATCGTGAATCAGAATGT
CACGGTGAATACGTTCCCGGGCCTTGTACACACCGCCCGTCACACCATGGGAGTGGACTGCACCAGAAGT
GGTTAGCCTAACGCAAGAGGGCGATCACCACGGTGTGGTTCATGACTGGGGTGAAGTCGTAACAAGGTAG
CCGTAGGGGAACC
>66878536|gb|AY958830.1|Halomonas|Uncultured bacterium clone rRNA057 16S ribosomal RNA gene, partial sequence
TTGAACGCTGGCGGCAGGCCTAACACATGCAAGTCGAGCGGTAGCACAAAGAAGCTTGCTTCTTGGGTGA
CGAGCGGCGGACGGGTGAGTAATGCATAGGAATCTGCCCGATAGTGGGGGATAACCTGGGGAAACCCAGG
CTAATACCGCATACGTCCTACGGGAGAAAGGGGGCTTCGGCTCCCGCTATTGGATGAGCCTATGTCGGAT
TAGCTAGTTGGTAGGGTAAAGGCCTACCAAGGCGACGATCCGTAGCTGGTCTGAGAGGATGATCAGCCAC
ATCGGGACTGAGACACGGCCCGAACTCCTACGGGAGGCAGCAGTGGGGAATATTGGACAATGGGGGCAAC
CCTGATCCAGCCATGCCGCGTGTGTGAAGAAGGCCCTCGGGTTGTAAAGCACTTTCAGCGAGGAAGAACG
CCTAGTGGTTAATACCCATTAGGAAAGACATCACTCGCAGAAGAAGCACCGGCTAACTCCGTGCCAGCAG
CCGCGGTAATACGGAGGGTGCAAGCGTTAATCGGAATTACTGGGCGTAAAGCGCGCGTAGGTGGCTTGAT
AAGCCGGTTGTGAAAGCCCCGGGCTCAACCTGGGAACGGCATCCGGAACTGTCAGGCTAGAGTGCAGGAG
AGGAAGGTAGAATTCCCGGTGTAGCGGTGAAATGCGTAGAGATCGGGAGGAATACCAGTGGCGAAGGCGG
CCTTCTGGACTGACACTGACACTGAGGTGCGAAAGCGTGGGTAGCAAACAGGATTAGATACCCTGGTAGT
CCACGCCGTAAACGATGTCGACCAGCCGTTGGGTGCCTAGCGCACTTTGTGGCGAAGTTAACGCGATAAG
TCGACCGCCTGGGGAGTACGGCCGCAAGGTTAAAACTCAAATGAATTGACGGGGGCCCGCACAAGCGGTG
GAGCATGTGGTTTAATTCGATGCAACGCGAAGAACCTTACCTACTCTTGACATCTACAGAAGCCGGAAGA
GATTCTGGTGTGCCTTCGGGAACTGTAAGACAGGTGCTGCATGGCTGTCGTCAGCTCGTGTTGTGAAATG
TTGGGTTAAGTCCCGTAACGAGCGCAACCCTTGTCCTTATTTGCCAGCGAGTAATGTCGGGAACTCTAAG
GAGACTGCCGGTGACAAACCGGAGGAAGGTGGGGACGACGTCAAGTCATCATGGCCCTTACGAGTAGGGC
TACACACGTGCTACAATGGCCGGTACAAAGGGCTGCGAGCTCGCGAGAGTCAGCGAATCCCTTAAAGCCG
GTCTCAGTCCGGATCGGAGTCTGCAACTCGACTCCGTGAAGTCGGAATCGCTAGTAATCGTGAATCAGAA
TGTCACGGTGAATACGTTCCCGGGCCTTGTACACACCGCCCGTCACACCATGGGAGTGGACTGCACCAGA
AGTGGTTAGCCTAACGCAAGAGGGCGATCACCACGGTGTGGTTCATGACTGGGGTGAAGTCGTAACAAGG
TAACCGTAAAGGGCG
>155001223|gb|EU083530.1|Haemophilus|Haemophilus parainfluenzae strain CIP 102513 16S ribosomal RNA gene, partial sequence
TAGAGTTTGATCCTGGCTCAGATTGAACGCTGGCGGCAGGCTTAACACATGCAAGTCGAACGGTAACATA
AAGAAGCTTGCTTCTTTGATGACGAGTGGCGGACGGGTGAGTAATGCTTGGGAATCTAGCTTATGGAGGG
GGATAACTACGGGAAACTGTAGCTAATACCGCGTAGAATCGAAAGATGAAAGTGTGGGACCTTCGGGCCA
CATGCCATAGGATGAGCCCAAGTGGGATTAGGTAGTTGGTGAGGTAAAGGCTCACCAAGCCGACGATCTC
TAGCTGGTCTGAGAGGATGACCAGCCACACTGGGACTGAGACACGGCCCAGACTCCTACGGGAGGCAGCA
GTGGGGAATATTGCGCAATGGGGGCAACCCTGACGCAGCCATGCCGCGTGAATGAAGAAGGCCTTCGGGT
TGTAAAGTTCTTTCGGTAGCGAGGAAGGCATTTAGTTTAATAGACTAGGTGATTGACGTTAACTACAGAA
GAAGCACCGGCTAACTCCGTGCCAGCAGCCGCGGTAATACGGAGGGTGCGAGCGTTAATCGGAATAACTG
GGCGTAAAGGGCACGCAGGCGGTGACTTAAGTGAGGTGTGAAAGCCCCGGGCTTAACCTGGGAATTGCAT
TTCATACTGGGTCGCTAGAGTACTTTAGGGAGGGGTAGAATTCCACGTGTAGCGGTGAAATGCGTAGAGA
TGTGGAGGAATACCGAAGGCGAAGGCAGCCCCTTGGGAATGTACTGACGCTCATGTGCGAAAGCGTGGGG
AGCAAACAGGATTAGATACCCTGGTAGTCCACGCTGTAAACGATGTCGATTTGGGGGTTGAGCTTTAAGC
TTGGCGCCCGTAGCTAACGTGATAAATCGACCGCCTGGGGAGTACGGCCGCAAGGTTAAAACTCAAATGA
ATTGACGGGGGCCCGCACAAGCGGTGGAGCATGTGGTTTAATTCGATGCAACGCGAAGAACCTTACCTAC
TCTTGACATCCAGAGAACATTCCAGAGATGGATTGGTGCCTTCGGGAACTCTGAGACAGGTGCTGCATGG
CTGTCGTCAGCTCGTGTTGTGAAATGTTGGGTTAAGTCCCGCAACGAGCGCAACCCTTATCCTTTGTTGC
CAGCGATTCGGTCGGGAACTCAAAGGAGACTGCCGGTGATAAACCGGAGGAAGGTGGGGATGACGTCAAG
TCATCATGGCCCTTACGAGTAGGGCTACACACGTGCTACAATGGCGTATACAGAGGGAAGCGAGAGTGCG
AGCTGGAGCGAATCTCACAAAGTACGTCTAAGTCCGGATTGGAGTCTGCAACTCGACTCCATGAAGTCGG
AATCGCTAGTAATCGCAAATCAGAATGTTGCGGTGAATACGTTCCCGGGCCTTGTACACACCGCCCGTCA
CACCATGGGAGTGGGTTGTACCAGAAGTAGATAGCTTAACCTTCGGGGGGGCGTTTACCACGGTATGATT
CATGACTGGGGTGAAGTCGTAACAAGGTAGCCGTA

>66878748|gb|AY959042.1|Haemophilus|Uncultured bacterium clone rRNA269 16S ribosomal RNA gene, partial sequence
CCCTTAGAGTTTGATCCTGGCTCAGATTGAACGCTGGCGGCAGGCTTAACACATGCAAGTCGAACGGTAA
CATAAAGGAGCTTGCTTCTTTGATGACGAGTGGCGGACGGGTGAGTAATGCTTGGGAATCTAGCTTATGG
AGGGGGATAACTACGGGAAACTGTAGCTAATACCGCGTAATATCGAAAGATTAAAGTGTGGGACCTTCGG
GCCACATGCCATAAGATGAGCCCAAGTGGGATTAGGTAGTTGGTGAGGTAAAGGCTCACCAAGCCGACGA
TCTCTAGCTGGTCTGAGAGGATGACCAGCCACACTGGGACTGAGACACGGCCCAGACTCCTACGGGAGGC
AGCAGTGGGGAATATTGCGCAATGGGGGCAACCCTGACGCAGCCATGCCGCGTGAATGAAGAAGGCCTTC
GGGTTGTAAAGTTCTTTCGGTAGCGAGGAAGGCATTTAGTTTAATAGACTAGATGATTGACGTTAACTAC
AGAAGAAGCACCGGCTAACTCCGTGCCAGCAGCCGCGGTAATACGGAGGGTGCGAGCGTTAATCGGAATA
ACTGGGCGTAAAGGGCACGCAGGCGGTGACTTAAGTGAGGTGTGAAAGCCCCGGGCTTAACCTGGGAATT
GCATTTCATACTGGGTCGCTAGAGTACTTTAGGGAGGGGTAGAATTCCACGTGTAGCGGTGAAATGCGTA
GAGATGTGGAGGAATACCGAAGGCGAAGGCAGCCCCTTGGGAATGTACTGACGCTCATGTGCGAAAGCGT
GGGGAGCAAACAGGATTAGATACCCTGGTAGTCCACGCTGTAAACGATGTCGATTTGGGGGTTGAGCTTT
AAGTTTGGCGCCCGTAGCTAACGTGATAAATCGACCGCCTGGGGAGTACGGCCGCAAGGTTAAAACTCAA
ATGAATTGACGGGGGCCCGCACAAGCGGTGGAGCATGTGGTTTAATTCGATGCAACGCGAAGAACCTTAC
CTACTCTTGACATCCAGAGAACTTTCCAGAGATGGATTGGTGCCTTCGGGAACTCTGAGACAGGTGCTGC
ATGGCTGTCGTCAGCTCGTGTTGTGAAATGTTGGGTTAAGTCCCGCAACGAGCGCAACCCTTATCCTTTG
TTGCCAGCGATTCGGTCGGGAACTCAAAGGAGACTGCCGGTGATAAACCGGAGGAAGGTGGGGATGACGT
CAAGTCATCATGGCCCTTACGAGTAGGGCTACACACGTGCTACAATGGCGTATACAGAGGGAAGCGATAG
TGCGAGCTGGAGCGAATCTCACAAAGTACGTCTAAGTCCGGATTGGAGTCTGCAACTCGACTCCATGAAG
TCGGAATCGCTAGTAATCGCAAATCAGAATGTTGCGGTGAATACGTTCCCGGGCCTTGTACACACCGCCC
GTCACACCATGGGAGTGGGTTGTACCAGAAGTAGATAGCTTAACCTTCGGGAGGGCGTTTACCACGGTAT
GATTCATGACTGGGGTGAA
>239923954|gb|GQ179676.1|Haemophilus|Uncultured Haemophilus sp. clone VE4A10 16S ribosomal RNA gene, partial sequence
ATTGAACGCTGGCGGCAGGCTTAACACATGCAAGTCGAACGGTAACATAAAGAAGCTTGCTTCTTTGATG
ACGAGTGGCGGACGGGTGAGTAATGCTTGGGAATCTAGCTTATGGAGGGGGATAACTACGGGAAACTGTA
GCTAATACCGCGTAATATCGAAAGATGAAAGTGTGGGACCTTCGGGCCACATGCCATAGGATGAGCCCAA
GTGGGATTAGGTAGTTGGTGAGGTAAAGGCTCACCAAGCCGACGATCTCTAGCTGGTCTGAGAGGATGAC
CAGCCACACTGGGACTGAGACACGGCCCAGACTCCTACGGGAGGCAGCAGTGGGGAATATTGCGCAATGG
GGGCAACCCTGACGCAGCCATGCCGCGTGAATGAAGAAGGCCTTCGGGTTGTAAAGTTCTTTCGGTAGCG
AGGAAGGCATTTAGTTTAATAGACTAGGTGATTGACGTTAACTACAGAAGAAGCACCGGCTAACTCCGTG
CCAGCAGCCGCGGTAATACGGGGGGTGCGAGCGTTAATCGGAATAACTGGGCGTAAAGGGCACGCAGGCG
GTGACTTAAGTGAGGTGTGAAAGCCCCGGGCTTAACCTGGGAATTGCATTTCATACTGGGTCGCTAGAGT
ACTTTAGGGAGGGGTAGAATTCCACGTGTAGCGGTGAAATGCGTAGAGATGTGGAGGAATACCGAAGGCG
AAGGCAGCCCCTTGGGAATGTACTGACGCTCATGTGCGAAAGCGTGGGGAGCAAACAGGATTAGATACCC
TGGTAGTCCACGCTGTAAACGATGTCGATTTGGGGGTTAAGCTTTGAGCTTGGCGCCCGTAGCTAACGTG
ATAAATCGACCGCCTGGGGAGTACGGCCGCAAGGTTAAAACTCAAATGAATTGACGGGGGCCCGCACAAG
CGGTGGAGCATGTGGTTTAATTCGATGCAACGCGAAGAACCTTACCTACTCTTGACATCCAGAGAACTTT
CCAGAGATGGATTGGTGCCTTCGGGAACTCTGAGACAGGTGCTGCATGGCTGTCGTCAGCTCGTGTTGTG
AAATGTTGGGTTAAGTCCCGCAACGAGCGCAACCCTTATCCTTTGTTGCCAGCGATTTGGTCGGGAACTC
AAAGGAGACTGCCGGTGATAAACCGGAGGAAGGTGGGGATGACGTCAAGTCATCATGGCCCTTACGAGTA
GGGCTACACACGTGCTACAATGGCGTATACAGAGGGAAGCGAGAGTGCGAGCTGGAGCGAATCTCACAAA
GTACGTCTAAGTCCGGATTGGAGTCTGCAACTCGACTCCATGAAGTCGGAATCGCTAGTAATCGCAAATC
AGAATGTTGCGGTGAATACGTTCCCGGGCCTTGTACACACCGCCCGTCACACCATGGGAGTGGGTTGTAC
CAGAAGTAGATAGCTTAACCTTTTGGAGGGCGTTTACCACGGTATGATTCATGACTGGGGTGAAGTCGTA
ACAAGGTA
>294721598|gb|GU145275.1|Acinetobacter|Acinetobacter radioresistens strain S13 16S ribosomal RNA gene, partial sequence
TTTGATCATGGCTCAGATTGAACGCTGGCGGCAGGCTTAACACATGCAAGTCGAGCGGATGAAGGTAGCT
TGCTACTGGATTCAGCGGCGGACGGGTGAGTAATGCTTAGGAATCTGCCTATTAGTGGGGGACAACGTTC
CGAAAGGAGCGCTAATACCGCATACGTCCTACGGGAGAAAGCAGGGGACCTTTGGGCCTTGCGCTAATAG
ATGAGCCTAAGTCGGATTAGCTAGTTGGTAGGGTAAAGGCCTACCAAGGCGACGATCTGTAGCGGGTCTG

AGAGGATGATCCGCCACACTGGGACTGAGACACGGCCCAGACTCCTACGGGAGGCAGCAGTGGGGAATAT
TGGACAATGGGGGGAACCCTGATCCAGCCATGCCGCGTGTGTGAAGAAGGCCTTTTGGTTGTAAAGCACT
TTAAGCGAGGAGGAGGCTACCTAGATTAATACTTTAGGATAGTGGACGTTACTCGCAGAATAAGCACCGG
CTAACTCTGTGCCAGCAGCCGCGGTAATACAGAGGGTGCGAGCGTTAATCGGATTTACTGGGCGTAAAGC
GTGCGTAGGCGGCCAATTAAGTCAAATGTGAAATCCCCGAGCTTAACTTGGGAATTGCATTCGATACTGG
TTGGCTAGAGTATGGGAGAGGATGGTAGAATTCCAGGTGTAGCGGTGAAATGCGTAGAGATCTGGAGGAA
TACCGATGGCGAAGGCAGCCATCTGGCCTAATACTGACGCTGAGGTACGAAAGCATGGGGAGCAAACAGG
ATTAGATACCCTGGTAGTCCATGCCGTAAACGATGTCTACTAGCCGTTGGGGCCCTTGAGGCTTTAGTGG
CGCAGCTAACGCGATAAGTAGACCGCCTGGGGAGTACGGTCGCAAGACTAAAACTCAAATGAATTGACGG
GGGCCCGCACAAGCGGTGGAGCATGTGGTTTAATTCGATGCAACGCGAAGAACCTTACCTGGCCTTGACA
TACAGAGAACTTTCCAGAGATGGATTGGTGCCTTCGGGAACTCTGATACAGGTGCTGCATGGCTGTCGTC
AGCTCGTGTCGTGAGATGTTGGGTTAAGTCCCGCAACGAGCGCAACCCTTTTCCTTATTTGCCAGCACTT
CGGGTGGGAACTTTAAGGATACTGCCAGTGACAAACTGGAGGAAGGCGGGGACGACGTCAAGTCATCATG
GCCCTTACGGCCAGGGCTACACACGTGCTACAATGGTCGGTACAAAGGGTTGCTACACAGCGATGTGATG
CTAATCTCAAAAAGCCGATCGTAGTCCGGATTGGAGTCTGCAACTCGACTCCATGAAGTCGGAATCGCTA
GTAATCGCGGATCAGAATGCCGCGGTGAATACGTTCCCGGGCCTTGTACACACCGCCCGTCACACCATGG
GAGTTTGTTGCACCAGAAGTAGGTAGTCTAACCGCAAGGAGGACGCTTACCACGGTGTGGCCGATGACTG
GGGTGAAGTCGTAACAAGGA
>66878500|gb|AY958794.1|Acinetobacter|Uncultured bacterium clone rRNA021
16S ribosomal RNA gene, partial sequence
CCTTAGAGTTTGATCCTGGCTCAGATTGAACGCTGGCGGCAGGCTTAACACATGCAAGTCGAGCGGATGA
AGGTAGCTTGCCACCGGATTCAGCGGCGGACGGGTGAGTAATGCTTAGGAATCTGCCTATTAGTGGGGGA
CAACGTTCCGAAAGGAGCGCTAATACCGCATACGTCCTACGGGAGAAAGCAGGGGACCTTCGGGCCTTGC
GCTAATAGATGAGCCTAAGTCGGATTAGCTAGTTGGTAGGGTAAAGGCCTACCAAGGCGACGATCTGTAG
CGGGTCTGAGAGGATGATCCGCCACACTGGGACTGAGACACGGCCCAGACTCCTACGGGAGGCAGCAGTG
GGGAATATTGGACAATGGGGGGAACCCTGATCCAGCCATGCCGCGTGTGTGAAGAAGGCCTTTTGGTTGT
AAAGCACTTTAAGCGAGGAGGAGGCTACCTAGATTAATACTTTAGGATAGTGGACGTTACTCGCAGAATA
AGCACCGGCTAACTCTGTGCCAGCAGCCGCGGTAATACAGAGGGTGCGAGCGTTAATCGGATTTACTGGG
CGTAAAGCGTGCGTAGGCGGCCAATTAAGTCAAATGTGAAATCCCCGAGCTTAACTTGGGAATTGCATTC
GATACTGGTTGGCTAGAGTATGGGAGAGGATGGTAGAATTCCAGGTGTAGCGGTGAAATGCGTAGAGATC
TGGAGGAATACCGATGGCGAAGGCAGCCATCTGGCCTAATACTGACGCTGAGGTACGAAAGCATGGGGAG
CAAACAGGATTAGATACCCTGGTAGTCCATGCCGTAAACGATGTCTACTAGCCGTTGGGGCCCTTGAGGC
TTTAGTGGCGCAGCTAACGCGATAAGTAGACCGCCTGGGGAGTACGGTCGCAAGACTAAAACTCAAATGA
ATTGACGGGGGCCCGCACAAGCGGTGGAGCATGTGGTTTAATTCGATGCAACGCGAAGAACCTTACCTGG
CCTTGACATACAGAGAACTTTCCAGAGATGGATTGGTGCCTTCGGGAACTCTGATACAGGTGCTGCATGG
CTGTCGTCAGCTCGTGTCGTGAGATGTTGGGTTAAGTCCCGCAACGAGCGCAACCCTTTTCCTTATTTGC
CAGCACTTCGGGTGGGAACTTTAAGGATACTGCCAGTGACAAACTGGAGGAAGGCGGGGACGACGTCAAG
TCATCATGGCCCTTACGGCCAGGGCTACACACGTGCTACAATGGTCGGTACAAAGGGTTGCTACACAGCG
ATGTGATGCTAATCTCAAAAAGCCGATCGTAGTCCGGATTGGAGTCTGCAACTCGACTCCATGAAGTCGG
AATCGCTAGTAATCGCGGATCAGAATGCCGCGGTGAATACGTTCCCGGGCCTTGTACACACCGCCCGTCA
CACCATGGGAGTTTGTTGCACCAGAAGTAGGTAGTCTAACCGCAAGGAGGACGCTTACCACGGTGTGGCC
GATGACTGGGGTGAA
>66878618|gb|AY958912.1|Acinetobacter|Uncultured bacterium clone rRNA139
16S ribosomal RNA gene, partial sequence
CCTTAGAGTTTGATCCTGGCTCAGATTGAACGCTGGCGGCAGGCTTAACACATGCAAGTCGAGCGGGGGA
GGTTGCTTCGGTAACTGACCTAGCGGCGGACGGGTGAGTAATACTTAGGAATCTGCCTATTAATGGGGGA
CAACATCTCGAAAGGGATGCTAATACCGCATACGCCCTACGGGGGAAAGCAGGGGATCACTTGTGACCTT
GCGTTAATAGATGAGCCTAAGTCGGATTAGCTAGTTGGTGGGGTAAAGGCCTACCAAGGCGACGATCTGT
AGCGGGTCTGAGAGGATGATCCGCCACACTGGGACTGAGACACGGCCCAGACTCCTACGGGAGGCAGCAG
TGGGGAATATTGGACAATGGGGGGAACCCTGATCCAGCCATGCCGCGTGTGTGAAGAAGGCCTTATGGTT
GTAAAGCACTTTAAGCGAGGAGGAGGCTCTCTTGGTTAATACCCAAGATGAGTGGACGTTACTCGCAGAA
TAAGCACCGGCTAACTCTGTGCCAGCAGCCGCGGTAATACAGAGGGTGCGAGCGTTAATCGGATTTACTG
GGCGTAAAGCGTGCGTAGGCGGCTTTTTAAGTCGGATGTGAAATCCCCGAGCTTAACTTGGGAATTGCAT
TCGATACTGGGAAGCTAGAGTATGGGAGAGGATGGTAGAATTCCAGGTGTAGCGGTGAAATGCGTAGAGA

TCTGGAGGAATACCGATGGCGAAGGCAGCCATCTGGCCTAATACTGACGCTGAGGTACGAAAGCATGGGG
AGCAAACAGGATTAGATACCCTGGTAGTCCATGCCGTAAACGATGTCTACTAGCCGTTGGGGCCTTTGAG
GCTTTAGTGGCGCAGCTAACGCGATAAGTAGACCGCCTGGGGAGTACGGTCGCAAGACTAAAACTCAAAT
GAATTGACGGGGGCCCGCACAAGCGGTGGAGCATGTGGTTTAATTCGATGCAACGCGAAGAACCTTACCT
GGTCTTGACATAGTAAGAACTTTCCAGAGATGGATTGGTGCCTTCGGGAACTTACATACAGGTGCTGCAT
GGCTGTCGTCAGCTCGTGTCGTGAGATGTTGGGTTAAGTCCCGCAACGAGCGCAACCCTTTTCCTTATTT
GCCAGCACTTCGGGTGGGAACTTTAAGGATACTGCCAGTGACAAACTGGAGGAAGGCGGGGACGACGTCA
AGTCATCATGGCCCTTACGACCAGGGCTACACACGTGCTACAATGGTCGGTACAAAGGGTTGCTACCTAG
CGATAGGATGCTAATCTCAAAAAGCCGATCGTAGTCCGGATTGGAGTCTGCAACTCGACTCCATGAAGAC
GGAATCGCTAGTAATCGCGGATCAGAATGCCGCGGTGAATACGTTCCCGGGCCTTGTACACACCGCCCGT
CACACCATGGGAGTTTGTTGCACCAGAAGTAGGTAGTCTAACCGTAAGGAGGACGCTTACCACGGTGTGG
CCGATGACTGGGGTGAAGTCGTAACAAGGTAACCGTAAAGGG
>239923994|gb|GQ179716.1|Acinetobacter|Uncultured Acinetobacter sp. clone VE12D01 16S ribosomal RNA gene, partial sequence
ATTGAACGCTGGCGGCAGGCTTAACACATGCAAGTCGAGCGGGGGAGATTGCTTCGGTAATTGACCTAGC
GGCGGACGGGTGAGTAATACTTAGGAATCTGCCTATTAATGGGGGACAACATCTCGAAAGGGATGCTAAT
ACCGCATACGCCCTACGGGGGAAAGCAGGGGATCACTTGTGACCTTGCGTTAATAGATGAGCCTAAGTCG
GATTAGCTAGTTGGTGGGGTAAAGGCCTACCAAGGCGACGATCTGTAGCGGGTCTGAGAGGATGATCCGC
CACACTGGGACTGAGACACGGCCCAGACTCCTACGGGAGGCAGCAGTGGGGAATATTGGACAATGGGGGG
AACCCTGATCCAGCCATGCCGCGTGTGTGAAGAAGGCCTTATGGTTGTAAAGCACTTTAAGCGAGGAGGA
GGCTCCTGTAGTTAATACCTACAGAGAGTGGACGTTACTCGCAGAATAAGCACCGGCTAACTCTGTGCCA
GCAGCCGCGGTAATACAGAGGGTGCGAGCGTTAATCGGATTTACTGGGCGTAAAGCGTGCGTAGGCGGCT
TTTTAAGTCGGATGTGAAATCCCCGAGCTTAACTTGGGAATTGCATTCGATACTGGGAAGCTAGAGTATG
GGAGAGGATGGTAGAATTCCAGGTGTAGCGGTGAAATGCGTAGAGATCCGGAGGAATACCGATGGCGAAG
GCAGCCATCTGGCCTAATACTGACGCTGAGGTACGAAAGCATGGGGAGCAAACAGGATTAGATACCCTGG
TAGTCCATGCCGTAAACGATGTCTACTAGCCGTTGGGGCCTTTGAGGCTTTAGTGGCGCAGCTAACGCGA
TAAGTAGACCGCCTGGGGAGTACGGTCGCAAGACTAAAACTCAAATGAATTGACGGGGGCCCGCACAAGC
GGTGGAGCATGTGGTTTAATTCGATGCAACGCGAAGAACCTTACCTGGTCTTGACATAGTAAGAACTTTC
CAGAGATGGATTGGTGCCTTCGGGAACTTACATACAGGTGCTGCATGGCTGTCGTCAGCTCGTGTCGTGA
GATGTTGGGTTAAGTCCCGCAACGAGCGCAACCCTTTTCCTTATTTGCCAGCACTTCGGGTGGGAACTTT
AAGGATACTGCCAGTGACAAACTGGAGGAAGGCGGGGACGACGTCAAGTCATCATGGCCCTTACGACCAG
GGCTACACACGTGCTACAATGGTCGGTACAAAGGGTTGCTACCTAGCGATAGGATGCTAATCTCAAAAAG
CCGATCGTAGTCCGGATTGGAGTCTGCAACTCGACTCCATGAAGTCGGAATCGCTAGTAATCGCGGATCA
GAATGCCGCGGTGAATACGTTCCCGGGCCTTGTACACACCGCCCGTCACACCATGGGAGTTTGTTGCACC
AGAAGTAGGTAGTCTAACCGTAAGGAGGACGCTTACCACGGTGTGGCCGATGACTGGGGTGAAGTCGTAA
CAAGGTA
>154240524|dbj|AB334528.1|Pseudomonas|Pseudomonas sp. MPU L18 gene for 16S ribosomal RNA, partial sequence
AGAGTTTGATCCTGGCTCAGATTGAACGCTGGCGGCAGGCCTAACACATGCAAGTCGAGCGGTAGAGAGA
AGCTTGCTTCTCTTGAGAGCGGCGGACGGGTGAGTAATGCCTAGGAATCTGCCTGGTAGTGGGGGATAAC
GTTCGGAAACGGACGCTAATACCGCATACGTCCTACGGGAGAAAGCAGGGGACCTTCGGGCCTTGCGCTA
TCAGATGAGCCTAGGTCGGATTAGCTAGTTGGTGAGGTAATGGCTCACCAAGGCGACGATCCGTAACTGG
TCTGAGAGGATGATCAGTCACACTGGAACTGAGACACGGTCCAGACTCCTACGGGAGGCAGCAGTGGGGA
ATATTGGACAATGGGCGAAAGCCTGATCCAGCCATGCCGCGTGTGTGAAGAAGGTCTTCGGATTGTAAAG
CACTTTAAGTTGGGAGGAAGGGCAGTAAATTAATACTTTGCTGTTTTGACGTTACCGACAGAATAAGCAC
CGGCTAACTCTGTGCCAGCAGCCGCGGTAATACAGAGGGTGCAAGCGTTAATCGGAATTACTGGGCGTAA
AGCGCGCGTAGGTGGTTTGTTAAGTTGGATGTGAAATCCCCGGGCTCAACCTGGGAACTGCATTCAAAAC
TGACTGACTAGAGTATGGTAGAGGGTGGTGGAATTTCCTGTGTAGCGGTGAAATGCGTAGATATAGGAAG
GAACACCAGTGGCGAAGGCGACCACCTGGACTAATACTGACACTGAGGTGCGAAAGCGTGGGGAGCAAAC
AGGATTAGATACCCTGGTAGTCCACGCCGTAAACGATGTCAACTAGCCGTTGGAAGCCTTGAGCTTTTAG
TGGCGCAGCTAACGCATTAAGTTGACCGCCTGGGGAGTACGGCCGCAAGGTTAAAACTCAAATGAATTGA
CGGGGGCCCGCACAAGCGGTGGAGCATGTGGTTTAATTCGAAGCAACGCGAAGAACCTTACCAGGCCTTG
ACATCCAATGAACTTTCTAGAGATAGATTGGTGCCTTCGGGAACATTGAGACAGGTGCTGCATGGCTGTC
GTCAGCTCGTGTCGTGAGATGTTGGGTTAAGTCCCGTAACGAGCGCAACCCTTGTCCTTAGTTACCAGCA

CGTCATGGTGGGCACTCTAAGGAGACTGCCGGTGACAAACCGGAGGAAGGTGGGGATGACGTCAAGTCAT
CATGGCCCTTACGGCCTGGGCTACACACGTGCTACAATGGTCGGTACAGAGGGTTGCCAAGCCGCGAGGT
GGAGCTAATCCCANAAAACCGATCGTAGTCCGGATCGCAGTCTGCAACTCGACTGCGTGAAGTCGGAATC
GCTAGTAATCGCGAATCAGAATGTCGCGGTGAATACGTTCCCGGGCCTTGTACACACCGCCCGTCACACC
ATGGGAGTGGGTTGCACCAGAAGTAGCTAGTCTAACCTTCGGGAGGACGGTTACCACGGTGTGATTCATG
ACTGGGGTGAAGTCGTAACAAGGTAACC
>66878583|gb|AY958877.1|Pseudomonas|Uncultured bacterium clone rRNA104
16S ribosomal RNA gene, partial sequence
CCTTAGAGTTTGATCCTGGCTCAGATTGAACGCTGGCGGCAGGCCTAACACATGCGAGTCGAGCGGTAGA
GAGGAGCTTGCTCCTCTTGAGAGCGGCGGACGGGTGAGTAATGCCTAGGAATCTGCCTGGTAGTGGGGGA
TAACGTTCGGAAACGGACGCTAATACCGCATACGTCCTACGGGAGAAAGCAGGGGACCTTCGGGCCTTGC
GCTATCAGATGAGCCTAGGTCGGATTAGCTAGTTGGTGAGGTAATGGCTCACCAAGGCGACGATCCGTAA
CTGGTCTGAGAGGATGATCAGTCACACTGGAACTGAGACACGGTCCAGACTCCTACGGGAGGCAGCAGTG
GGGAATATTGGACAATGGGCGAAAGCCTGATCCAGCCATGCCGCGTGTGTGAAGAAGGTCTTCGGATTGT
AAAGCACTTTAAGTTGGGAGGAAGGGCAGTAAATTAATACTTTGCTGTTTTGACGTTACCGACAGAATAA
GCACCGGCTAACTCTGTGCCAGCAGCCGCGGTAATACAGAGGGTGCAAGCGTTAATCGGAATTACTGGGC
GTAAAGCGCGCGTAGGTGGTTTGTTAAGTTGGATGTGAAATCCCCGGGCTCAACCTGGGAACTGCATTCA
AAACTGACTGACTAGAGTATGGTAGAGGGTGGTGGAATTTCCTGTGTAGCGGTGAAATGCGTAGATATAG
GAAGGAACACCAGTGGCGAAGGCGACCACCTGGACTAATACTGACACTGAGGTGCGAAAGCGTGGGGAGC
AAACAGGATTAGATACCCTGGTAGTCCACGCCGTAAACGATGTCAACTAGCCGTTGGAAGCCTTGAGCTT
TTAGTGGCGCAGCTAACGCATTAAGTTGACCGCCTGGGGAGTACGGCCGCAAGGTTAAAACTCAAATGAA
TTGACGGGGGCCCGCACAAGCGGTGGAGCATGTGGTTTAATTCGAAGCAACGCGAAGAACCTTACCAGGC
CTTGACATCCAATGAACTTTCTAGAGATAGATTGGTGCCTTCGGGAACATTGAGACAGGTGCTGCATGGC
TGTCGTCAGCTCGTGTCGTGAGATGTTGGGTTAAGTCCCGTAACGAGCGCAACCCTTGTCCTTAGTTACC
AGCACGTCATGGTGGGCACTCTAAGGAGACTGCCGGTGACAAACCGGAGGAAGGTGGGGATGACGTCAAG
TCATCATGGCCCTTACGGCCTGGGCTACACACGTGCTACAATGGTCGGTACAGAGGGTTGCCAAGCCGCG
AGGTGGAGCTAATCCCACAAAACCGATCGTAGTCCGGATCGCAGTCTGCAACTCGACTGCGTGAAGTCGG
AATCGCTAGTAATCGCGAATCAGAATGTCGCGGTGAATACGTTCCCGGGCCTTGTACACACCGCCCGTCA
CACCATGGGAGTGGGTTGCACCAGAAGTAGCTAGTCTAACCTTCGGGAGGACGGTTACCACGGTGTGATT
CATGACTGGGGTGAAGTCGTAACAAGGTAGCCGTAAAGGGCGA
>239923999|gb|GQ179721.1|Pseudomonas|Uncultured Pseudomonas sp. clone
VE51B10 16S ribosomal RNA gene, partial sequence
ATTGAACGCTGGCGGCAGGCCTAACACATGCAAGTCGAGCGGTAGAGAGGTGCTTGCACCTCTTGAGAGC
GGCGGACGGGTGAGTAATACCTAGGAATCTGCCTGGTAGTGGGGGATAACGTTCGGAAACGGACGCTAAT
ACCGCATACGTCCTACGGGAGAAAGCAGGGGACCTTCGGGCCTTGCGCTATCAGATGAGCCTAGGTCGGA
TTAGCTAGTTGGTGAGGTAATGGCTCACCAAGGCTACGATCCGTAACTGGTCTGAGAGGATGATCAGTCA
CACTGGAACTGAGACACGGTCCAGACTCCTACGGGAGGCAGCAGTGGGGAATATTGGACAATGGGCGAAA
GCCTGATCCAGCCATGCCGCGTGTGTGAAGAAGGTCTTCGGATTGTAAAGCACTTTAAGTTGGGAGGAAG
GGCATTAACCTAATACGTTAGTGTTTTGACGTTACCGACAGAATAAGCACCGGCTAACTCTGTGCCAGCA
GCCGCGGTAATACAGAGGGTGCAAGCGTTAATCGGAATTACTGGGCGTAAAGCGCGCGTAGGTGGTTTGT
TAAGTTGAATGTGAAATCCCCGGGCTCAACCTGGGAACTGCATCCAAAACTGGCAAGCTAGAGTATGGTA
GAGGGTAGTGGAATCTCCTGTGTAGCGGTGAAATGCGTAGATATAGGAAGGAACACCAGTGGCGAAGGCG
ACTACCTGGACTGATACTGACACTGAGGTGCGAAAGCGTGGGGAGCAAACAGGATTAGATACCCTGGTAG
TCCACGCCGTAAACGATGTCAACTAGCCGTTGGGAACCTTGAGTTCTTAGTGGCGCAGCTAACGCATTAA
GTTGACCGCCTGGGGAGTACGGCCGCAAGGTTAAAACTCAAATGAATTGACGGGGGCCCGCACAAGCGGT
GGAGCATGTGGTTTAATTCGAAGCAACGCGAAGAACCTTACCAGGCCTTGACATCCAATGAACTTTCCAG
AGATGGATTGGTGCCTTCGGGAACATTGAGACAGGTGCTGCATGGCTGTCGTCAGCTCGTGTCGTGAGAT
GTTGGGTTAAGTCCCGTAACGAGCGCAACCCTTGTCCTTAGTTACCAGCACGTAATGGTGGGCACTCTAA
GGAGACTGCCGGTGACAAACCGGAGGAAGGTGGGGATGACGTCAAGTCATCATGGCCCTTACGGCCTGGG
CTACACACGTGCTACAATGGTCGGTACAAAGGGTTGCCAAGCCGCGAGGTGGAGCTAATCCCATAAAACC
GATCGTAGTCCGGATCGCAGTCTGCAACTCGACTGCGTGAAGTCGGAATCGCTAGTAATCGTGAATCAGA
ATGTCACGGTGAATACGTTCCCGGGCCTTGTACACACCGCCCGTCACACCATGGGAGTGGGTTGCACCAG
AAGTAGCTAGTCTAACCTTCGGGAGGACGGTTACCACGGTGTGATTCATGACTGGGGTGAAGTCGTAACA
AGGTA

>239923990|gb|GQ179712.1|Stenotrophomonas|Uncultured Stenotrophomonas sp. clone VE12C08 16S ribosomal RNA gene, partial sequence
AGTGAACGCTGGCGGTAGGCCTAACACATGCAAGTCGAACGGCAGCACAGTAAGAGCTTGCTCTTACGGG
TGGCGAGTGGCGGACGGGTGAGGAATACATCGGAATCTACTTTTTCGTGGGGGATAACGTAGGGAAACTT
ACGCTAATACCGCATACGACCTACGGGTGAAAGCAGGGGATCTTCGGACCTTGCGCGATTGAATGAGCCG
ATGTCGGATTAGCTAGTTGGCGGGGTAAAGGCCCACCAAGGCGACGATCCGTAGCTGGTCTGAGAGGATG
ATCAGCCACACTGGAACTGAGACACGGTCCAGACTCCTACGGGAGGCAGCAGTGGGGAATACTGGACAAT
GGGCGCAAGCCTGATCCAGCCATACCGCGTGGGTGAAGAAGGCCTTCGGGTTGTAAAGCCCTTTTGTTGG
GAAAGAAATCCAGCCGGCTAATACCTGGTTGGGATGACGGTACCCAAAGAATAAGCACCGGCTAACTTCG
TGCCAGCAGCCGCGGTAATACGAAGGGTGCAAGCGTTACTCGGAATCACTGGGCGTAAAGCGTGCGTAGG
TGGTCGTTTAAGTCTGTTGTGAAAGCCCTGGGCTCAACCTGGGAACTGCAGTGGAAACTGGACGACTAGA
GTGTGGTAGAGGGTAGCGGAATTCCTGGTGTAGCAGTGAAATGCGTAGAGATCAGGAGGAACATCCATGG
CGAAGGCAGCTACCTGGACCAACACTGACACTGAGGCACGAAAGCGTGGGGAGCAAACAGGATTAGATAC
CCTGGTAGTCCACGCCCTAAACGATGCGAACTGGATGTTGGGTGCAATTTGGCACGCAGTATCGAAGCTA
ACGCGTTAAGTTCGCCGCCTGGGGAGTACGGTCGCAAGACTGAAACTCAAAGGAATTGACGGGGGCCCGC
ACAAGCGGTGGAGTATGTGGTTTAATTCGATGCAACGCGAAGAGCCTTACCTGGCCTTGACATGTCGAGA
ACTTTCCAGAGATGGATTGGTGCCTTCGGGAACTCGAACACAGGTGCTGCATGGCTGTCGTCAGCTCGTG
TCGTGAGATGTTGGGTTAAGTCCCGCAACGAGCGCAACCCTTGTCCTTAGTTGCCAGCACGTAATGGTGG
GAACTCTAAGGAGACCGCCGGTGACAAACCGGAGGAAGGTGGGGATGACGTCAAGTCATCATGGCCCTTA
CGGCCAGGGCTACACACGTACTACAATGGTAGGGACAGAGGGCTGCAAGCCGGCGACGGTAAGCCAATCC
CAGAAACCCTATCTCAGTCCGGATTGGAGTCTGCAACTCGACTCCATGAAGTCGGAATCGCTAGTAATCG
CAGATCAGCATTGCTGCGGTGAATACGTTCCCGGGCCTTGTACACACCGCCCGTCACACCATGGGAGTTT
GTTGCACCAGAAGCAGGTAGCTTAACCTTCGGGAGGGCGCTTGCCACGGTGTGGCCGATGACTGGGGTGA
AGTCGTAACAAGGTA
>2832587|emb|AJ002814.1|Stenotrophomonas|Stenotrophomonas sp. 16S rRNA gene, isolate S3
AGTGAACGCTGGCGGTAGGCCTAACACATGCAAGTCGAACGGCAGCACAGTAAGAGCTTGCTCTTACGGG
TGGCGAGTGGCGGACGGGTGAGGAATACATCGGAATCTACTTTTTCGTGGGGGATAACGTAGGGAAACTT
ACGCTAATACCGCATACGACCTACGGGTGAAAGCAGGGGATCTTCGGACCTTGCGCGATTGAATGAGCCG
ATGTCGGATTAGCTAGTTGGCGGGGTAAAGGCCCACCAAGGCGACGATCCGTAGCTGGTCTGAGAGGATG
ATCAGCCACACTGGAACTGAGACACGGTCCAGACTCCTACGGGAGGCAGCAGTGGGGAATATTGGACAAT
GGGCGCAAGCCTGATCCAGCCATACCGCGTGGGTGAAGAAGGCCTTCGGGTTGTAAAGCCCTTTTGTTGG
GAAAGAAATCCAGCTGGTTAATACCCGGTTGGGATGACGGTACCCAAAGAATAAGCACCGGCTAACTTCG
TGCCAGCAGCCGCGGTAATACGAAGGGTGCAAGCGTTACTCGGAATTACTGGGCGTAAAGCGTGCGTAGG
TGGTCGTTTAAGTCTGTTGTGAAAGCCCTGGGCTCAACCTGGGAACTGCAGTGGAAACTGGACGACTAGA
GTGTGGTAGAGGGTAGCGGAATTCCTGGTGTAGCAGTGAAATGCGTAGAGATCAGGAGGAACATCCATGG
CGAAGGCAGCTACCTGGACCAACACTGACACTGAGGCACGAAAGCGTGGGGAGCAAACAGGATTAGATAC
CCTGGTAGTCCACGCCCTAAACGATGCGAACTGGATGTTGGGTGCAATTTGGCACGCAGTATCGAAGCTA
ACGCGTTAAGTTCGCCGCCTGGGGAGTACGGTCGCAAGACTGAAACTCAAAGGAATTGACGGGGGCCCGC
ACAAGCGGTGGAGTATGTGGTTTAATTCGATGCAACGCGAAGAACCTTACCTGGCCTTGACATGTCGAGA
ACTTTCCAGAGATGGATTGGTGCCTTCGGGAACTCGAACACAGGTGCTGCATGGCTGTCGTCAGCTCGTG
TCGTGAGATGTTGGGTTAAGTCCCGCAACGAGCGCAACCCTTGTCCTTAGTTGCCAGCACGTAATGGTGG
GAACTCTAAGGAGACCGCCGGTGACAAACCGGAGGAAGGTGGGGATGACGTCAAGTCATCATGGCCCTTA
CGGCCAGGGCTACACACGTACTACAATGGTAGGGACAGAGGGCTGCAAGCCGGCGACGGTAAGCCAATCC
CAGAAACCCTATCTCAGTCCGGATTGGAGTCTGCAACTCGACTCCATGAAGTCGGAATCGCTAGTAATCG
CAGATCAGCATTGCTGCGGTGAATACGTTCCCGGGCCTTGTACACACCGCCCGTCACACCATGGGAGTTT
GTTGCACCAGAAGCAGGTAGCTTAACCTTCGGGAGGGCGCTTGCCACGGTGTGGCCGATGACTGGGGTGA
AGTCGTAACAAGGTAGCCGTATCGGAAGGTGC
>66878829|gb|AY959123.1|Stenotrophomonas|Uncultured bacterium clone rRNA350 16S ribosomal RNA gene, partial sequence
CTTAGAGTTTGATCCTGGCTCAGAGTGAACGCTGGCGGTAGGCCTAACACATGCAAGTCGAACGGCAGCA
CAGGAGAGCTTGCTCTCTGGGTGGCGAGTGGCGGACGGGTGAGGAATACATCGGAATCTACTTTTTCGTG
GGGGATAACGTAGGGAAACTTACGCTAATACCGCATACGACCTACGGGTGAAAGCAGGGGATCTTCGGAC
CTTGCGCGATTGAATGAGCCGATGTCGGATTAGCTAGTTGGCGGGGTAAAGGCCCACCAAGGCGACGATC

CGTAGCTGGTCTGAGAGGATGATCAGCCACACTGGAACTGAGACACGGTCCAGACTCCTACGGGAGGCAG
CAGTGGGGAATATTGGACAATGGGCGCAAGCCTGATCCAGCCATACCGCGTGGGTTAAGAAGGCCTTCGG
GTTGTAAAGCCCTTTTGTTGGGAAAGAAATCCAGCTGGCTAATACCCGGTTGGGATGACGGTACCCAAAG
AATAAGCACCGGCTAACTTCGTGCCAGCAGCCGCGGTAATACGAAGGGTGCAAGCGTTACTCGGAATTAC
TGGGCGTAAAGCGTGCGTAGGTGGTCGTTTAAGTCCGTTGTGAAAGCCCTGGGCTCAACCTGGGAACTGC
AGTGGATACTGGGCGACTAGAGTGTGGTAGAGGGTAGCGGAATTCCTGGTGTAGCAGTGAAATGCGTAGA
GATCAGGAGGAACATCCATGGCGAAGGCAGCTACCTGGACCAACACTGACACTGAGGCACGAAAGCGTGG
GGAGCAAACAGGATTAGATACCCTGGTAGTCCACGCCCTAAACGATGCGAACTGGATGTTGGGTGCAATT
TGGCACGCAGTATCGAAGCTAACGCGTTAAGTTCGCCGCCTGGGGAGTACGGTCGCAAGACTGAAACTCA
AAGGAATTGACGGGGGCCCGCACAAGCGGTGGAGTATGTGGTTTAATTCGATGCAACGCGAAGAACCTTA
CCTGGCCTTGACATGTCGAGAACTTTCCAGAGATGGATTGGTGCCTTCGGGAACTCGAACACAGGTGCTG
CATGGCTGTCGTCAGCTCGTGTCGTGAGATGTTGGGTTAAGTCCCGCAACGAGCGCAACCCTTGTCCTTA
GTTGCCAGCACGTAATGGTGGGAACTCTAAGGAGACCGCCGGTGACAAACCGGAGGAAGGTGGGGATGAC
GTCAAGTCATCATGGCCCTTACGGCCAGGGCTACACACGTACTACAATGGTAGGGACAGAGGGCTGCAAG
CCGGCGACGGTAAGCCAATCCCAGAAACCCTATCTCAGTCCGGATTGGAGTCTGCAACTCGACTCCATGA
AGTCGGAATCGCTAGTAATCGCAGATCAGCATTGCTGCGGTGAATACGTTCCCGGGCCTTGTACACACCG
CCCGTCACACCATGGGAGTTTGTTGCACCAGAAGCAGGTAGCTTAACCTTCGGGAGGGCGCTTGCCACGG
TGTGGCCGATGACTGGGGTGAAGTCGTAACAAGGTAGCCGTAAAGGGCGA
>52222164|gb|AY738675.1|Prevotella|Uncultured Prevotella sp. clone 127-Q
23 16S ribosomal RNA gene, partial sequence
TGAGGAATATTGGTCAATGGACGCAAGTCTGAACCAGCCAAGTAGCGTGCAGGATGACGGCCCTATGGGT
TGTAAACTGCTTTTATATGGGGATAAAGTGGGGAACGTGTTCCCTTTTGCAGGTACCATATGAATAAGGA
CCGGCTAATTCCGTGCCAGCAGCCGCGGTAATACGGAAGGTTCGGGCGTTATCCGGATTTATTGGGTTTA
AAGGGAGCGTAGGCCGTTTGGTAAGCGTGTTGTGAAATGTAGGAGCTCAACTTCTAGATTGCAGCGCGAA
CTGTCAGACTTGAGTGCGCACAACGTAGGCGGAATTCATGGTGTAGCGGTGAAATGCTTAGATATCATGA
AGAACTCCGATTGCGAAGGCAGCTTACGGGAGCGCAACTGACGCTGAAGCTCGAAGGTGCGGGTATCGAA
CAGGATTAGATACCCTGGTAGTCCGCACAGTAAACGATGGATGCCCGCTGTTAGCACCTAGTGTTAGCGG
CTAAGCGAAAGCATTAAGCATCCCACCTGGGGAGTACGCCGGCAACGGTGAAACTCAAAGGAATTGACGG
GGGCCCGCACAAGCGGAGGAACATGTGGTTTAATTCGATGATACGCGAGGAACCTTACCCGGGCTTGAAT
TGCAGATGAACGATTTAGAGATAATGAGGTCCTTCGGGACATCTGTGAAGGTGCTGCATGGTTGTCGTCA
GCTCGTGCCGTGAGGTGTCGGCTTAAGTGCCATAACGAGCGCAACCCCTTTCTTTAGTTGCCATCAGGTC
ATGCTGGGCACTCTGGAGATACTGCCACCGTAAGGTGTGAGGAAGGTGGGGATGACGTCAAATCAGCACG
GCCCTTACGTCCGGGGCTACACACGTGTTACAATGGGTGGTACAGATAGTTGGTCGTGTGCAAATACGAT
CTAATCCTTAAAACCATTCTCAGTTCGGACTGGGGTCTGCAACCCGACCCCACGAAGCTGGATTCGCTAG
TAATCGCGCATCAGCCATGGCGCGGTGAATACGTTCCCGGCCT
>343198448|ref|NR_041881.1|M.hominis|M.hominis ATCC 23114 strain PG21;
ATCC 23114 16S ribosomal RNA, partial sequence
TTTTATAAGAGTTTGATCCTGGCTCAGGATGAACGCTGGCTGTGTGCCTAATACATGCATGTCGAGCGAG
GTTAGCAATAACCTAGCGGCGAATGGGTGAGTAACACGTGCTTAATCTACCTTTTAGATTGGAATACCCA
TTGGAAACAATGGCTAATGCCGGATACGCATGGAACCGCATGGTTCCGTTGTGAAAGGCGCTGTAAGGCG
CCACTAAAAGATGAGGGTGCGGAACATTAGTTAGTTGGTGAGGTAATGGCCCACCAAGACTATGATGTTT
AGCCGGGTCGAGAGACTGAACGGCCACATTGGGACTGAGATACGGCCCAAACTCCTACGGGAGGCAGCAG
TAGGGAATATTCCACAATGAGCGAAAGCTTGATGGAGCGACACAGCGTGCACGATGAAGGTCTTCGGATT
GTAAAGTGCTGTTATAAGGGAAGAACATTTGCAATAGGAAATGATTGCAGACTGACGGTACCTTGTCAGA
AAGCGATGGCTAACTATGTGCCAGCAGCCGCGGTAATACATAGGTCGCAAGCGTTATCCGGAATTATTGG
GCGTAAAGCGTTCGTAGGCTGTTTGTTAAGTCTGGAGTTAAATCCCGGGGCTCAACCCCGGCTCGCTTTG
GATACTAGCAAACTAGAGTTAGATAGAGGTAAGCGGAATTCCATGTGAAGCGGTGAAATGCGTAGATATA
TGGAAGAACACCAAAGGCGAAGGCAGCTTACTGGGTCTATACTGACGCTGAGGGACGAAAGCGTGGGGAG
CAAACAGGATTAGATACCCTGGTAGTCCACGCCGTAAACGATGATCATTAGTCGGTGGAGAATCACTGAC
GCAGCTAACGCATTAAATGATCCGCCTGAGTAGTATGCTCGCAAGAGTGAAACTTAAAGGAATTGACGGG
GACCCGCACAAGCGGTGGAGCATGTGGTTTAATTTGAAGATACACGGAAAACCTTACCCACTCTTGACAT
CCTTCGCAAAGCTATAGAGATATAGTGGAGGTTATCGGAGTGACAGATGGTGCATGGTTGTCGTCAGCTC
GTGTCGTGAGATGTTTGGTCAAGTCCTGCAACGAGCGCAACCCCTATCTTTAGTTACTAACATTAAGTTG
AGGACTCTAGAGATACTGCCTGGGTAACTGGGAGGAAGGTGGGGATGACGTCAAATCATCATGCCTCTTA

CGAGTGGGGCCACACACGTGCTACAATGGTCGGTACAAAGAGAAGCAATATGGCGACATGGAGCAAATCT
CAAAAAGCCGATCTCAGTTCGGATTGGAGTCTGCAATTCGACTCCATGAAGTCGGAATCGCTAGTAATCG
CAGATCAGCTATGCTGCGGTGAATACGTTCTCGGGTCTTGTACACACCGCCCGTCACACCATGGGAGCTG
GTAATACCCAAAGTCGGTTTGCTAACCTCGGAGGCGACCGCCTAAGGTAGGACTGGTGACTGGGGTGAAG
TCGTAACAAGGTATCCCTACGAGAACGTGGGGATGGATCACCTCCTTT
>150134|gb|M96660.1|M.hominis|M.hominis 16S ribosomal RNA gene sequence
AGAGTTTGATCCTGGCTCAGGATGAACGCTGGCTGTGTGCCTAATACATGCATGTCGAGCGAGGTTAGCA
ATAACCTAGCGGCGAATGGGTGAGTAACACGTGCTTAATCTACCTTTTAGATTGGAATACCCATTGGAAA
CAATGGCTAATGCCGGATACGCATGGAACCGCATGGTTCCGTTGTGAAAGGCGCTGTAAGGCGCCACTAA
AAGATGAGGGTGCGGAACATTAGTTAGTTGGTGAGGTAATGGCCCACCAAGACTATGATGTTTAGCCGGG
TCGAGAGACTGAACGGCCACATTGGGACTGAGATACGGCCCAAACTCCTACGGGAGGCAGCAGTAGGGAA
TATTCCACAATGAGCGAAAGCTTGATGGAGCGACACAGCGTGCACGATGAAGGTCTTCGGATTGTAAAGT
GCTGTTATAAGGGAAGAACATTTGCAATAGGAAATGATTGCAGACTGACGGTACCTTGTCAGAAAGCGAT
GGCTAACTATGTGCCAGCAGCCGCGGTAATACATAGGTCGCAAGCGTTATCCGGAATTATTGGGCGTAAA
GCGTTCGTAGGCTGTTTGTTAAGTCTGGAGTTAAATCCCGGGGCTCAACCCCGCTCGCTTTGGATACTAG
CAAACTAGAGTTAGATAGAGGTAAGCGGAATTCCATGTGAAGCGGTGAAATGCGTAGATATATGGAAGAA
CACCAAAGGCGAAGGCAGCTTACTGGGTCTATACTGACGCTGGGACGAAAGCGTGGGGAGCAAACAGGAT
TAGATACCCTGGTAGTCCACGCCGTAAACGATGATCATTAGTCGGTGGAGAATCACTGACGCAGCTAACG
CATTAAATGATCCGCCTGAGTAGTATGCTCGCAAGAGTGAAACTTAAAGGAATTGACGGGNNACGCACAA
GNGGAGCATGTGGTTTAATTTGAAGATACACGGAAAACCTTACCCACTCTTGACATCCTTCGCAAAGCTA
TAGAGATATAGTGGAGGTTATCGGAGTGACAGATGGTGCATGGTTGTCGTCAGCTCGTGTCGTGAGATGT
TTGGTCAAGTCCTGCAACGAGCGCAACCCCTATCTTTAGTTACTAACATTAAGTTGAGGACTCTAGAGAT
ACTGCCTGGGTAACTGGGAGGAAGGTGGGGATGACGTCAAATCATCATGCCTCTTACGAGTGGGGCCACA
CACGTGCTACAATGGTCGGTACAAAGAGAAGCAATATGGCGACACTGAGCAAATCTCAAAAAGCCGATCT
CAGTTCGGATTGGAGTCTGCAATTCGACTCCATGAAGTCGGAATCGCTAGTAATCGCAGATCAGCTATGC
TGCGGTGAATACGTTCTCGGGTCTTGTACACACCGCCCGTCACACCATGGGAGCTGGTAATACCCAAAGT
CGGTTTGCTAACCTCGGAGGCGACCGCCTAAGGTAGGACTGGTGACTGGG
>39653271|gb|AY466443.1|M.genitalium|strain M2288 16S ribosomal RNA gene, partial sequence
TGAGAGTTTGATCCTGGCTCAGGATTAACGCTGGCGGCATGCCTAATACATGCAAGTCGATCGGAAGTAG
CAATACTTTAGAGGCGAACGGGTGAGTAACACGTATCCAATCTACCTTATAATGGGGGATAACTAGTTGA
AAAACTAGCTAATACCGCATAAGAACTTTAGTTCGCATGAATTAAAGTTGAAAGGACCTGCAAGGGTTCG
TTATTTGATGAGGGTGCGCCATATCAGCTAGTTGGTAGGGTAATGGCCTACCAAGGCAATGACGTGTAGC
TATGCTGAGAAGTAGAATAGCCACAATGGGACTGAGACACGGCCCATACTCCTACGGGAGGCAGCAGTAG
GGAATTTTTCACAATGAGCGAAAGCTTGATGGAGCAATGCCGCGTGAACGATGAAGGTCTTTTTGATTGT
AAAGTTCTTTTATTTGGGAAGAATGACTCTAGCAGGCAATGGCTGGAGTTTGACTGTACCACTTTGAATA
AGTGACGACTAACTATGTGCCAGCAGTCGCGGTAATACATAGGTCGCAAGCGTTATCCGGATTTATTGGG
CGTAAAGCAAGCGCAGGCGGATTGAAAAGTCTGGTGTTAAAGGCAGCTGCTTAACAGTTGTATGCATTGG
AAACTATCAGTCTAGAGTGTGGTAGGGAGTTTTGGAATTTCATGTGGAGCGGTGAAATGCGTAGATATAT
GAAGGAACACCAGTGGCGAAGGCGAAAACTTAGGCCATTACTGACGCTTAGGCTTGAAAGTGTGGGGAGC
AAATAGGATTAGATACCCTAGTAGTCCACACCGTAAACGATAGATACTAGCTGTCGGAGCGATCCCTTCG
GTAGTGAAGTTAACACATTAAGTATCTCGCCTGGGTAGTACATTCGCAAGAATGAAACTCAAACGGAATT
GACGGGGACCCGCACAAGTGGTGGAGCATGTTGCTTAATTCGACGGTACACGAAAAACCTTACCTAGACT
TGACATCCTTGGCAAAGTTATGGAAACATAATGGAGGTTAACCGAGTGACAGGTGGTGCATGGTTGTCGT
CAGCTCGTGTCGTGAGATGTTGGGTTAAGTCCCGCAACGAGCGCAACCCTTATCGTTAGTTACATTGTTT
AACGAGACTGCTAATGTAAATTGGAGGAAGGAAGGGATGACGTCAAATCATCATGCCCCTTATGTCTAGG
GCTGCAAACGTGCTACAATGGCCAATACAAACAGTAGCCAACTTGTAAAAGTGAGCAAATCTGAAAAGTT
GGTCTCAGTTCGGATTGAGGGCTGCAATTCGTCCTCATGAAGCTGGAATCACTAGTAATCGCGAATCAGC
TATGTCGCGGTGAATACGTTCTCGGGTCTTGTACACACCGCCCGTCAAACTATGAAAGCTGGTAATATTT
AAAAACGTGTTGCTAACCTTTATTGGAAGTGCATGTCAAGGATAGCACCGGTGATTGGAGTTAAGTCGTA
ACAAGGTACCCCTACGAGAACG
>459531|emb|X77334.1|M.genitalium| 16S rRNA gene
AGAGTTTGATCCTGGCTCAGGATTAACGCTGGCGGCATGCCTAATACATGCAAGTCGATCGGAAGTAGCA
ATACTTTAGAGGCGAACGGGTGAGTAACACGTATCCAATCTACCTTATAATGGGGGATAACTAGTTGAAA

AACTAGCTAATACCGCATAAGAACTTTAGTTCGCATGAATTAAAGTTGAAAGGACCTGCAAGGGTTCGTT
ATTTGATGAGGGTGCGCCATATCAGCTAGTTGGTAGGGTAATGGCCTACCAAGGCAATGACGTGTAGCTA
TGCTGAGAAGTAGAATAGCCACAATGGGACTGAGACACGGCCCATACTCCTACGGGAGGCAGCAGTAGGG
AATTTTTCACAATGAGCGAAAGCTTGATGGAGCAATGCCGCGTGAACGATGAAGGTCTTTTTGATTGTAA
AGTTCTTTTATTTGGGAAGAATGACTCTAGCAGGCAATGGCTGGAGTTTGACTGTACCACTTTGAATAAG
TGACGACTAACTATGTGCCAGCAGTCGCGGTAATACATAGGTCGCAAGCGTTATCCGGATTTATTGGGCG
TAAAGCAAGCGCAGGCGGATTGAAAAGTCTGGTGTTAAAGGCAGCTGCTTAACAGTTGTATGCATTGGAA
ACTATCAGTCTAGAGTGTGGTAGGGAGTTTTGGAATTTCATGTGGAGCGGTGAAATGCGTAGATATATGA
AGGAACACCAGTGGCGAAGGCGAAAACTTAGGCCATTACTGACGCTTAGGCTTGAAAGTGTGGGGAGCAA
ATAGGATTAGATACCCTAGTAGTCCACACCGTAAACGATAGATACTAGCTGTCGGAGCGATCCCTTCGGT
AGTGAAGTTAACACATTAAGTATCTCGCCTGGGTAGTACATTCGCAAGAATGAAACTCAAACGGAATTGA
CGGGGACCCGCACAAGTGGTGGAGCATGTTGCTTAATTCGACGGTACACGAAAAACCTTACCTAGACTTG
ACATCCTTGGCAAAGTTATGGAAACATAATGGAGGTTAACCGAGTGACAGGTGGTGCATGGTTGTCGTCA
GCTCGTGTCGTGAGATGTTGGGTTAAGTCCCGCAACGAGCGCAACCCTTATCGTTAGTTACATTGTTTAA
CGAGACTGCTAATGTAAATTGGAGGAAGGAAGGGATGACGTCAAATCATCATGCCCCTTATGTCTAGGGC
TGCAAACGTGCTACAATGGCCAATACAAACAGTAGCCAACTTGTAAAAGTGAGCAAATCTGAAAAGTTGG
TCTCAGTTCGGATTGAGGGCTGCAATTCGTCCTCATGAAGCTGGAATCACTAGTAATCGCGAATCAGCTA
TGTCGCGGTGAATACGTTCTCGGGTCTTGTACACACCGCCCGTCAAACTATGAAAGCTGGTAATATTTAA
AAACGTGTTGCTAACCTTTATTGGAAGCGCATGTCAAGGATAGCACCGGTGATTGGAGTTAAGTCGTAAC
AAGGTACCCCTACGAGAACG
>66878766|gb|AY959060.1|Dialister|Uncultured bacterium clone rRNA287 16S ribosomal RNA gene, partial sequence
GCCCTTAGAGTTTGATCCTGGCTCAGGACGAACGCTGGCGGCGTGCTTAACACATGCAAGTCGAACGAGA
GGACATGAAAAGCTTGCTTTTTATGAAATCTAGTGGCAAACGGGTGAGTAACACGTAAACAACCTGCCTT
CAAGATGGGGACAACAGACGGAAACGACTGCTAATACCGAATACGATCCGAAAGTCGCATGACATTTGGA
TGAAAGGGTGGCCTATCGAAGAAGCTATCGCTTGAAGAGGGGTTTGCGTCCGATTAGGTAGTTGGTGAGG
TAACGGCCCACCAAGCCGACGATCGGTAGCCGGTCTGAGAGGATGAACGGCCACACTGGAACTGAGACAC
GGTCCAGACTCCTACGGGAGGCAGCAGTGGGGAATCTTCCGCAATGGACGAAAGTCTGACGGAGCAACGC
CGCGTGAGTGAAGACGGCCTTCGGGTTGTAAAGCTCTGTGATTCGGGACGAAAGGCCATATGTGAATAAT
ATATGGAAATGACGGTACCGAAAAAGCAAGCCACGGCTAACTACGTGCCAGCAGCCGCGGTAATACGTAG
GTGGCAAGCGTTGTCCGGAATTATTGGGCGTAAAGCGCGCGCAGGCGGTCTCTTAAGTCCATCTTAGAAG
TGCGGGGCTTAACCCCGTGATGGGATGGAAACTGGGAGACTGGAGTATCGGAGAGGAAAGTGGAATTCCT
AGTGTAGCGGTGAAATGCGTAGATATTAGGAAGAACACCGGTGGGCGAAGGCGACTTTCTGGACGAAAAC
TGACGCTGAGGCGCGAAAGCGTGGGGAGCAAACAGGATTAGATACCCTGGTAGTCCACGCCGTAAACGAT
GGATACTAGGTGTAGGAGGTATCGACCCCTTCTGTGCCGGAGTTAACGCAATAAGTATCCCGCCTGGGAA
GTACGATCGCAAGATTAAAACTCAAAGGAATTGACGGGGGCCCGCACAAGCGGTGGAGTATGTGGTTTAA
TTCGACGCAACGCGAAGAACCTTACCAAGTCTTGACATTGATCGCCATTCCAAGAGATTGGAAGTTCTCC
TTCGGGAGACGAGAAAACAGGTGGTGCACGGCTGTCGTCAGCTCGTGTCGTGAGATGTTGGGTTAAGTCC
CGCAACGAGCGCAACCCCTATCTTTTGTTGCCAGCACGTAGAGGTGGGAACTCAGAAGAGACCGCCGCAG
ACAATGCGGAGGAAGGTGGGGATGACGTCAAGTCATCATGCCCCTTATGACTTGGGCTACACACGTACTA
CAATGGGCTTTAACAAAGAGCAGCGAAACCGCGAGGTGGAGCGAAACTCAAAAACAAGCCCCCAGTTCAG
ATCGCAGGCTGCAACTCGCCTGCGTGAAGCAGGAATCGCTAGTAATCGCGGGTCAGCATACCGCGGTGAA
TACGTTCCCGGGCCTTGTACACACCGCCCGTCACACTATGAGAGTCGGAAACACCCGAAGCCGGTGAGGT
AACCGCAAGGAGCCAGCCGTCGAAGGTGGGGCTGATGATTGGAGTGAAGTCGTAACAAGGTAACCGTAAA
GGGC
>334905726|gb|EU394679.2|L.reuteri|Lactobacillus reuteri strain ATCC 55730 16S ribosomal RNA gene, complete sequence
AGAGTTTGATCCTGGCTCAGGATGAACGCCGGCGGTGTGCCTAATACATGCAAGTCGTACGCACTGGCCC
AACTGATTGATGGTGCTTGCACCTGATTGACGATGGATCACCAGTGAGTGGCGGACGGGTGAGTAACACG
TAGGTAACCTGCCCCGGAGCGGGGGATAACATTTGGAAACAGATGCTAATACCGCATAACAACAAAAGCC
ACATGGCTTTTGTTTGAAAGATGGCTTTGGCTATCACTCTGGGATGGACCTGCGGTGCATTAGCTAGTTG
GTAAGGTAACGGCTTACCAAGGCGATGATGCATAGCCGAGTTGAGAGACTGATCGGCCACAATGGAACTG
AGACACGGTCCATACTCCTACGGGAGGCAGCAGTAGGGAATCTTCCACAATGGGCGCAAGCCTGATGGAG
CAACACCGCGTGAGTGAAGAAGGGTTTCGGCTCGTAAAGCTCTGTTGTTGGAGAAGAACGTGCGTGAGAG

TAACTGTTCACGCAGTGACGGTATCCAACCAGAAAGTCACGGCTAACTACGTGCCAGCAGCCGCGGTAAT
ACGTAGGTGGCAAGCGTTATCCGGATTTATTGGGCGTAAAGCGAGCGCAGGCGGTTGCTTAGGTCTGATG
TGAAAGCCTTCGGCTTAACCGAAGAAGTGCATCGGAAACCGGGCGACTTGAGTGCAGAAGAGGACAGTGG
AACTCCATGTGTAGCGGTGGAATGCGTAGATATATGGAAGAACACCAGTGGCGAAGGCGGCTGTCTGGTC
TGCAACTGACGCTGAGGCTCGAAAGCATGGGTAGCGAACAGGATTAGATACCCTGGTAGTCCATGCCGTA
AACGATGAGTGCTAGGTGTTGGAGGGTTTCCGCCCTTCAGTGCCGGAGCTAACGCATTAAGCACTCCGCC
TGGGGAGTACGACCGCAAGGTTGAAACTCAAAGGAATTGACGGGGGCCCGCACAAGCGGTGGAGCATGTG
GTTTAATTCGAAGCTACGCGAAGAACCTTACCAGGTCTTGACATCTTGCGCTAACCTTAGAGATAAGGCG
TTCCCTTCGGGGACGTAATGACAGGTGGTGCATGGTCGTCGTCAGCTCGTGTCGTGAGATGTTGGGTTAA
GTCCCGCAACGAGCGCAACCCTTGTTACTAGTTGCCAGCATTAAGTTGGGCACTCTAGTGAGACTGCCGG
TGACAAACCGGAGGAAGGTGGGGACGACGTCAGATCATCATGCCCCTTATGACCTGGGCTACACACGTGC
TACAATGGACGGTACAACGAGTCGCAAGCTCGCGAGAGTAAGCTAATCTCTTAAAGCCGTTCTCAGTTCG
GACTGTAGGCTGCAACTCGCCTACACGAAGTCGGAATCGCTAGTAATCGCGGATCAGCATGCCGCGGTGA
ATACGTTCCCGGGCCTTGTACACACCGCCCGTCACACCATGGGAGTTTGTAACGCCCAAAGTCGGTGGCC
TAACCTTTATGGAGGGAGCCGCCTAAGGCGGGACAGATGACTGGGGTGAAGTCGTAACAAGGTAGCCGTA
GGAGAACCTGCGGCTGGATCACCTCCTTTCT
>29825721|gb|AF542231.1|Parvimonas|Parvimonas micra ATCC 33270 16S ribosomal RNA gene, partial sequence
TTAACACATGCAAGTCGAACGTGATTTTTGTGGAAATTCTTTCGGGAATGGAAATGAAATGAAAGTGGCG
AACGGGTGAGTAACACGTGAGCAACCTACCTTACACAGGGGGATAGCCGTTGGAAACGACGATTAATACC
GCATGAGACCACAGAATCGCATGATATAGGGGTCAAAGATTTATCGGTGTAAGAAGGGCTCGCGTCTGAT
TAGCTAGTTGGAAGGGTAAAGGCCTACCAAGGCGACGATCAGTAGCCGGTCTGAGAGGATGAACGGCCAC
ATTGGAACTGAGACACGGTCCAAACTCCTACGGGAGGCAGCAGTGGGGAATATTGCACAATGGGGGGAAC
CCTGATGCAGCGACGCCGCGTGAGCGAAGAAGGTTTTCGAATCGTAAAGCTCTGTCCTATGAGAAGATAA
TGACGGTATCATAGGAGGAAGCCCCGGCTAAATACGTGCCAGCAGCCGCGGTAATACGTATGGGGCGAGC
GTTGTCCGGAATTATTGGGCGTAAAGGGTACGTAGGCGGTTTTTTAAGTCAGGTGTGAAAGCGTGAGGCT
TAACCTCATTAAGCACTTGAAACTGGAAGACTTGAGTGAAGGAGAGGAAAGTGGAATTCCTAGTGTAGCG
GTGAAATGCGTAGATATTAGGAGGAATACCGGTGGCGAAGGCGACTTTCTGGACTTTTACTGACGCTCAG
GTACGAAAGCGTGGGGAGCAAACAGGATTAGATACCCTGGTAGTCCACGCCGTAAACGATGAATGCTAGG
TGTTGGGAGTCAAATCTCGGTGCCGAAGTTAACACATTAAGCATTCCGCCTGGGGAGTACGGTGGCAACA
CTGAAACTCAAAGGAATTGACGGGGACCCGCACAAGCAGCGGAGCATGTGGTTTAATTCGAAGCAACGCG
AAGAACCTTACCAAGGCTTGACATATAGTTGAGTTATTGAGAAATTGATAAGTCCCTCGGGACAACTATA
CAGGTGGTGCATGGTTGTCGTCAGCTCGTGTCGTGAGATGTTGGGTTAAGTCCCGCAACGAGCGCAACCC
TTATCTTCAGTTGCCAGCACGTAGAGGTGGGAACTCTGGAGAGACTGCCGATGACAAATCGGAGGAAGGT
GGGGATGACGTCAAATCATCATGCCCTTTATGTCTTGGGCTACACACGTGCTACAATGGTTGGTACAACG
AGAAGCGAGATAGAGATGTTAAGCGAAACTCTAAAAACCAATCTCAGTTCGGATTGTAGGCTGCAACTCG
CCTACATGAAGTCGGAGTTGCTAGTAATCGCGAATCAGAATGTCGCGGTGAATGCGTTCCCGGGTCTTGT
ACACACCGCCCGTCACACCATGGGAGTTGGCAATACCCGAAGCCGCCGATCTAACCGCAAGGAGGAAGGC
GTCGAAGGTAGGGT
>52222147|gb|AY738658.1|Atopobium|Uncultured Atopobium sp. clone 123-f 36 16S ribosomal RNA gene, partial sequence
TGGGGATCTTGCACAATGGGCGAAAGCCTGATGCAGCGACGCCGCGTGCGGGATGAAGGCCTTCGGGTTG
TAAACCGCTTTCAGCAGGGACGAGGCCGCAAGGTGACGGTACCTGCAGAAGAAGCCCCGGCTAACTACGT
GCCAGCAGCCGCGGTAATACGTAGGGGGCAAGCGTTATCCGGATTCATTGGGCGTAAAGCGCGCGTAGGC
GGTTTGTTAGGTCAGGAGTTAAATCTGGGGGCTCAACCCCTATCCGCTCCTGATACCGGCAGGCTTGAGT
CTGGTAGGGGAAGGCGGAATTCCAAGTGTAGCGGTGAAATGCGCAGATATTTGGAAGAACACCGGTGGCG
AAGGCGGCCTTCTGGGCCACGACTGACGCTGAGGCGCGAAAGCTAGGGGAGCGAACAGGATTAGATACCC
TGGTAGTCCTAGCCGTAAACGATGGACACTAGGTGTGGGGAGATTATACTTTCCGTGCCGCAGCCAACGC
ATTAAGTGTCCCGCCTGGGGAGTACGGTCGCAAGACTAAAACTCAAAGGAATTGACGGGGGCCCGCACAA
GCAGCGGAGCATGTGGCTTAATTCGAAGCAACGCGAAGAACCTTACCAGGGCTTGACATTTAGGTGAAGC
AGTGGAAACACTGTGGCCGAAAGGAGCCTAAACAGGTGGTGCATGGCTGTCGTCAGCTCGTGTCGTGAGA
TGTTGGGTTAAGTCCCGCAACGAGCGCAACCCTTGTCGCATGTTGCCAGCGGTTAAAGCCGGGCACCCAT
GCGAGACCGCCGGCGTTAAGCCGGAGGAAGGTGGGGACGACGTCAAGTCATCATGCCCCTTATGTCCTGG
GCTGCACACGTGCTACAATGGCCGGCACAGAGGGCTGCAACTGCGCGAGCAGAAGCGAATCCCTAAAGCC

GGTCCCAGTTCGGATTGGAGGCTGCAACCCGCCTCCATGAAGTCGGAGTTGCTAGTAATCGCGGATCAGC
ACGCCGCGGTGAATGCGTTCCCGGGCCT
>78482952|emb|AJ585206.2|Atopobium|Atopobium vaginae 16S rRNA gene, strain BVS059A5
GATGAACGCTGGCGGCGCGCCTAACACATGCAAGTCGAACGATTAAAGCACCTTCGGGTGTGTATAAAGT
GGCGAACGGCTGAGTAACACGTGGGCAACCTGCCCTTTGCACTGGGATAGCCTCGGGAAACCGAGGTTAA
TACCGGATACTCCATATATATCGCATGATGTATATGGGAAAGCTCCGACGGCAAAGGATGGGCCCGCGGC
CTGTTAGCTAGTTGGTGGGGTAACGGCCTACCAAGGCAATGATGGGTAGCCGGGTTGAGAGACCGACCGG
CCAGATTGGGACTGAGACACGGCCCAGACTCCTACGGGAGGCAGCAGTGGGGAATCTTGCACAATGGGCG
AAAGCCTGATGCAGCGACGCCGCGTGCGGGATGAAGGCCTTCGGGTTGTAAACCGCTTTCAGCAGGGACG
AGGCCGCAAGGTGACGGTACCTGCAGAAGAAGCCCCGGCTAACTACGTGCCAGCAGCCGCGGTAATACGT
AGGGGGCAAGCGTTATCCGGATTCATTGGGCGTAAAGCGCGCGTAGGCGGTTTGTTAGGTCAGGAGTTAA
ATCTGGGGGCTCAACCCCTATCCGCTCCTGATACCGGCAGGCTTGAGTCTGGTAGGGGAAGGCGGAATTC
CAAGTGTAGCGGTGAAATGCGCAGATATTTGGAAGAACACCGGTGGCGAAGGCGGCCTTCTGGGCCACGA
CTGACGCTGAGGCGCGAAAGCTAGGGGAGCGAACAGGATTAGATACCCTGGTAGTCCTAGCCGTAAACGA
TGGACACTAGGTGTGGGGAGATTATACTTTCCGTGCCGCAGCCAACGCATTAAGTGTCCCGCCTGGGGAG
TACGGTCGCAAGACTAAAACTCAAAGGAATTGACGGGGGCCCGCACAAGCAGCGGAGCATGTGGCTTAAT
TCGAAGCAACGCGAAGAACCTTACCAGGGCTTGACATTTAGGTGAAGCAGTGGAAACACTGTGGCCGAAA
GGAGCCTAAACAGGTGGTGCATGGCTGTCGTCAGCTCGTGTCGTGAGATGTTGGGTTAAGTCCCGCAACG
AGCGCAACCCTTGTCGCATGTTGCCAGCGGTTAAAGCCGGGCACCCATGCGAGACCGCCGGCGTTAAGCC
GGAGGAAGGTGGGGACGACGTCAAGTCATCATGCCCCTTATGTCCTGGGCTGCACACGTGCTACAATGGC
CGGCACAGAGGGCTGCAACTGCGCGAGCAGAAGCGAATCCCTAAAGCCGGTCCCAGTTCGGATTGGAGGC
TGCAACCCGCCTCCATGAAGTCGGAGTTGCTAGTAATCGCGGATCAGCACGCCGCGGTGAATGCGTTCCC
GGGCCTTGTACACACCGCCCGTCACAC
>336454742|dbj|AB640696.1|Peptostreptococcus|P. anaerobius gene for 16S ribosomal RNA, partial sequence, strain: JCM 6478
AGAGTTTGATCCTGGCTCAGGATGAACGCTGGCGGCGTGCCTAACACATGCAAGTCGAGCGCGTCTGATT
TGATGCTTGCATTGATGAAAGATGAGCGGCGGACGGGTGAGTAACGCGTGGGTAACCTGCCCTATACACA
TGGATAACATACTGAAAAGTTTACTAATACATGATAATATATATTTACGGCATCGTAGATATATCAAAGT
GTTAGCGGTATAGGATGGACCCGCGTCTGATTAGCTAGTTGGTGAGATAACTGCCCACCAAGGCGACGAT
CAGTAGCCGACCTGAGAGGGTGATCGGCCACATTGGAACTGAGACACGGTCCAAACTCCTACGGGAGGCA
GCAGTGGGGAATATTGCACAATGGGCGCAAGCCTGATGCAGCAACGCCGCGTGAACGATGAAGGTCTTCG
GATCGTAAAGTTCTGTTGCAGGGGAAGATAATGACGGTACCCTGTGAGGAAGCCCCGGCTAACTACGTGC
CAGCAGCCGCGGTAATACGTAGGGGGCTAGCGTTATCCGGATTTACTGGGCGTAAAGGGTGCGTAGGTGG
TCTTTCAAGTCGGTGGTTAAAGGCTACGGCTCAACCGTAGTTAGCCTCCGAAACTGGAAGACTTGAGTGC
AGGAGAGGAAAGTGGAATTCCCAGTGTAGCGGTGAAATGCGTAGATATTGGGAGGAACACCAGTAGCGAA
GGCGGCTTTCTGGACTGCAACTGACACTGAGGCACGAAAGCGTGGGTAGCAAACAGGATTAGATACCCTG
GTAGTCCACGCTGTAAACGATGAGTACTAGGTGTCGGGGGTTACCCCCCTCGGTGCCGCAGCTAACGCAT
TAAGTACTCCGCCTGGGGAGTACGCACGCAAGTGTGAAACTCAAAGGAATTGACGGGGACCCGCACAAGT
AGCGGAGCATGTGGTTTAATTCGAAGCAACGCGAAGAACCTTACCTAAGCTTGACATCCCTTAGACCGGT
GTTTAATCACACCTTCCCTTCGGGGCTGAGGTGACAGGTGGTGCATGGTTGTCGTCAGCTCGTGTCGTGA
GATGTTGGGTTAAGTCCCGCAACGAGCGCAACCCTTGTCTTTAGTTGCCAGCATTCAGTTGGGCACTCTA
GAGAGACTGCCAGGGATAACCTGGAGGAAGGTGGGGATGACGTCAAATCATCATGCCCCTTATGCTTAGG
GCTACACACGTGCTACAATGGGTGGTACAGAGGGTTGCCAAACCGTGAGGTGGAGCTAATCCCTTAAAGC
CATTCTCAGTTCGGATTGTAGGCTGAAACTCGCCTACATGAAGCTGGAGTTACTAGTAATCGCAGATCAG
AATGCTGCGGTGAATGCGTTCCCGGGTCTTGTACACACCGCCCGTCACACCATGGGAGTCGGAAACACCC
GAAGCCGATTATCCAACCGCAAGGAGGAAGTCGTCGAAGGTGGCGTCGATAACTGGGGTGAAGTCGTAAC
AAGGTAACC
>52222156|gb|AY738667.1|Gardnerella|Uncultured Gardnerella sp. clone BV6-77 16S ribosomal RNA gene, partial sequence
TGGGGAATATTGCGCAATGGGGGAAACCCTGACGCAGCGACGCCGCGTGCGGGATGAAGGCCTTCGGGTT
GTAAACCGCTTTTGATTGGGAGCAAGCCTTCGGGTGAGTGTACCTTTCGAATAAGCGCCGGCTAACTACG
TGCCAGCAGCCGCGGTAATACGTAGGGCGCAAGCGTTATCCGGATTTATTGGGCGTAAAGAGCTTGTAGG
CGGTTCGTCGCGTCTGGTGTGAAAGCCCATCGCTTAACGGTGGGTCTGCGCCGGGTACGGGCGGGCTAGA

GTGCAGTAGGGGAAACTGGAATTCTCGGTGTAACGGTGGAATGTGTAGATATCGGGAAGAACACCAATGG
CGAAGGCAGGTTTCTGGGCTGTTACTGACGCTGAGAAGCGAAAGCGTGGGGAGCGAACAGGATTAGATAC
CCTGGTAGTCCACGCCGTAAACGGTGGACGCTGGATGTGGGGCCCATTCCACGGGTTCCGTGTCGGAGCT
AACGCGTTAAGCGTCCCGCCTGGGGAGTACGGCCGCAAGGCTAAAACTCAAAGAAATTGACGGGGGCCCG
CACAAGCGGCGGAGCATGCGGATTAATTCGATGCAACGCGAAGAACCTTACCTGGGCTTGACATGTGCCT
GATGACTGCAGAGATGTGGTTTCCTTTCGGGGCAGGTTCACAGGTGGTGCATGGTCGTCGTCAGCTCGTG
TCGTGAGATGTTGGGTTAAGTCCCGCAACGAGCGCAACCCTCGCCCTGTGTTGCCAGCGGGTTATGCCGG
GAACTCACGGGGGACCGCCGGGGTTAACTCGGAGGAAGGTGGGGATGACGTCAGATCATCATGCCCCTTA
CGTCCAGGGCTTCACGCATGCTACAATGGCCGGTACAACGGGGTGCGACATGGTGACATGGAGCTAATCC
CTTAAAACCGGTCTCAGTTCGGATCGTAGTCTGCAACTCGACTACGTGAAGGCGGAGTCGCTAGTAATCG
CGAATCAGCAACGTCGCGGTGAATGCGTTCCCGGGCCT
>66878859|gb|AY959153.1|Gardnerella|Uncultured bacterium clone rRNA380 16S ribosomal RNA gene, partial sequence
TCGCCCTTAGAGTTTGATCCTGGCTCAGGATGAACGCTGGCGGCGTGCTTAACACATGCAAGTCGAACGG
GATCTGACCAGCTTGCTGGTTGGTGAGAGTGGCGAACGGGTGAGTAATGCGTGACCAACCTACCCCATGC
TCCAGAATAGCTCCTGGAAACGGGTGGTAATGCTGGATGCTCCAACTTGACGCATGTTTTGTTGGGAAAG
TGTTTAGCGGCATGGGATGGGGTCGCGTCCTATCAGCTTGTAGGCGGGGTAATGGCCCACCTAGGCTTCG
ACGGGTAGCCGGCCTGAGAGGGCGGACGGCCACATTGGGACTGAGATACGGCCCAGACTCCTACGGGAGG
CAGCAGTGGGGAATATTGCGCAATGGGGGAAACCCTGACGCAGCGACGCCGCGTGCGGGATGAAGGCCTT
CGGGTTGTAAACCGCTTTTGATTGGGAGCAAGCCTTCGGGTGAGTGTACCTTTCGAATAAGCGCCGGCTA
ACTACGTGCCAGCAGCCGCGGTAATACGTAGGGCGCAAGCGTTATCCGGAATTATTGGGCGTAAAGAGCT
TGTAGGCGGTTCGTCGCGTCTGGTGTGAAAGCCCATCGCTTAACGGTGGGTCTGCGCCGGGTACGGGCGG
GCTAGAGTGCAGTAGGGGAAACTGGAATTCTCGGTGTAACGGTGGAATGTGTAGATATCGGGAAGAACAC
CAATGGCGAAGGCAGGTTTCTGGGCTGTTACTGACGCTGAGAAGCGAAAGCGTGGGGAGCGAACAGGATT
AGATACCCTGGTAGTCCACGCCGTAAACGGTGGACGCTGGATGTGGGGCCCATTCCACGGGTTCCGTGTC
GGAGCTAACGCGTTAAGCGTCCCGCCTGGGGAGTACGGCCGCAAGGCTAAAACTCAAAGAAATTGACGGG
GGCCCGCACAAGCGGCGGAGCATGCGGATTAATTCGATGCAACGCGAAGAACCTTACCTGGGCTTGACAT
GTGCCTGATGACTGCAGAGATGTGGTTTCCTTTCGGGGCAGGTTCACAGGTGGTGCATGGTCGTCGTCAG
CTCGTGTCGTGAGATGTTGGGTTAAGTCCCGCAACGAGCGCAACCCTCGCCCTGTGTTGCCAGCGGGTTA
TGCCGGGAACTCACGGGGGACCGCCGGGGTTAACTCGGAGGAAGGTGGGGATGACGTCAGATCATCATGC
CCCTTACGTCCAGGGCTTCACGCATGCTACAATGGCCGGTACAACGGGGTGCGACATGGTGACATGGAGC
TAATCCCTTAAAACCGGTCTCAGTTCGGATCGTAGTCTGCAACTCGACTACGTGAAGGCGGAGTCGCTAG
TAATCGCGAATCAGCAACGTCGCGGTGAATGCGTTCCCGGGCCTTGTACACACCGCCCGTCAAGTCATGA
AAGTGGGCAGCACCCGAAGCCGGTGGCCTGACCTTTTTGGAGGGAGCCGTCTAAGGTGAGGCTCGTGATT
GGGACTAAGTCGTAACAAGGTAACCGTAAAGG
>306850716|gb|GQ900640.1|BVAB2|Uncultured bacterium clone 123b-28 16S ribosomal RNA gene, partial sequence
TGGGGAATATTGGGCAATGGGCGAAAGCCTGACCCAGCAACGCCGCGTGAGTGATGAAGGCCTTCGGGTT
GTAAAACTCTTTGGACAGGGACGAAGAAAGTGACGGTACCTGTAGAACAAGCCACGGCTAACTACGTGCC
AGCAGCCGCGGTAATACGTAGGTGGCGAGCGTTATCCGGATTTACTGGGCGTAAAGGGCGTGTAGGCGGC
TAGATAAGTGTGATGTTTAAATCCAAGGCTTAACCTTGGGGTTCATTACAAACTGTTTAGCTTGAGTGCT
GGAGAGGATAGTGGAATTCCTAGTGTAGCGGTAAAATGCGTAGATATTAGGAGGAACACCGGTGGCGAAG
GCGGCTATCTGGACAGTAACTGACGCTGAGGCGCGAAAGCGTGGGGAGCAAACAGGATTAGATACCCTGG
TAGTCCACGCCGTAAACGATGAATACTAGCTGTAGGAGGTATCGACCCCTTCTGTGGCGCAGTTAACACA
ATAAGTATTCCGCCTGGGGAGTACGGCCGCAAGGTTAAAACTCAAAGGAATTGACGGGGACCCGCACAAG
CAGTGGATTATGTGGTTTAATTCGAAGCAACGCGAAGAACCTTACCAGGACTTGACATCCTCTGACGATT
CAGGAGACTGAATTTTCTCTTCGGAGACAGAGAGACAGGTGGTGCATGGTTGTCGTCAGCTCGTGTCGTG
AGATGTTGGGTTAAGTCCCGCAACGAGCGCAACCCCTATTGATTGTTGCTAACAGTAAGATGAGCACTCA
ATTGAGACTGCCGTTGATAAAACGGAGGAAGGTGGGGACGACGTCAAATCATCATGCCCCTTATGTTCTG
GGCTACACACGTAATACAATGGCTGTGACAGAGGGAAGCAAGAGGGCGACCTTAAGCGAA
>19744807|gb|AF487886.1|Prevotella|Prevotella sp. Smarlab 121567 16S ribosomal RNA gene, partial sequence
TAGAGTTTGATCCTGGCTCAGGATGAACGCTAGCTACAGGCTTAACACATGCAAGTCGCGGGGCAGCATG
AGGGTTGCTTGCAACCTTTGATGGCGACCGGCGCACGGGTGAGTAACACGTATCCAACCTACTCCTTACA

ACGGAATAACCCGGCGAAAGTCGGACTAATACCGTATGATATCCTATGCAGGCATCTAACTAGGATTAAA
GGTTTAGCGGTAAGGGATGGGGATGCGTCTGATTAGGCAGTTGGCGGGGTAACGGCCCACCAAACCGACG
ATCAGTAGGGGTTCTGAGAGGAAGGTCCCCCACATTGGAACTGAGACACGGTCCAAACTCCTACGGGAGG
CAGCAGTGAGGAATATTGGTCAATGGACGGAAGTCTGAACCAGCCAAGTAGCGTGCAGGATGACGGCCCT
ATGGGTTGTAAACTGCTTTTATACGGGGATAAAGTTAGGGACGTGTCCCTATTTGCAGGTACCGTATGAA
TAAGGACCGGCTAATTCCGTGCCAGCAGCCGCGGTAATACGGAAGGTCCGGGCGTTATCCGGATTTATTG
GGTTTAAAGGGAGCGTAGGCCGTAGATTAAGTGTGTTGTGAAATGTAGACGCTCAACGTCTGACTTGCAG
CGCATACTGGTTTACTTGAGTTTGCACAACGTTGGCGGAATTCGTCGTGTAGCGGTGAAATGCTTAGATA
TGACGAAGAACTCCGATTGCGAAGGCAGCTTGCGGGAGCAAAACTGACGCTGAAGCTCGAAAGTGCGGGT
ATCGAACAGGATTAGATACCCTGGTAGTCCGCACGGTAAACGATGGATGCCCGTTGTATGGCCTTTCGGC
TATGTGACCAAGCGAAAGCATTAAGCATCCCACCTGGGGAGTACGCCGGCAACGGTGAAACTCAAAGGAA
TTGACGGGGGCCCGCACAAGCGGAGGAACATGTGGTTTAATTCGATGATACGCGAGGAACCTTACCCGGG
CTTGAACTGTAGGCGAACGATCTAGAGATAGTGAGGCCCTTCGGGGCGCCTACGGAGGTGCTGCATGGTT
GTCGTCAGCTCGTGCCGTGAGGTGTCGGCTTAAGTGCCATAACGAGCGCAACCCCTGTTCTCAGTTGCCA
TCGGGTGATGCCGGGCACTCTGAGGATACTGCCTCCGCAAGGAGTGAGGAAGGTGGGGATGACGTCAAAT
CAGCACGGCCCTTACGTCCGGGGCTACACACGTGTTACAATGGCCGGTACAGAGCGTCGGTCGTATGCAA
ATACGATCTAATCTTTAAAACCGGTCTCAGTTCGGACTGGGGTCTGCAACCCGACCCCACGAAGCTGGAT
TCGCTAGTAATCGCGCATCAGCCATGGCGCGGTGAATACGTTCCCGGGCCTTGTACACACCGCCCGTCAA
GCCATGAAAGCCGGGGGCGCCTGAAGTCCGCGACCGCAAGGAACGGCCTAGGGCGAAACTGGTGATTGGG
GCTAAGTCGTAACAAGGTA
>164460376|gb|EU189000.1|Sneathia|Uncultured bacterium clone 123Q-34 16S ribosomal RNA gene, partial sequence
ACCTACAGAAGAAGCGACGGCTAAATACGTGCCAGCAGCCGCGGTAATACGTATGTCGCGAGCGTTATCC
GGAATTATTGGGCTTAAAGGGCATCTAGGCGGTTAAACAAGTTGAAGGTGAAAACCTGTGGCTCAACCAT
AGGCTTGCCTACAAAACTGTATAACTAGAGTACTGGAAAGGTGGGTGGAACTACACGAGTAGAGGTGAAA
TTCGTAGATATGTGTAGGAATGCCGATGATGAAGATAACTCACTGGACAGCAACTGACGCTGAAGTGCGA
AAGCTAGGGGAGCAAACAGGATTAGATACCCTGGTAGTCCTAGCTGTAAACGATGATCACTGGGTGTGGG
GATTCGAAGTCTCTGTGCCGAAGCAAAAGCGATAAGTGATCCGCCTGGGGAGTACGTTCGCAAGAATGAA
ACTCAAAGGAATTGACGGGGACCCGCACAAGTGGTGGAGCATGTGGTTTAATTCGACGCAACGCGAGGAA
CCTTACCAGATCTTGACATCCTCCGAAGAGCATAGAAGTATGCTTGTGCCTACGGGAACGGAGAGACAGG
TGGTGCATGGCTGTCGACAGCTCGTGTTGTGAGATGTTGGGTTAAGTCCCGCAACGAGCGAAACCCCTAT
CATTAGTTACCATCATTAAGTTGGGGACTCTAATGAAACTGCCTACGAAGNGTAGGAGGAAGGTGGGGAT
GACGTCAAGTCATCATGCCCCTTATGATCTGGGCTACACACGTGCTACAATGGATAGTACAAAGAGAAGC
TTTGTAGCGATACATGGCAAAACTAAGAAAGCTATTCTTAGTTCGGATTGAAGTCTGCAACTCGACTTCA
TGAAGTTGGAATCACTAGTAATCGTGACTCAGCAATGTCACGGTGAATACGTTCTCGGGT
>52222148|gb|AY738659.1|Sneathia|Uncultured Sneathia sp. clone 123-f 47 16S ribosomal RNA gene, partial sequence
TGGGGAATATTGGACAATGGAGGCAACTCTGATCCAGCAATTCTGTGTGTGTGAAGAAGGTTTTAGGACT
GTAAAACACTTTTAGTAGGGAAGAAAGAAATGACGGTACCTACAGAAGAAGCGACGGCTAAATACGTGCC
AGCAGCCGCGGTAATACGTATGTCGCGAGCGTTATCCGGAATTATTGGGCTTAAAGGGCATCTAGGCGGT
TAAACAAGTTGAAGGTGAAAACCTGTGGCTCAACCATAGGCTTGCCTACAAAACTGTATAACTAGAGTAC
TGGAAAGGTGGGTGGAACTACACGAGTAGAGGTGAAATTCGTAGATATGTGTAGGAATGCCGATGATGAA
GATAACTCACTGGACAGCAACTGACGCTGAAGTGCGAAAGCTAGGGGAGCAAACAGGATTAGATACCCTG
GTAGTCCTAGCTGTAAACGATGATCACTGGGTGTGGGGATTCGAAGTCTCTGTGCCGAAGCAAAAGCGAT
AAGTGATCCGCCTGGGGAGTACGTTCGCAAGAATGAAACTCAAAGGAATTGACGGGGACCCGCACAAGTG
GTGGAGCATGTGGTTTAATTCGACGCAACGCGAGGAACCTTACCAGATCTTGACATCCTCCGAAGAGCAT
AGAAGTATGCTTGTGCCTACGGGAACGGAGAGACAGGTGGTGCATGGCTGTCGACAGCTCGTGTTGTGAG
ATGTTGGGTTAAGTCCCGCAACGAGCGAAACCCCTATCATTAGTTACCATCATTAAGTTGGGGACTCTAA
TGAAACTGCCTACGAAGAGTAGGAGGAAGGTGGGGATGACGTCAAGTCATCATGCCCCTTATGATCTGGG
CTACACACGTGCTACAATGGATAGTACAAAGAGAAGCTTTGTAGCGATACATGGCAAAACTAAGAAAGCT
ATTCTTAGTTCGGATTGAAGTCTGCAACTCGACTTCATGAAGTTGGAATCACTAGTAATCGTGAATCAGC
AATGTCACGGTGAATACGTTCTCGGGTCT
>164460324|gb|EU188948.1|Aerococcus|Uncultured bacterium clone 123f-92 16S ribosomal RNA gene, partial sequence

GTTGTAAGAGAAGAACAAATTGTAGAGTAACTGCTACAGTCTTGACGGTATCTTACCAGAAAGCCACGGC
TAACTACGTGCCAGCAGCCGCGGTAATACGTAGGTGGCAAGCGTTGTCCGGATTTATTGGGCGTAAAGGG
GGCGCAGGCTGCTTCTTAAGTCTGATGTGAAAGCCCACGGCTTAACCGTGGAAGTGCATTGGAAACTGGG
AAGCTTGAGTACAGAAGAGGAAAGTGGAACTCCATGTGTAGCGGTGGAATGCGTAGATATATGGAAGAAC
ACCAGTGGCGAAAGCGACTTTCTGGTCTGTCACTGACGCTGAGGCCCGAAAGCGTGGGTAGCAAACAGGA
TTAGATACCCTGGTAGTCCACGCCGTAAACGATGAGCGCTAGGTGTTGGAGGGTTTCCACCCTTCAGTGC
CGCAGCTAACGCATTAAGCGCTCCGCCTGGGGAGTACGACCGCAAGGTTGAAACTCAAAGGAATTGACGG
GGACCCGCACAAGCGGTGGAGCATGTGGTTTAATTCGAAGCAACGCGAAGAACCTTACCAAGTCTTGACA
TCCTTTGACCACTCTAGAGATAGAGCTTTCCCTTCGGGGACAAAGTGACAGGTGGTGCATGGTTGTCGTC
AGCTCGTGTCGTGAGATGTTGGGTTAAGTCCCGCAACGAGCGCAACCCCTATTGTTAGTTGCCAGCATTG
AGTTGGGCACTCTAGCAAGACTGCCGGTGACAAACCGGAGGAAGGCGGGGATGACGTCAAATCATCATGC
CCCTTATGACTTGGGCTACACACGTGCTACAATGGATGGTACAACGGGCAGCGAGCTCGCGAGAGTCAGC
GAATCCCTTAAAGCCATTCTCAGTTCGGATTGTAGTCTGCAACTCGACTACATGAAGCCG
>315452163|gb|HQ641662.1|Gardnerella|Gardnerella vaginalis strain GV17
16S ribosomal RNA gene, partial sequence
TGACCAGCTTGCTGGTTGGTGAGAGTGGCGAACGGGTGAGTAATGCGTGACCAACCTGCCCCATGCTCCA
GAATAGCTCTTGGAAACGGGTGGTAATGCTGGATGCTCCAACTTGACGCATGTCTTGTTGGGAAAGTGTT
TAGTGGCATGGGATGGGGTCGCGTCCTATCAGCTTGTAGGCGGGGTAATGGCCCACCTAGGCTTCGACGG
GTAGCCGGCCTGAGAGGGCGGACGGCCACATTGGGACTGAGATACGGCCCAGACTCCTACGGGAGGCAGC
AGTGGGGAATATTGCGCAATGGGGGAAACCCTGACGCAGCGACGCCGCGTGCGGGATGAAGGCCTTCGGG
TTGTAAACCGCTTTTGATTGGGAGCAAGCTTTCGGGTGAGTGTACCTTTCGAATAAGCGCCGGCTAACTA
CGTGCCAGCAGCCGCGGTAATACGTAGGGCGCAAGCGTTATCCGGAATTATTGGGCGTAAAGAGCTTGTA
GGCGGTTCGTCGCGTCTGGTGTGAAAGCCCATCGCTTAACGGTGGGTTTGCGCCGGGTACGGGCGGGCTA
GAGTGCAGTAGGGGAGACTGGAATTCTCGGTGTAACGGTGGAATGTGTAGATATCGGGAAGAACACCAAT
GGCGAAGGCAGGTCTCTGGGCTGTTACTGACGCTGAGAAGCGAAAGCGTGGGGAGCGAACAGGATTAGAT
ACCCTGGTAGTCCACGCCGTAAACGGTGGACGCTGGATGTGGGGCCCATTCCACGGGTTCTGTGTCGGAG
CTAACGCGTTAAGCGTCCCGCCTGGGGAGTACGGCCGCAAGGCTAAAACTCAAAGAAATTGACGGGGGCC
CGCACAAGCGGCGGAGCATGCGGATTAATTCGATGCAACGCGAAGAACCTTACCTGGGCTTGACATGTGC
CTGTCGACTGCAGAGATGTGGTTTCCCTTCGGGGCAGGTTCACAGGTGGTGCATGGTCGTCGTCAGCTCG
TGTCGTGAGATGTTGGGTTAAGTCCCGCAACGAGCGCAACCCTCGCCCTGTGTTGCCAGCGGGTTATGCC
GGGAACTCACGGGGGACCGCCGGGGTTAACTCGGAGGAAGGTGGGGATGACGTCAGATCATCATGCCCCT
TACGTCCAGGGCTTCACGCATGCTACAATGGCCGGTACAACGGGATGCGACATGGTGACATGGAGCGGAT
CCCTTAAAACCGGTCTCAGTTCGGATCGTAGTCTGCAACTCGACTACGTGAAGGCGGAGTCGCTAGTAAT
CGCGAATCAGCAACGTCGCGGTGAATGCGTTCCCGGGCCTTGTACACACCGCCCGTCA
>52222151|gb|AY738662.1|Dialister|Uncultured Dialister sp. clone 127-Q 46
16S ribosomal RNA gene, partial sequence
TGGGGAATCTTCCGCAATGGACGAAAGTCTGACGGAGCAACGCCGCGTGAGTGAAGACGGCCTTCGGGTT
GTAAAGCTCTGTGATTCGGGACGAAAGGCCATATGTGAATAATATATGGAAATGACGGTACCGAAAAAGC
AAGCCACGGCTAACTACGTGCCAGCAGCCGCGGTAATACGTAGGTGGCAAGCGTTGTCCGGAATTATTGG
GCGTAAAGCGCGCGCAGGCGGTCACTTAAGTCCATCTTAGAAGTGCGGGGCTTAACCCCGTGATGGGATG
GAAACTGGGAGACTGGAGTATCGGAGAGGAAAGTGGAATTCCTAGTGTAGCGGTGAAATGCGTAGATATT
AGGAAGAACACCGGTGGCGAAGGCGACTTTCTGGACGAAAACTGACGCTGAGGCGCGAAAGCGTGGGGAG
CAAACAGGATTAGATACCCTGGTAGTCCACGCCGTAAACGATGGATACTAGGTGTAGGAGGTATCGACCC
CTTCTGTGCCGGAGTTAACGCAATAAGTATCCCGCCTGGGAAGTACGATCGCAAGATTAAAACTCAAAGG
AATTGACGGGGGCCCGCACAAGCGGTGGAGTATGTGGTTTAATTCGACGCAACGCGAAGAACCTTACCAA
GTCTTGACATTGATCGCCATTCCAAGAGATTGGAAGTTCTCCTTCGGGAGACGAGAAAACAGGTGGTGCA
CGGCTGTCGTCAGCTCGTGTCGTGAGATGTTGGGTTAAGTCCCGCAACGAGCGCAACCCCTATCTTTTGT
TGCCAGCACGTAGAGGTGGGAACTCAGAAGAGACCGCCGCAGACAATGCGGAGGAAGGTGGGGATGACGT
CAAGTCATCATGCCCCTTATGACTTGGGCTACACACGTACTACAATGGGCTTTAACAAAGAGCAGCGAAA
CCGCGAGGTGGAGCGAAACTCAAAAACAAGCCCCCAGTTCAGATCGCAGGCTGCAACTCGCCTGCGTGAA
GCAGGAATCGCTAGTAATCGCGGGTCAGCATACCGCGGTGAATACGTTCCCGGGCCT
>66878580|gb|AY958874.1|Dialister|Uncultured bacterium clone rRNA101 16S
ribosomal RNA gene, partial sequence
GCCCTTAGAGTTTGATCCTGGCTCAGGACGAACGCTGGCGGCGTGCTTAACACATGCAAGTCGAACGAGA

GGACATGAAAAGCTTGCTTTTTATGAAATCTAGTGGCAAACGGGTGAGTAACACGTAAACAACCTGCCTT
CAAGATGGGGACAACAGACGGAAACGACTGCTAATACCGAATACGATCCGAAAGTCGCATGACATTTGGA
TGAAAGGGTGGCCTATCGAAGAAGCTATCGCTTGAAGAGGGGTTTGCGTCCGATTAGGTAGTTGGTGAGG
TAACGGCCCACCAAGCCGACGATCGGTAGCCGGTCTGAGAGGATGAACGGCCACACTGGAACTGAGACAC
GGTCCAGACTCCTACGGGAGGCAGCAGTGGGGAATCTTCCGCAATGGACGAAAGTCTGACGGAGCAACGC
CGCGTGAGTGAAGACGGCCTTCGGGTTGTAAAGCTCTGTGATTCGGGACGAAAGGCCATATGTGAATAAT
ATATGGAAATGACGGTACCGAAAAAGCAAGCCACGGCTAACTACGTGCCAGCAGCCGCGGTAATACGTAG
GTGGCAAGCGTTGTCCGGAATTATTGGGCGTAAAGCGCGCGCAGGCGGTCACTTAAGTCCATCTTAGAAG
TGCGGGGCTTAACCCCGTGATGGGATGGAAACTGGGAGACTGGAGTATCGGAGAGGAAAGTGGAATTCCT
AGTGTAGCGGTGAAATGCGTAGATATTAGGAAGAACACCGGTGGCGAAGGCGACTTTCTGGACGAAAACT
GACGCTGAGGCGCGAAAGCGTGGGGAGCAAACAGGATTAGATACCCTGGTAGTCCACGCCGTAAACGATG
GATACTAGGTGTAGGAGGTATCGACCCCTTCTGTGCCGGAGTTAACGCAATAAGTATCCCGCCTGGGAAG
TACGATCGCAAGATTAAAACTCAAAGGAATTGACGGGGGCCCGCACAAGCGGTGGAGTATGTGGTTTAAT
TCGACGCAACGCGAAGAACCTTACCAAGTCTTGACATTGATCGCCATTCCAAGAGATTGGAAGTTCTCCT
TCGGGAGACGAGAAAACAGGTGGTGCACGGCTGTCGTCAGCTCGTGTCGTGAGATGTTGGGTTAAGTCCC
GCAACGAGCGCAACCCCTATCTTTTGTTGCCAGCACGTAGAGGTGGGAACTCAGAAGAGACCGCCGCAGA
CAATGCGGAGGAAGGTGGGGATGACGTCAAGTCATCATGCCCCTTATGACTTGGGCTACACACGTACTAC
AATGGGCTTTAACAAAGAGCAGCGAAACCGCGAGGTGGAGCGAAACTCAAAAACAAGCCCCCAGTTCAGA
TCGCAGGCTGCAACTCGCCTGCGTGAAGCAGGAATCGCTAGTAATCGCGGGTCAGCATACCGCGGTGAAT
ACGTTCCCGGGCCTTGTACACACCGCCCGTCACACTATGAGAGTCGGAAACACCCGAAGCCGGTGAGGTA
ACCGCAAGGAGCCAGCCGTCGAAGGTGGGGCTGATGATTGGAGTGAA
>183398320|gb|AY959087.2|L.iners|Uncultured bacterium clone rRNA314 16S ribosomal RNA gene, partial sequence
CCCTTAGAGTTTGATCCTGGCTCAGGACGAACGCTGGCGGCGTGCCTAATACATGCAAGTCGAGCGAGTC
TGCCTTGAGGATCGGAGTGCTTGCACTCTGTGAAACAAGATACAGGCTAGCGGCGGACGGGTGAGTAACA
CGTGGGTAACCTGCCCAAGAGATCGGGATAACACCTGGAAACAGATGCTAATACCGGATAACGACAGATG
ATGCCTATCAACTGTTTAAAAGATGGTTCTGCTATCACTCTTGGATGGACCTGCGGTGCATTAGGTAGTT
GGTAGGGTAACGGCCAACCAAGGCGATGATGCATAGCCGAGTTGAGAGACTGATCGGCCACATTGGGACT
GAGACACGGCCCAAACTCCTACGGGAGGCAGCAGTAGGGAATCTTCCACAATGGACGCAAGTCTGATGGA
GCAACGCCGCGTGAGTGAAGAAGGGTTTCGGCTCGTAAAGCTCTGTTGTTGGTGAAGAAGGACGGGGGTA
GTAACTGACCTTTGTTTGACGGTAATCAATTAGAAAGTCACGGCTAACTACGTGCCAGCAGCCGCGGTAA
TACGTAGGTGGCAAGCGTTGTCCGGATTTATTGGGCGTAAAGCGAGTGCAGGCGGTTCGATAAGTCTGAT
GTGAAAGCCTTCGGCTCAACCGGAGAATTGCATCAGAAACTGTCGAGCTTGAGTACAGAAGAGGAGAGTG
GAACTCCATGTGTAGCGGTGAAATGCGTAGATATATGGAAGAACACCGGTGGCGAAGGCGGCTCTCTGGT
CTGTTACTGACGCTGAGGCTCGAAAGCATGGGTAGCGAACAGGATTAGATACCCTGGTAGTCCATGCCGT
AAACGATGAGTGCTAAGTGTTGGGAGGTTTCCGCCTCTCAGTGCTGCAGCTAACGCATTAAGCACTCCGC
CTGGGGAGTACGACCGCAAGGTTGAAACTCAAAGGAATTGACGGGGGCCCGCACAAGCGGTGGAGCATGT
GGTTTAATTCGAAGCAACGCGAAGAACCTTACCAGGTCTTGACATCCATAGCCAGTCTAAGAGATTAGAT
GTTCCCTTCGGGGACTATGAGACAGGTGGTGCATGGCTGTCGTCAGCTCGTGTCGTGAGATGTTGGGTTA
AGTCCCGCAACGAGCGCAACCCTTGTCATTAGTTGCCAGCATTAAGTTGGGCACTCTAATGAGACTGCCG
GTGACAAACCGGAGGAAGGTGGGGATGACGTCAAGTCATCATGCCCCTTATGACCTGGGCTACACACGTG
CTACAATGGACGGTACAACGAGAAGCGACCCTGTGAAGGCAAGCGGATCTCTGAAAGCCGTTCTCAGTTC
GGATTGCAGGCTGCAACTCGCCTGCATGAAGCTGGAATCGCTAGTAATCGCAAATCAGCACGTTGCGGTG
AATACGTTCCCGGGCCTTGTACACACCGCCCGTCACACCATGAGAGTCTGTAACGCCCGAAGCCGGCGGG
ATAACCGAAAGGAGTCAGCCGTCTAAGGCGGGACAGATGATTAGGGTGAAGTCGTAACAAGGTAACCGTA
>239923964|gb|GQ179686.1|Fusobacterium|Uncultured Fusobacterium sp. clone VE32H03 16S ribosomal RNA gene, partial sequence
GATGAACGCTGACAGAATGCTTAACACATGCAAGTCGACTCGAGTCTTCGGACTTGGGTGGCGGACGGGT
GAGTAACGCGTAAAGAACTTGCCTCATAGTCTGGGACAACATTTGGAAACGGATGCTAATACCGGATATT
ATGCTTTCTTCGCATGGAGGAAGTATGAAAGCTATATGCGCTATGAGAGAGCTTTGCGTCCCATTAGCTA
GTTGGTGAGGTAACGGCCCACCAAGGCGATGATGGGTAGCCGGCCTGAGAGGGTGAACGGCCACAAGGGG
ACTGAGACACGGCCCTTACTCCTACGGGAGGCAGCAGTGGGGAATATTGGACAATGGACCAAAAGTCTGA
TCCAGCAATTCTGTGTGCACGATGACGTTTTTCGGAATGTAAAGTGCTTTCAGTCGGGAAGAAGCAAGTG
ACGGTACCGACAGAAGAAGCGACGGCTAAATACGTGCCAGCAGCCGCGGTAATACGTATGTCGCAAGCGT

TATCCGGATTTATTGGGCGTAAAGCGCGTCTAGGCGGCAAGGAAAGTCTGATGTGAAAATGCGGGGCTCA
ACTCCGTATTGCGTTGGAAACTGCCTTACTAGAGTACTGGAGAGGTAGGCGGAACTACAAGTGTAGAGGT
GAAATTCGTAGATATTTGTAGGAATGCCGATGGGGAAGCCAGCCTACTGGACAGATACTGACGCTAAAGC
GCGAAAGCGTGGGTAGCAAACAGGATTAGATACCCTGGTAGTCCACGCTGTAAACGATGATTACTAGGTG
TTGGGGGTCAAACCTCAGCGCCCAAGCTAACGCGATAAGTAATCCGCCTGGGGAGTACGTACGCAAGTAT
GAAACTCAAAGGAATTGACGGGGACCCGCACAAGCGGTGGAGCATGTGGTTTAATTCGACGCAACGCGAG
GAACCTTACCAGCGTTTGACATCCTACAAAGAGTGCAGAGATGCGCTTGTGCTTCTTCGGAAGAATGTAG
TGACAGGTGGTGCATGGCTGTCGTCAGCTCGTGTCGTGAGATGTTGGGTTAAGTCCCGCAACGAGCGCAA
CCCCTATCGTATGTTACCAGCCTTTAGTTGGGGACTCATGCGATACTGCCTGCGACGAGCAGGAGGAAGG
TGGGGATGACGTCAAGTCATCATGCCCCTTATACGCTGGGCTACACACGTGCTACAATGGGTAGTACAGA
GAGCGGCGAACCCGCGAGGGGGAGCAAATCTCAGAAAACTATTCTTAGTTCGGATTGTACTCTGCAACTC
GAGTACATGAAGTTGGAATCGCTAGTAATCGCAAATCAGCAATGTTGCGGTGAATACGTTCTCGGGTCTT
GTACACACCGCCCGTCACACCACGAGAGTTGGTTGCACCTGAAGTAGCAGGCCTAACCGTAAGGAAGGAT
GCTCCGAGGGTGTGGTTAGCGATTGGGGTGAAGTCGTAACAAGGTA
>343201461|ref|NR_042187.1|Cloacibacterium|Cloacibacterium normanense strain :CCUG 46293 16S ribosomal RNA, partial sequence
AGCGGGAGGCCTAACACATGCAAGCCGAGCGGTATTGTTTCTTCGGAAATGAGAGAGCGGCGTACGGGTG
CGGAACACGTGTGCAACCTGCCTTTATCTGGGGGATAGCCTTTCGAAAGGAAGATTAATACTCCATAACA
TATTGATTGGCATCAATTAATATTGAAAGCTCCGGCGGATAGAGATGGGCACGCGCAAGATTAGCTAGTT
GGTGAGGTAACGGCTCACCAAGGCGATGATCTTTAGGGGGCCTGAGAGGGTGATCCCCCACACTGGTACT
GAGACACGGACCAGACTCCTACGGGAGGCAGCAGTGAGGAATATTGGTCAATGGGTGCAAGCCTGAACCA
GCCATCCCGCGTGAAGGACGACTGCCCTATGGGTTGTAAACTTCTTTTGTATAGGGATAAACCTACCCTC
GTGAGGGTAGCTGAAGGTACTATACGAATAAGCACCGGCTAACTCCGTGCCAGCAGCCGCGGTAATACGG
AGGGTGCAAGCGTTATCCGGATTTATTGGGTTTAAAGGGTCCGTAGGCGGACTTATAAGTCAGTGGTGAA
AGCCTGTCGCTTAACGATAGAACTGCCATTGATACTGTAAGTCTTGAGTATATTTGAGGTAGCTGGAATA
AGTAGTGTAGCGGTGAAATGCATAGATATTACTTAGAACACCAATTGCGAAGGCAGGTTACCAAGATATA
ACTGACGCTGAGGGACGAAAGCGTGGGGAGCGAACAGGATTAGATACCCTGGTAGTCCACGCCGTAAACG
ATGCTAACTCGTTTTTGGGCTTTAGGGTTCAGAGACCAAGCGAAAGTGATAAGTTAGCCACCTGGGGAGT
ACGCTCGCAAGAGTGAAACTCAAAGGAATTGACGGGGGCCCGCACAAGCGGTGGATTATGTGGTTTAATT
CGATGATACGCGAGGAACCTTACCAAGACTTAAATGGGAATTGACAGTTTTAGAAATAGAACTTTCTTCG
GACAATTTTCAAGGTGCTGCATGGTTGTCGTCAGCTCGTGCCGTGAGGTGTTAGGTTAAGTCCTGCAACG
AGCGCAACCCCTGTCACTAGTTGCCATCATTCAGTTGGGGACTCTAGTGAGACTGCCTACGCAAGTAGAG
AGGAAGGTGGGGATGACGTCAAATCATCACGGCCCTTACGTCTTGGGCCACACACGTAATACAATGGCCG
GTACAGAGGGCAGCTACACAGCGATGTGATGCAAATCTCGAAAGCCGGTCTCAGTTCGGATTGGAGTCTG
CAACTCGACTCTATGAAGCTGGAATCGCTAGTAATCGCGCATCAGCCATGGCGCGGTGAATACGTTCCCG
GGCCTTGTACACACCGCCCGTCAAGCCATGGAAGCTGGGGGTACCTGAAGTCGGTGACCGTAAAAGGAGC
TGCCTAGGGTAAAACTAGTAACTAGGGCTAAGTCGTAACAAGGTAGCCGTACCGGAAGGTGCGGCTGGAT
CACCT
>456680|gb|L08642.1|U.urealyticum|Ureaplasma urealyticum 16S ribosomal RNA
TAGAATCCGTCAATTTTTAAAGAGTTTGATCCTGGCTCAGGATTAACGCTGGCGGCATGCCTAATACATG
CAAATCGAACGAAGCCTTTTAGGCTTAGTGGTGAACGGGTGAGTAACACGTATCCAATCTACCCTTAAGT
TGGGGATAACTAGTCGAAAGATTAGCTAATACCGAATAATAACATCAATATCGCATGAGAAGATGTAGAA
AGTCGCTCTTTGTGGCGACGCTTTTGGATGAGGGTGCGACGTATCAGATAGTTGGTGAGGTAATGGCTCA
CCAAGTCAATGACGCGTAGCTGTACTGAGAGGTAGAACAGCCACAATGGGACTGAGACACGGCCCATACT
CCTACGGGAGGCAGCAGTAGGGAATTTTTCACAATGGGCGCAAGCCTTATGAAGCAATGCCGCGTGAACG
ATGAAGGTCTTATAGATTGTAAAGTTCTTTTATATGGGAAGAAACGCTAAGATAGGAAATGATTTTAGTT
TGACTGTACCATTTGAATAAGTATCGGCTAACTATGTGCCAGCAGCCGCGGTAATACATAGGATGCAAGC
GTTATCCGGATTTACTGGGCGTAAAACGAGCGCAGGCGGGTTTGTAAGTTTGGTATTAAATCTAGATGCT
TAACGTCTAGCTGTATCAAAAACTGTAAACCTAGAGTGTAGTAGGGAGTTGGGGAACTCCATGTGGAGCG
GTAAAATGCGTAGATATATGGAAGAACACCGGTGGCGAAGGCGCCAACTTGGACTATCACTGACGCTTAG
GCTCGAAAGTGTGGGGAGCAAATAGGATTAGATACCCTAGTAGTCCACACCGTAAACGATCATCATTAAA
TGTCGGCCCGAATGGGTCGGTGTTGTAGCTAACGCATTAAATGATGTGCCTGGGTAGTACATTCGCAAGA
ATGAAACTCAAACGGAATTGACGGGGACCCGCACAAGTGGTGGAGCATGTTGCTTAATTTGACAATACAC

GTAGAACCTTACCTAGGTTTGACATCTATTGCGATGCTATAGAAATATAGTTGAGGTTAACAATATGACA
GGTGGTGCATGGTTGTCGTCAGCTCGTGTCGTGAGATGTTGGGTTAAGTCCGCAACGAGCGCAACCCCTT
TCGTTAGTTACTTTTCTAGCGATACTGCTACCGCAAGGTAGAGGAAGGTGGGGATGACGTCAAATCATCA
TGCCCCTTATATCTAGGGCTGCAAACGTGCTACAATGGCTAATACAAACTGCTGCAAAATCGTAAGATGA
AGCGAAACAGAAAAAGTTAGTCTCAGTTCGGATAGAGGGCTGCAATTCGTCCTCTTGAAGTTGGAATCAC
TAGTAATCGCGAATCAGACATGTCGCGGTGAATACGTTCTCGGGTCTTGTACACACCGCCCGTCAAACTA
TGGGAGCTGGTAATATCTAAAACCGGCAAAGCTAACCTTTTGGAGGCATGCGTCTAGGGTAGGATCGGTG
ACTGGAGTTAAGTCGTAACAAGGTATCCCTACGAGAACGTGGGGATGGATCACCTCCTTTCTTCGGAGTA
AATTTTTAATTTACGTACTAATTAGTGTACATTTTTTTAAAATCC
>1808583|dbj|D79212.1|L.paracasei|Lactobacillus paracasei subsp.
paracasei gene for 16S rRNA, partial sequence
GATGAACGCTGGCGGCGTGCCTAATACATGCAAGTCGAACGAGTTCTCGTTGATGATCGGTGCTTGCACC
GAGATTCAACATGGAACGAGTGGCGGACGGGTGAGTAACACGTGGGTAACCTGCCCTTAAGTGGGGGATA
ACATTTGGAAACAGATGCTAATACCGCATAGATCCAAGAACCGCATGGTTCTTGGCTGAAAGATGGCGTA
AGCTATCGCTTTTGGATGGACCCGCGGCGTATTAGCTAGTTGGTGAGGTAATGGCTCACCAAGGCGATGA
TACGTAGCCGAACTGAGAGGTTGATCGGCCACATTGGGACTGAGACACGGCCCAAACTCCTACGGGAGGC
AGCAGTAGGGAATCTTCCACAATGGACGCAAGTCTGATGGAGCAACGCCGCGTGAGTGAAGAAGGCTTTC
GGGTCGTAAAACTCTGTTGTTGGAGAAGAATGGTCGGCAGAGTAACTGTTGTCGGCGTGACGGTATCCAA
CCAGAAAGCCACGGCTAACTACGTGCCAGCAGCCGCGGTAATACGTAGGTGGCAAGCGTTATCCGGATTT
ATTGGGCGTAAAGCGAGCGCAGGCGGTTTTTTAAGTCTGATGTGAAAGCCCTCGGCTTAACCGAGGAAGC
GCATCGGAAACTGGGAAACTTGAGTGCAGAAGAGGACAGTGGAACTCCATGTGTAGCGGTGAAATGCGTA
GATATATGGAAGAACACCAGTGGCGAAGGCGGCTGTCTGGTCTGTAACTGACGCTGAGGCTCGAAAGCAT
GGGTAGCGAACAGGATTAGATACCCTGGTAGTCCATGCCGTAAACGATGAATGCTAGGTGTTGGAGGGTT
TCCGCCCTTCAGTGCCGCAGCTAACGCATTAAGCATTCCGCCTGGGGAGTACGACCGCAAGGTTGAAACT
CAAAGGAATTGACGGGGGCCCGCACAAGCGGTGGAGCATGTGGTTTAATTCGAAGCAACGCGAAGAACCT
TACCAGGTCTTGACATCTTTTGATCACCTGAGAGATCAGGTTTCCCCTTCGGGGGCAAAATGACAGGTGG
TGCATGGTTGTCGTCAGCTCGTGTCGTGAGATGTTGGGTTAAGTCCCGCAACGAGCGCAACCCTTATGAC
TAGTTGCCAGCATTTAGTTGGGCACTCTAGTAAGACTGCCGGTGACAAACCGGAGGAAGGTGGGGATGAC
GTCAAATCATCATGCCCCTTATGACCTGGGCTACACACGTGCTACAATGGATGGTACAACGAGTTGCGAG
ACCGCGAGGTCAAGCTAATCTCTTAAAGCCATTCTCAGTTCGGACTGTAGGCTGCAACTCGCCTACACGA
AGTCGGAATCGCTAGTAATCGCGGATCAGCACGCCGCGGTGAATACGTTCCCGGGCCTTGTACACACCGC
CCGTCACACCATGAGAGTTTGTAACACCCGAAGCCGGTGGCGTAACCCTTTTAGGGAGCGAGCCGTCTAA
GGTGGGACAAATGATTAGGGTGAAGTCGTAACAAGGTAGCCGTAGGAGAACC
>265678780|ref|NR_029085.1|L.saerimneri|Lactobacillus saerimneri strain
GDA154 16S ribosomal RNA, partial sequence
AGAGTTGATCCTGGCTCAGGATGAACGCTGGCGGCGTGCCTAATACATGCAAGTCGAGCGCATCGGCCCA
ACTGATTGAAGATGCTTGCATCCGATTGACGATGGTTTACCGATGAGCGGCGGACGGGTGAGTAACACGT
AGGTAACCTGCCCAGAAGCGGGGGATAACACCTGGAAACAGATGCTAATACCGCATAGGTCATTTGACCG
CATGGTCAAATGATTAAAGATGGCTCTGCTATCACTTCTGGATGGACCTGCGGCGTATTAGCTAGTTGGT
AAGGTAACGGCTTACCAAGGCGATGATACGTAGCCGAGTTGAGAGACTGATCGGCCACATTGGGACTGAG
ACACGGCCCAGACTCCTACGGGAGGCAGCAGTAGGGAATCTTCCACAATGGACGCAAGTCTGATGGAGCA
ACGCCGCGTGAGCGAAGAAGGTCTTCGGATCGTAAAACTCTGTTGTTAGAGAAGAACACGGGTGAGAGTA
ACTGTTCACCTGTTGACGGTATCTAACCAGCAAGTCACGGCTAACTACGTGCCAGCAGCCGCGGTAATAC
GTAGGTGGCAAGCGTTATCCGGATTTATTGGGCGTAAAGGGAACGCAGGCGGTTCTTTAAGTCTGATGTG
AAAGCCTTCGGCTTAACCGAAGATGTGCATTGGAAACTGGGGAACTTGAGTGCAGAAGAGGAGAGTGGAA
CTCCATGTGTAGCGGTGAAATGCGTAGATATATGGAAGAACACCAGTGGCGAAAGCGGCTCTCTGGTCTG
TAACTGACGCTGAGGTTCGAAAGCGTGGGTAGCGAACAGGATTAGATACCCTGGTAGTCCACGCCGTAAA
CGATGAATGCTAGGTGTTGGAGGGTTTCCGCCCTTCAGTGCCGCAGCTAACGCACTAAGCATTCCGCCTG
GGGAGTACGACCGCAAGGTTGAAACTCAAAGGAATTGACGGGGGCCCGCACAAGCGGTGGAGCATGTGGT
TTAATTCGAAGCTACGCGAAGAACCTTACCAGGTCTTGACATCTTTTGACCACCTAAGAGATTAGGTTTT
CCCTTCGGGGACAAAATGACAGGTGGTGCATGGTTGTCGTCAGCTCGTGTCGTGAGATGTTGGGTTAAGT
CCCGCAACGAGCGCAACCCTTGTTGTCAGTTGCCAGCATTCAGTTGGGCACTCTGGCGAGACTGCCGGTG
ACAAACCGGAGGAAGGTGGGGATGACGTCAAATCATCATGCCCCTTATGACCTGGGCTACACACGTGCTA
CAATGGGCAGTACAACGAGTCGCGAAACCGCGAGGTTTAGCAAATCTCTTAAAGCTGCTCTCAGTTCGGA

CTGTAGGCTGCAACTCGCCTRCACGAAGTCGGAATCGCTAGTAATCGCGAATCAGCATGTCGCGGTGAAT
ACGTTCCCGGGCCTTGTACACACCGCCCGTCACACCATGAGAGTTTGTAACACCCAAAGCCGGTGGGGTA
ACCTTTTGGAGCCAGCCGTCTAAGGTGGGACAGATGATTGGGGTGAAGTCGTAACAAGGTAGCCGTAGGA
GAACCTGCGGCTGGATCACCTCCTTTCT
>37655076|gb|AY391826.1|L.saerimneri|Lactobacillus sp. T059 16S ribosomal RNA gene, partial sequence
AGAGTTTGATCCTGGCTCAGGATGAACGCTGGCGGCGTGCCTAATACATGCAAGTCGAGCGCATCGGCCC
AACTGATTGAAGATGCTTGCATCCGATTGACGATGGTTTACCGATGAGCGGCGGACGGGTGAGTAACACG
TAGGTAACCTGCCCAGAAGCGGGGGATAACACCTGGAAACAGATGCTAATACCGCATAGGTCATTTGACC
GCATGGTCAAATGATTAAAGATGGCTCTGCTATCACTTCTGGATGGACCTGCGGCGTATTAGCTAGTTGG
TAAGGTAACGGCTTACCAAGGCGATGATACGTAGCCGAGTTGAGAGACTGATCGGCCACATTGGGACTGA
GACACGGCCCAGACTCCTACGGGAGGCAGCAGTAGGGAATCTTCCACAATGGACGCAAGTCTGATGGAGC
AACGCCGCGTGAGCGAAGAAGGTCTTCGGATCGTAAAACTCTGTTGTTAGAGAAGAACACGGGTGAGAGT
AACTGTTCACCTGTTGACGGTATCTAACCAGCAAGTCACGGCTAACTACGTGCCAGCAGCCGCGGTAATA
CGTAGGTGGCAAGCGTTATCCGGATTTATTGGGCGTAAAGGGAACGCAGGCGGTTCTTTAAGTCTGATGT
GAAAGCCTTCGGCTTAACCGAAGATGTGCATTGGAAACTGGGGAACTTGAGTGCAGAAGAGGAGAGTGGA
ACTCCATGTGTAGCGGTGAAATGCGTAGATATATGGAAGAACACCAGTGGCGAAAGCGGCTCTCTGGTCT
GTAACTGACGCTGAGGTTCGAAAGCGTGGGTAGCGAACAGGATTAGATACCCTGGTAGTCCACGCCGTAA
ACGATGAATGCTAGGTGTTGGAGGGTTTCCGCCCTTCAGTGCCGCAGCTAACGCACTAAGCATTCCGCCT
GGGGAGTACGACCGCAAGGTTGAAACTCAAAGGAATTGACGGGGGCCCGCACAAGCGGTGGAGCATGTGG
TTTAATTCGAAGCTACGCGAAGAACCTTACCAGGTCTTGACATCTTTTGACCACCTAAGAGATTAGGTTT
TCCCTTCGGGGACAAAATGACAGGTGGTGCATGGTTGTCGTCAGCTCGTGTCGTGAGATGTTGGGTTAAG
TCCCGCAACGAGCGCAACCCTTGTTGTCAGTTGCCAGCATTCAGTTGGGCACTCTGGCGAGACTGCCGGT
GACAAACCGGAGGAAGGTGGGGATGACGTCAAATCATCATGCCCCTTATGACCTGGGCTACACACGTGCT
ACAATGGGCAGTACAACGAGTCGCGAAACCGCGAGGTTTAGCAAATCTCTTAAAGCTGCTCTCAGTTCGG
ACTGCAGGCTGCAACTCGCCTGCACGAAGTCGGAATCGCTAGTAATCGCGAATCAGCATGTCGCGGTGAA
TACGTTCCCGGGCCTTGTACACACCGCCCGTCACACCATGAGAGTTTGTAACACCCAAAGCCGGTGGGGT
AACCTTTTGGAGCCAGCCGTCTAAGGTGGGACAGATGATTGGGGTGAAGTCGTAACAAGGTAACC
>66878779|gb|AY959073.1|P.bivia|94%Uncultured bacterium clone rRNA300 16S ribosomal RNA gene, partial sequence
GCCCTTAGAGTTTGATCCTGGCTCAGGATGAACGCTAGCTATAGGCTTAACACATGCAAGTCGAGGGGCA
GCGAATAGATGGCTTGCTATTTATGTCGGCGACCGGCGCACGGGTGAGTAACGCGTATCCAACCTGCCCA
TAACTAAGGGATAACCCAGCGAAAGTTGGACTAATACCTTATGTATTCGTTTGATCTCATGAGATTACGA
ATAAAGATTTATCGGTTATGGATGGGGATGCGTCTGATTAGCTTGTTGGCGGGGTAACGGCCCACCAAGG
CAACGATCAGTAGGGGTTCTGAGAGGAAGGTCCCCCACATTGGAACTGAGACACGGTCCAAACTCCTACG
GGAGGCAGCAGTGAGGAATATTGGTCAATGGACGCAAGTCTGAACCAGCCAAGTAGCGTGCAGGATGACG
GCCCTATGGGTTGTAAACTGCTTTTATATGGGGATAAAGTGGGGAACGTGTTCCCTTTTGCAGGTACCAT
ATGAATAAGGACCGGCTAATTCCGTGCCAGCAGCCGCGGTAATACGGAAGGTTCGGGCGTTATCCGGATT
TATTGGGTTTAAAGGGAGCGTAGGCCGTTTGGTAAGCGTGTTGTGAAATGTAGTAGCTCAACTTCTAGAT
TGCAGCGCGAACTGTCAGACTTGAGTGCGCACAACGTAGGCGGAATTCATGGTGTAGCGGTGAAATGCTC
AGATATCATGAAGAACTCCGATTGCGAAGGCAGCTTACGGGAGCGCAACTGACGCTGAAGCTCGAAGGTG
CGGGTATCGAACAGGATTAGATACCCTGGTAGTCCGCACAGTAAACGATGGATGCCCGCTGTTAGCACCT
AGTGTTAGCGGCTAAGCGAAAGCATTAAGCATCCCACCTGGGGGAGTACGCCGGCAACGGTGAAACTCAA
AGGAATTGGCGGGGCCCCGCACAAGCGGCGGGTCATGTGGTTTAATTCGATGATACGCGACGAACCTTGC
CCGGGCTTGAATTGCAGATGAACGATTTAGAGATAATGAGGTCCTTCGGGACATCTGTGAAGGTGCTGCA
TGGTTGTCGTCAGCTGGTGCCGTGAGGTGTCGGATTAAGTACCATAACGAGCGCCCCCCTTTTTTTTAGT
CCTCATCAGGTTTTGGTGCGCTCTGTGGAGATACCCCCCCTGTAAGGTGTGAGGAAGGTGGGGATGGTGT
CAAATCAGCGCGCCCCTTATCTCCGGGGCCACACGCGTGTCACAATGGGTGGCACAGATAGTTTGTGTGT
ATGCAAATCCTATTTTCTCCTTAAACCCATTTCCAGTTCGGATTGGGTTTTCCACCCCGCCCCCCCGAAG
CTGGATTCGTTAGTATTCGGCCATCACCCATGGCGCGGTGAATCGGTTCCGGGCCTTGTGTCCCCCCCCC
CCGTCAACCCATGAAACCGGGGGGTCCTTGAAGTTCGTGCCCGTAAGGATCGACCTAGGGCAAAACTGGT
AATTGGGGCTAA
>302129311|dbj|AB547673.1|P.bivia| Prevotella bivia gene for 16S ribosomal RNA, partial sequence, strain: JCM 6331

AGAGTTTGATCCTGGCTCAGGATGAACGCTAGCTATAGGCTTAACACATGCAAGTCGAGGGGCAGCGAAT
AGATAGCTTGCTATTTATGTCGGCGACCGGCGCACGGGTGAGTAACGCGTATCCAACCTACCCATAACTA
AGGGATAACCCAGCGAAAGTTGGACTAATACCTTATGTATTCGTTTGATCTCATGAGATTACGAATAAAG
ATTTATCGGTTATGGATGGGGATGCGTCTGATTAGCTTGTTGGCGGGGTAACGGCCCACCAAGGCAACGA
TCAGTAGGGGTTCTGAGAGGAAGGTCCCCCACATTGGAACTGAGACACGGTCCAAACTCCTACGGGAGGC
AGCAGTGAGGAATATTGGTCAATGGACGCAAGTCTGAACCAGCCAAGTAGCGTGCAGGATGACGGCCCTA
TGGGTTGTAAACTGCTTTTATATGGGGATAAAGTGGGAACGTGTTCCCTTTTGCAGGTACCATATGAAT
AAGGACCGGCTAATTCCGTGCCAGCAGCCGCGGTAATACGGAAGGTTCGGGCGTTATCCGGATTTATTGG
GTTTAAAGGGAGCGTAGGCCGTTTGGTAAGCGTGTTGTGAAATGTAGGAGCTCAACTTCTAGATTGCAGC
GCGAACTGTCAGACTTGAGTGCGCACAACGTAGGCGGAATTCATGGTGTAGCGGTGAAATGCTTAGATAT
CATGAAGAACTCCGATTGCGAAGGCAGCTTACGGGAGCGCAACTGACGCTGAAGCTCGAAGGTGCGGGTA
TCGAACAGGATTAGATACCCTGGTAGTCCGCACAGTAAACGATGGATGCCCGCTGTTAGCACCTAGTGTT
AGCGGCTAAGCGAAAGCATTAAGCATCCCACCTGGGGAGTACGCCGGCAACGGTGAAACTCAAAGGAATT
GACGGGGGCCCGCACAAGCGGAGGAACATGTGGTTTAATTCGATGATACGCGAGGAACCTTACCCGGGCT
TGAATTGCAGATGAACGATTTAGAGATAATGAGGTCCTTCGGGACATCTGTGAAGGTGCTGCATGGTTGT
CGTCAGCTCGTGCCGTGAGGTGTCGGCTTAAGTGCCATAACGAGCGCAACCCCTTTCTTTAGTTGCCATC
AGGTTCTGCTGGGCACTCTGGAGATACTGCCACCGTAAGGTGTGAGGAAGGTGGGGATGACGTCAAATCA
GCACGGCCCTTACGTCCGGGGCTACACACGTGTTACAATGGGTGGTACAGATAGTTGGTCGTGTGCAAAT
ACGATCTAATCCTTAAAACCATTCTCAGTTCGGACTGGGGTCTGCAACCCGACCCCACGAAGCTGGATTC
GCTAGTAATCGCGCATCAGCCATGGCGCGGTGAATACGTTCCCGGGCCTTGTACACACCGCCCGTCAAGC
CATGAAAGCCGGGGGTGCCTGAAGTTCGTGACCGTAAGGATCGACCTAGGGCAAAACTGGTAATTGGGGC
TAAGTCGTAACAAGGTAACC
>345296748|gb|HQ219658.1|S.sanguinis|Streptococcus sanguinis 16S ribosomal RNA gene, partial sequence
GCAAGTAGGACGCACAGTTTATACCGTAGCTTGCTACACCATAGACTGTGAGTTGCGAACGGGTGAGTAA
CGCGTAGGTAACCTGCCTATTAGAGGGGGATAACTATTGGAAACGATAGCTAATACCGCATAACAGTATG
TAACACATGTTAGATGCTTGAAAGATGCAATTGCATCGCTAGTAGATGGACCTGCGTTGTATTAGCTAGT
AGGTAGGGTAATGGCCTACCTAGGCGACGATACATAGCCGACCTGAGAGGGTGATCGGCCACACTGGGAC
TGAGACACGGCCCAGACTCCTACGGGAGGCAGCAGTAGGGAATCTTCGGCAATGGGGGGAACCCTGACCG
AGCAACGCCGCGTGAGTGAAGAAGGTTTTCGGATCGTAAAGCTCTGTTGTTAAGGAAGAACGAGTGTGAG
AATGGAAAGTTCATACTGTGACGGTACTTAACCAGAAAGGGACGGCTAACTACGTGCCAGCAGCCGCGGT
AATACGTAGGTCCCGAGCGTTGTCCGGATTTATTGGGCGTAAAGCGAGCGCAGGCGGTTAGAAAAGTCTG
AAGTGAAAGGCAGTGGCTCAACCATTGTAGGCTTTGGAAACTGTTTAACTTGAGTGCAGAAGGGGAGAGT
GGAATTCCATGTGTAGCGGTGAAATGCGTAGATATATGGAGGAACACCGGTGGCGAAAGCGGCTCTCTGG
TCTGTAACTGACGCTGAGGCTCGAAAGCGTGGGGAGCGAACAGGATTAGATACCCTGGTAGTCCACGCCG
TAAACGATGAGTGCTAGGTGTTAGGTCCTTTCCGGGACTTAGTGCCGCAGCTAACGCATTAAGCACTCCG
CCTGGGGAGTACGACCGCAAGGTTGAAACTCAAAGGAATTGACGGGGGCCCGCACAAGCGGTGGAGCATG
TGGTTTAATTCGAAGCAACGCGAAGAACCTTACCAGGTCTTGACATCCCGATGCTATTTCTAGAGATAGG
AAGTTTCTTCGGAACATCGGTGACAGGTGGTGCATGGTTGTCGTCAGCTCGTGTCGTGAGATGTTGGGTT
AAGTCCCGCAACGAGCGCAACCCTTATTGTTAGTTGCCATCATTGAGTTGGGCACTCTAGCGAGACTGCC
GGTAATAAACCGGAGGAAGGTGGGGATGACGTCAAATCATCATGCCCCTTATGACCTGGGCTACACACGT
GCTACAATGGCTGGTACAACGAGTCGCAAGCCGGTGACGGCAAGCTAATCTCTGAAAGCCAGTCTCAGTT
CGGATTGTAGGCTGCAACTCGCCTACATGAAGTCGGAATCGCTAGTAATCGCGGATCAGCACGCCGCGGT
GAATACGTTCCCGGGCCTTGTACACACCGCCCGTCACACCACGAGAGTTTGTAACACCCGAAGTCGGTGA
GGTAACCGTAA
>66878628|gb|AY958922.1|Lactobacillus|Uncultured bacterium clone rRNA149 16S ribosomal RNA gene, partial sequence
CCCTTAGAGTTTGATCCTGGCTCAGGACGAACGCTGGCGGCGTGCCTAATACATGCAAGTCGAGCGAGTC
TGCCTTGAAGATCGGAGTGCTTGCACTCTGTGAAACAAGATACAGGCTAGCGGCGGACGGGTGAGTAACA
CGTGGGTAACCTGCCCAAGAGATCGGGATAACACCTGGAAACAGATGCTAATACCGGATAACAACAGATG
ATGCCTATCAACTGTTTAAAAGATGGTTCTGCTATCACTCTTGGATGGACCTGCGGTGCATTAGCTAGTT
GGTAGGGTAACGGCCTACCAAGGCGATGATGCATAGCCGAGTTGAGAGACTGATCGGCCACATTGGGACT
GAGACACGGCCCAAACTCCTACGGGAGGCAGCAGTAGGGAATCTTCCACAATGGACGCAAGTCTGATGGA
GCAACGCCGCGTGAGTGAAGAAGGGTTTCGGCTCGTAAAGCTCTGTTGTTGGTGAAGAAGGACAGGGGTA

GTAACTGACCTTTGTTTGACGGTAATCAATTAGAAAGTCACGGCTAACTACGTGCCAGCAGCCGCGGTAA
TACGTAGGTGGCAAGCGTTGTCCGGATTTATTGGGCGTAAAGCGAGTGCAGGCGGCTCGATAAGTCTGAT
GTGAAAGCCTTCGGCTCAACCGGAGAATTGCATCAGAAACTGTCGAGCTTGAGTACAGAAGAGGAGAGTG
GAACTCCATGTGTAGCGGTGAAATGCGTAGATATATGGAAGAACACCGGTGGCGAAGGCGACCACCTGGA
CTAATACTGACACTGAGGTGCGAAAGCGTGGGGAGCAAACAGGATTAGATACCCTGGTAGTCCACGCCGT
AAACGATGTCAACTAGCCGTTGGAAGCCTTGAGCTTTTAGTGGCGCAGCTAACGCATTAAGTTGACCGCC
TGGGGAGTACGGCCGCAAGGTTAAAACTCAAATGAATTGACGGGGGCCCGCACAAGCGGTGGAGCATGTG
GTTTAATTCGAAGCAACGCGAAGAACCTTACCAGGCCTTGACATCCAATGAACTTTCTAGAGATAGATTG
GTGCCTTCGGGAACATTGAGACAGGTGCTGCATGGCTGTCGTCAGCTCGTGTCGTGAGATGTTGGGTTAA
GTCCCGTAACGAGCGCAACCCTTGTCCTTAGTTACCAGCACGTCATGGTGGGCACTCTAAGGAGACTGCC
GGTGACAAACCGGAGGAAGGTGGGGATGACGTCAAGTCATCATGGCCCTTACGGCCTGGGCTACACACGT
GCTACAATGGTCGGTACAGAGGGTTGCCAAGCCGCGAGGTGGAGCTAATCCCACAAAACCGATCGTAGTC
CGGATCGCAGTCTGCAACTCGACTGCGTGAAGTCGGAATCGCTAGTAATCGCGAATCAGAATGTCGCGGT
GAATACGTTCCCGGGCCTTGTACACACCGCCCGTCACACCATGGGAGTGGGTTGCACCAGAAGTAGCTAG
TCTAACCTTCGGGAGGACGGTTACCACGGTGTGATTCATGACTGGGGTGAA
>78033738|emb|AM111051.1|Carnobacterium|Carnobacterium sp. 7196 partial
16S rRNA gene
AGAGTTTGATCCTGGCTCAGGACGAACGCTGGCGGCATGCCTAATACATGCAAGTCGAACGCTTTTGTTT
CACCGGGTGCTTGCACCCACCGAGACAAAAGAGTGGCGGACGGGTGAGTAACACGTGGGTAACCTGCCCA
TAAGAGGGGGATAACATCCGGAAACGGATGCTAATACCGCATATTTCCAATTGTCTCCTGACAGATGGAA
AAAAGGTGGCTTCGGCTACCGCTTATGGATGGACCCGCGGCGTATTAGCTAGTTGGTGAGGTAATGGCTC
ACCAAGGCGATGATACGTAGCCGACCTGAGAGGGTGATCGGCCACACTGGGACTGAGACACGGCCCAGAC
TCCTACGGGAGGCAGCAGTAGGGAATCTTCCGCAATGGACGAAAGTCTGACGGAGCAATGCCGCGTGAG
TGAAGAAGGTTTTCGGATCGTAAAACTCTGTTGTTAGAGAAGAACAAGGATGAGAGTAACTGCTCATCCC
CCTGACGGTATCTAACCAGAAAGCCACGGCTAACTACGTGCCAGCAGCCGCGGTAATACGTAGGTGGCAA
GCGTTGTCCGGATTTATTGGGCGTAAAGCGAGCGCAGGCGGTTCTTTAAGTCTGATGTGAAAGCCCCCG
GCTCAACCGGGGAGGGTCATTTGGAAACTGGAGAACTTGAGTGCAGAAAGAGGAGAGTGGAATTCCACGT
GTAGCGGTGAAATGCGTAGATATGTGGAGGAACACCAGTGGCGAAGGCGACTCTCTGGTCTGTAACTGAC
GCTGAGGCTCGAAAGCGTGGGGAGCAAACAGGATTAGATACCCTGGTAGTCCACGCCGTAAACGATGAGT
GCTAAGTGTTGGAGGGTTTCCGCCCTTCAGTGCTGCAGCTAACGCATTAAGCACTCCGCCTGGGGAGTAC
GACCGCAAGGTTGAAACTCAAAGGAATTGACGGGGACCCGCACAAGCGGTGGAGCATGTGGTTTAATTCG
AAGCAACGCGAAGAACCTTACCAGGTCTTGACATCCTTTGACCACTCTAGAGATAGAGCTTTCCCTTCGG
GGACAAAGTGACAGGTGGTGCATGGTTGTCGTCAGCTCGTGTCGTGAGAGTGGTAGGTTAAGTCCCGCAA
CGAGCGCAACCCTTGTCCTTAGTTACCAGCGGTTTGGCCGGGAACTCTAGTGAGACTGCCAGTGACAAAC
TGGAGGAAGGCGGGGACGACGTCAAGTCCCCATGGCCCTTACGACCAGGGCTACACCGTGCTACAATGGA
TGGTACAGAGGGCAGCTAGGTCGCGATGCCATGCGAATCTCTTAAAGCCATTCGTAGTCCGGATTGGAGT
CTGCCACTCGACTCCATGAAGTAGGAATAGCTAGTAATCGCGGATCATAATGCCGCGGTGAATACGTTCC
CGGGCCTTGTACACACCGCCCGTAACACCATGGGAGTTTGTAACACCTGAAGTCGGTGAGGTAACCCTTT
TGGGAGCCAGCCGCCACGGTGTGACAGATGACTGGGGTGAAGTCGTAACAAGGTAACC
>AE014295.3|159243-160772|AE014295.3|B.longum|Bifidobacterium longum
NCC2705, complete genome
TTTTTGTGGAGGGTTCGATTCTGGCTCAGGATGAACGCTGGCGGCGTGCTTAACACATGCAAGTCGAACG
GGATCCATCAGGCTTTGCTTGGTGGTGAGAGTGGCGAACGGGTGAGTAATGCGTGACCGACCTGCCCCAT
ACACCGGAATAGCTCCTGGAAACGGGTGGTAATGCCGGATGCTCCAGTTGATCGCATGGTCTTCTGGGAA
AGCTTTCGCGGTATGGGATGGGGTCGCGTCCTATCAGCTTGACGGCGGGGTAACGGCCCACCGTGGCTTC
GACGGGTAGCCGGCCTGAGAGGGCGACCGGCCACATTGGGACTGAGATACGGCCCAGACTCCTACGGGAG
GCAGCAGTGGGGAATATTGCACAATGGGCGCAAGCCTGATGCAGCGACGCCGCGTGAGGGATGGAGGCCT
TCGGGTTGTAAACCTCTTTTATCGGGGAGCAAGCGAGAGTGAGTTTACCCGTTGAATAAGCACCGGCTAA
CTACGTGCCAGCAGCCGCGGTAATACGTAGGGTGCAAGCGTTATCCGGAATTATTGGGCGTAAAGGGCTC
GTAGGCGGTTCGTCGCGTCCGGTGTGAAAGTCCATCGCTTAACGGTGGATCCGCGCCGGGTACGGGCGGG
CTTGAGTGCGGTAGGGGAGACTGGAATTCCCGGTGTAACGGTGGAATGTGTAGATATCGGGAAGAACACC
AATGGCGAAGGCAGGTCTCTGGGCCGTTACTGACGCTGAGGAGCGAAAGCGTGGGGAGCGAACAGGATTA
GATACCCTGGTAGTCCACGCCGTAAACGGTGGATGCTGGATGTGGGGCCCGTTCCACGGGTTCCGTGTCG
GAGCTAACGCGTTAAGCATCCCGCCTGGGGAGTACGGCCGCAAGGCTAAAACTCAAAGAAATTGACGGGG

GCCCGCACAAGCGGCGGAGCATGCGGATTAATTCGATGCAACGCGAAGAACCTTACCTGGGCTTGACATG
TTCCCGACGGTCGTAGAGATACGGCTTCCCTTCGGGGCGGGTTCACAGGTGGTGCATGGTCGTCGTCAGC
TCGTGTCGTGAGATGTTGGGTTAAGTCCCGCAACGAGCGCAACCCTCGCCCCGTGTTGCCAGCGGATTAT
GCCGGGAACTCACGGGGGACCGCCGGGGTTAACTCGGAGGAAGGTGGGGATGACGTCAGATCATCATGCC
CCTTACGTCCAGGGCTTCACGCATGCTACAATGGCCGGTACAACGGGATGCGACGCGGCGACGCGGAGCG
GATCCCTGAAAACCGGTCTCAGTTCGGATCGCAGTCTGCAACTCGACTGCGTGAAGGCGGAGTCGCTAGT
AATCGCGAATCAGCAACGTCGCGGTGAATGCGTTCCCGGGCCTTGTACACACCGCCCGTCAAGTCATGAA
AGTGGGCAGCACCCGAAGCCGGTGGCCTAACCCCTTGTGGGATGGAGCCGTCTAAGGTGAGGCTCGTGAT
TGGGACTAAGTCGTAACAAGGTAGCCGTACCGGAAGGTGCGGCTGGATCACCTCCTTTCT
>110436448|gb|DQ796048.1|P.copri|P. copri 88%Uncultured bacterium (fecal) clone RL202_aai50g03 16S ribosomal RNA gene, partial sequence
AGAGTTTGATCCTGGCTCAGGATGAACGCTAGCTACAGGCTTAACACATGCAAGTCGAGGGGAAACGATA
TTGGAAGCTTGCTTCCGATGGGCGTCGACCGGCGCACGGGTGAGTAACGCGTATCCAACCTGCCCATCAC
TTGGGGATAACCTTGCGAAAGTAAGACTAATACCCAATGATATCTCAAGAAGACATCTGAATGAGATTAA
AGATTTATCGGTGATGGATGGGGATGCGTCTGATTAGCTTGTTGGCGGGGTAACGGCCCACCAAGGCTAC
GATCAGTAGGGGTTCTGAGAGGAAGGTCCCCCACATTGGAACTGAGACACGGTCCAAACTCCTACGGGAG
GCAGCAGTGAGGAATATTGGTCAATGGGCGAGAGCCTGAACCAGCCAAGTAGCGTGCAGGATGACGGCCC
TATGGGTTGTAAACTGCTTTTATAAGGGAATAAAGTGAGCCTCGTGAGGCTTTTTGCATGTACCTTATGA
ATAAGGACCGGCTAATTCCGTGCCAGCAGCCGCGGTAATACGGAAGGTCCGGGCGTTATCCGGATTTATT
GGGTTTAAAGGGAGCGTAGGCCGGAGATTAAGCGTGTTGTGAAATGTAGATGCTCAACATCTGCACTGCA
GCGCGAACTGGTTTCCTTGAGTACGCACAAAGTGGGCGGAATTCGTGGTGTAGCGGTGAAATGCTTAGAT
ATCACGAAGAACTCCGATTGCGAAGGCAGCTCACTGGAGCGCAACTGACGCTGAAGCTCGAAAGTGCGGG
TATCGAACAGGATTAGATACCCTGGTAGTCCACACTGTAAACGATGAATGCTAGGTTTAGGGGGTATCGA
CCCCTTCTGTGCCGCAGTCAACACAATAAGCATTCCGCCTGGGGAGTACGGCCGCAAGGTTGAAACTCAA
AGGAATTGACGGGGGCCCGCACAAGCAGCGGAGCATGTGGTTTAATTCGACGCAACGCGAAGAACCTTAC
CAGGTCTTGACATCCACTTAAACTTACAGAGAAGTAAGGTGTGCTTGCACAAAGTGAGACAGGTGGTGCA
TGGTTGTCGTCAGCTCGTGTCGTGAGATGTTGGGTTAAGTCCCGCAACGAGCGCAACCCTTATCTTCAGT
TACTAACGCGTAAAGGTGAGGACTCTGAAGAGACTGCCGGGGACAACTCGGAGGAAGGTGGGGACGACGT
CAAATCATCATGCCCCTTATGACCTGGGCTACACACGTGCTACAATGGCCACGACAGAGAGAAGCGAAAT
CGTAAGGTAGAGCGGAACTCAAAAAAGTGGTCCCAGTTCGGATTGTGGGCTGCAACCCGCCCACATGAAG
TCGGAGTTGCTAGTAATCGCGGATCAGCATGCCGCGGTGAATACGTTCCCGGGCCTTGCACACACCGCCC
GTCAAG
>4406233|gb|AF104671.1|S.intermedius|Streptococcus intermedius strain ATCC27335 16S ribosomal RNA gene, partial sequence
TTTGATCCTGGTTCAGGACGAACGCTGGCGGCGTGCCTAATACATGCAAGTAGAACGCACAGGATGCACC
GTAGTTTACTACACCGTATTCTGTGAGTTGCGAACGGGTGAGTAACGCGTAGGTAACCTGCCTGGTAGCG
GGGGATAACTATTGGAAACGATAGCTAATACCGCATAAGAACATTTACTGCATGGTAGATGTTTAAAAGG
TGCAAATGCATCACTACCAGATGGACCTGCGTTGTATTAGCTAGTAGGTGAGGTAACGGCTCACCTAGGC
GACGATACATAGCCGACCTGAGAGGGTGATCGGCCACACTGGGACTGAGACACGGCCCAGACTCCTACGG
GAGGCAGCAGTAGGGAATCTTCGGCAATGGGGGGAACCCTGACCGAGCAACGCCGCGTGAGTGAAGAAGG
TTTTCGGATCGTAAAGCTCTGTTGTTAAGGAAGAACGAGTGTGAGAATGGAAAGTTCATACTGTGACGGT
ACTTAACCAGAAAGGGACGGCTAACTACGTGCCAGCAGCCGCGGTAATACGTAGGTCCCGAGCGTTGTCC
GGATTTATTGGGCGTAAAGCGAGCGCAGGCGGTTAGATAAGTCTGAAGTTAAAGGCAGTGGCTCAACCAT
TGTAGGCTTTGGAAACTGTTTAACTTGAGTGCAGAAGGGGAGAGTGGAATTCCATGTGTAGCGGTGAAAT
GCGTAGATATATGGAGGAACACCGGTGGCGAAAGCGGCTCTCTGGTCTGTAACTGACGCTGAGGCTCGAA
AGCGTGGGGAGCGAACAGGATTAGATACCCTGGTAGTCCACGCCGTAAACGATGAGTGCTAGGTGTTAGG
TCCTTTCCGGGACTTAGTGCCGCAGCTAACGCATTAAGCACTCCGCCTGGGGAGTACGACCGCAAGGTTG
AAACTCAAAGGAATTGACGGGGGCCCGCACAAGCGGTGGAGCATGTGGTTTAATTCGAAGCAACGCGAAG
AACCTTACCAGGTCTTGACATCCCGATGCCCGCTCTAGAGATAGAGCTTTACTTCGGTACATCGGTGACA
GGTGGTGCATGGTTGTCGTCAGCTCGTGTCGTGAGATGTTGGGTTAAGTCCCGCAACGAGCGCAACCCTT
ATTGTTAGTTGCCATCATTCAGTTGGGCACTCTAGCGAGACTGCCGGTAATAAACCGGAGGAAGGTGGGG
ATGACGTCAAATCATCATGCCCCTTATGACCTGGGCTACACACGTGCTACAATGGCTGGTACAACGAGTC
GCAAGCCGGTGACGGCAAGCTAATCTCTGAAAGCCAGTCTCAGTTCGGATTGTAGGCTGCAACTCGCCTA
CATGAAGTCGGAATCGCTAGTAATCGCGGATCAGCACGCCGCGGTGAATACGTTCCCGGGCCTTGTACAC

ACCGCCCGTCACACCACGAGAGTTTGTAACACCCGAAGTCGGTGAGGTAACCGTAAGGAGCCAGCCGCCT
AAGGTGGGATAGATGATTGGGGTGAAGTCGTAACAAGGTAGCCGTATCGGAAGGTGCGGCTGGATCACCT
CCTTGGTCATAGCTGTTT
>2290269|gb|U88435.1|Rahnella|RSU88435 Rahnella sp. 'CDC 21234' 16S ribosomal RNA gene, partial sequence
AGATTTGATCCTGGCTCAGATTGAACGCTGGCGGCAGGCCTAACACATGCAAGTCGAGCGGCAGCGGGAA
GTAGCTTGCTACTTTGCCGGCGAGCGGCGGACGGGTGAGTAATGTCTGGGAAACTGCCTGATGGAGGGGG
ATAACTACTGGAAACGGTAGCTAATACCGCATGACCTCGCAAGACCAAAGTGGGGGACCTTCGGGCCTCA
CGCCATCGGATGTGCCCAGATGGGATTAGCTAGTAGGTGAGGTAATGGCTCACCTAGGCGACGATCCCTA
GCTGGTCTGAGAGGATGACCAGCCACACTGGAACTGAGACACGGTCCAGACTCCTACGGGAGGCAGCAGT
GGGGAATATTGCACAATGGGCGCAAGCCTGATGCAGCCATGCCGCGTGTGTGAAGAAGGCCTTAGGGTTG
TAAAGCACTTTCAGCGAGGAGGAAGGGTTCAGTGTTAATAGCACTGAACATTGACGTTACTCGCAGAAGA
AGCACCGGCTAACTCCGTGCCAGCAGCCGCGGTAATACGGAGGGTGCAAGCGTTAATCGGAATTACTGGG
CGTAAAGCGCACGCAGGCGGTTTGTTAAGTCAGATGTGAAATCCCCGAGCTTAACTTGGGAACTGCATTT
GAAACTGGCAAGCTAGAGTCTTGTAGAGGGGGGTAGAATTCCAGGTGTAGCGGTGAAATGCGTAGAGATC
TGGAGGAATACCGGTGGCGAAGGCGGCCCCCTGGACAAAGACTGACGCTCAGGTGCGAAAGCGTGGGGAG
CAAACAGGATTAGATACCCTGGTAGTCCACGCTGTAAACGATGTCGACTTGGAGGTTGTGCCCTTGAGGC
GTGGCTTCCGGAGCTAACGCGTTAAGTCGACCGCCTGGGGAGTACGGCCGCAAGGTTAAAACTCAAATGA
ATTGACGGGGGCCCGCACAAGCGGTGGAGCATGTGGTTTAATTCGATGCAACGCGAAGAACCTTACCTAC
TCTTGACATCCAGAGAATTCGCTAGAGATAGCTTAGTGCCTTCGGGAACTCTGAGACAGGTGCTGCATGG
CTGTCGTCAGCTCGTGTTGTGAAATGTTGGGTTAAGTCCCGCAACGAGCGCAACCCTTATCCTTTGTTGC
CAGCACGTAATGGTGGGAACTCAAAGGAGACTGCCGGTGATAAACCGGAGGAAGGTGGGGATGACGTCAA
GTCATCATGGCCCTTACGAGTAGGGCTACACACGTGCTACAATGGCATATACAAAGAGAAGCAAACTCGC
GAGAGCAAGCGGACCTCATAAAGTATGTCGTAGTCCGGATTGGAGTCTGCAACTCGACTCCATGAAGTCG
GAATCGCTAGTAATCGTAGATCAGAATGCTACGGTGAATACGTTCCCGGGCCTTGTACACACCGCCCGTC
ACACCATGGGAGTGGGTTGCAAAAGAAGTAGGTAGCTTAACCTTCGGGAGGGCGCTTACCACTTTGTGAT
TCATGACTGGGGTGAAGTCGTAACAAGGTAACCGTAGGGGAACCTGCGGCTGGATCACCTCC
>110189035|gb|DQ819169.1|E.coli|Uncultured bacterium clone aab26f06 16S ribosomal RNA gene, partial sequence (mouse-cecal)
AGAGTTTGATCCTGGGTCAGATTGAACGCTGGCGGCAGGCCTAACACATGCAAGTCGAACGGTAACAGGA
AGAAGCTTGCTTCTTTGCTGACGAGTGGCGGACGGGTGAGTAATGTCTGGGAAACTGCCTGATGGAGGGG
GATAACTACTGGAAACGGTAGCTAATACCGCATAACGTCGCAAGACCAAAGAGGGGGACCTTCGGGCCTC
TTGCCATCGGATGTGCCCAGATGGGATTAGCTAGTAGGTGGGGTAACGGCTCACCTAGGCGACGATCCCT
AGCTGGTCTGAGAGGATGACCAGCCACACTGGAACTGAGACACGGTCCAGACTCCTACGGGAGGCAGCAG
TGGGGAATATTGCACAATGGGCGCAAGCCTGATGCAGCCATGCCGCGTGTATGAAGAAGGCCTTCGGGTT
GTAAAGTACTTTCAGCGGGGAGGAAGGGAGTAAAGTTAATACCTTTGCTCATTGACGTTACCCGCAGAAG
AAGCACCGGCTAACTCCGTGCCAGCAGCCGCGGTAATACGGAGGGTGCAAGCGTTAATCGGAATTACTGG
GCGTAAAGCGCACGCAGGCGGTTTGTTAAGTCAGATGTGAAATCCCCGGGCTCAACCTGGGAACTGCATC
TGATACTGGCAAGCTTGAGTCTCGTAGAGGGGGGTAGAATTCCAGGTGTAGCGGTGAAATGCGTAGAGAT
ATGGAGGAACACCAGTGGCGAAGGCGGCTCTCTGGTCTGTAACTGACGCTGATGTGCGAAAGCGTGGGGA
TCAAACAGGATTAGATACCCTGGTAGTCCACGCCGTAAACGATGAGTGCTAAGTGTTAGGGGGGTTTCCG
CCCCTTAGTGCTGCAGCTAACGCATTAAGCACTCCGCCTGGGGAGTACGACCGCAAGGTTGAAACTCAAA
GGAATTGACGGGGACCCGCACAAGCGGTGGAGCATGTGGTTTAATTCGAAGCAACGCGAAGAACCTTACC
AAATCTTGACATCCTTTGACCGCTCTAGAGATAGAGTTTTCCCCTTCGGGGGACAAAGTGACAGGTGGTG
CATGGTTGTCGTCAGCTCGTGTCGTGAGATGTTGGGTTAAGTCCCGCAACGAGCGCAACCCTTAAGCTTA
GTTGCCATCATTAAGTTGGGCACTCTAGGTTGACTGCCGGTGACAAACCGGAGGAAGGTGGGGATGACGT
CAAATCATCATGCCCCTTATGATTTGGGCTACACACGTGCTACAATGGATAATACAAAGGGCAGCGAACC
CGCGAGGTCAAGCAAATCCCATAAAATTATTCTCAGTTCGGATTGTAGTCTGCAACTCGACTACATGAAG
CTGGAATCGCTAGTAATCGTAGATCAGCATGCTACGGTGAATACGTTCCCGGGTCTTGTACACACCGCCC
GTCACACCACGAGAGTTTGTAACACCCGAAGCCGGTGGAGTAACCTT
>66878717|gb|AY959011.1|E.coli|Uncultured bacterium clone rRNA238 16S ribosomal RNA gene, partial sequence
TCGCCCTTAGAGTTTGATCCTGGCTCAGATTGAACGCTGGCGGCAGGCCTAACACATGCAAGTCGAACGG
TAACAGGAATCAGCTTGCTGATTCGCTGACGAGTGGCGGACGGGTGAGTAATGTCTGGGAAACTGCCTGA

TGGAGGGGGATAACTACTGGAAACGGTAGCTAATACCGCATAACGTCGCAAGACCAAAGAGGGGGACCTT
CGGGCCTCTTGCCATCGGATGTGCCCAGATGGGATTAGCTAGTAGGTGGGGTAAAGGCTCACCTAGGCGA
CGATCCCTAGCTGGTCTGAGAGGATGACCAGCCACACTGGAACTGAGACACGGTCCAGACTCCTACGGGA
GGCAGCAGTGGGGAATATTGCACAATGGGCGCAAGCCTGATGCAGCCATGCCGCGTGTCTGAAGAAGGCC
TTCGGGTTGTAAAGTACTTTCAGCGGGGAGGAAGGGAGTAAAGTTAATACCTTTGCTCATTGACGTTACC
CGCAGAAGAAGCACCGGCTAACTCCGTGCCAGCAGCCGCGGTAATACGGAGGGTGCAAGCGTTAATCGGA
ATTACTGGGCGTAAAGCGCACGTAGGCGGTTTGTTAAGTCAGATGTGAAATCCCCGGGCTCAACCTGGGA
ACTGCATCTGATACTGGCAAGCTTGAGTCTCGTAGAGGGGGGGTAGAATTCCAGGTGTAGCGGTGAAATG
CGTAGAGATCTGGAGGAATACCGGTGGCGAAGGCGGCTCTCTGGTCTGTAACTGACGCTGAGGCTCGAAA
GCGTGGGGAGCAAACAGGATTAGATACCCTGGTAGTCCACGCCGTAAACGATGAGTGCTAAGTGTTGGAG
GGTTTCCGCCCTTCAGTGCTGCAGCAAACGCATTAAGCACTCCGCCTGGGGAGTACGACCGCAAGGTTGA
AACTCAAAGGAATTGACGGGGGCCCGCACAAGCGGTGGAGCATGTGGTTTAATTCGAAGCAACGCGAAGA
ACCTTACCAGGTCTTGACATCCTTTGACCACTCTAGAGATGGAGCTTTCCCTTCGGGGACAAAGTGACAG
GTGGTGCATGGTTGTCGTCAGCTCGTGTCGTGAGATGTTGGGTTAAGTCCCGCAACGAGCGCAACCCTTA
TTGTTAGTTGCCATCATTTAGTTGGGCACTCTAGCGAGACTGCCGGTGACAGACCGGAGGAAGGTGGGGA
TGACGTCGAATCATCATGCCCCTTATGACCTGGGCTACACACGTGCTACAATGGGAAGTACAACGAGTCG
CTAGACCGCGAGGTCATGCAAATCTCTTAAAGCTTCTCTCAGTTCGGATTGCAGGCTGCAACTCGCCTGC
ATGAAGCCGGAATCGCTAGTAATCGCGGATCAGCACGCCGCGGTGAATACGTTCCCGGGCCTTGTACACA
CCGCCCGTCACACCACGAGAGTTTGTAACACCCGAAGTCGGTGAGGTAACCTTTTTGGAGCCAGCCGCCT
AAGGTGGGATAGATGATTGGGGTGAA
>4730879|dbj|D89332.1|Scardovia|Scardovia inopinata gene for 16S rRNA, partial sequence, strain: DSM 10107
TTTCGATTCTGGCTCAGGATGAACGCTGGCGGCGTGCTTAACACATGCAAGTCGAACGGATCCTGCTTG
GTTTTTGCTGGGTGGGTGAGAGTGGCGAACGGGTGAGTAATGCGTGACTAACCTGCCGTGTAGTTGGGTA
TAGCTCCTGGAAACGGGTGGTAATACCGAATGGTCCACTGGGCTGCATGGTCTGGTGGGAAAGTTTTGTG
TGCTATGCGATGGGGTCGCGTCCTATCAGCTTGTTGGTGGGGTGATGGCCTACCAAGGCTTCGACGGGTA
GCCGGCCTAAGAGGGCGACCGGCCACATTGGGACTGAGATACGGCCCAGACTCCTACGGGAGGCAGCAGT
GGGGAATATTGCACAATGGGGGGAACCCTGATGCAGCGACGCCGCGTGCGGGATGGAGGCCTTCGGGTTG
TGAACCGCTTTTATTGGGGAGCAAGCTGGCCCTGTGTGGGTTGGTGAGTGTACTTGGTGAATAAGCACCG
GCTAACTACGTGCCAGCAGCCGCGGTAATACGTAGGGTGCAAGCGTTGTCCGGATTTATTGGGCGTAAAG
GGCTCGTAGGCGGTTCGTCGCGTCTGGTGTGAAAGCTTACTGCTTAACGGTGGGTTTGCGTTGGATACGG
GCGGGCTTGAGTGCAGTAGGGGAGACTGGAATTCTCGGTGTAACGGTGGAATGTGTAGATATCGGGAAGA
ACACCTATGGCGAAGGCAGGTCTCTGGGCTGTTACTGACGCTGAGGAGCGAAAGCGTGGGGAGCGAACAG
GATTAGATACCCTGGTAGTCCACGCTGTAAACGGTGGACGCTGGATGTGGGGCCTATTTCCACGGGTTCT
GTGTCGGAGCTAACGCGTTAAGCGTCCCGCCTGGGGAGTACGGCCGCAAGGTTAAAACTCAAAGAAATTG
ACGGGGGCCCGCACAAGCGGCGGAGCATGCGGATTAATTCGATGCAACGCGAAGAACCTTACCAGGGCTT
GACATGTGAGCGTTGATCTTAGAGATAGGGTTTCCCTTCGGGGCGCTTTCACAGGTGGTGCATGGTCGTC
GTCAGCTCGTGTCGTGAGATGTTGGGTTAAGTCCCGCAACGAGCGCAACCCTTGCCTTGTGTTGCCAGCG
GGTCGTGCCGGGGACTCACAAGGGACCGCCGGGGTTAACTCGGAGGAAGGTGGGGATGACGTCAGATCAT
CATGCCCCTTACGTCCTGGGCTTCACGCATGCTACAATGGCTGGTACAGCGGGATGCGATACGGTGACGT
GGAGCGGATCCTGTAAAACCGGTCTCAGTTCGGATCGGAGCCTGCAACTCGGCTCCGTGAAGGTGGAGTC
GCTAGTAATCGCGGATCAGCAGTGCCGCGGTGAATGCGTTCCCGGGCCTTGTACACACCGCCCGTCAAGT
CATGAAAGTGGGCAGCACCCGAAGCCGGTGGCCTAACCCTTGTGGGGAGAGCCGTCTAAGGTGAGGTTCG
CGATTGGGACTAAGTCGTAACAAGGTAGCCGTACCGGAAGGTGCGGCTGGATCACCTCCTTA
>255708358|dbj|AB480776.1|E.coli|Escherichia coli gene for 16S ribosomal RNA, partial sequence, strain: Acj 212
GAGAGTTTGATCCTGGCTCAGATTGAACGCTGGCGGCAGGCCTAACACATGCAAGTCGAACGGTAACAGG
AAGAAGCTTGCTTCTTTGCTGACGAGTGGCGGACGGGTGAGTAATGTCTGGGAAACTGCCTGATGGAGGG
GGATAACTACTGGAAACGGTAGCTAATACCGCATAACGTCGCAAGACCAAAGAGGGGGACCTTCGGGCCT
CTTGCCATCGGATGTGCCCAGATGGGATTAGCTAGTAGGTGGGGTAACGGCTCACCTAGGCGACGATCCC
TAGCTGGTCTGAGAGGATGACCAGCCACACTGGAACTGAGACACGGTCCAGACTCCTACGGGAGGCAGCA
GTGGGGAATATTGCACAATGGGCGCAAGCCTGATGCAGCCATGCCGCGTGTATGAAGAAGGCCTTCGGGT
TGTAAAGTACTTTCAGCGGGGAGGAAGGGAGTAAAGTTAATACCTTTGCTCATTGACGTTACCCGCAGAA
GAAGCACCGGCTAACTCCGTGCCAGCAGCCGCGGTAATACGGAGGGTGCAAGCGTTAATCGGAATTACTG

GGCGTAAAGCGCACGCAGGCGGTTTGTTAAGTCAGATGTGAAATCCCCGGGCTCAACCTGGGAACTGCAT
CTGATACTGGCAAGCTTGAGTCTCGTAGAGGGGGGTAGAATTCCAGGTGTAGCGGTGAAATGCGTAGAGA
TCTGGAGGAATACCGGTGGCGAAGGCGGCCCCCTGGACGAAGACTGACGCTCAGGTGCGAAAGCGTGGGG
AGCAAACAGGATTAGATACCCTGGTAGTCCACGCCGTAAACGATGTCGACTTGGAGGTTGTGCCCTTGAG
GCGTGGCTTCCGGAGCTAACGCGTTAAGTCGACCGCCTGGGGAGTACGGCCGCAAGGTTAAAACTCAAAT
GAATTGACGGGGGCCCGCACAAGCGGTGGAGCATGTGGTTTAATTCGATGCAACGCGAAGAACCTTACCT
GGTCTTGACATCCACAGAACTTTCCAGAGATGGATTGGTGCCTTCGGGAACTGTGAGACAGGTGCTGCAT
GGCTGTCGTCAGCTCGTGTTGTGAAATGTTGGGTTAAGTCCCGCAACGAGCGCAACCCTTATCCTTTGTT
GCCAGCGGTCCGGCCGGGAACTCAAAGGAGACTGCCAGTGATAAACTGGAGGAAGGTGGGGATGACGTCA
AGTCATCATGGCCCTTACGACCAGGGCTACACACGTGCTACAATGGCGCATACAAAGAGAAGCGACCTCG
CGAGAGCAAGCGGACCTCATAAAGTGCGTCGTAGTCCGGATTGGAGTCTGCAACTCGACTCCATGAAGTC
GGAATCGCTAGTAATCGTGGATCAGAATGCCACGGTGAATACGTTCCCGGGCCTTGTACACACCGCCCGT
CACACCATGGGAGTGGGTTGCAAAAGAAGTAGGTAGCTTAACCTTCGGGAGGGCGCTTACCACTTTGTGA
TTCATGACTGGGGTGAAGTCGTAACAAGGTAGCCGTAG
>8038004|gb|AF257096.1|L.crispatus|Lactobacillus crispatus strain
ATCC33197 16S ribosomal RNA gene, partial sequence
GACGAACGCTGGCGGCGTGCCTAATACATGCAAGTCGAGCGAGCGGAACTAACAGATTTACTTCGGTAAT
GACGTTAGGAAAGCGAGCGGCGGATGGGTGAGTAACACGTGGGGAACCTGCCCCATAGTCTGGGATACCA
CTTGGAAACAGGTGCTAATACCGGATAAGAAAGCAGATCGCATGATCAGCTTTTAAAAGGCGGCGTAAGC
TGTCGCTATGGGATGACCCCGCGGTGCATTAGCTAGTTGGTAAGGCAAAGGCTTACCAAGGCGATGATGC
ATAGCCGAGTTGAGAGACTGATCGGCCACATTGGGACTGAGACACGGCCCAAACTCCTACGGGAGGCAGC
AGTAGGGAATCTTCCACAATGGACGCAAGTCTGATGGAGCAACGCCGCGTGAGTGAAGAAGGTTTTCGGA
TCGTAAAGCTCTGTTGTTGGTGAAGAAGGATAGAGGTAGTAACTGGCCTTTATTTGACGGTAATCAACCA
GAAAGTCACGGCTAACTACGTGCCAGCAGCCGCGGTAATACGTAGGTGGCAAGCGTTGTCCGGATTTATT
GGGCGTAAAGCGAGCGCAGGCGGAAGAATAAGTCTGATGTGAAAGCCCTCGGCTTAACCGAGGAACTGCA
TCGGAAACTGTTTTTCTTGAGTGCAGAAGAGGAGAGTGGAACTCCATGTGTAGCGGTGGAATGCGTAGAT
ATATGGAAGAACACCAGTGGCGAAGGCGGCTCTCTGGTCTGCAACTGACGCTGAGGCTCGAAAGCATGGG
TAGCGAACAGGATTAGATACCCTGGTAGTCCATGCCGTAAACGATGAGTGCTAAGTGTTGGGAGGTTTCC
GCCTCTCAGTGCTGCAGCTAACGCATTAAGCACTCCGCCTGGGGAGTACGACCGCAAGGTTGAAACTCAA
AGGAATTGACGGGGGCCCGCACAAGCGGTGGAGCATGTGGTTTAATTCGAAGCAACGCGAAGAACCTTAC
CAGGTCTTGACATCTAGTGCCATTTGTAGAGATACAAAGTTCCCTTCGGGGACGCTAAGACAGGTGGTGC
ATGGCTGTCGTCAGCTCGTGTCGTGAGATGTTGGGTTAAGTCCCGCAACGAGCGCAACCCTTGTTATTAG
TTGCCAGCATTAAGTTGGGCACTCTAATGAGACTGCCGGTGACAAACCGGAGGAAGGTGGGGATGACGTC
AAGTCATCATGCCCCTTATGACCTGGGCTACACACGTGCTACAATGGGCAGTACAACGAGAAGCGAGCCT
GCGAAGGCAAGCGAATCTCTGAAAGCTGTTCTCAGTTCGGACTGCAGTCTGCAACTCGACTGCACGAAGC
TGGAATCGCTAGTAATCGCGGATCAGCACGCCGCGGTGAATACGTTCCCGGGCCTTGTACACACCGCCCG
TCACACCATGGGAGTCTGCAATGCCCAAAGCCGGTGGCCTAACCTTCGGGAAGGAGCCGTCTAAGGCAGG
GCAGATGACTGGGGTGAAGTCGTAACAAGGTAGCCGTAGGAGAACTGC
>18958005|gb|AY078425.1|L.amnionii|Leptotrichia amnionii 16S ribosomal
RNA gene, partial sequence
GGTGATCCAGCCGCACCTTCCGGTACGGATACCTTGTTCCNNCTCCACCCCAATCACTATCCACACCTTC
AGTACCTCCCTCCTTACGGTTAGGCCGGTAATTTCAGGTGCAAACAACTCTCGTGGTGTGACGGGCGGTG
TGTACAAGGCCCGAGAACGTATTCACCGTGACATTGCTGATTCACGATTACTAGTGATTCCAACTTCATG
AAGTCGAGTTGCAGACTTCAATCCGAACTAAGAATAGCTTTTTAAGTTTCGCCATGTATCGCTACAAAGC
TTCTCTTTGTACTACCCATTGTAGCACGTGTGTAGCCCAGATCATAAGGGGCATGATGACTTGACGTCAT
CCCCACCTTCCTCCTACTCTTCGTAGGCAGTTTCATTAGAGTCCCCAACTTAATGATGGCAACTAATGAT
AGGGGTTTCGCTCGTTGCGGGACTTAACCCAACATCTCACAACACGAGCTGTCGACAGCCATGCACCACC
TGTCTCTCGGTTCCCGAAGGCACAAGTATACTTCTATACTCTCCCGAGGATGTCAAGATCTGGTAAGGTT
CCTCGCGTTGCGTCGAATTAAACCACATGCTCCACCACTTGTGCGGGCCCCCGTCAATTCCTTTGAGTTT
CATTCTTGCGAACGTACTCCCCAGGCGGATCACTTATCGCTTTTGCTTCGGCACAGAGACTTCGCATCCC
CACACCCAGTGATCATCGTTTACAGCTAGGACTACCAGGGTATCTAATCCTGTTTGCTCCCCTAGCTTTC
GCACTTCAGCGTCAGTTTCTGTCCAGTGAGTTATCTTCATCATCGGCATTCCTACACATATCTACGAATT
TCACCTCTACTCGTGTAGTTCCACCCACCTTTCCAGTACTCTAGTTCAACAGTTTTGTAGGCAAGCCTAT
GGTTGAGCCACAGGTTTTCACCTTCAACTTGTCTTACCGCCTAGATGCCCTTTAAGCCCAATAATTCCGG

ATAACGCTCGCNACATACGTATTACCGCGGCTGCTGGCACGTATTTAGCCGTTGCTTCTTCTGTAGGTAC
CGTCATTTTTTTCTTCCCTACTAAAAGTGTTTTACAGTCCTAAAACCTTCTTCACACACACAGAATTGCT
GGATCAGAGTTCCCTCCATTGTCCAATATTCCCCACTGCTGCCTCCCGTAGGAGTAAGGGCCGTATCTCA
GTCCCCTTGTGGCCGTCCACCCTCTCAGGCCGGCTACCTATCATCGCCTTGGTAAGCTCTTACCTTACCA
ACTAGCTAATAGGACGCAAAGCTCTCCTTTAGTGCCGTAGCTTTCATTTGCATATCATGCGATATACTAA
CTTATCCAGTATTATCAGAAGTTTCCCCCTGTTATCCCAGTCTAAAGGGCAAGTTCTTTACGCGTTACTC
ACCCGTCCGCCATGAGTCCATTTAGCAAGCTAAACTTCCTCATAGACTTGCATGTGTTAAGCATTCTGTC
AGCGTTTATCCTGAGCCAGGATC
>27530269|dbj|AB089057.1|Mycoplasma|Uncultured Mycoplasma sp. gene for 16S rRNA, partial sequence, clone: Rs-E42
GATTAACGCTAGCGGTATGCCTAATACATGCAAGTCAAACGGCCAGCAATGGCAGTGGCAAACGGGTGAG
TAATACGTATCTAATCTACCCTTTAGAGAAGAATAACTGATCGAAAGATTAGCTAATACTTCATAGGAAA
TTAGTTCACATGAATTAATTTTTAAAGCTCCGTCTGGAGCGCTTTAGGATGAGGGTGCGGCTTATCAGAT
AGTTGGTGAGGTAACGGCCCACCAAGTCAATGACGAGTAGCTATGCTGAGAGGTAGAATAGCCACAATGG
AACTGAGAAACGGTCCATACTCCTACGGGAGGCAGCAGTAGGGAATTTTTCACAATGGGCGAAAGCCTGA
TGGAGCAATACCGCGTGGATGATGAAGGTCTTTGGATTGTAAAATCCTTTTATTAGGGACGAACGATACT
AGTAGGAAATGATTAGTATTTGACTGTACCTTTTGAATAAGTAACGGCAAACTATGTGCCAGCAGCCGCG
GTAATACATAGGTTACAAGCGTTATCCGGATTTACTGGGCGTAAAGCGAGCGCGGGCTGATTTACAAGTC
TGGTGTGAAATGCATTTGCTTAACAAATGTTTGCATTGGAAACTGTAAGTCTAGAGTATGATAGAGAGTT
TTAGAACTCCATGTGGAGCGGTGGAATGCGTAGATATATGGAAGAATACCAGTGGTGAAGACGAAAACTT
AGGTCATAACTGACGCTTAGGCTCGAAAGTGTGGGGAGCAAATAGGATTAGATACCCTAGTAGTCCACAC
TGTAAACGATGATCATTAGGTGTTGGTATCACTATACCAGCGCCGAAGCTAACGCATTAAATGATCCGCC
TGGGTAGTACATTCGCAAGAATGAAACTCAAACGGAATTGACGGGGACCCGCACAAGTGGTGGAGCATGT
TGTTTAATTCGACATCACACGCAAAACCTTACCAGGGTTTGACATATCTGGCAATGCTATAGAAATATAG
CGGAGGTTAACCGGAATACAGGTGGTGCATGGTTGTCGTCAGCTCGTGTCGTGAGATGTTGGGTTAAGTC
CCGCAACGAGCGCAACCCTTATTGCTAGTTACTTTATCTAGCGAGACTGCTAACGCAAGTTAGAGGAAGG
TGGGGATGACGTCAAATCATCATGCCCTTTATACCCTGGGCTACAAACGTGCTACAATGGTCGGTACAAA
CTGTCGCGAAGCTGTAAAGTGGAGCCAATCAGAAAAAGCCGATCTCAGTTCAGATCGAGGGCTGCAATTC
GTCCTCGTGAAGTTGGAATCACTAGTAATCGCGAATAAGTCATGTCGCGGTGAATACGTTCTCGGGTCTT
GTACACACCGCCCGTCAAACTATGAGAGCCGTTAATACCTAAAACCGTTTTGCTAACCGCAAGGAGGCGA
ACGTCTAGGGTAGGAATGGTGATTGGAGTT
>343202531|ref|NR_042880.1|Pasteurella|Pasteurella bettyae strain CCUG 2042 16S ribosomal RNA, partial sequence
ATTGAACGCTGGCGGCAGGCTTAACACATGCAAGTCGAACGGTAGCACAGGGGAGCTTGCTCCCTGGGTG
ACGAGTGGCGGACGGGTGAGTAATGCTTGGGAATTTGGCTTRTGGAGGGGGATAACTACGGGAAACTGTA
GCTAATACCGCGTAATGTCTACGGACTAAAGGGTGGGACTATTTGGCCACCTGCCATGAGATGAGCCCAA
GTGGGATTAGGTAGTTGGTGGGGTAAAGGCCTACCAAGCCGACGATCTCTAGCTGGTCTGAGAGGATGAC
CAGCCACACTGGAACTGAGACACGGTCCAGACTCCTACGGGAGGCAGCAGTGGGGAATATTGCACAATGG
GGGGAACCCTGATGCAGCCATGCCGCGTGAATGAAGAAGGCCTTCGGGTTGTAAAGTTCTTTCGGTGATG
AGGAAGGCAGGTATTTTAATAGAATACTTGATTGACGTTAATCACAGAAGAAGCACCGGCTAACTCCGTG
CCAGCAGCCGCGGTAATACGGAGGGTGCGAGCGTTAATCGGAATAACTGGGCGTAAAGGGCATGCAGGCG
GTTGTTTAAGTGAGATGTGAAAGCCCCGGGCTTAACCTGGGAATTGCATTTCAGACTGGACAGCTAGAGT
ACTTTAGGGAGGGGTAGAATTCCACGTGTAGCGGTGAAATGCGTAGAGATGTGGAGGAATACCGAAGGCG
AAGGCAGCCCCTTGGGAATGTACTGACGCTCATATGCGAAAGCGTGGGGAGCAAACAGGATTAGATACCC
TGGTAGTCCACGCTGTAAACGATGTCGATTTGGGGATTGAGCAATAAGCTTGGTGCTCGAAGCTAACGTG
ATAAATCGACCGCCTGGGGAGTACGGCCGCAAGGTTAAAACTCAAATGAATTGACGGGGGCCCGCACAAG
CGGTGGAGCATGTGGTTTAATTCGATGCAACGCGAAGAACCTTACCTACTCTTGACATCCATAGAAGAAC
TCAGAGATGAGTTTGTGCCTTCGGGAGCTATGAGACAGGTGCTGCATGGCTGTCGTCAGCTCGTGTTGTG
AAATGTTGGGTTAAGTCCCGCAACGAGCGCAACCCTTATCCTTTGTTGCCAGCGACATGGTCGGGAACTC
AAAGGAGACTGCCAGTGATAAACTGGAGGAAGGTGGGGATGACGTCAAGTCATCATGGCCCTTACGAGTA
GGGCTACACACGTGCTACAATGGTGCATACAGAGGGAAGCGAGCCTGCGAGGGGGAGCGAATCTCAGAAA
GTGCATCTAAGTCCGGATTGGAGTCTGCAACTCGACTCCATGAAGTCGGAATCGCTAGTAATCGCAAATC
AGAATGTTGCGGTGAATACGTTCCCGGGCCTT

>38260405|gb|AY362929.1|Actinobacillus|Actinobacillus arthritidis strain CCUG 24862 16S ribosomal RNA gene, partial sequence
ATTGAACGCTGGCGGCAGGCTTAACACATGCAAGTCGAACGGTAACAGGGATTAGCTTGCTAATTTGCTG
ACGAGTGGCGGACGGGTGAGTAATGCTTGGGAATCTGGCTCATGGAGGGGGATAACTACGGGAAACTGTA
GCTAATACCGCGTAATATCTTCGGATTAAAGTGTGGGACCGCAAGGCCACATGCCATGAGATGAGCCCAA
GTGGGATTAGGTAGTTGGTGGGGTAAAGGCCTACCAAGCCGACGATCTCTAGCTGGTCTGAGAGGATGAC
CAGCCACACTGGAACTGAGACACGGTCCAGACTCCTACGGGAGGCAGCAGTGGGGAATATTGCACAATGG
GGGGAACCCTGATGCAGCCATGCCGCGTGAATGAAGAAGGCCTTCGGGTTGTAAAGTTCTTTCGGTAGCG
AGGAAGGTATCAAATTTAATAGATTTGGTAATTGACGTTAACTACAGAAGAAGCACCGGCTAACTCCGTG
CCAGCAGCCGCGGTAATACGGAGGGTGCGAGCGTTAATCGGAATAACTGGGCGTAAAGGGCACGCAGGCG
GTTGATTAAGTGAGATGTGAAAGCCCCGGGCTTAACCTGGGAATTGCATTTCATACTGGTCAACTAGAGT
ACTTTAGGGAGGGGTAGAATTCCACGTGTAGCGGTGAAATGCGTAGAGATGTGGAGGAATACCGAAGGCG
AAGGCAGCCCCTTGGGAATGTACTGACGCTCATGTGCGAAAGCGTGGGGAGCAAACAGGATTAGATACCC
TGGTAGTCCACGCTGTAAACGCTGTCGATTTGGGGATTGGACTTTAAGTTTGGTGCCCGAAGCTAACGTG
ATAAATCGACCGCCTGGGGAGTACGGCCGCAAGGTTAAAACTCAAATGAATTGACGGGGGCCCGCACAAG
CGGTGGAGCATGTGGTTTAATTCGATGCAACGCGAAGAACCTTACCTACTCTTGACATCCAAAGAAGAAC
TCAGAGATGAGTTTGTGCCTTCGGGAACTTTGAGACAGGTGCTGCATGGCTGTCGTCAGCTCGTGTTGTG
AAATGTTGGGTTAAGTCCCGCAACGAGCGCAACCCTTATCCTTTGTTGCCAGCGGTTCGGCCGGGAACTC
AAAGGAGACTGCCAGTGATAAACTGGAGGAAGGTGGGGATGACGTCAAGTCATCATGGCCCTTACGAGTA
GGGCTACACACGTGCTACAATGGCGTATACAGAGGGAAGCAATATGGCGACATGGAGCGAATCTCACAAA
GTACGTCTAAGTCCGGATTGGAGTCTGCAACTCGACTCCATGAAGTCGGAATCGCTAGTAATCGCAAATC
AGAATGTTGCGGTGAATACGTTCCCGGGCCTT
>302129321|dbj|AB547683.1|P.disiens|Prevotella disiens gene for 16S ribosomal RNA, partial sequence, strain: JCM 6333 (88%)
TGGCTCAGGATGAACGCTAGCTATAGGCTTAACACATGCAAGTCGAGGGGAAACGGCAGAGAGTGCTTGC
ACACTTTGGACGTCGACCGGCGCACGGGTGAGTAACGCGTATCCAACCTGCCCTTTACTTGGGGATAACC
CGTTGAAAGACGGACTAATACCCAATGATATTCATTGATGACATCTGATTTGAATTAAAGATTTATCGGT
ATAGGATGGGGATGCGTCTGATTAGCTTGTTGGTGAGGTAACGGCTCACCAAGGCTACGATCAGTAGGGG
TTCTGAGAGGAAGATCCCCCACATTGGAACTGAGACACGGTCCAAACTCCTACGGGAGGCAGCAGTGAGG
AATATTGGTCAATGGACGAGAGTCTGAACCAGCCAAGTAGCGTGCAGGATGACGGCCCTATGGGTTGTAA
ACTGCTTTTGTTTGGGAATAATCGACATTACGTGTAATGTTTTGCATGTACCATTCGAATAAGGACCGGC
TAATTCCGTGCCAGCAGCCGCGGTAATACGGAAGGTCCAGGCGTTATCCGGATTTATTGGGTTTAAAGGG
AGTGTAGGCGGTTGGTTAAGCGTGTTGTGAAATGTAGATGCTCAACATCTGACTTGCAGCGCGAACTGGC
TGACTTGAGTACACACAACGTAGGCGGAATTCATGGTGTAGCGGTGAAATGCTTAGATATCATGAAGAAC
TCCGATTGCGAAGGCAGCTTACGGGAGTGTTACTGACGCTTAAGCTCGAAGGTGCGGGTATCGAACAGGA
TTAGATACCCTGGTAGTCCGCACAGTAAACGATGGATGCCCGCTGTTAGCACCTGGTGTTAGCGGCTAAG
CGAAAGCATTAAGCATCCCACCTGGGGAGTACGCCGGCAACGGTGAAACTCAAAGGAATTGACGGGGGCC
CGCACAAGCGGAGGAACATGTGGTTTAATTCGATGATACGCGAGGAACCTTACCCGGGCTTGAATTGTAG
GAGCACGATACAGAGATGTTGAGGTCCTTCGGGATTCCTATGAAGGTGCTGCATGGTTGTCGTCAGCTCG
TGCCGTGAGGTGTCGGCTTAAGTGCCATAACGAGCGCAACCCCTTTCCTTAGTTGCCATCAGGTAATGCT
GGGCACTCTGAGGATACTGCCACCGTAAGGTGTGAGGAAGGTGGGGATGACGTCAAATCAGCACGGCCCT
TACGTCCGGGGCTACACACGTGTTACAATGGCCGGTACAGAATGTTGGTTGCATGTAAATGTAATCTAAT
CTTTAAAGCCGGTCCCAGTTCGGACTGAGGTCTGCAACCCGACCTCACGAAGCTGGATTCGCTAGTAATC
GCGCATCAGCCATGGCGCGGTGAATACGTTCCCGGGCCTTGTACACACCGCCCGTCAAGCCATGAAAGCC
GGGGGTGCCTGAAGTTCGTAACCGCAAGGAGCGACCTAGGGCAAAACTGGTAATTGGGGCTAAGTCGTAA
CAAGGTAACC
>S002290667|PG21|FP236530|M.hominis|Mycoplasma hominis
TTTTATAAGAGTTTGATCCTGGCTCAGGATGAACGCTGGCTGTGTGCCTAATACATGCATGTCGAGCGAGGTT
AGCAATA
ACCTAGCGGCGAATGGGTGAGTAACACGTGCTTAATCTACCTTTTAGATTGGAATACCCATTGGAAACAATGG
CTAATGC
CGGATACGCATGGAACCGCATGGTTCCGTTGTGAAAGGCGCTGTAAGGCGCCACTAAAAGATGAGGGTGCGGA
ACATTAG

TTAGTTGGTGAGGTAATGGCCCACCAAGACTATGATGTTTAGCCGGGTCGAGAGACTGAACGGCCACATTGGG
ACTGAGA
TACGGCCCAAACTCCTACGGGAGGCAGCAGTAGGGAATATTCCACAATGAGCGAAAGCTTGATGGAGCGACAC
AGCGTGC
ACGATGAAGGTCTTCGGATTGTAAAGTGCTGTTATAAGGGAAGAACATTTGCAATAGGAAATGATTGCAGACT
GACGGTA
CCTTGTCAGAAAGCGATGGCTAACTATGTGCCAGCAGCCGCGGTAATACATAGGTCGCAAGCGTTATCCGGAA
TTATTGG
GCGTAAAGCGTTCGTAGGCTGTTTGTTAAGTCTGGAGTTAAATCCCGGGGCTCAACCCCGGCTCGCTTTGGAT
ACTAGCA
AACTAGAGTTAGATAGAGGTAAGCGGAATTCCATGTGAAGCGGTGAAATGCGTAGATATATGGAAGAACACCA
AAGGCGA
AGGCAGCTTACTGGGTCTATACTGACGCTGAGGGACGAAAGCGTGGGGAGCAAACAGGATTAGATACCCTGGT
AGTCCAC
GCCGTAAACGATGATCATTAGTCGGTGGAGAATCACTGACGCAGCTAACGCATTAAATGATCCGCCTGAGTAG
TATGCTC
GCAAGAGTGAAACTTAAAGGAATTGACGGGGACCCGCACAAGCGGTGGAGCATGTGGTTTAATTTGAAGATAC
ACGGAAA
ACCTTACCCACTCTTGACATCCTTCGCAAAGCTATAGAGATATAGTGGAGGTTATCGGAGTGACAGATGGTGC
ATGGTTG
TCGTCAGCTCGTGTCGTGAGATGTTTGGTCAAGTCCTGCAACGAGCGCAACCCCTATCTTTAGTTACTAACAT
TAAGTTG
AGGACTCTAGAGATACTGCCTGGGTAACTGGGAGGAAGGTGGGGATGACGTCAAATCATCATGCCTCTTACGA
GTGGGGC
CACACACGTGCTACAATGGTCGGTACAAAGAGAAGCAATATGGCGACATGGAGCAAATCTCAAAAAGCCGATC
TCAGTTC
GGATTGGAGTCTGCAATTCGACTCCATGAAGTCGGAATCGCTAGTAATCGCAGATCAGCTATGCTGCGGTGAA
TACGTTC
TCGGGTCTTGTACACACCGCCCGTCACACCATGGGAGCTGGTAATACCCAAAGTCGGTTTGCTAACCTCGGAG
GCGACCG
CCTAAGGTAGGACTGGTGACTGGGGTGAAGTCGTAACAAGGTATCCCTACGAGAACGTGGGGATGGATCACCT
CCTTT
>7008166|gb|AF221119.1|M.spermatophilum|Mycoplasma spermatophilum isolate AH159(T) 16S ribosomal RNA gene, partial sequence
CTGGCTGTGTGCCTAATACATGCATGTCGAGCGAAGTAGCAATACTTAGCGGCGAATGGGTGAGTAACAC
GTGCTCAACGTGCCCTCAAGATTGGGATAGCAACTGGAAACAGTTGATAATACCAAATACTTATATTTTT
CGCATGAAGAATATATAAAAGGAGCGTTTGCTCCGCTCGAGGATCGGGGTGCGTAACATTAGCTAGTTGG
TGAGGTAACGGCCCACCAAGGCGATGATGTTTAGCGGGGTTGAGAGACTGAACCGCCACACTGGGACTGA
GATACGGCCCAGACTCCTACGGGAGGCAGCAGTAGGGAATATTCCACAATGAACGAAAGTTTGATGGAGC
GACACAGCGTGCAGGATGAAGGTCCTATGGATTGTAAACTGCTGTGGTAAGGGAAGAAAAAGTAGCTTAG
GAAATGAAGTTACATTGACGGTACCTTATTAGAAAGCAACGGCTAACTATGTGCCAGCAGCCGCGGTAAT
ACATAGGTTGCAAGCGTTATCCGGAATTATTGGGCGTAAAGCATCTGTAGGTTGTTTGTTAAGTCTGGCG
TCAAATTTTGGGGCTCAACCCCAAATCGCGTTGGATACTGGCAGACTAGAGTTATGTAGAGGTTAGCAGA
ATTCCTTGTGAAGCGGTGAAATGCGTAGATATAAGGAAGAATATCAATATGGCGAAGGCAGCTAACTGGA
CATACACTGACACTGAGAGATGAAAGCGTGGGGAGCAAACAGGATTAGATACCCTGGTAGTCCACGCCCT
AAACGATGATCATTAGCTGATGGAGAATTCATCGGCGCAGCTAACGCATTAAATGATCCGCCTGAGTAGT
ACGTTCGCAAGAATAAAACTTAAAGGAATTGACGGGGATCCGCACAAGCGGTGGAGCATGTGGTTTAATT
TGAAGATACGCGTAGAACCTTACCCACTCTTGACATCTTCTGCAATGCTATGGAGACATAGCGGAGGTTA
ACAGAATGACAGATGGTGCATGGTTGTCGTCAGCTCGTGTCGTGAGATGTTTGGTTAAGTCCTGCAACGA
GCGCAACCCTTATCCTTAGTTACCATCATTTAGTTGGGGACTCTAGGGAGACTGCCCGAGTAATCGGGAG
GAAGGTGGGGATGACGTCAAATCATCATGCCTCTTACGAGTGGGGCAACACACGTGCTACAATGGACGGT
ACAAAGAGAAGCGAAGTGGTGACATGGAGCAAACCTCAAAAAACCGTTCTCAGTTCGGACTGTAGTCTGC
AACTCGACTACACGAAGTCGGAATCGCTAGTAATCGTAGATCAGCTACGCTACGGTGAATACGTTCTCGG
GTCTTGTACACACCGCCCGTCAAACCATGGGAGCTGGTAATGCCCGAAGTCGGTTTATAAACAAACTGCC

TAAGGCAGGACTGGTGACTGGGGTTAAGT
>281186793|gb|GU227398.1|U.diversum|Ureaplasma diversum strain ATCC 49783 16S ribosomal RNA gene, partial sequence
ATACATGCAATCGAACGAAGCCTTTTTGGCTTAGTGGTGAACGGGTGAGTAACACGTATCCAATCTACCC
TTAAGATTGGGATAACTAGTCGAAAGATTAGCTAATACCGAATAATGACATTCTTTTGCATGAAAGAATG
TAGAAAGTTGCGTTTGCAACGCTTTTGGATGAGGGTGCGACGTATCAGATAGTTGGTGAGGTAACGGCTC
ACCAAGTCATTGACGCGTAGCTGTACTGAGAGGTAGAACAGCCACAATGGGACTGAGACACGGCCCATAC
TCCTACGGGAGGCAGCAGTAGGGAATTTTTCACAATGGGCGAAAGCCTTATGAAGCAATGCCGCGTGAAC
GATGAAGGTCTATAAGATTGTAAAGTTCTTTTATTTGGGAAGAACCACTAAAATAGGAAATGATTTTAGT
TTGACTGTACCATTTGAATAAGTATCGGCTAACTATGTGCCAGCAGCCGCGGTAATACATAGGATGCAAG
CGTTATCCGGATTTACTGGGCGTAAAACGAGCGCAGGCGGATTTGTAAGTTTGGTATGAAATCTAGATGC
TTAACGTCTAGCTGTATCAAAAACTACGAATCTAGAGTGTAGCAGAGAGTTGGGGAACTCCATGTGGAGC
GGTAAAATGCGTAGATATATGGAAGAACACCGGTGGCGAAGGCGCCAACTTGGACTATTACTGACGCTTA
GGCTCGAAAGTGTGGGGAGCAAATAGGATTAGATACCCTAGTAGTCCACACCGTAAACGATCATCATTAA
ATGTCGGCTCGATTATGAGTCGGTGTTGTAGCTAACGCATTAAATGATGTGCCTGGGTAGTACATTCGCA
AGAATGAAACTCAAACGGAATTGACGGGGACCCGCACAAGTGGTGGAGCATGTTGCTTAATTTGACAATA
CACGTAGAACCTTACCTAGGTTTGACATCTATTGCAATGCTATAGAAATATAGCGGAGGTTACAATATGA
CAGTGGTGCATGTTGTCGTCAGCTCGTGTCGTGAGATGTTGGGTTAAGTCCCGCAACGAGCGCAACCCCT
TTCGCTAGTTAATTTATCTATCGATACTGCTACCGCAAGGTAGAGGAAGGTGGGGATGACGTCAAATCAT
CATGCCCCTTATATCTAGGGCTGCAAACGTGCTACAATGGCTAATACAAACAGCTGCAATTCCGCAAGGT
TGAGCGAATCTGATAAAGTTAGTCTCAGTTCGGATAGAGGGCTGCAATTCGCCCTCTTGAAGTTGGAATC
ACTAGTAATCGCGAATCAGACATGTCGCGGTGAATACGTTCTCGGGTCTTGTACACACCGCCCGTCAAAC
TATGGGAGCCGGTAATATCTGAAACCGTACTGTAACCTTTTTG
>281186792|gb|GU227397.1|U.diversum|Ureaplasma diversum strain ATCC 49782 16S ribosomal RNA gene, partial sequence
ATACATGCAATCGAACGAAGCCTTTTTGGCTTAGTGGTGAACGGGTGAGTAACACGTATCCAATCTACCC
TTAAGTTTGGGATAACTAGTCGAAAGATTAGCTAATACCGGATAATAACATTTACTTGCATGAGTGAATG
TAGAAAGTTGCGTTTGCAACGCTTTTGGATGAGGGTGCGACGTATCAGATAGTTGGTGAGGTAACGGCTC
ACCAAGTCATTGACGCGTAGCTGTACTGAGAGGTAGAACAGCCACAATGGGACTGAGACACGGCCCATAC
TCCTACGGGAGGCAGCAGTAGGGAATTTTTCACAATGGGCGAAAGCCTTATGAAGCAATGCCGCGTGAAC
GATGAAGGTCTATAAGATTGTAAAGTTCTTTTATTTGGGAAGAACCACTAAAATAGGAAATGATTTTAGT
TTGACTGTACCATTTGAATAAGTATCGGCTAACTATGTGCCAGCAGCCGCGGTAATACATAGGATGCAAG
CGTTATCCGGATTTACTGGGCGTAAAACGAGCGCAGGCGGATTTGTAAGTTTGGTATGAAATCTAGATGC
TTAACGTCTAGCTGTATCAAAAACTACGAATCTAGAGTGTAGCAGAGAGTTGGGGAACTCCATGTGGAGC
GGTAAAATGCGTAGATATATGGAAGAACACCGGTGGCGAAGGCGCCAACTTGGACTATTACTGACGCTTA
GGCTCGAAAGTGTGGGGAGCAAATAGGATTAGATACCCTAGTAGTCCACACCGTAAACGATCATCATTAA
ATGTCGGCTCGATTATGAGTCGGTGTTGTAGCTAACGCATTAAATGATGTGCCTGGGTAGTACATTCGCA
AGAATGAAACTCAAACGGAATTGACGGGGACCCGCACAAGTGGTGGAGCATGTTGCTTAATTTGACAATA
CACGTAGAACCTTACCTAGGTTTGACATCTATTGCAATGCTATAGAAATATAGCGGAGGTTAACAATATG
ACAGGTGGTGCATGGTTGTCGTCAGCTCGTGTCGTGAGATGTTGGGTTAAGTCCCGCAACGAGCGCAACC
CCTTTCGCTAGTTAATTTATCTATCGATACTGCTACCGCAAGGTAGAGGAAGGTGGGGATGACGTCAAAT
CATCATGCCCCTTATATCTAGGGCTGCAAACGTGCTACAATGGCTAATACAAACAGCTGCAATTCCGCAA
GGTTGAGCGAATCTGATAAAGTTAGTCTCAGTTCGGATAGAGGGCTGCAATTCGCCCTCTTGAAGTTGGA
ATCACTAGTAATCGCGAATCAGACATGTCGCGGTGAATACGTTCTCGGGTCTTGTACACACCGCCCGTCA
AACTATGGGAGCCGGTAATATCTGAAACCGTACTGTAACCTTTTTG
>281186787|gb|GU227392.1|Ureaplasma|Ureaplasma sp. USP45 16S ribosomal RNA gene, partial sequence
ATACATGCAATCGAACGAAGCCTTTTTGGCTTAGTGGTGAACGGGTGAGTAACACGTATCCAATCTACCC
TTAAGTTTGGGATAACTAGTCGAAAGATTAGCTAATACCGGATAATAACATTTGCTTGCATGAGTGAATG
TAGAAAGTTGCGTTTGCAACGCTTTTGGATGAGGGTGCGACGTATCAGATAGTTGGTGAGGTAACGGCTC
ACCAAGTCATTGACGCGTAGCTGTACTGAGAGGTAGAACAGCCACAATGGGACTGAGACACGGCCCATAC
TCCTACGGGAGGCAGCAGTAGGGAATTTTTCACAATGGGCGAAAGCCTTATGAAGCAATGCCGCGTGAAC
GATGAAGGTCTATAAGATTGTAAAGTTCTTTTATTTGGGAAGAACCACTAAAATAGGAAATGATTTTAGT
TTGACTGTACCATTTGAATAAGTATCGGCTAACTATGTGCCAGCAGCCGCGGTAATACATAGGATGCAAG

CGTTATCCGGATTTACTGGGCGTAAAACGAGCGCAGGCGGATTCGTAAGTTTGGTATGAAATCTAGATGC
TTAACGTCTAGCTGTATCAAAAACTACGAATCTAGAGTGTAGCAGAGAGTTGGGGAACTCCATGTGGAGC
GGTAAAATGCGTAGATATATGGAAGAACACCGGTGGCGAAGGCGCCAACTTGGACTATTACTGACGCTTA
GGCTCGAAAGTGTGGGGAGCAAATAGGATTAGATACCCTAGTAGTCCACACCGTAAACGATCATCATTAA
ATGTCGGCTCGATTATGAGTCGGTGTTGTAGCTAACGCATTAAATGATGTGCCTGGGTAGTACATTCGCA
AGAATGAAACTCAAACGGAATTGACGGGGACCCGCACAAGTGGTGGAGCATGTTGCTTAATTTGACAATA
CACGTAGAACCTTACCTAGGTTTGACATCTATTGCAATGCTATAGAAATATAGCGGAGGTTAACAATATG
ACAGGTGGTGCATGGTTGTCGTCAGCTCGTGTCGTGAGATGTTGGGTTAAGTCCCGCAACGAGCGCAACC
CCTTTCGCTAGTTAATTTATCTATCGATACTGCTACCGCAAGGTAGAGGAAGGTGGGGATGACGTCAAAT
CATCATGCCCCTTATATCTAGGGCTGCAAACGTGCTACAATGGCTAATACAAACAGCTGCAAAACCGTAA
GGTGGAGCGAATCTGATAAAGTTAGTCTCAGTTCGGATAGAGGGCTGCAATTCGCCCTCTTGAAGTTGGA
ATCACTAGTAATCGCGAATCAGACATGTCGCGGTGAATACGTTCTCGGGTCTTGTACACACCGCCCGTCA
AACTATGGGAGCCGGTAATATCTGAAACCGTACTGTTAACCTTTTG
>164460338|gb|EU188962.1|Pv.123b-95|
CCCTATGGGTTGTAAACTGCTTTTATGTGGGGATAAAGTGCGTGACGTGTCATGCATTGCAGGTACCACA
TGAATAAGGACCGGCTAATTCCGTGCCAGCAGCCGCGGTAATACGGAAGGTCCGGGCGTTATCCGGATTT
ATTGGGTTTAAAGGGAGCGTAGGCTGTCTATTAAGCGTGTTGTGAAATTTACCGGCTCAACCGGTGGCTT
GCAGCGCGAACTGGTCGACTTGAGTATGCAGGAAGTAGGCGGAATTCATGGTGTAGCGGTGAAATGCTTA
GATATCATGACGAACTCCGATTGCGCAGGCAGCTTACTGTAGCATAACTGACGCTGATGCTCGAAAGTGC
GGGTATCAAACAGGATTAGATACCCTGGTAGTCCGCACGGTAAACGATGGATGCTCGCTATTCGTCCTAT
TTGGATGAGTGGCCAAGTGAAAACATTAAGCATCCCACCTGGGGAGTACGCCGGCAACGGTGAAACTCAA
AGGAATTGACGGGGGCCCGCACAAGCGGAGGAACATGTGGTTTAATTCGATGATACGCGAGGAACCTTAC
CCGGGCTTGAACTGCCGGCGAACGATCCAGAGATGGTGAGGCCCCTCGGGGCGCCGGTGGAGGTGCTGCA
TGGTTGTCGTCAGCTCGTGCCGTGAGGTGTCGGCTTAAGTGCCATAACGAGCGCAACCCCTCTCCGTAGT
TGCCATCAGGTAGTGCTGGGCACTCTGCGGACACTGCCACCGCAAGGTGCGAGGAAGGTGGGGATGACGT
CAAATCAGCACGGCCCTTACGTCCGGGGCTACACACGTGTTACAATGGGGGGCACAGCGAGTCGGCCGCG
CGCAAGCTCGGTCCAATCAAGAAATCCCCCCTCAGTTCGGACTGGGGTCTGCAACCCGAC
>164460319|gb|EU188943.1|Pv.123f3-83|
GCAGGATGACGGCCCTATGGGTTGTAAACTGCTTTTATGTGGGGATAAAGTGCGTGACGTGTCATGCATT
GCAGGTACCACATGAATAAGGACCGGCTAATTCCGTGCCAGCAGCCGCGGTAATACGGAAGGTCCGGGCG
TTATCCGGATTTATTGGGTTTAAAGGGAGCGTAGGCTGTCTATTAAGCGTGTTGTGAAATTTACCGGCTC
AACCGGTGGCTTGCAGCGCGAACTGGTCGACTTGAGTATGCAGGAAGTAGGCGGAATTCATGGTGTAGCG
GTGAAATGCTTAGATATCATGACGAACTCCGATTGCGCAGGCAGCTTACTGTAGCATAACTGACGCTGAT
GCTCGAAAGTGCGGGTATCAAACAGGATTAGATACCCTGGTAGTCCGCACAGTAAACGATGGATGCCCGC
TGTTAGCACCTAGTGTTAGCGGCTAAGCGAAAGCATTAAGCATCCCACCTGGGGAGTACGCCGGCAACGG
TGAAACTCAAAGGAATTGACGGGGGCCCGCACAAGCGGAGGAACATGTGGTTTAATTCGATGATACGCGA
GGAACCTTACCCGGGCTTGAATTGCAGATGTTTATATCAGAGATGATATATTCCCTTCGGGGCATTTGTG
AAGGTGCTGCATGGTTGTCGTCAGCTCGTGCCGTGAGGTGTCGGCTTAAGTGCCATAACGAGCGCAACCC
CTTTTTTTAGTTGCCATCAGGTAGTGCTGGGCACTCTAGAGATACTGCCACCGTAAGGTGTGAGGAAGGT
GGGGATGACGTCAAATCAGCACGGCCCTTACGTCCGGGGCTACACACGTGTTACAATGGGTGGTACAGAG
AGTTGGTTGTACGCAAGTGCAATCTAATCCTAAAAACCATTCTCAGTTCGGACTGGGGTC
>164460332|gb|EU188956.1|Pv.6BV-87|
TACGGAAGGTCCAGGCGTTATCCGGATTTATTGGGTTTAAAGGGAGCGTAGGCTGTTTGTTAAGCGTGTT
GTGAAATGTAAGAGCTCAACTTTTAGATTGCAGCGCGAACTGGCAGACTTGAGTGCGCACAACGTAGGCG
GAATTCATGGTGTAGCGGTGAAATGCTTAGATATCATGACGAACTCCGATTGCGAAGGCAGCTTACGGGA
GCGCAACTGACGCTAAAGCTCGAAGGTGCGGGTATCGAACAGGATTAGATACCCTGGTAGTCCGCACAGT
AAACGATGGATGCCCGCTGTTAGCACCTAGTGTTAGCGGCTAAGCGAAAGCATTAAGCATCCCACCTGGG
GAGTACGCCGGCAACGGTGAAACTCAAAGGAATTGACGGGGGCCCGCACAAGCGGAGGAACATGTGGTTT
AATTCGATGATACGCGAGGAACCTTACCCGGGCTTGAACTGCCAGCGAACGATACAGAGATGTTGAGGCC
CTTCGGGGCGCTGGTGGAGGTGCTGCATGGTTGTCGTCAGCTCGTGCCGTGAGGTGTCGGCTTAAGTGCC
ATAACGAGCGCAACCCTTTTCTTTAGTTGCCATCAGGTAATGCTGGGCACTCTATGGATACTGCCACCGT
AAGGTGTGAGGAAGGTGGGGATGACGTCAAATCAGCACGGCCCTTACGTCCGGGGCTACACACGTGTTAC
AATGGGGCATACAGAGTGTTGGCTTAACGCAAGTTTGGTCTAATCTTCAAAGTGTCTCCCAGTTCGGATT
GGGGTCTGCAACCCGACCCCATGAAGCTGGATTCGCTAGTAATCGCGCATCAGCCATGGCGCGGTGAATA

CGTTCCCGGG
>164460316|gb|EU188940.1|Pv.127b-13|
AAATGTACCGGCTCAACCGGTGAATTGCAGCGCGAACTGTTTGGCTTGAGTGCACGGTAAGCAGGCGGAA
TTCATGGTGTAGCGGTGAAATGCTTAGATATCATGAAGAACTCCGATTGCGAAGGCAGCTTGCTGCAGTG
CGACTGACGCTGATGCTCGAAGGTGCGGGTATCAAACAGGATTAGATACCCTGGTAGTCCGCACGGTAAA
CGATGGATGCCCGCTGTCCGCCTTTTGTGGCGGGTGGCCAAGCGAAAGCGTTAAGCATCCCACCTGGGGA
GTACGCCGGCAACGGTGAAACTCAAAGGAATTGACGGGGGCCCGCACAAGCGGAGGAACATGTGGTTTAA
TTCGATGATACGCGAGGAACCTTACCCGGGCTTGAACTGCCGGTGAACGATACAGAGATGTTGAGGCCCT
TCGGGGCGCCGGTGGAGGTGCTGCATGGTTGTCGTCAGCTCGTGCCGTGAGGTGTCGGCTTAAGTGCCAT
AACGAGCGCAACCCCTTTTTTCAGTTGCCATCAGGTAATGCTGGGCACTCTGGAGATACTGCCACCGCAA
GGTGTGAGGAAGGTGGGGATGACGTCAAATCAGCACGGCCCTTACGTCCGGGGCTACACACGTGTTACAA
TGGGGCATACAGAGTGTTGGCTTAACGCAAGTTTGGTCTAATCTTCAAAGTGTCTCCCAGTTCGGACTGG
GGTCTGCAACCCGACCCCACGAAGCTGGATTCGCTAGTAATCGCGCATCAGCCATGGCGCGGTGAATACG
TTCCCGGG
>164460321|gb|EU188945.1|Pv.136b-68|
TCATGAAGAACTCCGATTGCGAAGGCAGCTTGCTGCAGTGCGACTGACGCTGATGCTCGAAGGTGCGGGT
ATCAAACAGGATTAGATACCCTGGTAGTCCGCACGGTAAACGATGGATGCCCGCTGTCCGCCTTTTGTGG
CGGGTGGCCAAGCGAAAGCGTTAAGCATCCCACCTGGGGAGTACGCCGGCAACGGTGAAACTCAAAGGAA
TTGACGGGGGCCCGCACAAGCGGAGGAACATGTGGTTTAATTCGATGATACGCGAGGAACCTTACCCGGG
CTTGAACTGCCAGTGAACGATACAGAGATGTTGAGGCCCTTCGGGGCGCTGGTGGAGGTGCTGCATGGTT
GTCGTCAGCTCGTGCCGTGAGGTGTCGGCTTAAGTGCCATAACGAGCGCAACCCCTTTTTTCAGTTGCCA
TCAGGTAATGCTGGGCACTCTGGAGATACTGCCACCGCAAGGTGTGAGGAAGGTGGGGATGACGTCAAAT
CAGCACGGCCCTTACGTCCGGGGCTACACACGTGTTACAATGGGGCATACAGAGTGTTGGCTTAACGCAA
GTTTGGTCTAATCTTCAAAGTGTCTCCCAGTTCGGACTGGGGTCTGCAACCCGACCCCACGAAGCTGGAT
TCGCTAGTAATCGCGCATCAGCCATGGCGCGGTGAATACGTTCCCGGG
>164460379|gb|EU189003.1|Pv.123b-4|
TGAGGAATATTGGTCAATGGGCGAGAGCCTGAACCAGCCAAGTAGCGTGCAGGATGACGGCCCTATGGGT
TGTAAACTGCTTTTATGTGGGGATAAAGTGCGCGACGTGTCGTGCATTGCAGGTACCACATGAATAAGGA
CCGGCTAATTCCGTGCCAGCAGCCGCGGTAATACGGAAGGTCCAGGCGTTATCCGGATTTATTGGGTTTA
AAGGGAGCGTAGGCTGTCTATTAAGCGTGTTGTGAAATTTACCGGCTCAACCGGTGGCTTGCAGCGCGAA
CTGGTCGACTTGAGTATGCAGGAAGTAGGCGGAATTCATGGTGTAGCGGTGAAATGCTTAGATATCATGA
GGAACTCCGATTGCGAAGGCAGCTTGCTGCAGTGCGACTGACGCTTAGGCTCGAAGGTGCGGGTATCAAA
CAGGATTAGATACCCTGGTAGTCCGCACGGTAAACGATGGATGCCCGCTGTCCGCCCATTCGTGGCGGGT
GGCCAAGCGAAAGCGTTAAGCATCCCACCTGGGGAGTACGCCGGCAACGGTGAAACTCAAAGGAATTGAC
GGGGGCCCGCACAAGCGGAGGAACATGTGGTTTAATTCGATGATACGCGAGGAACCTTACCCGGGCTTGA
ACTGCCGGCGAACGATACAGAGATGTTGAGGCCCTTCGGGGCGCCGGTGGAGGTGCTGCATGGTTGTCGT
CAGCTCGTGCCGTGAGGTGTCGGCTTAAGTGCCATAACGAGCGCAACCCCTCTCTTCAGTTGCCATCAGG
TGATGCTGGGCACTCTGGAGACACTGCCACCGCAAGGTGTGAGGAAGGTGGGGATGACGTCAAATCAGCA
CGGCCCTTACGTCCGGGGCTACACACGTGTTACAATGGGGCATACAGAACGTCGGTTCAA
>164460361|gb|EU188985.1|Pv.123b-9|
GTGCCAGCAGCCGCGGTAATACGGAAGGTCCGGGCGTTATCCGGATTTATTGGGTTTAAAGGGAGCGTAG
GCTGTCTATTAAGCGTGTTGTGAAATTTACCGGCTCAACCGGTGGCTTGCAGCGCGAACTGGTCGACTTG
AGTATGCAGGAAGTAGGCGGAATTCATGGTGTAGCGGTGAAATGCTTAGATATCATGACGAACTCCGATT
GCGCAGGCAGCTTACTGTAGCATAACTGACGCTGATGCTCGAAAGTGCGGGTATCAAACAGGATTAGATA
CCCTGGTAGTCCGCACGGTAAACGATGGATGCTCGCTATTCGTCCTATTTGGATGAGTGGCCAAGTGAAA
ACATTAAGCATCCCACCTGGGGAGTACGCCGGCAACGGTGAAACTCAAAGGAATTGACGGGGGCCCGCAC
AAGCGGAGGAACATGTGGTTTAATTCGATGATACGCGAGGAACCTTACCCGGGCTTGAACTGCCAGCGAA
CGATACAGAGATGTTGAGGCCCTTCGGGGCGCTGGTGGAGGTGCTGCATGGTTGTCGTCAGCTCGTGCCG
TGAGGTGTCGGCTTAAGTGCCATAACGAGCGCAACCCTTTTCTTTAGTTGCCATCAGGTAATGCTGGGCA
CTCTATGGATACTGCCACCGTAAGGTGTGAGGAAGGTGGGGATGACGTCAAATCAGCACGGCCCTTACGT
CCGGGGCTACACACGTGTTACAATGGGGCATACAGAGTGTTGGCTTAACGCAAGTTTGGTCTAATCTTCA
AAGTGTCTCCCAGTTCGGATTGGGGTCTGCAACCCGACCCCATGAAGCTGGATTCGCTAGTAATCGCGCA
TCAGCCATGGCGCGGTGAATACGTTCCCGGG

>164460393|gb|EU189017.1|Pv.113s1-20|
TGAGGAATATTGGTCAATGGGCGAGAGCCTGAACCAGCCAAGTAGCGTGCAGGACGACGGCCCTATGGGT
TGTAAACTGCTTTTATGTGGGGATAAAGTGCGTGACGTGTCATGCATTGCAGGTACCACATGAATAAGGA
CCGGCTAATTCCGTGCCAGCAGCCGCGGTAATACGGAAGGTCCGGGCGTTATCCGGATTTATTGGGTTTA
AAGGGAGCGTAGGCTGTCTATTAAGCGTGTTGTGAAATTTACCGGCTCAACCGGTGGCTTGCAGCGCGAA
CTGGTCGACTTGAGTATGCAGGAAGTAGGCGGAATTCATGGTGTAGCGGTGAAATGCTTAGATATCATGA
CGAACTCCGATTGCGCAGGCAGCTTACTGTAGCATAACTGACGCTGATGCTCGAAAGTGCGGGTATCAAA
CAGGATTAGATACCCTGGTAGTCCGCACGGTAAACGATGGATGCTCGCTATTCGTCCGTTTAGGATGAGT
GGCCAAGTGAAAACATTAAGCATCCCACCTGGGGAGTACGCCGGCAACGGTGAAACTCAAAGGAATTGAC
GGGGGCCCGCACAAGCGGAGGAACATGTGGTTTAATTCGATGATACGCGAGGAACCTTACCCGGGCTTGA
ACTGCCAGCGAACGATACAGAGATGTTGAGGCCCTTCGGGGCGCTGGTGGAGGTGCTGCATGGTTGTCGT
CAGCTCGTGCCGTGAGGTGTCGGCTTAAGTGCCATAACGAGCGCAACCCTTTTCTTTAGTTGCCATCAGG
TGATGCTGGGCACTCTATGGATACTGCCACCGTAAGGTGTGAGGAAGGTGGGGATGACGTCAAATCAGCA
CGGCCCTTACGTCCGGGGCTACACACGTGTTACAATGGGGCATACAGAGTGTTGGCTTAA
>99907986|gb|DQ518919.1|P.timonensis|
GATGAACGCTAGCTACAGGCTTAACACATGCAAGTCGCAGGGTAACATGAGGAAAGCTTGCTTTCCTTGA
TGACGACTGGCGCACGGGTGAGTAACGCGTATCCAACCTTCCCATAACTACGGGATAACCCGTTGAAAGA
CGGCCTAATACCGTATGAYATCGTTTGCTGACATCAAATAACGATTAAAGGTTTAGCGGTTATGGATGGG
GATGCGTCTGATTAGCTTGTTGGCGGGGTAACGGCCCACCAAGGCTACGATCAGTAGGGGTTCTGAGAGG
AAGGTCCCCCACATTGGAACTGAGACACGGTCCAAACTCCTACGGGAGGCAGCAGTGAGGAATATTGGTC
AATGGGCGAGAGCCTGAACCAGCCAAGTAGCGTGCAGGATGACGGCCCTATGGGTTGTAAACTGCTTTTA
TGTGGGGATAAAGTGCGTGACGTGTCATGCATTGCAGGTACCACATGAATAAGGACCGGCTAATTCCGTG
CCAGCAGCCGCGGTAATACGGAAGGTCCGGGCGTTATCCGGATTTATTGGGTTTAAAGGGAGCGTAGGCT
GTCTATTAAGCGTGTTGTGAAATTTACCGGCTCAACCGGTGGCTTGCAGCGCGAACTGGTCGACTTGAGT
ATGCAGGAAGTAGGCGGAATTCATGGTGTAGCGGTGAAATGCTTAGATATCATGACGAACTCCGATTGCG
CAGGCAGCTTACTGTAGCATAACTGACGCTGATGCTCGAAAGTGCGGGTATCAAACAGGATTAGATACCC
TGGTAGTCCGCACGGTAAACGATGGATGCTCGCTATTCGTCCTATTTGGATGAGTGGCCAAGTGAAAACA
TTAAGCATCCCACCTGGGGAGTACGCCGGCAACGGTGAAACTCAAAGGAATTGACGGGGGCCCGCACAAG
CGGAGGAACATGTGGTTTAATTCGATGATACGCGAGGAACCTTACCCGGGCTTGAACTGCCAGCGAACGA
TACAGAGATGTTGAGGCCCTTCGGGGCGCTGGTGGAGGTGCTGCATGGTTGTCGTCAGCTCGTGCCGTGA
GGTGTCGGCTTAAGTGCCATAACGAGCGCAACCCTTTTCTTTAGTTGCCATCAGGTGATGCTGGGCACTC
TATGGATACTGCCACCGTAAGGTGTGAGGAAGGTGGGGATGACGTCAAATCAGCACGGCCCTTACGTCCG
GGGCTACACACGTGTTACAATGGGGCATACAGAGTGTTGGCTTAACGCAAGTTTGGTCTAATCTTCAAAG
TGTCTCCCAGTTCGGATTGGGGTCTGCAACCCGACCCCATGAAGCTGGATTCGCTAGTAATCGCGCATCA
GCCATGGCGCGGTGAATACGTTCCCGGGCCTTGTACACACCGCCCGTCAAGCCATGAAAGCTGGGGGTGC
CTGAAGTCCGTAACCGTTAAGGAGCGGCCTAGGGCAAAACTGGTGATTGGGGCT
>294430|gb|L16476.1|P.buccalis|
TACAATGTAGAGTTTGATCCTGGCTCAGGATNAACGCTAGCTACAGGCTTAACACATGCAAGTCGCAGGG
TAACGTGAGGGAAGCTTGCTTCCCTTGACGACGACTGGCGCACGGGTGAGTAACGCGTATCCAACCTTCC
CATGACCACGGGATAACCCGTTGAAAGACGGACTAATACCGTATGACGTCGTTTGCTGACATCAAATAAC
GATTAAAGGTTTAGCGGTGATGGATGGGGATGCGTCTGATTAGCTTGTTGGCGGGGTAACGGCCCACCAA
GGCGACGATCAGTAGGGGTTCTGAGAGGAAGGTCCCCCACATTGGAACTGAGACACGGTCCNAACTCCTA
CGGGAGGCAGCAGTGAGGAATATTGGTCAATGGGCGAGAGCCTGAACCAGCCAAGTAGCGTGCAGGATGA
CGGCCCTATGGGTTGTAAACTGCTTTTATGCGGGGATAAAGTGYGCGACGTGTCGTGCATTGCAGGTACC
GCATGAATAAGGACCGGCTNATTCCGTGCCAGCAGCCGCGGTAATACGGAAGGTCCNGGCGTTATCCGGA
TTTATTGGGTTTNNAGGGAGCGTAGGCCGCCAGGTAAGCGTGTTGTGAAATGTACCGGCTCAACCGGTNA
ATTGCAGCGCGAACTGTCTGGCTTGAGTGCACGGTAAGCAGGCGGAATTCATGGTGTAGCGGTGAAATGC
TTAGATATCATGAAGAACTCCGATTGCGAAGGCAGCTTGCTGCAGTGCGACTGACGCTGATGCTCGAAGG
TGCGGGTATCAAACAGGATTAGATACCCTGGTAGTCCGCACGGTAAACGATGGATGCCCCTGTTCCGCCT
TTTTGTGGCGGNTGGCCAAGCGAAAGCGTTAAGCATCCCACCTGGGGAGTACGCCGGCAACGGTGAAACT
CAAAGGAATTGACGGGGGCCCGCACAAGCGGAGGAACATGTGGTTTAATTCGATGNTACGCGAGGAACCT
TACCCGGGCTTGAACTGCCAGTGAACGATACAGAGATGTTGAGGCCTTCGGGGCGCTGGTGGAGGTGCTG
CATGGTTGTCGTCAGCTCGTGCCGTGAGGTGTCGGCTTAAGTGCCATAACGAGCGCAACCCCNNTTTTCA
GTTGCCATCAGGTAATGCTGGGCACTCTGGAGATACTGCCACCGCAAGGTGTGAGGAAGGTGGGGATGAC

GTCAAATCAGCACGGCCCTTACGTCCGGGGCTACACACGTGTTACAATGGGGCATACAGAGTGTTGGCTT
AACGCAAGTTTGGTCTAATCTTCAAAGTGTCTCCCAGTTCGGACTGGGGTCTGCAACCCGACCCCACGAA
GCTGGATTCGCTAGTAATCGCGCATCAGCCATGGCGCGGTGAATACGTTCCCGGGCCTTGTACACACCGC
CCGTCAAGCCATGAAAGCCGGGGGTGCCTGAAGTCCGTAACCGTCAAGGAGCGGCCTAGGGCAAAACTGG
TGAT
>294419|gb|L16465.1|P.corporis|
TACAATGGAGAGTTTGATCCTGGCTCAGGATNAACGCTAGCTACAGGCTTAACACATGCAAGTCGAGGGG
AAACGGCATTAAGTGCTTGCACTTTTTGGACGTCGACCGGCGCACGGGTGAGTAACGCGTATCCAACCTG
CCCCTTACCAGGGAATAACCCGTTGAAAGACGGACTAATGCCCTATGGAGTCCTTTGACGGCATCAGATT
AGGACTAAAGATTCATCGGTATGGGATGGGGATGCGTCTGATTAGCTTGTTGGCGGGGTAACGGCCCACC
AAGGCATCGATCAGTAGGGGTTCTGAGAGGAAGGTCCCCCACATAGGAACTGAGACACGGTCCTAACTCC
TACGGGAGGCAGCAGTGAGGAATATTGGTCAATGGGCGCTAGCCTGAACCAGCCAAGTAGCGTGCAGGAT
GACGGCCCTATGGGTTGTAAACTNCTTTTATGCGGGGATAAAGTCACCCACGTGTGGGTNTTTGCAGGTA
CCGCATGAATAAGGACCGGCTAATTCCGTGCCAGCAGCCGCGGTAATACGGAAGGTCCGGGCGTTATCCG
GATTTATTGGGTTTAAAGGGAGTGTAGGCGGCCTGTTAAGCGTGTTGTGAAATGTAGATGCTCAACATCT
GAACTGCAGCGCGAACTGGCTGGCTTGAGTACACGCAACGTGGGCGGAATTCATGGTGTAGCGGTGAAAT
GCTTAGATATCATGAGGAACTCCTATTGCGAAGGCAGCTCACGGGAGTGTCACTGACGCTTAAGCTCGAA
GGTGCGGGTATCAAACAGGATTAGATACCCTGGTAGTCCGCACGGTAAACGATGGATGCCCGCTGTTGGC
GCTTTGCGTCAGCGGCTAAGCGAAAGCATTAAGCATCCNACCTGGGGAGTACGCCGGCAACGGTGAAACT
CAAAGGAATTGACGGGGGCCCGCACAAGCGGAGGAACATGTGGTTTAATTCGATGATACGCGAGGAACCT
TACCCGGGCTTGAATTGCAGAGGAAAGATCCAGAGATGGTGATNCCCTTCGGGGTCTCTGTGAAGGTGCT
GCATGGTTGTCGTCAGCTCGTGCCGTGAGGTGTCGGCTTAAGTGCCATAACGAGCGCAACCCCNNTTTCC
GGTTGCCATCAGGTGATGCTGGGCACTCCGGGGACACTGCCACCGTAAGGTGTGAGGAAGGTGGGGATGA
CGTCAAATCAGCACGGCCCTTACGTCCGGGGCTACACACGTGTTACAATGGCCGGTACAGAGGGCAGGTG
CAATGCAAATTGCATCAAATCTATAAATCCGGTCTCAGTTCGGACTGGGGTCTGCAACCCGACCCCACGA
AGCTGGATTCGCTAGTAATCGCGCATCAGCCATGGCGCGGTGAATACGTTCCCGGGCCTTGTACACACCG
CCCGTCAAGCCATGAAAGCNGGGGGTGCCTGAAGTTCGTAACCGCAAGGAGCGACCTAGGGCAAAACTGG
TGA
>66878632|gb|AY958926.1|Pv.rRNA153|
CGCCCTTAGAGTTTGATCCTGGCTCAGGATGAACGCTAGCTACAGGCTTAACACATGCAAGTCGAGGGGA
AACGGCATTAAGTGCTTGCACTTTTTGGACGTCGACCGGCGCACGGGTGAGTAACGCGTATCCAACCTGC
CCCTTACCAGGGAATAACCCGTTGAAAGACGGACTAATGCCCTATGCAGTCCTTTGACGGCATCAGATTA
GGACTAAAGATTTATCGGTATGGGATGGGGATGCGTCTGATTAGCTTGTTGGCGGGGTAACGGCCCACCA
AGGCATCGATCAGTAGGGGTTCTGAGAGGAAGGTCCCCCACATAGGAACTGAGACACGGTCCTAACTCCT
ACGGGAGGCAGCAGTGAGGAATATTGGTCAATGGGCGCTAGCCTGAACCAGCCAAGTAGCGTGCAGGACG
ACGGCCCTATGGGTTGTAAACTGCTTTTATGCGGGGATAAAGTCACCCACGTGTGGGTGTTTGCAGGTAC
CGCATGAATAAGGACCGGCTAATTCCGTGCCAGCAGCCGCGGTAATACGGAAGGTCCGGGCGTTATCCGG
ATTTATTGGGTTTAAAGGGAGTGTAGGCGGCCTGTTAAGCGTGTTGTGAAATGTAGATGCTCAACATCTG
AACTGCAGCGCGAACTGGCTGGCTTGAGTACACGCAACGTGGGCGGAATTCATGGTGTAGCGGTGAAATG
CTTAGATATCATGAGGAACTCCTATTGCGAAGGCAGCTCACGGGAGTGTCACTGACGCTTAAGCTCGAAG
GTGCGGGTATCAAACAGGATTAGATACCCTGGTAGTCCGCACGGTAAACGATGGATGCCCGCTGTTGGCG
CTTTGCGTCAGCGGCTAAGCGAAAGCATTAAGCATCCCACCTGGGGAGTACGCCGGCAACGGTGAAACTC
AAAGGAATTGACGGGGGCCCGCACAAGCGGAGGAACATGTGGTTTAATTCGATGATACGCGAGGAACCTT
ACCCGGGCTTGAATTGCAGAGGAAAGATCCAGAGATGGTGATGCCCTTCGGGGCCTCTGTGAAGGTGCTG
CATGGTTGTCGTCAGCTCGTGCCGTGAGGTGTCGGCTTAAGTGCCATAACGAGCGCAACCCCTTTTTCCG
GTTGCCATCAGGTGATGCTGGGCACTCCGGGGACACTGCCACCGTAAGGTGTGAGGAAGGTGGGGATGAC
GTCAAATCAGCACGGCCCTTACGTCCGGGGCTACACACGTGTTACAATGGCCGGTACAGAGGGCAGGTGC
AATGCAAATTGCATCAAATCTATAAATCCGGTCTCAGTTCGGACTGGGGTCTGCAACCCGACCCCACGAA
GCTGGATTCGCTAGTAATCGCGCATCAGCCATGGCGCGGTGAATACGTTCCCGGGCCTTGTACACACCGC
CCGTCAAGCCATGAAAGCCGGGGGTGCCTGAAGTTCGTAACCGCAAGGAGCGACCTAGGGCAAAACTGGT
GATTGGGGCTAAGTCGTAACAAGGTAACCGTAAAGG
>164460360|gb|EU188984.1|Pv.112Q-24|
ACGAACTCCGATTGTGAAGGCAGCTTACGGGAGNGCAACTGACGCTAAAGCTCGAAGGTGCGGGTATCGA
ACAGGATTAGATACCCTGGTAGTCCGCACAGTAAACGATGGATGCCCGCTGTTAGCACCTAGTGTTAGCG

GCTAAGCGAAAGCATTAAGCATCCCACCTGGGGAGTACGCCGGCAACGGTGAAACTCAAAGGAATTGACG
GGGGCCCGCACAAGCGGCGGAACATGTGGTTTAATTCGATGATACGCGAGGAACCTTACCCGGGCTTGAA
TTGCAGATGTTTATATCAGAGATGATATATTCCCTTCGGGGCATTTGTGAAGGTGCTGCATGGTTGTCGT
CAGCTCGTGCCGTGAGGTGTCGGCTTAAGTGCCATAACGAGCGCAACCCCTTTTTTTAGTTGCCATCAGG
TAGTGCTGGGCACTCTAGAGATACTGCCACCGTAAGGTGTGAGGAAGGTGGGGATGACGTCAAATCAGCA
CGGCCCTTACGTCCGGGGCTACACACGTGTTNCAATGGGTGGTACAGAGAGTTGGTTGTACGCAAGTGCG
ATCTAATCCTAAAAACCATTCTCAGTTCGGACTGGGGTCTGCAACCCGACCCCACGAAGCTGGATTCGCT
AGTAATCGCGCATCAGCCATGGCGCGGTGAATACGTTCCCGGG
>164460359|gb|EU188983.1|Pv.112Q-3|
CTGAACCAGCCAAGTAGCGTGCAGGATGACGGCCCTATGGGTTGTAAACTGCTTTTATATGGGAATAAAG
TGAGGGACGTGTCCCTTATTGCATGTACCATACGAATAAGGACCGGCTAATTCCGTGCCAGCAGCCGCGG
TAATACGGAAGGTCCAGGCGTTATCCGGATTTATTGGGTTTAAAGGGAGCGTAGGCTGTTTGTTAAGCGT
GTTGTGAAATGTAGGAGCTCAACTTTTAGATTGCAGCGCGAACTGGCAGACTTGAGTGCGCACAACGTAG
GCGGAATTCATGGTGTAGCGGTGAAATGCTTAGATATCATGACGAACTCCGATTGCGAAGGCAGCTTACG
GGAGCGCAACTGACGCTAAAGCTCGAAGGTGCGGGTATCGAACAGGATTAGATACCCTGGTAGTCCGCAC
AGTAAACGATGGATGCCCGCTGTTAGCACCTAGTGTTAGCGGCTAAGCGAAAGCATTAAGCATCCCACCT
GGGGAGTACGCCGGCAACGGTGAAACTCAAAGGAATTGACGGGGGCCCGCACAAGCGGAGGAACATGTGG
TTTAATTCGATGATACGCGAGGAACCTTACCCGGGCTTGAATTGCAGATGTTTATATCAGAGATGATATA
TTCCCTTCGGGGCATTTGTGAAGGTGCTGCATGGTTGTCGTCAGCTCGTGCCGTGAGGTGTCGGCTTAAG
TGCCATAACGAGCGCAACC
>164460348|gb|EU188972.1|Pv.1BV-42|
CAGTGAGGAATATTGCCAATGGGCGAGAGCCTGAACCAGCCAAGTAGTCGTGCAGGATGACGGCCCTATG
TGCGTTGTAAACTGCTTTTATATGGCGAATAAATGTGAGGGACGTGTCCCTTATTGCATGTACCATACGA
ATAAGGACCGGCTAATTCCGTGCCAGCAGCCGCGGTAATACGGAAGGTCCAGGCGTTATCCGGATTTATT
GGGTTTAAAGGGAGCGTAGGCTGTTTGTTAAGCGTGTTGTGAAATGTAAGAGCTCAACTTTTAGATTGCA
GCGCGAACTGGCAGACTTGAGTGCGCACAACGNAGGCCGGAATTCATGGTGTAGCGGNGAAATGCTTAGA
TATCATGACGAACTCCGATTGCGAAGGCAGCTTACGGGAGCGCAACTGACGCNTATAGCTCGAAGGTGCG
GGTATCCGAACAGGATTAGATACCCTGGTAGTCCGCACAGTAAACGATGGATGCCCGCTGTTAGCACCTA
GTGTTAGCGGCTAAGCGAAAGNCATTAAGCATCCCACCTGGGGAGTACNCCGGCAACGGTGAAACTCAAA
GGAATTGACGGGGGCCCGCACTAGCGGAGGAACATGTGGTTTAATTCGATGATNGGCAAGGAACCTTACC
GGGGCTTGAATTGCATATGTTTATATCAGANATGATATATTCCCTTCGGGNCATTTTGTGAANGGGCTGC
ATGNNTGTNCTACANCNCGTGCCACGAGGNGTCCGCTTTAAGNCCCATAAACGAGCCCNACCCTTTTTNT
TNTATTTGCCATTNCGGGTAATGNC
>164460326|gb|EU188950.1|Pv.123Q-10|
TGAGGAATATTGNTCANTGGACGAGAGTCTGAACCAGCCAAGTAGCGTGCAGGATGACGGCCCTATGGGT
TGTAAACTGCTTTTGTTTGGGAATAATCGACATTACGTGTAATGTTTTGCATGTACCATTCGAATAAGGA
CCGGCTAATTCCGTGCCAGCAGCCGCGGTAATACGGAAGGTCCAGGCGTTATCCGGATTTATTGGGTTTA
AAGGGAGTGTAGGCGGTTGGTTAAGCGTGTTGTGAAATGTAGATGCTCAACATCTGACTTGCAGCGCGAA
CTGGCTGACTTGAGTACACACAACGTAGGCGGAATTCATGGTGTAGCGGTGAAATGCTTAGATATCATGA
AGAACTCCGATTGCGAAGGCAGCTTACGGGAGTGTTACTGACGCTTAAGCTCGAAGGTGCGGGTATCGAA
CAGGATTAGATACCCTGGTAGTCCGCACAGTAAACGATGGATGCCCGCTGTTAGCACCTGGTGTTAGCGG
CTAAGCGAAAGCATTAAGCATCCCACCTGGGGAGTACGCCGGCAACGGTGAAACTCAAAGGAATTGACGG
GGGCCCGCACAAGCGGAGGAACATGTGGTTTAATTCGATGATACGCGAGGAACCTTACCCGGGCTTGAAT
TGTAGGAGCACGATACAGAGATGTTGAGGTCCTTCGGGACTCCTATGAAGGTGCTGCATGGTTGTCGTCA
GCTCGTGCCGTGAGGTGTCGGCTTAAGTGCCATAACGAGCGCAACCCCTTTCCTTAGTTGCCATCAGGTA
ATGCTGGGCACTCTGAGGATACTGCCACCGTAAGGTGTGAGGAAGGTGGGGATGACGTCAAATCAGCACG
GCCCTTACGTCCGGGGCTACACACGTGTTACAATGGCCGGTACAGAATGTTGGTTGCATG
>294437|gb|L16483.1|P.disiens|
TACAATGGAGAGTTTGATCCTGGCTCAGGATGAACGCTAGCTATAGGCTTAACACATGCAAGTCGAGGGG
AACGGCAAAGAGTGCTTGCACACTTTGGACGTGACCGGCGCACGGGTGAGTAACGCGTATCCAACCTGCC
CTTTACTTGGGGATAACCCGTTGAAAGACGGACTAATACCCAATGATATTCATTGATGACATCTCATTTG
AATTAAAGATTTATCGGTATAGGATGGGGATGCGTCTGATTAGCTTGTTGGTGAGGTAACGGCTCACCAA
GGCAACGATCAGTAGGGGTTCTGAGAGGAAGGTCCCCCACATTGGAACTGAGACACGGTCCAAACTCCTA
CGGGAGGCAGCAGTGAGGAATATTGGTCAATGGACGAGAGTCTGAACCAGCCAAGTAGCGTGCAGGATGA

CGGCCCTATGGGTTGTAAACTGCNTTTGTTTGGGNATAATCGACATTACGTGTAATGTCTTTGCATGTAC
CATTCGAATAAGGACCGGCTNATTCCGTGCCAGCAGCCGCGGTAATACGGAAGGTCCAGGCGTTATCCGG
ATTTATTGGGTTTAAAGGGAGTGTAGGCGGTTGGTTAAGCGTGTTGTGAAATGTAGATGCTCAACATCTG
ACTTGCAGCGCGAACTGGCTGACTTGAGTACACACAACGTAGGCGGAATTCATGGTGTAGCGGTGAAATG
CTTAGATATCATGAAGAACTCCGATTGCGAAGGCAGCTTACGGGAGTGTTACTGACGCTTAAGCTCGAAG
GTGCGGGTATCGAACAGGATTAGATACCCTGGTAGTCCGCACAGTAAACGATGGATGCCCGCTGTTAGCA
CCTGGTGTTAGCGGCTAAGCGAAAGCATTAAGCATCCCACCTGGGGAGTACGCCGGCAACGGTGAAACTC
AAAGGAATTGACGGGGGCCCGCACAAGCGGAGGAACATGTGGTTTAATTCGATGATACGCGAGGAACCTT
ACCCGGGCTTGAATTGTAGGAGCACGATACAGAGATGTTGAGGTCCTTCGGGACTCCTATGAAGGTGCTG
CATGGTTGTCGTCAGCTCGTGCCGTGAGGTGTCGGCTTAAGTGCCATAACGAGCGCAACCCCTATCCTTA
GTTGCCATCAGGTAATGCTGGGCACTCTGAGGATACTGCCACCGTAAGGTGTGAGGAAGGTGGGGATGAC
GTCAAATCAGCACGGCCCTTACGTCCGGGGCTACACACGTGTTACAATGGCCGGTACAGAATGTTGGTTT
CATGTAAATGTAATCTAATCTTTAAAGCCGGTCCCAGTTCGGACTGAGGTCTGCAACCCGACCTCACGAA
GCTGGATTCGCTAGTAATCGCGCATCAGCCATGGCGCGGTGAATACGTTCCCGGGCCTTGTACACACCGC
CCGTCAAGCCATGAAAGCCGGGGGTGCCTGAAGTTCGTAACCGCAAGGAGCGACCTAGGGCAAAACTGGT
AATTGGGGC
>2108320|emb|Y13105.1|P.pallens|
CGCTAGCTATAGGCTTAACACATGCAAGTCGAGGGGAAACGGCATTATGTGCTTGCACATTTTGGACGTC
GACCGGCGCACGGGTGAGTATCGCGTATCCAACCTGCCCTTTACTTGGGGGATAACCCCGTTGAAAGACG
GCCTAATAACCCGATGTAATTCATTGATGGCATCAGATATGAATAAAAGATTTATCGGTAAAGGATGGGG
ATGCGTCTGATTAGCTTGTTGGTGAGGTAAAGGCTCACCAAGGCNACGATCAGTAGGGGTTCTGAGAGGA
AGGTCCCCCACATTGGAACTGAGACACGGTCCAAACTCCTACGGGAGGCAGCAGTGAGGAATATTGGTCA
ATGGGCGCAAGCCTGAACCAGCCAAGTAGCGTGCAGGAAGACGGCCCTATGGGTTGTAAACTGCTTTTAT
ACGAGAATAATTTGATGCACGTGTGCGTTATTGCATGTATCGTATGAATAAGGACCGGCTAATTCCGTGC
CAGCAGCCGCGGTAATACGGAAGGTCCAGGCGTTATCCGGATTTATTGGGTTTAAAGGGAGTGTAGGCGG
TTTGTTAAGCGTGTTGTGAAATTTAGGTGCTCAACATTTAACTTGCAGCGCGAACTGTCAAACTTGAGTA
CACGCAACGTATGCGGAATTCATGGTGTAGCGGTGAAATGCTTAGATATCATGAAGAACTCCGATTGCGA
AGGCAGCATACGGGAGTGTAACTGACGCTTAAGCTCGAAGGTGCGGGTATCGAACAGGATTAGATACCCT
GGTAGTCCGCACAGTAAACGATGGATGCCCGCTGTTAGCGCCTGGCGTTAGCGGCTAAGCGAAAGCATTA
AGCATCCCACCTGGGGAGTACGCCGGCAACGGTGAAACTCAAAGGAATTGACGGGGGCCCGCACAAGCGG
AGGAACATGTGGTTTAATTCGATGATACGCGAGGAACCTTACCCGGGCTTGAATTGCAGAGGAATTATTT
AGAGATAAATATGCCCTTCGGGGTCTCTGTGAAGGTGCTGCATGGTTGTCGTCAGCTCGTGCCGTGAGGT
GTCGGCTTAAGTGCCATAACGAGCGCAACCCCTTTTCTTAGTTGCCATCAGGTAATGCTGGGCACTCTGG
GAACACTGCCACCGCAAGGTGTGAGGAAGGTGGGGATGACGTCAAATCAGCACGGCCCTTACGTCCGGGG
CTACACACGTGTTACAATGGCCGGTACAGAGGGATGGTGTAATGTAAATTGCATCAAATCTTAAAAGCCG
GTCCCAGTTCGGACTGAGGTCTGCAACCCGACCTCACGAAGCTGGATTCGCTAGTAATCGCGCATCAGCC
ATGGCGCGGTGAATACGTTCCCGGGCCTTGTACACACCGCCCGTCAAGCCATGAAAGCCGGGGGTGCCCT
GAAGTTTCGTAACCGTAAGGAGCGACCTANGGGCAAAACTGGTAATTGGGGCTA
>164460325|gb|EU188949.1|Pv.123f-82|
TGAGGAATATTGGTCAATGGACGCAAGTCTGAACCAGCCAAGTAGCGTGCAGGATGACGGCCCTATGGGT
TGTAAACTGCTTTTATATGGGGATAAAGTGGGGAACGTGTTCCCTTTTGCAGGTACCATATGAATAAGGA
CCGGCTAATTCCGTGCCAGCAGCCGCGGTAATACGGAAGGTTCGGGCGTTATCCGGATTTATTGGGTTTA
AAGGGAGCGTAGGCCGTTTGGTAAGCGTGTTGTGAAATGTAGGAGCTCAACTTCTAGATTGCAGCGCGAA
CTGTCAGACTTGAGTGCGCACAACGTAGGCGGAATTCATGGTGTAGCGGTGAAATGCTTAGATATCATGA
AGAACTCCGATTGCGAAGGCAGCTTACGGGAGCGCAACTGACGCTGAAGCTCGAAGGTGCGGGTATCGAA
CAGGATTAGATACCCTGGTAGTCCGCACAGTAAACGATGGATGCCCGCTGTTAGCACCTAGTGTTAGCGG
CTAAGCGAAAGCATTAAGCATCCCACCTGGGGAGTACGCCGGCAACGGTGAAACTCAAAGGAATTGACGG
GGGCCCGCACAAGCGGAGGAACATGTGGTTTAATTCGATGATACGCGAGGAACCTTACCCGGGCTTGAAT
TGCAGATGAACGATTTAGAGATAATGAGGTCCTTCGGGACATCTGTGAAGGTGCTGCATGGTTGTCGTCA
GCTCGTGCCGTGAGGTGTCGGCTTAAGTGCCATAACGAGCGCAACCCCTTTCTTTAGTTGCCATCAGGTT
CTGCTGGGCACTCTGGAGATACTGCCACCGTAAGGTGTGAGGAAGGTGGGGATGACGTCAAATCAGCACG
GCCCTTACGTCCGGGGCTACACACGTGTTACAATGGGTGGTACAGATAGTTGGTCGTGTG
>294429|gb|L16475.1|P.bivia|
ACAATGGAGAGTTTGATCCTGGCTCAGGATNAACGCTAGCTATAGGCTTAACACATGCAAGTCGAGGGGC

AGCGAATAGATAGCTTGCTATTTATGTCGGCGACCGGCGCACGGGTGAGTAACGCGTATCCAACCTACCC
ATAACTAAGGGATAACCCAGCGAAAGTTGGACTNATACCTNATGTATTCGTTTGATCTCATGAGATTNCG
AATAAAGATTTATCGGTTATGGATGGGGATGCGTCTGATTAGCTTGTTGGCGGGGTAACGGCCCACCAAG
GCAACGATCAGTAGGGGTTCTGAGAGGAAGGTCCCCCACATTGGAACTGAGACACGGTCCNAACTCCTAC
GGGAGGCAGCAGTGAGGAATATTGGTCAATGGACGCAAGTCTGAACCAGCCAAGTAGCGTGCAGGATGAC
GGCCCTATGGGTTGTAAACTNCTTTTATATGGGGATAAAGTGGGGAACGTGTTCCCNTTTGCAGGTACCA
TATGAATAAGGACCGGCTNATTCCGTGCCAGCAGCCGCGGTAATACGGAAGGTTCGGGCGTTATCCGGAT
TTATTGGGTTTAAAGGGAGCGTAGGCCGTTTGGTAAGCGTGTTGTGAAATGTAGTAGCTCAACTTCTAGA
TTGCAGCGCGAACTGTCAGACTTGAGTGCGCACAACGTAGGCGGAATTCATGGTGTAGCGGTGAAATGCT
TAGATATCATGAAGAACTCCGATTGCGAAGGCAGCTTACGGGAGCGCAACTGACGCTGAAGCTCGAAGGT
GCGGGTATCGAACAGGATTAGATACCCTGGTAGTCCGCACAGTAAACGATGGATGCCCGCTGTTAGCACC
TAGTGTTAGCGGCTAAGCGAAAGCATTAAGCATCCCACCTGGGGAGTACGCCGGCAACGGTGAAACTCAA
AGGAATTGACGGGGGCCCGCACAAGCGGAGGAACATGTGGTTTAATTCGATGATACGCGAGGAACCTTAC
CCGGGCTTGAATTGCAGATGAACGATTTAGAGATAATGAGGTCCTTCGGGACATCTGTGAAGGTGCTGCA
TGGTTGTCGTCAGCTCGTGCCGTGAGGTGTCGGCTTAAGTGCCATAACGAGCGCAACCCCNNTCTTTAGT
TGCCATCAGGTTCTGCTGGGCACTCTGGAGATACTGCCACCGTAAGGTGTGAGGAAGGTGGGGATGACGT
CAAATCAGCACGGCCCTTACGTCCGGGGCTACACACGTGTTACAATGGGTGGTACAGATAGTTGGTCGTG
TGCAAATACGATCTAATCCTTAAAACCATTCTCAGTTCGGACTGGGGTCTGCAACCCGACCCCACGAAGC
TGGATTCGCTAGTAATCGCGCATCAGCCATGGCGCGGTGAATACGTTCCCGGGCCTTGTACACACCGCCC
GTCAAGCCATGAAAGCCGGGGGTGCCTGAAGTTCGTGACCGTAAGGATCGACCTAGGGCAAAACTGGTAA
T
>164460358|gb|EU188982.1|Pv.112Q-53|
CGTCTGCATNGCAGCGCGAACTGGAACGCTTGAGTACGCGCAACGTTGGCGGAATTNGTCGTGTAGCGGT
GAAATGCTTAGATATGACGAAGAACTCCGATTGCGAAGGCAGCTGACGGGAGCGGCACTGACGCTTAAGC
TCGAAGGTGCGGGTATCAAACAGGATTAGATACCCTGGTAGTCCGCACGGTAAACGATGGATGCTCGCTG
TGTGCCTTTTGTGGTACGCGGCTAAGCGAAAGCGTTAAGCATCCCACCTGGGGAGTACGCCGGCAACGGT
GAAACTCAAAGGAATTGACGGGGGCCCGCACAAGCGGAGGAACATGTGGTTTAATTCGATGATACGCGAG
GAACCTTACCCGGGCTTGAACTGCCGGCGAACGATCCAGAGATGGTGAGGCCCCTCGGGGCGCCGGTGGA
GGTGCTGCATGGTTGTCGTCAGCTCGTGCCGTGAGGTGTCGGCTTAAGTGCCATAACGAGCGCAACCCCT
CTCCGTAGTTGCCATCAGGTAGTGCTGGGCACTCTGCGGACACTGCCACCGCAAGGTGCGAGGAAGGTGG
GGATGACGTCAAATCAGCACGGCCCTTACGTCCGGGGCTACACACGTGTTACAATGGGGGGCACANCGAG
TCGGCCGCGCGCAAGCTCGGTCCAATCAAGAAATCCCCCCTCAGTTCGGACTGGGGTCTGCAACCCGACC
CCACGAAGCTGGATTCGCTAGTAATCGCGCATCAGCCATGGCGCGGTGAATACGTTCCCGGG
>164460378|gb|EU189002.1|Pv.123b-5|
TGAGGAATATTGGTCAATGGGCGGGAGCCTGAACCAGCCAAGTAGCGTGCAGGAAGACGGCCCTATGGGT
TGTAAACTGCTTTTATGCGGGGATAAAGGAGTCCACGTGTGGGCTTTTGCAGGTACCGCATGAATAAGGA
CCGGCTAATTCCGTGCCAGCAGCCGCGGTAATACGGAAGGTCCGGGCGTTATCCGGATTTATTGGGTTTA
AAGGGAGCGTAGGCCGCGTTTTAAGCGTGTTGTGAAATGTAGGCGCCCAACGTCTGCATCGCAGCGCGAA
CTGGAACGCTTGAGTACGCGCAACGTTGGCGGAATTCGTCGTGTAGCGGTGAAATGCTTAGATATGACGA
AGAACTCCGATTGCGAAGGCAGCTGACGGGAGCGGCACTGACGCTTAAGCTCGAAGGTGCGGGTATCAAA
CAGGATTAGATACCCTGGTAGTCCGCACGGTAAACGATGGATGCTCGCTGTGTGCCTTTTGTGGTACGCG
GCTAAGCGAAAGCGTTAAGCATCCCACCTGGGGAGTACGCCGGCAACGGTGAAACTCAAAGGAATTGACG
GGGGCCCGCACAAGCGGAGGAACATGTGGTTTAATTCGATGATACGCGAGGAACCTTACCCGGGCTTGAA
CTGCCGGCGAACGATCCAGAGATGGTGAGGCCCCTCGGGGCGCCGGTGGAGGTGCTGCATGGTTGTCGTC
AGCTCGTGCCGTGAGGTGTCGGCTTAAGTGCCATAACGAGCGCAACCCCTCTCCGTAGTTGCCATCAGGT
AGTGCTGGGCACTCTGCGGACACTGCCACCGCAAGGTGCGAGGAAGGTGGGGATGACGTCAAATCAGCAC
GGCCCTTACGTCCGGGGCTACACACGTGTTACAATGGGGGGCACAGCGAGTCGGCCGCGC
>164460323|gb|EU188947.1|Pv.123f2-17|
TGAGGAATATTGGTCAATGGGCGTGAGCCTGAACCAGCCAAGTAGCGTGCAGGAAGACGGCCCTATGGGT
TGTAAACTGCTTTTATGCGGGGATAAAGGAGTCCACGTGTGGATTTTTGCAGGTACCGCATGAATAAGGA
CCGGCTAATTCCGTGCCAGCAGCCGCGGTAATACGGAAGGTCCGGGCGTTATCCGGATTTATTGGGTTTA
AAGGGAGCGTAGGCCGCGTTTTAAGCGTGTTGTGAAATGTAGACGCCCAACGTCTGCATCGCAGCGCGAA
CTGGGACGCTTGAGTACGCGCAACGTTGGCGGAATTCGTCGTGTAGCGGTGAAATGCTTAGATATGACGA
GGAACTCCGATTGCGAAGGCAGCTGACGGGAGCGGCACTGACGCTTAAGCTCGAAGGTGCGGGTATCAAA

CAGGATTAGATACCCTGGTAGTCCGCACAGTAAACGATGGATGCCCGCTGTGTGCCATTTATGGTACGCG
GCTAAGCGAAAGCGTTAAGCATCCCACCTGGGGAGTACGCCGGCAACGGTGAAACTCAAAGGAATTGACG
GGGGCCCGCACAAGCGGAGGAACATGTGGTTTAATTCGATGATACGCGAGGAACCTTACCCGGGCTTGAA
CTGCCGGTGCACGATCCAGAGATGGTGAGGCCCTTCGGGGCGCCGGTGGAGGTGCTGCATGGTTGTCGTC
AGCTCGTGCCGTGAGGTGTCGGCTTAAGTGCCATAACGAGCGCAACCCCTCTCCGTAGTTGCCATCAGGT
AATGCTGGGCACTCTGCGGATACTGCCACCGCAAGGTGCGAGGAAGGTGGGGATGACGTCAAATCAGCAC
GGCCCTTACGTCCGGGGCTACACACGTGTTACAATGGGGGGCACAGCAAGTAGGCCGCGC
>40363455|dbj|AB108826.1|P.salivae|
AGAGTTTGATCCTGGCTCAGGATGAACGCTAGCTACAGGCTTAACACATGCAAGTCGAGGGGAAACGACA
TTGAAGCTTGCTTCGATGGGCGTCGACCGGCGCACGGGTGAGTAACGCGTATCCAACCTGCCTCTGACTA
AGGGATAACCCGGCGAAAGTCGGACTAATACCTTATGAGGTTTTCAGCAGACATCTAACGAAAACGAAAG
ATTTATCGGTCAGTGATGGGGATGCGTCTGATTAGCTTGTTGGCGGGGTAACGGCCCACCAAGGCAACGA
TCAGTAGGGGTTCTGAGAGGAAGGTCCCCCACATTGGAACTGAGACACGGTCCAAACTCCTACGGGAGGC
AGCAGTGAGGAATATTGGTCAATGGGCGAGAGCCTGAACCAGCCAAGTAGCGTGCAGGATGACGGCCCTA
TGGGTTGTAAACTGCTTTTATGTGGGGATAAAGTGAGCTACGTGTAGTTTATTGCAGGTACCACATGAAT
AAGGACCGGCTAATTCCGTGCCAGCAGCCGCGGTAATACGGAAGGTCCAGGCGTTATCCGGATTTATTGG
GTTTAAAGGGAGCGTAGGCCGTGGATTAAGCGTGTTGTGAAATGTAGACGCTCAACGTCTGAATTGCAGC
GCGAACTGGTTCACTTGAGTATGCACAACGTAGGCGGAATTCGTCGTGTAGCGGTGAAATGCTTAGATAT
GACGAAGAACTCCGATTGCGAAGGCAGCTTACGGGAGCACAACTGACGCTGAAGCTCGAAGGTGCGGGTA
TCAAACAGGATTAGATACCCTGGTAGTCCGCACAGTAAACGATGGATGCCCGTTGTTAGTTCACTTATGA
ATTAGCGACCAAGCGAAAGCATTAAGCATCCCACCTGGGGAGTACGCCGGCAACGGTGAAACTCAAAGGA
ATTGACGGGGGCCCGCACAAGCGGAGGAACATGTGGTTTAATTCGATGATACGCGAGGAACCTTACCCGG
GCTTGAATTGCAGACGAACGATTCAGAGATGATGAGGCCCTTCGGGGCGTCTGTGAAGGTGCTGCATGGT
TGTCGTCAGCTCGTGCCGTGAGGTGTCGGCTTAAGTGCCATAACGAGCGCAACCCCTTTTTTCAGTTGCC
ATCAGGTGATGCTGGGCACTCTGGAGATACTGCCACCGTAAGGTGTGAGGAAGGTGGGGATGACGTCAAA
TCAGCACGGCCCTTACGTCCGGGGCTACACACGTGTTACAATGGCCGGTACAGAGCGTTGGTCGTGTGCA
AATACGATCTAATCCTTAAAGCCGGTCCCAGTTCGGACTGGGGTCTGCAACCCGACCCCACGAAGCTGGA
TTCGCTAGTAATCGCGCATCAGCCATGGCGCGGTGAATACGTTCCCGGGCCTTGTACACACCGCCCGTCA
AGCCATGAAAGCCGGGGGTGCCTGAAGTCTGTGACCGCAAGGAACGGCCTAGGGCAAAACTGGTGATTGG
GGCTAAGTCGTAACAAGGTAACC
>58339332|gb|AY880055.1|Pv.T05-04|
CGGCATTAAGTGCTTGCACTTTTTGGACGTCGACCGGCGCACGGGTGAGTAACGCGTATCCAACCTTCCC
ATGACTAAGGGATAACCTGCCGAAAGGCAGACTAATACCTTATGGTCTTCACTGACGGCATCAGATGTGA
AGTAAAGATTTATCGGTTATGGATGGGGATGCGTCTGATTAGCTTGTTGGCGGGGTAACGGCCCACCAAG
GCAACGATCAGTAGGGGTTCTGAGAGGAAGGTCCCCCACATTGGAACTGAGACACGGTCCAAACTCCTAC
GGGAGGCAGCAGTGAGGAATATTGGTCAATGGGCGAGAGCCTGAACCAGCCAAGTAGCGTGCAGGATGAC
GGCCCTATGGGTTGTAAACTGCTTTTGTATGGGGATAAAGTCAATCACGTGTGATTGTTTGCAGGTACCA
TACGAATAAGGACCGGCTAATTCCGTGCCAGCAGCCGCGGTAATACGGAAGGTCCGGGCGTTATCCGGAT
TTATTGGGTTTAAAGGGAGCGTAGGCTGGAGATTAAGTGTGTTGTGAAATGTAGACGCTCAACGTCTGAC
TTGCAGCGCATACTGGTTTCCTTGAGTACGCACAACGTTGGCGGAATTCGTCGTGTAGCGGTGAAATGCT
TAGATATGACGAAGAACTCCGATTGCGAAGGCAGCTGACGGGAGCGCAACTGACGCTGAAGCTCGAAGGT
GCGGGTATCGAACAGGATTAGATACCCTGGTAGTCCGCACAGTAAACGATGGATGCCCGCTGTTGGTACT
TGGTATCAGCGGCTAAGCGAAAGCATTAAGCATCCCACCTGGGGAGTACGCCGGCAACGGTGAAACTCAA
AGGAATTGACGGGGGCCCGCACAAGCGGAGGAACATGTGGTTTAATTCGATGATACGCGAGGAACCTTAC
CCGGGCTTGAATTGCAGAGGAAGGATTTAGAGATAATGACGCCCTTCGGGGTCTCTGTGAAGGTGCTGCA
TGGTTGTCGTCAGCTCGTGCCGTGAGGTGTCGGCTTAAGTGCCATAACGAGCGCAACCCCTGTCTTTAGT
TGCCATCAGGTTAAGCTGGGCACTCTGGAGATACTGCCACCGTAAGGTGTGAGGAAGGTGGGGATGACGT
CAAATCAGCACGGCCCTTACGTCCGGGGCTACACACGTGTTACAATGGCCGGTACAGAGGGACGGTGTAA
TGTAAATTGCATCTAATCTTGAAAGCCGGTCCCAGTTCGGACTGGGGTCTGCAACCCGACCCCACGAAGC
TGGATTCGCTAGTAATCGCGCATCAGCCATGGCGCGGTGAATACGTTCCCGGGCC
>66878726|gb|AY959020.1|Pv.rRNA247|
GCCCTTAGAGTTTGATCCTGGCTCAGGATGAACGCTAGCTACAGGCTTAACACATGCAAGTCGAGGGGAA
ACGGTATTTAGTGCTTGCACCGAATGGACGTCGACCGGCGCACGGGTGAGTAACGCGTATCCAACCTTCC
CATAACTAAGGGATAACCTGCCGAAAGGCAGACTAATACCTTATGTAATCCTTTGATGGCATCAGATAAG

GATAAAAGATTTATCGGTTATGGATGGGGATGCGTCTGATTAGCTTGTTGGCGGGGTAACGGCCCACCAA
GGCAACGATCAGTAGGGGTTCTGAGAGGAAGGTCCCCCACATTGGAACTGAGACACGGTCCAAACTCCTA
CGGGAGGCAGCAGTGAGGAATATTGGTCAATGGACGGAAGTCTGAACCAGCCAAGTAGCGTGCAGGATGA
CGGCCCTATGGGTTGTAAACTGCTTTTGTATGGGGATAAAGTCAATCACGTGTGATTGTTTGCAGGTACC
ATACGAATAAGGACCGGCTAATTCCGTGCCAGCAGCCGCGGTAATACGGAAGGTTCGGGCGTTATCCGGA
TTTATTGGGTTTAAAGGGAGCGTAGGCCGGAGATTAAGTGTGTTGTGAAATGTAGACGCTCAACGTCTGA
CTTGCAGCGCATACTGGTTTCCTTGAGTACGCACAACGTTGGCGGAATTCGTCGTGTAGCGGTGAAATGC
TTAGATATGACGAAGAACTCCGATTGCGAAGGCAGCTGACGGGAGCGCAACTGACGCTGAAGCTCGAAGG
TGCGGGTATCGAACAGGATTAGATACCCTGGTAGTCCGCACAGTAAACGATGGATGCCCGCTGTTGGTAC
CTAGTATCAGCGGCTAAGCGAAAGCATTAAGCATCCCACCTGGGGAGTACGCCGGCAACGGTGAAACTCA
AAGGAATTGACGGGGGCCCGCACAAGCGGAGGAACATGTGGTTTAATTCGATGATACGCGAGGAACCTTA
CCCGGGCTTGAATTGCAGAGGAAGGATTTAGAGATAATGACGCCCTTCGGGGTCTCTGTGAAGGTGCTGC
ATGGTTGTCGTCAGCTCGTGCCGTGAGGTGTCGGCTTAAGTGCCATAACGAGCGCAACCCCTCTCTTCAG
TTGCCATCAGGTTAAGCTGGGCACTCTGGAGACACTGCCACCGTAAGGTGTGAGGAAGGTGGGGATGACG
TCAAATCAGCACGGCCCTTACGTCCGGGGCTACACACGTGTTACAATGGCCGGTACAGAGGGACGGTGTA
ATGTAAATTGCATCTAATCTTGAAAGCCGGTCCCAGTTCGGACTGGGGTCTGCAACCCGACCCCACGAAG
CTGGATTCGCTAGTAATCGCGCATCAGCCATGGCGCGGTGAATACGTTCCCGGGCCTTGTACACACCGCC
CGTCAAGCCATGAAAGCCGGGGGTGCCTGAAGTCCGTGACCGGAAGGATCGGCCTAGGGCAAAACTGGTA
ATTGGGGCTAAGTCGTAACAAGGTAACCGTAAAGGG
>6942139|gb|AF218617.1|P.ruminicola|
AGAGTTTGATCCTGGCTCAGGATGAACGCTAGCTACAGGCTTAACACATGCAAGTCGAGGGGTAACGACA
GCGAAAGCTTGCTTTTGCTGGGCGACGACCGGCGCACGGGTGAGTAACGCGTATCCAACCTGCCCATAAC
TAAGGGATAATCCGTAGAAATGCGGTCTAATACCTTATGTGTTCCGACGAAGACATCTGAATTGGAATAA
AGATTTATCGGTTATGGATGGGGATGCGTCTGATTAGATTGTTGGCGGGGCAACGGCCCACCAAGTCTAC
GATCAGTAGGGGTTCTGAGAGGAAGGTCCCCCACATTGGAACTGAGACACGGTCCAAACTCCTACGGGAG
GCAGCAGTGAGGAATATTGGTCAATGGGCGAGAGCCTGAACCAGCCAAGTAGCGTGCAGGAWGACGGCCC
TATGGGTTGTAAACTGCTTTTGTATGGGRATAAAGTGCTCCACGTGTGGAGTTTTGTAGGTACCATACGA
ATAAGGACCGGCTAATTCCGTGCCAGCAGCCGCGGTAATACGGAAGGTCCGGGCGTTATCCGGATTTATT
GGGTTTAAAGGGAGCGTAGGCCGTGGATTAAGTGTGTTGTGAAATGTAGGCGCTCAACGTCTGACTTGCA
GCGCATACTGGTCCACTTGAGTGCGCACAACGCGGGCGGAATTTGTCGTGTAGCGGTGAAATGCTTAGAT
ATGACGAAGAACCCCGATTGCGAAGGCAGCTCGCGGGAGCGCAACTGACGCTGAAGCTCGAAAGTGCGGG
TATCGAACAGGATTAGATACCCTGGTAGTCCGCACGGTAAACGATGGATGCCCGCTTTTCGGTCATTAGA
CCGTGAGGCCAAGTGAAAACATTAAGCATCCCACCTGGGGAGTACGCCGGCAACGGTGAAACTCAAAGGA
ATTGACGGGGGCCCGCACAAGCGGAGGAACATGTGGTTTAATTCGATGATACGCGAGGAACCTTACCCGG
GCTTGAATTGCAGATGAAGGATCCAGAGATGGTGACGCCCTTCGGGGCATCTGTGAAGGTGCTGCATGGT
TGTCGTCAGCTCGTGCCGTGAGGTGTCGGCTCAAGTGCCATAACGAGCGCAACCCCTCTCTTCAGTTGCC
ATCAGGCTATGCTGGGCACTCTGGAGACACTGCCACCGCAAGGTGTGAGGAAGGTGGGGATGACGTCAAA
TCAGCACGGCCCTTACGTCCGGGGCTACACACGTGTTACAATGGGGGGTACAGAAAGCCGGCTGCCCGCA
AGGTCAGTCCAATCAAGAAAGCCCTCCTCAGTTCGGACTGGGGTCTGCAACCCGACCCCACGAAGCTGGA
TTCGCTAGTAATCGCGCATCAGCCATGGCGCGGTGAATACGTTCCCGGGCCTTGTACACACCGCCCGTCA
AGCCATGAAAGCCGGGGGTGCCTGAAGTCCGTGACCGCAAGGATCGGCCTAGGGCAAAACTGGTAATTGG
GGCTAAGTCGTAACAAGGTAGCCGT
>33943622|gb|AY350613.1|P.bergensis|
GATGAACGCTAGCTACAGGCTTAACACATGCAAGTCGAGGGGAAACGGCATTTGGTGCTTGCACCATTTG
GACGTCGACCGGCGCACGGGTGAGTAACGCGTATCCAACCTGCCCATAAGTAGGGTATAACCCGCAGAAA
TGCGGACTAATCCCCTATGTTGTTCAAAGACGGCATCAGATTTGAACCAAAGGCTTGCCGCTTATGGATG
GGGATGCGTCCGATTAGCTTGTTGGTGAGGTAACGGCTCACCAAGGCTTCGATCGGTAGGGGTTCTGAGA
GGAAGGCCCCCCACATAGGAACTGAGACACGGTCCTAACTCCTACGGGAGGCAGCAGTGAGGAATATTGG
TCAATGGGCGAGAGCCTGAACCAGCCAAGTAGCGTGCAGGACGACGGCCCTATGGGTTGTAAACTGCTTT
TATGCGGGGATAACCGGCCTCACGTGTGAGGCCCTGCAGGTACCGCATGAATAAGGACCGGCTAATTCCG
TGCCAGCAGCCGCGGTAATACGGAAGGTCCGGGCGTTATCCGGATTTATTGGGTTTAAAGGGAGCGTAGG
CCGTGGGTTAAGTGTGTTGTGAAATCCGGTTGCTCAACATCCGGTTTGCAGCGCATACTGTCCCACTTGA
GTGCGCACAACGCAGGCGGAATTCGTCGTGTAGCGGTGAAATGCTTAGATATGACGAGGAACCCCGATTG
CGAAGGCAGCTTGCGGGAGCGCAACTGACGCTTAAGCTCGAAGGTGCGGGTATCAAACAGGATTAGATAC

CCTGGTAGTCCGCACGGTAAACGATGGATGCCCGCTGTTTGCCCTATTTTGGGCATGCGGCCAAGCGAAA
GCATTAAGCATCCCACCTGGGGAGTACGCCGGCAACGGTGAAACTCAAAGGAATTGACGGGGGCCCGCAC
AAGCGGAGGAACATGTGGTTTAATTCGATGATACGCGAGGAACCTTACCCGGGCTTGAACTGCCAGTGAA
CGATACAGAGATGTTGAGGCCCTTCGGGGCGCTGGTGGAGGTGCTGCATGGTTGTCGTCAGCTCGTGCCG
TGAGGTGTCGGCTTAAGTGCCATAACGAGCGCAACCCTTTTCCACAGTTGCCATCAGGTCATGCTGGGCA
CTCTGTGGATACTGCCGCCGCAAGGTGTGAGGAAGGTGGGGATGACGTCAAATCAGCACGGCCCTTACGT
CCGGGGCTACACACGTGTTACAATGGGGGGCACAGCGTGCCGTACGTGCGNAAGCACGTTCCAATCATGA
AATCCCCCCTCAGTTCGGACTGGGGTCTGCAACCCGACCCCACGAAGCTGGATTCGCTAGTAATCGCGCA
TCAGCCATGGCGCGGTGAATACGTTCCCGGGCCTTGTACACACCGCCCGTCAAGCCATGAAAGCCGGGGG
TGCCTGAAGTCCGTAACCGCAAGGGGCGGCCTAGGGCAAGACCGGTGATTGGGGCT
>30908827|gb|AY278625.1|P.genomosp|
GATGAACGCTAGCTACAGGCTTAACACATGCAAGTCGAGGGGAAACGGGAGAATAGCTTGCTATTCTTTG
TCGTCGACCGGCGCACGGGTGAGTAACGCGTATCCAACCTTCCCATAACTAAGGAATACCCCGTAGAAAT
GCGGCCTAATCCCTTATGGTCTCTTACGCTGAGATCGGAATAAGAGTAAAGATTTATCGGTTATGGATGG
GGATGCGTCTGATTAGCTTGCTGGCGGGGTAACGGCCCACCAGGGCATCGATCAGTAGGGGTTCTGAGAG
GAAGGTCCCCCACATTGGAACTGAGACACGGTCCAAACTCCTACGGGAGGCAGCAGTGAGGAATATTGGT
CAATGGGCGAGAGCCTGAACCAGCCAAGTAGCGTGCAGGATGACGGCCCTATGGGTTGTAAACTGCTTTT
TAAGGGGAATAAAGAGGGACACGCGTGTCCTGTTGCATGTACCCTTAGAATAAGGACCGGCTAATTCCGT
GCCAGCAGCCGCGGTAATACGGAAGGTCCAGGTGTTATCCGGATTTATTGGGTTTAAAGGGAGCGTAGGC
CGCAGGTTAAGTGTGTTGTGAAAAGCAGTCGCCCAACGTCTGCCTTGCAGCGCAAACTGTCCTGCTTGAG
TGCGCACAACGCAGGCGGAATCCGTCGTGTAGCGGTGAAATGCTTAGATATGACGAAGAACTCCGATTGC
GAAGGCAGCTTGCGGGAGCGCAACTGACGCTGAAGCTCGAAAGTGCGGGTATCGAACAGGATTAGATACC
CTGGTAGTCCGCACGGTAAACGCTGGATGCCCGTTTTTGGCTTATTTTAAGTCAGAGACCAAGCGAAAGC
ATTAAGCATCCCACCTGGGGAGTACGCCGGCAACGGTGAAACTCAAAGGAATTGACGGGGGCCCGCACAA
GCGGAGGAACATGTGGTTTAATTCGATGATACGCGAGGAACCTTACCCGGGCTTGAATTGCCAGAGACGG
CTCCAGAGATGGAGCTTCCCTTCGGGGCTTTGGTGAAGGTGCTGCATGGTTGTCGTCAGCTCGTGCCGTG
AGGTGTCGGCTTAAGTGCCATAACGAGCGCAACCCCTTTCTTCAGTTGCCATCGGGTAATGCCGGGCACT
CTGTAGATACTGCCGCCGTAAGGTGTGAGGAAGGTGGGGATGACGTCAAATCAGCACGGCCCTTACGTCC
GGGGCTACACACGTGTTACAATGGGGGGCACAGCGAGCGGGATGCACGCAAGGTCATTCAAATCAATAAA
TCCCCCCTCAGTTCGGACTGGGGTCTGCAACCCGACCCCACGAAGCTGGATTCGCTAGTAATCGCGCATC
AGCCATGGCGCGGTGAATACGTTCCCGGGCCTTGTACACACCGCCCGTCAAGCCATGAAAGCCGGGGGTG
CCTGAAGTCCGTGACCGCAAGGATCGGCCTAGGGCAAAACTGGTAATTGGGGCTAAGTCGTAACAAGGTA
GCCGTACCGGAAGGTGC
>32492914|gb|AY323522.1|P.oralis|
GATGAACGCTAGCTACAGGCTTAACACATGCAAGTCGCGGGGCATCATGGAGGTTGTTTTCAACTTTTGA
TGGCGACCGGCGCACGGGTGAGTAACGCGTATCCAACCTTCCCATTACTACGGCATACCCCGTTGAAAGG
CGGCCTAATTCCGTATGCAGTCCGAAGCAGTCATCTAATTTGGACGAAAGGTATATTCTATCGGTAATGG
ATGGGGATGCGTCCGATTAGCCAGCCGGCGGGGTAACGGCCCACCGGGGCATCGATCGGTAGGGGTTCTG
AGAGGAAGGTCCCCCACACTGGAACTGAGACACGGTCCAGACTCCTACGGGAGGCAGCAGTGAGGAATAT
TGGTCAATGGGCGTAAGCCTGAACCAGCCAAGTAGCGTGCAGGATGACGGCCCTATGGGTTGTAAACTGC
TTTTATGCGGGGATAAAGTTGGGGACGTGTCCCTTTTTGCAGGTACCGCATGAATAAGGACCGGCTAATT
CCGTGCCAGCAGCCGCGGTAATACGGAAGGTCCGGGCGTTATCCGGATTTATTGGGTTTAAAGGGAGCGT
AGGCAGTTTTTTAAGCGTGCTGTGAAATGTACCGGCTCAACCGGTGATGTGCAGCGCGAACTGGGAATCT
TGAGTACGCAGTAAGCAGGCGGAATTCGTGGTGTAGCGGTGAAATGCTTAGATATCACGAAGAACTCCGA
TTGCGTAGGCAGCTTGCTGTAGCGTAACTGACGCTGAAGCTCGAAAGTGCGGGTATCGAACAGGATTAGA
TACCCTGGTAGTCCGCACGGTAAACGATGGATGCCCGCTGTCGGCCTTTTTTGGTCGGTGGCCAAGCGAA
AGCGTTAAGCATCCCACCTGGGGAGTACGCCGGCAACGGTGAAACTCAAAGGAATTGACGGGGGCCCGCA
CAAGCGGAGGAACATGTGGTTTAATTCGATGATACGCGAGGAACCTTACCCGGGCTTGAACTGCCATTGA
CTTGAATAGAGATATTCATTCCCTTCGGGGCATTGGTGGAGGTGCTGCATGGTTGTCGTCAGCTCGTGCC
GTGAGGTGTCGGCTTAAGTGCCATAACGAGCGCAACCCCTTTTTTCAGTTGCCATCGGTTAAAGCCGGGC
ACTCTGTGAATACTGCCGCCGTAAGGTGTGAGGAAGGTGGGGATGACGTCAAATCAGCACGGCCCTTACG
TCCGGGGCTACACACGTGTTACAATGGCGCGTACAGCGAGTCGGCCGTATGTAAATGCGGTCCAATCCTT
AAAACGTGCCTCAGTTCGGACTGGGGTCTGCAACCCGACCCCACGAAGCTGGATTCGCTAGTAATCGCGC
ATCAGCCATGGCGCGGTGAATACGTTCCCGGGCCTTGTACACACCGCCCGTCAAGCCATGAAAGCCGGGG

GTGCCTGAAGTCCGTGACCGCAAGGGTCGGCCTAAGGCAATACTGGTAATTGGGGCTAAGTCGTAACAAG
GTAGCCGTACCGGAAGGTGCGGCTGGAACACCTCCTTTCT
>146142527|gb|EF534315.1|P.maculosa|
GATGAACGCTAGCTACAGGCTTAACACATGCAAGTCGAGGGGAAACGATGAGAGAGCTTGCTCTCTCAGG
CGTCGACCGGCGCACGGGTGAGTAACGCGTATCCAACCTGCCTCTGACCAAGGGATAACCCGTCGAAAGT
CGGACTAATACCTTATGCAGTCGTCGGAAGTCATCTGATGACGACGAAAGATTTCATCGGTGAGAGATGG
GGATGCGTCTGATTAGCTTGTTGGCGGGGTAACGGCCCACCAAGGCGACGATCAGTAGGGGTTCTGAGAG
GAAGGTCCCCCACATTGGAACTGAGACACGGTCCAAACTCCTACGGGAGGCAGCAGTGAGGAATATTGGT
CAATGGGCGCGAGCCTGAACCAGCCAAGTAGCGTGCAGGATGACGGCCCTATGGGTTGTAAACTGCTTTT
ATGCGGGGATAAAGTGAGGGACGTGTCCTTCATTGCAGGTACCGCATGAATAAGGACCGGCTAATTCCGT
GCCAGCAGCCGCGGTAATACGGAAGGTCCGGGCGTTATCCGGATTTATTGGGTTTAAAGGGAGCGTAGGC
CGTGGATTAAGCGTGTTGTGAAATGTAGACGCTCAACGTCTGACTTGCAGCGCGAACTGGTCCACTTGAG
TGTGCACAACGCAGGCGGAATTCGTCGTGTAGCGGTGAAATGCTTAGATATGACGAAGAACCCCGATTGC
GAAGGCAGCTTGCGGGAGCACGACTGACGCTGAAGCTCGAAAGTGCGGGTATCGAACAGGATTAGATACC
CTGGTAGTCCGCACGGTAAACGATGGATGCCCGTTGTCAGGCTGTATCAGTCTGGTGACCAAGCGAAAGC
ATTAAGCATCCCACCTGGGGAGTACGCCGGCAACGGTGAAACTCAAAGGAATTGACGGGGGCCCGCACAA
GCGGAGGAACATGTGGTTTAATTCGATGATACGCGAGGAACCTTACCCGGGCTTGAATTGCAGACGTACG
ATTCAGAGATGAAGAGGCCCTTCGGGGCGTCTGTGAAGGTGCTGCATGGTTGTCGTCAGCTCGTGCCGTG
AGGTGTCGGCTTAAGTGCCATAACGAGCGCAACCCCTATCCTCGGTTGCCATCGGGTAATGCCGGGCACT
CCGTGGAGACTGCCACCGTAAGGTGTGAGGAAGGTGGGGACGACGTCAAATCAGCACGGCCCTTACGTCC
GGGGCTACACACGTGTTACAATGGCCGGTACAGCGAGTCGGCCGCATGCAAATGCGGTCCAATCCTGAAA
GCCGGTCCCAGTTCGGACCGGGGTCTGCAACCCGACCCCGCGAAGCCGGATTCGCTAGTAATCGCGCATC
AGCCATGGCGCGGTGAATACGTTCCCGGGCCTTGTACACACCGCCCGTCAAGCCATGAAAGCCGGGGGTG
CCTGAAGTCCGTTACCGCGAGGGTCGGCCTAAGGCAAAACCGGTGATTGGGGCT
>164460384|gb|EU189008.1|Pv.113f4-12|
TGGGCGAGAGCCTGAACCAGCCAAGTAGCGTGCAGGATGACGGCCCTATGGGTTGTAAACTGCTTTTATG
CGGGGATAAAGTGAGGGACGTGTCCTTCATTGCAGGTACCGCATGAATAAGGACCGGCTAATTCCGTGCC
AGCAGCCGCGGTAATACGGAAGGTCCGGGCGTTATCCGGATTTATTGGGTTTAAAGGGAGCGTAGGCCGT
GGATTAAGCGTGTTGTGAAATGCAGGTGCTCAACGTCTGCACTGCAGCGCGAACTGGTCCACTTGAGTGT
GCGCAACGCAGGCGGAATTCGTCGTGTAGCGGTGAAATGCTTAGATATGACGAAGAACTCCGATTGCGAA
GGCAGCTTGCGGGAGCACAACTGACGCTGAAGCTCGAAAGTGCGGGTATCGAACAGGATTAGATACCCTG
GTAGTCCGCACGGTAAACGATGGATGCCCGTTGTCAGGCTGTTTCAGCCTGGTGACCAAGCGAAAGCATT
AAGCATCCCACCTGGGGAGTACGCCGGCAACGGTGAAACTCAAAGGAATTGACGGGGGCCCGCACAAGCG
GAGGAACATGTGGTTTAATTCGATGATACGCGAGGAACCTTACCCGGGCTTGAATTGCAGACGAAGGCTT
CAGAGATGAAGCGGCCCTTCGGGGCGTCTGTGAAGGTGCTGCATGGTTGTCGTCAGCTCGTGCCGTGAGG
TGTCGGCTTAAGTGCCATAACGAGCGCAACCCCTTTCTTCAGTTGCCATCAGGTGATGCTGGGCACTCTG
GAGACACTGCCACCGCAAGGTGTGAGGAAGGTGGGGATGACGTCAAATCAGCACGGCCCTTACGTCCGGG
GCTACACACGTGTTACAATGGCCGGTACAGAGAGTTGATTTTGTGCAAACACGATCTAAT
>294428|gb|L16474.1|P.oris|
TACNATGGAGAGTTTGATCCTGGCTCAGGATNAACGCTGGCTACAGGCTTAACACATGCAAGTCGAGGGG
AAACGACGGGGAAGCTTGCTTCCCCGGGCGTCGACCGGCGCACGGGTGAGTAACGCGTATCCAACCTGCC
TCTGACTGAGGGATAACCCGTCGAAAGTCGGCCTAATACCTCATGGCATCGTCTGCGGGCATCCAACGAC
GATTAAAGATTTCATCGGTCAGGGATGGGGATGCGTCTGATTAGCTTGTTGGCGGGGTAACGGCCCACCA
AGGCGNCGATCAGTAGGGGTTCTGAGAGGAAGGTCCCCCACATTGGAACTGAGACACGGTCCAAACTCCT
ACGGGAGGCAGCAGTGAGGAATATTGGTCAATGGGCGAGAGCCTGAACCAGCCAAGTAGCGTGCAGGATG
ACGGCCCTATGGGTTGTAAACTNCTTTTATGCGGGGATAAAGTGAGGGACGTGTCCTTCATTGCAGGTAC
CGCATGAATAAGGACCGGCTNATTCCGTGCCAGCAGCCGCGGTAATACGGAAGGTCCNGGCGTTATCCGG
ATTTATTGGGTTTAAAGGGAGCGTAGGCCGTGGATTAAGCGTGTTGTGAAATGCAGGTGCTCAACGTCTG
CACTGCAGCGCGAACTGGTCCACTTGAGTGTGCGCAACGCAGGCGGAATTCGTCGTGTAGCGGTGAAATG
CTTAGATATGACGAAGAACTCCGATTGCGAAGGCAGCTTGCGGGAGCACAACTGACGCTGAAGCTCGAAA
GTGCGGGTATCGAACAGGATTAGATACCCTGGTAGTCCGCACGGTAAACGATGGATGCCCGTTGTCAGGC
TGTTTCAGCCTGGTGACCAAGCGAAAGCATTAAGCATCCNACCTGGGGAGTACGCCGGCAACGGTGAAAC
TCAAAGGAATTGACGGGGGCCCGCACAAGCGGAGGAACATGTGGTTTAATTCGATGATACGCGAGGAACC
TTACCCGGGCTTGAATTGCAGACGAASGMTTCAGAGATGATGACGGCCTTCGGGGCGTCTGTGAAGGTGC

TGCATGGTTGTCGTCAGCTCGTGCCGTGAGGTGTCGGCTNAAGTGCCATAACGAGCGCAACCCNTNTCTT
CAGTTGCCATCAGGTGATGCTGGGCACTCTGGAGACACTGCCACCGCAAGGTGTGAGGAAGGTGGGGATG
ACGTCAAATCAGCACGGCCCTTACGTCCGGGGCTACACACGTGTTACAATGGCCGGTACAGAGAGTTGAT
TTTGTGCAAACACGATCTAATCCTTAAATCCGGTCCCAGTTCGGACTGGGGTCTGCAACCCGACCCCACG
AAGCTGGATTCGCTAGTAATCGCGCATCAGCCATGGCGCGGTGAATACGTTCCCGGGCCTTGTACACACC
GCCCGTCAAGCCATGAAAGCCGGGGGTGCCTGAAGTCTGTGACCGCAAGGAACGGCCTAGGGCAAAACCG
GTGATTGGGG
>164460396|gb|EU189020.1|Pv.113f4-94|
CCGCATGAATAAGGACCGGCTAATTCCGTGCCAGCAGCCGCGGTAATACGGAAGGTCCGGGCGTTATCCG
GATTTATTGGGTTTAAAGGGAGCGTAGGCCGTGGATTAAGCGTGTTGTGAAATGCAGGTGCTCAACGTCT
GCACTGCAGCGCGAACTGGTTCACTTGAGTGTGCGCAACGCAGGCGGAATTCGTCGTGTAGCGGTGAAAT
GCTTAGATATGACGAAGAACTCCGATTGCGAAGGCAGCTTGCGGGAGCACAACTGACGCTGAAGCTCGAA
AGTGCGGGTATCGAACAGGATTAGATACCCTGGTAGTCCGCACGGTAAACGATGGATGCCCGTTGTCAGG
CTGTTTCAGCCTGGTGACCAAGCGAAAGCATTAAGCATCCCACCTGGGGAGTACGCCGGCAACGGTGAAA
CTCAAAGGAATTGACGGGGGCCCGCACAAGCGGAGGAACATGTGGTTTAATTCGATGATACGCGAGGAAC
CTTACCCGGGCTTGAATTGCAGACGAAGGCTTCAGAGATGAAGAGTCCCTTCGGGGCGTCTGTGAAGGTG
CTGCATGGTTGTCGTCAGCTCGTGCCGTGAGGTGTCGGCTTAAGTGCCATAACGAGCGCAACCCCTTTCT
TCAGTTGCCATCAGGTGATGCTGGGCACTCTGTAGACACTGCCACCGCAAGGTGTGAGGAAGGTGGGGAT
GACGTCAAATCAGCACGGCCCTTACGTCCGGGGCTACACACGTGTTACAATGGCCGGTACAGAGAGTTGA
TTTTGTGCAAACACGATCTAATCCTTAAATCCGGTCCCAGTTCGGACTGGGGTCTGCAACCCGACCCCAC
GAAGCTGGATTCGCTAGTAATCGCGCATCAGCCATGGCGCGGTTAATACGTTCTCCGG
>3176095|emb|AJ006457.1|P.bryantii|
TTGAATTCGTCGACAGAGTTTGATCCTGGCTCAGGATGAACGCTAGCTACAGGCTTAACACATGCAAGTC
GAGGGGTAACATGAAGAAAGCTTGCTTTCTTTGATGACGACCGGCGCACGGGTGAGTATCGCGTATCCAA
CCTGCCCATAAGTAGGGAATAGCCTTGCGAAAGTAAGATTAATGCCCTATGGTTTCCATTAAAGACATCT
GAGATGGAATAAAGATTTATCGCTTATGGATGGGGATGCGTCTGATTAGGTAGTAGGCGGGGTAACGGCC
CACCTAGCCGACGATCAGTAGGGGTTCTGAGAGGAAGGTCCCCCACATTGGAACTGAGACACGGTCCAAA
CTCCTACGGGAGGCAGCAGTGAGGAATATTGGTCAATGGACGGAAGTCTGAACCAGCCAAGTAGCGTGCA
GGATGACGGCCCTATGGGTTGTAAACTGCTTTTTTAGGGGAATAAAGTTAGCCACGTGTGGTTATTTGCA
TGTACCCTACGAATAAGGACCGGCTAATTCCGTGCCAGCAGCCGCGGTAATACGGAAGGTCCGGGCGTTA
TCCGGATTTATTGGGTTTAAAGGGAGCGCAGGCCGTTTGGTAAGCGTGTTGTGAAATGTCCGGGCTCAAC
CTGGGCACTGCAGCGCGAACTGTCAGACTTGAGTGCACAGGAAGCGGGCGGAATTCGTGGTGTAGCGGTG
AAATGCTTAGATATCACGAAGAACTCCAATTGCGAAGGCAGCTCGCTGTAGTGTTACTGACGCTAAAGCT
CGAAAGTGCGGGTATCGAACAGGATTAGATACCCTGGTAGTCCGCACGGTAAACGATGGATGCCCGCTGT
TTGCCCTTCGGGGTGAGTGGCTAAGCGAAAGCGTTAAGCATCCCACCTGGGGAGTACGCCGGCAACGGTG
AAACTCAAAGGAATTGACGGGGGCCCGCACAAGCGGAGGAACATGTGGTTTAATTCGATGATACGCGAGG
AACCTTACCCGGGCTTGAATTGCAGATGACGGATCTAGAGATAGTGACTTCCTTCGGGACATCTGTGAAG
GTGCTGCATGGTTGTCGTCAGCTCGTGCCGTGAGGTGTCGGCTTAAGTGCCATAACGAGCGCAACCCTTC
TCTTCAGTTGCCATCAGGTAGAGCTGGGCACTCTGGAGACACTGCCACCGTAAGGTGTGAGGAAGGTGGG
GATGACGTCAAATCAGCACGGCCCTTACGTCCGGGGCTACACACGTGTTACAATGGCCGGTACAGAAAGT
CGGATGCCCGTAAGGTCATTCTAATCAAGAAAGCCGGTCCCAGTTCGGACTGAGGTCTGCAACCCGACCT
CACGAAGCTGGATTCGCTAGTAATCGCGCATCAGCCATGGCGCGGTGAATACGTTCCCGGGCCTTGTACA
CACCGCCCGTCAAGCCATGAAAGCCGGGGGTGCCTGAAGTCCGTGACCGCAAGGGTCGGCCTAGGGCAAA
ACTGGTAATTGGGGCT
>26324275|gb|AY158021.1|Pv.RS2|
AGGGTTTGATCATCGCTCAGGATGAACGCTAGCTACAGGCTTAACACATGCAAGTCGAGGGGAAACGACA
TTGGAAGCTTGCTTCCTTTGGGCGTCGACCGGCGCACGGGTGAGTAACGCGTATCCAACCTTCCCGCAAG
TAAGGGATAACCCGTAGAAATGCGGCCTAATACCTTATGTTTTCCTTAGATGGCATCTGATGAGGAACAA
AGATCCGTCGCTTGCGGATGGGGATGCGTCTGATTAGTTAGTCGGCGGGGTAACGGCCCACCGAGACGAC
GATCAGTAGGGGTTCTGAGAGGAAAGTCCCCCACATTGGAACTGAGACACGGTCCAAACTCCTACGGGAG
GCAGCAGTGAGGAATATTGGTCAATGGGCGGAAGCCTGAACCAGCCAAGTAGCGTGCAGGACGACGGCCC
TATGGGTTGTAAACTGCTTTTATGCGGGGATAAAGTGGGCCACGTGTGGCCTTTTGCAGGTACCGCATGA
ATAAGGACCGGCTAATTCCGTGCCAGCAGCCGCGGTAATACGGAAGGTCCGGGCGTTATCCGGATTTATT
GGGTTTAAAGGGAGCGCAGGCCGCCGGGCAAGCGTGTTGTGAAATGCAGTCGCTCAACGTCTGCACTGCA

GCGCGAACTGCCCAGCTTGAGTGCGCGCAACGTTGGCGGAATTCGCCGTGTAGCGGTGAAATGCTTAGAT
ATGGCGAAGAACTCCGATTGCGAAGGCAGCTGACGGGTGCGTAACTGACGCTCATGCTCGAAAGTGCGGG
TATCGAACAGGATTAGATACCCTGGTAGTCCGCACGGTAAACGATGGATGCCCGCTATTCGGCCCCTGCG
GTTGAGTGGCCAAGCGAAAGCGTTAAGCATCCCACCTGGGGAGTACGCCGGCAACGGTGAAACTCAAAGG
AATTGACGGGGGCCCGCACAAGCGGAGGAACATGTGGTTTAATTCGATGATACGCGAGGAACCTTACCCG
GGCTTGAATTGCAGCCGAACGATCCAGAGATGGTGAGGCCCTTCGGGGCGGCTGTGAAGGTGCTGCATGG
TTGTCGTCAGCTCGTGCCGTGAGGTGTCGGCTTAAGTGCCATAACGAGCGCAACCCCTCTCCCCAGTTGC
CATCGGGTAATGCCGGGCACTCCAGGGACACTGCCACCGTAAGGTGCGAGGAAGGTGGGGATGACGTCAA
ATCAGCACGGCCCTTACGTCCGGGGCTACACACGTGTTACAATGGCCGGTACAGAGAGCCGGGTCTGCGC
AAGCAGTCTCCAATCCCAAAAGCCGGCCTCAGTTCGGACTGGGGTCTGCAACCCGACCCCACGAAGCTGG
ATTCGCTAGTAATCGCGCATCAGCCATGGCGCGGTGAATACGTTCCCGGGCCTTGTACACACCGCCCGTC
AAGCCATGAAAGCCGGGGGTGCCTGAAGTCCGTGACCGCAAGGATCGGCCTAGGGCAAAACTGGTAATTG
GGGCTAAGTCGTAACAAGGTATC
>3724160|emb|AJ011683.1|P.albensis|
AGAGTTTGATCCTGGCTCAGGATGAACGCTAGCTACAGGCTTAACACATGCAAGTCGAGGGGAAACGACA
GAGAGTGCTTGCACACTTTGGGCGTCGACCGGCGAATGGGTGAGTAACGCGTATCCAACCTGCCCTTGAC
AGAGGGATAGCCCAGTGAAAACTGAATTAATACCTCATGTCCTCCTCCGACGGCATCAGACGAGGAGTAA
AGATTTATCGGTCAAGGATGGGGATGCGTCTGATTAGGTAGTAGGCGGGGTAACGGCCCACCTAGCCGAC
GATCAGTAGGGGTTCTGAGAGGAAGGTCCCCCACATTGGTACTGAGACACGGACCAAACTCCTACGGGAG
GCAGCAGTGAGGAATATTGGTCAATGGGCGGAAGCCTGAACCAGCCAAGTAGCGTGCAGGACGACGGCCC
TATGGGTTGTAAACTGCTTTTATAGGGGAATAAAGTTATCCACGTGTGGATATTTGCATGTACCCTATGA
ATAAGGACCGGCTAATTCCGTGCCAGCAGCCGCGGTAATACGGAAGGTCCGGGCGTTATCCGGATTTATT
GGGTTTAAAGGGAGCGTAGGCGGTATATTAAGCGTGTTGTGAAATGTAGGTGCTCAACATCTGACTTGCA
GCGCGAACTGGTTTACTTGAGTACGCACAACGTAGGCGGAATTCGTCGTGTAGCGGTGAAATGCTTAGAT
ATGACGAAGAACTCCGATTGCGAAGGCAGCTTACGGGAGCGCCACTGACGCTGAAGCTCGAAAGTGCGGG
TATCGAACAGGATTAGATACCCTGGTAGTCCGCACGGTAAACGATGGATGCCCGCTCTGAGTCCTTTTGG
ATTTGGGGCCAAGCGAAAGCATTAAGCATCCCACCTGGGGAGTACGCCGGCAACGGTGAAACTCAAAGGA
ATTGACGGGGGCCCGCACAAGCGGAGGAACATGTGGTTTAATTCGATGATACGCGAGGAACCTTACCCGG
GCTTGAATTGCAGGAGAACGATCCAGAGATGGTGAGGCCCTTCGGGGCTCCTGTGAAGGTGCTGCATGGT
TGTCGTCAGCTCGTGCCGTGAGGTGTCGGCTTAAGTGCCATAACGAGCGCAACCCCTCTCCTTAGTTGCC
ATCAGGTAATGCTGGGCACTCTGGGGACACTGCCACCGTAAGGTGTGAGGAAGGTGGGGATGACGTCAAA
TCAGCACGGCCCTTACGTCCGGGGCTACACACGTGTTACAATGGCGTGTACAGAGAGTTGGTGACATGCA
AATGTCATCTAATCCTAAAAGCACGTCTCAGTTCGGACTGGGGTCTGCAACCCGACCCCACGAAGCTGGA
TTCGCTAGTAATCGCGCATCAGCCATGGCGCGGTGAATACGTTCCCGGGCCTTGTACACACCGCCCGTCA
AGCCATGAAAGCCGGGGGTGCCTGAAGTCCGTGACCGGAAGGATCGGCCTAGGGCAAAACTGGTAATTGG
GGCTAAGTCGTAACAAGGTAGCCGT
>164460363|gb|EU188987.1|Pv.113f4-66|
CAATGGGCGAGAGCCTGAACCAGCCAAGTAGCGTGCAGGATGACGGCCCTCCGGGTTGTAAACTGCTTTT
AGTTGGGAATAAAAAAAGGGACTTGTCTCTTCTTGTATGTACCTTCAGAAAAAGGACCGGCTAATTCCGT
GCCAGCAGCCGCGGTAATACGGAAGGTCCAGGCGTTATCCGGATTTATTGGGTTTAAAGGGAGCGTAGGC
GGATTGTTAAGTCAGCGGTTAAAGGGTGTGGCTCAACCATGCATTGCCGTTGAAACTGGCGATCTTGAGT
GCAGACAGGGATGCCGGAATTCGTGGTGTAGCGGTGAAATGCTTAGATATCACGAAGAACTCCGATCGCG
AAGGCAGGTGTCCGGGCTGCAACTGACGCTGAGGCTCGAAAGTGTGGGTATCAAACAGGATTAGATACCC
TGGTAGTCCACACAGTAAACGATGTATACTCGCGGTTTGCGATAGACAGTAAGCCGCCAAGCGAAAGCAT
TAAGTATACCACCTGGGGAGTACGCCGGCAACGGTGAAACTCAAAGGAATTGACGGGGGCCCGCACAAGC
GGAGGAACATGTGGTTTAATTCGATGATACGCGAGGAACCTTACCCGGGCTTGAACTAACGGTGACGAAT
CTAGAGATAGATTTTTCTTCGGACACCGTTGGAGGTGCTGCATGGTTGTCGTCAGCTCGTGCCGTGAGGT
GTCGGCTTAAGTGCCATAACGAGCGCAACCCTTCTCCTCGGTTGCCATCGGGTAATGCCGGGCACTCCGT
GGACACTGCCATCGTAAGATGTGAGGAAGGTGGGGATGACGTCAAATCAGCACGGCCCTTACGTCCGGGG
CTACACACGTGTTACAATGGGGGGTACAGAAGGCCGCTACCCGGCGACGGGATGCTAA
>52222167|gb|AY738678.1|Pv.123-b-46|
TGAGGAATATTGGTCAATGGGCGAGAGCCTGAACCAGCCAAGTAGCGTGCAGGATGACGGCCCTATGGGT
TGTAAACTGCTTTTATGTGGGGATAAAGTGCGTGACGTGTCATGCATTGCAGGTACCACATGAATAAGGA
CCGGCTAATTCCGTGCCAGCAGCCGCGGTAATACGGAAGGTCCGGGCGTTATCCGGATTTATTGGGTTTA

AAGGGAGCGTAGGCTGTCTATTAAGCGTGTTGTGAAATTTACCGGCTCAACCGGTGGCTTGCAGCGCGAA
CTGGTCGACTTGAGTATGCAGGAAGTAGGCGGAATTCATGGTGTAGCGGTGAAATGCTTAGATATCATGA
CGAACTCCGATTGCGCAGGCAGCTTACTGTAGCATAACTGACGCTGATGCTCGAAAGTGCGGGTATCAAA
CAGGATTAGATACCCTGGTAGTCCGCACGGTAAACGATGGATGCTCGCTATTCGTCCTATTTGGATGAGT
GGCCAAGTGAAAACATTAAGCATCCCACCTGGGGAGTACGCCGGCAACGGTGAAACTCAAAGGAATTGAC
GGGGGCCCGCACAAGCGGAGGAACATGTGGTTTAATTCGATGATACGCGAGGAACCTTACCCGGCTTGA
ACTGCCAGCGAACGATACAGAGATGTTGAGGCCCTTCGGGGCGCTGGTGGAGGTGCTGCATGGTTGTCGT
CAGCTCGTGCCGTGAGGTGTCGGCTTAAGTGCCATAACGAGCGCAACCCTTTTCTTTAGTTGCCATCAGG
TAATGCTGGGCACTCTATGGATACTGCCACCGTAAGGTGTGAGGAAGGTGGGGATGACGTCAAATCAGCA
CGGCCCTTACGTCCGGGGCTACACACGTGTTACAATGGGGCATACAGAGTGTTGGCTTAACGCAAGTTTG
GTCTAATCTTCAAAGTGTCTCCCAGTTCGGATTGGGGTCTGCAACCCGACCCCATGAAGCTGGATTCGCT
AGTAATCGCGCATCAGCCATGGCGCGGTGAATACGTTCCCGGGCCT
>66878797|gb|AY959091.1|Pv.rRNA318|
TCGCCCTTAGAGTTTGATCCTGGCTCAGGATGAACGCTAGCTATAGGCTTAACACATGCAAGTCGAGGGG
CAGCATATAGATTGCTTGCAATTTATGATGGCGACCGGCGCACGGGTGAGTAACGCGTATCCAACCTACC
CATTACTAGGGAATAACCCAGCGAAAGTTGGCCTAATGCCCTATGTAGTCGTTTGATCGCCTGAGATTTC
GACGAAAGATTTATCGGTATTGGATGGGGATGCGTCTGATTAGCTTGTTGGCGGGGTAAAGGCCCACCAA
GGCAACGATCAGTAGGGGTTCTGAGAGGAAGGTCCCCCACATTGGAACTGAGACACGGTCCAAACTCCTA
CGGGAGGCAGCAGTGAGGAATATTGGTCAATGGGCGAGAGCCTGAACCAGCCAAGTAGCGTGCAGGATGA
CGGCCCTATGGGTTGTAAACTGCTTTTATATGGGAATAAAGTGAGGGACGTGTCCCTTATTGCATGTACC
ATATGAATAAGGACCGGCTAATTCCGTGCCAGCAGCCGCGGTAATACGGAAGGTCCAGGCGTTATCCGGA
TTTATTGGGTTTAAAGGGAGCGTAGGCTGTTTGTTAAGCGTGTTGTGAAATGTAAGAGCTCAACTTTTAG
ATTGCAGCGCGAACTGGCAGACTTGAGTGCGCACAACGTAGGCGGAATTCATGGTGTAGCGGTGAAATGC
TTAGATATCATGACGAACTCCGATTGCGAAGGCAGCTTACGGGAGCGCAACTGACGCTAAAGCTCGAAGG
TGCGGGTATCGAACAGGATTAGATACCCTGGTAGTCCGCACAGTAAACGATGGATGCCCGCTGTTAGCAC
CTAGTGTTAGCGGCTAAGCGAAAGCATTAAGCATCCCACCTGGGGAGTACGCCGGCAACGGTGAAACTCA
AAGGAATTGACGGGGGCCCGCACAAGCGGAGGAACATGTGGTTTAATTCGATGATACGCGAGGAACCTTA
CCCGGGCTTGAATTGCAGATGTTTATATCAGAGATGATATATTCCCTTCGGGGCATTTGTGAAGGTGCTG
CATGGTTGTCGTCAGCTCGTGCCGTGAGGTGTCGGCTTAAGTGCCATAACGAGCGCAACCCCTTTTTTTA
GTTGCCATCAGGTAGTGCTGGGCACTCTAGAGATACTGCCACCGTAAGGTGTGAGGAAGGTGGGGATGAC
GTCAAATCAGCACGGCCCTTACGTCCGGGGCTACACACGTGTTACAATGGGTGGTACAGAGAGTTGGTTG
TACGCAAGTGCAATCTAATCCTAAAAACCATTCTCAGTTCGGACTGGGGTCTGCAACCCGACCCCACGAA
GCTGGATTCGCTAGTAATCGCGCATCAGCCATGGCGCGGTGAATACGTTCCCGGGCCTTGTACACACCGC
CCGTCAAGCCATGAAAGCCGGGGGTGCCTGAAGTTCGTGACCGTAAGGATCGACCTAGGGCAAAACTGGT
AATTGGGGCTAAGTCGTAACAAGGTAGCCGTAAAGGGCG
>52222166|gb|AY738677.1|Pv.123-f2-42|
TGAGGAATATTGGTCAATGGGCGAGAGCCTGAACCAGCCAAGTAGCGTGCAGGATGACGGCCCTATGGGT
TGTAAACTGCTTTTATATGGGAATAAAGTGAGGGACGTGTCCCTTATTGCATGTACCATACGAATAAGGA
CCGGCTAATTCCGTGCCAGCAGCCGCGGTAATACGGAAGGTCCAGGCGTTATCCGGATTTATTGGGTTTA
AAGGGAGCGTAGGCTGTTTGTTAAGCGTGTTGTGAAATGTAAGAGCTCAACTTTTAGATTGCAGCGCGAA
CTGGCAGACTTGAGTGCGCACAACGTAGGCGGAATTCATGGTGTAGCGGTGAAATGCTTAGATATCATGA
CGAACTCCGATTGCGAAGGCAGCTTACGGGAGCGCAACTGACGCTAAAGCTCGAAGGTGCGGGTATCGAA
CAGGATTAGATACCCTGGTAGTCCGCACAGTAAACGATGGATGCCCGCTGTTAGCACCTAGTGTTAGCGG
CTAAGCGAAAGCATTAAGCATCCCACCTGGGGAGTACGCCGGCAACGGTGAAACTCAAAGGAATTGACGG
GGGCCCGCACAAGCGGAGGAACATGTGGTTTAATTCGATGATACGCGAGGAACCTTACCCGGGCTTGAAT
TGCAGATGTTTATATCAGAGATGATATATTCCCTTCGGGGCATTTGTGAAGGTGCTGCATGGTTGTCGTC
AGCTCGTGCCGTGAGGTGTCGGCTTAAGTGCCATAACGAGCGCAACCCCTTTTTTTAGTTGCCATCAGGT
AATGCTGGGCACTCTAGAGATACTGCCACCGTAAGGTGTGAGGAAGGTGGGGATGACGTCAAATCAGCAC
GGCCCTTACGTCCGGGGCTACACACGTGTTACAATGGGTGGTACAGAGAGTTGGTTGTACGCAAGTGCAA
TCTAATCCTAAAAACCATTCTCAGTTCGGACTGGGGTCTGCAACCCGACCCCACGAAGCTGGATTCGCTA
GTAATCGCGCATCAGCCATGGCGCGGTGAATACGTTCCCGGGCCT
>52222168|gb|AY738679.1|Pv.123-f-110|
TGAGGAATATTGGTCAATGGGCGAGAGCCTGAACCAGCCAAGTAGCGTGCAGGATGACGGCCCTATGGGT
TGTAAACTGCTTTTACGCGGGGATAAAGTGCGTGACGTGTCTCGCATTGCAGGTACCGCGTGAATAAGGA

CCGGCTAATTCCGTGCCAGCAGCCGCGGTAATACGGAAGGTCCGGGCGTTATCCGGATTTATTGGGTTTA
AAGGGAGCGCAGGCCGCCCGATAAGCGTGTTGTGAAATGTACCGGCTCAACCGGTGAGTTGCAGCGCGAA
CTGTCAGGCTTGAGTGCACGGTAAGCAGGCGGAATTCATGGTGTAGCGGTGAAATGCTTAGATATCATGA
GGAACTCCGATTGCGAAGGCAGCTTGCTGCAGTGCGACTGACGCTTAGGCTCGAAGGTGCGGGTATCAAA
CAGGATTAGATACCCTGGTAGTCCGCACGGTAAACGATGGATGCCCGCTGTCCGCCCATTCGTGGCGGGC
GGCCAAGCGAAAGCGTTAAGCATCCCACCTGGGGAGTACGCCGGCAACGGTGAAACTCAAAGGAATTGAC
GGGGGCCCGCACAAGCGGAGGAACATGTGGTTTAATTCGATGATACGCGAGGAACCTTACCCGGGCTTGA
ACTGCCAGTGAACGATACAGAGATGTTGAGGCCCTTCGGGGCGCTGGTGGAGGTGCTGCATGGTTGTCGT
CAGCTCGTGCCGTGAGGTGTCGGCTTAAGTGCCATAACGAGCGCAACCCCTCTCTTCAGTTGCCATCAGG
TGATGCTGGGCACTCTGGAGACACTGCCACCGCAAGGTGTGAGGAAGGTGGGGATGACGTCAAATCAGCA
CGGCCCTTACGTCCGGGGCTACACACGTGTTACAATGGGGCATACAGAGCGTCGGTTCAACGCAAGTTGG
ACCCAATCTTCAAAGTGCCTCCCAGTTCGGACTGGGGTCTGCAACCCGACCCCACGAAGCTGGATTCGCT
AGTAATCGCGCATCAGCCATGGCGCGGTGAATACGTTCCCGGGCCT
>52222174|gb|AY738685.1|Pv.136-b-40|
TGAGGAATATTGGTCAATGGGCGAGAGCCTGAACCAGCCAAGTAGCGTGCAGGATGACGGCCCTATGGGT
TGTAAACTGCTTTTATGCGGGGATAAAGTGCGTGACGTGTCATGCATTGCAGGTACCGCATGAATAAGGA
CCGGCTAATTCCGTGCCAGCAGCCGCGGTAATACGGAAGGTCCGGGCGTTATCCGGATTTATTGGGTTTA
AAGGGAGCGTAGGCCGCCAGATAAGCGTGTTGTGAAATGTACCGGCTCAACCGGTGAATTGCAGCGCGAA
CTGTTTGGCTTGAGTGCACGGTAAGCAGGCGGAATTCATGGTGTAGCGGTGAAATGCTTAGATATCATGA
AGAACTCCGATTGCGAAGGCAGCTTGCTGCAGTGCGACTGACGCTGATGCTCGAAGGTGCGGGTATCAAA
CAGGATTAGATACCCTGGTAGTCCGCACGGTAAACGATGGATGCCCGCTGTCCGCCTTTTGTGGCGGGTG
GCCAAGCGAAAGCGTTAAGCATCCCACCTGGGGAGTACGCCGGCAACGGTGAAACTCAAAGGAATTGACG
GGGGCCCGCACAAGCGGAGGAACATGTGGTTTAATTCGATGATACGCGAGGAACCTTACCCGGGCTTGAA
CTGCCAGTGAACGATACAGAGATGTTGAGGCCCTTCGGGGCGCTGGTGGAGGTGCTGCATGGTTGTCGTC
AGCTCGTGCCGTGAGGTGTCGGCTTAAGTGCCATAACGAGCGCAACCCCTTTTTTCAGTTGCCATCAGGT
AATGCTGGGCACTCTGGAGATACTGCCACCGCAAGGTGTGAGGAAGGTGGGGATGACGTCAAATCAGCAC
GGCCCTTACGTCCGGGGCTACACACGTGTTACAATGGGGCATACAGAGTGTTGGCTTAACGCAAGTTTGG
TCTAATCTTCAAAGTGTCTCCCAGTTCGGACTGGGGTCTGCAACCCGACCCCACGAAGCTGGATTCGCTA
GTAATCGCGCATCAGCCATGGCGCGGTGAATACGTTCCCGGGCCT
